# Asymmetric Synthesis of Dialkyl Carbinols by Ni‐Catalyzed Reductive‐Oxidative Relay of Distinct Alkenes

**DOI:** 10.1002/advs.202409592

**Published:** 2024-10-28

**Authors:** Quan‐Xing Zi, Wei Shu

**Affiliations:** ^1^ Guangming Advanced Research Institute Department of Chemistry and Guangdong Provincial Key Laboratory of Catalysis Southern University of Science and Technology Shenzhen Grubbs Institute Shenzhen Guangdong 518055 P. R. China; ^2^ State Key Laboratory of Coordination Chemistry Nanjing University Nanjing Jiangsu 210093 P. R. China

**Keywords:** alkene‐alkene coupling, asymmetric alkyl‐alkyl coupling, dialkyl carbinols, nickel catalysis, reductive‐oxidative relay

## Abstract

Enantioenriched unsymmetric dialkyl carbinol derivatives are of importance in natural products, bioactive molecules, and functional organic materials. However, the catalytic asymmetric synthesis of dialkyl carbinol derivatives remains challenging due to the similar steric and electronic properties of two alkyl substituents. Herein, an unprecedented synthesis of chiral dialkyl carbinol ester derivatives from Ni‐catalyzed reductive‐oxidative relay cross‐coupling of two alkenes is developed for the first time. The reaction features the use of enol esters and unactivated alkenes as two different alkyl equivalents to undergo head‐to‐tail and enantioselective alkyl‐alkyl cross‐coupling. The reaction undergoes two‐electron reduction and single electron oxidation in the presence of both reductants and oxidants. The use of an allyl bromide as single electron acceptor is crucial for the success of this non‐trivial asymmetric cross‐coupling, providing a new reaction mode for asymmetric alkyl‐alkyl bond‐forming event in the absence of stoichiometric alkyl electrophiles.

## Graphical abstract

Herein, the Ni‐catalyzed regio‐ and enantioselective alkyl‐alkyl cross‐coupling between two alkenes via a reductive and oxidative relay is reported to afford enantioenriched dialkyl carbinol esters. The reaction undergoes anti‐Markovnikov hydrometallation of enol esters and Markovnikov hydrometallation of unactivated alkenes, allowing for asymmetric alkyl‐alkyl bond formation in the absence of stoichiometric alkyl electrophiles.

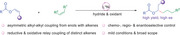

## 1 Introduction

**Figure 1:**
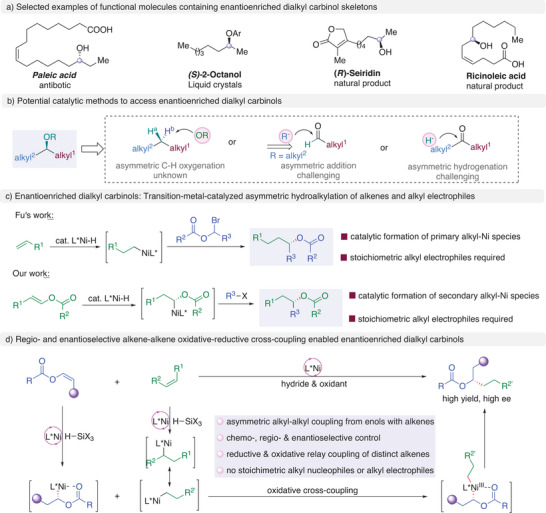
Significance and impetus for the developing of enantioenriched dialkyl carbinol derivatives of unactivated alkenes, allowing for an unprecedented asymmetric oxidative cross‐coupling of two distinct alkyl metallic species to forge alkyl–alkyl bond.

Enantioenriched unsymmetric dialkyl carbinol derivatives are of importance in natural products, pharmaceuticals, and liquid crystals (**Figure** **1**a).^[^ ^1^ ^]^ To this end, the enantioselective synthesis of dialkyl carbinol derivatives has been recognized as a long‐term yet challenging goal in chemistry community. Known protocols for catalytic asymmetric synthesis of unsymmetric dialkyl carbinol derivatives heavily relies on addition of alkyl organometallic reagents to aldehydes, hydrogenation of ketones, or enol esters, and ring‐opening of epoxides (Figure 1b).^[^ ^2^ ^]^ However, these methods are typically applied to chiral aliphatic alcohols with the stereogenic center adjacent *sp* ^2^ hybridized groups, yet construction of enantioenriched unsymmetric dialkyl carbinols remains a formidable challenge. The major challenge is attributes to the difficulty in identifying different faces of two alkyl groups on prochiral centers, which are extremely similar in steric and electronic properties. In 2020, Zhou achieved a ground‐breaking work on Ir‐catalyzed asymmetric hydrogenation of dialkyl ketones using a sophisticated PNP ligand based on spiro‐skeleton, affording enantioenriched unsymmetric dialkyl carbinols with good levels of enantioselectivity. Alternatively, transition‐metal‐catalyzed asymmetric alkyl‐alkyl cross‐coupling offers a potential alternative to access enantioenriched unsymmetric dialkyl carbinol derivatives.^[^ ^3^ ^]^ In 2016, Fu group reported a pioneer work on Ni‐catalyzed hydrometallation of alkenes to catalytically form alkyl‐metallic species for coupling with alkyl electrophiles.^[^ ^4^ ^]^ In 2018, Fu group realized the asymmetric alkyl‐alkyl coupling between alkenes and alkyl halides via an *anti*‐Markovnikov hydroalkylation of alkenes.^[^ ^5^ ^]^ The use of abundant and bench‐stable alkenes as latent alkyl nucleophiles circumvents the use of stoichiometric amount of alkyl metallic reagents.^[^ ^6^ ^]^ In 2020, Fu developed an efficient and versatile method for the direct synthesis of esters of enantioenriched dialkyl carbinols via a Ni‐ catalyzed enantioconvergent hydroalkylation of terminal alkenes with racemic *O*‐acyl α‐bromoalcohols (Figure 1c).^[^ ^7^ ^]^ In 2021, our group reported a Ni‐catalyzed asymmetric hydroalkylation of enol esters with alkyl halide (Figure 1c).^[^ ^8^ ^]^ In 2023, Gong developed a nickel‐catalyzed head‐to‐head homo‐ and cross‐dimerization of unactivated terminal alkenes to afford linear alkyl‒alkyl coupling product.^[^ ^9^ ^]^ Recently, we first discovered a new reaction mode of Ni‐catalyzed head‐to‐tail cross‐hydrodimerization of *N*‐acyl enamines with unactivated alkenes to undergo enantioenriched alkyl‐alkyl coupling to afford *N*‐acyl α‐branched aliphatic amines.^[^ ^10^ ^]^ We wonder the possibility of coupling enol esters with terminal alkenes to forge enantioenriched dialkyl carbinols, representing a new reaction mode to for the synthesis of enantioenriched dialkyl carbinols (Figure 1d). To realize this proposal, several challenges must be tackled: 1) Selective hydrometallation of different alkenes with chemo‐, regio‐ and enantioselectivity control. 2) Asymmetric alkyl‐alkyl bond‐forming via reductive and oxidative relay of alkenes. 3) Two electron hydrometallation of alkenes to form alkyl metallic species and single electron oxidative asymmetric cross‐coupling between two different alkyl metallic species. 4) parallel existence of reductants and oxidants. It is known that enol esters are less reactive compared to *N*‐acyl enamines as well as the lower stability of resulting alkyl‐Ni intermediate,^[^ ^6h^ ^]^ which imposes additional challenges for controlling the reactivity and enantioselectivity. Herein, we reported the Ni‐catalyzed regio‐ and enantioselective alkyl‐alkyl cross‐coupling between two alkenes via a reductive and oxidative relay to afford enantioenriched dialkyl carbinol esters. The reaction undergoes anti‐Markovnikov hydrometallation of enol esters and Markovnikov hydrometallation.

## 2 Results and Discussion

We started to test the feasibility of this reductive and oxidative asymmetric head‐to‐tail cross‐dimerization of alkenes by using vinyl benzoate (**1a**) and 1‐hexene (**2a**) as prototype substrates to explore the reaction conditions (**Table** **1**). After extensive evaluation of different parameters, the use of NiBr_2_·glyme as precatalyst, chiral bisoxazoline ligand, allyl bromide (**O1**) as electron acceptor, trimethoxyl silane as hydride source in the presence of cesium fluoride and sodium carbonate as promotor in a mixture of dioxane and 1,2‐dichloroethane as the standard conditions, affording the desired unsymmetric dialkyl carbinol **3a** in 81% yield with 92% ee (Table 1, entry 1). No head‐to‐head homo‐ or cross‐hydrodimerization products were observed under the reaction conditions. Ligand evaluation revealed the ligand has a significant effect on the reaction, chiral bisoxazoline ligand is the superior type of ligand for this reaction (Table 1, entries 2−11). Increasing the steric hindrance of alkyl substituents on the BOX ligand enhanced both the yield and enantioselectivity of the desired head‐to‐tail cross‐hydrodimerizaiton product (**3a**). Other tested chiral bisoxazoline ligands (**L2**‐**L11**) with different substitution patterns could catalyze this transformation, albeit in lower efficiency or enantioselectivity, indicating that ligand is crucial for this enantioselective alkene‐alkene cross‐coupling reaction. Allyl bromides proved to be essential as the electron acceptor. A wide range of acyclic allyl bromides (**O1**‐**O4**) could mediate this oxidative cross‐hydrometallation coupling reaction (Table 1, entries 12−14). Increasing steric hindrance of allyl bromides may slow down the electron transfer process between allyl bromides and alkyl‐Ni species to tune the reactivity and enantioselectivity of the reaction. Evaluation of solvent effect on the reaction revealed that both dioxane and dichloromethane are necessary to achieve excellent levels of yields and enantioselectivities (Table 1, entries 15 and 16). Using 1,4‐dioxane as solvent resulted in low yield of **3a**, probably due to the poor solubility of the reaction. The addition of dichloromethane as a cosolvent improved the solubility of the reaction to increase the yield of **3a** without erasing the enantioselectivity. Control experiments showed that no desired reaction occurred in the absence of an oxidant (Table 1, entry 17). Notably, the use of **A1** as additive significantly enhanced the reactivity of the reaction to furnish **3a** in higher yield (Table 1, entry 18), probably due to ligand exchange of **A1** with alkyl‐nickel intermediates to enhance the reactivity of the reaction to facilitate the desired cross‐coupling reaction.^[^ ^11^ ^]^ The combination of sodium carbonate with cesium fluoride is required to furnish **3a** in good yield with excellent level of enantioselectivity (Table 1, entries 19 and 20).

**Table 1:** Condition evaluation for the reaction.

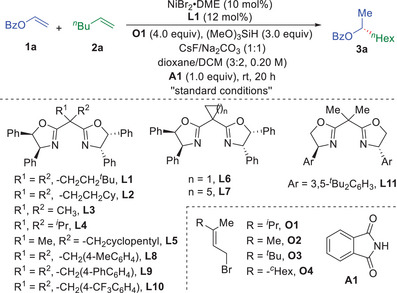			
Entry^a)^	variation from “standard conditions”	yield (3a)	ee (3a)
1	none	81% (74%)	92%
2	**L2** instead of **L1**	86%	85%
3	**L3** instead of **L1**	61%	78%
4	**L4** instead of **L1**	83%	73%
5	**L5** instead of **L1**	80%	90%
6	**L6** instead of **L1**	40%	72%
7	**L7** instead of **L1**	77%	66%
8	**L8** instead of **L1**	25%	63%
9	**L9** instead of **L1**	27%	61%
10	**L10** instead of **L1**	72%	73%
11	**L11** instead of **L1**	76%	90%
12	**O2** instead of **O1**	73%	81%
13	**O3** instead of **O1**	80%	91%
14	**O4** instead of **O1**	68%	91%
15	dioxane as solvent	67%	92%
16	DCM as solvent	41%	81%
17	no **O1**	N.R.	−
18	no **A1**	62%	95%
19	no CsF	80%	88%
20	no Na_2_CO_3_	34%	92%

^a)^ The reaction was conducted using **1a** (0.1 mmol) and **2a** (0.4 mmol) in the presence of (MeO)_3_SiH (0.3 mmol), Na_2_CO_3_ (0.2 mmol), CsF (0.2 mmol), **O1** (0.4 mmol) and **A1** (0.1 mmol) in 0.5 mL of solvent under indicated conditions for 20 h unless otherwise stated. Yield was determined by GC using *n*‐dodecane as an internal standard. Isolated yield is shown in the parentheses. The enantiomeric excess was determined by HPLC using a chiral stationary phase. ee = enantiomeric excess. N.R. = No reaction. DCM = Dichloromethane.

**Figure 2:**
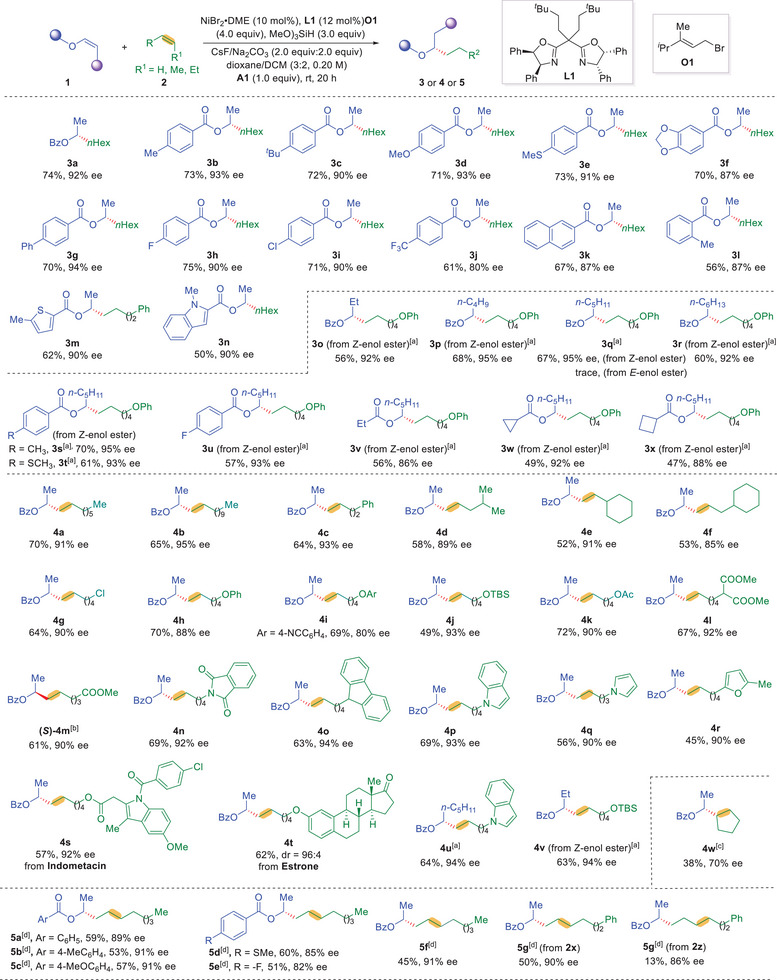
Standard conditions, see Table 1 for details. Isolated yield is shown on 0.2 mmol scale. Enantiomeric excess (ee) was determined by chiral HPLC analysis. [a] **L11** (12 mol%), CsF/KHCO_3_ (2.0 equiv: 3.0 equiv), (MeO)_3_SiH (4.0 equiv), **O1** (4.0 equiv), Et_2_O/TCE (3/2) 0.25 m, **A1** (1.6 equiv), 50 °C, 20 h, from (*Z*)‐enol ester. [b] (*R*,*R”*,*S*,*S”*)‐**L1** was used instead of (*S*,”*S*,*R*,”*R*)‐**L1**. [c] The reaction was conducted under standard conditions using **L2** instead of **L1**. Yield based on the recovery of **1a**. [d] The reaction was conducted using (MeO)_2_MeSiH instead of (MeO)_3_SiH in 2‐MeTHF/DCM (2/3). TCE = 1.1.2‐Trichloroethane. 2‐MeTHF = 2‐Methyltetrahydrofuran.

With the optimized reaction conditions in hand, the scope of enol esters was investigated (**Figure** **2**). Terminal enol benzoates are well‐tolerated under the reaction conditions to undergo tail‐to‐head reductive oxidative cross‐dimerization reaction with 1‐hexene, delivering enantioenriched α‐methyl branched alcohol esters in good yields and excellent levels of enantioselectivity (**3a**‐**3n**). Benzoates bearing electron‐donating or electron‐withdrawing substituents were all good substrates for the reaction, delivering enantioenriched unsymmetric dialkyl carbinol derivatives (**3b**‐**3l**) in 56–75% yields with excellent enantioselectivity (80%‐94% ee). Notably, enol benzoates with halogens were good substrates for the reaction, delivering corresponding dialkyl carbinol derivatives (**3h** and **3i**) in 71% and 75% yields with 90% ee. Sterically demanding enol benzoates underwent the desired transformation to give (**3l**) in 56% yield with 87% ee. Heteroaryl carboxylic acids, including thiophenes and indoles, derived enolates reacted smoothly with unactivated alkenes to furnish corresponding products (**3m** and **3n**) in 62% and 50% yields with 90% ee. It is noteworthy that more challenging *Z*‐internal enol esters with different aliphatic substituents were successfully involved in the reaction, delivering more structurally diversed dialkyl carbinol derivatives (**3o**‐**3r**) with various substituted patterns in 56–68% yields with 92–95% ee. Moreover, internal Z‐enol esters derived from different benzoic acids or aliphatic acids furnished corresponding dialkyl carbinols (**3s‐3x**) in good yields with excellent levels of enantioselectivity. Unfortunately, *E*‐configuration of internal enol esters gave trace amounts of desired product (**3q**), probably due to the increased steric hindrance of *E*‐configuration.

Next, we turned to investigate the scope of unactivated alkenes (Figure 2). 1‐Alkyl substituted unactivated alkenes (**4a** and **4b**) could be smmothly converted to corresponding products in 70% and 65% yield with 91% and 95% ee, respectively. Terminal unactivated alkenes bearing remote aryl groups could transformed to desired compound **4c** in 64% yield with 93% ee. Branched unactivated alkenes with more steric hindrance were also found to be good substrates under the reaction conditions, giving corresponding coupling product (**4d**‐**4f**) in 52%‐58% yields with 85–91% ee. Chloride tethered alkenes were compatible in the reaction, delivering the corresponding product (**4g**) in 64% yield with 90% ee. Unactivated alkenes containing ethers and esters could also be involved in this reaction (**4h**‐**4** **m**). Unactivated alkenes with pendant aryl and silyl ethers were good substrates, giving corresponding chiral dialkyl carbinol derivatives (**4h**‐**4j**) in 49–70% yields with 80–93% ee. Different esters were tolerated under standard conditions, delivering corresponding products (**4k**‐**4** **m**) in 61%‐72% yields with 90%‐92% ee. Alkenes containing heterocycles, such as phthalimides, carbazoles, indoles, pyrroles, and furans, were compatible under the reaction conditions, delivering desired products (**4n**‐**4r**) in synthetic useful yields with 90%‐94% ee. Notably, this regio‐ and enantioselective alkene‐alkene cross‐coupling protocol could be successfully applied to late‐stage modification of complex molecules including natural products and drug molecules. Indomethacin and Estrone derived unactivated alkenes were successfully transformed to corresponding products (**4s** and **4t**) in 57% and 62% yields with

92% ee and 96:4 dr. It is worth mentioning that cyclopentene could also couple with **1a** to give desired product **4w**, albeit in lower efficiency and enantioselectivity. Furthermore, unactivated 2‐alkenes can also be applied to this reaction, giving desired product (**5a**‐**5** **g**) in a migratory manner in 45–60% yields with 82–91% ee. 1‐Pheny‐3‐hexene could also be applied to the reaction conditions to deliver **5** **g** in 13% yield with 86% ee. The absolute configuration of the products was determined to be *R* by comparing with the reported data in literature.^[^ ^1^, ^7^, ^12^ ^]^ Unfortunately, styrenes are not suitable for this reaction, probably due to the mismatched reactivity of styrenes with enol esters.

**Figure 3:**
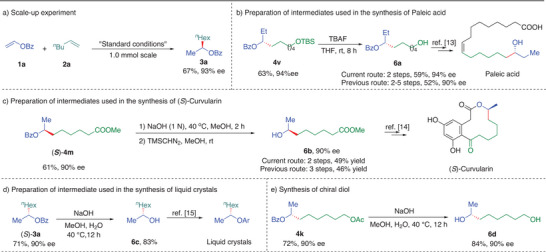
Scale‐up experiment and synthetic applications.TBAF = Tetrabutylammonium fluoride. TMSCHN2 = (Trimethylsilyl)diazomethane.

To demonstrate the robustness of this regio‐ and enantioselective alkene‐alkene cross‐coupling protocol, we evaluated the scalability of the reaction which proceeds smoothly on 1.0 mmol scale to afford the desired product **3a** in 67% yield with 93% ee (**Figure** **3**a). Next, a series of applications were conducted to demonstrate the synthetic potential of this regio‐ and enantioselective alkene‐alkene cross‐coupling protocol. First, benzoate ester of dialkyl alcohol (**6a**), an intermediate for the synthesis of paleic acid, an antimicrobial agent that is effective Mannheimia and Pasteurella, was obtained in 93% yield with 94% ee in one step from **3z** (Figure 3b),^[^ ^1^, ^7^, ^13^ ^]^ Next, selective hydrolysis of (*S*)‐**4** **m** gave hydroxy ester (*S*)‐**6b**, an intermediate in the synthesis of *S*‐curvularin, an antimicrobial agent that is effective antimicrobial activities, can be generated from commercially available starting materials in two steps in 49% yield with 90% ee through our catalytic asymmetric cross‐coupling of alkenes (Figure 3c).^[^ ^14^ ^]^ Moreover, (*S*)‐2‐octanol (**6c**), an intermediate in the synthesis of liquid crystal material, was synthesized from (*S*)‐**3a** by hydrolysis (Figure 3d).^[^ ^15^ ^]^ In addition, the product **4k** from this protocol was easily converted to enantioenriched dialkyl diol (6d) in 84% yield with 90% ee, which was difficult to access following traditional methods (Figure 3e).

**Figure 4:**
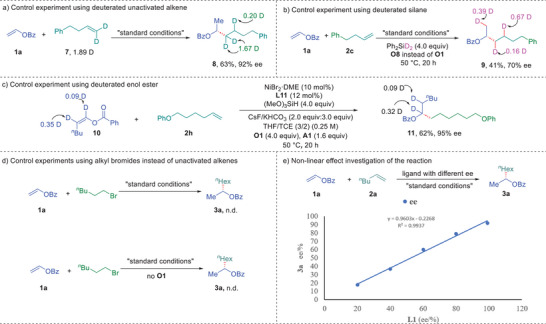
Control experiments and mechanistic studies.

To gain some insight into the mechanism of this reaction, a series of control experiments were carried out to probe the reaction mechanism (**Figure** **4**). First, deuterium labeling experiments with different sets of substrates were conducted (Figure 4a–c). Using *d*2‐4‐phenyl‐1‐butene (**7**) as unactivated alkene to react with 1a under standard conditions afforded **8** in 63% yield with 92% ee. Deuterium migration was observed on the fragment of **7**, suggesting that nickel hydride insertion onto unactivated alkenes was reversible (Figure 4a). The reaction of **1a** with 4‐phenyl‐1‐butene using deuterated silane (Ph_2_SiD_2_) instead of (MeO)_3_SiH under otherwise identical to standard conditions gave deuterated product (**9**) in 41% yield (Figure 4b). The distribution of deuterium further proved the insertion of nickel hydride onto unactivated alkenes was reversible, yet indicated the insertion of nickel hydride onto enol esters was irreversible. This rationalization was further proved by the reaction of deuterated enol ester (**10**) with an aliphatic alkene (**2h**), furnishing desired cross‐coupling product (**11**) in 62% yield with 95% ee without deuterium migration. The results revealed that insertion of nickel hydride to enol esters to form secondary alkyl‐Ni species might be irreversible and enantio‐determining (Figure 4c). Next, control experiments of vinyl benzoate (**1a**) with 1‐bromohexane under standard conditions with or without oxidant (**O1**) were conducted (Figure 4d). No desired hydroalkylation product (**3a**) was detected, excluding the possibility of forming alkyl bromides as reaction intermediates to further undergo hydroalkylation of enol benzoates. Next, time course of the reaction of **1a** with **2a** was monitored under standard conditions (Figure S5, Supporting Information). The yield increased over time while the enantiomeric excess maintained the same level over the reaction course. This result ruled out a kinetic resolution mechanism for the reaction. Furthermore, nonlinear effect of the reaction was investigated using **1a** and **2a** (Figure 4e). The enantiomeric excess of correlates with the enantiomeric excess of chiral ligand, suggesting the involvement of a single metallic center for the enantio‐determining step. Finally, we carried out kinetic studies for each reaction components to determine the turnover‐limiting step of the reaction (Figures S11, S17, S22, S28, and S34, Supporting Information). The combination of nickel catalyst and ligand was determined as first‐order‐dependent, while vinyl esters (**1a**), unactivated alkene (**2a**), oxidant, and (MeO)_3_SiH were determined as zero‐order‐ dependent. The results indicated that only catalyst was involved in the turnover‐limiting step, suggesting the generation of Ni−H is likely to be the turnover‐limiting step.

**Figure 5:**
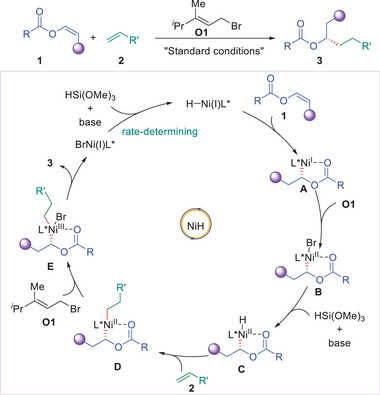
Proposed mechanism for the reaction.

Based on experimental results and literature precedence,^[^ ^6^, ^10^ ^]^ a plausible mechanism for this reaction is proposed and depicted in **Figure** **5**. First, L^*^Ni(I)−H species could be formed in the presence of a base and a silane.^[^ ^6j^ ^]^ L^*^Ni(I)−H could undergo regioselective insertion onto alkenes (**1**) with the assistance of coordination of the carbonyl group of **1** to form enantioenriched secondary alkyl‐Ni(I)L^*^ species (**A**).^[^ ^6i^ ^]^ Alkyl‐Ni(I)L^*^ (**A**) was transformed into alkyl‐Ni(II)L^*^−Br (**B**) via single electron oxidative addition with (*E*)‐1‐bromo‐3,4‐dimethylpent‐2‐ene (**O1**), which could further undergo transmetalation with silane to form alkyl‐Ni(II)L^*^‐H hydride intermediate (**C**).^[^ ^6^, ^9^ ^]^ The intermediate **C** would undergo migratory insertion onto unactivated alkenes (**2**) to give dialkyl Ni(II) species (**D**).^[^ ^6i–k^ ^]^ Ni(II) (**D**) was transformed into Ni(III) (**E**) via single electron oxidative addition with (*E*)‐1‐bromo‐3,4‐dimethylpent‐2‐ene (**O1**), which affords the desired products (**3**) and BrNi(I)L^*^ via reductive elimination.^[^ ^6^, ^9^ ^]^ L^*^Ni(I)−H could be regenerated from BrNi(I)L^*^ in the presence of a base and a silane to close the catalytic cycle.

## 3 Conclusions

In summary, an unprecedented synthesis of chiral dialkyl carbinol ester derivatives from Ni‐catalyzed reductive‐oxidative cross‐coupling of two alkenes has been developed for the first time. The reaction features the use of enol esters and unactivated alkenes to undergo head‐to‐tail and enantioselective alkyl‐alkyl cross‐coupling, opening a new avenue to fulfill asymmetric alkyl‐alkyl bond‐forming event. The reaction undergoes two‐electronreduction and single‐electron oxidation in the presence of both reductants and oxidants. The use of an allyl bromide as a single electron acceptor is crucial for the success of this non‐trivial asymmetric cross‐coupling, offering a reaction mode for enantioselective alkyl‐alkyl cross‐coupling in the absence of stoichiometric alkyl nucleophiles or alkyl electrophiles. Further work on this reaction mode is undergoing in our laboratory and will be reported in due course.

## Conflict of Interest

The authors declare no conflict of interest.

## Supporting information



Supporting Information

## Data Availability

The data that support the findings of this study are available in the supplementary material of this article.
