# Supplementary material for: Asymmetric Synthesis of Dialkyl Carbinols by Ni‐Catalyzed Reductive‐Oxidative Relay of Distinct Alkenes
Source: Adv Sci (Weinh). 2024 Oct 28;11(48):2409592. doi: 10.1002/advs.202409592 (PMC11672252; doi:10.1002/advs.202409592)
Supplement: Supplementary file 1 — Supporting Information [file ADVS-11-2409592-s001.pdf]

ADVANCED SCIENCE
Open Access

Supporting Information

for *Adv. Sci.*, DOI 10.1002/advs.202409592

Asymmetric Synthesis of Dialkyl Carbinols by Ni-Catalyzed Reductive-Oxidative Relay of Distinct Alkenes

*Quan-Xing Zi and Wei Shu**

Supporting Information

for

Asymmetric Synthesis of Dialkyl Carbinols by Ni-Catalyzed Reductive-Oxidative Relay of Distinct Alkenes

Quan-Xing Zi[a] and Wei Shu[a,b,*]

[a] *Guangming Advanced Research Institute, Shenzhen Grubbs Institute, Department of Chemistry, and Guangdong Provincial Key Laboratory of Catalysis, Southern University of Science and Technology, Shenzhen 518055, Guangdong, P.R. China*

[b] *State Key Laboratory of Coordination Chemistry, Nanjing University, Nanjing 210093, Jiangsu, P. R. China*

*E-mail: shuw@sustech.edu.cn

Table of Contents

I. General Information S2

II. Preparation of Chiral Ligands S3

III. Preparation of Substrates and Oxidants S10

IV. Optimization of Reaction Conditions S26

V. Experimental Procedure and Characterization Data S34

VI. Scale-up Experiment and Synthetic Application...S60

VII. Assignment of Absolute Configuration of the Prodcuts S65

VIII. Mechanistic Experiments..S66

IX. References..S100

X. ^{1}H NMR, ^{13}C NMR and ^{19}F NMR Spectra S103

XI. HPLC Traces S166

I. General Information

General remarks: NMR spectra were recorded on 400 MHz or 600MHz Bruker spectrometers. Chemical shifts are given in ppm. The spectra are calibrated to the residual ^{1}H and ^{13}C signals of the solvents. Multiplicities are abbreviated as follows: singlet (s), doublet (d), triplet (t), quartet (q), doublet-doublet (dd), quintet (quint), septet (sept), multiplet (m), and broad single (bs). High-pressure liquid chromatography (HPLC) was performed on Agilent 1200 Series chromatographs using a chiral column (25 cm) as noted for each compound. Enantiomer excess was determined by HPLC analysis employing Darcel Chiracel OD-H, AD-H, IA and OJ-H column. High-resolution electrospray ionization and electronic impact mass spectrometry was performed on a Thermo Scientific Q Exactive mass spectrometer (mass analyzer type: Orbitrap). A mass accuracy $\leq$ 2 ppm was obtained in the peak matching acquisition mode by using a solution containing 2<lPEG200, 2<lPPG450, and 1.5 mg NaOAc (all obtained from Sigma-Aldrich, CH-Buchs) dissolved in 100 mL MeOH (HPLC Supra grade, Scharlau, E-Barcelona) as internal standard.

Materials: Unless otherwise noted, commercial reagents were purchased from Energy Chemical Limited, J&K, Adamas-beta®, Aladdin, Macklin Reagent, Bidepharm, Meryer and used directly without further purification. DCM, TCE, Dioxane were distilled over CaH_2, stored under nitrogen atmosphere.

II. Preparation of Chiral Ligands

L1-L4, L6-L10, L12 were synthesized following reported method.[1]

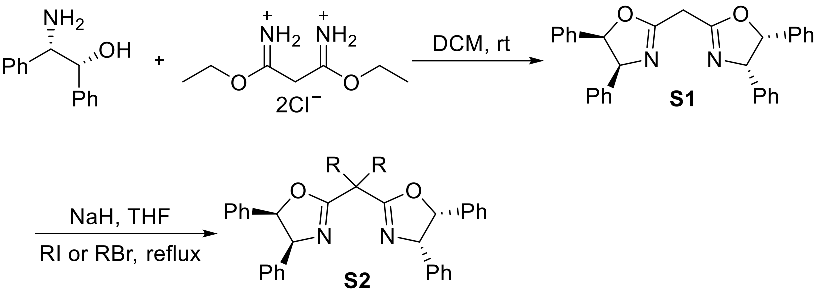

General procedure (A): A mixture of (1*R*, 2*S*)-2-amino-1,2-diphenylethan-1-ol (1.0 equiv) and diethyl malonimidate dihydrochloride (1.0 equiv) in CH_2Cl_2 (0.1 M) was stirred at room temperature for 24 hours. Water was added and the organic layer was separated, dried over Na_2SO_4, filtered and concentrated in vacuo. The residue was purified by chromatography on silica gel to give compound **S1** as a white solid in 83% yield.

To a stirred solution of **S1** (1.0 equiv) in THF (0.1 M) was added NaH (7.0 equiv) at 0 °C, and the mixture was kept at the same temperature for 0.5 hour. Then alkyl bromide or alkyl iodide (3.0 equiv) was added dropwise. The resulting mixture was warmed to room temperature and stirred for 12 hours at 80 °C. The mixture was quenched with water and extracted with dichloromethane. The organic layer was washed with brine, dried over Na_2SO_4, filtered and concentrated in vacuo. The residue was purified by chromatography on silica gel to give compound **S2** as a white solid in 80% yield.

(4*S*,4'*S*,5*R*,5'*R*)-2,2'-(2,2,8,8-Tetramethylnonane-5,5-diyl)bis(4,5-diphenyl-4,5-dihydrooxazole) (L1)

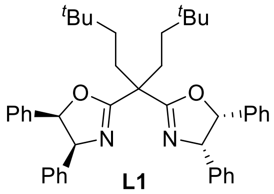

L1

Following general procedure (A), **L1** was obtained as white solid in 70% yield.

^{1}H NMR (600 MHz, $CDCl_3$) δ 7.16 – 6.84 (m, 20H), 5.94 (d, *J* = 10.1 Hz, 2H), 5.61 (d, *J* = 10.1 Hz, 2H), 2.41 (td, *J* = 13.5, 4.1 Hz, 2H), 2.25 (td, *J* = 13.5, 4.6 Hz, 2H), 1.45 (td, *J* = 13.2, 4.6 Hz, 2H), 1.35 (td, *J* = 13.2, 4.2 Hz, 2H), 1.02 (s, 18H).

^{13}C NMR (151 MHz, $CDCl_3$) δ 169.4, 137.5, 136.2, 128.1, 127.70, 127.69, 127.5, 127.0, 126.8, 86.3, 73.8, 46.8, 37.9, 30.4, 29.6, 27.3.

HRMS (ESI-TOF) Calcd for $C_{43}H_{51}N_2O_2$ $(M+H)^+$ 627.3945. Found 627.3948.

(4*S*,4'*S*,5*R*,5'*R*)-2,2'-(1,5-Dicyclohexylpentane-3,3-diyl)bis(4,5-diphenyl-4,5-dihydrooxazole) (L2)

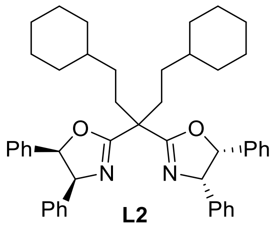
L2

Following general procedure (A), **L2** was obtained as white solid in 67% yield.
^{1}H NMR (600 MHz, $CDCl_3$) δ 7.10 – 6.86 (m, 20H), 5.93 (d, *J* = 10.2 Hz, 2H), 5.59 (d, *J* = 10.2 Hz, 2H), 2.41 (td, *J* = 13.4, 4.1 Hz, 2H), 2.32 – 2.23 (m, 2H), 1.85 (d, *J* = 13.7 Hz, 4H), 1.78 – 1.70 (m, 4H), 1.69 – 1.63 (m, 2H), 1.44 – 1.31 (m, 6H), 1.28 – 1.19 (m, 6H), 1.06 – 0.95 (m, 4H).
^{13}C NMR (151 MHz, $CDCl_3$) δ 169.4, 137.6, 136.2, 128.1, 127.7, 127.5, 127.0, 126.8, 86.3, 73.8, 46.9, 38.2, 33.8, 33.6, 31.8, 30.1, 26.8, 26.5.
HRMS (ESI-TOF) Calcd for $C_{47}H_{55}N_2O_2$ $(M+H)^+$ 679.4258. Found 679.4259.

(4*S*,4'*S*,5*R*,5'*R*)-2,2'-(Propane-2,2-diyl)bis(4,5-diphenyl-4,5-dihydrooxazole) (L3)[2]

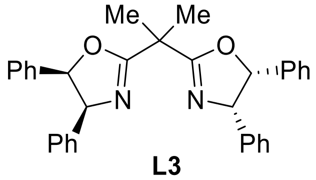
L3

Following general procedure (A), **L3** was obtained as white solid in 72% yield.
^{1}H NMR (400 MHz, $CDCl_3$) δ 7.05 – 6.94 (m, 20H), 5.97 (d, *J* = 10.1 Hz, 2H), 5.60 (d, *J* = 10.1 Hz, 2H), 1.92 (s, 6H).
^{13}C NMR (101 MHz, $CDCl_3$) δ 170.6, 137.6, 136.3, 128.0, 127.8, 127.7, 127.5, 127.1, 126.7, 86.4, 73.9, 39.7, 24.9.

(4*S*,4'*S*,5*R*,5'*R*)-2,2'-(2,4-Dimethylpentane-3,3-diyl)bis(4,5-diphenyl-4,5-dihydrooxazole) (L4)

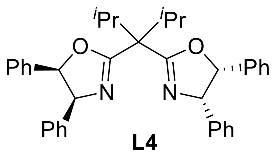
L4

Following general procedure (A), **L4** was obtained as white solid in 68% yield.
^{1}H NMR (400 MHz, $CDCl_3$) δ 7.06 – 6.86 (m, 20H), 5.99 (d, *J* = 10.6 Hz, 2H), 5.65 (d, *J* = 10.6 Hz, 2H), 2.98 – 2.91 (m, 2H), 1.36 (d, *J* = 6.7 Hz, 6H), 1.21 (d, *J* = 6.8 Hz, 6H).
^{13}C NMR (101 MHz, $CDCl_3$) δ 167.1, 138.0, 136.4, 128.2, 127.7, 127.6, 127.3, 127.1, 126.9, 85.2, 73.8, 55.6, 32.2, 20.6, 17.8.
HRMS (ESI-TOF) Calcd for $C_{37}H_{39}N_2O_2$ $(M+H)^+$ 543.3006. Found 543.3011.

(4*S*,4'*S*,5*R*,5'*R*)-2,2'-(Cyclopropane-1,1-diyl)bis(4,5-diphenyl-4,5-dihydrooxazole) (L6)[3]

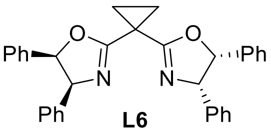

Following general procedure (A), **L6** was obtained as white solid in 71% yield.
^{1}H NMR (400 MHz, $CDCl_3$) δ 7.05 – 6.97 (m, 20H), 5.96 (d, *J* = 10.2 Hz, 2H), 5.60 (d, *J* = 10.2 Hz, 2H), 1.86 – 1.80 (m, 2H), 1.80 – 1.76 (m, 2H).
^{13}C NMR (101 MHz, $CDCl_3$) δ 167.2, 137.8, 136.5, 128.0, 127.8, 127.7, 127.5, 127.0, 126.7, 86.2, 74.0, 19.1, 15.9.

(4*S*,4'*S*,5*R*,5'*R*)-2,2'-(Cyclohexane-1,1-diyl)bis(4,5-diphenyl-4,5-dihydrooxazole) (L7)

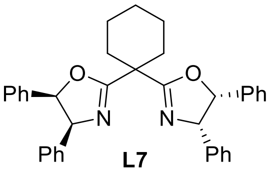

Following general procedure (A), **L7** was obtained as white solid in 64% yield.
^{1}H NMR (400 MHz, $CDCl_3$) δ 7.01 – 7.03 (m, 10H), 7.01 – 6.87 (m, 10H), 5.95 (d, *J* = 10.3 Hz, 2H), 5.65 (d, *J* = 10.3 Hz, 2H), 2.58 – 2.36 (m, 4H), 1.93 – 1.77 (m, 4H), 1.67 – 1.62 (m, 2H).
^{13}C NMR (101 MHz, $CDCl_3$) δ 169.5, 137.9, 136.6, 128.1, 127.73, 127.69, 127.4, 127.0, 126.7, 85.9, 74.1, 44.4, 32.9, 25.6, 23.0.
HRMS (ESI-TOF) Calcd for $C_{36}H_{35}N_2O_2$ $(M+H)^+$ 527.2693. Found 527.2689.

(4*S*,4'*S*,5*R*,5'*R*)-2,2'-(1,3-Di-*p*-tolylpropane-2,2-diyl)bis(4,5-diphenyl-4,5-dihydrooxazole) (L8)[1a]

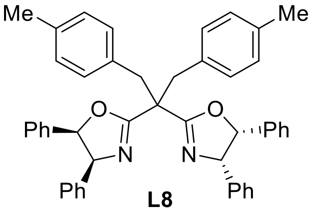

Following general procedure (A), **L8** was obtained as white solid in 67% yield.
^{1}H NMR (400 MHz, $CDCl_3$) δ 7.41 – 7.43 (m, 4H), 7.24 – 7.15 (m, 4H), 7.03 – 6.97 (m, 6H), 6.96 – 6.83 (m, 14H), 5.79 (dd, *J* = 10.3, 1.7 Hz, 2H), 5.43 (dd, *J* = 10.4, 1.5 Hz, 2H), 3.83 (dd, *J* = 14.1, 1.8 Hz, 2H), 3.52 (dd, *J* = 14.1, 1.9 Hz, 2H), 2.40 (s, 6H).
^{13}C NMR (151 MHz, $CDCl_3$) δ 168.2, 137.5, 136.6, 136.0, 133.9, 130.8, 129.1, 128.1, 127.6, 127.5, 127.1, 127.0, 86.3, 73.7, 49.6, 40.1, 21.3.

(4*S*,4'*S*,5*R*,5'*R*)-2,2'-(1,3-Di([1,1'-biphenyl]-4-yl)propane-2,2-diyl)bis(4,5-diphenyl-4,5-dihydrooxazole) (L9)

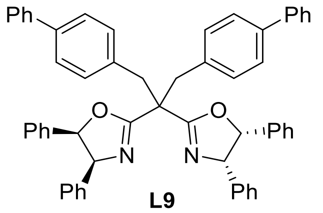

L9

Following general procedure (A), **L9** was obtained as white solid in 69% yield.
^{1}H NMR (400 MHz, $CDCl_3$) δ 7.72 – 7.57 (m, 12H), 7.52 – 7.45 (m, 4H), 7.41 – 7.35 (m, 2H), 7.07 – 6.73 (m, 20H), 5.83 (d, *J* = 10.2 Hz, 2H), 5.47 (d, *J* = 10.2 Hz, 2H), 3.96 (d, *J* = 14.1 Hz, 2H), 3.65 (d, *J* = 14.1 Hz, 2H).
^{13}C NMR (101 MHz, $CDCl_3$) δ 168.0, 141.0, 140.0, 137.4, 136.0, 135.8, 131.4, 129.0, 128.1, 127.70, 127.67, 127.5, 127.4, 127.2, 127.08, 127.05, 86.4, 73.7, 49.6, 40.4.
HRMS (ESI-TOF) Calcd for $C_{57}H_{47}N_2O_2$ $(M+H)^+$ 791.3632. Found 791.3631.

(4*S*,4'*S*,5*R*,5'*R*)-2,2'-(1,3-Bis(4-(trifluoromethyl)phenyl)propane-2,2-diyl)bis(4,5-diphenyl-4,5-dihydrooxazole) (L10)[1a]

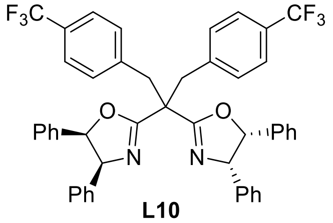

L10

Following general procedure (A), **L10** was obtained as white solid in 59% yield.
^{1}H NMR (400 MHz, $CDCl_3$) δ 7.71 – 7.59 (m, 5H), 7.47 – 7.39 (m, 3H), 7.03 – 6.85 (m, 20H), 5.88 – 5.64 (m, 2H), 5.54 – 5.39 (m, 2H), 3.98 – 3.80 (m, 2H), 3.65 – 3.47 (m, 2H).
^{13}C NMR (101 MHz, $CDCl_3$) δ 167.4, 140.9, 137.1, 135.4, 131.2, 129.7 (q, J_{C-F} = 32.2 Hz), 128.0, 127.8, 127.7, 127.3, 127.0, 125.7, 125.3 (q, J_{C-F} = 4.0 Hz), 123.0 (q, J_{C-F} = 273.1 Hz), 86.6, 73.7, 49.3, 41.4.

(4*S*,4'*S*,5*R*,5'*R*)-2,2'-(pentane-3,3-diyl)bis(4,5-diphenyl-4,5-dihydrooxazole) (L12)[4]

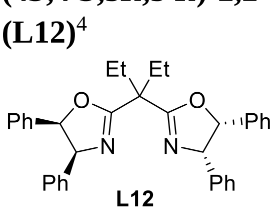

L12

Following general procedure (A), **L12** was obtained as white solid in 70% yield.
^{1}H NMR (600 MHz, $CDCl_3$) δ 7.07 – 6.90 (m, 20H), 5.95 (d, *J* = 10.2 Hz, 2H), 5.60 (d, *J* = 10.2 Hz, 2H), 2.54 – 2.40 (m, 2H), 2.39 – 2.28 (m, 2H), 1.14 (t, *J* = 7.5 Hz, 6H).
^{13}C NMR (151 MHz, $CDCl_3$) δ 169.1, 137.6, 136.2, 128.0, 127.7, 127.5, 127.0, 126.8, 86.2, 73.8, 47.7, 25.9, 8.9.

Procedure for the synthesis of L5[3]

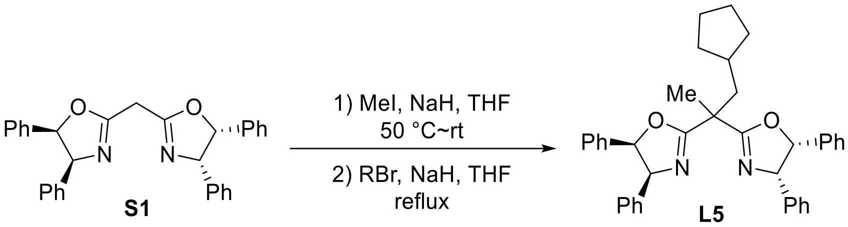

To a solution of compound **S1** (1.0 equiv) in anhydrous THF (20.0 mL) was added NaH (60%) (1.05 equiv) at rt. The resulting orange solution was stirred for another 30 min at 50 °C. Then the solution was removed to ice-bath and MeI (1.0 equiv) was added at 0°C, then washed with 5 mL THF. 10 min later, the solution was warmed to room temperature and stirred for another 1 h. The color of the mixture was turned to yellow. NaH (1.5 equiv) was then added to the mixture. After 1 h, alkyl bromide (1.0 equiv) was added to the mixture, then washed with 5 mL more THF. The reaction was stirred at 80 °C for 12 h (monitoring by thin-layer chromatography). The mixture was quenched with NH_4Cl, extracted with CH_2Cl_2. The organic layer was dried over anhydrous Na_2SO_4 and filtrated. The filtrate was concentrated under reduced pressure. The residue was purified by chromatography on silica gel to give compound **L5** as a white solid in 57% yield.

(4*S*,4'*S*,5*R*,5'*R*)-2,2'-(1-Cyclopentylpropane-2,2-diyl)bis(4,5-diphenyl-4,5-dihydrooxazole) (L5)

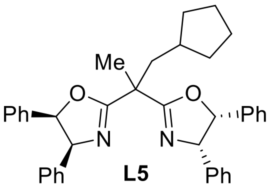

^{1}H NMR (400 MHz, $CDCl_3$) δ 7.12 – 6.85 (m, 20H), 5.96 (dd, *J* = 10.1, 3.9 Hz, 2H), 5.59 (dd, *J* = 10.1, 1.9 Hz, 2H), 2.54 (dd, *J* = 14.1, 7.5 Hz, 1H), 2.41 (dd, *J* = 14.2, 5.0 Hz, 1H), 2.19 – 2.07 (m, 1H), 2.01 (m, 2H), 1.93 (s, 3H), 1.76 – 1.52 (m, 4H), 1.37 – 1.25 (m, 2H).

^{13}C NMR (101 MHz, $CDCl_3$) δ 170.7, 169.9, 137.7, 137.6, 136.4, 136.2, 128.1, 128.0, 127.68, 127.74, 127.5, 127.4, 127.1, 127.0, 126.8, 126.8, 86.4, 86.3, 74.0, 73.8, 43.5, 42.7, 36.7, 34.9, 33.7, 25.5, 25.0, 22.4.

HRMS (ESI-TOF) Calcd for $C_{38}H_{39}N_2O_2$ $(M+H)^+$ 555.3006. Found 555.3009.

Procedure for the synthesis of L11[5]

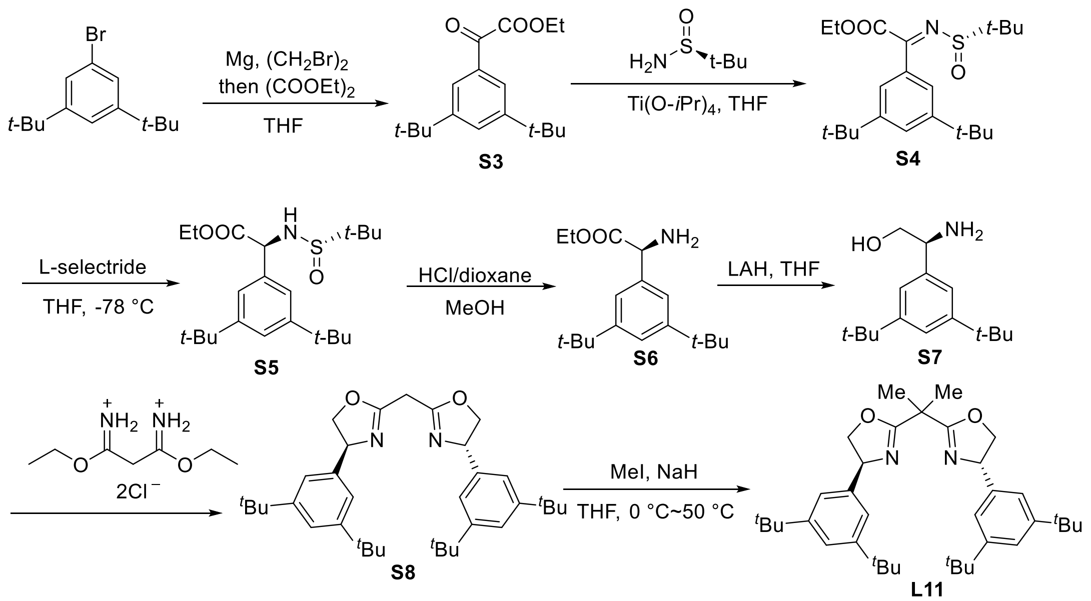

Under nitrogen atmosphere, to a 100 mL Schleck flask containing magnesium (13.0 mmol, 1.3 equiv) was added anhydrous THF (5.0 mL) followed by $(CH_2Br)_2$ (0.02 mL). To this mixture, a solution of 1-bromo-3,5-di-*tert*-butylbenzene (10.0 mmol, 1.0 equiv) in THF (5.0 mL) was added dropwise under stirring at room temperature. Then the mixture was stirring at 65 °C for 1 h. Diethyl oxalate (20.0 mmol) was dissolved in THF (5.0 mL) in a 100 mL round-bottom flask under nitrogen. The solution was cooled to -78 °C, then the above Grignard reagent was added to the reaction mixture in 10 min. After stirring for 1 h at -78 °C, the reaction was warmed up to room temperature and stirred for another 20 min. After the reaction completed, saturated $NaHCO_3$ solution (8.0 mL) was added. After stirring for 5 min, the mixture was filtered through a plug of Celite with ethyl acetate. The filtrate was extracted with ethyl acetate, washed with brine (8.0 mL × 2), dried over anhydrous Na_2SO_4 and concentrated, the residue was purified by chromatography on silica gel to give compound **S3**.

S3 (10.0 mmol) and (*S*)-*tert*-butylsulfinamide (10.0 mmol) were dissolved in anhydrous THF (15.0 mL), then Ti(O-*i*Pr)$_4$ (15.0 mmol, 1.5 equiv) was added. After stirring at 65 °C for 12 h under nitrogen, the solution was cooled to room temperature, diluted with ethyl acetate (15.0 mL), quenched by aqueous NaOH solution (10.0 % w/w, 10.0 mL) and the mixture was stirring for another 10 min before being filtered through a plug of Celite with ethyl acetate. The filtrate was then washed with brine (20.0 mL × 2), dried over anhydrous sodium sulfate, concentrated and purified by flash column chromatography to afford the **S4** in 30% yield over two steps.

S4 (10.0 mmol, 1.0 equiv) was dissolved in THF (30.0 mL) in a 100 mL round-bottom flask under nitrogen. The solution was cooled to -78 °C and stirred for 5 min, then L-Selectride in THF (1.0 M, 11.0 mmol, 1.1 equiv) was slowly added to the reaction mixture via a syringe at -78 °C Cover 40 min. The reaction was stirred at -

78 °C for another 5 h, quenched with saturated NH_4Cl solution (30.0 mL) at -78 °C, and warmed up to room temperature. The mixture was transferred to a separatory funnel containing brine (30.0 mL) and ethyl acetate (30.0 mL). The organic layer was separated and the aqueous layer was extracted with ethyl acetate (30.0 mL). The combined organic layers were washed with brine (30.0 mL × 2), dried over sodium sulfate, and concentrated. The crude product **S5** was used in the next step without further purification.

S5 was dissolved in methanol (20.0 mL) in a 100 mL round-bottom flask in open air. A solution of hydrogen chloride in dioxane (4.0 M, 40.0 mmol) was then added to the reaction mixture at 0 °C, over 2 min. The reaction flask was capped with a rubber septum and stirred at room temperature for 1 h. Then aqueous NaOH solution (10.0% w/w, 15.0 mL) was added slowly and the solution was extracted with ethyl acetate (50.0 mL), washed with brine (50.0 mL × 2), dried over sodium sulfate, and concentrated to afford the crude product **S6**.

S6 was dissolved in anhydrous THF (30.0 mL) in a 100 mL round-bottom flask under nitrogen and the solution was cooled to 0 °C. Lithium aluminum hydride (30.0 mmol) was then added portion-wise. The reaction was stirred at 0 °C overnight, and carefully quenched by sequential addition of 2.0 mL H_2O and 2.0 mL 15.0 wt% NaOH solution. The mixture was filtered through Celite with DCM, the filtrate was concentrated and purified by flash column chromatography on silica gel to afford the pure amino alcohol **S7** in 28% yield over three steps. **S8**, **L11** were synthesized following general procedure (A).

(4*S*,4'*S*)-2,2'-(Propane-2,2-diyl)bis(4-(3,5-di-*tert*-butylphenyl)-4,5-dihydrooxazole) (L11)

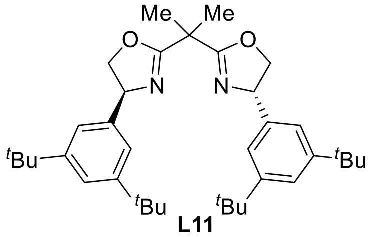

L11

L11 was obtained as white solid in 70% yield over two steps.
^{1}H NMR (400 MHz, $CDCl_3$) δ 7.32 (t, *J* = 1.8 Hz, 2H), 7.10 (d, *J* = 1.8 Hz, 4H), 5.17 (dd, *J* = 10.0, 6.7 Hz, 2H), 4.64 (dd, *J* = 10.1, 8.3 Hz, 2H), 4.25 (dd, *J* = 8.3, 6.7 Hz, 2H), 1.69 (s, 6H), 1.29 (s, 36H).
^{13}C NMR (101 MHz, $CDCl_3$) δ 170.2, 151.2, 141.9, 121.8, 121.2, 76.1, 70.3, 38.9, 35.0, 31.6, 24.8.

III. Preparation of Substrates and Oxidants

3.1 Synthesis of enol esters.

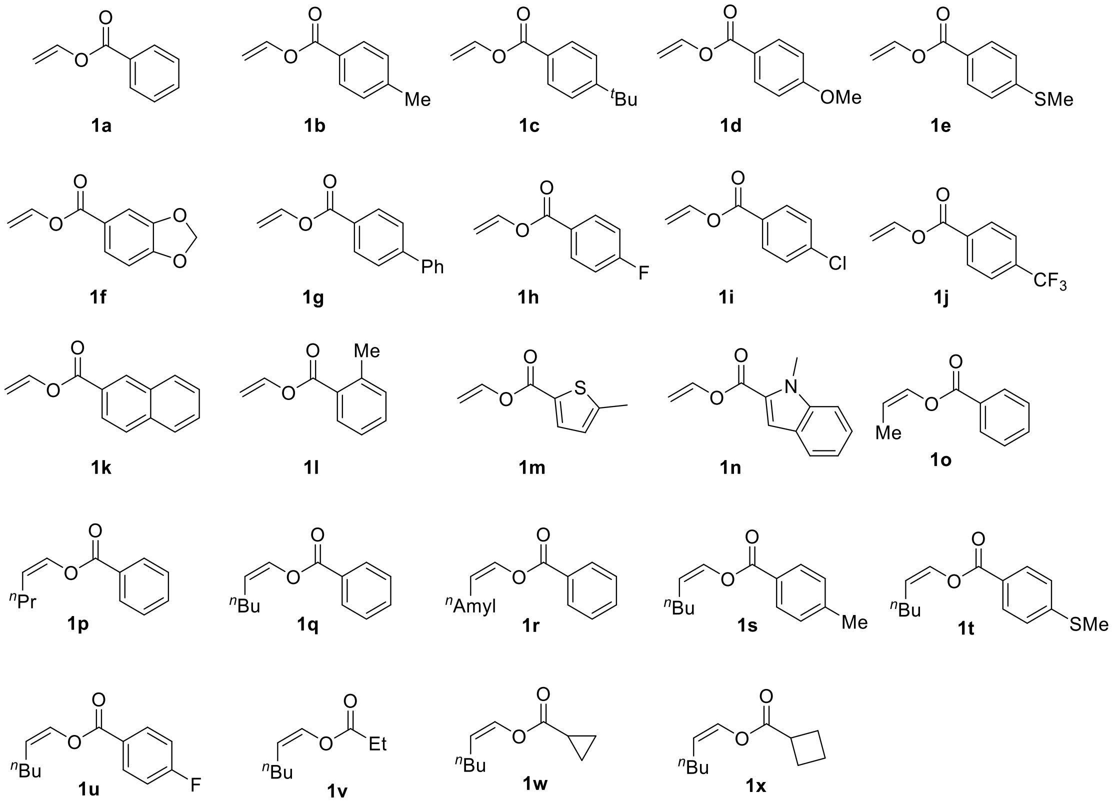

1a and **1c** were purchased from Energy Chemical.

General procedure for the synthesis of 1b, 1d-1n[6]

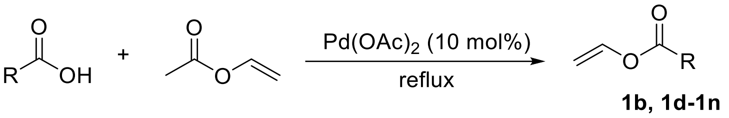

General procedure (B): To a pressure tube were added acid (1.0 equiv), $Pd(OAc)_2$ (0.1 equiv) and vinyl acetate (0.2 M). The reaction mixture was heated to reflux and stirred for 8 h at 80 ºC. After cooling down to room temperature, the mixture was filtered through a short pad of Celite and washed with CH_2Cl_2. The filtrate was concentrated under reduced pressure. The residue was purified by column chromatography on silica gel with gradient of petroleum ether/ethyl acetate to afford products. Characterization data is consistent with reported data.

Vinyl 4-methylbenzoate (1b)[7]

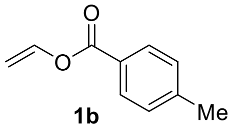

1b

1b was obtained as colorless oil in 80% yield.
^{1}H NMR (400 MHz, $CDCl_3$) δ 8.00 – 7.96 (m, 2H), 7.50 (dd, *J* = 14.0, 6.3 Hz, 1H), 7.26 – 7.24 (m, 2H), 5.04 (dd, *J* = 14.0, 1.6 Hz, 1H), 4.67 (dd, *J* = 6.3, 1.6 Hz, 1H), 2.41 (s, 3H).
^{13}C NMR (101 MHz, $CDCl_3$) δ 163.8, 144.6, 141.6, 130.2, 129.4, 126.3, 98.1, 21.9.

Vinyl 4-methoxybenzoate (1d)[7]

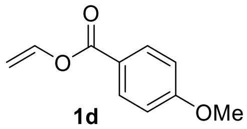

1d

1d was obtained as white solid in 82% yield.
^{1}H NMR (400 MHz, $CDCl_3$) δ 8.14 – 7.91 (m, 2H), 7.50 (dd, *J* = 14.0, 6.3 Hz, 1H), 7.01 – 6.86 (m, 2H), 5.03 (dd, *J* = 14.0, 1.6 Hz, 1H), 4.66 (dd, *J* = 6.3, 1.6 Hz, 1H), 3.86 (s, 3H).
^{13}C NMR (101 MHz, $CDCl_3$) δ 164.0, 163.5, 141.6, 132.2, 121.3, 113.9, 97.8, 55.6.

Vinyl 4-(methylthio)benzoate (1e)

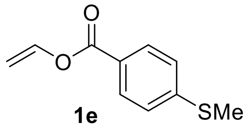

1e

1e was obtained as white solid in 79% yield.
^{1}H NMR (400 MHz, $CDCl_3$) δ 8.03 – 7.96 (m, 2H), 7.50 (dd, *J* = 14.0, 6.3 Hz, 1H), 7.29 – 7.24 (m, 2H), 5.05 (dd, *J* = 14.0, 1.7 Hz, 1H), 4.69 (dd, *J* = 6.3, 1.6 Hz, 1H), 2.52 (s, 3H).
^{13}C NMR (101 MHz, $CDCl_3$) δ 163.6, 146.7, 141.5, 130.4, 125.04, 124.97, 98.2, 14.9.
HRMS (ESI-TOF) Calcd for $C_{10}H_{11}O_2S$ $(M+H)^+$ 195.0474. Found 195.0470.

Vinyl benzo[*d*][1,3]dioxole-5-carboxylate (1f)[8]

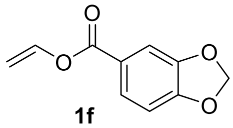

1f

1f was obtained as white solid in 81% yield.
^{1}H NMR (600 MHz, $CDCl_3$) δ 7.71 – 7.69 (m, 1H), 7.49 – 7.43 (m, 2H), 6.86 – 6.81 (m, 1H), 6.03 (s, 2H), 5.00 (d, *J* = 14.0 Hz, 1H), 4.64 (d, *J* = 6.3 Hz, 1H).
^{13}C NMR (151 MHz, $CDCl_3$) δ 163.1, 152.3, 148.0, 141.6, 126.2, 122.9, 109.8, 108.2, 102.1, 97.9.

Vinyl [1,1'-biphenyl]-4-carboxylate (1g)[8]

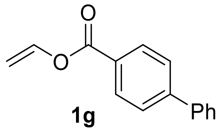

1g

1g was obtained as white solid in 82% yield.

^{1}H NMR (400 MHz, $CDCl_3$) δ 8.25 – 8.15 (m, 2H), 7.76 – 7.68 (m, 2H), 7.68 – 7.62 (m, 2H), 7.58 (dd, *J* = 14.0, 6.2 Hz, 2H), 7.51 – 7.39 (m, 2H), 5.10 (dd, *J* = 14.0, 1.7 Hz, 1H), 4.73 (dd, *J* = 6.2, 1.7 Hz, 1H).

^{13}C NMR (101 MHz, $CDCl_3$) δ 163.7, 146.5, 141.6, 140.0, 130.7, 129.1, 128.5, 127.7, 127.5, 127.3, 98.4.

Vinyl 4-fluorobenzoate (1h)[7]

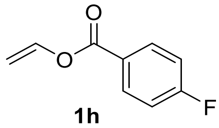

1h

1h was obtained as colorless oil in 76% yield.

^{1}H NMR (400 MHz, $CDCl_3$) δ 8.16 – 8.08 (m, 2H), 7.49 (dd, *J* = 14.0, 6.3 Hz, 1H), 7.19 – 7.06 (m, 2H), 5.06 (dd, *J* = 14.0, 1.7 Hz, 1H), 4.71 (dd, *J* = 6.3, 1.8 Hz, 1H).

^{13}C NMR (101 MHz, $CDCl_3$) δ 164.8 (d, $J_{C\text{-}F}$ = 256.0 Hz), 162.8, 141.4, 132.7 (d, $J_{C\text{-}F}$ = 9.5 Hz), 125.3 (d, $J_{C\text{-}F}$ = 3.0 Hz), 115.9 (d, $J_{C\text{-}F}$ = 22.0 Hz), 98.5.

Vinyl 4-chlorobenzoate (1i)[9]

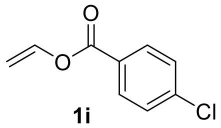

1i

1i was obtained as white solid in 79% yield.

^{1}H NMR (600 MHz, $CDCl_3$) δ 8.07 – 8.00 (m, 2H), 7.49 (dd, *J* = 14.0, 6.2 Hz, 1H), 7.46 – 7.42 (m, 2H), 5.07 (dd, *J* = 14.0, 1.8 Hz, 1H), 4.72 (dd, *J* = 6.3, 1.8 Hz, 1H).

^{13}C NMR (151 MHz, $CDCl_3$) δ 162.9, 141.4, 140.3, 131.5, 129.1, 127.5, 98.7.

Vinyl 4-(trifluoromethyl)benzoate (1j)

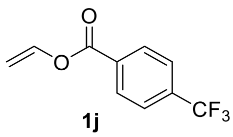

1j

1j was obtained as colorless oil in 68% yield.

^{1}H NMR (600 MHz, $CDCl_3$) δ 8.22 (d, *J* = 8.1 Hz, 2H), 7.74 (d, *J* = 8.1 Hz, 2H), 7.51 (dd, *J* = 13.9, 6.2 Hz, 1H), 5.12 (d, *J* = 13.0 Hz, 1H), 4.77 (d, *J* = 6.3 Hz, 1H).

^{13}C NMR (151 MHz, $CDCl_3$) δ 162.6, 141.4, 135.2 (q, $J_{C\text{-}F}$ = 32.7 Hz), 132.4, 130.5, 125.7 (q, $J_{C\text{-}F}$ = 3.0 Hz), 123.7 (q, $J_{C\text{-}F}$ = 273.3 Hz), 99.2.

HRMS (ESI-TOF) Calcd for $C_{10}H_8F_3O_2$ $(M+H)^+$ 217.0471. Found 217.0470.

Vinyl 2-naphthoate (1k)[8]

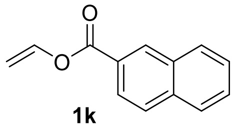

1k

1k was obtained as white solid in 72% yield.

^{1}H NMR (400 MHz, $CDCl_3$) δ 8.69 (s, 1H), 8.11 (dd, *J* = 8.6, 1.8 Hz, 1H), 7.97 (dd, *J* = 8.3, 1.6 Hz, 1H), 7.94 – 7.85 (m, 2H), 7.65 – 7.51 (m, 3H), 5.15 (dd, *J* = 14.0, 1.7 Hz, 1H), 4.76 (dd, *J* = 6.3, 1.7 Hz, 1H).

^{13}C NMR (101 MHz, $CDCl_3$) δ 163.9, 141.7, 135.9, 132.6, 131.9, 129.6, 128.8, 128.5, 127.9, 126.9, 126.2, 125.4, 98.4.

Vinyl 2-methylbenzoate (1l)[9]

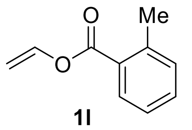

1l

1l was obtained as colorless oil in 70% yield.

^{1}H NMR (400 MHz, $CDCl_3$) δ 8.01 (dd, *J* = 8.2, 1.5 Hz, 1H), 7.53 – 7.48 (m, 1H), 7.46 – 7.41 (m, 1H), 7.30 – 7.24 (m, 2H), 5.04 (dd, *J* = 14.0, 1.6 Hz, 1H), 4.69 (dd, *J* = 6.3, 1.6 Hz, 1H), 2.64 (s, 3H).

^{13}C NMR (101 MHz, $CDCl_3$) δ 164.2, 141.3, 141.1, 132.5, 131.7, 130.9, 128.1, 125.8, 97.9, 21.7.

Vinyl 5-methylthiophene-2-carboxylate (1m)

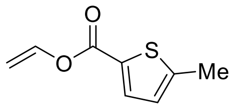

1m

1m was obtained as yellow oil in 65% yield.

^{1}H NMR (400 MHz, $CDCl_3$) δ 7.70 (dd, *J* = 3.8, 1.6 Hz, 1H), 7.42 (dd, *J* = 13.9, 6.3 Hz, 1H), 6.80 – 6.78 (m, 1H), 5.00 (d, *J* = 13.9 Hz, 1H), 4.65 (d, *J* = 6.3 Hz, 1H), 2.53 (s, 3H).

^{13}C NMR (101 MHz, $CDCl_3$) δ 159.3, 149.6, 141.2, 135.2, 129.6, 126.8, 97.9, 15.9.

HRMS (ESI-TOF) Calcd for $C_8H_9O_2S$ $(M+H)^+$ 169.0318. Found 169.0318.

Vinyl 1-methyl-1*H*-indole-2-carboxylate (1n)

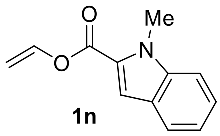

1n

1n was obtained as yellow solid in 61% yield.

^{1}H NMR (400 MHz, $CDCl_3$) δ 7.72 – 7.69 (m, 1H), 7.54 – 7.48 (m, 1H), 7.45 (s, 1H), 7.43 – 7.36 (m, 2H), 7.22 – 7.15 (m, 1H), 5.07 (dd, *J* = 14.0, 1.7 Hz, 1H), 4.70 (dd, *J* = 6.3, 1.7 Hz, 1H), 4.10 (s, 3H).
^{13}C NMR (101 MHz, $CDCl_3$) δ 159.2, 141.0, 140.3, 126.4, 126.0, 125.7, 123.0, 120.9, 111.8, 110.5, 98.0, 31.8.
HRMS (ESI-TOF) Calcd for $C_{12}H_{12}NO_2$ $(M+H)^+$ 202.0863. Found 202.0864.

Procedure for the synthesis of 1o-1s[10]

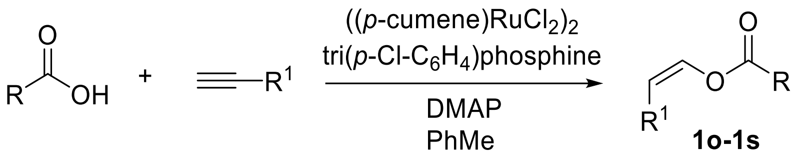

General procedure (C): A solution of ((*p*-cumene)$RuCl_2)_2$ (0.01 equiv), tri(*p*-Cl-C_6H_4)phosphine (0.03 equiv) and DMAP (0.04 equiv) in dry toluene was added to a solution of carboxylic acid (1.0 equiv) and alkyne (1.3 equiv) in dry toluene (0.1 M). The mixture was stirred for 16 h at 80 °C. After complete conversion (GC), usually 16 h, the mixture was cooled and filtered over a small plug of silica gel. The solvent was removed and the crude mixture was purified by column chromatography on silica gel with gradient of petroleum ether/ethyl acetate to afford products. Characterization data is consistent with reported data.

(*Z*)-Prop-1-en-1-yl benzoate (1o)[10]

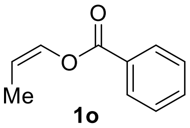

1o

1o was obtained as colorless oil in 70% yield.
^{1}H NMR (400 MHz, $CDCl_3$) δ 8.16 – 8.06 (m, 2H), 7.62 – 7.55 (m, 1H), 7.50 – 7.43 (m, 2H), 7.27 (dq, *J* = 6.4, 1.8 Hz, 1H), 5.09 -5.03 (m, 1H), 1.80 (dd, *J* = 6.9, 1.8 Hz, 3H).
^{13}C NMR (101 MHz, $CDCl_3$) δ 163.7, 135.1, 133.5, 130.0, 129.5, 128.6, 109.3, 10.1.

(*Z*)-Pent-1-en-1-yl benzoate (1p)[11]

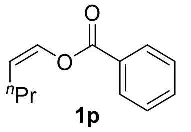

1p

1p was obtained as colorless oil in 85% yield.
^{1}H NMR (400 MHz, $CDCl_3$) δ 8.14 – 8.07 (m, 2H), 7.63 – 7.56 (m, 1H), 7.52 – 7.43 (m, 2H), 7.28 (dt, *J* = 6.4, 1.5 Hz, 1H), 5.02 (td, *J* = 7.5, 6.3 Hz, 1H), 2.30 – 2.24 (m, 2H), 1.53 – 1.44 (m, 2H), 0.97 (t, *J* = 7.4 Hz, 3H).
^{13}C NMR (101 MHz, $CDCl_3$) δ 163.7, 134.4, 133.5, 130.0, 129.6, 128.6, 114.8, 26.8, 22.5, 13.9.

(*Z*)-Hex-1-en-1-yl benzoate (1q)[12]

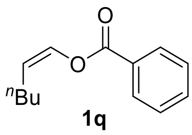

1q was obtained as colorless oil in 84% yield.

^{1}H NMR (400 MHz, $CDCl_3$) δ 8.11 (dd, *J* = 8.4, 1.4 Hz, 2H), 7.63 – 7.57 (m, 1H), 7.50 – 7.45 (m, 2H), 7.27 – 7.25 (m, 1H), 5.04 – 4.99 (m, 1H), 2.32 – 2.27 (m, 2H), 1.51 – 1.32 (m, 4H), 0.94 (t, *J* = 7.2 Hz, 3H).

^{13}C NMR (101 MHz, $CDCl_3$) δ 163.7, 134.3, 133.5, 130.0, 129.6, 128.7, 115.0, 31.5, 24.5, 22.4, 14.0.

(*Z*)-Hept-1-en-1-yl benzoate (1r)

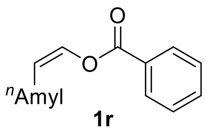

1r was obtained as colorless oil in 79% yield.

^{1}H NMR (400 MHz, $CDCl_3$) δ 8.13 – 8.07 (m, 2H), 7.64 – 7.55 (m, 1H), 7.48 (dd, *J* = 8.4, 7.0 Hz, 2H), 7.27 – 7.25 (m, 1H), 5.07 – 4.96 (m, 1H), 2.32 – 2.26 (m, 2H), 1.49 – 1.42 (m, 2H), 1.38 – 1.30 (m, 4H), 0.90 (t, *J* = 7.2 Hz, 3H).

^{13}C NMR (101 MHz, $CDCl_3$) δ 163.8, 134.3, 133.5, 130.0, 129.6, 128.7, 115.1, 31.5, 29.0, 24.8, 22.6, 14.2.

HRMS (ESI-TOF) Calcd for $C_{14}H_{19}O_2$ $(M+H)^+$ 219.1380. Found 219.1374.

(*Z*)-Hex-1-en-1-yl 4-methylbenzoate (1s)[13]

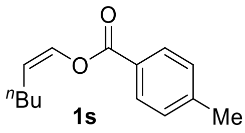

1s was obtained as colorless oil in 84% yield.

^{1}H NMR (400 MHz, $CDCl_3$) δ 8.08 – 7.91 (m, 2H), 7.31 – 7.24 (m, 3H), 5.05 – 4.96 (m, 1H), 2.41 (s, 3H), 2.35 – 2.26 (m, 2H), 1.48 – 1.36 (m, 4H), 0.94 (t, *J* = 7.3 Hz, 3H).

^{13}C NMR (101 MHz, $CDCl_3$) δ 162.4, 144.1 134.3, 130.1, 129.4, 126.8, 114.8, 31.5, 24.5, 22.4, 21.9, 14.1.

(*Z*)-Hex-1-en-1-yl 4-(methylthio)benzoate (1t)[14]

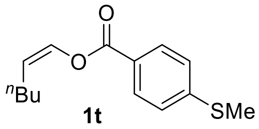

1t was obtained as colorless oil in 81% yield.

^{1}**H NMR** (400 MHz, $CDCl_3$) δ 8.01 – 7.95 (m, 2H), 7.29 – 7.22 (m, 3H), 5.02 – 4.95 (m, 1H), 2.52 (s, 3H), 2.31 – 2.25 (m, 2H), 1.46 – 1.33 (m, 4H), 0.93 (t, *J* = 7.1 Hz, 3H).
^{13}**C NMR** (101 MHz, $CDCl_3$) δ 163.5, 146.4, 134.3, 130.2, 125.6, 125.1, 114.8, 31.5, 24.5, 22.4, 14.9, 14.0.

(*Z*)-Hept-1-en-1-yl 4-fluorobenzoate (1u)

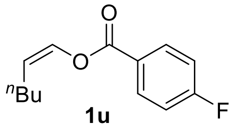

1u

1u was obtained as colorless oil in 72% yield.
^{1}**H NMR** (400 MHz, $CDCl_3$) δ 8.15 – 8.08 (m, 2H), 7.25 – 7.23 (m, 1H), 7.18 – 7.09 (m, 2H), 5.11 – 4.88 (m, 1H), 2.31 – 2.25 (m, 2H), 1.48 – 1.32 (m, 4H), 0.93 (t, *J* = 7.1 Hz, 3H).
^{13}**C NMR** (101 MHz, $CDCl_3$) δ 166.2 (d, $J_{C\text{-}F}$ = 255.8 Hz), 162.8, 134.2, 132.6 (d, $J_{C\text{-}F}$ = 9.5 Hz), 125.9 (d, $J_{C\text{-}F}$ =3.0 Hz), 115.9, 115.1, 31.4, 24.5, 22.4, 14.0.
HRMS (ESI-TOF) Calcd for $C_{13}H_{16}FO_2$ $(M+H)^+$ 223.1129. Found 223.1123.

(*Z*)-Hex-1-en-1-yl propionate (1v)

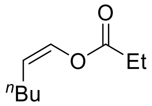

1v

1v was obtained as colorless oil in 75% yield.
^{1}**H NMR** (400 MHz, $CDCl_3$) δ 7.03 – 7.00 (m, 1H), 4.90 – 4.81 (m, 1H), 2.46 – 2.40 (m, 2H), 2.17 – 2.11 (m, 2H), 1.37 – 1.31 (m, 4H), 1.18 (t, *J* = 7.6 Hz, 3H), 0.90 (t, *J* = 7.2 Hz, 3H).
^{13}**C NMR** (101 MHz, $CDCl_3$) δ 171.8, 134.1, 114.3, 31.4, 27.6, 24.2, 22.3, 14.0, 9.0.
HRMS (ESI-TOF) Calcd for $C_9H_{17}O_2$ $(M+H)^+$ 157.1223. Found 157.1224.

(*Z*)-Hex-1-en-1-yl cyclopropanecarboxylate (1w)

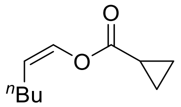

1w

1w was obtained as colorless oil in 79% yield.
^{1}**H NMR** (400 MHz, $CDCl_3$) δ 7.06 – 6.98 (m, 1H), 4.86 – 4.80 (m, 1H), 2.20 – 2.09 (m, 2H), 1.72 – 1.62 (m, 1H), 1.40 – 1.28 (m, 4H), 1.10 – 1.04 (m, 2H), 0.95 – 0.87 (m, 5H).
^{13}**C NMR** (101 MHz, $CDCl_3$) δ 172.2, 134.2, 113.9, 31.4, 24.2, 22.3, 14.0, 12.9, 9.0.
HRMS (ESI-TOF) Calcd for $C_{10}H_{17}O_2$ $(M+H)^+$ 169.1223. Found 169.1224.

(*Z*)-Hex-1-en-1-yl cyclobutanecarboxylate (1x)

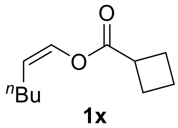

1x

1s was obtained as colorless oil in 70% yield.

^{1}H NMR (400 MHz, $CDCl_3$) δ 7.03 – 7.00 (m, 1H), 4.90 – 4.84 (m, 1H), 3.29 – 3.10 (m, 1H), 2.39 – 2.29 (m, 2H), 2.29 – 2.19 (m, 2H), 2.17 – 2.09 (m, 2H), 2.06 – 1.87 (m, 2H), 1.40 – 1.27 (m, 4H), 0.89 (t, *J* = 6.7 Hz, 3H).

^{13}C NMR (101 MHz, $CDCl_3$) δ 172.7, 134.3, 114.4, 38.1, 31.4, 25.3, 24.2, 22.3, 18.6, 14.0.

HRMS (ESI-TOF) Calcd for $C_{11}H_{19}O_2$ $(M+H)^+$ 183.1380. Found 183.1381.

3.2 Synthesis and characterization of unactivated alkenes

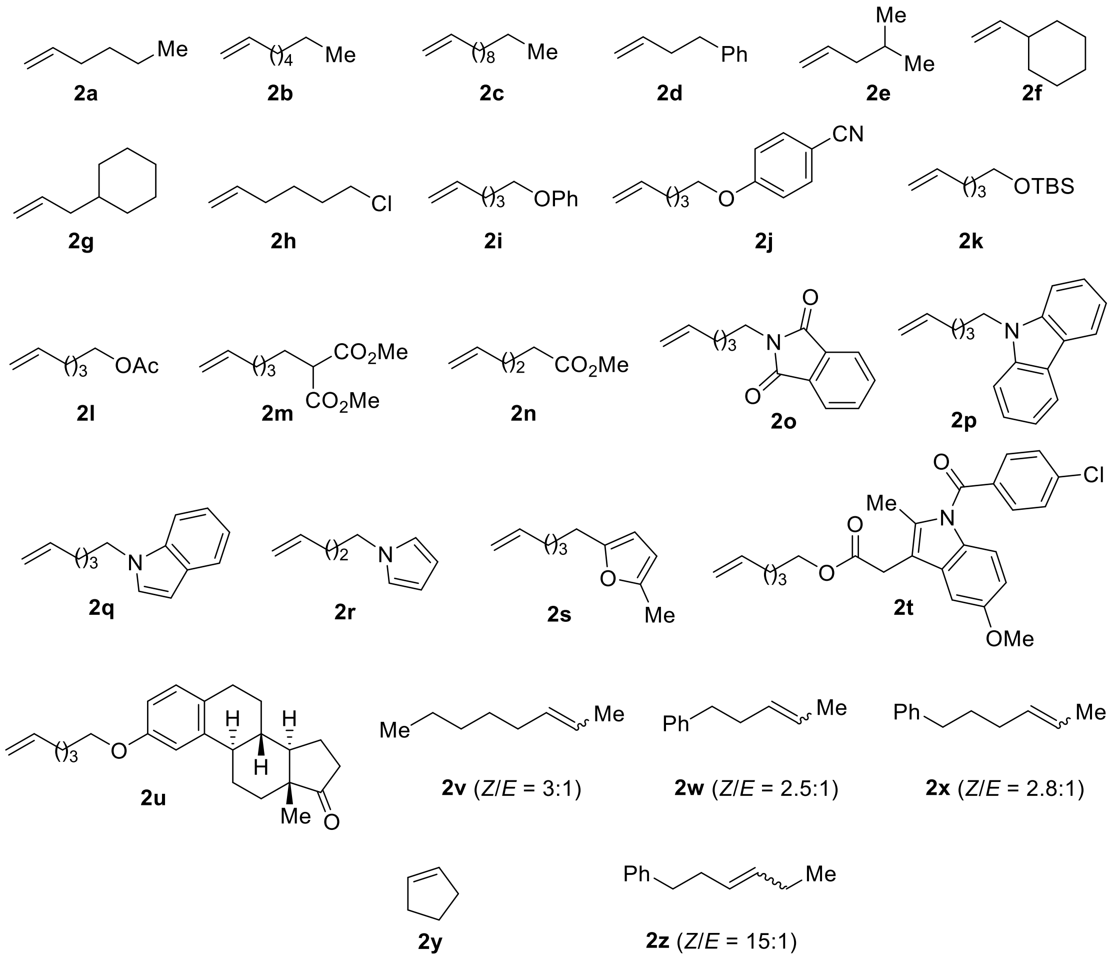

2a-2h, 2l, **2n** and **2v** were purchased from Aladdin, Macklin Reagent, J&K and Bidepharm.

General procedure for the synthesis of 2i, 2j, 2u[15]

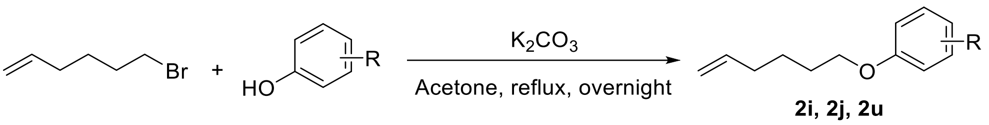

2i, 2j, 2u

6-Bromo-1-hexene (1.2 equiv) was added to a suspension of the phenol derivate (1.0 equiv) and potassium carbonate (2.5 equiv) in acetone (0.2 M). The reaction mixture was heated to reflux for overnight, cooled to room temperature, diluted with EtOAc, and quenched with water. The organic layer was separated, and the aqueous layer was extracted twice with EtOAc. The combined organic layers were dried over Na_2SO_4, filtered, and concentrated. The crude mixture was purified by column chromatography to afford desired product.

(Hex-5-en-1-yloxy)benzene (2i)[16]

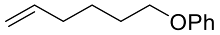

2i

2i was obtained as colorless oil in 85% yield.

^{1}H NMR (600 MHz, $CDCl_3$) δ 7.34 – 7.28 (m, 2H), 6.99 – 6.90 (m, 3H), 5.96 – 5.78 (m, 1H), 5.19 – 4.95 (m, 2H), 4.00 – 3.98 (m, 2H), 2.27 – 2.10 (m, 2H), 1.94 – 1.76 (m, 2H), 1.67 – 1.58 (m, 2H).

^{13}C NMR (151 MHz, $CDCl_3$) δ 159.2, 138.7, 129.5, 120.6, 114.8, 114.6, 67.7, 33.6, 28.9, 25.5.

4-(Hex-5-en-1-yloxy)benzonitrile (2j)[15]

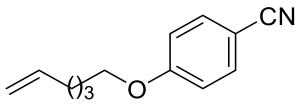

2j

2j was obtained as colorless oil in 79% yield.

^{1}H NMR (400 MHz, $CDCl_3$) δ 7.56 (d, J = 8.9 Hz, 2H), 6.92 (d, J = 8.9 Hz, 2H), 5.81 – 5.80 (m, 1H), 5.09 – 4.93 (m, 2H), 4.00 (t, J = 6.4 Hz, 2H), 2.17 – 2.08 (m, 2H), 1.87 – 1.77 (m, 2H), 1.61 – 1.52 (m, 2H).

^{13}C NMR (101 MHz, $CDCl_3$) δ 162.5, 138.4, 134.1, 119.4, 115.3, 115.1, 103.8, 68.3, 33.4, 28.5, 25.3.

(8*S*,9*R*,13*R*,14*R*)-2-(Hex-5-en-1-yloxy)-13-methyl-6,7,8,9,11,12,13,14,15,16-decahydro-17*H*-cyclopenta[*a*]phenanthren-17-one (2u)

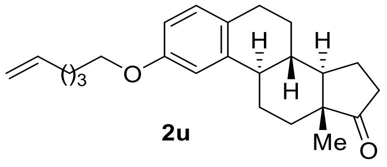

2u

2u was obtained as white solid in 80% yield.

^{1}H NMR (400 MHz, $CDCl_3$) δ 7.19 (d, J = 8.5 Hz, 1H), 6.76 – 6.55 (m, 2H), 5.92 – 5.74 (m, 1H), 5.12 – 4.89 (m, 2H), 3.94 (t, J = 6.4 Hz, 2H), 2.96 – 2.80 (m, 2H), 2.54 – 2.38 (m, 2H), 2.32 – 2.20 (m, 1H), 2.20 – 1.91 (m, 6H), 1.84 – 1.73 (m, 2H), 1.71 – 1.35 (m, 8H), 0.91 (s, 3H).

^{13}C NMR (101 MHz, $CDCl_3$) δ 221.1, 157.2, 138.6, 137.7, 131.9, 126.3, 114.8, 114.6, 112.1, 67.7, 50.4, 48.1, 44.0, 38.4, 35.9, 33.5, 31.6, 29.7, 28.8, 26.6, 26.0, 25.4, 21.6, 13.9.
HRMS (ESI-TOF) Calcd for $C_{24}H_{33}O_2$ $(M+H)^+$ 353.2475. Found 353.2466.

Procedure for the synthesis of 2k[17]

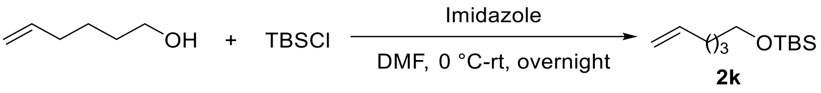

Imidazole (1.1 equiv) was added in one portion to a solution of 5-hexen-1-ol (1.0 equiv) in *N*,*N*-dimethylformamide (0.5 M). The resulting solution was stirred for 10 min at 0 °C before *t*-butyldimethylsilyl chloride (1.1 equiv) was added. After stirring an additional 10 min at 0 °C, the reaction solution was warmed to room temperature and stirred for overnight. Excess *tert*-butyldimethylsilyl chloride was then quenched by the addition of water, and the resulting aqueous solution was extracted with ethyl ether. The combined organic extracts were washed with water and brine, and then dried over Na_2SO_4. The resulting residue was purified by silica gel column chromatography to give desired compound **2k** as colorless oil in 76% yield.

***tert*-Butyl(hex-5-en-1-yloxy)dimethylsilane (2k)**

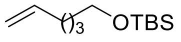

2k

^{1}H NMR (400 MHz, $CDCl_3$) δ 5.83– 5.78 (m, 1H), 5.06 – 4.88 (m, 2H), 3.61 (t, J = 6.4 Hz, 2H), 2.17 – 1.97 (m, 2H), 1.58 – 1.49 (m, 2H), 1.48 – 1.37 (m, 2H), 0.89 (s, 9H), 0.05 (s, 6H).
^{13}C NMR (101 MHz, $CDCl_3$) δ 139.1, 114.5, 63.2, 33.7, 32.5, 26.1, 25.3, 18.5, -5.1.

Procedure for the synthesis of 2m[18]

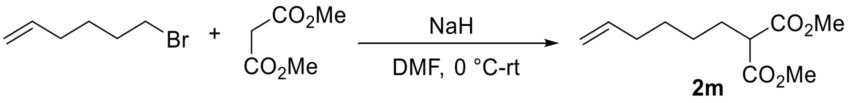

To a suspension of NaH (1.5 equiv) in DMF was slowly added malonic ester (1.25 equiv) at 0 °C, and the mixture was gradually warmed to room temperature. Then 6-bromohex-1-ene (1.0 equiv) was added dropwise to the flask and the mixture was stirred for 18 h. A saturated aqueous solution of ammonium chloride was introduced to the mixture, which was then extracted with EtOAc three times. Combined organic portions were washed with brine and dried over Na_2SO_4, filtered, and concentrated. Column chromatography of the crude material on silica gel to afford the desired compound **2m** as colorless oil in 68% yield.

Dimethyl 2-(hex-5-en-1-yl)malonate (2m)

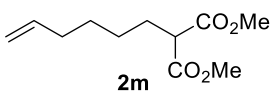

2m

¹H NMR (400 MHz, $CDCl_3$) δ 5.85 – 5.66 (m, 1H), 5.06 – 4.85 (m, 2H), 3.72 (s, 6H), 3.34 (t, *J* = 7.6 Hz, 1H), 2.08 – 1.98 (m, 2H), 1.89 (q, *J* = 7.7 Hz, 2H), 1.44 – 1.27 (m, 4H).
¹³C NMR (101 MHz, $CDCl_3$) δ 170.0, 138.6, 114.7, 52.6, 51.7, 33.5, 28.8, 28.5, 26.8.
HRMS (ESI-TOF) Calcd for $C_{11}H_{19}O_4$ $(M+H)^+$ 215.1278. Found 215.1274.

Procedure for the synthesis of 2o[19]

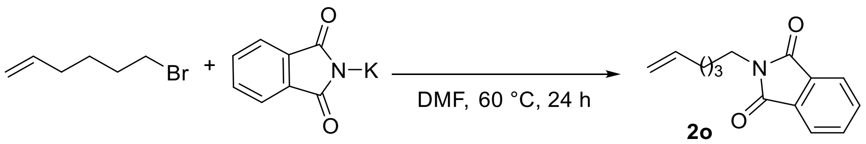

To an oven-dried round-bottom flask containing a magnetic stir bar were added 6-bromohex-1-ene (1.0 equiv), potassium phthalimide (1.1 equiv) and DMF (0.5 M). The solution was heated to 60 °C for 24 h. After filtration, the mixture was washed with H_2O and extracted with DCM. The organic layers were washed with brine, dried over Na_2SO_4, filtered, and concentrated in vacuo. The resulting residue was purified by silica gel column chromatography to give **2o** as colorless oil in 80% yield.

2-(Hex-5-en-1-yl)isoindoline-1,3-dione (2o)

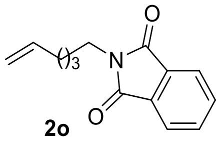

¹H NMR (400 MHz, $CDCl_3$) δ 7.83 (dd, *J* = 5.4, 3.0 Hz, 2H), 7.70 (dd, *J* = 5.4, 3.0 Hz, 2H), 5.80 – 5.76 (m, 1H), 5.04 – 4.89 (m, 2H), 3.68 (t, *J* = 7.3 Hz, 2H), 2.15 – 2.00 (m, 2H), 1.74 – 1.63 (m, 2H), 1.51 – 1.37 (m, 2H).
¹³C NMR (101 MHz, $CDCl_3$) δ 168.6, 138.4, 134.1, 132.3, 123.3, 115.1, 38.0, 33.4, 28.1, 26.2.

General procedure for the synthesis of 2p, 2q and 2r[20]

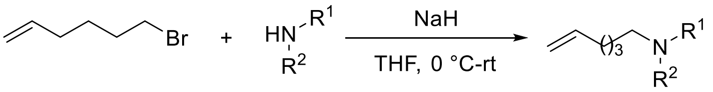

To an oven-dried 100 mL double-neck round bottom flask was added NaH (2.0-3.0 equiv) and dry THF or DMF (0.25 M) under N_2 atmosphere. The mixture was further cooled to 0 °C and then the corresponding amine (1.0 equiv) was added to this solution at 0 °C. The mixture was stirred for 30 min at 0 °C, and subsequently, respective 6-bromohex-1-ene (1.5 equiv) was added. After the addition was complete, the solution was kept stirring by gradually increasing to room temperature and was stirred for 12 h. After the reaction completion, the mixture was extracted with EtOAc and dried before purified on a silica column to afford desire compound.

9-(Hex-5-en-1-yl)-9*H*-carbazole (2p)[21]

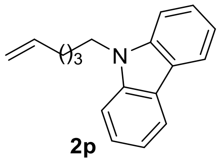

2p

2p was obtained as colorless oil in 79% yield.

^{1}H NMR (600 MHz, $CDCl_3$) δ 8.13 (d, *J* = 7.7 Hz, 2H), 7.49 (t, *J* = 7.7 Hz, 2H), 7.45 – 7.39 (m, 2H), 7.25 (t, *J* = 7.5 Hz, 2H), 5.80 – 5.77 (m, 1H), 5.03 – 4.94 (m, 2H), 4.34 (t, *J* = 7.3 Hz, 2H), 2.15 – 2.08 (m, 2H), 1.96 – 1.87 (m, 2H), 1.54 – 1.45 (m, 2H).

^{13}C NMR (151 MHz, $CDCl_3$) δ 140.5, 138.3, 125.7, 122.9, 120.5, 118.9, 115.1, 108.7, 43.0, 33.6, 28.5, 26.6.

1-(Hex-5-en-1-yl)-1*H*-indole (2q)[20]

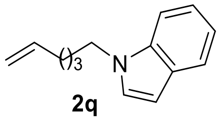

2q

2q was obtained as yellow oil in 72% yield.

^{1}H NMR (400 MHz, $CDCl_3$) δ 7.68 – 7.59 (m, 1H), 7.38 – 7.33 (m, 1H), 7.25 – 7.18 (m, 1H), 7.15 – 7.05 (m, 2H), 6.52 – 6.48 (m, 1H), 5.80 – 5.76 (m, 1H), 5.05 – 4.92 (m, 2H), 4.13 (t, *J* = 7.1 Hz, 2H), 2.12 – 2.06 (m, 2H), 1.91 – 1.83 (m, 2H), 1.47 – 1.41 (m, 2H).

^{13}C NMR (101 MHz, $CDCl_3$) δ 138.3, 136.1, 128.7, 127.9, 121.4, 121.1, 119.3, 115.1, 109.5, 101.0, 46.3, 33.4, 29.8, 26.3.

1-(Pent-4-en-1-yl)-1*H*-pyrrole (2r)[20]

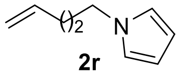

2r

2r was obtained as yellow oil in 64% yield.

^{1}H NMR (400 MHz, $CDCl_3$) δ 6.66 (t, *J* = 2.1 Hz, 2H), 6.15 (t, *J* = 2.0 Hz, 2H), 5.91 – 5.70 (m, 1H), 5.13 – 4.94 (m, 2H), 3.89 (t, *J* = 7.0 Hz, 2H), 2.15 – 1.99 (m, 2H), 1.99 – 1.79 (m, 2H).

^{13}C NMR (101 MHz, $CDCl_3$) δ 137.5, 120.6, 115.6, 108.0, 48.9, 30.8, 30.7.

Procedure for the synthesis of 2s[22]

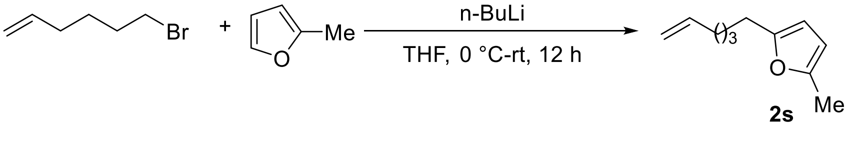

To a stirred solution of furan (1.3 equiv) in dry THF was slowly added n-BuLi at at 0 °C under a nitrogen atmosphere, After the resulting mixture was stired for 1 h at the same temperature, a solution of 6-bromohex-1-ene (1.0 equiv) in dry THF (0.1 M) was added slowly at -20 °C. The reaction mixture was warmed to room temperature and stirred for an additional 12 h. Then quenched by the addition of water, and the resulting aqueous solution was extracted with ethyl ether. The combined organic extracts were

washed with water and brine, and then dried over Na_2SO_4. The resulting residue was purified by silica gel column chromatography to give desired compound **2s** as yellow oil in 74% yield.

2-(Hex-5-en-1-yl)-5-methylfuran (2s)

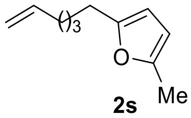

^{1}H NMR (400 MHz, $CDCl_3$) δ 5.97 – 5.67 (m, 3H), 5.09 – 4.98 (m, 1H), 4.98 – 4.90 (m, 1H), 2.57 (t, *J* = 7.6 Hz, 2H), 2.26 (s, 3H), 2.08 (q, *J* = 7.1 Hz, 2H), 1.68-1.60 (m, 2H), 1.50 – 1.41 (m, 2H).

^{13}C NMR (101 MHz. $CDCl_3$) δ 154.7, 150.2, 139.0, 114.6, 105.9, 105.3, 33.7, 28.6, 28.0, 27.8, 13.6.

HRMS (ESI): Calcd for $C_{11}H_{17}O$ $[M+H]^+$: 165.1274. found: 165.1273.

General procedure for the synthesis of 2t[23]

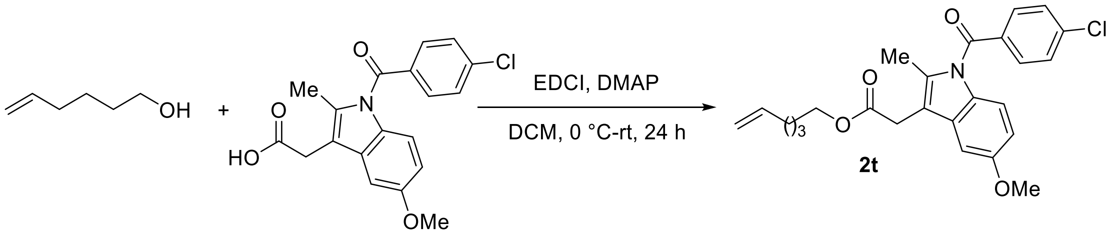

To a solution of acid (1.2 equiv) in CH_2Cl_2 at 0 °C were added successively 4-dimethylaminopyridine (DMAP, 0.2 equiv), hex-5-en-1-ol (1.0 equiv), and *N*-(3-dimethylaminopropyl)-*N'*-ethylcarbodiimide hydrochloride (EDCI, 2.0 equiv). The reaction mixture was stirred at room temperature for 24 h, filtered through celite and concentrated. The resulting residue was purified by silica gel column chromatography to give desired compound **2t** as yellow oil in 80% yield.

Hex-5-en-1-yl 2-(1-(4-chlorobenzoyl)-5-methoxy-2-methyl-1*H*-indol-3-yl)acetate (2t)

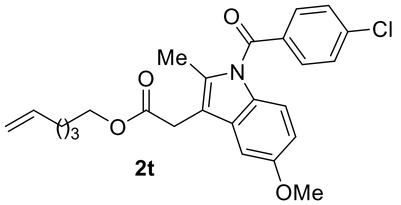

^{1}H NMR (400 MHz, $CDCl_3$) δ 7.69 – 7.62 (m, 2H), 7.50 – 7.44 (m, 2H), 6.96 (d, *J* = 2.5 Hz, 1H), 6.87 (d, *J* = 9.0 Hz, 1H), 6.67 (dd, *J* = 9.0, 2.5 Hz, 1H), 5.76 – 5.73 (m, 1H), 5.04 – 4.90 (m, 2H), 4.10 (t, *J* = 6.6 Hz, 2H), 3.83 (s, 3H), 3.66 (s, 2H), 2.39 (s, 3H), 2.09 – 1.97 (m, 2H), 1.71 – 1.57 (m, 2H), 1.47 – 1.34 (m, 2H).

^{13}C NMR (101 MHz, $CDCl_3$) δ 171.1, 168.4, 156.2, 139.4, 138.3, 136.0, 134.0, 131.3, 130.9, 130.8, 129.2, 115.1, 115.0, 112.8, 111.8, 101.4, 65.1, 55.8, 33.3, 30.5, 28.1, 25.3, 13.5.

HRMS (ESI-TOF) Calcd for $C_{25}H_{27}ClNO_4$ $(M+H)^+$ 440.1623. Found 440.1615.

Procedure for the synthesis of 2w and 2x[24]

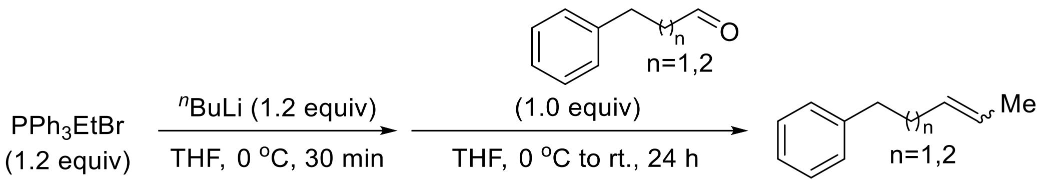

2w, **2x**

PPh3EtBr (12.0 mmol) was added to an oven-dried transparent Schlenk tube (100 mL) equipped with a stirring bar. The tube was evacuated and filled with argon (three times). Anhydrous tetrahydrofuran (40.0 mL) was added to the tube under argon, and then *n*-BuLi (2.5 M, 12.0 mmol) was added dropwise to the solution at 0 °C. The mixture was stirred at 0 °C for 30 min, then a THF solution of aldehyde (10.0 mmol) was added at 0 °C. The reaction mixture was stirred at room temperature for 24 h. The resulting solution was quenched with aqueous solution of NH_4Cl and the mixture was extracted with ethyl acetate (3 x 40.0 mL). The combined organic phase was dried over Na_2SO_4, filtrated and concentrated under vacuo. The residue was purified by column chromatography on silica gel to afford desired compound.

Pent-3-en-1-ylbenzene (2w)[25]

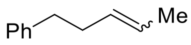

2w (*Z*/*E* = 2.5:1)

2w was obtained as colorless oil in 75% yield.
^{1}H NMR (400 MHz, $CDCl_3$) δ 7.28 – 7.19 (m, 2H), 7.18 – 7.09 (m, 3H), 5.57 – 5.25 (m, 2H), 2.76 – 2.46 (m, 2H), 2.38 – 2.19 (m, 2H), 1.60 (d, *J* = 3.5 Hz, 0.86H), 1.52 (d, *J* = 5.5 Hz, 2.14H).
^{13}C NMR (101 MHz, $CDCl_3$) δ 142.3, 130.7, 129.8, 128.6, 128.4, 125.9, 125.8, 125.5, 124.6, 36.3, 35.9, 34.6, 28.9, 18.1, 12.9.

Hex-4-en-1-ylbenzene (2x)[26]

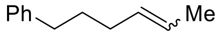

2x (*Z*/*E* = 2.8:1)

2x was obtained as colorless oil in 72% yield.
^{1}H NMR (400 MHz, $CDCl_3$) δ 7.35 – 7.27 (m, 2H), 7.24 – 7.17 (m, 3H), 5.68 – 5.34 (m, 2H), 2.64 (q, *J* = 8.0 Hz, 2H), 2.15 – 2.00 (m, 2H), 1.80 – 1.68 (m, 2H), 1.77 – 1.68 (m, 0.80H), 1.62 (d, *J* = 5.7 Hz, 2.20H).
^{13}C NMR (101 MHz, $CDCl_3$) δ 142.6, 131.0, 130.2, 128.4, 128.2, 125.6, 125.2, 35.5, 35.4, 32.1, 31.3, 26.4, 17.9, 12.8.

3.3 Synthesis and characterization of oxidants

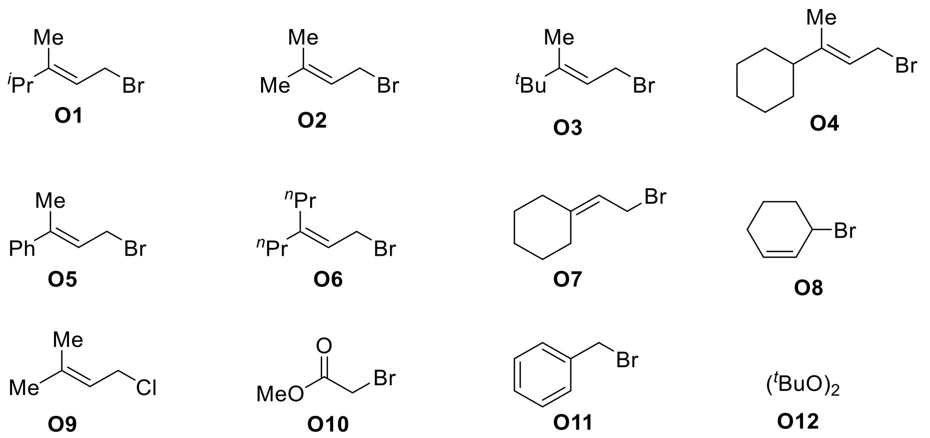

O2, **O8**, **O9** and **O10-O12** were purchased from Meryer, Bidepharm, Adamas-beta® and Aladdin.

O1, O3-O7 was synthesized following reported method.[27]

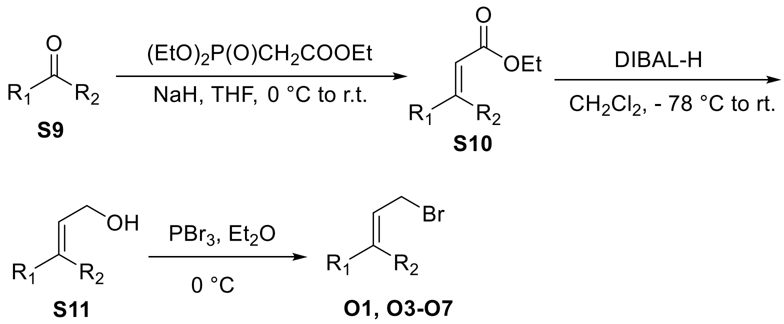

General procedure (D): To a cooled (0 °C) suspension of NaH (60 wt% in mineral oil, 2.0 equiv) in THF (0.15 M) is added triethyl phosphonoacetate (1.1 equiv). The resulting mixture is stirred at 0 °C for 30 min and the corresponding ketone (1.0 equiv) is added (neat for liquids, in solution in THF for solids). The mixture is stirred at the indicated temperature until full conversion of the ketone is detected by NMR analysis. The reaction is quenched by addition of H_2O and the aqueous phase is extracted with ethyl acetate. The combined organic layers are dried over Na_2SO_4 and filtered. Removal of all volatiles under reduced pressure and purification by flash column chromatography (cyclohexane/*tert*-butyl methyl ether) yields the pure products **S10** in 80% yield.

To a solution of the corresponding α,β-unsaturated ester **S10** (1.0 equiv) in CH_2Cl_2 (0.2 M) is added a solution of DIBAL-H (1.2 M in toluene, 3.0 equiv) at -78 °C. After complete addition the cooling bath is removed and the resulting solution is stirred at rt. until full conversion of the starting material S1 is detected by TLC analysis. The reaction was quenched with water at 0 °C. To the resulting mixture, saturated aqueous solution of sodium hydroxide and water were added. After filtration of the mixture over celite, removal of all volatiles under reduced pressure yields the crude products **S11**, which are purified by flash column chromatography in 67% yield.

The corresponding allylic alcohol **S11** (1.0 equiv) is dissolved in Et_2O (0.1 M) and the resulting mixture cooled to 0 °C. PBr_3 (1.05 equiv) is added and stirred at 0 °C for approximately 30 min. After completion of the reaction the reaction is poured into ice-

cooled sat. aq. NH_4Cl-solution. The aqueous layer is extracted with Et_2O. The combined organic layers are washed with water dried over Na_2SO_4 and filtered. All volatiles are removed under reduced pressure. Allylic bromides **O1**, **O3-O7** were used, without further purification.

(*E*)-1-Bromo-3,4-dimethylpent-2-ene (O1)[28]

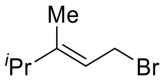

O1

O1 was obtained as yellow oil in 92% yield.
^{1}H NMR (400 MHz, $CDCl_3$) δ 5.57 – 5.52 (m, 1H), 4.03 (d, *J* = 8.5 Hz, 2H), 2.38 – 2.19 (m, 1H), 1.70 (s, 3H), 1.01 (d, *J* = 6.9 Hz, 6H).
^{13}C NMR (101 MHz, $CDCl_3$) δ 149.4, 118.5, 36.8, 30.0, 21.1, 13.5.

(*E*)-1-Bromo-3,4,4-trimethylpent-2-ene (O3)[28]

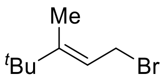

O3

O3 was obtained as yellow oil in 90% yield.
^{1}H NMR (400 MHz, $CDCl_3$) δ 5.55 – 5.51 (m, 1H), 3.97 (d, *J* = 8.4 Hz, 2H), 1.66 (d, *J* = 1.4 Hz, 3H), 0.98 (s, 9H).
^{13}C NMR (101 MHz, $CDCl_3$) δ 151.7, 117.6, 36.7, 30.5, 28.8, 12.6.

(*E*)-(4-Bromobut-2-en-2-yl)cyclohexane (O4)[29]

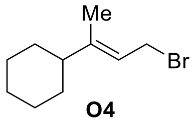

O4

O4 was obtained as yellow oil in 89% yield.
^{1}H NMR (400 MHz, $CDCl_3$) δ 5.52 (tq, *J* = 8.5, 1.0 Hz, 1H), 4.01 (d, *J* = 8.4 Hz, 2H), 1.89 – 1.67 (m, 5H), 1.60 (d, *J* = 1.0 Hz, 3H), 1.56 – 1.45 (m, 3H), 1.40 – 1.27 (m, 3H).
^{13}C NMR (101 MHz, $CDCl_3$) δ 149.0, 118.8, 47.4, 31.6, 30.2, 26.7, 26.4, 14.5.

(*E*)-(4-Bromobut-2-en-2-yl)benzene (O5)[27]

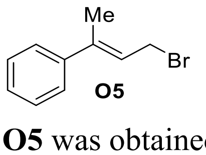

O5

O5 was obtained as yellow oil in 86% yield.
^{1}H NMR (400 MHz, $CDCl_3$) δ 7.22 – 7.02 (m, 5H), 5.84 (tq, *J* = 8.6, 1.4 Hz, 1H), 3.95 (d, *J* = 8.6 Hz, 2H), 1.89 (d, *J* = 1.5 Hz, 3H).
^{13}C NMR (151 MHz, $CDCl_3$) δ 142.3, 141.6, 128.5, 127.9, 126.1, 122.9, 29.5, 15.7.

4-(2-Bromoethylidene)heptane (O6)[27]

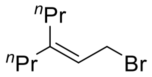

O6

O6 was obtained as yellow oil in 90% yield.
^{1}H NMR (400 MHz, $CDCl_3$) δ 5.52 (t, *J* = 8.5 Hz, 1H), 4.04 (d, *J* = 8.5 Hz, 2H), 2.13 – 2.05 (m, 2H), 2.05 – 1.98 (m, 2H), 1.49 – 1.40 (m, 4H), 0.94 – 0.86 (m, 6H).
^{13}C NMR (101 MHz, $CDCl_3$) δ 147.6, 120.9, 39.0, 32.2, 29.7, 21.5, 21.0, 14.3, 13.9.

(2-Bromoethylidene)cyclohexane (O7)[30]

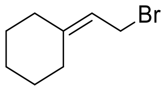

O7

O7 was obtained as yellow oil in 93% yield.
^{1}H NMR (400 MHz, $CDCl_3$) δ 5.43 (t, *J* = 8.5 Hz, 1H), 3.98 (d, *J* = 8.5 Hz, 2H), 2.21 – 2.15 (m, 2H), 2.11 – 2.04 (m, 2H), 1.63 – 1.43 (m, 6H).
^{13}C NMR (101 MHz, $CDCl_3$) δ 148.1, 117.6, 37.1, 29.1, 28.7, 28.4, 27.6, 26.7.

IV. Optimization of Reaction Conditions

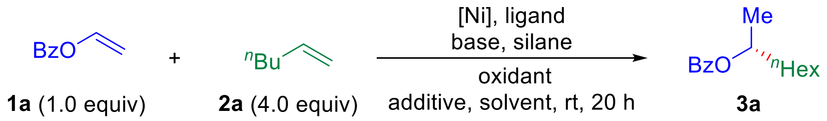

General procedure (E): To an oven-dried 10 mL Teflon-screw cap tube containing a magnetic stir was charged with Ni-catalyst (10 mol%) and ligand (12 mol%) in a nitrogen-filled glove-box. Subsequently, anhydrous solvent was added. The mixture was stirred for 5-10 min at room temperature. Then, base, olefin **1a** (0.10 mmol, 1.0 equiv) and **2a** (0.40 mmol, 4.0 equiv) were added. oxidant and additive were added subsequently. Finally, silane was added dropwise to the above mixture. The tube was sealed and removed from the glove box, and the reaction was stirred at 25 °C for 20 h at 1400 rpm. After the reaction was completed, the reaction mixture was diluted with saturated NH_4Cl (aqueous solution, 0.5 mL) and EtOAc (3.0 mL). Dodecane (23.0 μL, 0.1 mmol) was added as an internal standard and a small aliquot of the organic phase was removed for GC analysis to confirm yield. For the remaining mixture, the aqueous phase was extracted with EtOAc (2 × 3.0 mL). The combined organic phase was dried over Na_2SO_4, and the volatiles were removed to afford the crude product. Then, the mixture was purified by PTLC and the enantiomeric excess was determined by HPLC analysis.

Table S1. Evaluation of solvent.[a]

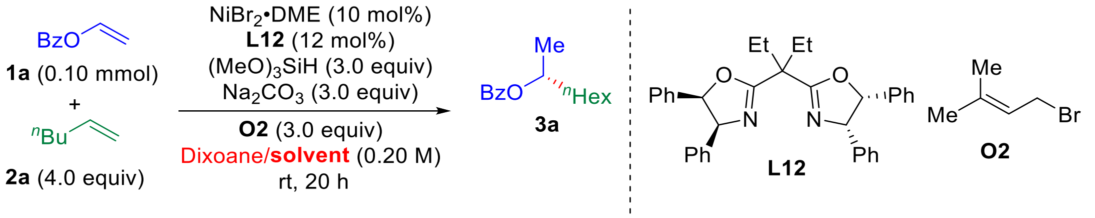

entry	solvent	1a rsm (%)	yield (%)	ee (%)
1	Dioxane\Acetone (3:2)	16	41	82
2	Dioxane\DCE (3:2)	0	45	82
3	Dioxane\$CHCl_3$ (3:2)	15	49	81
4	Dioxane\DCM (3:2)	18	48	85
5	Dioxane\$PhCF_3$ (3:2)	16	40	69
6	Dioxane\2-Butanone (3:2)	28	32	71
7	Dioxane\2-MeTHF (3:2)	31	29	73
8	Dioxane\DME (3:2)	26	30	69
9	Dioxane\DMA (3:2)	17	22	59
10	Dioxane\CH_3CN (3:2)	11	58	53

[a] The yield was determined by GC using dodecane as internal standard. The ee value was determined by HPLC on a chiral stationary phase.

Table S2. Evaluation of loading of oxidant.[a]

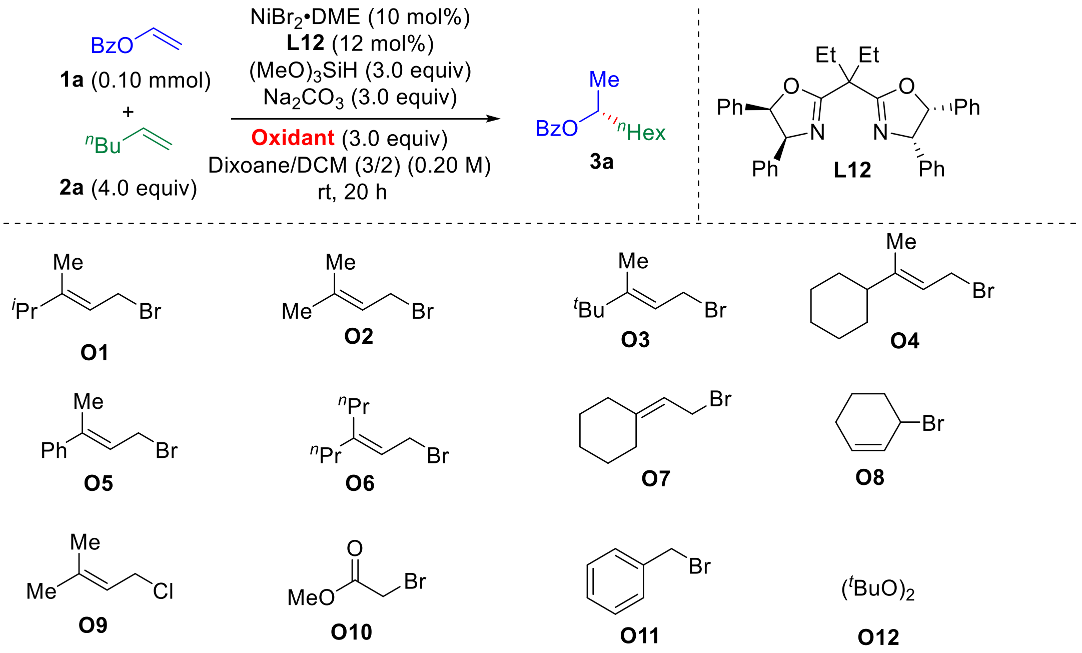

entry	Oxidant	1a rsm (%)	yield (%)	ee (%)
1	**O1**	27	52	85
2	**O2**	18	48	85
3	**O3**	35	40	85
4	**O4**	16	47	85
5	**O5**	21	63	78
6	**O6**	52	23	82
7	**O7**	62	18	82
8	**O8**	38	17	65
9	**O9**	48	16	79
10	**O10**	0	38	64
11	**O11**	81	-	-
12	**O12**	84	-	-
13[b]	**O1**	15	62	85

[a] The yield was determined by GC using dodecane as internal standard. The ee value was determined by HPLC on a chiral stationary phase. [b] 4.0 equiv oxidant was used.

Table S3. Evaluation of additive.[a]

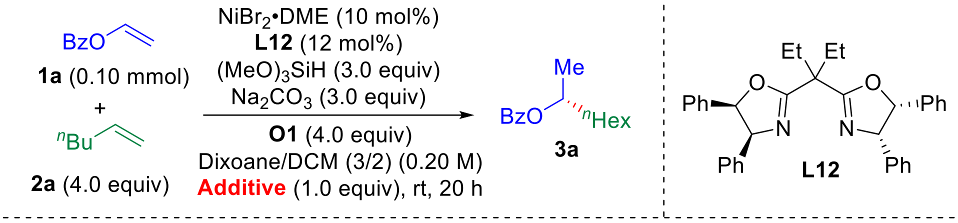

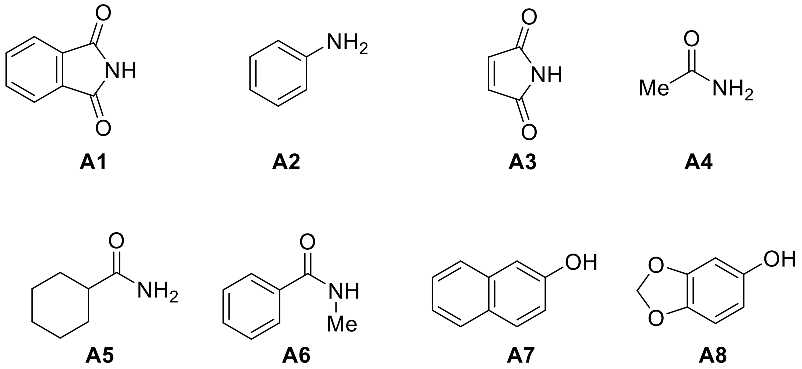

entry	additive	1a rsm (%)	yield (%)	ee (%)
1	**A1**	0	84	86
2	**A2**	45	25	82
3	**A3**	27	46	85
4	**A4**	51	30	79
5	**A5**	47	33	79
6	**A6**	43	26	80
7	**A7**	36	46	80
8	**A8**	42	40	80

[a] The yield was determined by GC using dodecane as internal standard. The ee value was determined by HPLC on a chiral stationary phase.

Table S4. Evaluation of base.[a]

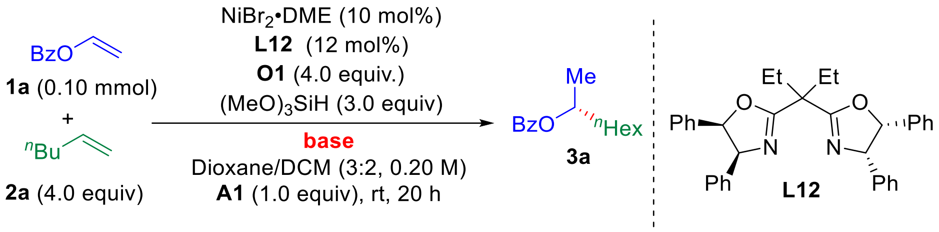

entry	Base	1a rsm (%)	yield (%)	ee (%)
1	Li_2CO_3 (3.0 equiv)	89	-	-
2	K_2CO_3 (3.0 equiv)	23	61	82
3	$K_3PO_4 \cdot H_2O$ (3.0 equiv)	8	77	85
4	Na_3PO_4 (3.0 equiv)	32	49	79
5	K_3PO_4 (3.0 equiv)	31	54	80
6	NaF (3.0 equiv)	51	17	91
7	KF (3.0 equiv)	47	30	80
8	CsF (3.0 equiv)	15	60	91
9	CsF\\Na_2CO_3 (2.0 : 0.5 equiv)	20	62	92
10	CsF\\Na_2CO_3 (2.0 : 1.0 equiv)	11	71	88
11	CsF\\Na_2CO_3 (2.0 : 1.5 equiv)	8	74	88
12	CsF\\Na_2CO_3 (2.0 : 2.0 equiv)	5	76	88

[a] The yield was determined by GC using dodecane as internal standard. The ee value was determined by HPLC on a chiral stationary phase.

Table S5. Evaluation of ligand.[a]

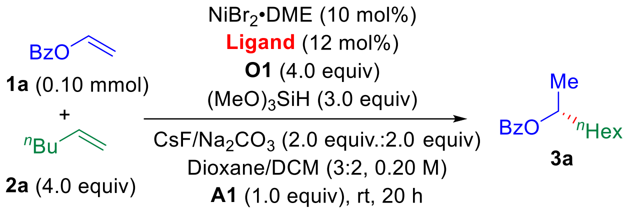

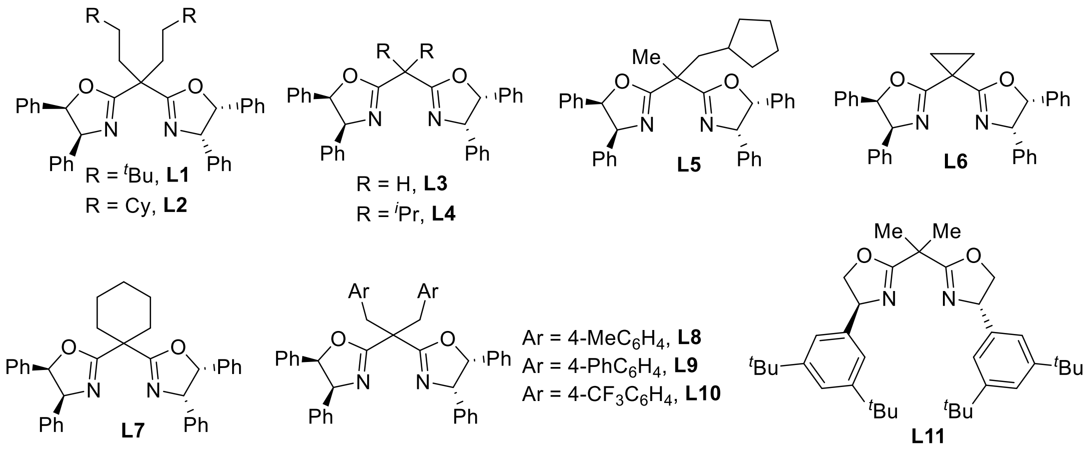

entry	Ligand	1a rsm (%)	yield (%)	ee (%)
1	**L1**	0	81 (74)	92
2	**L2**	0	86	85
3	**L3**	15	61	78
4	**L4**	0	83	73
5	**L5**	0	80	90
6	**L6**	27	40	72
7	**L7**	0	77	66
8	**L8**	48	25	63
9	**L9**	42	27	61
10	**L10**	0	72	73
11	**L11**	0	76	90

[a] The yield was determined by GC using dodecane as internal standard. The ee value was determined by HPLC on a chiral stationary phase.

Table S6. Evaluation of hydride source.[a]

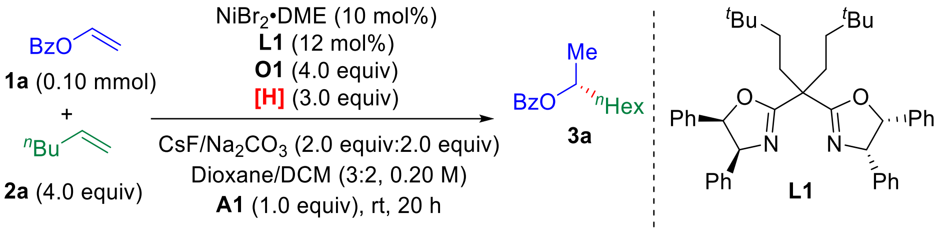

entry	[H]	1a rsm (%)	yield (%)	ee (%)
1	$(MeO)_3SiH$	-	81 (74)	92
2	HBpin	46	15	86
3	$(EtO)_3SiH$	65	34	90
4	$Me(EtO)_2SiH$	84	12	88
5	$Me(MeO)_2SiH$	25	76	90
6	$PhSiH_3$	92	-	-
7	Ph_2SiH_2	75	9	-
8	Ph_3SiH	93	-	-

[a] The yield was determined by GC using dodecane as internal standard. The ee value was determined by HPLC on a chiral stationary phase.

Table S7. Evaluation of Ni-precatalyst.[a]

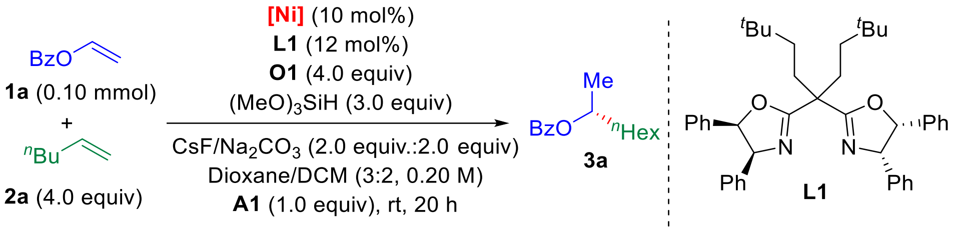

entry	[Ni]	1a rsm (%)	yield (%)	ee (%)
1	$NiBr_2\cdot DME$	-	81 (74)	92
2	$NiBr_2\cdot$diglyme	-	89	90
3	$Ni(cod)_2$	54	29	82
4	$NiBF_4\cdot 6H_2O$	93	-	-
5	$Ni(OAc)_2\cdot 4H_2O$	96	-	-
6	$Ni(NO_3)_2\cdot 6H_2O$	51	53	-
7	$Ni(acac)_2$	94	-	-
8	$NiClO_4\cdot 6H_2O$	37	62	-
9	$Ni(OH)_2$	91	-	-

[a] The yield was determined by GC using dodecane as internal standard. The ee value was determined by HPLC on a chiral stationary phase.

Table S8. Evaluation of commercially available BOX ligands.[a]

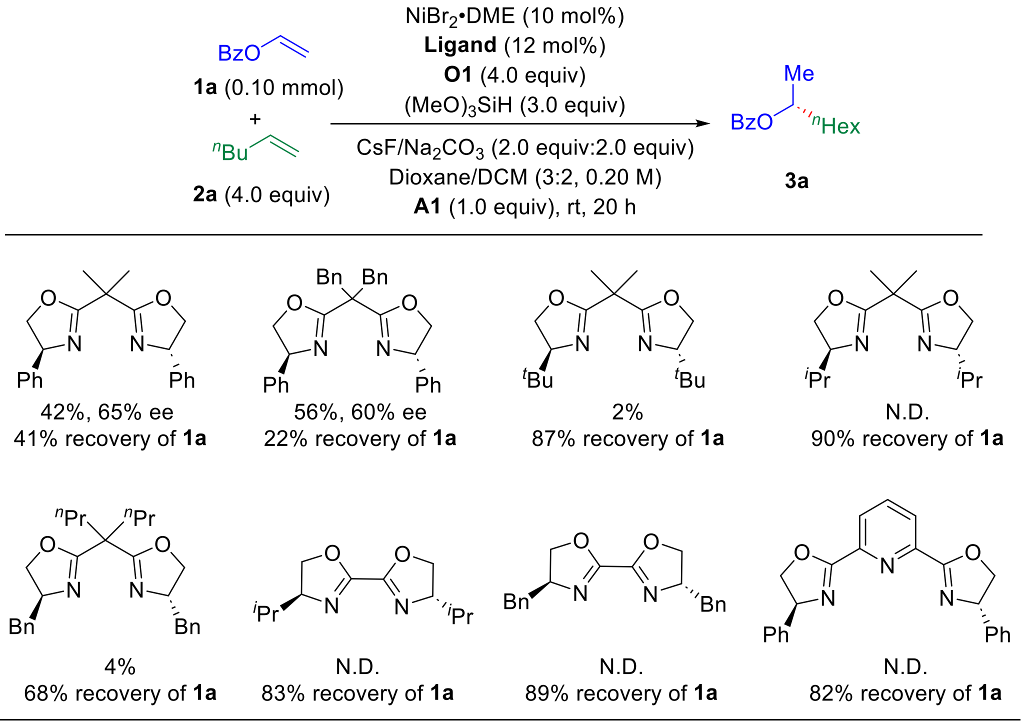

V. Experimental Procedure and Characterization Data

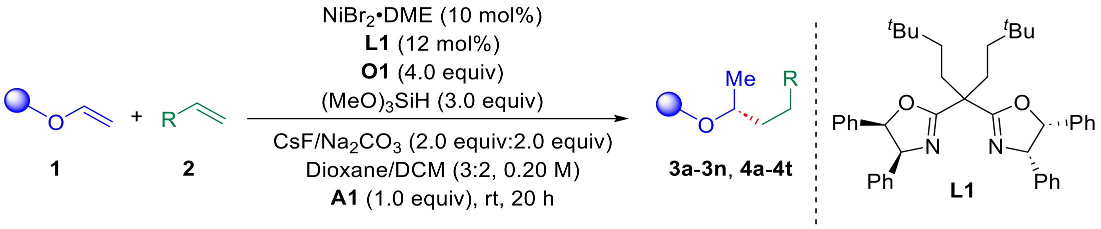

General procedure (F): To an oven-dried 10 mL Teflon-screw cap tube containing a magnetic stir was charged with $NiBr_2$·DME (6.2 mg, 0.02 mmol, 10 mol%) and **L1** (15.0 mg, 0.024 mmol, 12 mol%) in a nitrogen-filled glove-box. Subsequently, anhydrous solvent (Dioxane/DCM, 3:2, 0.20 M) was added. The mixture was stirred for 5-10 min at room temperature. Then CsF/Na_2CO_3 (60.8/42.4 mg, 2.0 equiv/2.0 equiv), olefin **1** (0.20 mmol, 1.0 equiv), **2** (0.80 mmol, 4.0 equiv) were added. **O1** (129.6 mg, 0.80 mmol, 4.0 equiv) and phthalimide **A1** (29.4 mg, 0.2 mmol, 1.0 equiv) were added subsequently. Finally, $(MeO)_3SiH$ (76.0 uL, 0.60 mmol, 3.0 equiv) was added dropwise under N_2 atmosphere, and the reaction was stirred at 25 °C for 20 h at 1400 rpm. After the reaction was completed, the reaction mixture was H_2O (0.5 mL) and EtOAc (3.0 mL). The organic phase was separated, then the aqueous phase was extracted with EtOAc (2 × 3.0 mL). The combined organic phase was dried over Na_2SO_4. The crude mixture was purified by flash column chromatography on silica gel

to give the desired product. Then the enantiomeric excess was determined by HPLC analysis.

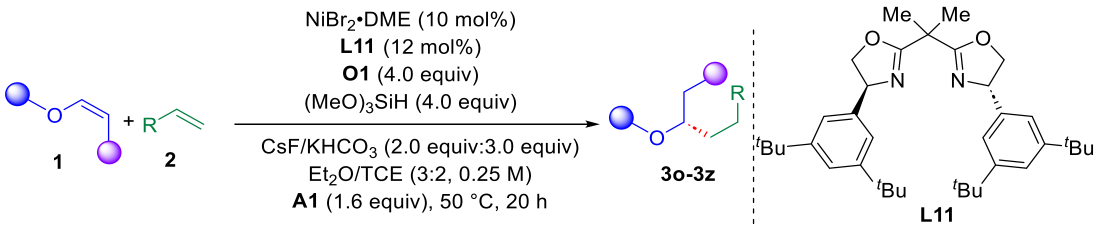

General procedure (G): To an oven-dried 10 mL Teflon-screw cap tube containing a magnetic stir was charged with $NiBr_2$·DME (6.2 mg, 0.02 mmol, 10 mol%) and **L11** (13.4 mg, 0.024 mmol, 12 mol%) in a nitrogen-filled glove-box. Subsequently, anhydrous solvent (Et_2O/TCE, 3:2, 0.25 M) was added. The mixture was stirred for 5-10 min at room temperature. Then CsF/$KHCO_3$ (60.8/60.0 mg, 2.0 equiv/3.0 equiv), olefin **1** (0.20 mmol, 1.0 equiv), **2** (0.80 mmol, 4.0 equiv) were added. **O1** (129.6 mg, 0.80 mmol, 4.0 equiv) and phthalimide **A1** (47.0 mg, 0.32 mmol, 1.6 equiv) were added subsequently. Finally, $(MeO)_3SiH$ (101.1 uL, 0.80 mmol, 4.0 equiv) was added dropwise under N_2 atmosphere, and the reaction was stirred at 50 °C for 20 h at 1400 rpm. After the reaction was completed, the reaction mixture was H_2O (0.5 mL) and EtOAc (3.0 mL). The organic phase was separated, then the aqueous phase was extracted with EtOAc (2 × 3.0 mL). The combined organic phase was dried over Na_2SO_4. The crude mixture was purified by flash column chromatography on silica gel to give the desired product. Then the enantiomeric excess was determined by HPLC analysis.

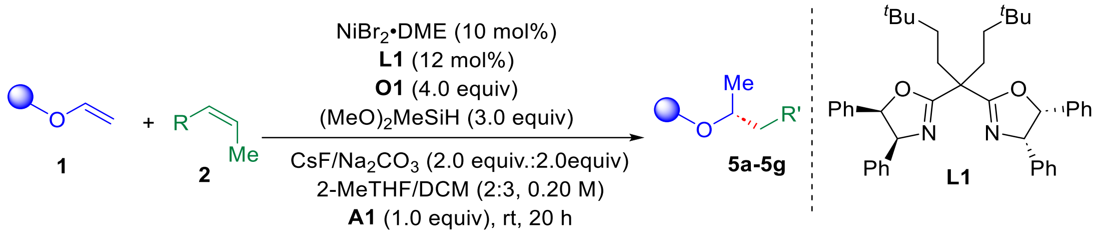

General procedure (H): To an oven-dried 10 mL Teflon-screw cap tube containing a magnetic stir was charged with $NiBr_2$·DME (6.2 mg, 0.02 mmol, 10 mol%) and **L1** (15.0 mg, 0.024 mmol, 12 mol%) in a nitrogen-filled glove-box. Subsequently, anhydrous solvent (2-MeTHF/DCM, 2:3, 0.20 M) was added. The mixture was stirred for 5-10 min at room temperature. Then CsF/Na_2CO_3 (60.8/42.4 mg, 2.0 equiv/2.0 equiv), olefin **1** (0.20 mmol, 1.0 equiv), **2** (0.80 mmol, 4.0 equiv) were added. **O1** (129.6 mg, 0.80 mmol, 4.0 equiv) and phthalimide **A1** (29.4.0 mg, 0.2 mmol, 1.0 equiv) were added subsequently. Finally, $(MeO)_2MeSiH$ (74.0 uL, 0.6 mmol, 3.0 equiv) was added dropwise under N_2 atmosphere, and the reaction was stirred at 25 °C for 20 h at 1400 rpm. After the reaction was completed, the reaction mixture was H_2O (0.5 mL) and EtOAc (3.0 mL). The organic phase was separated, then the aqueous phase was extracted with EtOAc (2 × 3.0 mL). The combined organic phase was dried over

Na_2SO_4. The crude mixture was purified by flash column chromatography on silica gel to give the desired product. Then the enantiomeric excess was determined by HPLC analysis.

(*R*)-Octan-2-yl benzoate (3a)

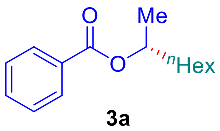

3a

Following **General procedure (F)**, the reaction of **1a** (29.6 mg, 0.2 mmol) with 1-hexene (67.3 mg, 0.8 mmol) afforded **3a** as colorless oil (34.6 mg; 74% yield; 92% ee) by flash column chromatography on silica gel (eluent: PE:EA = 100:1).

^{1}H NMR (400 MHz, $CDCl_3$) δ 8.06 – 8.04 (m, 2H), 7.57 – 7.52 (m, 1H), 7.45 – 7.41 (m, 2H), 5.20 – 5.12 (m, 1H), 1.79 – 1.71 (m, 1H), 1.65 – 1.56 (m, 1H), 1.40 – 1.26 (m, 11H), 0.88 (t, *J* = 6.8 Hz, 3H).

^{13}C NMR (101 MHz, $CDCl_3$) δ 166.4, 132.8, 131.1, 129.6, 128.4, 71.9, 36.2, 31.9, 29.3, 25.5, 22.7, 20.2, 14.2.

HRMS (ESI-TOF) Calcd for $C_{15}H_{22}O_2$ $(M+Na)^+$ 257.1512. Found 257.1508.

HPLC (OJ-H, 0.46*25 cm, 5 µm, hexane/isopropanol = 99.5/0.5, flow rate = 1.0 mL/min, detection at 210 nm) retention time = 5.1 min (major) and 5.5 min (minor).

$[\alpha]^{25}$D = −7.0 (c 0.6, CH_2Cl_2).

(*R*)-Octan-2-yl 4-methylbenzoate (3b)

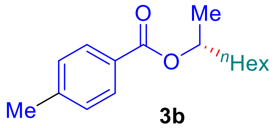

3b

Following **General procedure (F)**, the reaction of **1b** (32.4 mg, 0.2 mmol) with 1-hexene (67.3 mg, 0.8 mmol) afforded **3b** as colorless oil (36.2 mg; 73% yield; 93% ee) by flash column chromatography on silica gel (eluent: PE:EA = 100:1).

^{1}H NMR (400 MHz, $CDCl_3$) δ 7.95 – 7.92 (m, 2H), 7.24 – 7.22 (m, 2H), 5.16 – 5.12 (m, 1H), 2.40 (s, 3H), 1.74 – 1.69 (m, 1H), 1.69 – 1.55 (m, 1H), 1.41 – 1.25 (m, 11H), 0.88 (t, *J* = 6.8 Hz, 3H).

^{13}C NMR (101 MHz, $CDCl_3$) δ 166.4, 143.4, 129.7, 129.1, 128.4, 71.6, 36.2, 31.9, 29.3, 25.5, 22.7, 21.7, 20.2, 14.2.

HRMS (ESI-TOF) Calcd for $C_{16}H_{24}O_2$ $(M+Na)^+$ 271.1669. Found 271.1666.

HPLC (AD-H, 0.46*25 cm, 5 µm, hexane = 100, flow rate = 1.0 mL/min, detection at 230 nm) retention time = 5.6 min (minor) and 5.9 min (major).

$[\alpha]^{25}$D = −30.70 (c 1.0, CH_2Cl_2).

(*R*)-Octan-2-yl 4-(*tert*-butyl)benzoate (3c)

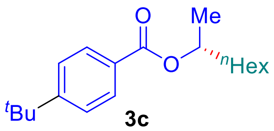

3c

Following **General procedure (F)**, the reaction of **1c** (40.8 mg, 0.2 mmol) with 1-hexene (67.3 mg, 0.8 mmol) afforded **3c** as colorless oil (41.8 mg; 72% yield; 90% ee) by flash column chromatography on silica gel (eluent: PE:EA = 100:1).
^{1}H NMR (600 MHz, $CDCl_3$) δ 7.98 – 7.96 (m, 2H), 7.46 – 7.44 (m, 2H), 5.17 – 5.11 (m, 1H), 1.76 – 1.70 (m, 1H), 1.60 – 1.57 (m, 1H), 1.42 – 1.24 (m, 20H), 0.87 (t, *J* = 6.6 Hz, 3H).
^{13}C NMR (151 MHz, $CDCl_3$) δ 166.4, 156.4, 129.5, 128.3, 125.4, 71.6, 36.3, 35.2, 31.9, 31.3, 29.3, 25.6, 22.7, 20.3, 14.2.
HRMS (ESI-TOF) Calcd for $C_{19}H_{30}O_2$ $(M+Na)^+$ 313.2138. Found 313.2135.
HPLC (AD-H, 0.46*25 cm, 5 μm, hexane/isopropanol = 99.2/0.8, flow rate = 1.0 mL/min, detection at 230 nm) retention time = 6.1 min (major) and 6.8 min (minor).
$[\alpha]^{25}_{D}$ = −27.91 (c 0.7, CH_2Cl_2).

(*R*)-Octan-2-yl 4-methoxybenzoate (3d)

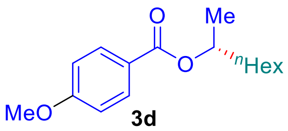

3d

Following **General procedure (F)**, the reaction of **1d** (35.6 mg, 0.2 mmol) with 1-hexene (67.3 mg, 0.8 mmol) afforded **3d** as colorless oil (37.3 mg; 71% yield; 93% ee) by flash column chromatography on silica gel (eluent: PE:EA = 100:1).
^{1}H NMR (400 MHz, $CDCl_3$) δ 8.01 – 7.97 (m, 2H), 6.93 – 6.89 (m, 2H), 5.16 – 5.08 (m, 1H), 3.85 (s, 3H), 1.75 – 1.69 (m, 1H), 1.61 – 1.54 (m, 1H), 1.40 – 1.25 (m, 11H), 0.87 (t, *J* = 6.4 Hz, 3H).
^{13}C NMR (101 MHz, $CDCl_3$) δ 166.2, 163.3, 131.6, 123.6, 113.6, 71.5, 55.6, 36.3, 31.9, 29.3, 25.6, 22.7, 20.3, 14.2.
HRMS (ESI-TOF) Calcd for $C_{16}H_{24}O_3$ $(M+Na)^+$ 287.1618. Found 287.1615.
HPLC (AD-H, 0.46*25 cm, 5 μm, hexane/isopropanol = 99.2/0.8, flow rate = 0.5 mL/min, detection at 230 nm) retention time = 13.3 min (major) and 14.3 min (minor).
$[\alpha]^{25}_{D}$ = −26.94 (c 0.4, CH_2Cl_2).

(*R*)-Octan-2-yl 4-(methylthio)benzoate (3e)

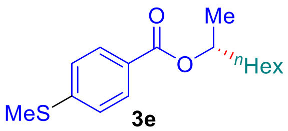

3e

Following **General procedure (F)**, the reaction of **1e** (38.8 mg, 0.2 mmol) with 1-hexene (67.3 mg, 0.8 mmol) afforded **3e** as colorless oil (40.9 mg; 73% yield; 91% ee) by flash column chromatography on silica gel (eluent: PE:EA = 100:1).
^{1}H NMR (400 MHz, $CDCl_3$) δ 7.93 – 7.90 (m, 2H), 7.25 – 7.21 (m, 2H), 5.15 – 5.07 (m, 1H), 2.49 (s, 3H), 1.74 – 1.67 (m, 1H), 1.60 – 1.53 (m, 1H), 1.38 – 1.24 (m, 11H), 0.85 (t, *J* = 6.8 Hz, 3H).
^{13}C NMR (101 MHz, $CDCl_3$) δ 166.1, 145.2, 129.9, 127.2, 125.0, 71.8, 36.2, 31.9, 29.3, 25.5, 22.7, 20.2, 15.0, 14.2.
HRMS (ESI-TOF) Calcd for $C_{16}H_{24}O_2S$ $(M+Na)^+$ 303.1389. Found 303.1388.
HPLC (AD-H, 0.46*25 cm, 5 µm, hexane/isopropanol = 99/1, flow rate = 1.0 mL/min, detection at 254 nm) retention time = 6.9 min (major) and 7.3 min (minor).
$[\alpha]^{25}_D = -21.54$ (c 0.4, CH_2Cl_2).

(*R*)-Octan-2-yl benzo[*d*][1,3]dioxole-5-carboxylate (3f)

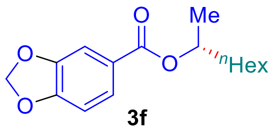

3f

Following **General procedure (F)**, the reaction of **1f** (38.4 mg, 0.2 mmol) with 1-hexene (67.3 mg, 0.8 mmol) afforded **3f** as colorless oil (38.9 mg; 70% yield; 87% ee) by flash column chromatography on silica gel (eluent: PE:EA = 100:1).
^{1}H NMR (400 MHz, $CDCl_3$) δ 7.64 (dd, *J* = 8.2, 1.7 Hz, 1H), 7.46 (d, *J* = 1.7 Hz, 1H), 6.83 (d, *J* = 8.2 Hz, 1H), 6.03 (s, 2H), 5.14 – 5.06 (m, 1H), 1.74 – 1.66 (m, 1H), 1.61 – 1.53 (m, 1H), 1.39 – 1.25 (m, 11H), 0.87 (t, *J* = 6.4 Hz, 3H).
^{13}C NMR (101 MHz, $CDCl_3$) δ 165.7, 151.5, 147.8, 125.3, 125.1, 109.6, 108.0, 101.9, 71.8, 36.2, 31.9, 29.3, 25.5, 22.7, 20.2, 14.2.
HRMS (ESI-TOF) Calcd for $C_{16}H_{22}O_4$ $(M+Na)^+$ 301.1410. Found 301.1410.
HPLC (OJ-H, 0.46*25 cm, 5 µm, hexane/isopropanol = 99.5/0.5, flow rate = 1.0 mL/min, detection at 254 nm) retention time = 10.7 min (minor) and 12.4 min (major).
$[\alpha]^{25}_D = -27.50$ (c 0.5, CH_2Cl_2).

(*R*)-Octan-2-yl [1,1'-biphenyl]-4-carboxylate (3g)

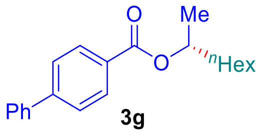

3g

Following **General procedure (F)**, the reaction of **1g** (44.8 mg, 0.2 mmol) with 1-hexene (67.3 mg, 0.8 mmol) afforded **3g** as colorless oil (43.4 mg; 70% yield; 94% ee) by flash column chromatography on silica gel (eluent: PE:EA = 100:1).
^{1}H NMR (400 MHz, $CDCl_3$) δ 8.13 – 8.10 (m, 2H), 7.68 – 7.61 (m, 4H), 7.49 – 7.37 (m, 3H), 5.23 – 5.15 (m, 1H), 1.80 – 1.72 (m, 1H), 1.67 – 1.59 (m, 1H), 1.44 – 1.26 (m, 11H), 0.89 (t, *J* = 6.8 Hz, 3H).

^{13}C NMR (101 MHz, $CDCl_3$) δ 166.2, 145.6, 140.3, 130.2, 129.8, 129.0, 128.2, 127.4, 127.1, 71.9, 36.2, 31.9, 29.3, 25.6, 22.7, 20.2, 14.2.
HRMS (ESI-TOF) Calcd for $C_{21}H_{26}O_2$ $(M+Na)^+$ 333.1825. Found 333.1821.
HPLC (OJ-H, 0.46*25 cm, 5 μm, hexane/isopropanol = 99.2/0.8, flow rate = 0.5 mL/min, detection at 254 nm) retention time = 12.6 min (minor) and 13.0 min (major).
$[\alpha]^{25}D$ = −37.60 (c 1.0, CH_2Cl_2).

(*R*)-Octan-2-yl 4-fluorobenzoate (3h)

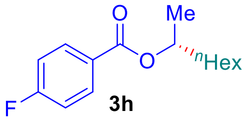

3h

Following **General procedure (F)**, the reaction of **1h** (33.2 mg, 0.2 mmol) with 1-hexene (67.3 mg, 0.8 mmol) afforded **3h** as colorless oil (37.0 mg; 75% yield; 90% ee) by flash column chromatography on silica gel (eluent: PE:EA = 100:1).
^{1}H NMR (400 MHz, $CDCl_3$) δ 8.07 – 8.03 (m, 2H), 7.12 – 7.07 (m, 2H), 5.18 – 5.09 (m, 1H), 1.75 – 1.68 (m, 1H), 1.62 – 1.55 (m, 1H), 1.40 – 1.25 (m, 11H), 0.87 (t, J = 6.4 Hz, 3H).
^{13}C NMR (101 MHz, $CDCl_3$) δ 165.8 (d, J_{C-F} = 254.5 Hz), 165.4, 132.1 (d, J_{C-F} = 9.1 Hz), 127.3 (d, J_{C-F} = 3.0 Hz), 115.6 (d, J_{C-F} = 21.2 Hz), 72.1, 36.2, 31.9, 29.3, 25.5, 22.7, 20.2, 14.2.
^{19}F NMR (376 MHz, $CDCl_3$) δ -106.30 – -106.38 (m).
HRMS (ESI-TOF) Calcd for $C_{15}H_{21}FO_2$ $(M+Na)^+$ 275.1418. Found 275.1412.
HPLC (AD-H, 0.46*25 cm, 5 μm, hexane = 100, flow rate = 0.5 mL/min, detection at 254 nm) retention time = 15.0 min (major) and 16.5 min (minor).
$[\alpha]^{25}D$ = −9.40 (c 1.0, CH_2Cl_2).

(*R*)-Octan-2-yl 4-chlorobenzoate (3i)

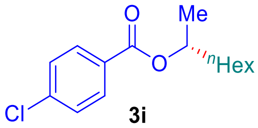

3i

Following **General procedure (F)**, the reaction of **1i** (36.4 mg, 0.2 mmol) with 1-hexene (67.3 mg, 0.8 mmol) afforded **3i** as colorless oil (38.0 mg; 71% yield; 90% ee) by flash column chromatography on silica gel (eluent: PE:EA = 100:1).
^{1}H NMR (400 MHz, $CDCl_3$) δ 7.98 – 7.96 (m, 2H), 7.42 – 7.39 (m, 2H), 5.16 – 5.11 (m, 1H), 1.74 – 1.68 (m, 1H), 1.64 – 1.56 (m, 1H), 1.40 – 1.26 (m, 11H), 0.87 (t, J = 6.8 Hz, 3H).
^{13}C NMR (101 MHz, $CDCl_3$) δ 165.5, 139.2, 131.1, 129.5, 128.8, 72.3, 36.2, 31.9, 29.3, 25.5, 22.7, 20.2, 14.2.
HRMS (ESI-TOF) Calcd for $C_{15}H_{21}ClO_2$ $(M+Na)^+$ 291.1122. Found 291.1120.
HPLC (IA, 0.46*25 cm, 5 μm, hexane/isopropanol = 99.8/0.2, flow rate = 0.5 mL/min, detection at 254 nm) retention time = 17.1 min (major) and 18.1 min (minor).
$[\alpha]^{25}D$ = −7.5 (c 0.4, CH_2Cl_2).

(*R*)-Octan-2-yl 4-(trifluoromethyl)benzoate (3j)

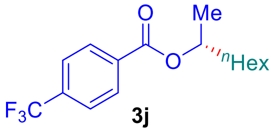

3j

Following **General procedure (F)**, the reaction of **1j** (43.2 mg, 0.2 mmol) with 1-hexene (67.3 mg, 0.8 mmol) afforded **3j** as colorless oil (36.8 mg; 61% yield; 80% ee) by flash column chromatography on silica gel (eluent: PE:EA = 100:1).
^{1}H NMR (600 MHz, $CDCl_3$) δ 8.15 (d, *J* = 7.8 Hz, 2H), 7.70 (d, *J* = 7.8 Hz, 2H), 5.20 – 5.15 (m, 1H), 1.77 – 1.72 (m, 1H), 1.65 – 1.59 (m, 1H), 1.42 – 1.25 (m, 11H), 0.87 (t, *J* = 6.1 Hz, 3H).
^{13}C NMR (151 MHz, $CDCl_3$) δ 165.2, 134.4 (q, J_{C-F} = 33.2 Hz), 134.3, 130.0, 125.5 (q, J_{C-F} = 3.0 Hz), 123.8 (q, J_{C-F} = 273.3 Hz), 72.7, 36.1, 31.9, 29.3, 25.5, 22.7, 20.2, 14.2.
^{19}F NMR (565 MHz, $CDCl_3$) δ -63.07 (s).
HRMS (ESI-TOF) Calcd for $C_{16}H_{21}F_3O_2$ $(M+Na)^+$ 325.1386. Found 325.1385.
HPLC (OJ-H, 0.46*25 cm, 5 μm, hexane/isopropanol = 99.5/0.5, flow rate = 0.5 mL/min, detection at 230 nm) retention time = 8.4 min (minor) and 9.2 min (major).
$[\alpha]^{25}D$ = −18.31 (c 0.8, CH_2Cl_2).

(*R*)-Octan-2-yl 2-naphthoate (3k)

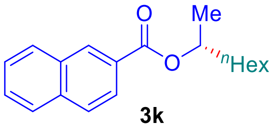

3k

Following **General procedure (F)**, the reaction of **1k** (39.6 mg, 0.2 mmol) with 1-hexene (67.3 mg, 0.8 mmol) afforded **3k** as colorless oil (38.1 mg; 67% yield; 87% ee) by flash column chromatography on silica gel (eluent: PE:EA = 50:1).
^{1}H NMR (600 MHz, $CDCl_3$) 8.61 (dd, *J* = 1.6, 0.8 Hz, 1H), 8.08 (dd, *J* = 8.6, 1.7 Hz, 1H), 7.97 – 7.95 (m, 1H), 7.88 (dd, *J* = 8.3, 1.6 Hz, 2H), 7.60 – 7.53 (m, 2H), 5.26 – 5.21 (m, 1H), 1.84 – 1.78 (m, 1H), 1.69 – 1.63 (m, 1H), 1.47 – 1.27 (m, 11H), 0.89 (t, *J* = 6.9 Hz, 3H).
^{13}C NMR (151 MHz, $CDCl_3$) δ 166.5, 135.6, 132.7, 131.0, 129.5, 128.3, 128.20, 128.16, 127.9, 126.7, 125.4, 72.1, 36.3, 31.9, 29.3, 25.6, 22.7, 20.3, 14.2.
HRMS (ESI-TOF) Calcd for $C_{19}H_{24}O_2$ $(M+Na)^+$ 307.1669. Found 307.1666.
HPLC (AD-H, 0.46*25 cm, 5 μm, hexane/isopropanol = 99/1, flow rate = 1.0 mL/min, detection at 254 nm) retention time = 6.7 min (major) and 7.6 min (minor).
$[\alpha]^{25}D$ = −23.7 (c 1.0, CH_2Cl_2).

(*R*)-Octan-2-yl 2-methylbenzoate (3l)

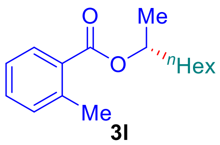

3l

Following **General procedure (F)**, the reaction of **1l** (32.4 mg, 0.2 mmol) with 1-hexene (67.3 mg, 0.8 mmol) afforded **3l** as colorless oil (27.8 mg; 56% yield; 87% ee) by flash column chromatography on silica gel (eluent: PE:EA = 100:1).

^{1}H NMR (400 MHz, $CDCl_3$) δ 7.88 (dd, *J* = 8.1, 1.6 Hz, 1H), 7.40 – 7.30 (m, 1H), 7.26 – 7.22 (m, 2H), 5.17 – 5.12 (m, 1H), 2.59 (s, 3H), 1.74 – 1.69 (m, 1H), 1.63 – 1.57 (m, 1H), 1.42 – 1.24 (m, 11H), 0.88 (t, *J* = 6.6 Hz, 3H).

^{13}C NMR (101 MHz, $CDCl_3$) δ 167.6, 139.9, 131.8, 131.7, 130.6, 130.5, 125.8, 71.7, 36.2, 31.9, 29.3, 25.6, 22.7, 21.9, 20.2, 14.2.

HRMS (ESI-TOF) Calcd for $C_{16}H_{24}O_2$ $(M+Na)^+$ 271.1669. Found 271.1666.

HPLC (IA, 0.46*25 cm, 5 µm, hexane/isopropanol = 99.5/0.5, flow rate = 0.5 mL/min, detection at 254 nm) retention time = 14.5 min (major) and 16.4 min (minor).

$[\alpha]^{25}D$ = −23.61 (c 0.4, CH_2Cl_2).

(*R*)-6-Phenylhexan-2-yl 5-methylthiophene-2-carboxylate (3m)

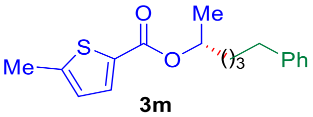

3m

Following **General procedure (F)**, the reaction of **1m** (33.6 mg, 0.2 mmol) with **2d** (105.7 mg, 0.8 mmol) afforded **3m** as colorless oil (37.5 mg; 62% yield; 91% ee) by flash column chromatography on silica gel (eluent: PE:EA = 50:1).

^{1}H NMR (400 MHz, $CDCl_3$) δ 7.59 (d, *J* = 3.7 Hz, 1H), 7.28 – 7.24 (m, 2H), 7.19 – 7.14 (m, 3H), 6.76 (dd, *J* = 3.7, 1.2 Hz, 1H), 5.10 – 5.05 (m, 1H), 2.61 (t, *J* = 7.7 Hz, 2H), 2.52 (s, 3H), 1.78 – 1.57 (m, 4H), 1.50 – 1.38 (m, 2H), 1.31 (d, *J* = 6.2 Hz, 3H).

^{13}C NMR (101 MHz, $CDCl_3$) δ 162.1, 147.7, 142.6, 133.6, 132.0, 128.5, 128.4, 126.4, 125.8, 71.8, 36.0, 35.9, 31.4, 25.1, 20.2, 15.9.

HRMS (ESI-TOF) Calcd for $C_{18}H_{22}O_2S$ $(M+Na)^+$ 325.1233. Found 325.1229.

HPLC (OD-H, 0.46*25 cm, 5 µm, hexane/isopropanol = 99/1, flow rate = 1.0 mL/min, detection at 230 nm) retention time = 7.6 min (major) and 11.5 min (minor).

$[\alpha]^{25}D$ = −40.70 (c 1.0, CH_2Cl_2).

(*R*)-Octan-2-yl 1-methyl-1*H*-indole-2-carboxylate (3n)

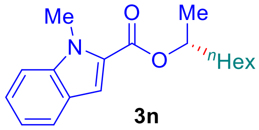

3n

Following **General procedure (F)**, the reaction of **1n** (40.2 mg, 0.2 mmol) with 1-hexene (67.3 mg, 0.8 mmol) afforded **3n** as colorless oil (28.7 mg; 50% yield; 90% ee) by flash column chromatography on silica gel (eluent: PE:EA = 20:1).

^{1}H NMR (400 MHz, $CDCl_3$) δ 7.69 – 7.66 (m, 1H), 7.40 – 7.33 (m, 2H), 7.30 (s, 1H), 7.17 – 7.13 (m, 1H), 5.19 – 5.12 (m, 1H), 4.09 (s, 3H), 1.80 – 1.71 (m, 1H), 1.67 – 1.58 (m, 1H), 1.46 – 1.26 (m, 11H), 0.88 (t, *J* = 6.8 Hz, 3H).
^{13}C NMR (101 MHz, $CDCl_3$) δ 162.1, 139.8, 128.6, 126.0, 124.9, 122.6, 120.6, 110.3, 110.0, 71.5, 36.3, 31.9, 31.8, 29.3, 25.6, 22.7, 20.3, 14.2.
HRMS (ESI-TOF) Calcd for $C_{18}H_{26}NO_2$ $(M+H)^+$ 288.1958. Found 288.1956.
HPLC (OJ-H, 0.46*25 cm, 5 μm, hexane/isopropanol = 99.2/0.8, flow rate = 1.0 mL/min, detection at 230 nm) retention time = 8.9 min (major) and 9.9 min (minor).
$[\alpha]^{25}D$ = −38.14 (c 1.0, CH_2Cl_2).

(*R*)-9-Phenoxynonan-3-yl benzoate (3o)

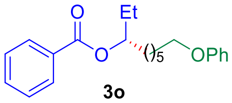

3o

Following **General procedure (G)**, the reaction of **1o** (32.4 mg, 0.2 mmol) with **2i** (140.9 mg, 0.8 mmol) afforded **3o** as colorless oil (38.1 mg; 56% yield; 92% ee) by flash column chromatography on silica gel (eluent: PE:EA = 50:1).
^{1}H NMR (400 MHz, $CDCl_3$) δ 8.08 – 8.05 (m, 2H), 7.58 – 7.54 (m, 1H), 7.47 – 7.43 (m, 2H), 7.29 – 7.25 (m, 2H), 6.95 – 6.88 (m, 3H), 5.13 – 5.07 (m, 1H), 3.94 (t, *J* = 6.5 Hz, 2H), 1.79 – 1.68 (m, 6H), 1.46 – 1.39 (m, 6H), 0.96 (t, *J* = 7.4 Hz, 3H).
^{13}C NMR (101 MHz, $CDCl_3$) δ 166.5, 159.2, 132.8, 131.0, 129.7, 129.5, 128.4, 120.6, 114.6, 76.2, 67.9, 33.8, 29.4, 29.3, 27.2, 26.1, 25.4, 9.8.
HRMS (ESI-TOF) Calcd for $C_{22}H_{28}O_3$ $(M+Na)^+$ 363.1931. Found 363.1938.
HPLC (IC, 0.46*25 cm, 5 μm, hexane/isopropanol = 98/2, flow rate = 1.0 mL/min, detection at 230 nm) retention time = 5.2 min (minor) and 5.6 min (major).
$[\alpha]^{25}D$ = −8.9 (c 1.0, CH_2Cl_2).

(*R*)-11-Phenoxyundecan-5-yl benzoate (3p)

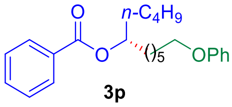

3p

Following **General procedure (G)**, the reaction of **1p** (38.0 mg, 0.2 mmol) with **2i** (140.9 mg, 0.8 mmol) afforded **3p** as colorless oil (50.1 mg; 68% yield; 95% ee) by flash column chromatography on silica gel (eluent: PE:EA = 50:1).
^{1}H NMR (400 MHz, $CDCl_3$) δ 8.07 – 8.04 (m, 2H), 7.56 – 7.53 (m, 1H), 7.46 – 7.42 (m, 2H), 7.28 – 7.24 (m, 2H), 6.89 – 6.86 (m, 3H), 5.17 – 5.11 (m, 1H), 3.92 (t, *J* = 6.5 Hz, 2H), 1.78 – 1.65 (m, 6H), 1.46 – 1.32 (m, 10H), 0.89 (t, *J* = 6.8 Hz, 3H).
^{13}C NMR (101 MHz, $CDCl_3$) δ 166.5, 159.2, 132.8, 131.0, 129.7, 129.5, 128.4, 120.6, 114.6, 75.1, 67.9, 34.3, 34.1, 29.4, 29.3, 27.7, 26.1, 25.4, 22.8, 14.1.
HRMS (ESI-TOF) Calcd for $C_{24}H_{32}O_3$ $(M+Na)^+$ 391.2244. Found 391.2239.
HPLC (IC, 0.46*25 cm, 5 μm, hexane/isopropanol = 98/2, flow rate = 1.0 mL/min, detection at 254 nm) retention time = 4.5 min (minor) and 5.0 min (major).
$[\alpha]^{25}D$ = −4.80 (c 1.0, CH_2Cl_2).

(*R*)-12-Phenoxydodecan-6-yl benzoate (3q)

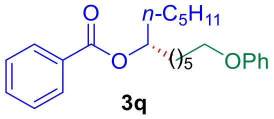

3q

Following **General procedure (G)**, the reaction of **1q** (40.8 mg, 0.2 mmol) with **2i** (140.9 mg, 0.8 mmol) afforded **3q** as colorless oil (51.2 mg; 67% yield; 95% ee) by flash column chromatography on silica gel (eluent: PE:EA = 50:1).

^{1}H NMR (400 MHz, $CDCl_3$) δ 8.07 – 8.04 (m, 2H), 7.54 – 7.52 (m, 1H), 7.45 – 7.41 (m, 2H), 7.28 – 7.24 (m, 2H), 6.91 – 6.86 (m, 3H), 5.17 – 5.11 (m, 1H), 3.92 (t, *J* = 6.5 Hz, 2H), 1.78 – 1.65 (m, 6H), 1.46 – 1.27 (m, 12H), 0.87 (t, *J* = 6.8 Hz, 3H).

^{13}C NMR (101 MHz, $CDCl_3$) δ 166.5, 159.2, 132.8, 130.9, 129.7, 129.5, 128.4, 120.6, 114.6, 75.1, 67.8, 34.31, 34.27, 31.9, 29.4, 29.3, 26.1, 25.4, 25.2, 22.7, 14.2.

HRMS (ESI-TOF) Calcd for $C_{25}H_{34}O_3$ $(M+Na)^+$ 405.2400. Found 405.2397.

HPLC (IC, 0.46*25 cm, 5 μm, hexane/isopropanol = 98/2, flow rate = 1.0 mL/min, detection at 230 nm) retention time = 4.4 min (minor) and 4.8 min (major).

$[\alpha]^{25}D = -3.10$ (c 1.0, CH_2Cl_2).

Following **General procedure (G)**, the reaction of (*E*)-**1q** (40.8 mg, 0.2 mmol) with **2i** (140.9 mg, 0.8 mmol) afforded trace amount of **3q** determined by ^{1}H NMR of the crude mixture using mesitylene as internal standard.

(*R*)-1-Phenoxytridecan-7-yl benzoate (3r)

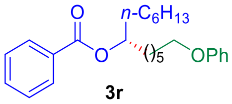

3r

Following **General procedure (G)**, the reaction of **1r** (43.6 mg, 0.2 mmol) with **2i** (140.9 mg, 0.8 mmol) afforded **3r** as colorless oil (47.6 mg; 60% yield; 92% ee) by flash column chromatography on silica gel (eluent: PE:EA = 50:1).

^{1}H NMR (400 MHz, $CDCl_3$) δ 8.07 – 8.04 (m, 2H), 7.57 – 7.52 (m, 1H), 7.45 – 7.42 (m, 2H), 7.28 – 7.24 (m, 2H), 6.92 – 6.86 (m, 3H), 5.17 – 5.11 (m, 1H), 3.92 (t, *J* = 6.5 Hz, 2H), 1.76 – 1.66 (m, 6H), 1.45 – 1.25 (m, 14H), 0.86 (t, *J* = 6.6 Hz, 3H).

^{13}C NMR (101 MHz, $CDCl_3$) δ 166.5, 159.2, 132.8, 130.9, 129.7, 129.5, 128.4, 120.6, 114.6, 75.2, 67.8, 34.4, 34.3, 31.9, 29.44, 29.38, 29.3, 26.1, 25.5, 25.4, 22.7, 14.2.

HRMS (ESI-TOF) Calcd for $C_{26}H_{36}O_3$ $(M+Na)^+$ 419.2557. Found 419.2553.

HPLC (IC, 0.46*25 cm, 5 μm, hexane/isopropanol = 98/2, flow rate = 1.0 mL/min, detection at 210 nm) retention time = 4.7 min (minor) and 5.2 min (major).

$[\alpha]^{25}D = -4.30$ (c 1.0, CH_2Cl_2).

(*R*)-12-Phenoxydodecan-6-yl 4-methylbenzoate (3s)

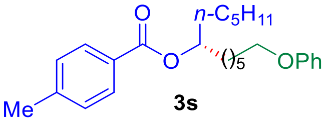

3s

Following **General procedure (G)**, the reaction of **1s** (43.6 mg, 0.2 mmol) with **2i** (140.9 mg, 0.8 mmol) afforded **3s** as colorless oil (55.4 mg; 70% yield; 95% ee) by flash column chromatography on silica gel (eluent: PE:EA = 50:1).
^{1}H NMR (400 MHz, $CDCl_3$) δ 7.95 – 7.93 (m, 2H), 7.28 – 7.22 (m, 4H), 6.91 – 6.86 (m, 3H), 5.16 – 5.09 (m, 1H), 3.92 (t, J = 6.5 Hz, 2H), 2.40 (s, 3H), 1.76 – 1.64 (m, 6H), 1.44 – 1.27 (m, 12H), 0.87 (t, J = 6.8 Hz, 3H).
^{13}C NMR (101 MHz, $CDCl_3$) δ 166.6, 159.2, 143.4, 129.7, 129.5, 129.1, 128.2, 120.5, 114.5, 74.8, 67.8, 34.33, 34.28, 31.9, 29.4, 29.3, 26.1, 25.4, 25.1, 22.7, 21.7, 14.1.
HRMS (ESI-TOF) Calcd for $C_{26}H_{36}O_3$ $(M+Na)^+$ 419.2557. Found 419.2552.
HPLC (IC, 0.46*25 cm, 5 μm, hexane/isopropanol = 99.2/0.8, flow rate = 1.0 mL/min, detection at 210 nm) retention time = 4.8 min (minor) and 6.0 min (major).
$[\alpha]^{25}D = -5.80$ (c 1.0, CH_2Cl_2).

(*R*)-12-Phenoxydodecan-6-yl 4-(methylthio)benzoate (3t)

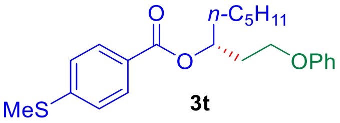

Following **General procedure (G)**, the reaction of **1t** (50.0 mg, 0.2 mmol) with **2i** (140.9 mg, 0.8 mmol) afforded **3t** as colorless oil (52.2 mg; 61% yield; 93% ee) by flash column chromatography on silica gel (eluent: PE:EA = 50:1).
^{1}H NMR (400 MHz, $CDCl_3$) δ 7.96 – 7.93 (m, 2H), 7.29 – 7.2 (m, 4H), 6.91 – 6.86 (m, 3H), 5.15 – 5.08 (m, 1H), 3.92 (t, J = 6.5 Hz, 2H), 2.50 (s, 3H), 1.77 – 1.63 (m, 6H), 1.42 – 1.27 (m, 12H), 0.87 (t, J = 6.8 Hz, 3H).
^{13}C NMR (101 MHz, $CDCl_3$) δ 166.2, 159.2, 145.2, 130.0, 129.5, 127.1, 125.1, 120.6, 114.6, 75.0, 67.8, 34.31, 34.26, 31.9, 29.4, 29.3, 26.1, 25.4, 25.1, 22.7, 15.0, 14.1.
HRMS (ESI-TOF) Calcd for $C_{26}H_{36}O_3S$ $(M+Na)^+$ 451.2277. Found 451.2274.
HPLC (IC, 0.46*25 cm, 5 μm, hexane/isopropanol = 98/2, flow rate = 1.0 mL/min, detection at 254 nm) retention time = 6.0 min (minor) and 10.3 min (major).
$[\alpha]^{25}D = -6.70$ (c 1.0, CH_2Cl_2).

(*R*)-12-Phenoxydodecan-6-yl 4-fluorobenzoate (3u)

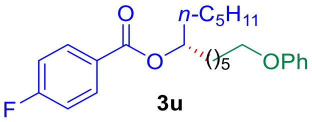

Following **General procedure (G)**, the reaction of **1u** (44.4 mg, 0.2 mmol) with **2i** (140.9 mg, 0.8 mmol) afforded **3u** as colorless oil (45.6 mg; 57% yield; 93% ee) by flash column chromatography on silica gel (eluent: PE:EA = 50:1).
^{1}H NMR (400 MHz, $CDCl_3$) δ 8.07 – 8.04 (m, 2H), 7.28 – 7.24 (m, 2H), 7.12 – 7.07 (m, 2H), 6.93 – 6.86 (m, 3H), 5.14 – 5.10 (m, 1H), 3.92 (t, J = 6.5 Hz, 2H), 1.77 – 1.64 (m, 6H), 1.46 – 1.27 (m, 12H), 0.87 (t, J = 6.7 Hz, 3H).
^{13}C NMR (101 MHz, $CDCl_3$) δ 165.8 (d, J = 254.5 Hz), 165.5, 159.2, 132.1 (d, J = 9.1 Hz), 129.5, 127.1 (d, J = 2.0 Hz), 120.6, 115.5 (d, J = 22.1 Hz), 114.6, 75.3, 67.8, 34.3, 34.2, 31.8, 29.4, 29.3, 26.1, 25.4, 25.1, 22.7, 14.1.

^{19}F NMR (376 MHz, $CDCl_3$) δ -106.16 – -106.24 (m).
HRMS (ESI-TOF) Calcd for $C_{25}H_{33}FO_3$ $(M+Na)^+$ 423.2306. Found 423.2301.
HPLC (IC, 0.46*25 cm, 5 μm, hexane/isopropanol = 98/2, flow rate = 1.0 mL/min, detection at 210 nm) retention time = 5.6 min (minor) and 6.7 min (major).
$[\alpha]^{25}D = -3.90$ (c 1.0, CH_2Cl_2).

(*R*)-12-Phenoxydodecan-6-yl propionate (3v)

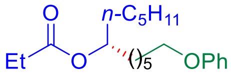

3v

Following **General procedure (G)**, the reaction of **1v** (31.2 mg, 0.2 mmol) with **2i** (140.9 mg, 0.8 mmol) afforded **3v** as colorless oil (37.4 mg; 56% yield; 86% ee) by flash column chromatography on silica gel (eluent: PE:EA = 50:1).
^{1}H NMR (400 MHz, $CDCl_3$) δ 7.29 – 7.24 (m, 2H), 6.94 – 6.86 (m, 3H), 4.90 – 4.84 (m, 1H), 3.93 (t, *J* = 6.5 Hz, 2H), 2.30 (q, *J* = 7.6 Hz, 2H), 1.80 – 1.72 (m, 2H), 1.55 – 1.41 (m, 6H), 1.38 – 1.23 (m, 10H), 1.13 (t, *J* = 7.6 Hz, 3H), 0.86 (t, *J* = 6.7 Hz, 3H).
^{13}C NMR (101 MHz, $CDCl_3$) δ 174.5, 159.2, 129.5, 120.6, 114.6, 74.2, 67.9, 34.3, 34.2, 31.9, 29.4, 29.3, 28.1, 26.1, 25.4, 25.1, 22.7, 14.1, 9.5.
HRMS (ESI-TOF) Calcd for $C_{21}H_{34}O_3$ $(M+Na)^+$ 357.2400. Found 357.2397.
HPLC (OD-H, 0.46*25 cm, 5 μm, hexane/isopropanol = 99/1, flow rate = 1.0 mL/min, detection at 210 nm) retention time = 10.7 min (minor) and 12.1 min (major).
$[\alpha]^{25}D = -5.40$ (c 1.0, CH_2Cl_2).

(*R*)-12-Phenoxydodecan-6-yl cyclopropanecarboxylate (3w)

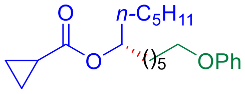

3w

Following **General procedure (G),** the reaction of **1w** (33.6 mg, 0.2 mmol) with **2i** (140.9 mg, 0.8 mmol) afforded **3w** as colorless oil (33.9 mg; 49% yield; 92% ee) by flash column chromatography on silica gel (eluent: PE:EA = 50:1).
^{1}H NMR (400 MHz, $CDCl_3$) δ 7.29 – 7.24 (m, 2H), 6.94 – 6.86 (m, 3H), 4.89 – 4.83 (m, 1H), 3.93 (t, *J* = 6.5 Hz, 2H), 1.80 – 1.73 (m, 2H), 1.60 – 1.41 (m, 7H), 1.40 – 1.22 (m, 10H), 0.99 – 0.95 (m, 2H), 0.87 (t, *J* = 6.7 Hz, 3H), 0.84 – 0.79 (m, 2H).
^{13}C NMR (101 MHz, $CDCl_3$) δ 174.8, 159.2, 129.5, 120.6, 114.6, 74.3, 67.9, 34.24, 34.20, 31.8, 29.4, 29.3, 26.1, 25.3, 25.1, 22.7, 14.1, 13.2, 8.2.
HRMS (ESI-TOF) Calcd for $C_{22}H_{34}O_3$ $(M+Na)^+$ 369.2400. Found 369.2395.
HPLC (OD-H, 0.46*25 cm, 5 μm, hexane/isopropanol = 98/2, flow rate = 1.0 mL/min, detection at 210 nm) retention time = 7.0 min (minor) and 9.9 min (major).
$[\alpha]^{25}D = -4.40$ (c 1.0, CH_2Cl_2).

(*R*)-12-Phenoxydodecan-6-yl cyclobutanecarboxylate (3s)

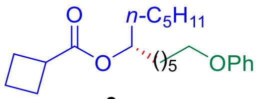

3s

Following **General procedure (G),** the reaction of **1x** (36.4 mg, 0.2 mmol) with **2i** (140.9 mg, 0.8 mmol) afforded **3s** as colorless oil (33.8 mg; 47% yield; 88% ee) by flash column chromatography on silica gel (eluent: PE:EA = 50:1).

^{1}H NMR (400 MHz, $CDCl_3$) δ 7.28 – 7.24 (m, 2H), 6.93 – 6.87 (m, 3H), 4.90 – 4.83 (m, 1H), 3.93 (t, J = 6.5 Hz, 2H), 3.15 – 3.06 (m, 1H), 2.31 – 2.14 (m, 4H), 2.01 – 1.84 (m, 2H), 1.79 – 1.72 (m, 2H), 1.55 – 1.41 (m, 6H), 1.39 – 1.21 (m, 10H), 0.97 – 0.79 (t, J = 6.4 Hz, 3H).

^{13}C NMR (101 MHz, $CDCl_3$) δ 175.5, 159.2, 129.5, 120.6, 114.6, 74.0, 67.9, 38.5, 34.3, 34.2, 31.8, 29.4, 29.3, 26.1, 25.40, 25.38, 25.35, 25.1, 22.7, 18.6, 14.1.

HRMS (ESI-TOF) Calcd for $C_{23}H_{36}O_3$ $(M+Na)^+$ 383.2557. Found 383.2550.

HPLC (OD-H, 0.46*25 cm, 5 µm, hexane/isopropanol = 98/2, flow rate = 1.0 mL/min, detection at 210 nm) retention time = 5.4 min (minor) and 6.5 min (major).

$[\alpha]^{25}D$ = −6.0 (c 1.0, CH_2Cl_2).

(*R*)-Decan-2-yl benzoate (4a)

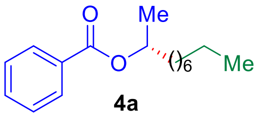

4a

Following **General procedure (G)**, the reaction of **1a** (29.6 mg, 0.2 mmol) with **2b** (89.7 mg, 0.8 mmol) afforded **4a** as colorless oil (36.7 mg; 70% yield; 91% ee) by flash column chromatography on silica gel (eluent: PE:EA = 100:1).

^{1}H NMR (400 MHz, $CDCl_3$) δ 8.05 – 8.02 (m, 2H), 7.57 – 7.52 (m, 1H), 7.45 – 7.41 (m, 2H), 5.20 – 5.11 (m, 1H), 1.77 – 1.69 (m, 1H), 1.60 – 1.56 (m, 1H), 1.41 – 1.25 (m, 15H), 0.87 (t, J = 6.4 Hz, 3H).

^{13}C NMR (101 MHz, $CDCl_3$) δ 166.5, 132.9, 131.2, 129.7, 128.5, 72.0, 36.3, 32.1, 29.73, 29.72, 29.5, 25.7, 22.9, 20.3, 14.3.

HRMS (ESI-TOF) Calcd for $C_{17}H_{26}O_2$ $(M+Na)^+$ 285.1825. Found 285.1823.

HPLC (AD-H, 0.46*25 cm, 5 µm, hexane/isopropanol = 99.5/0.5, flow rate = 0.5 mL/min, detection at 230 nm) retention time = 16.1 min (major) and 18.7 min (minor).

$[\alpha]^{25}D$ = −5.14 (c 0.7, CH_2Cl_2).

(*R*)-Tetradecan-2-yl benzoate (4b)

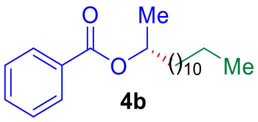

4b

Following **General procedure (F)**, the reaction of **1a** (29.6 mg, 0.2 mmol) with **2c** (134.6 mg, 0.8 mmol) afforded **4b** as colorless oil (41.4 mg; 65% yield; 95% ee) by flash column chromatography on silica gel (eluent: PE:EA = 100:1).

^{1}H NMR (400 MHz, $CDCl_3$) δ 8.06 – 8.03 (m, 2H), 7.57 – 7.52 (m, 1H), 7.46 – 7.41 (m, 2H), 5.20 – 5.12 (m, 1H), 1.79 – 1.70 (m, 1H), 1.65 – 1.56 (m, 1H), 1.41 – 1.25 (m, 23H), 0.88 (t, *J* = 6.7 Hz, 3H).
^{13}C NMR (101 MHz, $CDCl_3$) δ 166.4, 132.8, 131.1, 129.6, 128.4, 71.9, 36.2, 32.1, 29.81, 29.78, 29.77, 29.71, 29.67, 29.63, 29.5, 25.6, 22.8, 20.2, 14.3.
HRMS (ESI-TOF) Calcd for $C_{21}H_{34}O_2$ $(M+Na)^+$ 341.2451. Found 341.2450.
HPLC (AD-H, 0.46*25 cm, 5 μm, hexane/isopropanol = 99.8/0.2, flow rate = 1 mL/min, detection at 230 nm) retention time = 7.6 min (major) and 9.2 min (minor).
$[\alpha]^{25}D$ = −32.10 (c 1.0, CH_2Cl_2).

(*R*)-6-Phenylhexan-2-yl benzoate (4c)

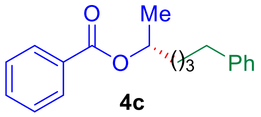

4c

Following **General procedure (F)**, the reaction of **1a** (29.6 mg, 0.2 mmol) with **2d** (105.7 mg, 0.8 mmol) afforded **4c** as colorless oil (36.1 mg; 64% yield; 93% ee) by flash column chromatography on silica gel (eluent: PE:EA = 100:1).
^{1}H NMR (400 MHz, $CDCl_3$) δ 8.04 – 8.01 (m, 2H), 7.56 – 7.51 (m, 1H), 7.44 – 7.40 (m, 2H), 7.27 – 7.22 (m, 2H), 7.17 – 7.13 (m, 3H), 5.18 – 5.11 (m, 1H), 2.60 (t, *J* = 7.7 Hz, 2H), 1.80 – 1.72 (m, 1H), 1.69 – 1.65 (m, 3H), 1.51 – 1.38 (m, 2H), 1.32 (d, *J* = 6.2 Hz, 3H).
^{13}C NMR (101 MHz, $CDCl_3$) δ 166.3, 142.6, 132.8, 131.0, 129.6, 128.5, 128.41, 128.38, 125.8, 71.7, 36.0, 35.9, 31.4, 25.2, 20.2.
HRMS (ESI-TOF) Calcd for $C_{19}H_{22}O_2$ $(M+Na)^+$ 305.1512. Found 305.1510.
HPLC (OD-H, 0.46*25 cm, 5 μm, hexane/isopropanol = 99/1, flow rate = 1.0 mL/min, detection at 230 nm) retention time = 7.4 min (major) and 9.5 min (minor).
$[\alpha]^{25}D$ = −24.70 (c 1.0, CH_2Cl_2).

(*R*)-6-Methylheptan-2-yl benzoate (4d)

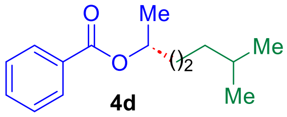

4d

Following **General procedure (F)**, the reaction of **1a** (29.6 mg, 0.2 mmol) with **2e** (67.3 mg, 0.8 mmol) afforded **4d** as colorless oil (27.2 mg; 58% yield; 89% ee) by flash column chromatography on silica gel (eluent: PE:EA = 100:1).
^{1}H NMR (400 MHz, $CDCl_3$) δ 8.07 – 8.02 (m, 2H), 7.57 – 7.52 (m, 1H), 7.45 – 7.41 (m, 2H), 5.20 – 5.12 (m, 1H), 1.78 – 1.68 (m, 1H), 1.63 – 1.48 (m, 2H), 1.45 – 1.30 (m, 5H), 1.27 – 1.17 (m, 2H), 0.86 (d, *J* = 6.6Hz, 6H).
^{13}C NMR (101 MHz, $CDCl_3$) δ 166.3, 132.7, 130.9, 129.5, 128.3, 71.8, 38.8, 36.3, 27.8, 23.2, 22.6, 22.5, 20.1.
HRMS (ESI-TOF) Calcd for $C_{15}H_{22}O_2$ $(M+Na)^+$ 257.1512. Found 257.1510.
HPLC (OJ-H, 0.46*25 cm, 5 μm, hexane/isopropanol = 99.5/0.5, flow rate = 0.5 mL/min, detection at 254 nm) retention time = 9.3 min (major) and 11.8 min (minor).

$[\alpha]^{25}D = -27.09$ (c 1.0, CH_2Cl_2).

(*R*)-4-Cyclohexylbutan-2-yl benzoate (4e)

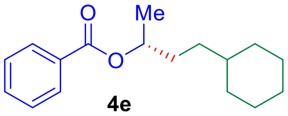

4e

Following **General procedure (F)**, the reaction of **1a** (29.6 mg, 0.2 mmol) with **2f** (88.1 mg, 0.8 mmol) afforded **4e** as colorless oil (27.1 mg; 52% yield; 90% ee) by flash column chromatography on silica gel (eluent: PE:EA = 100:1).
^{1}H NMR (400 MHz, $CDCl_3$) δ 8.06 – 8.02 (m, 2H), 7.57 – 7.52 (m, 1H), 7.45 – 7.41 (m, 2H), 5.17 – 5.08 (m, 1H), 1.76 – 1.59 (m, 7H), 1.33 (d, *J* = 6.3 Hz, 3H), 1.30 – 1.11 (m, 6H), 0.93 – 0.83 (m, 2H).
^{13}C NMR (101 MHz, $CDCl_3$) δ 166.4, 132.8, 131.1, 129.6, 128.4, 72.2, 37.7, 33.53, 33.45, 33.2, 26.8, 26.5, 20.2.
HRMS (ESI-TOF) Calcd for $C_{17}H_{24}O_2$ $(M+Na)^+$ 283.1669. Found 283.1668.
HPLC (IA, 0.46*25 cm, 5 µm, hexane/isopropanol = 99.8/0.2, flow rate = 1.0 mL/min, detection at 210 nm) retention time = 7.3 min (major) and 8.6 min (minor).
$[\alpha]^{25}D = -14.20$ (c 1.0, CH_2Cl_2).

(*R*)-5-Cyclohexylpentan-2-yl benzoate (4f)

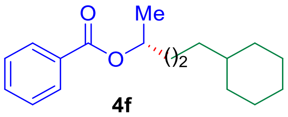

4f

Following **General procedure (F)**, the reaction of **1a** (29.6 mg, 0.2 mmol) with **2g** (99.3 mg, 0.8 mmol) afforded **4f** as colorless oil (29.1 mg; 53% yield; 85% ee) by flash column chromatography on silica gel (eluent: PE:EA = 100:1).
^{1}H NMR (400 MHz, $CDCl_3$) δ 8.06 – 8.03 (m, 2H), 7.57 – 7.52 (m, 1H), 7.45 – 7.42 (m, 2H), 5.19 – 5.11 (m, 1H), 1.77 – 1.53 (m, 7H), 1.44 – 1.32 (m, 2H), 1.33 (d, *J* = 6.2 Hz, 3H), 1.25 – 1.09 (m, 6H), 0.89 – 0.80 (m, 2H).
^{13}C NMR (101 MHz, $CDCl_3$) δ 166.4, 132.8, 131.1, 129.6, 128.4, 71.9, 37.6, 37.4, 36.5, 33.5, 26.8, 26.5, 22.8, 20.2.
HRMS (ESI-TOF) Calcd for $C_{18}H_{26}O_2$ $(M+Na)^+$ 297.1825. Found 297.1824.
HPLC (AD-H, 0.46*25 cm, 5 µm, hexane/isopropanol = 99.2/0.8, flow rate = 1.0 mL/min, detection at 230 nm) retention time = 5.3 min (major) and 5.6 min (minor).
$[\alpha]^{25}D = -27.70$ (c 1.0, CH_2Cl_2).

(*R*)-8-Chlorooctan-2-yl benzoate (4g)

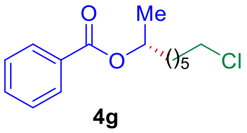

4g

Following **General procedure (F)**, the reaction of **1a** (29.6 mg, 0.2 mmol) with **2h** (94.4 mg, 0.8 mmol) afforded **4g** as colorless oil (34.3 mg; 64% yield; 90% ee) by flash column chromatography on silica gel (eluent: PE:EA = 100:1).

^{1}H NMR (400 MHz, $CDCl_3$) δ 7.98 – 7.96 (m, 2H), 7.50 – 7.46 (m, 1H), 7.38 – 7.35 (m, 2H), 5.13 – 5.05 (m, 1H), 3.45 (t, *J* = 6.7 Hz, 2H), 1.72 – 1.63 (m, 3H), 1.59 – 1.50 (m, 1H), 1.40 – 1.26 (m, 9H).

^{13}C NMR (101 MHz, $CDCl_3$) δ 166.4, 132.9, 131.0, 129.6, 128.4, 71.7, 45.2, 36.1, 32.7, 28.9, 26.9, 25.4, 20.2.

HRMS (ESI-TOF) Calcd for $C_{15}H_{21}ClO_2$ $(M+Na)^+$ 291.1122. Found 291.1121.

HPLC (AD-H, 0.46*25 cm, 5 μm, hexane/isopropanol = 99/1, flow rate = 0.8 mL/min, detection at 230 nm) retention time = 7.2 min (major) and 8.3 min (minor).

$[\alpha]^{25}D$ = −25.45 (c 0.6, CH_2Cl_2).

(*R*)-8-Phenoxyoctan-2-yl benzoate (4h)

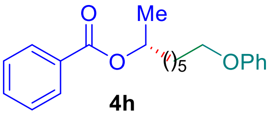

Following **General procedure (F)**, the reaction of **1a** (29.6 mg, 0.2 mmol) with **2i** (140.9 mg, 0.8 mmol) afforded **4h** as colorless oil (45.7 mg; 70% yield; 88% ee) by flash column chromatography on silica gel (eluent: PE:EA = 50:1).

^{1}H NMR (600 MHz, $CDCl_3$) δ 8.05 – 8.03 (m, 2H), 7.55 – 7.52 (m, 1H), 7.42 (t, *J* = 7.7 Hz, 2H), 7.28 – 7.24 (m, 2H), 6.93 – 6.87 (m, 3H), 5.19 – 5.14 (m, 1H), 3.93 (t, *J* = 6.5 Hz, 2H), 1.79 – 1.72 (m, 3H), 1.65 – 1.60 (m, 1H), 1.49 – 1.38 (m, 6H), 1.34 (d, *J* = 6.2 Hz, 3H).

^{13}C NMR (151 MHz, $CDCl_3$) δ 166.3, 159.2, 132.8, 131.0, 129.6, 129.5, 128.4, 120.6, 114.6, 71.8, 67.9, 36.1, 29.4, 29.3, 26.1, 25.5, 20.2.

HRMS (ESI-TOF) Calcd for $C_{21}H_{26}O_3$ $(M+Na)^+$ 349.1774. Found 349.1770.

HPLC (IA, 0.46*25 cm, 5 μm, hexane/isopropanol = 99/1, flow rate = 1.0 mL/min, detection at 254 nm) retention time = 7.5 min (major) and 7.9 min (minor).

$[\alpha]^{25}D$ = −16.20 (c 1.0, CH_2Cl_2).

(*R*)-8-(4-Cyanophenoxy)octan-2-yl benzoate (4i)

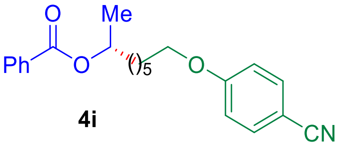

Following **General procedure (F)**, the reaction of **1a** (29.6 mg, 0.2 mmol) with **2j** (160.9 mg, 0.8 mmol) afforded **4i** as colorless oil (48.5 mg; 69% yield; 80% ee) by flash column chromatography on silica gel (eluent: PE:EA = 30:1).

^{1}H NMR (400 MHz, $CDCl_3$) δ 8.05 – 8.02 (m, 2H), 7.57 – 7.52 (m, 3H), 7.45 – 7.41 (m, 2H), 6.93 – 6.89 (m, 2H), 5.21 – 5.13 (m, 1H), 3.97 (t, *J* = 6.5 Hz, 2H), 1.82 – 1.72 (m, 3H), 1.69 – 1.58 (m, 1H), 1.48 – 1.38 (m, 6H), 1.34 (d, *J* = 6.3 Hz, 3H).

^{13}**C NMR** (101 MHz, $CDCl_3$) δ 166.3, 162.5, 134.1, 132.9, 130.9, 129.6, 128.4, 119.4, 115.3, 103.8, 71.6, 68.4, 36.1, 29.2, 29.0, 26.0, 25.4, 20.2.
HRMS (ESI-TOF) Calcd for $C_{22}H_{25}NO_3$ $(M+Na)^+$ 374.1727. Found : 374.1722.
HPLC (IA, 0.46*25 cm, 5 μm, hexane/isopropanol = 98/2, flow rate = 1.0 mL/min, detection at 230 nm) retention time = 15.4 min (major) and 16.1 min (minor).
$[\alpha]^{25}D = -19.50$ (c 1.0, CH_2Cl_2).

(*R*)-8-((*tert*-Butyldimethylsilyl)oxy)octan-2-yl benzoate (4j)

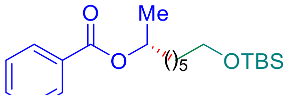

4j

Following **General procedure (F)**, the reaction of **1a** (29.6 mg, 0.2 mmol) with **2k** (171.3 mg, 0.8 mmol) afforded **4j** as colorless oil (35.7 mg; 49% yield; 93% ee) by flash column chromatography on silica gel (eluent: PE:EA = 40:1).
^{1}**H NMR** (400 MHz, $CDCl_3$) δ 8.06 – 8.03 (m, 2H), 7.56 – 7.52 (m, 1H), 7.45 – 7.41 (m, 2H), 5.19 – 5.11 (m, 1H), 3.59 (t, J = 6.6 Hz, 2H), 1.77 – 1.70 (m, 1H), 1.65 – 1.56 (m, 1H), 1.52 – 1.47 (m, 2H), 1.43 – 1.32 (m, 9H), 0.89 (s, 9H), 0.04 (s, 6H).
^{13}**C NMR** (101 MHz, $CDCl_3$) δ 166.3, 132.8, 131.0, 129.6, 128.4, 71.8, 63.3, 36.1, 32.9, 29.4, 26.1, 25.8, 25.6, 20.2, 18.5, -5.1.
HRMS (ESI-TOF) Calcd for $C_{21}H_{36}O_3Si$ $(M+Na)^+$ 387.2326. Found 387.2322.
HPLC (OD-H, 0.46*25 cm, 5 μm, hexane = 100, flow rate = 1.0 mL/min, detection at 254 nm) retention time = 11.8 min (major) and 15.2 min (minor).
$[\alpha]^{25}D = -19.60$ (c 1.0, CH_2Cl_2).

(*R*)-8-Phenoxyoctan-2-yl benzoate (4k)

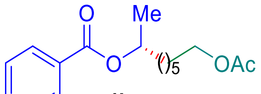

4k

Following **General procedure (F)**, the reaction of **1a** (29.6 mg, 0.2 mmol) with **2l** (113.7 mg, 0.8 mmol) afforded **4k** as colorless oil (42.1 mg; 72% yield; 90% ee) by flash column chromatography on silica gel (eluent: PE:EA = 30:1).
^{1}**H NMR** (400 MHz, $CDCl_3$) δ 8.05 – 8.02 (m, 2H), 7.56 – 7.51 (m, 1H), 7.45 – 7.40 (m, 2H), 5.19 – 5.11 (m, 1H), 4.03 (t, J = 6.7 Hz, 2H), 2.02 (s, 3H), 1.80 – 1.69 (m, 1H), 1.64 – 1.56 (m, 3H), 1.46 – 1.24 (m, 9H).
^{13}**C NMR** (101 MHz, $CDCl_3$) δ 171.3, 166.3, 132.8, 130.9, 129.6, 128.4, 71.7, 64.6, 36.0, 29.2, 28.6, 25.9, 25.4, 21.1, 20.2.
HRMS (ESI-TOF) Calcd for $C_{17}H_{24}O_4$ $(M+Na)^+$ 315.1567. Found 315.1564.
HPLC (IA, 0.46*25 cm, 5 μm, hexane/isopropanol = 99/1, flow rate = 1.0 mL/min, detection at 230 nm) retention time = 8.3 min (major) and 9.5 min (minor).
$[\alpha]^{25}D = -22.40$ (c 1.0, CH_2Cl_2).

Dimethyl (*R*)-2-(7-(benzoyloxy)octyl)malonate (4l)

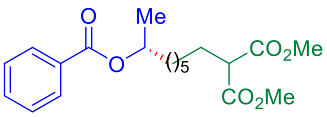

4l

Following **General procedure (F)**, the reaction of **1a** (29.6 mg, 0.2 mmol) with **2m** (171.3 mg, 0.8 mmol) afforded **4l** as colorless oil (48.8 mg; 67% yield; 92% ee) by flash column chromatography on silica gel (eluent: PE:EA = 30:1).

^{1}H NMR (400 MHz, $CDCl_3$) δ 8.03 – 8.01 (m, 2H), 7.55 – 7.51 (m, 1H), 7.44 – 7.40 (m, 2H), 5.17 – 5.09 (m, 1H), 3.71 (s, 6H), 3.33 (t, J = 7.5 Hz, 1H), 1.90 – 1.84 (m, 2H), 1.75 – 1.67 (m, 1H), 1.62 – 1.54 (m, 1H), 1.39 – 1.24 (m, 11H).

^{13}C NMR (101 MHz, $CDCl_3$) δ 170.1, 166.4, 132.9, 131.0, 129.7, 128.5, 71.8, 52.6, 51.8, 36.2, 29.3, 29.2, 29.0, 27.4, 25.5, 20.3.

HRMS (ESI-TOF) Calcd for $C_{20}H_{28}O_6$ $(M+Na)^+$ 387.1778. Found 387.1774.

HPLC (AD-H, 0.46*25 cm, 5 µm, hexane/isopropanol = 98/2, flow rate = 1.0 mL/min, detection at 230 nm) retention time = 10.6 min (major) and 11.5 min (minor).

$[\alpha]^{25}D$ = −21.50 (c 1.0, CH_2Cl_2).

(*S*)-5-Oxohexan-2-yl benzoate ((*S*)-4m)

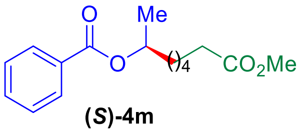

(*S*)-4m

Following **General procedure (F)**, the reaction of **1a** (29.6 mg, 0.2 mmol) with **2n** (102.5 mg, 0.8 mmol) afforded **(*S*)-4m** as colorless oil (33.9 mg; 61% yield; 90% ee) by flash column chromatography on silica gel (eluent: PE:EA = 20:1).

^{1}H NMR (400 MHz, $CDCl_3$) δ 8.05 – 8.02 (m, 2H), 7.57 – 7.52 (m, 1H), 7.45 – 7.41 (m, 2H), 5.19 – 5.11 (m, 1H), 3.65 (s, 3H), 2.30 (t, J = 7.5 Hz, 2H), 1.77 – 1.70 (m, 1H), 1.67 – 1.57 (m, 3H), 1.44 – 1.35 (m, 4H), 1.33 (d, J = 6.2 Hz, 3H).

^{13}C NMR (101 MHz, $CDCl_3$) δ 174.3, 166.3, 132.9, 131.0, 129.6, 128.4, 71.7, 51.6, 36.0, 34.1, 29.1, 25.2, 24.9, 20.2.

HRMS (ESI-TOF) Calcd for $C_{16}H_{23}O_4$ $(M+H)^+$ 279.1591. Found 279.1592.

HPLC (AD-H, 0.46*25 cm, 5 µm, hexane/isopropanol = 99/1, flow rate = 1.0 mL/min, detection at 210 nm) retention time = 11.2 min (minor) and 12.7 min (major).

$[\alpha]^{25}D$ = +23.0 (c 1.0, CH_2Cl_2).

(*R*)-8-(1,3-Dioxoisoindolin-2-yl)octan-2-yl benzoate (4n)

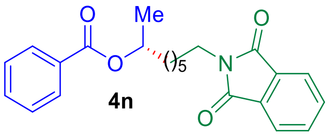

4n

Following **General procedure (F)**, the reaction of **1a** (29.6 mg, 0.2 mmol) with **2o** (183.3 mg, 0.8 mmol) afforded **4n** as colorless oil (52.3 mg; 69% yield; 92% ee) by flash column chromatography on silica gel (eluent: PE:EA = 20:1).

^{1}H NMR (600 MHz, $CDCl_3$) δ 8.03 – 8.01 (m, 2H), 7.83 – 7.80 (m, 2H), 7.70 – 7.67 (m, 2H), 7.53 – 7.51 (m, 1H), 7.43 – 7.40 (m, 2H), 5.15 – 5.10 (m, 1H), 3.66 (t, *J* = 7.3 Hz, 2H), 1.75 – 1.70 (m, 1H), 1.69 – 1.63 (m, 2H), 1.62 – 1.56 (m, 1H), 1.42 – 1.31 (m, 9H).
^{13}C NMR (151 MHz, $CDCl_3$) δ 168.5, 166.3, 133.9, 132.8, 132.3, 131.0, 129.6, 128.4, 123.2, 71.7, 38.1, 36.0, 29.1, 28.6, 26.8, 25.4, 20.1.
HRMS (ESI-TOF) Calcd for $C_{23}H_{25}NO_4$ $(M+Na)^+$ 402.1676. Found 402.1673.
HPLC (AD-H, 0.46*25 cm, 5 μm, hexane/isopropanol = 98/2, flow rate = 1.0 mL/min, detection at 230 nm) retention time = 20.8 min (major) and 23.3 min (minor).
$[\alpha]^{25}D = -12.20$ (c 1.0, CH_2Cl_2).

(*R*)-8-(9*H*-Carbazol-9-yl)octan-2-yl benzoate (4o)

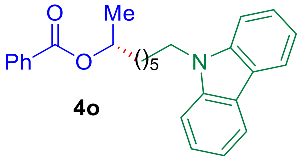

4o

Following **General procedure (F)**, the reaction of **1a** (29.6 mg, 0.2 mmol) with **2p** (199.3 mg, 0.8 mmol) afforded **4o** as colorless oil (50.3 mg; 63% yield; 94% ee) by flash column chromatography on silica gel (eluent: PE:EA = 20:1).
^{1}H NMR (600 MHz, $CDCl_3$) δ 8.13 – 8.11 (m, 2H), 8.06 – 8.04 (m, 2H), 7.57 – 7.54 (m, 1H), 7.48 – 7.43 (m, 4H), 7.41 – 7.39 (m, 2H), 7.26 – 7.23 (m, 2H), 5.18 – 5.13 (m, 1H), 4.30 (t, *J* = 7.2 Hz, 2H), 1.91 – 1.86 (m, 2H), 1.76 – 1.70 (m, 1H), 1.60 – 1.56 (m, 1H), 1.43 – 1.36 (m, 6H), 1.33 (d, *J* = 6.2 Hz, 3H).
^{13}C NMR (151 MHz, $CDCl_3$) δ 166.3, 140.5, 132.8, 131.0, 129.6, 128.43, 128.42, 125.7, 122.9, 120.4, 118.8, 71.7, 43.1, 36.1, 29.4, 29.0, 27.3, 25.4, 20.2.
HRMS (ESI-TOF) Calcd for $C_{27}H_{29}NO_2$ $(M+Na)^+$ 422.2091. Found 422.2089.
HPLC (AD-H, 0.46*25 cm, 5 μm, hexane/isopropanol = 98/2, flow rate = 1.0 mL/min, detection at 254 nm) retention time = 10.0 min (major) and 11.5 min (minor).
$[\alpha]^{25}D = -16.70$ (c 1.0, CH_2Cl_2).

(*R*)-8-(1*H*-Indol-1-yl)octan-2-yl benzoate (4p)

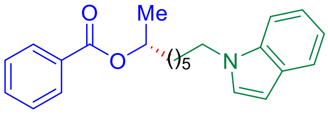

4p

Following **General procedure (F)**, the reaction of **1a** (29.6 mg, 0.2 mmol) with **2q** (159.3 mg, 0.8 mmol) afforded **4p** as colorless oil (48.2 mg; 69% yield; 93% ee) by flash column chromatography on silica gel (eluent: PE:EA = 30:1).
^{1}H NMR (400 MHz, $CDCl_3$) δ 8.07 – 8.04 (m, 2H), 7.66 – 7.63 (m, 1H), 7.59 – 7.54 (m, 1H), 7.47 – 7.43 (m, 2H), 7.35 (dd, *J* = 8.2, 1.1 Hz, 1H), 7.23 – 7.19 (m, 1H), 7.13 – 7.09 (m, 2H), 6.49 (dd, *J* = 3.1, 0.8 Hz, 1H), 5.20 – 5.12 (m, 1H), 4.11 (t, *J* = 7.1 Hz, 2H), 1.88 – 1.81 (m, 2H), 1.76 – 1.69 (m, 1H), 1.64 – 1.57 (m, 1H), 1.42 – 1.31 (m, 9H).

^{13}C NMR (101 MHz, $CDCl_3$) δ 166.3, 136.0, 132.9, 131.0, 129.6, 128.7, 128.4, 127.9, 121.4, 121.0, 119.3, 109.5, 101.0, 71.7, 46.4, 36.1, 30.3, 29.2, 27.0, 25.4, 20.2.
HRMS (ESI-TOF) Calcd for $C_{23}H_{27}NO_2$ $(M+Na)^+$ 372.1934. Found 372.1931.
HPLC (AD-H, 0.46*25 cm, 5 μm, hexane/isopropanol = 99/1, flow rate = 0.8 mL/min, detection at 254 nm) retention time = 16.4 min (major) and 22.9 min (minor).
$[\alpha]^{25}D = -19.20$ (c 1.0, CH_2Cl_2).

(*R*)-7-(1*H*-Pyrrol-1-yl)heptan-2-yl benzoate (4q)

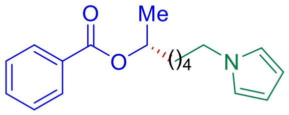

4q

Following **General procedure (F)**, the reaction of **1a** (29.6 mg, 0.2 mmol) with **2r** (108.1 mg, 0.8 mmol) afforded **4q** as colorless oil (31.9 mg; 56% yield; 90% ee) by flash column chromatography on silica gel (eluent: PE:EA = 30:1).
^{1}H NMR (400 MHz, $CDCl_3$) δ 8.06 – 8.03 (m, 2H), 7.58 – 7.54 (m, 1H), 7.47 – 7.43 (m, 2H), 6.64 (t, J = 2.1 Hz, 2H), 6.13 (t, J = 2.1 Hz, 2H), 5.19 – 5.12 (m, 1H), 3.86 (t, J = 7.1 Hz, 2H), 1.81 – 1.70 (m, 3H), 1.65 – 1.58 (m, 1H), 1.48 – 1.27 (m, 7H).
^{13}C NMR (101 MHz, $CDCl_3$) δ 166.4, 133.0, 131.0, 129.7, 128.5, 120.7, 108.0, 71.7, 49.7, 36.1, 31.7, 26.9, 25.3, 20.3.
HRMS (ESI-TOF) Calcd for $C_{18}H_{23}NO_2$ $(M+Na)^+$ 308.1621. Found 308.1618.
HPLC (AD-H, 0.46*25 cm, 5 μm, hexane/isopropanol = 99/1, flow rate = 1.0 mL/min, detection at 210 nm) retention time = 8.8 min (major) and 10.6 min (minor).
$[\alpha]^{25}D = -22.93$ (c 0.8, CH_2Cl_2).

(*R*)-8-(5-Methylfuran-2-yl)octan-2-yl benzoate (4r)

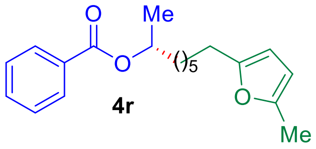

4r

Following **General procedure (F)**, the reaction of **1a** (29.6 mg, 0.2 mmol) with **2s** (131.3 mg, 0.8 mmol) afforded **4r** as colorless oil (28.3 mg; 45% yield; 90% ee) by flash column chromatography on silica gel (eluent: PE:EA = 30:1).
^{1}H NMR (400 MHz, $CDCl_3$) δ 8.06 – 8.03 (m, 2H), 7.57 – 7.53 (m, 1H), 7.46 – 7.42 (m, 2H), 5.84 – 5.82 (m, 2H), 5.20 – 5.12 (m, 1H), 2.55 (t, J = 7.6 Hz, 2H), 2.24 (s, 3H), 1.77 – 1.70 (m, 1H), 1.62 – 1.57 (m, 3H), 1.43 – 1.33 (m, 9H).
^{13}C NMR (101 MHz, $CDCl_3$) δ 166.5, 154.9, 150.3, 132.9, 131.1, 129.7, 128.5, 105.9, 105.4, 71.9, 36.2, 29.4, 29.3, 28.3, 28.2, 25.6, 20.3, 13.7.
HRMS (ESI-TOF) Calcd for $C_{20}H_{26}O_3$ $(M+Na)^+$ 337.1774. Found 337.1772.
HPLC (AD-H, 0.46*25 cm, 5 μm, hexane/isopropanol = 99.2/0.8, flow rate = 0.5 mL/min, detection at 210 nm) retention time = 10.1 min (major) and 10.6 min (minor).
$[\alpha]^{25}D = -25.80$ (c 1.0, CH_2Cl_2).

(*R*)-8-(2-(1-(4-Chlorobenzoyl)-5-methoxy-3-methyl-1*H*-indol-2-yl)acetoxy)octan-2-yl benzoate (4s)

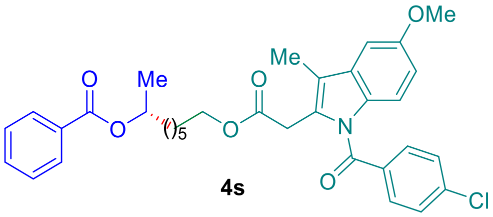

Following **General procedure (F)**, the reaction of **1a** (29.6 mg, 0.2 mmol) with **2t** (351.3 mg, 0.8 mmol) afforded **4s** as colorless oil (67.2 mg; 57% yield; 92% ee) by flash column chromatography on silica gel (eluent: PE:EA = 10:1).

^{1}H NMR (400 MHz, $CDCl_3$) δ 8.05 – 8.02 (m, 2H), 7.68 – 7.64 (m, 2H), 7.57 – 7.52 (m, 1H), 7.49 – 7.41 (m, 4H), 6.96 (d, *J* = 2.6 Hz, 1H), 6.86 (d, *J* = 9.0 Hz, 1H), 6.65 (dd, *J* = 9.0, 2.5 Hz, 1H), 5.18 – 5.10 (m, 1H), 4.08 (t, *J* = 6.7 Hz, 2H), 3.82 (s, 3H), 3.65 (s, 2H), 2.38 (s, 3H), 1.73 – 1.67 (m, 1H), 1.62 – 1.55 (m, 3H), 1.38 – 1.25 (m, 9H).

^{13}C NMR (101 MHz, $CDCl_3$) δ 171.1, 168.4, 166.3, 156.1, 139.4, 136.0, 134.0, 132.9, 131.3, 131.0, 130.9, 130.8, 129.6, 129.2, 128.4, 115.1, 112.8, 111.7, 101.4, 71.7, 65.2, 55.8, 36.1, 30.5, 29.2, 28.7, 25.9, 25.4, 20.2, 13.5.

HRMS (ESI-TOF) Calcd for $C_{34}H_{36}ClNO_6$ $(M+Na)^+$ 612.2123. Found 612.2117.

HPLC (IA, 0.46*25 cm, 5 µm, hexane/isopropanol = 90/10, flow rate = 1.0 mL/min, detection at 230 nm) retention time = 15.9 min (major) and 18.4 min (minor).

$[\alpha]^{25}D$ = −6.8 (c 1.0, CH_2Cl_2).

(*R*)-8-(((8*S*,9*R*,13*R*,14*R*)-8,13-Dimethyl-17-oxo-7,8,9,11,12,13,14,15,16,17-decahydro-6*H*-cyclopenta[a]phenanthren-2-yl)oxy)octan-2-yl benzoate (4t)

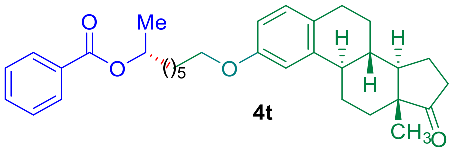

Following **General procedure (F)**, the reaction of **1a** (29.6 mg, 0.2 mmol) with **2u** (281.8 mg, 0.8 mmol) afforded **3a** as colorless oil (62.3 mg; 62% yield; dr = 96:4) by flash column chromatography on silica gel (eluent: PE:EA = 5:1).

^{1}H NMR (400 MHz, $CDCl_3$) δ 8.07 – 8.04 (m, 2H), 7.57 – 7.53 (m, 1H), 7.46 – 7.42 (m, 2H), 7.19 (d, *J* = 8.6 Hz, 1H), 6.71 (dd, *J* = 8.5, 2.8 Hz, 1H), 6.64 (d, *J* = 2.7 Hz, 1H), 5.21 – 5.13 (m, 1H), 3.92 (t, *J* = 6.5 Hz, 2H), 2.97 – 2.91 (m, 2H), 2.50 (dd, *J* = 18.9, 8.7 Hz, 1H), 2.42 – 2.37 (m, 1H), 2.28 – 2.21 (m, 1H), 2.19 – 1.93 (m, 4H), 1.84 – 1.72 (m, 3H), 1.67 – 1.57 (m, 3H), 1.55 – 1.53 (m, 1H), 1.50 – 1.38 (m, 9H), 1.35 (d, *J* = 6.2 Hz, 3H), 0.91 (s, 3H).

^{13}C NMR (101 MHz, $CDCl_3$) δ 221.1, 166.3, 157.2, 137.7, 132.8, 131.9, 130.9, 129.6, 128.37, 128.36, 126.4, 114.6, 112.2, 71.7, 67.8, 50.5, 48.1, 44.0, 38.4, 36.1, 35.9, 31.6, 29.7, 29.31, 29.29, 26.6, 26.1, 26.0, 25.5, 21.6, 20.2, 13.9.

HRMS (ESI-TOF) Calcd for $C_{33}H_{42}O_4$ $(M+Na)^+$ 525.2975. Found 525.2976.
HPLC (AD-H, 0.46*25 cm, 5 μm, hexane/isopropanol = 99/1, flow rate = 1.0 mL/min, detection at 254 nm) retention time = 64.2 min (major) and 73.6 min (minor).
$[\alpha]^{25}D$ = +54.70 (c 1.0, CH_2Cl_2).

(*R*)-12-(1H-Indol-1-yl)dodecan-6-yl benzoate (4u)

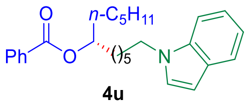

4u

Following **General procedure (G),** the reaction of **1q** (40.8 mg, 0.2 mmol) with **2q** (159.3 mg, 0.8 mmol) afforded **4u** as colorless oil (51.9 mg; 64% yield; 94% ee) by flash column chromatography on silica gel (eluent: PE:EA = 20:1).
^{1}H NMR (400 MHz, $CDCl_3$) δ 8.10 – 8.07 (m, 2H), 7.66 – 7.64 (m, 1H), 7.60 – 7.56 (m, 1H), 7.48 – 7.45 (m, 2H), 7.36 – 7.34 (m, 1H), 7.24 – 7.20 (m, 1H), 7.14 – 7.09 (m, 2H), 6.50 (d, *J* = 3.1 Hz, 1H), 5.19 – 5.13 (m, 1H), 4.11 (t, *J* = 7.1 Hz, 2H), 1.87 – 1.80 (m, 2H), 1.74 – 1.60 (m, 4H), 1.42 – 1.31 (m, 12H), 0.92 (t, *J* = 6.8 Hz, 3H).
^{13}C NMR (101 MHz, $CDCl_3$) δ 166.5, 136.0, 132.8, 130.9, 129.7, 128.7, 128.4, 127.9, 121.4, 121.0, 119.3, 109.5, 101.0, 75.0, 46.4, 34.3, 34.2, 31.9, 30.2, 29.3, 27.0, 25.3, 25.1, 22.7, 14.1.
HRMS (ESI-TOF) Calcd for $C_{27}H_{35}NO_2$ $(M+Na)^+$ 428.2560. Found 428.2556.
HPLC (IC, 0.46*25 cm, 5 μm, hexane/isopropanol = 95/5, flow rate = 1.0 mL/min, detection at 254 nm) retention time = 5.8 min (minor) and 6.3 min (major).
$[\alpha]^{25}D$ = −7.0 (c 1.0, CH_2Cl_2).

(*R*)-9-((*tert*-Butyldimethylsilyl)oxy)nonan-3-yl benzoate (4v)

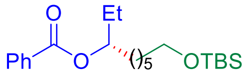

4v

Following **General procedure (G),** the reaction of **1o** (32.4 mg, 0.2 mmol) with **2k** (171.3 mg, 0.8 mmol) afforded **4v** as colorless oil (51.1 mg; 63% yield; 94% ee) by flash column chromatography on silica gel (eluent: PE:EA = 20:1).
^{1}H NMR (400 MHz, $CDCl_3$) δ 8.07 – 8.04 (m, 2H), 7.57 – 7.53 (m, 1H), 7.46 – 7.42 (m, 2H), 5.11 – 5.05 (m, 1H), 3.58 (t, *J* = 6.6 Hz, 2H), 1.74 – 1.64 (m, 4H), 1.53 – 1.46 (m, 2H), 1.39 – 1.29 (m, 6H), 0.95 (t, *J* = 7.4 Hz, 3H), 0.88 (s, 9H), 0.03 (s, 6H).
^{13}C NMR (101 MHz, $CDCl_3$) δ 166.5, 132.8, 131.0, 129.7, 128.4, 76.3, 63.4, 33.8, 32.9, 29.5, 27.2, 26.1, 25.8, 25.5, 18.5, 9.8, -5.1.
HRMS (ESI-TOF) Calcd for $C_{22}H_{38}O_3Si$ $(M+Na)^+$ 401.2482. Found 401.2480.
HPLC (OD-H, 0.46*25 cm, hexane/isopropanol = 99.2/0.8, flow rate = 1.0 mL/min, detection at 230 nm) retention time = 10.2 min (major) and 12.7 min (minor).
$[\alpha]^{25}D$ = −20.6 (c 1.0, CH_2Cl_2).

(*R*)-1-Cyclopentylethyl benzoate (4w)

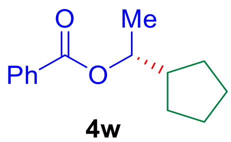

4w

Following **General procedure (G),** the reaction of **1a** (29.6 mg, 0.2 mmol) with **2y** (54.5 mg, 0.8 mmol) using **L2** (16.3 mg, 0.024 mmol) instead of **L1** afforded **4w** as colorless oil (11.3 mg; 68% conversion of **1a**; 38% yield (based on recovery of **1a**); 70% ee) by flash column chromatography on silica gel (eluent: PE:EA = 100:1),
^{1}H NMR (600 MHz, $CDCl_3$) δ 8.05 – 8.03 (m, 2H), 7.56 – 7.53 (m, 1H), 7.45 – 7.42 (m, 2H), 5.06 – 5.02 (m, 1H), 2.18 – 2.11 (m, 1H), 1.86 – 1.73 (m, 2H), 1.68 – 1.52 (m, 4H), 1.44 – 1.38 (m, 1H), 1.34 (d, J = 6.3, 3H), 1.31 – 1.26 (m, 1H).
^{13}C NMR (151 MHz, $CDCl_3$) δ 166.4, 132.8, 131.1, 129.6, 128.4, 75.3, 45.5, 29.1, 29.0, 25.8, 25.6, 19.3.
HRMS (ESI-TOF) Calcd for $C_{14}H_{18}O_2$ $(M+H)^+$ 219.1380. Found 219.1380.
HPLC (OD-H, 0.46*25 cm, hexane/isopropanol = 99.2/0.8, flow rate = 1.0 mL/min, detection at 230 nm) retention time = 11.1 min (major) and 12.1 min (minor).
$[\alpha]^{25}D$ = −18.4 (c 1.0, CH_2Cl_2).

(*R*)-Decan-2-yl benzoate (5a)

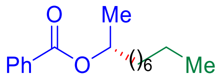

5a

Following **General procedure (H)**, the reaction of **1a** (29.6 mg, 0.2 mmol) with **2v** (89.7 mg, 0.8 mmol) afforded **5a** as colorless oil (30.9 mg; 59% yield; 89% ee) by flash column chromatography on silica gel (eluent: PE:EA = 100:1).
^{1}H NMR (400 MHz, $CDCl_3$) δ 8.05 – 8.02 (m, 2H), 7.57 – 7.52 (m, 1H), 7.45 – 7.41 (m, 2H), 5.20 – 5.11 (m, 1H), 1.77 – 1.69 (m, 1H), 1.60 – 1.56 (m, 1H), 1.41 – 1.25 (m, 15H), 0.87 (t, J = 6.4 Hz, 3H).
^{13}C NMR (101 MHz, $CDCl_3$) δ 166.5, 132.9, 131.2, 129.7, 128.5, 72.0, 36.3, 32.1, 29.73, 29.72, 29.5, 25.7, 22.9, 20.3, 14.3.
HRMS (ESI-TOF) Calcd for $C_{17}H_{26}O_2$ $(M+Na)^+$ 285.1825. Found 285.1823.
HPLC (OJ-H, 0.46*25 cm, 5 μm, hexane/isopropanol = 99.5/0.5, flow rate = 0.5 mL/min, detection at 230 nm) retention time = 10.4 min (major) and 10.9 min (minor).
$[\alpha]^{25}D$ = −5.90 (c 1.0, CH_2Cl_2).

(*R*)-Decan-2-yl 4-methylbenzoate (5b)

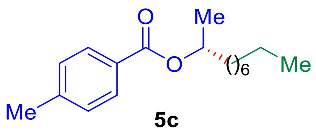

5c

Following **General procedure (H)**, the reaction of **1b** (32.4 mg, 0.2 mmol) with **2v** (89.7 mg, 0.8 mmol) afforded **5c** as colorless oil (29.3 mg; 53% yield; 91% ee) by flash column chromatography on silica gel (eluent: PE:EA = 100:1).

^{1}H NMR (400 MHz, $CDCl_3$) δ 7.93 (d, J = 8.1 Hz, 2H), 7.23 (d, J = 7.9 Hz, 2H), 5.18 – 5.10 (m, 1H), 2.40 (s, 3H), 1.78 – 1.69 (m, 1H), 1.64 – 1.55 (m, 1H), 1.44 – 1.25 (m, 15H), 0.87 (t, J = 6.6 Hz, 3H).

^{13}C NMR (101 MHz, $CDCl_3$) δ 166.5, 143.4, 129.7, 129.1, 128.3, 71.6, 36.2, 32.0, 29.62, 29.63, 29.4, 25.6, 22.8, 21.7, 20.2, 14.2.

HRMS (ESI-TOF) Calcd for $C_{18}H_{28}O_2$ $(M+Na)^+$ 299.1982. Found 299.1978.

HPLC (OJ-H, 0.46*25 cm, 5 μm, hexane/isopropanol = 99.5/0.5, flow rate = 0.5 mL/min, detection at 230 nm) retention time = 11.3 min (minor) and 12.2 min (major).

$[\alpha]^{25}D$ = −6.70 (c 1.0, CH_2Cl_2).

(*R*)-Decan-2-yl 4-methoxybenzoate (5c)

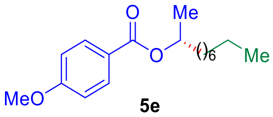

5e

Following **General procedure (H)**, the reaction of **1d** (35.6 mg, 0.2 mmol) with **2v** (89.7 mg, 0.8 mmol) afforded **5e** as colorless oil (33.3 mg; 57% yield; 91% ee) by flash column chromatography on silica gel (eluent: PE:EA = 50:1).

^{1}H NMR (400 MHz, $CDCl_3$) δ 8.01 – 7.97 (m, 2H), 6.93 – 6.89 (m, 2H), 5.15 – 5.08 (m, 1H), 3.85 (s, 3H), 1.75 – 1.68 (m, 1H), 1.63 – 1.54 (m, 1H), 1.40 – 1.25 (m, 15H), 0.87 (t, J = 6.4 Hz, 3H).

^{13}C NMR (101 MHz, $CDCl_3$) δ 166.2, 163.3, 131.6, 123.5, 113.6, 71.5, 55.5, 36.2, 32.0, 29.63, 29.64, 29.4, 25.6, 22.8, 20.3, 14.2.

HRMS (ESI-TOF) Calcd for $C_{18}H_{28}O_3$ $(M+Na)^+$ 315.1931. Found 315.1927.

HPLC (IC, 0.46*25 cm, 5 μm, hexane/isopropanol = 99/1, flow rate = 0.5 mL/min, detection at 254 nm) retention time = 11.2 min (major) and 12.2 min (minor).

$[\alpha]^{25}D$ = −29.7 (c 1.0, CH_2Cl_2).

(*R*)-Decan-2-yl 4-(methylthio)benzoate (5d)

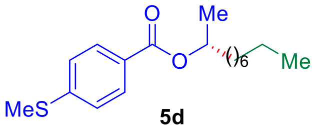

5d

Following **General procedure (H)**, the reaction of **1e** (38.8 mg, 0.2 mmol) with **2v** (89.7 mg, 0.8 mmol) afforded **5d** as colorless oil (36.9 mg; 60% yield; 85% ee) by flash column chromatography on silica gel (eluent: PE:EA = 50:1).

^{1}H NMR (400 MHz, $CDCl_3$) δ 7.95 – 7.91 (m, 2H), 7.26 – 7.23 (m, 2H), 5.16 – 5.09 (m, 1H), 2.51 (s, 3H), 1.75 – 1.67 (m, 1H), 1.62 – 1.54 (m, 1H), 1.42 – 1.25 (m, 15H), 0.87 (t, J = 6.6 Hz, 3H).

^{13}C NMR (101 MHz, $CDCl_3$) δ 166.1, 145.2, 130.0, 127.2, 125.0, 71.8, 36.2, 32.0, 29.62, 29.63, 29.4, 25.6, 22.8, 20.2, 15.0, 14.2.

HRMS (ESI-TOF) Calcd for $C_{18}H_{28}O_2S$ $(M+Na)^+$ 331.1702. Found 331.1698.
HPLC (OD-H, 0.46*25 cm, 5 μm, hexane/isopropanol = 99/1, flow rate = 1.0 mL/min, detection at 254 nm) retention time = 5.5 min (minor) and 6.6 min (major).
$[\alpha]^{25}D = -23.7$ (c 0.4, CH_2Cl_2).

(*R*)-Decan-2-yl 4-fluorobenzoate (5e)

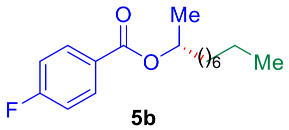

5b

Following **General procedure (H)**, the reaction of **1h** (33.2 mg, 0.2 mmol) with **2v** (89.7 mg, 0.8 mmol) afforded **5b** as colorless oil (28.6 mg; 51% yield; 82% ee) by flash column chromatography on silica gel (eluent: PE:EA = 100:1).
^{1}H NMR (400 MHz, $CDCl_3$) δ 8.07 – 8.02 (m, 2H), 7.13 – 7.07 (m, 2H), 5.17 – 5.09 (m, 1H), 1.77 – 1.68 (m, 1H), 1.62 – 1.55 (m, 1H), 1.40 – 1.25 (m, 15H), 0.87 (t, *J* = 6.6 Hz, 3H).
^{13}C NMR (101 MHz, $CDCl_3$) δ 165.8 (d, *J* = 254.5 Hz), 132.1 (d, *J* = 9.1 Hz), 127.3 (d, *J* = 3.0 Hz), 115.5 (d, *J* = 22.0 Hz), 165.4, 72.1, 36.2, 32.0, 29.61, 29.62, 29.4, 25.6, 22.8, 20.2, 14.2.
^{19}F NMR (376 MHz, $CDCl_3$) δ -106.28 – -106.38 (m).
HRMS (ESI-TOF) Calcd for $C_{17}H_{25}FO_2$ $(M+Na)^+$ 303.1731. Found 303.1724.
HPLC (OD-H, 0.46*25 cm, 5 μm, hexane/isopropanol = 99.5/0.5, flow rate = 1.0 mL/min, detection at 230 nm) retention time = 15.6 min (major) and 16.9 min (minor).
$[\alpha]^{25}D = -23.2$ (c 1.0, CH_2Cl_2).

(*R*)-7-Phenylheptan-2-yl benzoate (5f)

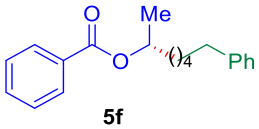

5f

Following **General procedure (H)**, the reaction of **1a** (29.6 mg, 0.2 mmol) with **2w** (116.9 mg, 0.8 mmol) afforded **5a** as colorless oil (26.4 mg; 45% yield; 91% ee) by flash column chromatography on silica gel (eluent: PE:EA = 50:1).
^{1}H NMR (400 MHz, $CDCl_3$) δ 8.04 – 8.01 (m, 2H), 7.54 – 7.50 (m, 1H), 7.43 – 7.39 (m, 2H), 7.26 – 7.21 (m, 2H), 7.17 – 7.13 (m, 3H), 5.16 – 5.11 (m, 1H), 2.58 (t, *J* = 7.6 Hz, 2H), 1.75 – 1.69 (m, 1H), 1.65 – 1.54 (m, 3H), 1.45 – 1.33 (m, 4H), 1.31 (d, *J* = 6.3 Hz, 3H).
^{13}C NMR (101 MHz, $CDCl_3$) δ 166.3, 142.8, 132.8, 131.0, 129.6, 128.5, 128.40, 128.35, 125.7, 71.8, 36.1, 36.0, 31.5, 29.2, 25.4, 20.2.
HRMS (ESI-TOF) Calcd for $C_{20}H_{24}O_2$ $(M+Na)^+$ 319.1669. Found 319.1664.
HPLC (OD-H, 0.46*25 cm, 5 μm, hexane/isopropanol = 99/1, flow rate = 1.0 mL/min, detection at 230 nm) retention time = 7.3 min (major) and 10.6 min (minor).
$[\alpha]^{25}D = -20.60$ (c 1.0, CH_2Cl_2).

(*R*)-8-Phenyloctan-2-yl benzoate (5g)

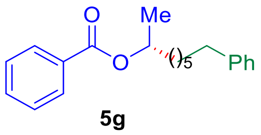

5g

Following **General procedure (H)**, the reaction of **1a** (29.6 mg, 0.2 mmol) with **2x** (128.1 mg, 0.8 mmol) afforded **5g** as colorless oil (30.1 mg; 50% yield; 90% ee) by flash column chromatography on silica gel (eluent: PE:EA = 50:1).

^{1}H NMR (600 MHz, $CDCl_3$) δ 8.04 – 8.02 (m, 2H), 7.54 – 7.51 (m, 1H), 7.43 – 7.40 (m, 2H), 7.26 – 7.23 (m, 2H), 7.16 – 7.14 (m, 3H), 5.17 – 5.11 (m, 1H), 2.58 (t, *J* = 7.8 Hz, 2H), 1.75 – 1.69 (m, 1H), 1.62 – 1.56 (m, 3H), 1.42 – 1.31 (m, 9H).

^{13}C NMR (151 MHz, $CDCl_3$) δ 166.4, 142.9, 132.8, 131.1, 129.6, 128.5, 128.41, 128.35, 125.7, 71.8, 36.2, 36.0, 31.5, 29.5, 29.3, 25.5, 20.2.

HRMS (ESI-TOF) Calcd for $C_{21}H_{26}O_2$ $(M+Na)^+$ 333.1825. Found 333.1820.

HPLC (OD-H, 0.46*25 cm, 5 μm, hexane/isopropanol = 99/1, flow rate = 1.0 mL/min, detection at 230 nm) retention time = 7.9 min (major) and 8.4 min (minor).

$[\alpha]^{25}$D = −18.70 (c 1.0, CH_2Cl_2).

Following **General procedure (H)**, the reaction of **1a** (29.6 mg, 0.2 mmol) with **2z** (128.1 mg, 0.8 mmol) afforded **5g** as colorless oil (7.8 mg; 13% yield; 86% ee) by flash column chromatography on silica gel (eluent: PE:EA = 50:1).

^{1}H NMR (400 MHz, $CDCl_3$) δ 8.06 – 8.04 (m, 2H), 7.56 – 7.53 (m, 1H), 7.43 – 7.40 (m, 2H), 7.26 – 7.23 (m, 2H), 7.16 – 7.14 (m, 3H), 5.17 – 5.11 (m, 1H), 2.60 (t, *J* = 7.8 Hz, 2H), 1.75 – 1.69 (m, 1H), 1.69 – 1.52 (m, 3H), 1.42 – 1.31 (m, 9H).

^{13}C NMR (101 MHz, $CDCl_3$) δ 166.4, 142.9, 132.8, 131.0, 129.6, 128.5, 128.41, 128.35, 125.7, 71.8, 36.2, 36.0, 31.5, 29.5, 29.3, 25.5, 20.2.

HRMS (ESI-TOF) Calcd for $C_{21}H_{26}O_2$ $(M+Na)^+$ 333.1825. Found 333.1824.

HPLC (AD-H, 0.46*25 cm, 5 μm, hexane/isopropanol = 99/1, flow rate = 1.0 mL/min, detection at 230 nm) retention time = 7.8 min (major) and 8.7 min (minor).

$[\alpha]^{25}$D = −17.92 (c 1.0, CH_2Cl_2).

Unsuccessful examples

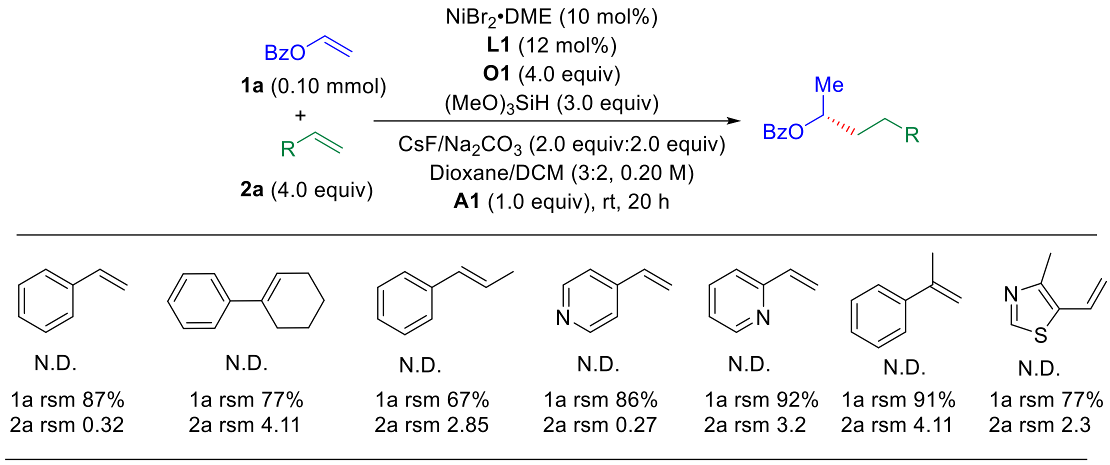

Figure S1. The reaction outcomes using styrenes.

Determination of head-to-head by-product

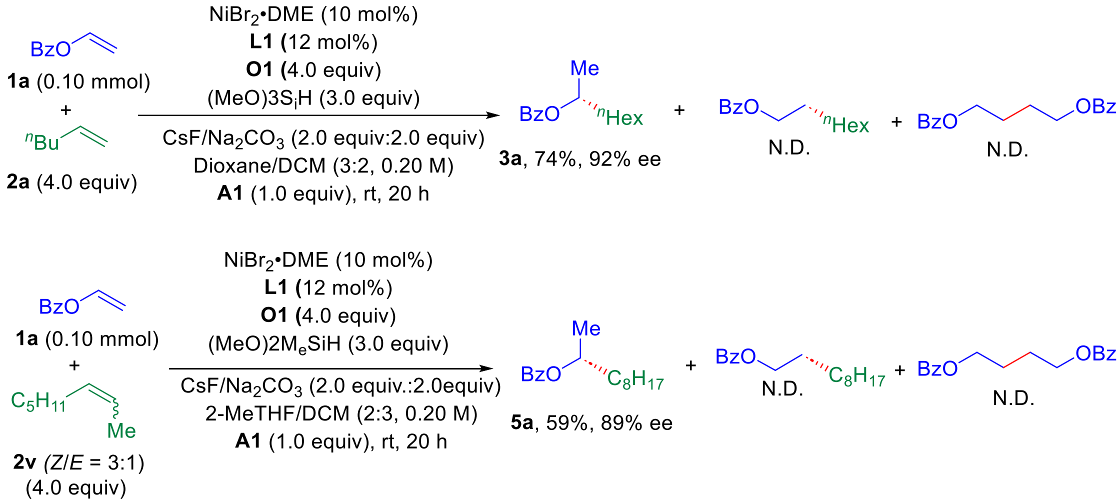

Figure S2. Determination of by-product.

VI. Scale-up Experiment and Synthetic Application

6.1 Scale-up experiment

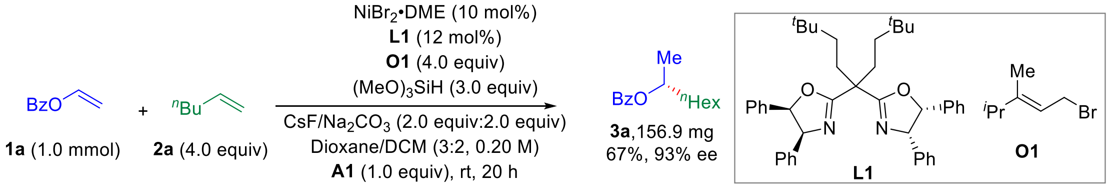

To an oven-dried 100 mL Teflon-screw cap tube containing a magnetic stir was charged with $NiBr_2$·DME (31.0 mg, 0.10 mmol, 10 mol%) and **L1** (75.0 mg, 0.24 mmol, 12 mol%) in a nitrogen-filled glove-box. Subsequently, anhydrous solvent

(Dioxane/DCM, 3:2, 0.20 M) was added. The mixture was stirred for 5-10 min at room temperature. Then CsF/Na_2CO_3 (300.4/210.2 mg, 2.0 equiv/2.0 equiv), olefin **1** (148.1mg, 1.0 mmol, 1.0 equiv), **2a** (0.5 mL, 4.0 mmol, 4.0 equiv) were added. **O1** (648.0 mg, 4.0 mmol, 4.0 equiv) and A1 (147.0 mg, 2.0 mmol, 1.0 equiv) were added subsequently. Finally, (380.0 uL, 3.0 mmol, 3.0 equiv) was added dropwise under N_2 atmosphere, and the reaction was stirred at 25 °C for 20 h at 1400 rpm. After the reaction was completed, the reaction mixture was H_2O (3.0 mL) and EtOAc (15.0 mL). The organic phase was separated, then the aqueous phase was extracted with EtOAc (2 × 15.0 mL). The combined organic phase was dried over Na_2SO_4. The crude mixture was purified by flash column chromatography on silica gel using PE/EA (100:1) as eluent to give **3a** as colorless liquid (156.9 mg, 67% yield, 93% ee).

6.2 Preparation of intermediates used in the synthesis of paleic acid[31]

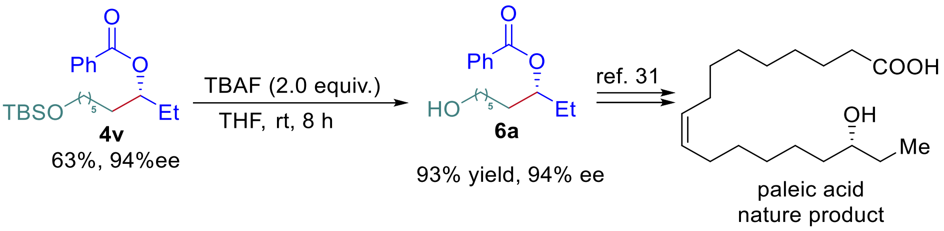

(*R*)-9-Hydroxynonan-3-yl benzoate (6a)[32]

To a round-bottom flask containing of **4v** (37.8 mg, 0.1 mmol) dissolved in anhydrous THF (1 mL) was added TBAF (1 M in THF; 0.2 mL, 0.2 mmol, 2.0 equiv) and stirred at rt for 8 h. The reaction mixture was evaporated and diluted with diethyl ether. The organics was washed with saturated bicarbonate, dried over sodium sulphate, filtered and concentrated in vacuo. The crude mixture was purified by flash column chromatography on silica gel using PE/EA (5:1) as eluent to give **6a** as colorless liquid (24.6 mg, 93% yield, 94% ee).

^{1}H NMR (600 MHz, $CDCl_3$) δ 8.08 – 8.01 (m, 2H), 7.59 – 7.52 (m, 1H), 7.47 – 7.40 (m, 2H), 5.12 – 5.02 (m, 1H), 3.62 (t, J = 6.6 Hz, 2H), 1.74 – 1.61 (m, 4H), 1.57 – 1.53 (m, 3H), 1.43 – 1.29 (m, 6H), 0.95 (t, J = 7.4 Hz, 3H).

^{13}C NMR (151 MHz, $CDCl_3$) δ 166.6, 132.9, 131.0, 129.7, 128.4, 76.2, 63.1, 33.7, 32.8, 29.4, 27.2, 25.7, 25.4, 9.8.

The characterization NMR data are consistent with the reported data.[33]

HRMS (ESI-TOF) Calcd for $C_{16}H_{25}O_3$ $(M+H)^+$ 265.1798. Found 265.1797.

HPLC (OJ-H, 0.46*25 cm, 5 μm, hexane/isopropanol = 95/5, flow rate = 1.0 mL/min, detection at 230 nm) retention time = 7.9 min (minor) and 8.8 min (major).

$[\alpha]^{23}$D = −8.9 (c 0.4, $CHCl_3$); 94% ee.

6.3 Preparation of intermediates used in the synthesis of (*S*)-Curvularin[34]

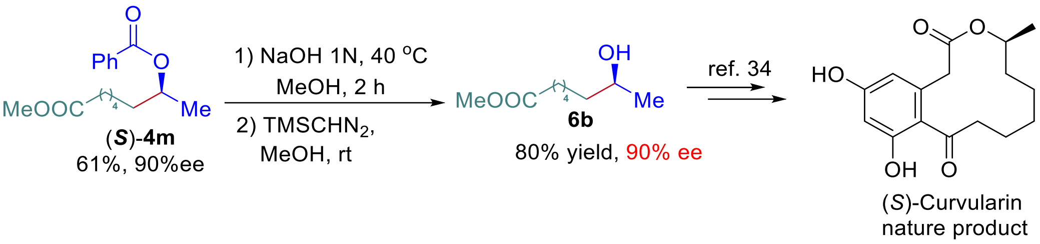

Methyl (*S*)-7-hydroxyoctanoate (6b)[35]

To the solution of (*S*)-**4m** (27.8 mg, 0.1 mmol) in 860.0 uL of methanol and 570.0 uL of THF was added 1.0 N solution of NaOH (1.3 mL). The mixture was stirred 2 h at 40 °C. 1.0 N solution of $NaHSO_4$ (1.4 mL) was then added for neutralization. The reaction was diluted in water (1.0 mL) and extracted with ethyl acetate (3 × 5.0 mL). The organic layers were dried with Na_2SO_4 and evaporated. The crude was dissolved in MeOH (0.5 ml), and (trimethylsily)diazomethane (2.0 M solution in hexanes, approximately 0.5 ml)) was added over two minutes at room temperature. After evaporation of solvents, the crude product was purified by flash chromatography on silica gel to give of **6b** as colorless liquid (22.2 mg, 80% yield over two steps). The ee was determined by transforming **6b** to corresponding benzoyl protected product (*S*)-**4m**.

The characterization NMR data are consistent with the reported data.[36]

^{1}H NMR (400 MHz, $CDCl_3$) δ 3.84 – 3.73 (m, 1H), 3.65 (s, 3H), 2.30 (t, *J* = 7.5 Hz, 2H), 1.70 – 1.58 (m, 2H), 1.55 (s, 1H), 1.48 – 1.38 (m, 3H), 1.38 – 1.28 (m, 3H), 1.17 (d, *J* = 6.2 Hz, 3H).

^{13}C NMR (101 MHz, $CDCl_3$) δ 174.4, 68.1, 51.6, 39.2, 34.1, 29.2, 25.5, 25.0, 23.6.

$[\alpha]^{25}D$ = +5.88 (c 1.0, CH_2Cl_2).

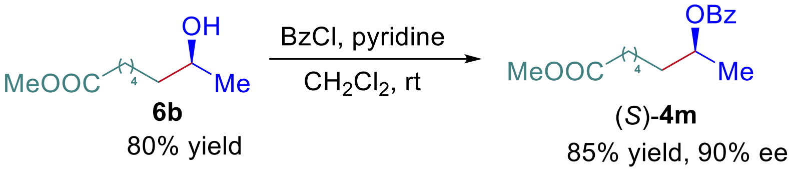

To a solution of **6b** (0.1 mmol) in DCM (0.1 mL) and pyridine (0.1 mL), was added BzCl (28.0 mg, 0.2 mmol, 2.0 equiv). The resulting mixture was stirred at rt for overnight. After the reaction reached completion, the reaction mixture was filtered through a celite pad. The filtrate was concentrated under vacuum. The crude residue was purified by column chromatography on silica gel with PE/EA (20:1) to afford product (*S*)-**4m** (23.6 mg, 85% yield, 90% ee).

^{1}H NMR (400 MHz, $CDCl_3$) δ 8.04 – 8.02 (m, 2H), 7.57 – 7.52 (m, 1H), 7.45 – 7.41 (m, 2H), 5.19 – 5.11 (m, 1H), 3.65 (s, 3H), 2.30 (t, *J* = 7.5 Hz, 2H), 1.77 – 1.70 (m, 1H), 1.67 – 1.57 (m, 3H), 1.44 – 1.35 (m, 4H), 1.33 (d, *J* = 6.2 Hz, 3H).

^{13}C NMR (101 MHz, $CDCl_3$) δ 174.3, 166.3, 132.9, 131.1, 129.6, 128.3, 71.7, 51.6, 36.0, 34.1, 29.1, 25.2, 24.9, 20.2.

HRMS (ESI-TOF) Calcd for $C_{16}H_{23}O_4$ $(M+Na)^+$ 301.1410. Found 301.1409.

HPLC (AD-H, 0.46*25 cm, 5 μm, hexane/isopropanol = 99/1, flow rate = 1.0 mL/min, detection at 230 nm) retention time = 10.6 min (minor) and 12.3 min (major).
$[\alpha]^{25}D$ = +23.4 (c 1.0, CH_2Cl_2).

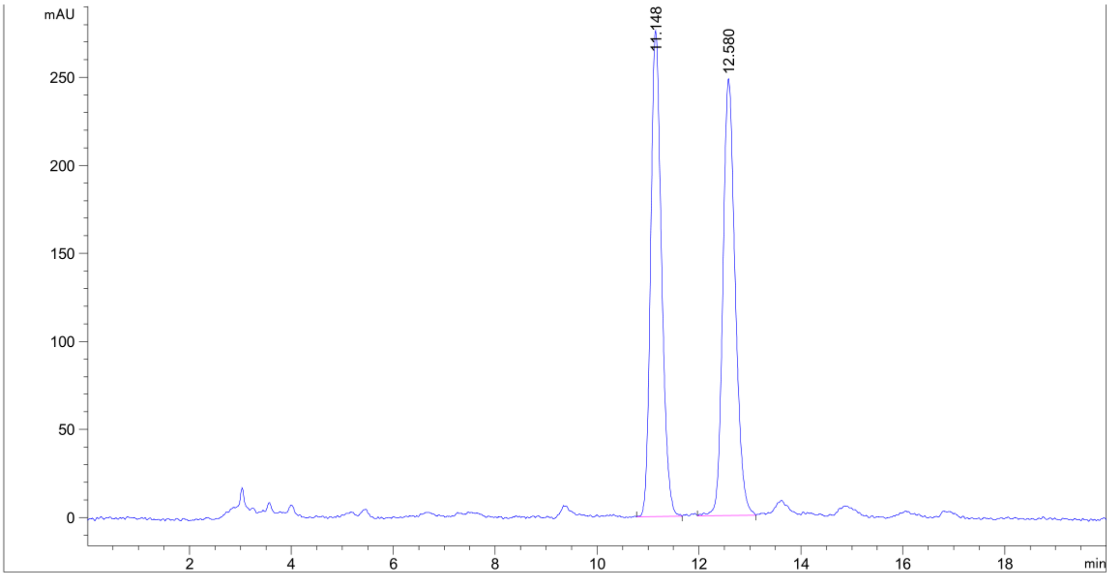

Peak #	RetTime [min]	Type	Width [min]	Area [mAU*s]	Height [mAU]	Area %
1	11.148	VV R	0.2268	4089.81836	276.12997	49.5225
2	12.580	VB R	0.2561	4168.68896	248.03644	50.4775

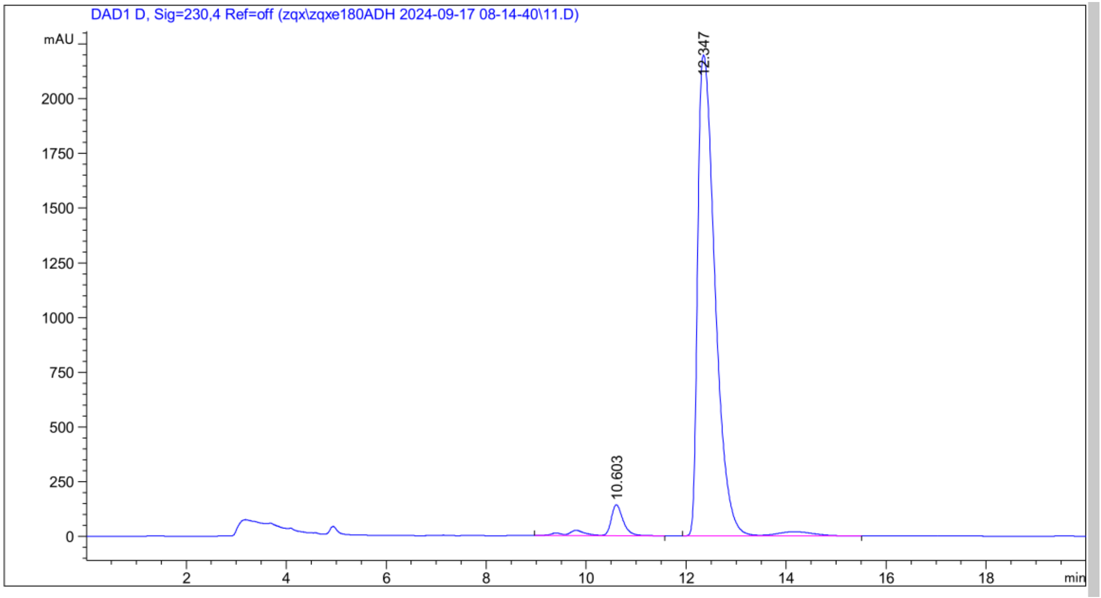

Peak #	RetTime [min]	Type	Width [min]	Area [mAU*s]	Height [mAU]	Area %
1	10.603	VB R	0.2499	3034.57397	142.08772	5.3565
2	12.347	BV R	0.3713	5.36178e4	2198.86328	94.6435

6.4 Preparation of intermediates used in the synthesis of liquid crystals[37]

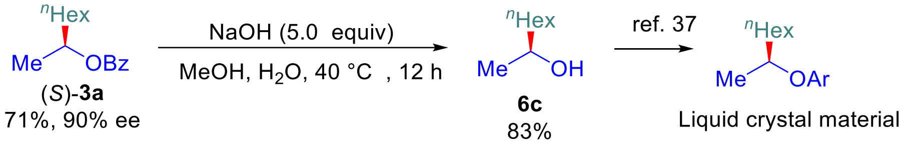

(*S*)-2-Octanol (6c)[38]

To a solution of (*S*)-**3a** (44.0 mg, 0.2 mmol) in MeOH (0.5 mL) was added NaOH aq (40.0 mg, 1.0 mmol, 5.0 equiv in 0.5 mL H_2O), then the mixture was stirred at 40 °C for 12 hours. After the reaction completed, the reaction mixture was concentrated under vacuum and H_2O (1.0 mL) was added. The aqueous layer was extracted with ethyl acetate. The combined organic layers were dried over $MgSO_4$ and concentrated in vacuo. The resulting residue was purified by column chromatography on silica gel with PE/EA (5:1) to afford the desired product **6c** (21.6 mg, 83% yield), as colorless oil. The characterization NMR data are consistent with the reported data.[39]

^{1}H NMR (400 MHz, $CDCl_3$) δ 3.86 – 3.68 (m, 1H), 1.84 (s, 1H), 1.50 – 1.34 (m, 3H), 1.23 – 1.34 (m, 7H), 1.16 (d, *J* = 6.2 Hz, 3H), 0.87 (t, *J* = 6.8 Hz, 3H).

^{13}C NMR (101 MHz, $CDCl_3$) δ 68.3, 39.4, 31.9, 29.4, 25.8, 23.5, 23.5, 22.7, 14.2.

6.5 Synthesis of chiral diol

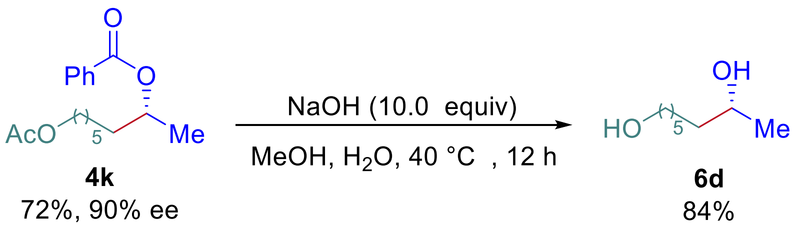

(*R*)-Octane-1,7-diol (6d)[38]

To a solution of **4k** (58.4 mg, 0.2 mmol) in MeOH (0.5 mL) was added NaOH aq (80.0 mg, 2.0 mmol, 10.0 equiv in 0.5 mL H_2O), then the mixture was stirred at 40 °C for 12 hours. After the reaction completed, the reaction mixture was concentrated under vacuum and H_2O (1.0 mL) was added. The aqueous layer was extracted with ethyl acetate. The combined organic layers were dried over $MgSO_4$ and concentrated in vacuo. The resulting residue was purified by column chromatography on silica gel with PE/EA (5:1) to afford the desired product **6c** (23.4 mg, 83% yield), as colorless oil.

The characterization NMR data are consistent with the reported data.[40]
^{1}H NMR (400 MHz, $CDCl_3$) δ 3.82 – 3.68 (m, 1H), 3.58 (t, *J* = 6.6 Hz, 2H), 2.45 (s, 2H), 1.60 – 1.48 (m, 2H), 1.48 – 1.22 (m, 8H), 1.15 (d, *J* = 6.2 Hz, 3H).
^{13}C NMR (101 MHz, $CDCl_3$) δ 68.1, 62.8, 39.2, 32.6, 29.4, 25.8, 25.7, 23.5.
$[\alpha]^{25}D$ = −2.93 (c 1.0, CH_2Cl_2).

(*R*)-Octane-1,7-diyl dibenzoate (12)[38]

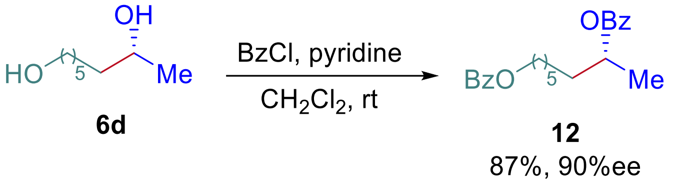

To a solution of **6d** (0.1 mmol) in DCM (0.1 mL) and pyridine (0.2 mL), was added BzCl (56.0 mg, 0.4 mmol, 4.0 equiv), then stirred at rt overnight. After the reaction completed, the reaction mixture was filtered through a celite pad. The filtrate was concentrated under vacuum. The crude residue was purified by column chromatography on silica gel with PE/EA (20:1) to afford product **7a** (30.8 mg, 87% yield, 90% ee).
^{1}H NMR (400 MHz, $CDCl_3$) δ 8.10 – 7.95 (m, 4H), 7.59 – 7.50 (m, 2H), 7.48 – 7.39 (m, 4H), 5.23 – 5.11 (m, 1H), 4.31 (t, *J* = 6.6 Hz, 2H), 1.83 – 1.71 (m, 3H), 1.70 – 1.57 (m, 1H), 1.52 – 1.37 (m, 6H), 1.34 (d, *J* = 6.3 Hz, 3H).
^{13}C NMR (101 MHz, $CDCl_3$) δ 166.8, 166.3, 132.9, 132.8, 131.0, 130.6, 129.65, 129.62, 128.44, 128.41, 71.7, 65.1, 36.1, 29.3, 28.8, 26.1, 25.5, 20.2.
HRMS (ESI-TOF) Calcd for $C_{22}H_{27}O_4$ $(M+H)^+$ 355.1902. Found 355.1902.
HPLC (AD-H, 0.46*25 cm, 5 μm, hexane/isopropanol = 98/2, flow rate = 1.0 mL/min, detection at 254 nm) retention time = 8.5 min (major) and 9.5 min (minor).
$[\alpha]^{25}D$ = −5.10 (c 1.0, CH_2Cl_2).

VII. Assignment of Absolute Configuration of the Prodcuts

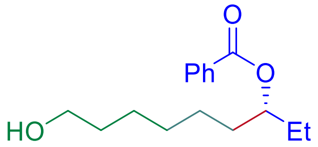

6a

(*R*)-9-Hydroxynonan-3-yl benzoate (**6a**) The absolute configuration of this compound has been reported.[33,32] The use of (**4*S*,4'*S*,5*R*,5'*R***)-**L1** as ligand afforded **6a** in (*R*) configuration, by comparing the data of optical rotation and HPLC traces with the published data.
Optical rotation: $[\alpha]^{23}D$ = −8.9 (c 0.4, $CHCl_3$); 94% ee, from (**4*S*,4'*S*,5*R*,5'*R***)-**L1**
Lit1.: $[\alpha]^{23}D$ = −9.5 (c 1.0, $CHCl_3$); 90% ee, (*R*) configuration.[33]
Lit2.: $[\alpha]^{20}D$ = −8.6 (c 1.3, $CHCl_3$); 99% ee, (*R*) configuration.[32]

Lit1 HPLC trace for *R*-6a

HPLC Analysis: CHIRALCEL OJ column (5% *i*-PrOH in hexane, 1.0 mL/min).
Compound **40**: 90% ee from (*S*,*S*)-**L***

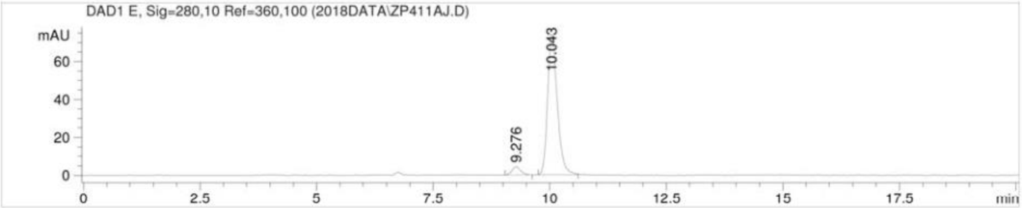

Peak #	RetTime [min]	Type	Width [min]	Area [mAU*s]	Height [mAU]	Area %
1	9.276	BB	0.1917	55.18505	4.24286	4.8447
2	10.043	BB	0.2247	1083.89282	74.09279	95.1553

6a HPLC trace

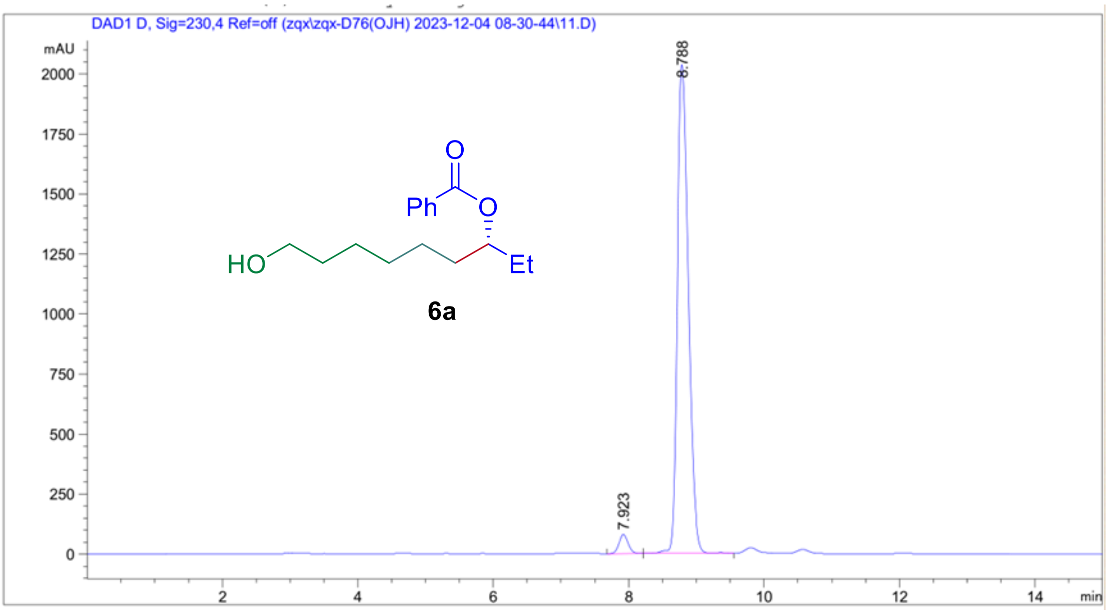

Peak #	RetTime [min]	Type	Width [min]	Area [mAU*s]	Height [mAU]	Area %
1	7.923	BB	0.1480	759.67529	80.60741	3.1837
2	8.788	BV R	0.1760	2.31020e4	2036.51660	96.8163

VIII. Mechanistic Experiments

7.1 Deuterium labeling experiments

7 was synthesized following the reported method.[41]

(But-3-en-1-yl-4,4-d2)benzene (7)

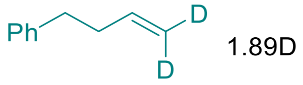

7

^{1}H NMR (400 MHz, $CDCl_3$) δ 7.30 – 7.23 (m, 2H), 7.19 – 7.13 (m, 3H), 5.90 – 5.76 (m, 1H), 5.04 – 4.91(m, 0.11H), 2.69 (dd, *J* = 9.0, 6.6 Hz, 2H), 2.41 – 2.29 (m, 2H).
^{13}C NMR (101 MHz, $CDCl_3$) δ 141.7, 137.7, 128.2, 128.1, 125.6, 114.41, 35.2, 35.2.

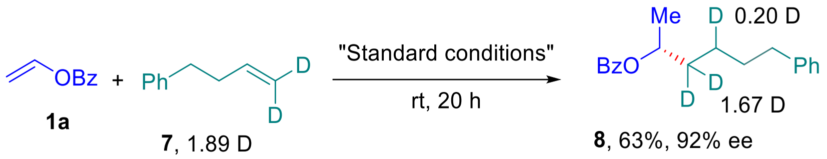

To an oven-dried 10 mL Teflon-screw cap tube containing a magnetic stir was charged with $NiBr_2$·DME (6.2 mg, 0.02 mmol, 10.0 mol%) and **L1** (15.0 mg, 0.024 mmol, 12 mol%) in a nitrogen-filled glove-box. Subsequently, anhydrous solvent (Dioxane/DCM, 3:2, 0.20 M) was added. The mixture was stirred for 5-10 min at room temperature. Then CsF/Na_2CO_3 (60.8/42.4 mg, 0.4 mmol/0.4 mmol, 2.0 equiv/2.0 equiv), olefin **1** (0.20 mmol, 1.0 equiv), **7** (105.8 mg, 0.80 mmol, 4.0 equiv) were added. **O1** (129.6 mg, 0.80 mmol, 4.0 equiv) and **A1** (29.4 mg, 0.2 mmol, 1.0 equiv) were added subsequently. Finally, $(MeO)_3SiH$ (76.0 uL, 0.60 mmol, 3.0 equiv) was added dropwise under N_2 atmosphere, and the reaction was stirred at 25 °C for 20 h at 1400 rpm. After the reaction was completed, the reaction mixture was H_2O (0.5 mL) and EtOAc (3.0 mL). The organic phase was separated, then the aqueous phase was extracted with EtOAc (2 × 3.0 mL). The combined organic phase was dried over Na_2SO_4. The crude mixture was purified by flash column chromatography to give **8** as a colorless oil (35.9 mg, 63% yield, 92% ee).
^{1}H NMR (400 MHz, $CDCl_3$) δ 8.07 – 7.97 (m, 2H), 7.58 – 7.49 (m, 1H), 7.42 (dd, *J* = 8.4, 6.9 Hz, 2H), 7.28 – 7.20 (m, 2H), 7.17 – 7.13 (m, 3H), 5.17 – 5.11 (m, 1H), 2.60 (t, *J* = 7.7 Hz, 2H), 1.76 – 1.71 (m, 0.22H), 1.68 – 1.61 (m, 2.11H), 1.47 – 1.37 (m, 1.80H), 1.32 (d, *J* = 6.3 Hz, 3H).
^{13}C NMR (101 MHz, $CDCl_3$) δ 166.3, 142.5, 132.8, 131.0, 129.6, 128.5, 128.39, 128.37, 125.8, 71.7, 71.6, 35.9, 35.8, 31.33, 31.25, 25.04, 24.94, 20.2, 20.1.
^{2}H NMR (61 MHz, CH_3Cl) δ 1.77, 1.64, 1.46.
HRMS (ESI-TOF) Calcd for $C_{19}H_{20}D_2O_2$ $(M+Na)^+$ 307.1638. Found 307.1636.
HPLC (OD-H, 0.46*25 cm, 5 µm, hexane/isopropanol = 99/1, flow rate = 1.0 mL/min, detection at 230 nm) retention time = 7.5 min (major) and 9.4 min (minor).

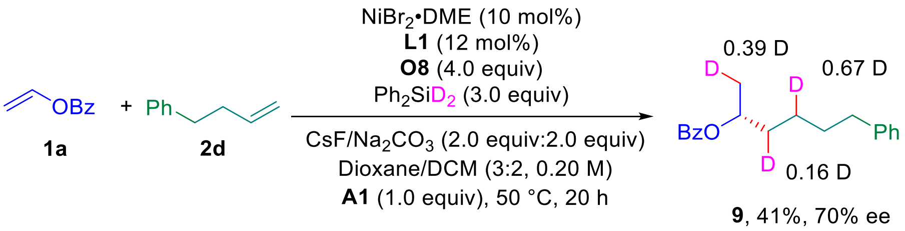

To an oven-dried 10.0 mL Teflon-screw cap tube containing a magnetic stir was charged with $NiBr_2$·DME (6.2 mg, 0.02 mmol, 10.0 mol%) and **L1** (15.0 mg, 0.024 mmol, 12.0 mol%) in a nitrogen-filled glove-box. Subsequently, anhydrous solvent (Dioxane/DCM, 3:2, 0.20 M) was added. The mixture was stirred for 5-10 min at room temperature. Then CsF/Na_2CO_3 (60.8/42.4 mg, 0.4 mmol/0.4 mmol, 2.0 equiv/2.0 equiv), olefin **1** (0.20 mmol, 1.0 equiv), **2d** (105.7 mg, 0.80 mmol, 4.0 equiv) were added. **O8** (92.0 uL, 0.80 mmol, 4.0 equiv) and **A1** (29.4.0 mg, 0.2 mmol, 1.0 equiv) were added subsequently. Finally, Ph_2SiD_2 (111.8 mg, 0.60 mmol, 3.0 equiv) was added dropwise under N_2 atmosphere, and the reaction was stirred at 50 °C for 20 h at 1400 rpm. After the reaction was completed, the reaction mixture was H_2O (0.5 mL) and EtOAc (3.0 mL). The organic phase was separated, then the aqueous phase was extracted with EtOAc (2 × 3.0 mL). The combined organic phase was dried over Na_2SO_4. The crude mixture was purified by flash column chromatography to give **9** as a colorless oil (23.3 mg, 41% yield, 70% ee).

^{1}H NMR (600 MHz, $CDCl_3$) δ 8.10 – 7.96 (m, 2H), 7.59 – 7.51 (m, 1H), 7.48 – 7.41 (m, 2H), 7.28 – 7.24 (m, 2H), 7.21 – 7.09 (m, 3H), 5.21 – 5.12 (m, 1H), 2.63 (t, *J* = 7.7 Hz, 2H), 1.82 – 1.75 (m, 0.91H), 1.71 – 1.63 (m, 2.93H), 1.51 – 1.41 (m, 1.61H), 1.35 – 1.31 (m, 2.33H).

^{13}C NMR (151 MHz, $CDCl_3$) δ 166.4, 142.6, 134.5, 132.8, 131.0, 129.6, 128.5, 128.4, 128.4, 125.8, 71.73, 71.69, 35.99, 35.96, 35.88, 35.86, 31.39, 31.36, 31.29, 25.2, 20.2.

^{2}H NMR (61 MHz, CH_3Cl) δ 1.79, 1.65, 1.47, 1.35.

HRMS (ESI-TOF) Calcd for $C_{19}H_{20}D_2O_2$ $(M+Na)^+$ 307.1638. Found 307.1634.

HPLC (OD-H, 0.46*25 cm, 5 μm, hexane/isopropanol = 99/1, flow rate = 1.0 mL/min, detection at 230 nm) retention time = 7.9 min (major) and 10.0 min (minor).

$[\alpha]^{25}D = -20.6$ (c 1.0, CH_2Cl_2)

10 was synthesized following the reported method.[10, 42]

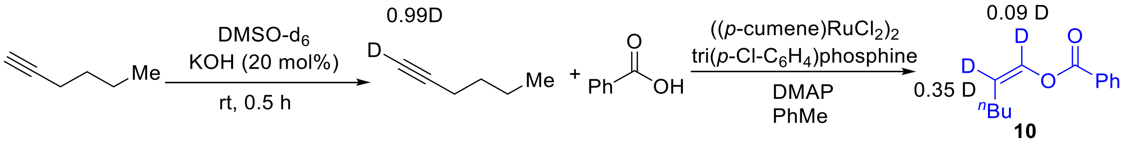

10

In an oven-dried round bottom flask, terminal alkynes (5.0 mmol) and 20 mol % of crushed KOH in 8.0 mL of DMSO-d6 were added. The resulting reaction mixture was stirring at 25 °C for 30 minutes. Progression of the reaction was monitored by crude ^{1}H NMR analysis; after complete consumption of starting material the reaction was cooled to room temperature. The reaction mixture was diluted with CH_2Cl_2 (20.0 mL × 3) and water (10.0 mL × 3). The layers were separated, and the organic layer was washed with aqueous saturated brine solution and dried over Na_2SO_4. Organic layer was concentrated under reduced pressure. Simple distillation technique can also be applied for low boiling point alkynes. **10** were synthesized following general procedure (C).

^{1}H NMR (400 MHz, DMSO-*d*$_6$) δ 8.12 – 7.94 (m, 2H), 7.79 – 7.66 (m, 1H), 7.66 – 7.51 (m, 2H), 7.22 (dq, *J* = 5.0, 1.6 Hz, 0.91H), 5.10 (td, *J* = 7.5, 6.3 Hz, 0.68H), 2.35 – 2.13 (m, 2H), 1.40 – 1.27 (m, 4H), 0.89 (t, *J* = 7.1 Hz, 3H).

^{13}C NMR (101 MHz, DMSO-d_6) 163.8, 134.3, 133.5, 130.0, 129.6, 128.7, 115.1, 31.5, 24.5, 22.4, 14.0.
HRMS (ESI-TOF) Calcd for $C_{13}H_{14}D_2O_2$ $(M+H)^+$ 207.1349. Found 207.1374.

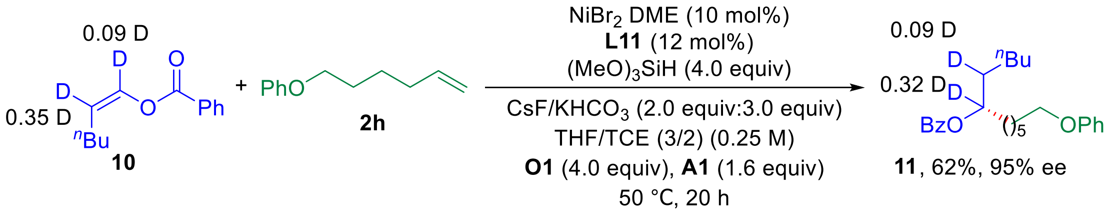

To an oven-dried 10 mL Teflon-screw cap tube containing a magnetic stir was charged with NiBr$_2$·DME (6.2 mg, 0.02 mmol, 10.0 mol%) and **L11** (13.4 mg, 0.024 mmol, 12.0 mol%) in a nitrogen-filled glove-box. Subsequently, anhydrous solvent (Et$_2$O/TCE, 3:2, 0.25 M) was added. The mixture was stirred for 5-10 min at room temperature. Then CSF/KHCO$_3$ (30.4/30.0 mg, 2.0 equiv/3.0 equiv), olefin **10** (0.20 mmol, 1.0 equiv), **2** (0.80 mmol, 4.0 equiv) were added. **O1** (129.6 mg, 0.80 mmol, 4.0 equiv) and **A1** (47.0 mg, 0.32 mmol, 1.6 equiv) were added subsequently. Finally, (MeO)$_3$SiH (101.1 uL, 0.80 mmol, 4.0 equiv) was added dropwise under N$_2$ atmosphere, and the reaction was stirred at 50 °C for 20 h at 1400 rpm. After the reaction was completed, the reaction mixture was H$_2$O (0.5 mL) and EtOAc (3.0 mL). The organic phase was separated, then the aqueous phase was extracted with EtOAc (2 × 3.0 mL). The combined organic phase was dried over Na$_2$SO$_4$. The crude mixture was purified by flash column chromatography on silica gel to give as a colorless oil (51.3 mg, 62% yield, 96% ee).
^{1}H NMR (400 MHz, CDCl$_3$) δ 8.13 – 7.94 (m, 2H), 7.59 – 7.50 (m, 1H), 7.48 – 7.37 (m, 2H), 7.31 – 7.17 (m, 2H), 7.01 – 6.78 (m, 3H), 5.18 – 5.09 (m, 0.91H), 3.92 (t, J = 6.5 Hz, 2H), 1.79 – 1.62 (m, 5.68H), 1.50 – 1.26 (m, 12H), 0.93 – 0.79 (m, 3H).
^{13}C NMR (101 MHz, CDCl$_3$) δ 166.5, 159.2, 132.8, 131.0, 129.7, 129.5, 128.4, 120.6, 114.6, 75.13, 75.08, 67.8, 34.31, 34.27, 34.2, 31.9, 31.8, 29.4, 29.3, 26.1, 25.4, 25.1, 25.0, 22.7, 14.1.
HRMS (ESI-TOF) Calcd for $C_{25}H_{32}D_2O_3$ $(M+Na)^+$ 407.2526. Found 407.2503.
HPLC (IC, 0.46*25 cm, 5 μm, hexane/isopropanol = 98/2, flow rate = 1.0 mL/min, detection at 254 nm) retention time = 5.5 min (minor) and 6.7 min (major).
$[\alpha]^{25}D$ = −4.0 (c 1.0, CH_2Cl_2)

7.2 Reaction of enol benzoates with alkyl halide

To an oven-dried 10 mL Teflon-screw cap tube containing a magnetic stir was charged with NiBr$_2$·DME (3.1 mg, 0.01 mmol, 10 mol%) and **L1** (7.5 mg, 0.012 mmol, 12 mol%) in a nitrogen-filled glove-box. Subsequently, Subsequently, anhydrous solvent (Dioxane/DCM, 3:2, 0.20 M) was added. The mixture was stirred for 5-10 min at room temperature. Then CsF/Na$_2$CO$_3$ (30.4/21.2 mg, 2.0 equiv/2.0 equiv), vinyl benzoate **1a** (14.8 mg, 0.10 mmol, 1.0 equiv), 1-bromohexane (66.0 mg, 0.40 mmol,

4.0 equiv) were added. **O1** (129.6 mg, 0.80 mmol, 4.0 equiv) and phthalimide **A1** (29.4 mg, 0.2 mmol, 1.0 equiv) were added subsequently. Finally, $(MeO)_3SiH$ (76.0 uL, 0.60 mmol, 3.0 equiv) was added dropwise under N_2 atmosphere, and the reaction was stirred at 25 °C for 20 h at 1400 rpm. After the reaction was completed, the reaction mixture was diluted with saturated NH_4Cl (aqueous solution, 0.5 mL) and EtOAc (3.0 mL). Dodecane (23.0 µL, 0.1 mmol) was added as an internal standard and a small aliquot of the organic phase was removed for GC analysis to confirm yield.

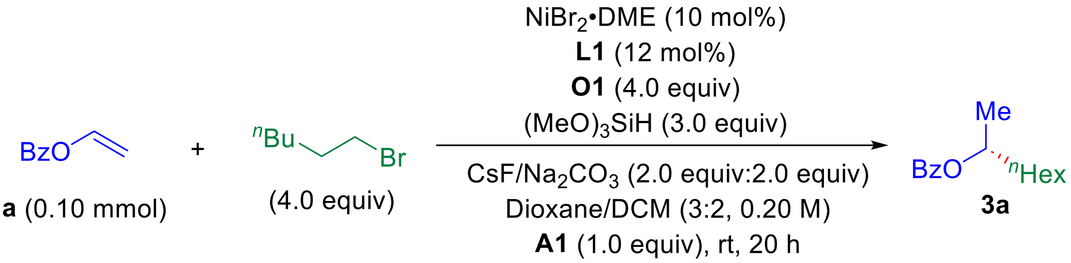

Table S9. Reaction of enol benzoates with alkyl halide.

Entry	variation	recovery of 1a	3a	ee
1	none	90%	N.D.	-
2	no oxidant	87%	N.D.	-

7.3 Monitoring of the reaction progress

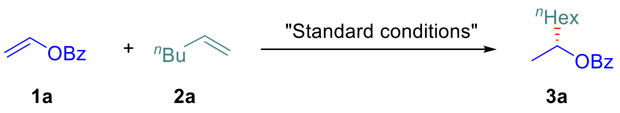

Table S10. Yield and ee value of **3a** during the time course.

entry	time (h)	2	yield of 3a (%)
1	0.6	92	16
2	2	92	33
3	5	92	64
4	10	92	73
5	20	92	81

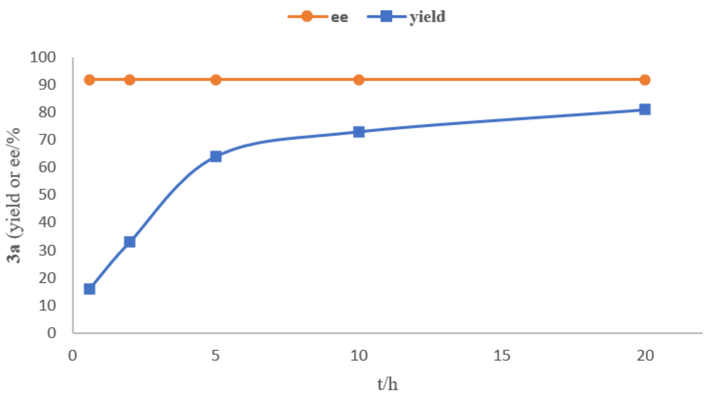

Figure S3. Monitoring the time course of forming **3a**.

To an oven-dried 10 mL Teflon-screw cap tube containing a magnetic stir was charged with $NiBr_2$·DME (3.1 mg, 0.01 mmol, 10 mol%) and **L1** (7.5 mg, 0.012 mmol, 12 mol%) in a nitrogen-filled glove-box. Subsequently, anhydrous solvent (Dioxane/DCM, 3:2, 0.20 M) was added. The mixture was stirred for 5-10 min at room temperature. Then CsF/Na_2CO_3 (30.4/21.2 mg, 2.0 equiv/2.0 equiv), olefin **1a** (0.10 mmol, 1.0 equiv), **2a** (0.40 mmol, 4.0 equiv) were added. **O1** (64.8 mg, 0.40 mmol, 4.0 equiv) and phthalimide **A1** (14.7 mg, 0.1 mmol, 1.0 equiv) were added subsequently. Finally, $(MeO)_3SiH$ (38.0 uL, 0.30 mmol, 3.0 equiv) was added dropwise under N_2 atmosphere, and the reaction was stirred at 25 °C for 20 h at 1400 rpm. After the reaction was completed, the reaction mixture was H_2O (0.5 mL) and EtOAc (3.0 mL). Dodecane (23.0 μL, 0.1 mmol) was added as an internal standard and a small aliquot of the organic phase was removed for GC analysis to confirm yield. For the remaining mixture, the aqueous phase was extracted with EtOAc (2 × 3.0 mL). The combined organic phase was dried over Na_2SO_4, and the volatiles were removed to afford the crude product. Then, the mixture was purified by PTLC and the enantiomeric excess was determined by HPLC analysis.

7.4 Non-linear effect study

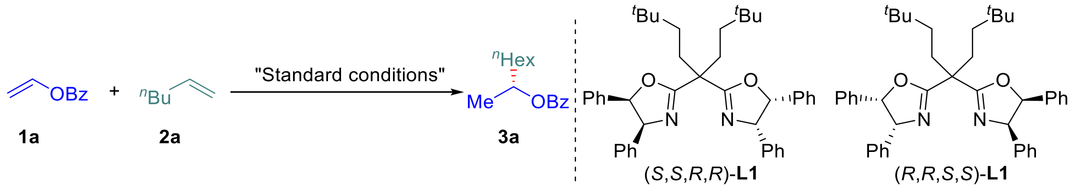

Table S11. Nonlinear effect studies.

ee (%), L1	(*S*,*S*,*R*,*R*)-L1 (mg)	(*R*,*R*,*S*,*S*)-L1 (mg)	ee (%), 3a
20	4.5	3.0	18
40	5.25	2.25	37
60	6.0	1.5	60
80	6.75	0.75	79
99	7.5	0	92

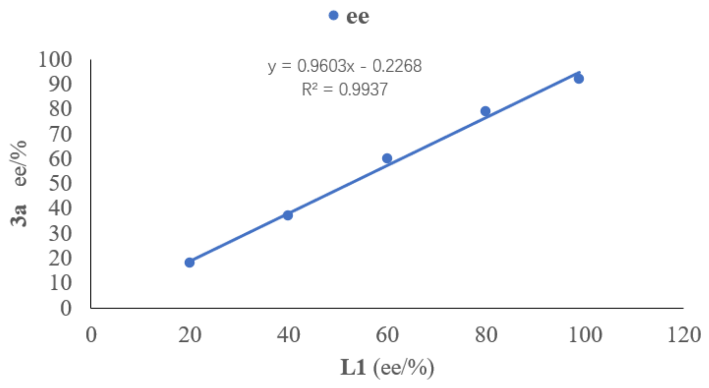

Figure S4. Non-linear effect.

In a nitrogen-filled glove-box, six 10 mL Teflon-screw cap tube containing a magnetic stir were charged with the specified amount of (*S*,*S*,*R*,*R*)-**L1** and (*R*,*R*,*S*,*S*)-**L1** to provide the enantiomeric composition of **L1** as specified in Table S9. To each of these vials $NiBr_2$·DME (3.1 mg, 0.01 mmol, 10 mol%) and anhydrous solvent (Dioxane/DCM, 3:2, 0.20 M) was added. The mixture was stirred for 5-10 min at room temperature. Then CsF/Na_2CO_3 (30.4/21.2 mg, 2.0 equiv/2.0 equiv), olefin **1a** (0.10 mmol, 1.0 equiv), **2a** (0.40 mmol, 4.0 equiv) were added. **O1** (64.8 mg, 0.40 mmol, 4.0 equiv) and phthalimide **A1** (14.7 mg, 0.1 mmol, 1.0 equiv) were added subsequently. Finally, $(MeO)_3SiH$ (38.0 uL, 0.30 mmol, 3.0 equiv) was added dropwise under N_2 atmosphere, and the reaction was stirred at 25 °C for 20 h at 1400 rpm. After the reaction was completed, the reaction mixture was diluted with saturated NH_4Cl (aqueous solution, 0.5 mL) and EtOAc (3.0 mL). The aqueous phase was extracted with EtOAc (2 × 3.0 mL). The combined organic phase was dried over Na_2SO_4, and the volatiles were removed to afford the crude product. Then, the mixture was purified by PTLC and the enantiomeric excess was determined by HPLC analysis.

7.5 Kinetic analysis of the reaction

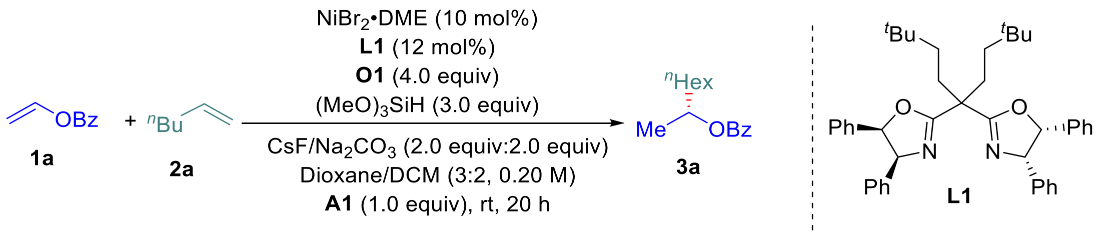

General procedure (E): To an oven-dried 10 mL Teflon-screw cap tube containing a magnetic stir was charged with $NiBr_2$·DME and **L1** in a nitrogen-filled glove-box. Subsequently, anhydrous solvent (Dioxane/DCM, 3:2, 0.20 M) was added. The mixture was stirred for 5-10 min at room temperature. Then CSF/Na_2CO_3 (60.8/42.4 mg, 2.0 equiv/2.0 equiv), olefin **1a**, **2a** were added. **O1** and phthalimide **A1** (29.4 mg, 0.2 mmol, 1.0 equiv) were added subsequently. Finally, $(MeO)_3SiH$ was added dropwise, dodecane (23.0 uL, 0.10 mmol) was added as internal standard. The tube was sealed and removed from the glove box and stirred at 25 °C. The reaction mixture was taken each time under N_2 atmosphere and quenched with H_2O immediately. The composition of mixture was analyzed by GC.

Table S12. Monitor of the reaction profile**.**

Entry	time (h)	3a (M)
1	0.2	0.008
2	0.4	0.03
3	1.2	0.042
4	2.0	0.066
5	2.4	0.084
6	3.2	0.1
7	4	0.116

8	5	0.128
9	7	0.14
10	10	0.15
11	13	0.154
12	16	0.157
13	18	0.16
14	20	0.162

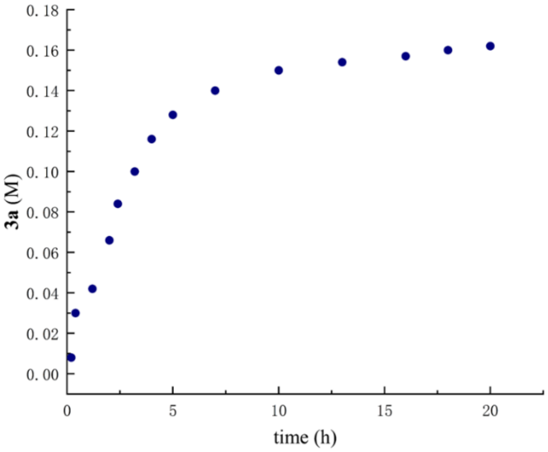

Figure S5. Monitor of the reaction profile

The rate on the concentration of 1a.

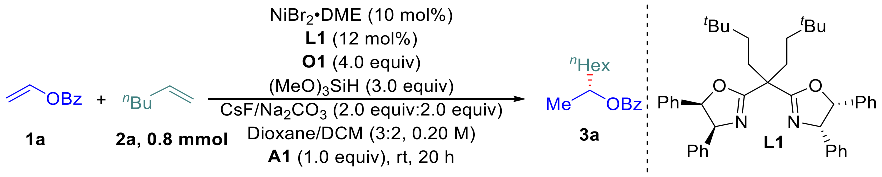

Table S13. [**1a**]₀ 0.8 eq.

Entry	Time, h	3a (M)
1	30	0.009
2	90	0.04
3	120	0.05
4	150	0.07
5	180	0.08

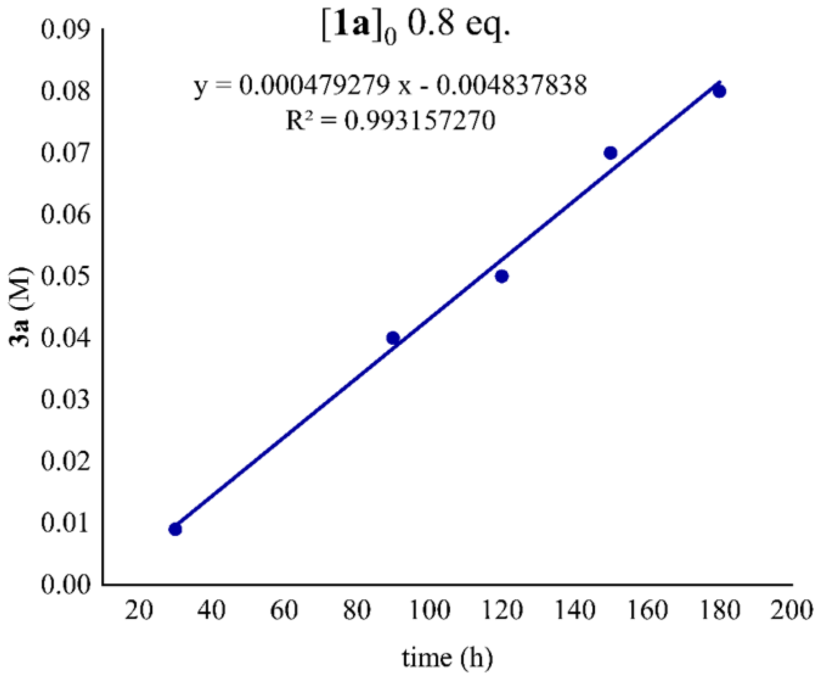

Figure S6. [**1a**]$_0$ 0.8 eq.

Table S14. [**1a**]$_0$ 1.0 eq.

**Entry**	**Time, h**	**3a (M)**
1	30	0.012
2	90	0.03
3	120	0.04
4	150	0.05
5	180	0.07

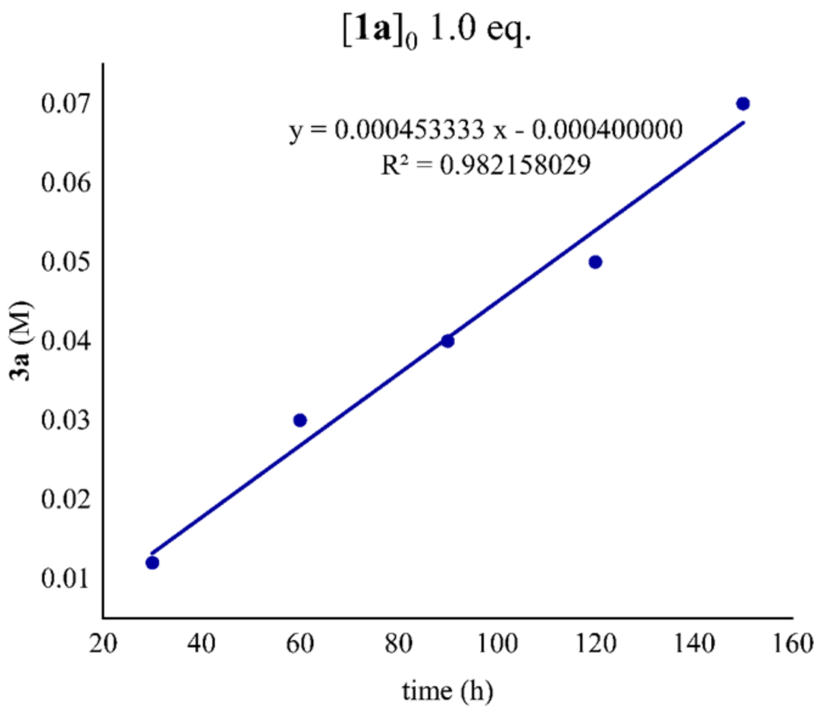

Figure S7. [**1a**]$_0$ 1.0 eq.

Table S15. [**1a**]$_0$ 1.2 eq.

Entry	Time, h	3a (M)
1	30	0.009
2	90	0.0286
3	120	0.0414
4	150	0.067
5	180	0.076

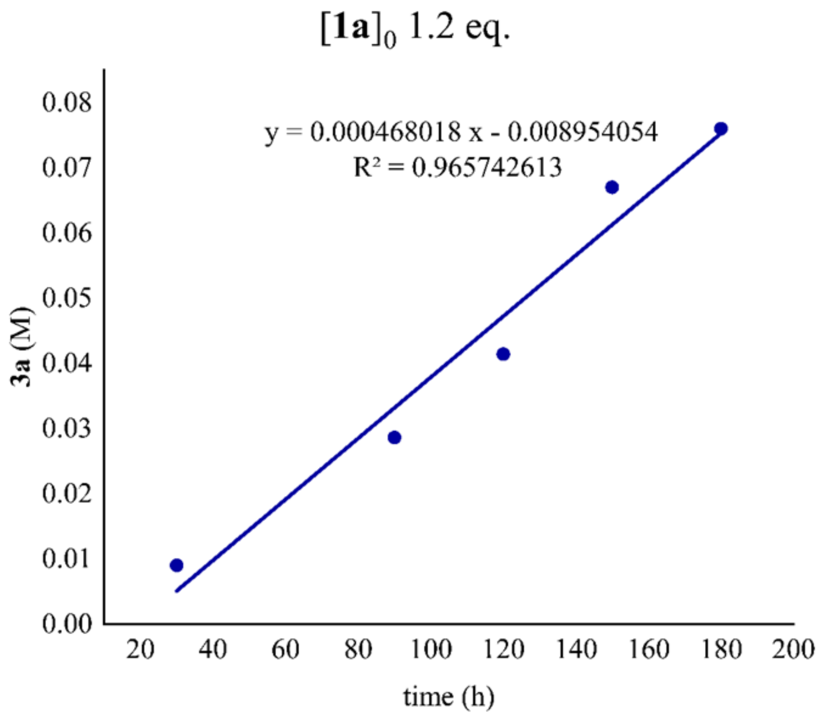

Figure S8. [**1a**]$_0$ 1.2 eq.

Table S16. [**1a**]$_0$ 1.5 eq.

Entry	Time, h	3a (M)
1	30	0.01
2	90	0.024
3	120	0.06
4	150	0.066
5	180	0.076

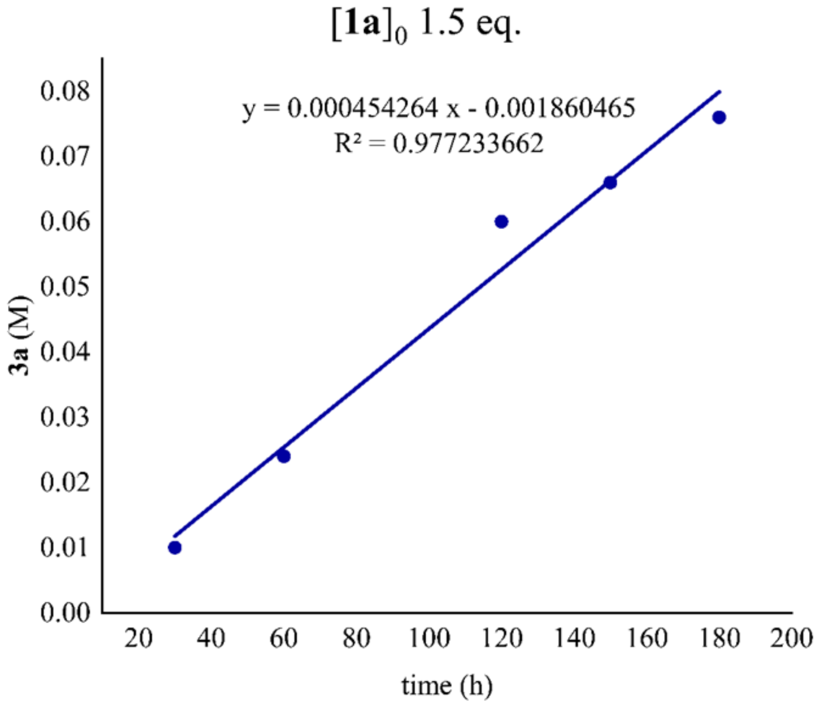

Figure S9. [**1a**]$_0$ 1.5 eq.

Table S17. [**1a**]$_0$ slope.

Entry	ln([1a]$_0$)	ln(rate)
1	-1.83258	-7.71955
2	-1.60944	-7.70694
3	-1.42712	-7.667
4	-1.20397	-7.69683

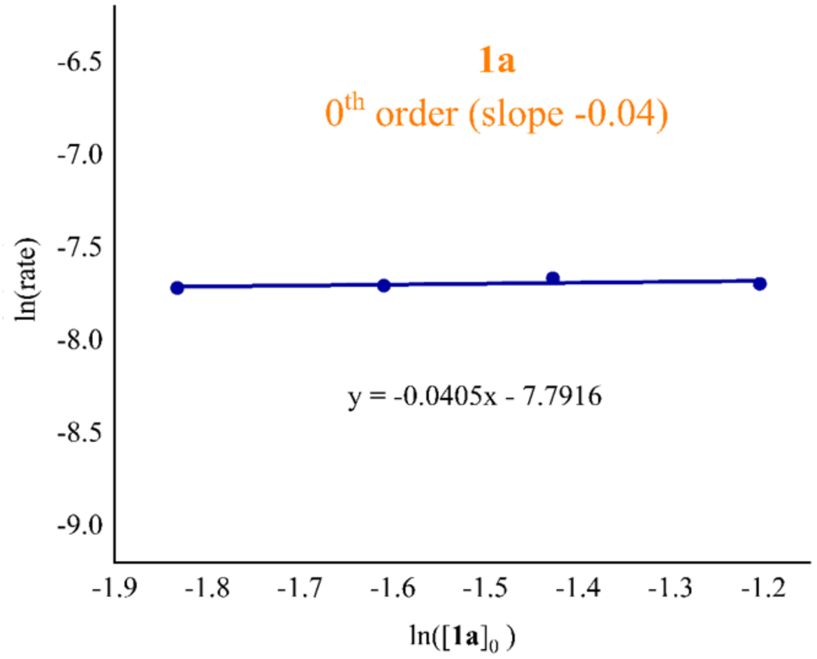

Figure S10. [**1a**]$_0$ slope.

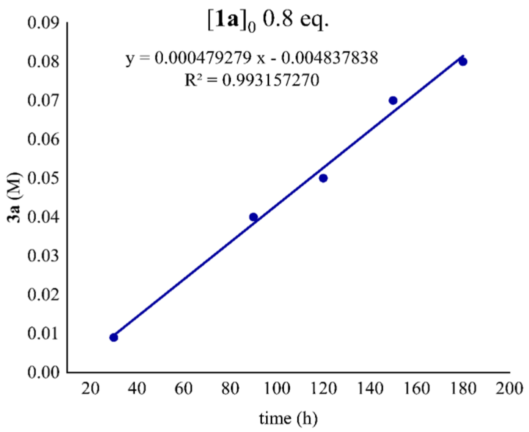

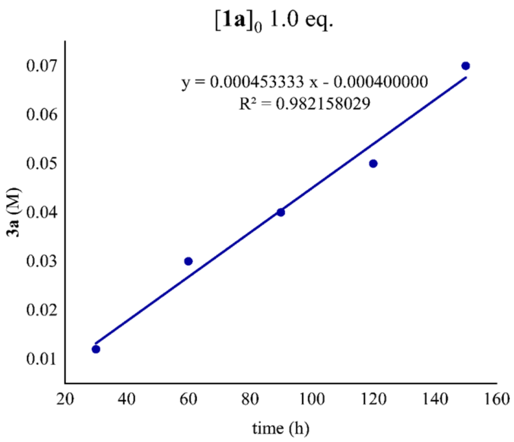

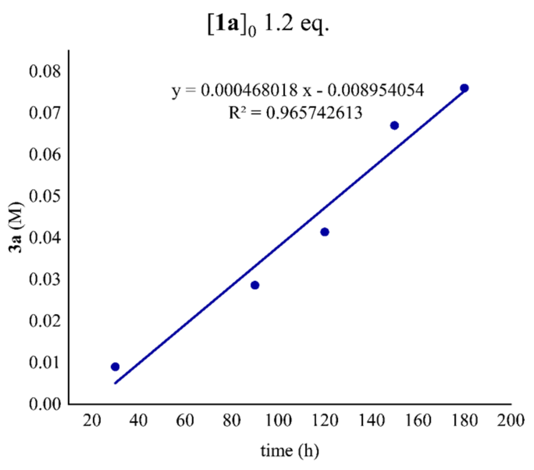

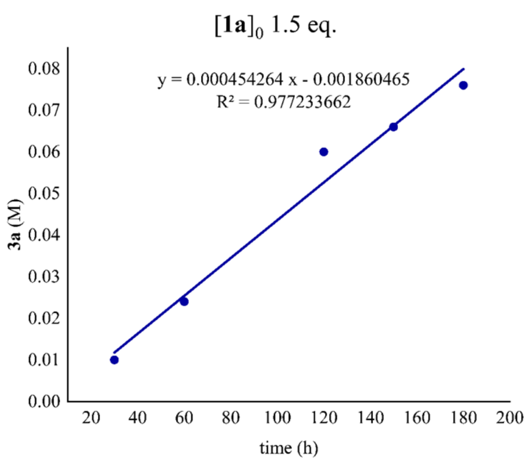

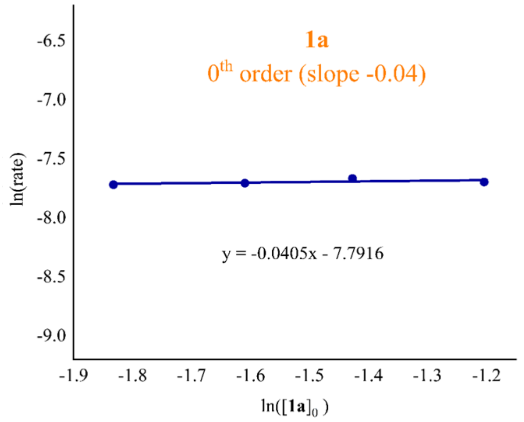

Figure S11. Rate on the concentration of **1a** from the reaction of **2a** (0.80 M), Na_2CO_3/CsF (0.40/0.40 M), $(MeO)_3SiH$ (0.60 M), $NiBr_2$·DME&**L2** (0.02 M), **O1** (0.80 M), **A1** (0.20 M) with 0.16 M, 0.20 M, 0.24 M, 0.30 M of **1a**.

The rate on the concentration of 2a

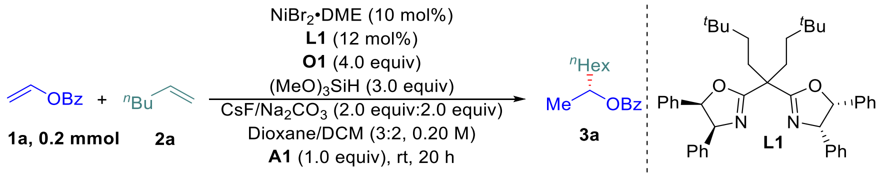

Table S18. [**2a**]$_0$ 3.5 eq**.**

Entry	Time, h	3a (M)
1	30	0.01186
2	60	0.02497
3	90	0.04304
4	120	0.05729
5	160	0.07069
6	190	0.08851

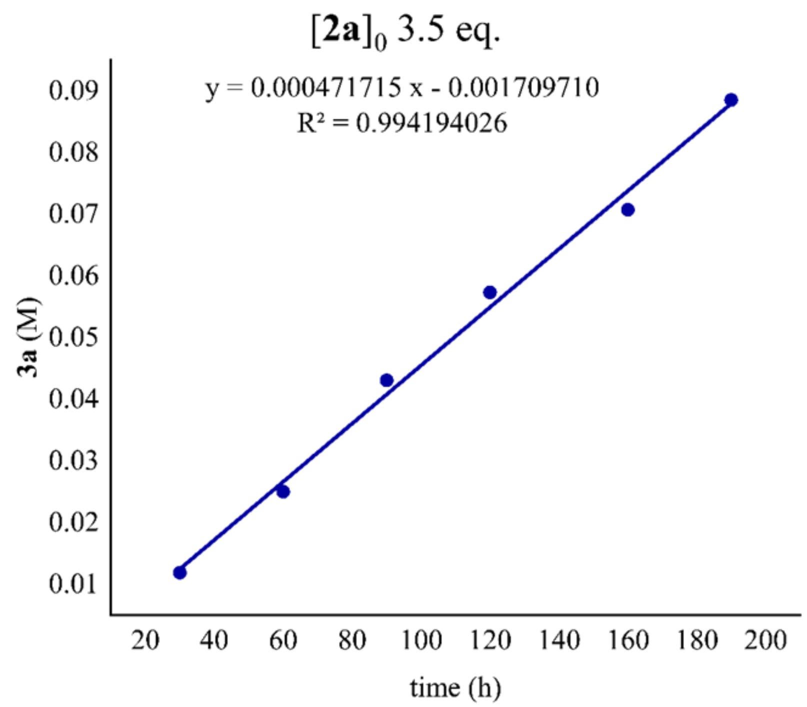

Figure S12. [**2a**]$_0$ 3.5 eq.

Table S19. [**2a**]$_0$ 4.0 eq.

Entry	Time, h	3a (M)
1	30	0.02171
2	60	0.03371
3	90	0.05771
4	120	0.07143
5	160	0.09543
6	190	0.11886

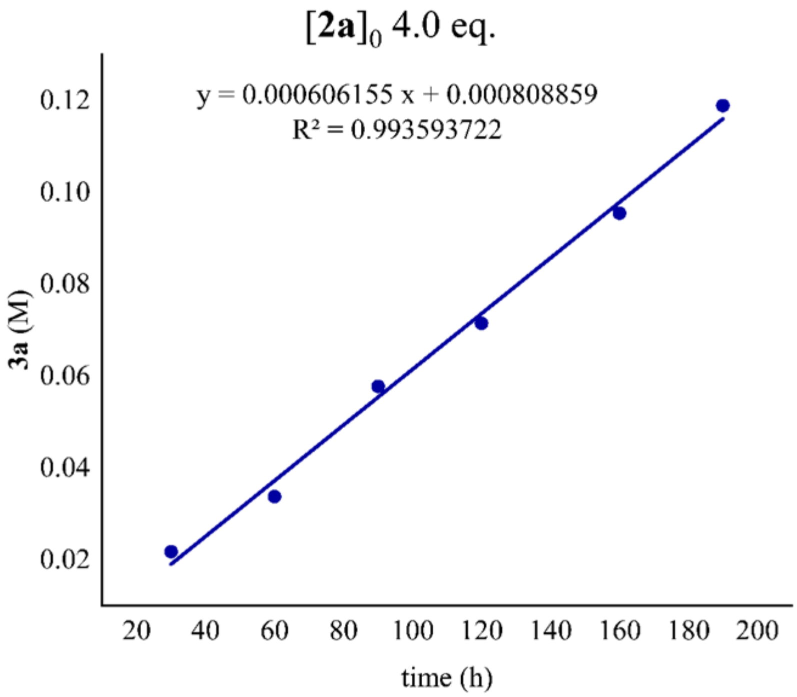

Figure S13. [**2a**]$_0$ 4.0 eq.

Table S20. [**2a**]$_0$ 4.5 eq.

Entry	Time, h	3a (M)
1	30	0.00998
2	60	0.0282
3	90	0.04631
4	120	0.06274
5	160	0.07703
6	190	0.09257

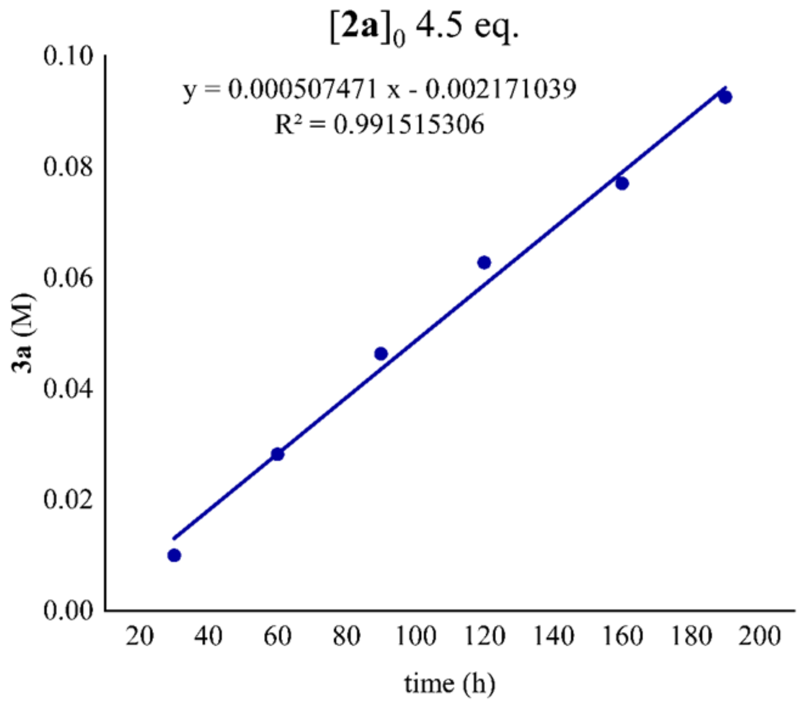

Figure S14. [**2a**]$_0$ 4.5 eq.

Table S21. [**2a**]$_0$ 5.0 eq.

Entry	Time, h	3a (M)
1	30	0.006
2	60	0.03483
3	90	0.05409
4	120	0.06674
5	160	0.08248

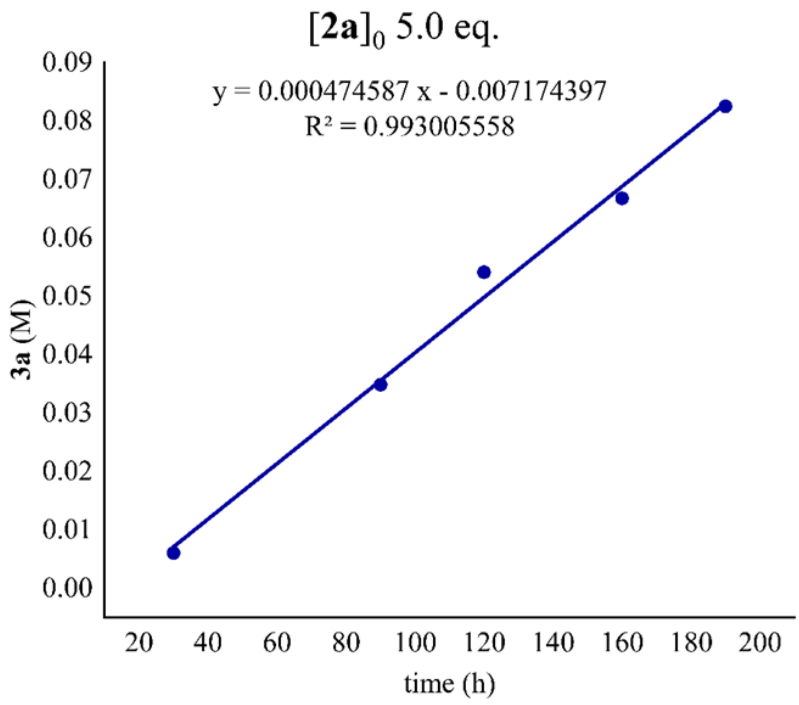

Figure S15. [2a]$_0$ 5.0 eq.

Table S22. [2a]$_0$ slope.

Entry	ln([2a]$_0$)	ln(rate)
1	-0.35667	-7.65914
2	-0.22314	-7.40837
3	-0.10536	-7.58607
4	0	-7.65307

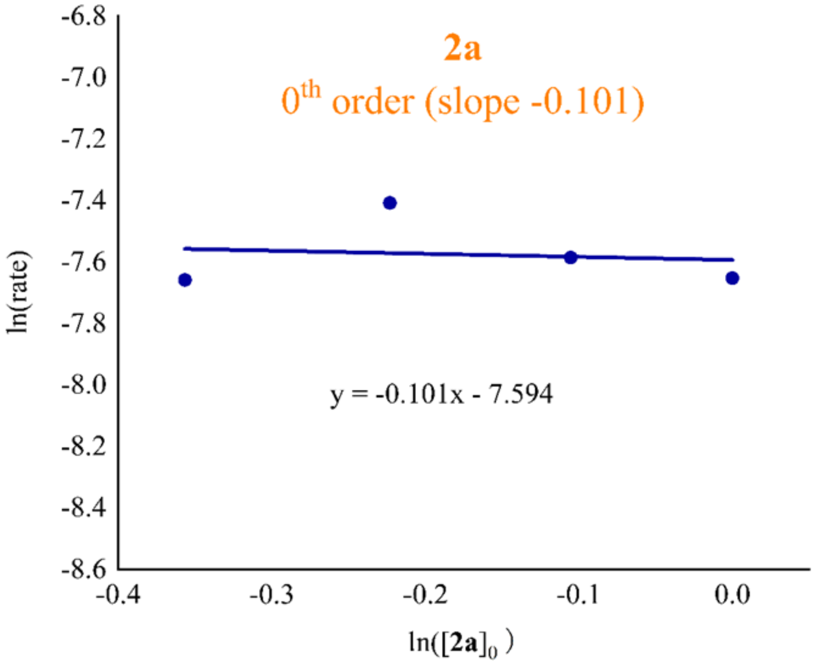

Figure S16. [**2a**]$_0$ slope.

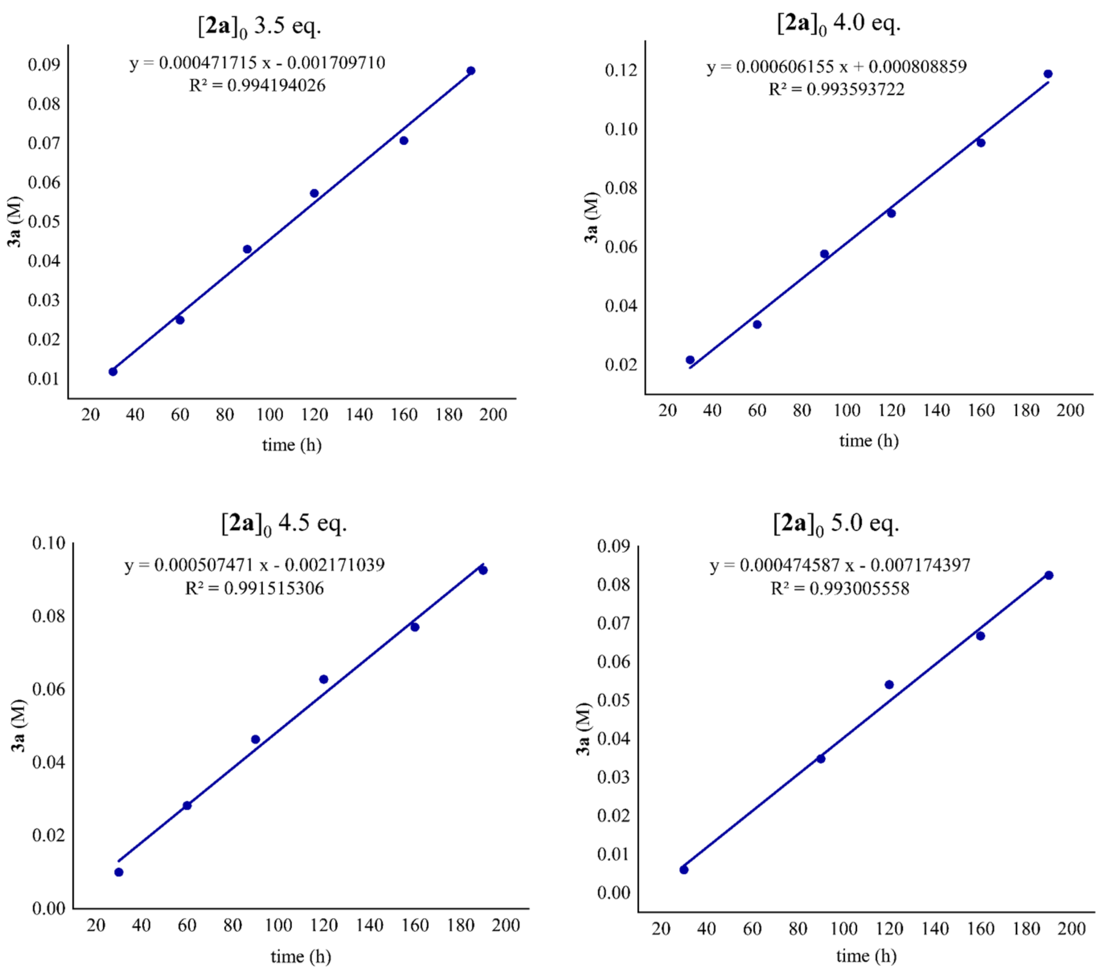

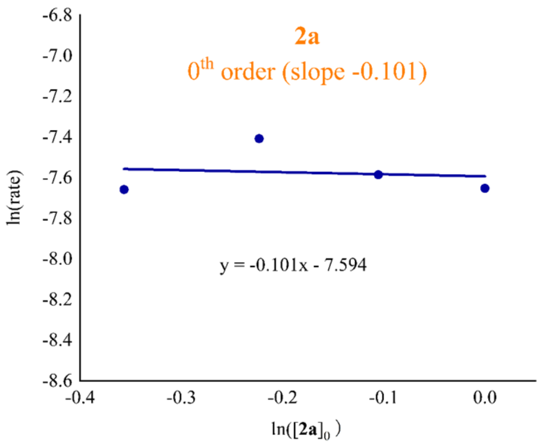

Figure S17. Rate on the concentration of **2a** from the reaction of **1a** (0.20 M), Na_2CO_3/CsF (0.40/0.40 M), $(MeO)_3SiH$ (0.60 M), $NiBr_2$·DME&**L2** (0.02 M), O1 (0.80 M), **A1** (0.20 M) with 0.70 M, 0.80 M, 0.90 M, 1.0 M of **2a.**

The rate on the concentration of $NiBr_2$·DME&L1

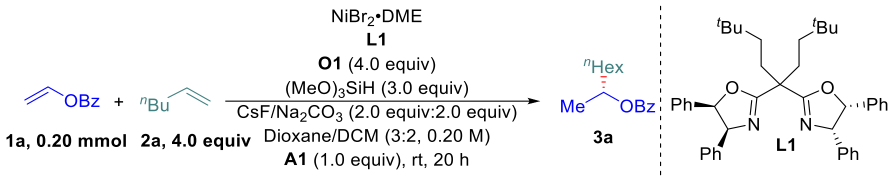

Table S23. [NiBr$_2$·DME&**L1**]$_0$ 7 mol%.

Entry	Time, h	3a (M)
1	30	0.00956
2	60	0.02209
3	90	0.03695
4	120	0.05097
5	150	0.05962
6	180	0.07767

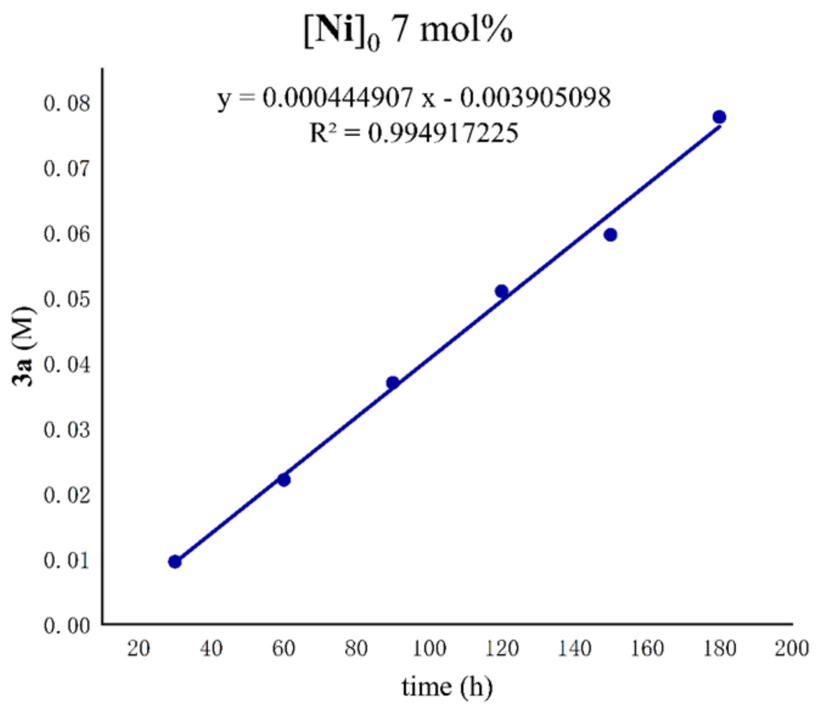

Figure S18. [NiBr$_2$·DME&L1]$_0$ 7 mol%.

Table S24. [NiBr$_2$·DME&**L1**]$_0$ 10 mol%.

Entry	Time, h	3a (M)
1	30	0.02171
2	60	0.03371
3	90	0.05771
4	120	0.07143
5	150	0.09543

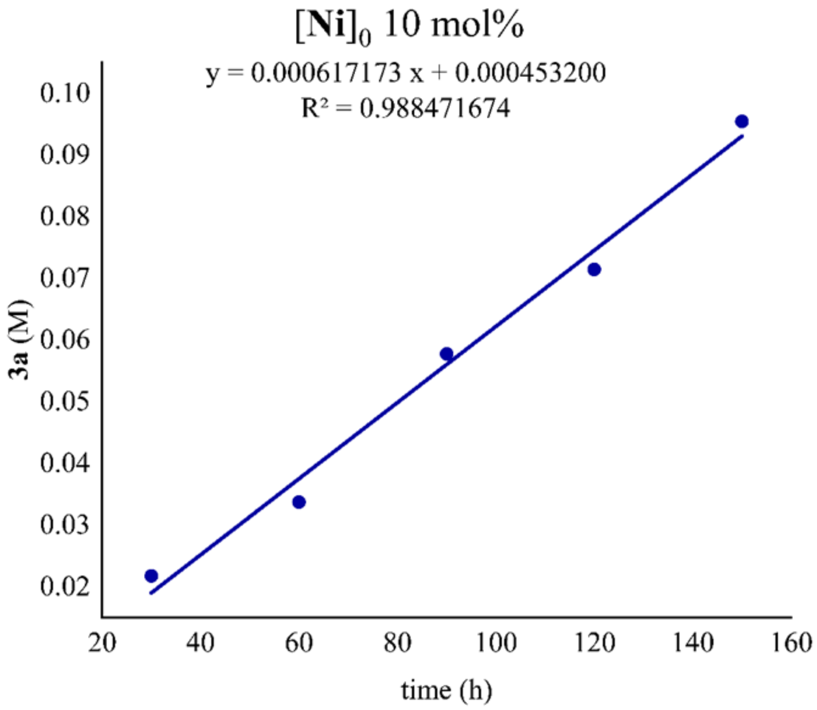

Figure S19. [$NiBr_2$·DME&L1]$_0$ 10 mol%.

Table S25. [$NiBr_2$·DME&**L1**]$_0$ 13 mol%.

**Entry**	**Time, h**	**3a (M)**
1	30	0.03371
2	90	0.07543
3	120	0.09634
4	150	0.1209

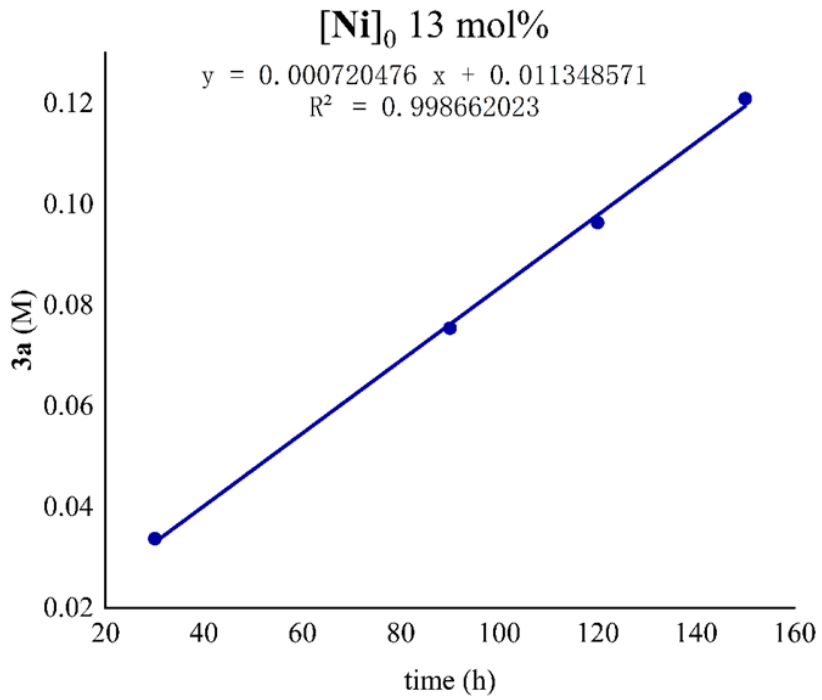

Figure S20. [NiBr2·DME&L1]0 13 mol%.

Table S26. [NiBr2·DME&**L1**]0 slope.

Entry	ln([Ni]0)	ln(rate)
1	-4.2687	-7.71765
2	-3.91202	-7.39036
3	-3.64966	-7.2356

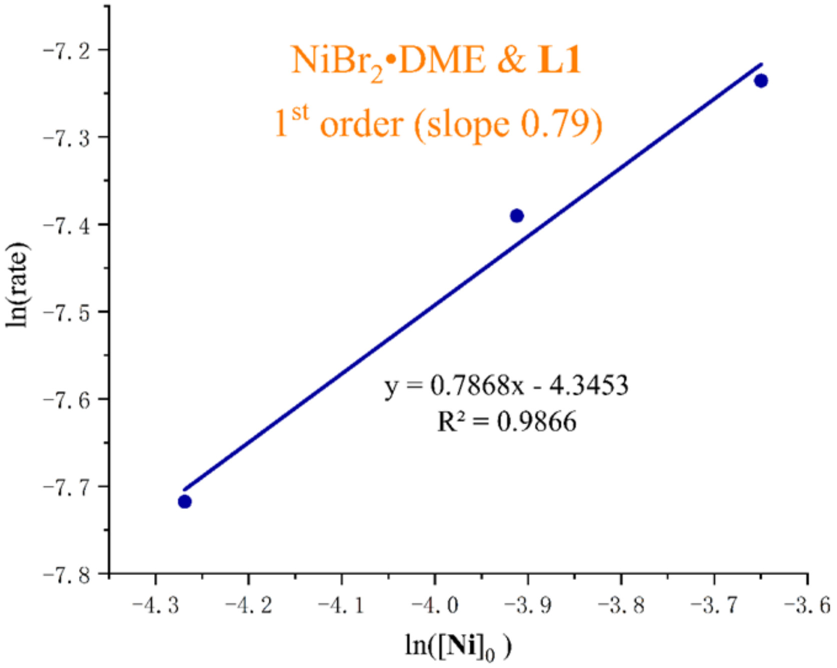

Figure S21. [NiBr$_2$·DME&L1]$_0$ slope.

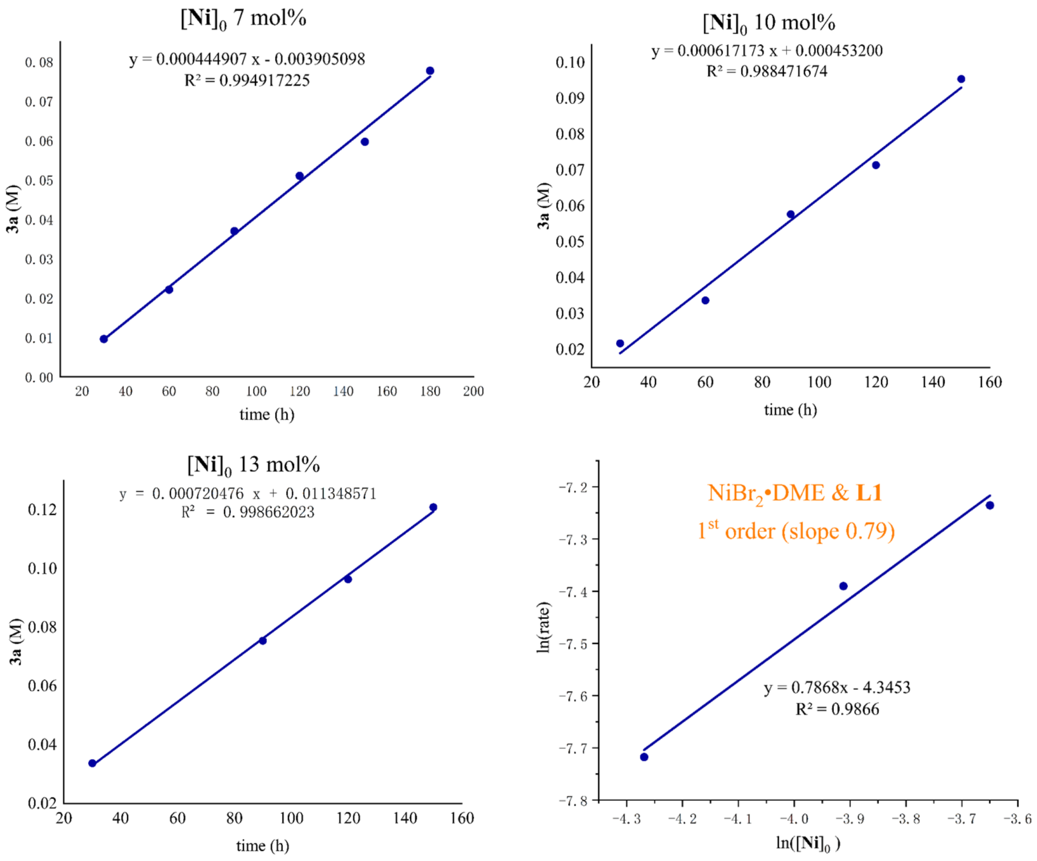

Figure S22. Rate on the concentration of NiBr$_2$·DME&**L2** from the reaction of **1a** (0.20 M), **2a** (0.80 M), Na_2CO_3/CsF (0.40/0.40 M), $(MeO)_3SiH$ (0.60 M), **O1** (0.80 M), **A1** (0.20 M) with 0.014 M, 0.020 M, 0.026 M of NiBr$_2$·DME&**L2**.

The rate on the concentration of O1.

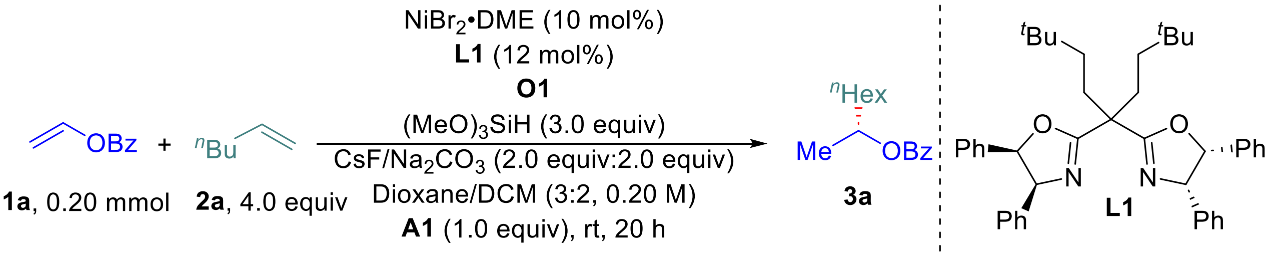

Table S27. [O1]$_0$ 3.0 eq.

Entry	Time, h	3a (M)
1	30	0.01666
2	60	0.03514
3	90	0.05086
4	120	0.07114
5	160	0.08971
6	190	0.10114

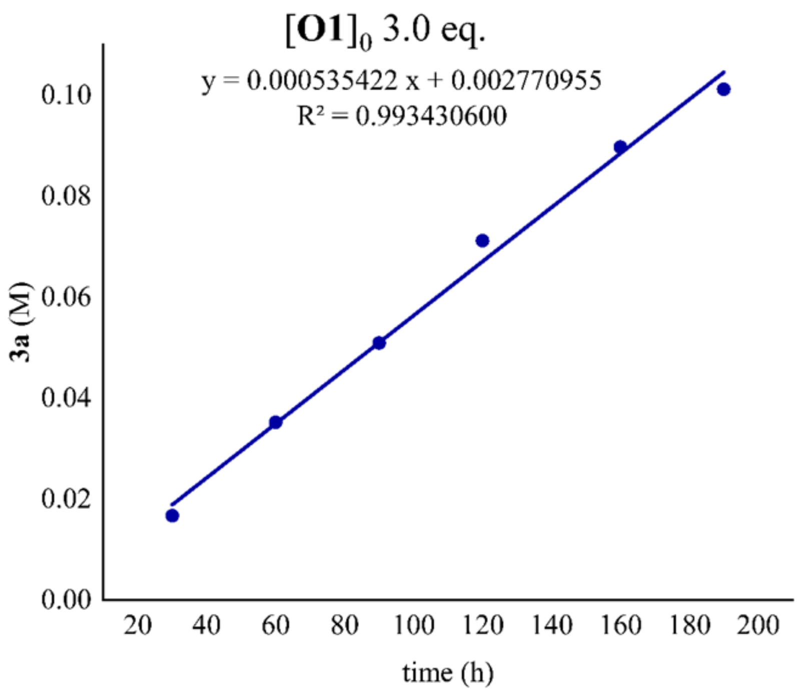

Figure S23. $[\mathbf{O1}]_0$ 3.0 eq.

Table S28. $[\mathbf{O1}]_0$ 3.5 eq.

**Entry**	**Time, h**	**3a (M)**
1	60	0.032
2	90	0.05286
3	120	0.06943
4	160	0.09286

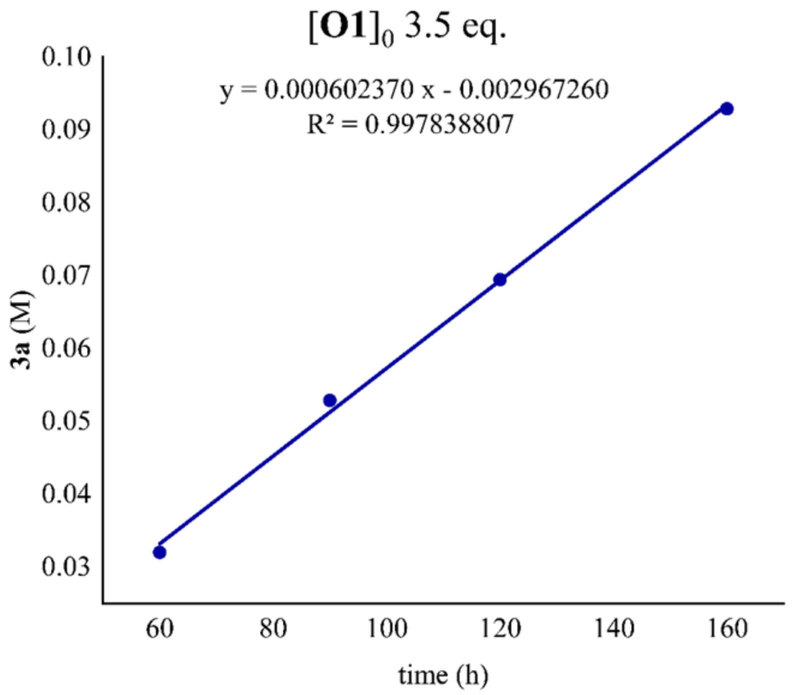

Figure S24. [O1]$_0$ 3.5 eq.

Table S29. [**O1**]$_0$ 4.0 eq**.**

Entry	Time, h	3a (M)
1	30	0.02171
2	60	0.03371
3	90	0.05771
4	120	0.07143
5	160	0.09543
6	190	0.11886

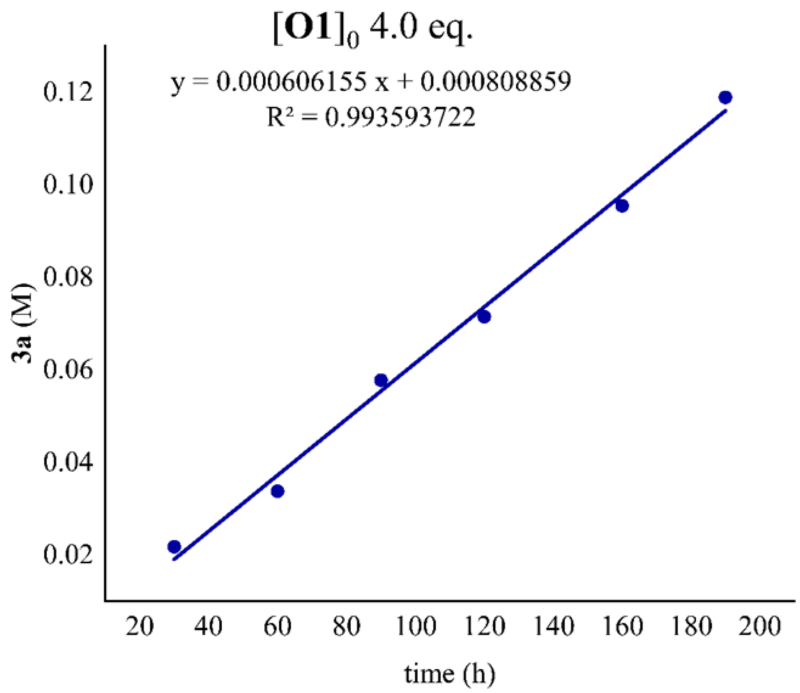

Figure S25. [**O1**]$_0$ 4.0 eq.

Table S30. [**O1**]$_0$ 4.5 eq.

Entry	Time, h	3a (M)
1	30	0.01997
2	60	0.03686
3	120	0.07286
4	160	0.096
5	190	0.1037

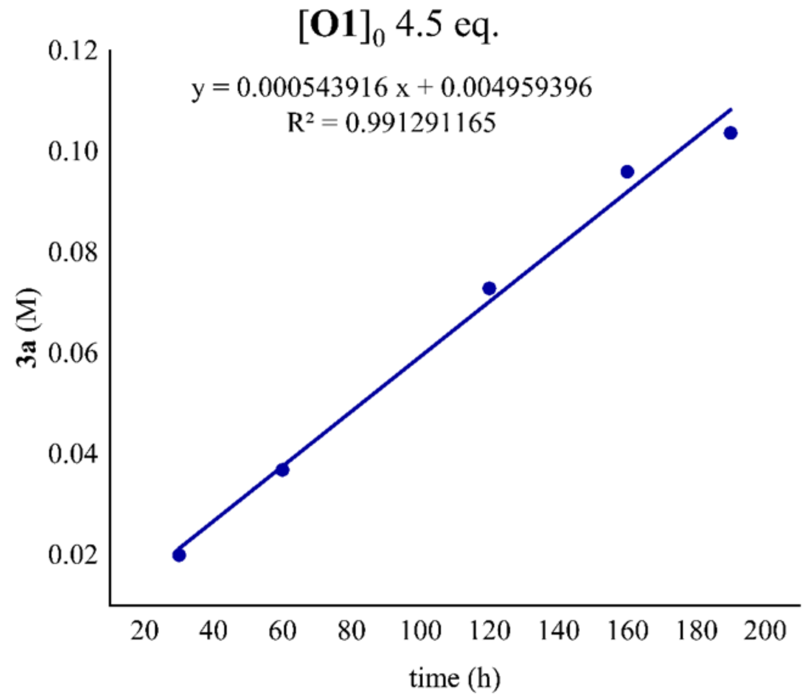

Figure S26. [O1]$_0$ 4.5 eq.

Table S31. [**O1**]$_0$ slope.

Entry	ln([O1]$_0$)	ln(rate)
1	-0.51083	-7.53246
2	-0.35667	-7.41464
3	-0.22314	-7.40837
4	-0.10536	-7.51672

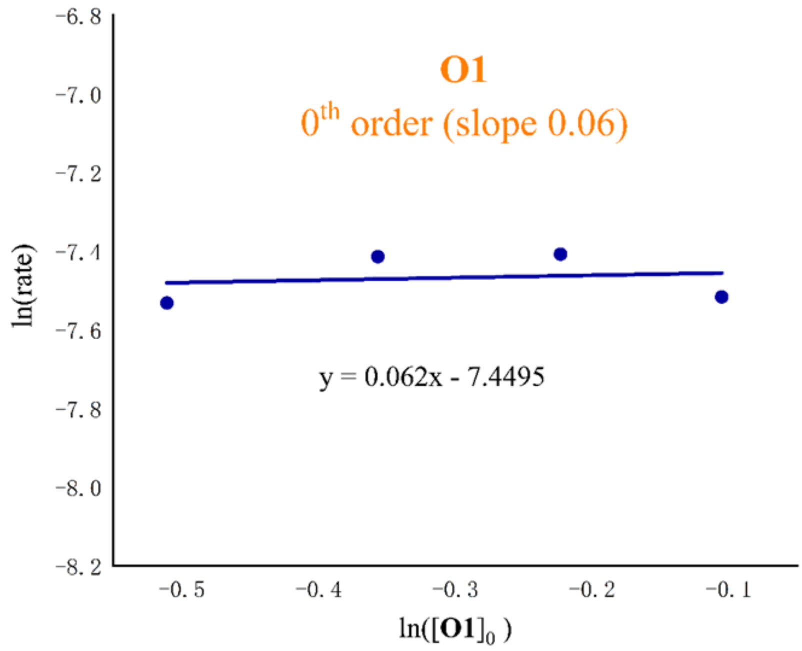

Figure S27. [**O1**]$_0$ slope.

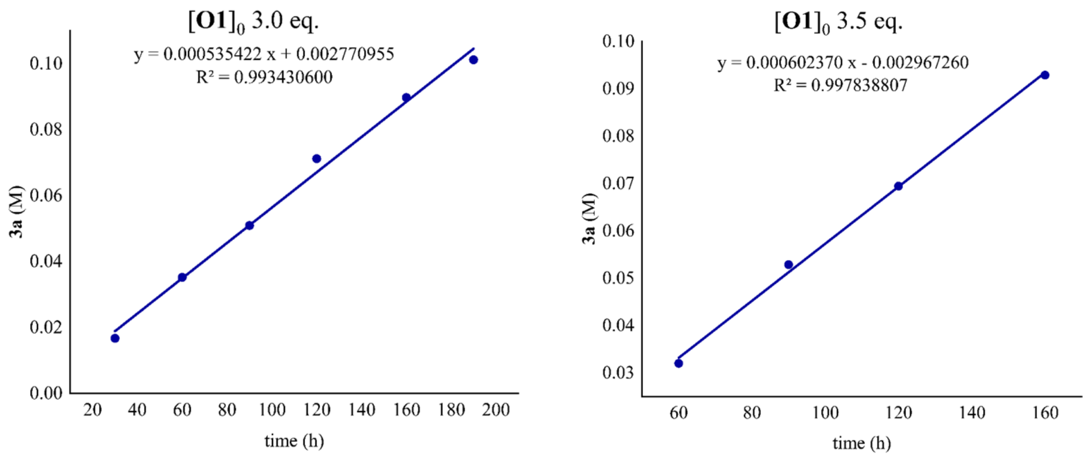

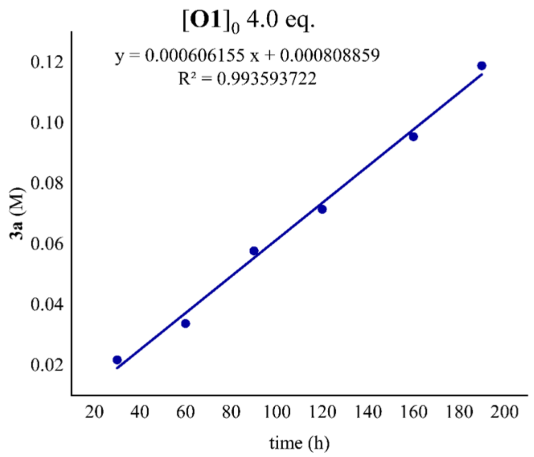

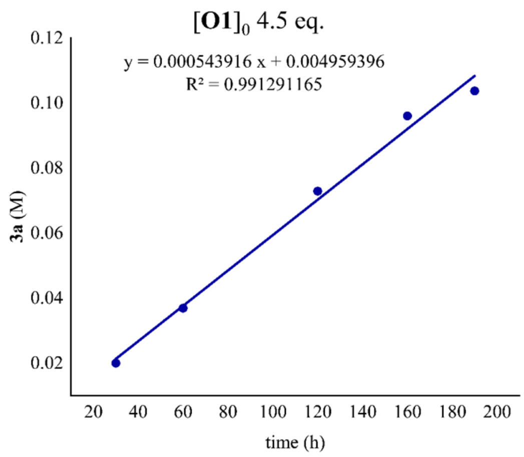

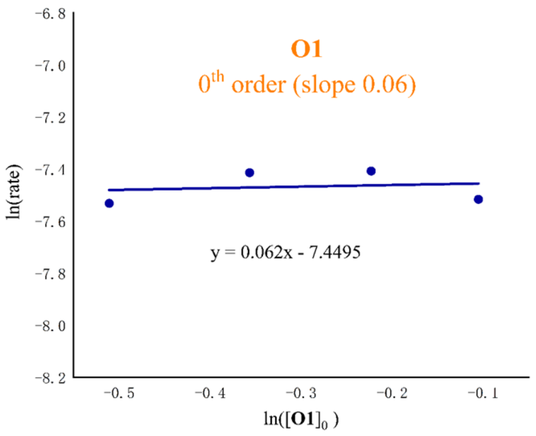

Figure S28. Rate on the concentration of **O1** from the reaction of **1a** (0.20 M), **2a** (0.80 M), Na_2CO_3/CsF (0.40/0.40 M), $(MeO)_3SiH$ (0.60 M), $NiBr_2$·DME&**L2** (0.02 M), **A1** (0.20 M) with 0.60 M, 0.7 M, 0.80 M, 0.90 M of **O1**.

The rate on the concentration of $(MeO)_3SiH$.

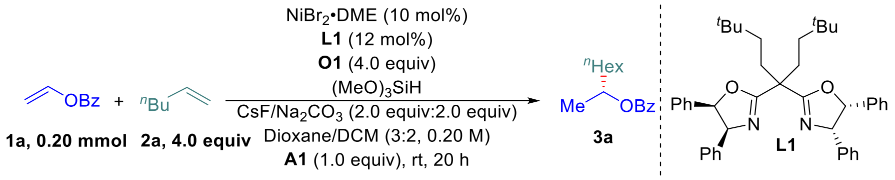

Table S32. $[(MeO)_3SiH]_0$ 2.6 eq.

Entry	Time, h	3a (M)
1	60	0.02556
2	90	0.03952
3	120	0.05742
4	150	0.06835
5	180	0.08251

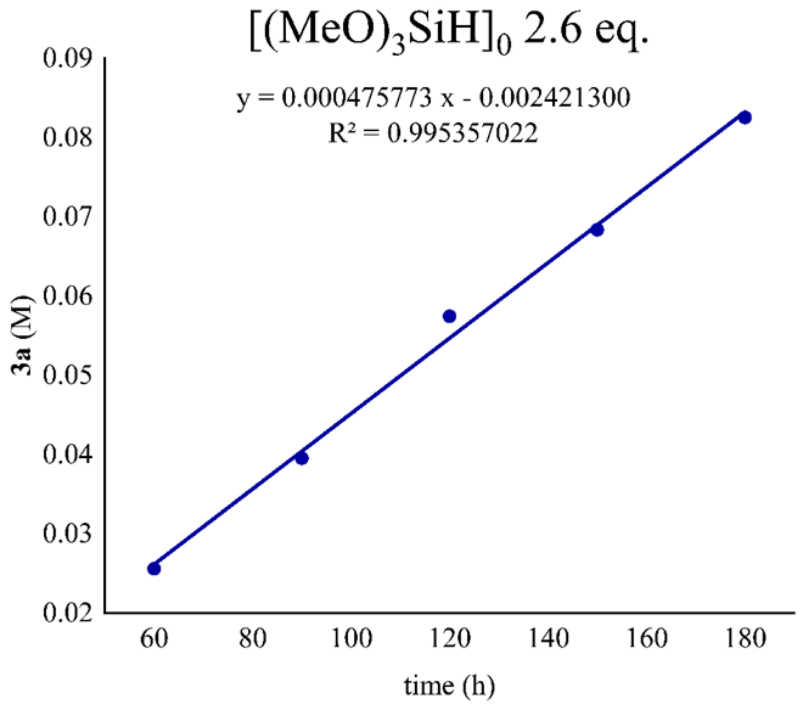

Figure S29. [(MeO)$_3$SiH]$_0$ 2.6 equiv.

Table S33. [(MeO)$_3$SiH]$_0$ 3.0 eq.

Entry	Time, h	3a (M)
1	30	0.02171
2	60	0.03371
3	90	0.05771
4	120	0.07143
5	150	0.08947

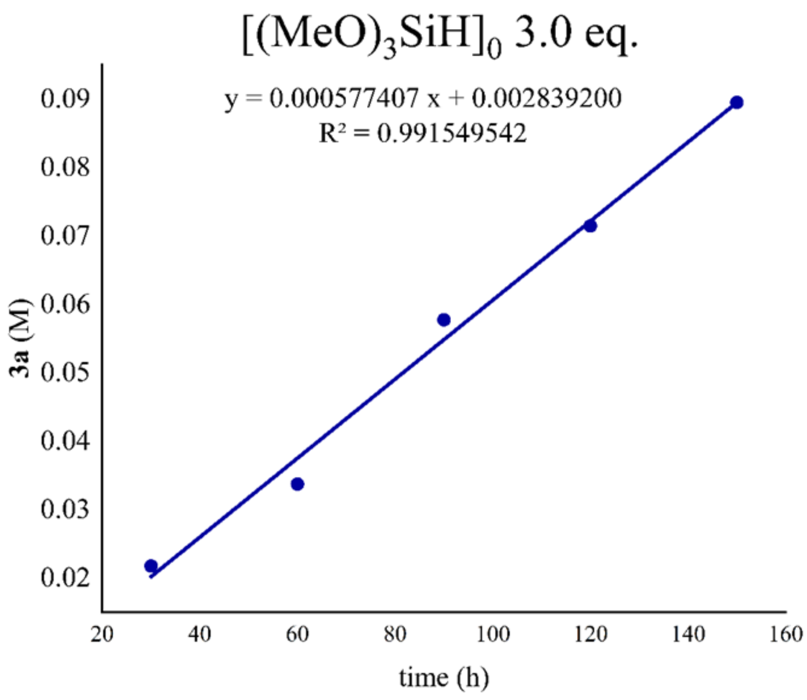

Figure S30. $[(MeO)_3SiH]_0$ 3.0 equiv.

Table S34. $[(MeO)_3SiH]_0$ 3.4 eq.

Entry	Time, h	3a (M)
1	30	0.014
2	60	0.03657
3	90	0.05363
4	120	0.06881
5	150	0.07863
6	180	0.10215

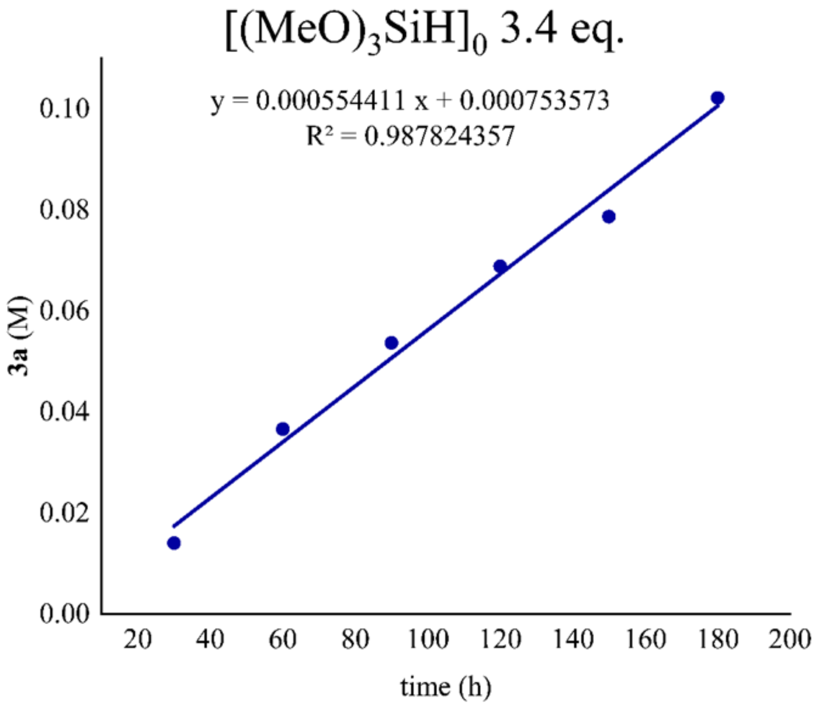

Figure S31. $[(MeO)_3SiH]_0$ 3.4 eq.

Table S35. $[(MeO)_3SiH]_0$ 3.8 eq.

Entry	Time, h	3a (M)
1	60	0.027
2	90	0.04279
3	120	0.05888
4	150	0.07049
5	180	0.08245

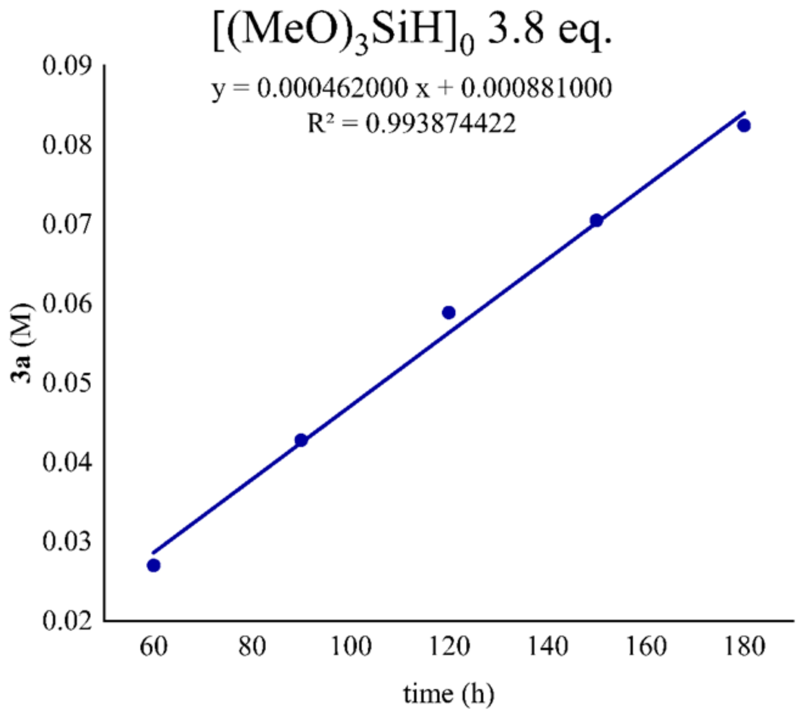

Figure S32. $[(MeO)_3SiH]_0$ 3.8 eq.

Table S36. $[(MeO)_3SiH]_0$ slope.

Entry	$\ln([(MeO)_3SiH]_0)$	ln(rate)
1	-0.65393	-7.65057
2	-0.51083	-7.45696
3	-0.38566	-7.4976
4	-0.27444	-7.67995

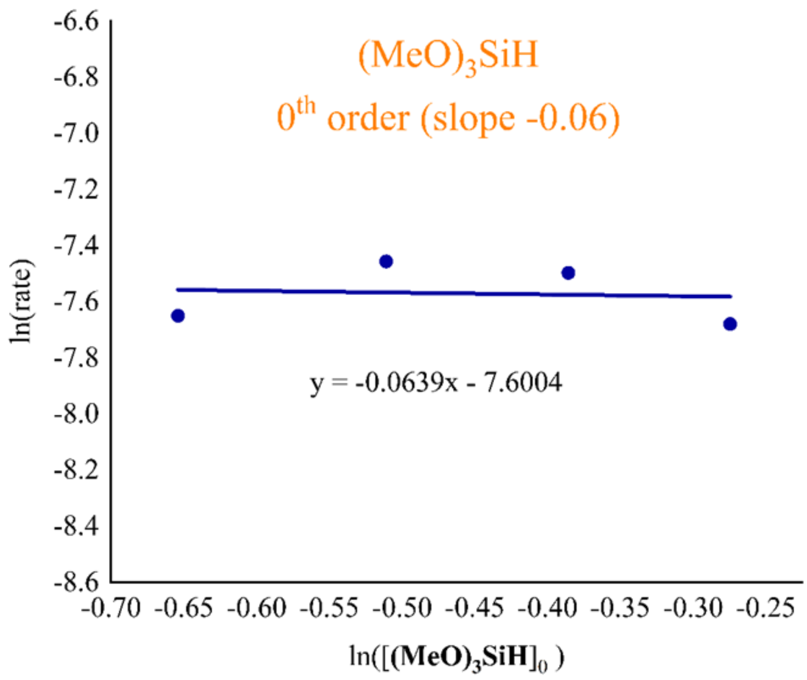

Figure S33. [(MeO)3SiH] slpoe.

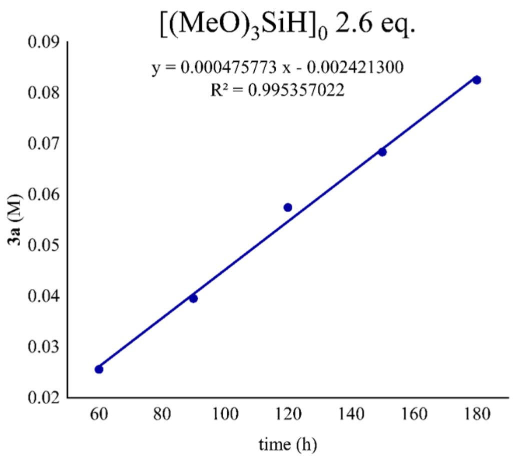

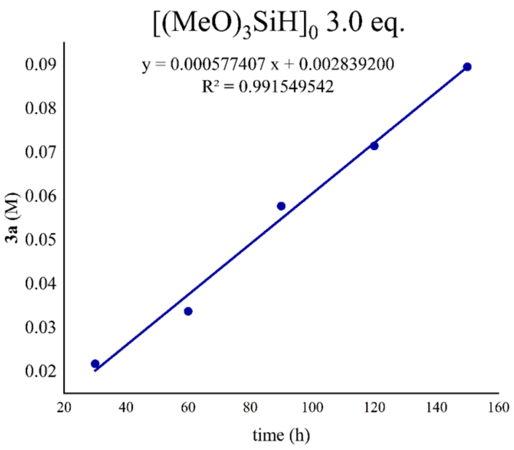

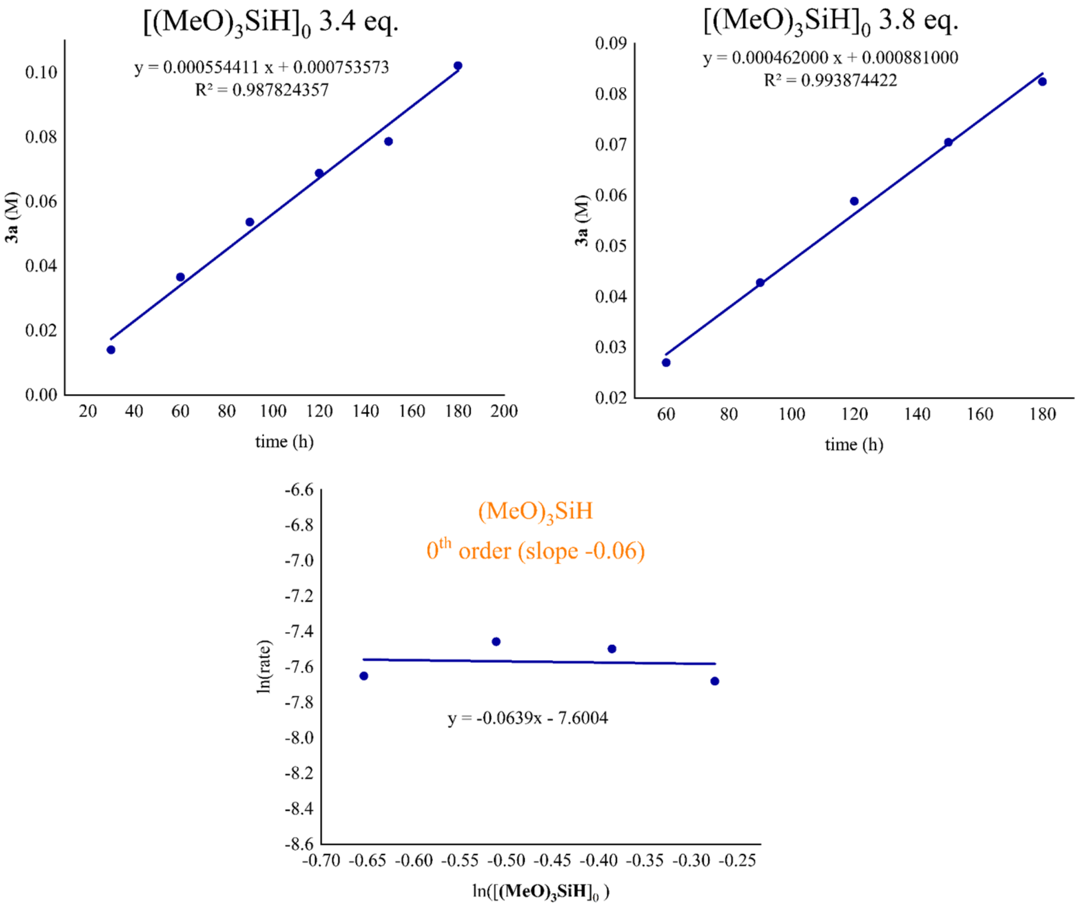

Figure S34. Rate on the concentration of $(MeO)_3SiH$ from the reaction of **1a** (0.20 M), **2a** (0.80 M), Na_2CO_3/CsF (0.40/0.40 M), **O1** (0.80 M), $NiBr_2$·DME&**L2** (0.02 M), **A1** (0.20 M) with 0.52 M, 0.60 M, 0.68 M, 0.76 M of $(MeO)_3SiH$**.**

IX. References

1. (a) Mou, X.-Q.; Rong, F.-M.; Zhang, H.; Chen, G.; He, G. *Org. Lett.* **2019**, *21*, 4657−4661. (b) Qiao, J.-B.; Zhao, Y.-M.; Gu, P. *Org. Lett.* **2016**, *18*, 1984−1987.

2. Bovino, M. T.; Chemler, S. R. *Angew. Chem., Int. Ed.* **2012**, *51*, 3923−3927.

3. Wong, A.; Welch, C. J.; Kuethe, J. T.; Vazquez, E.; Shaimi, M.; Henderson, D.; Davies, I. W.; Hughes, D. L. *Org. Biomol. Chem.* **2004**, *2*, 168−174.

4. Wu, L.; Shao, Q.; Kong, L.; Chen, J.; Wei, Q.; Zhang, W. *Org. Chem. Front.* **2020**, *7*, 862−867.

5. Li, Q.; Zhang, M.; Zhan, S.; Gu, Z. *Org. Lett.* **2019**, *21*, 6374−6377.

6. Zhang, G.; Zhou, S.; Fu, L.; Chen, P.; Li, Y.; Zou, J.; Liu, G. *Angew. Chem., Int. Ed.* **2020**, *59*, 20439−20444.

7. Martínez-Montero, S.; Fernández, S.; Sanghvi, Y. S.; Gotor, V.; Ferrero, M. *Org. Biomol. Chem.* **2011**, *9*, 5960−5966.

8. Deng, W.; Hu, Y.; Hu, J.; Li, X.; Li, Y.; Huang, Y. *Chem. Commun.* **2022**, *58*, 12094−12097.
9. Jiang, R.; Chen, Z.; Zhan, K.; Liu, L.; Zhou, J.; Ai, Y.; Li, S.; Bao, H.; Hu, Z. n.; Qi, L.; Wang, J.; Sun, H.-B. *Tetrahedron Lett.* **2018**, *59*, 3279−3282.
10. Goossen, L. J.; Paetzold, J.; Koley, D. *Chem. Commun.* **2003**, 706−707.
11. Den Reijer, C. J.; Drago, D.; Pregosin, P. S. *Organometallics* **2001**, *20*, 2982−2989.
12. Lumbroso, A.; Vautravers, N. R.; Breit, B. *Org. Lett.* **2010**, *12*, 5498−5501.
13. Nicks, F.; Libert, L.; Delaude, L.; Demonceau, A. *Aust. J. Chem.* **2009**, *62*, 227−231.
14. Abrams, M. L.; Foarta, F.; Landis, C. R. *J. Am. Chem. Soc.* **2014**, *136*, 14583–14588.
15. Li, G.; Wang, L.; Wu, L.; Guo, Z.; Zhao, J.; Liu, Y.; Bai, R.; Yan, X. *J. Am. Chem. Soc.* **2020**, *142*, 14343−14349.
16. Yang, S.; Hu, H.; Chen, M. *Org. Lett.* **2023**, *25*, 7968−7973.
17. Myers, A. G.; Lanman, B. A. *J. Am. Chem. Soc.* **2002**, *124*, 12969−12971.
18. Shigekane, M.; Arai, T.; Tamura, M.; Uchida, T.; Kakiuchi, F.; Kochi, T. *Tetrahedron Lett.* **2023**, *114*, 154292.
19. Erchinger, J. E.; Hoogesteger, R.; Laskar, R.; Dutta, S.; Hümpel, C.; Rana, D.; Daniliuc, C. G.; Glorius, F. *J. Am. Chem. Soc.* **2023**, *145*, 2364−2374.
20. Banerjee, A.; Sarkar, S.; Shah, J. A.; Frederiks, N. C.; Bazan-Bergamino, E. A.; Johnson, C. J.; Ngai, M.-Y. *Angew. Chem., Int. Ed.* **2022**, *61*, e202113841.
21. Zhang, Z.-K.; Feng, Y.-L.; Ruan, Z.; Xu, Y.-Q.; Cao, Z.-Y.; Li, M.-H.; Wang, C. *Chem. Commun.* **2022**, *58*, 11709−11712.
22. Garland, A. D.; Bryant, G. C.; Chambrier, I.; Cammidge, A. N.; Cook, M. J. *J. Porphyr. Phthalocyanines* **2014**, *18*, 944−949.
23. Liu, Y.; Wu, H.; Guo, Y.; Xiao, J.-C.; Chen, Q.-Y.; Liu, C. *Angew. Chem., Int. Ed.* **2017**, *56*, 15432−15435.
24. Wang, G.-Z.; Shang, R.; Fu, Y. *Org. Lett.* **2018**, *20*, 888−891.
25. Liu, X.; Liu, B.; Liu, Q. *Angew. Chem., Int. Ed.* **2020**, *59*, 6750−6755.
26. Haydl, A. M.; Hartwig, J. F. *Org. Lett.* **2019**, *21*, 1337−1341.
27. Nguyen, T. N. T.; Thiel, N. O.; Pape, F.; Teichert, J. F. *Org. Lett.* **2016**, *18*, 2455−2458.
28. Xu, L.; Liu, Z.; Dong, W.; Song, J.; Miao, M.; Xu, J.; Ren, H. *Org. Biomol. Chem.* **2015**, *13*, 6333−6337.
29. Doyle, M. G. J.; Gabbey, A. L.; McNutt, W.; Lundgren, R. J. *Angew. Chem., Int. Ed.* **2021**, *60*, 26495−26499.
30. Franchino, A.; Martí, À.; Echavarren, A. M. *J. Am. Chem. Soc.* **2022**, *144*, 3497−3509.
31. (a) Watanabe, T.; Kurata, I.; Hayashi, C.; Igarashi, M.; Sawa, R.; Takahashi, Y.; Akamatsu, Y. *Bioorg. Med. Chem. Lett.* **2010**, *20*, 5843−5846. (b) Jones, G. B.; Huber, R. S.; Chapman, B. J. *Tetrahedron: Asymmetry* **1997**, *8*, 1797−1809.
32. Watanabe, T.; Kurata, I.; Hayashi, C.; Igarashi, M.; Sawa, R.; Takahashi, Y.; Akamatsu, Y. *Bioorg. Med. Chem. Lett.* **2010**, *20*, 5843−5846.
33. Yang, Z.-P.; Fu, G. C. *J. Am. Chem. Soc.* **2020**, *142*, 5870−5875.

34. Bracher, F.; Schulte, B. *Nat. Prod. Lett.* **1995**, *7*, 65−68.
35. Durand, T.; Henry, O.; Guy, A.; Roland, A.; Vidal, J.-P.; Rossi, J.-C. *Tetrahedron* **2003**, *59*, 2485−2495.
36. Ngooi, T. K.; Scilimati, A.; Guo, Z. W.; Sih, C. J. *J. Org. Chem.* **1989**, *54*, 911−914.
37. Parra, M. L.; Saavedra, C. G.; Hidalgo, P. I.; Elgueta, E. Y. *Liq. Cryst.* **2008**, *35*, 55−64.
38. Yang, X.; Li, X.; Chen, P.; Liu, G. *J. Am. Chem. Soc.* **2022**, *144*, 7972−7977.
39. Fianu, G. D.; Schipper, K. C.; Flowers Ii, R. A. *Catal. Sci. Technol.* **2017**, *7*, 3469−3473.
40. Tian, Y.; Liu, Z.-Q. *Green Chem.* **2017**, *19*, 5230−5235.
41. Zhang, Q.; Wang, S.; Zhang, Q.; Xiong, T.; Zhang, Q. *ACS Catal.* **2022**, *12*, 527−535.
42. Kumar, S.; Patel, M.; Verma, A. K. *Asian J. Org. Chem.* **2021**, *10*, 2365−2369.

X. ^{1}H NMR, ^{13}C NMR and ^{19}F NMR Spectra

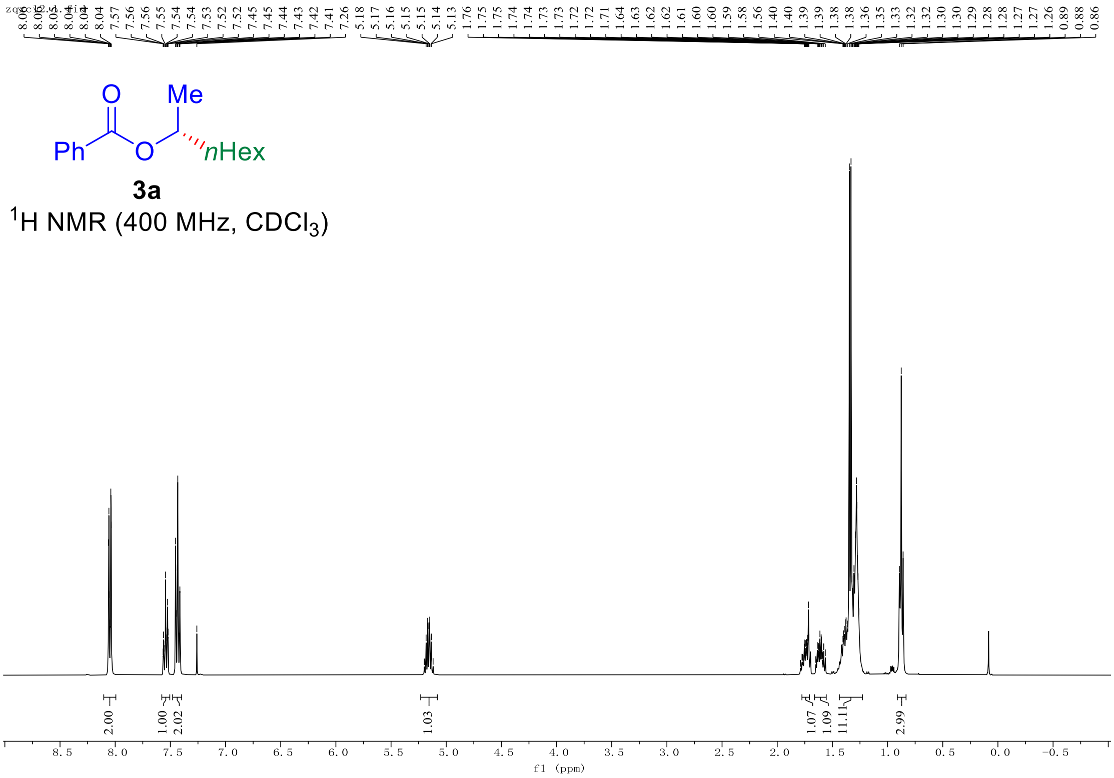

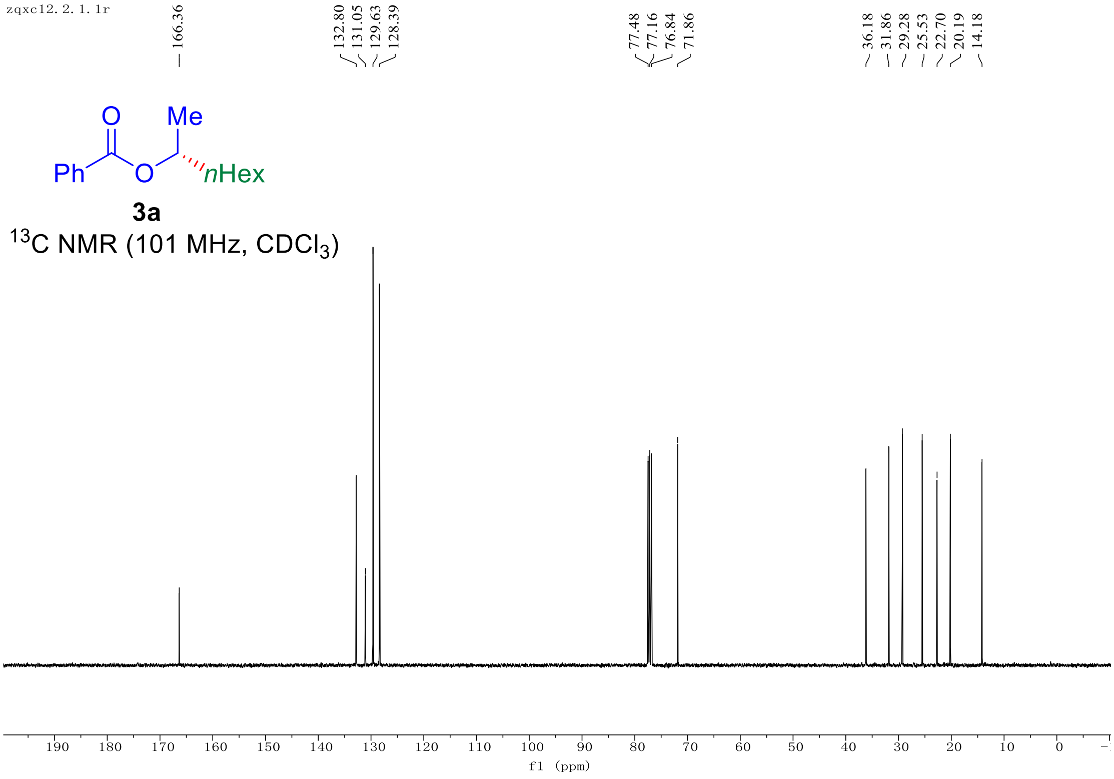

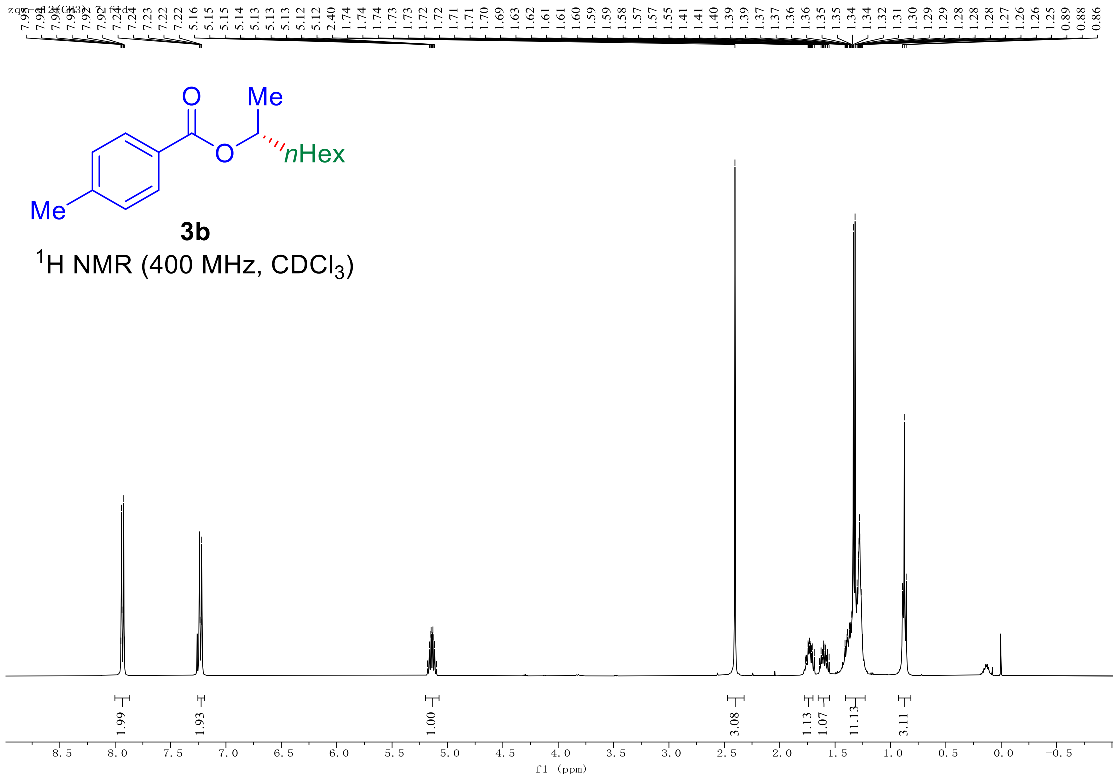
Me
O
O
nHex
Me
3b
1H NMR (400 MHz, CDCl3)
1.99
1.93
1.00
3.08
1.13
1.07
11.13
3.11
f1 (ppm)

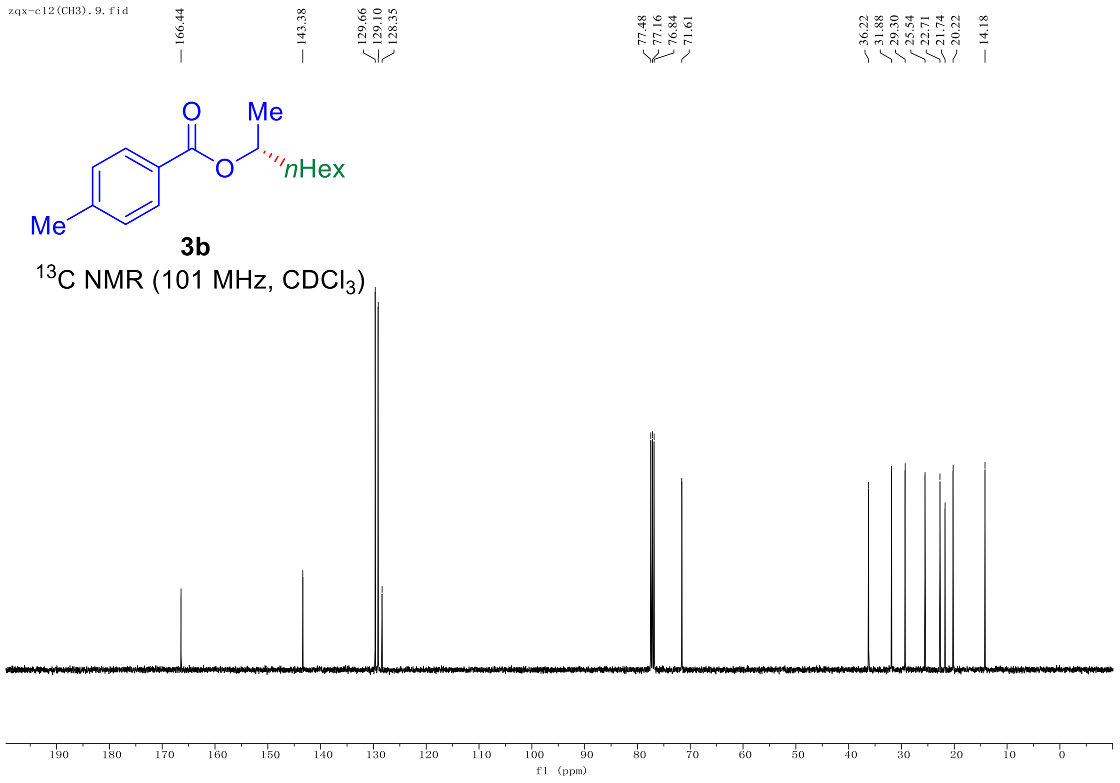
zqx-c12(CH3).9.fid
166.44
143.38
129.66
129.10
128.35
77.48
77.16
76.84
71.61
36.22
31.88
29.30
25.54
22.71
21.74
20.22
14.18
Me
O
O
nHex
Me
3b
13C NMR (101 MHz, CDCl3)
f1 (ppm)

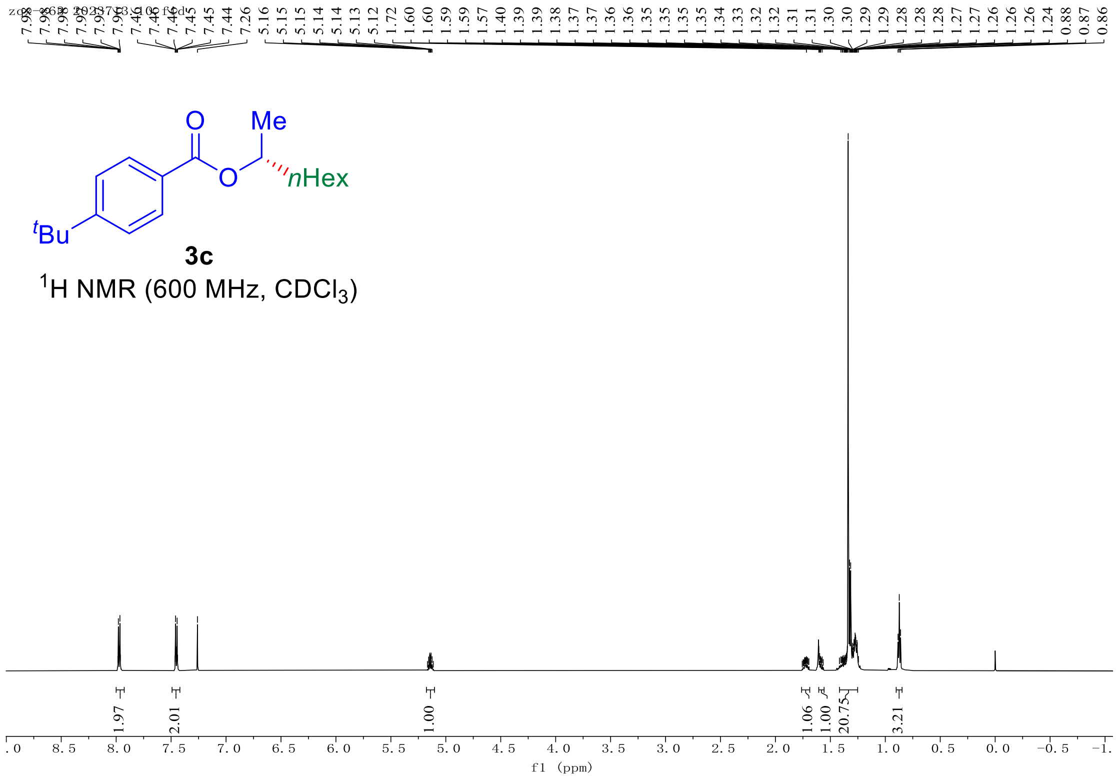
3c
^{1}H NMR (600 MHz, CDCl$_3$)
f1 (ppm)

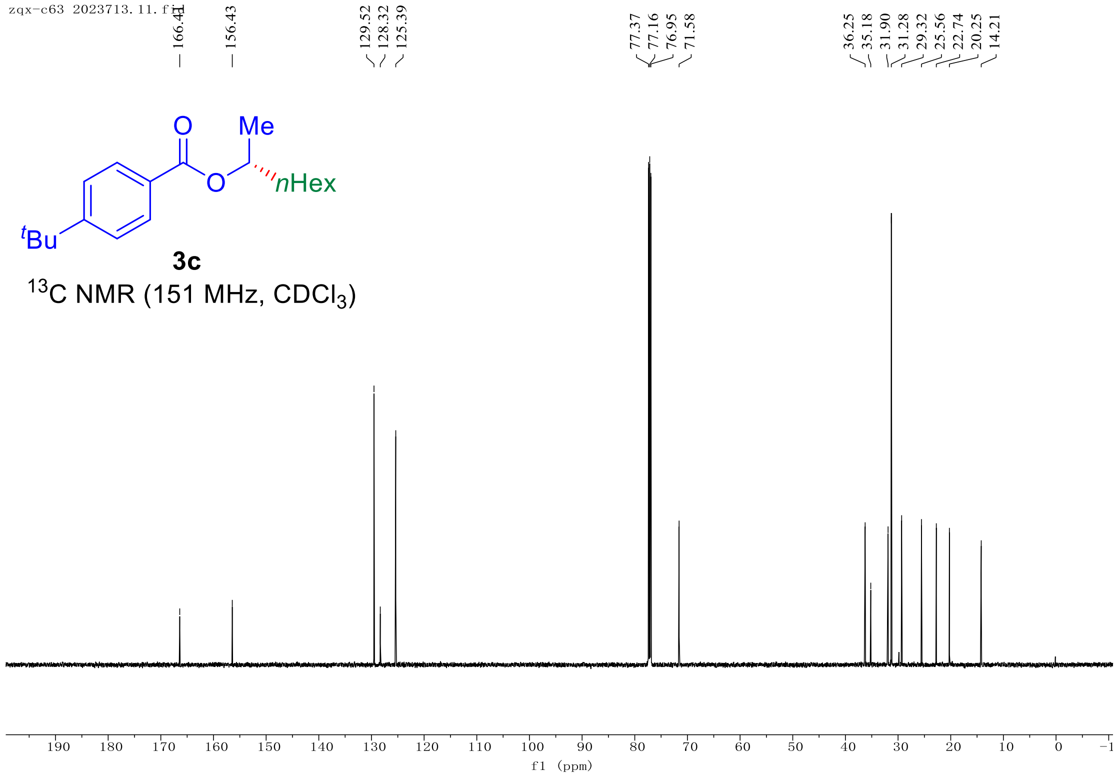
3c
^{13}C NMR (151 MHz, CDCl$_3$)
166.41
156.43
129.52
128.32
125.39
77.37
77.16
76.95
71.58
36.25
35.18
31.90
31.28
29.32
25.56
22.74
20.25
14.21
f1 (ppm)

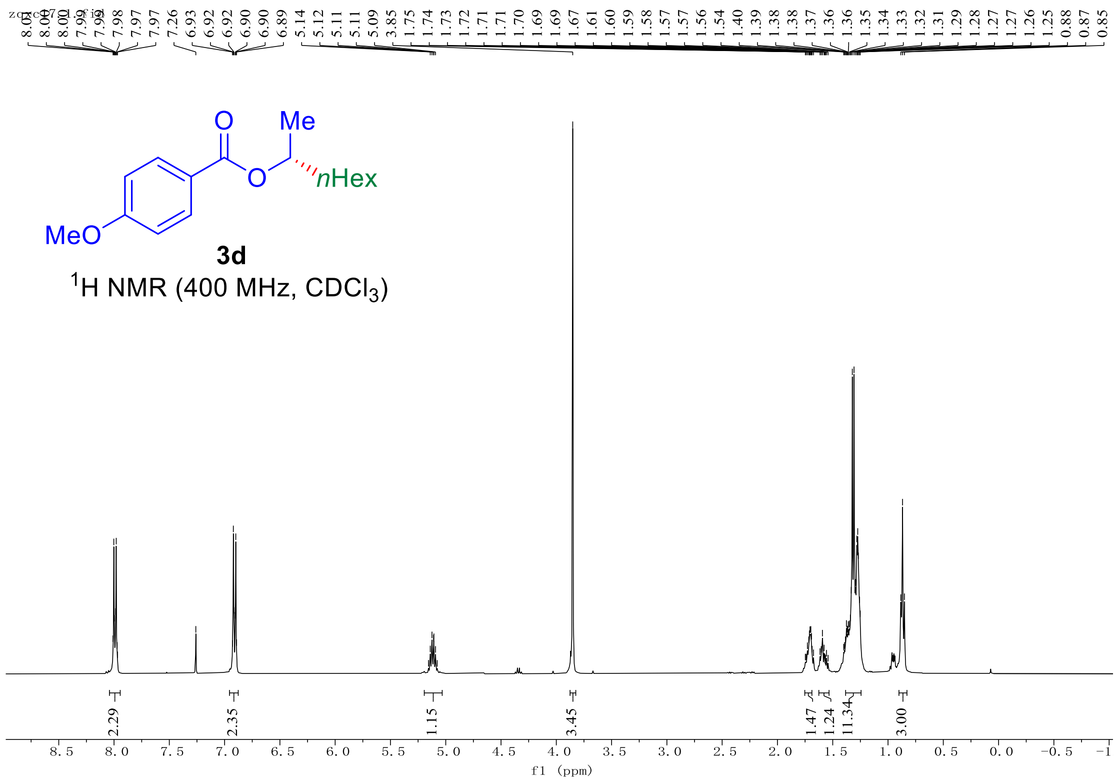
7.98
7.97
7.97
7.26
6.93
6.92
6.92
6.90
6.90
6.89
5.14
5.12
5.11
5.11
5.09
3.85
1.75
1.74
1.73
1.72
1.71
1.71
1.70
1.69
1.69
1.67
1.61
1.60
1.59
1.58
1.57
1.57
1.56
1.54
1.40
1.39
1.38
1.38
1.37
1.36
1.36
1.35
1.34
1.33
1.32
1.31
1.29
1.28
1.28
1.27
1.27
1.26
1.25
0.88
0.87
0.85
O
Me
O
nHex
MeO
3d
1H NMR (400 MHz, CDCl3)
2.29
2.35
1.15
3.45
1.47
1.24
11.34
3.00
f1 (ppm)

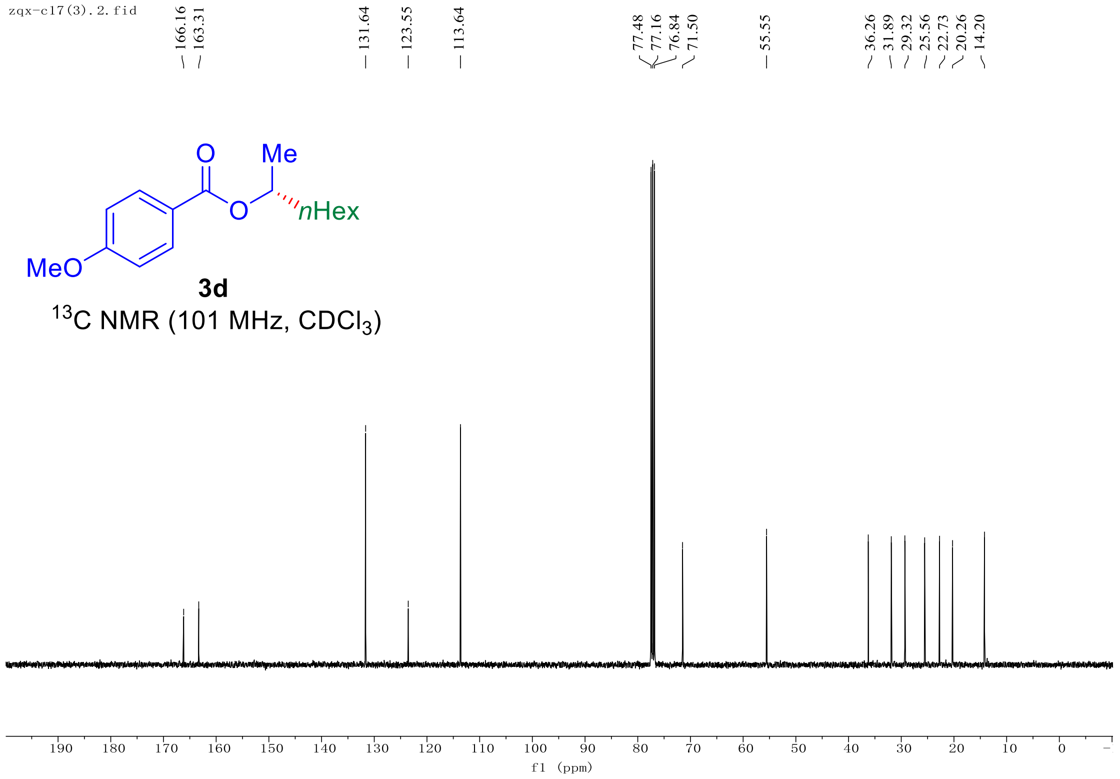
zqx-c17(3).2.fid
166.16
163.31
131.64
123.55
113.64
77.48
77.16
76.84
71.50
55.55
36.26
31.89
29.32
25.56
22.73
20.26
14.20
O
Me
O
nHex
MeO
3d
13C NMR (101 MHz, CDCl3)
f1 (ppm)

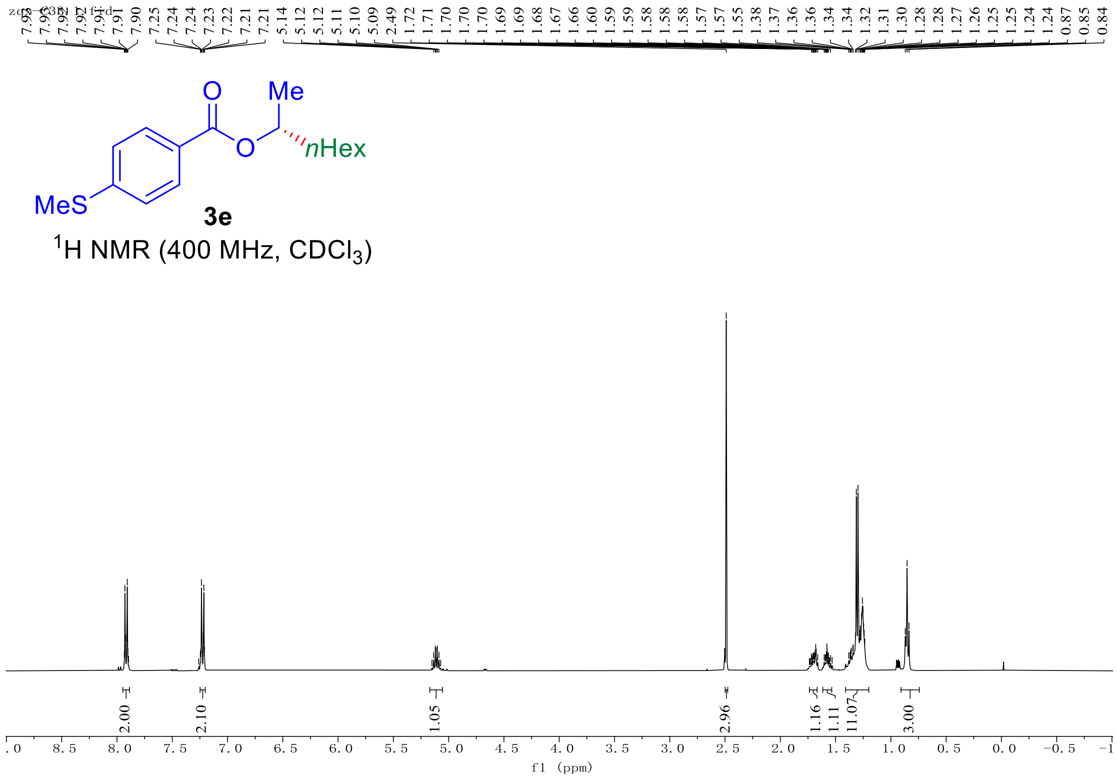

7.25 7.24 7.24 7.23 7.22 7.21 7.21 5.14 5.12 5.12 5.11 5.10 5.09 2.49 1.72 1.71 1.70 1.70 1.70 1.69 1.69 1.68 1.67 1.66 1.60 1.59 1.59 1.58 1.58 1.58 1.57 1.57 1.55 1.38 1.37 1.36 1.36 1.34 1.34 1.32 1.31 1.30 1.28 1.28 1.27 1.26 1.25 1.25 1.24 0.87 0.85 0.84
O
Me
O
nHex
MeS
3e
1H NMR (400 MHz, CDCl3)
2.00
2.10
1.05
2.96
1.16
1.11
11.07
3.00
f1 (ppm)

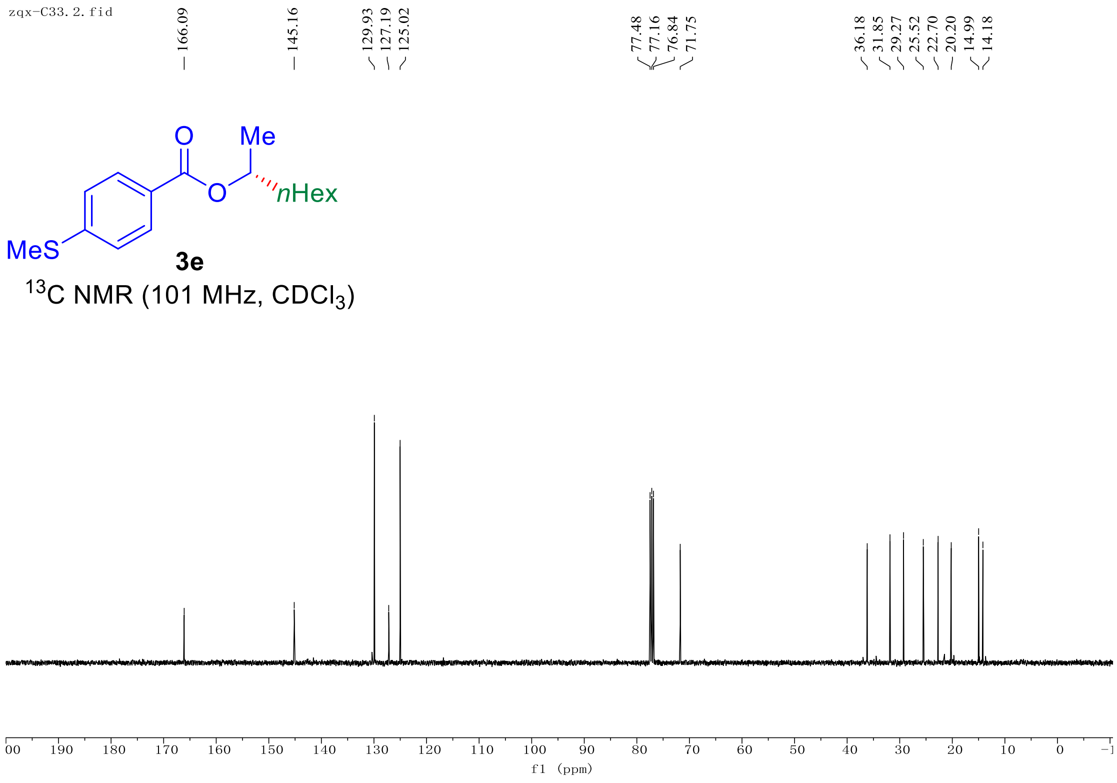

zqx-C33.2.fid
166.09
145.16
129.93
127.19
125.02
77.48
77.16
76.84
71.75
36.18
31.85
29.27
25.52
22.70
20.20
14.99
14.18
O
Me
O
nHex
MeS
3e
13C NMR (101 MHz, CDCl3)
f1 (ppm)

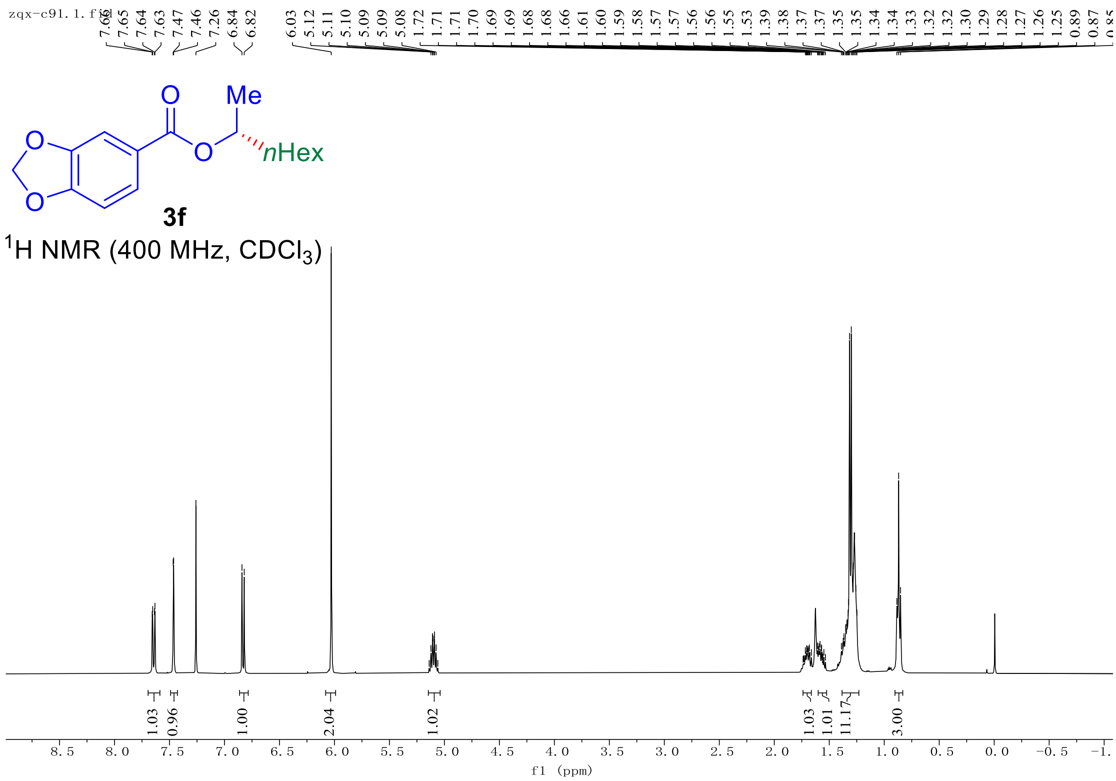

zqx-c91.1.fid
7.66 7.65 7.64 7.63 7.47 7.46 7.26 6.84 6.82
6.03 5.12 5.11 5.10 5.09 5.09 5.08 1.72 1.71 1.71 1.70 1.69 1.69 1.68 1.68 1.66 1.61 1.60 1.59 1.58 1.57 1.57 1.56 1.56 1.55 1.53 1.39 1.38 1.37 1.37 1.35 1.35 1.34 1.34 1.33 1.32 1.32 1.30 1.29 1.28 1.27 1.26 1.25 0.89 0.87
Me
nHex
3f
1H NMR (400 MHz, CDCl3)
1.03 0.96 1.00 2.04 1.02 1.03 1.01 11.17 3.00
f1 (ppm)

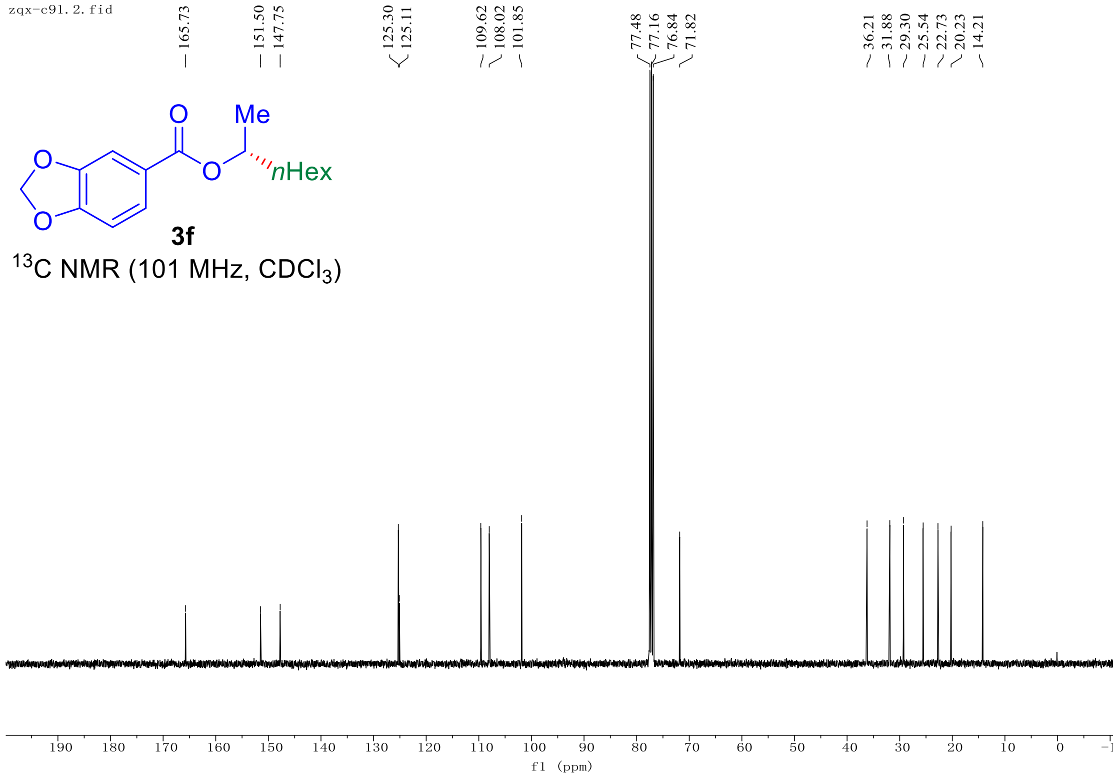

zqx-c91.2.fid
165.73 151.50 147.75 125.30 125.11 109.62 108.02 101.85 77.48 77.16 76.84 71.82 36.21 31.88 29.30 25.54 22.73 20.23 14.21
Me
nHex
3f
13C NMR (101 MHz, CDCl3)
f1 (ppm)

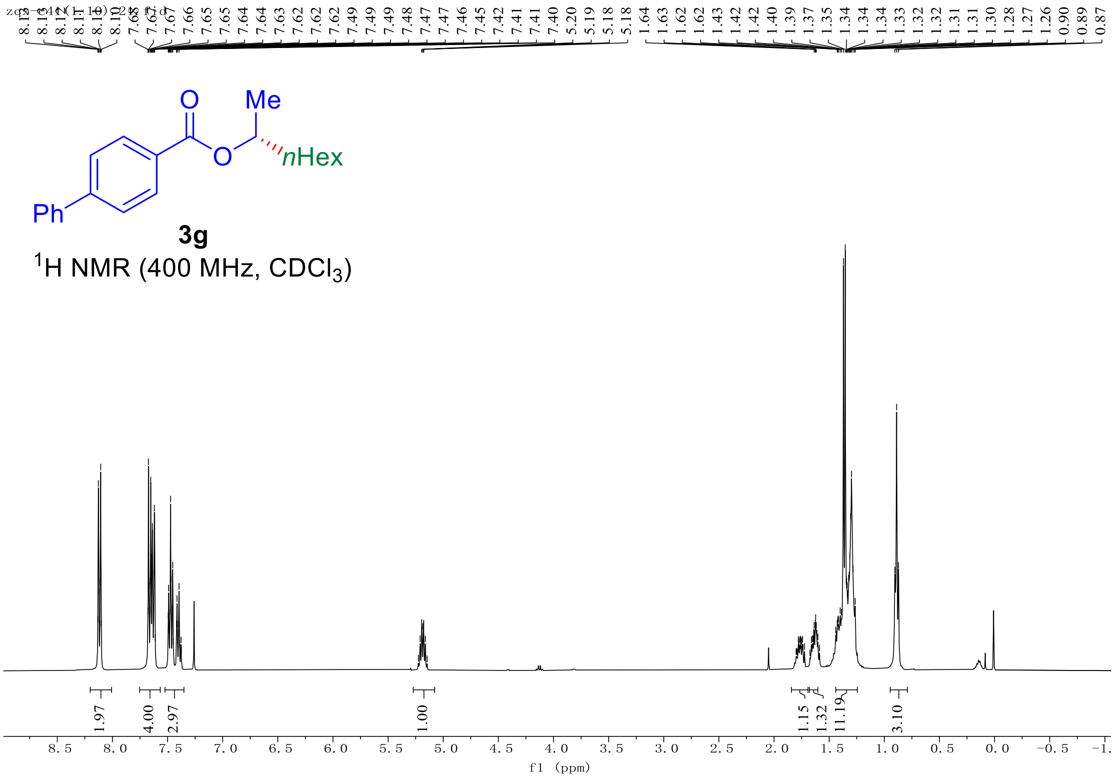
Me
O
O
nHex
Ph
3g
1H NMR (400 MHz, CDCl3)
f1 (ppm)

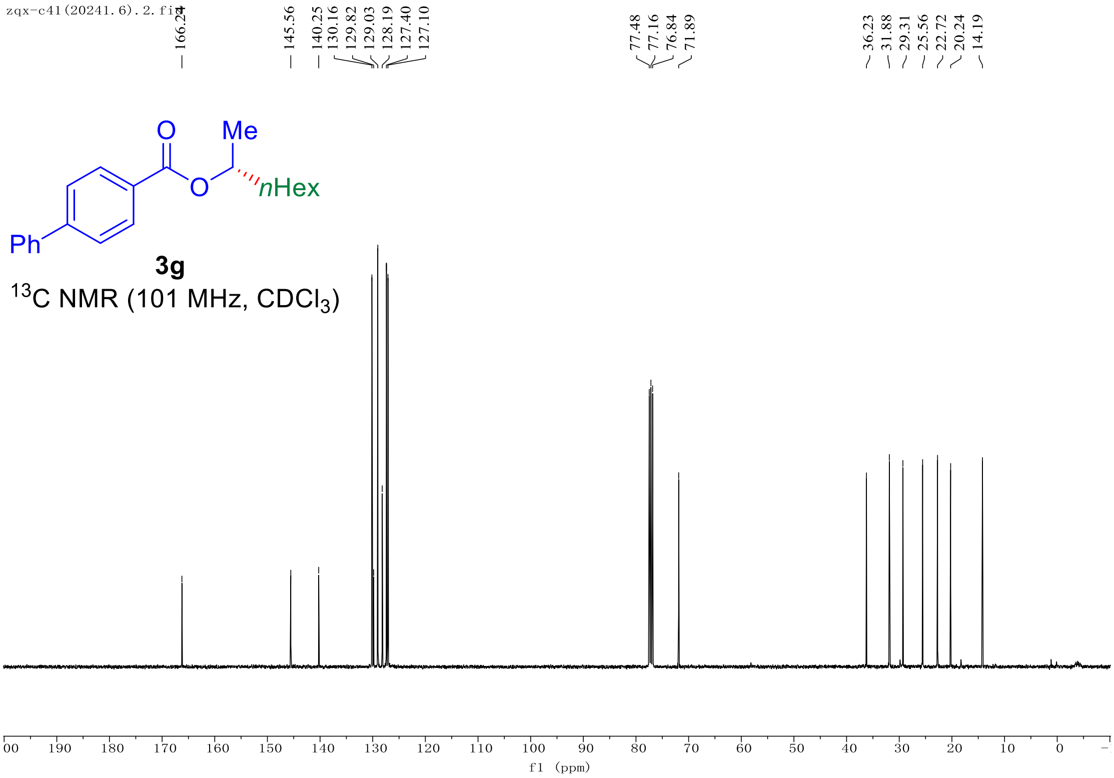
Me
O
O
nHex
Ph
3g
13C NMR (101 MHz, CDCl3)
f1 (ppm)

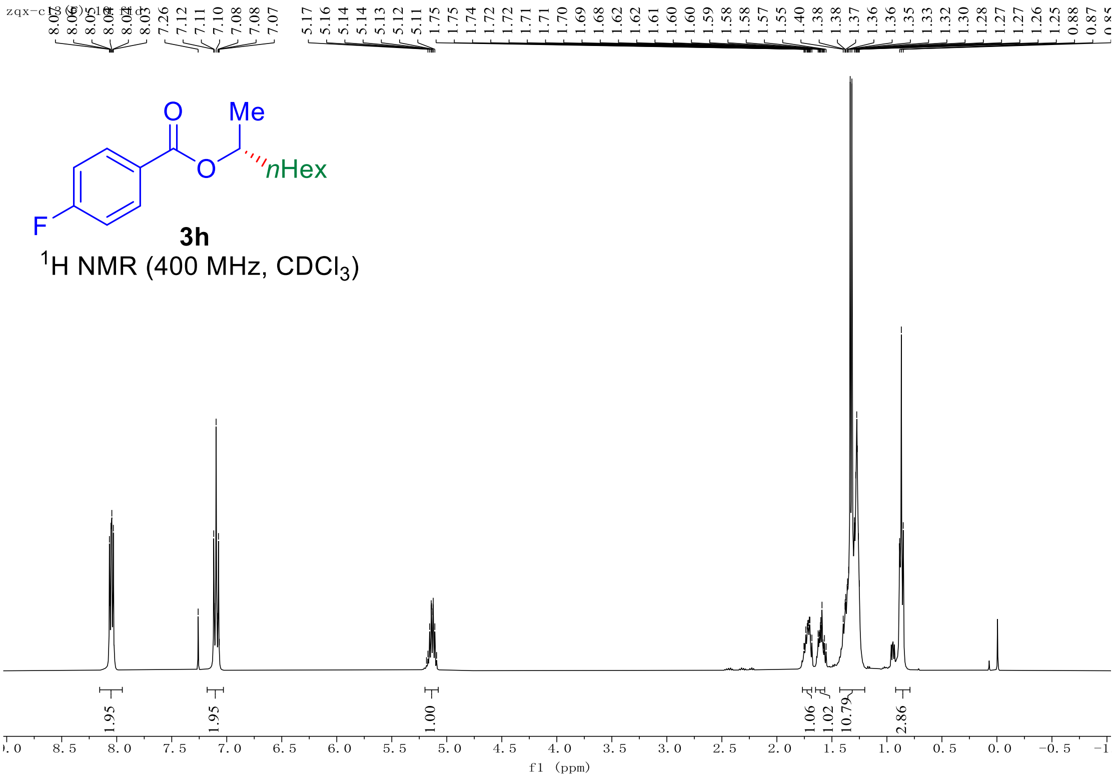
3h
^{1}H NMR (400 MHz, $CDCl_3$)

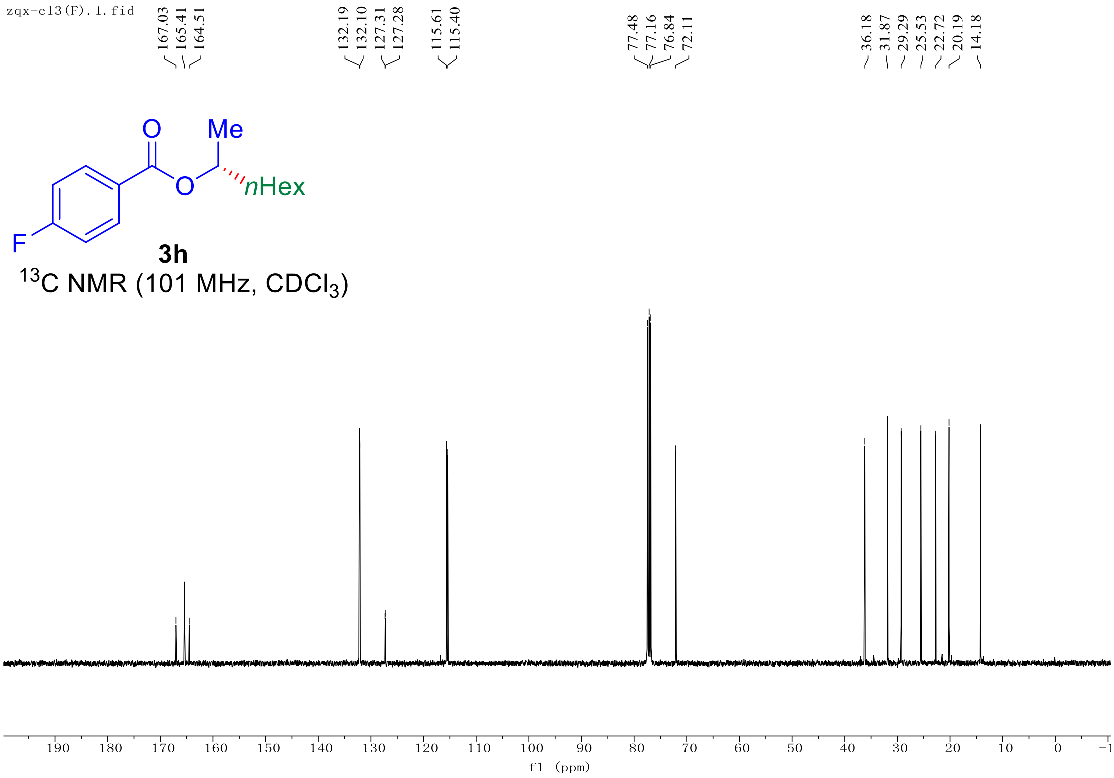
3h
^{13}C NMR (101 MHz, $CDCl_3$)

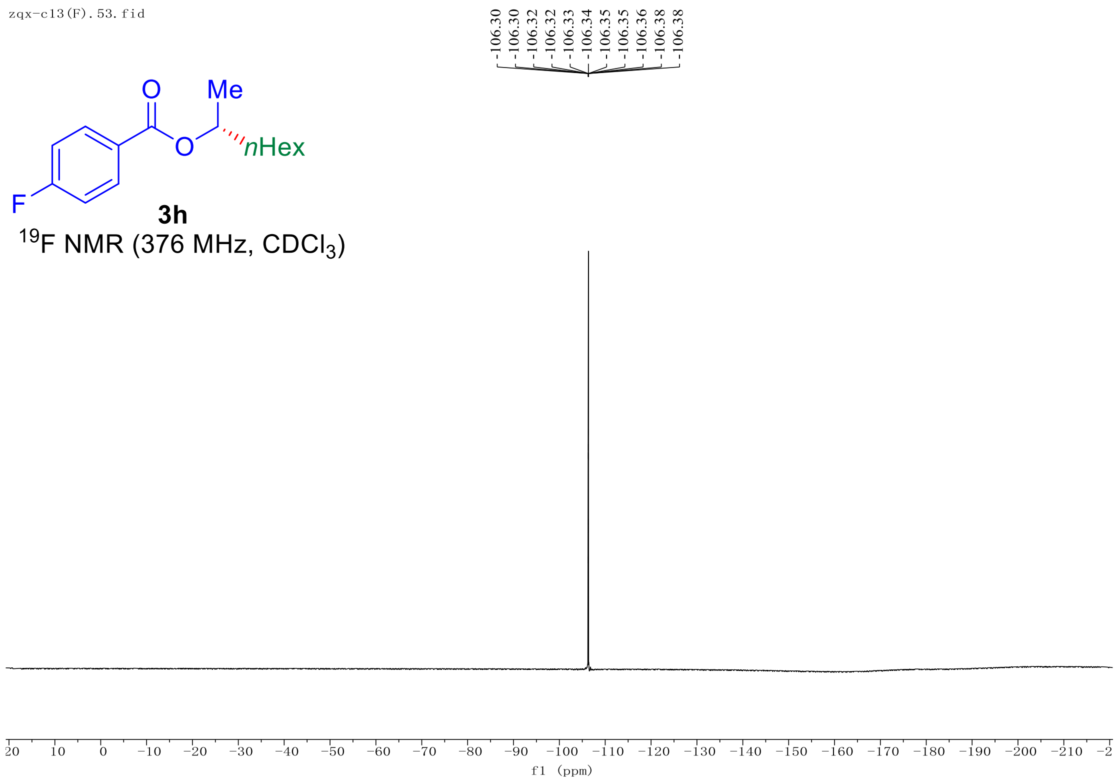
zqx-c13(F).53.fid
-106.30 -106.30 -106.32 -106.32 -106.33 -106.34 -106.35 -106.35 -106.36 -106.38 -106.38
O
Me
O
nHex
F
3h
^{19}F NMR (376 MHz, CDCl$_3$)
f1 (ppm)

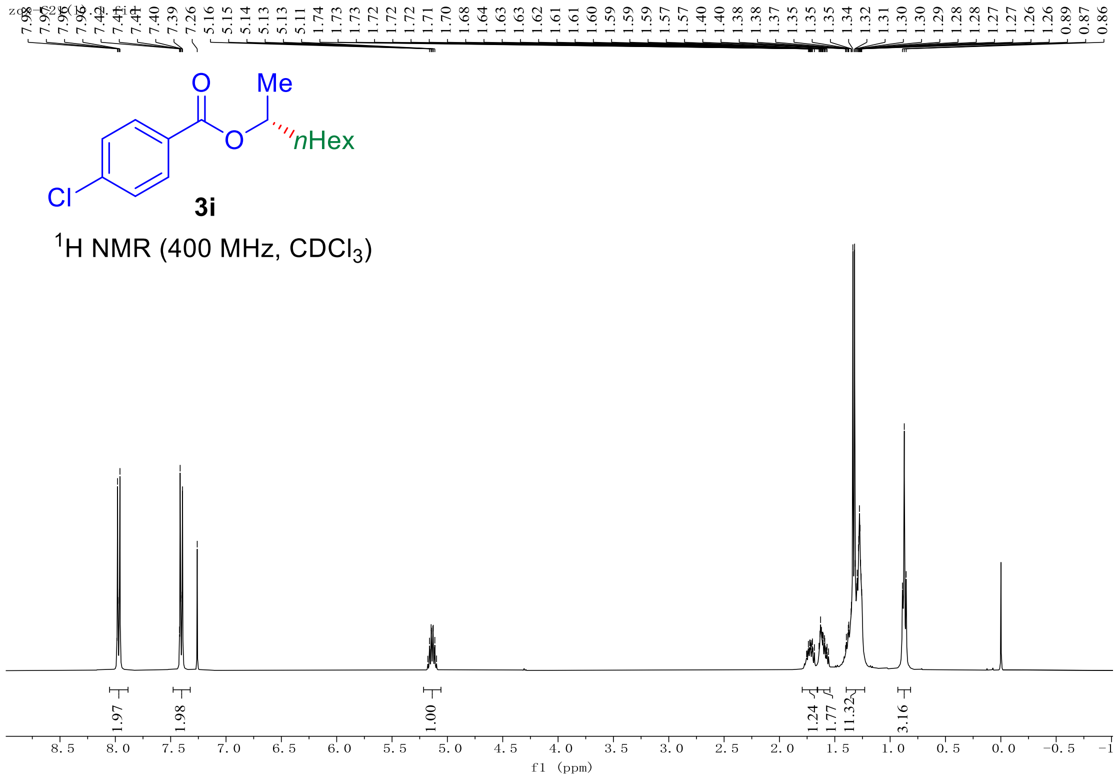
7.42 7.41 7.41 7.40 7.39 7.26 5.16 5.15 5.14 5.13 5.13 5.11 1.74 1.73 1.73 1.72 1.72 1.72 1.71 1.70 1.68 1.64 1.63 1.63 1.62 1.61 1.61 1.60 1.59 1.59 1.59 1.57 1.57 1.40 1.40 1.38 1.38 1.37 1.35 1.35 1.34 1.32 1.31 1.30 1.30 1.29 1.28 1.28 1.27 1.27 1.26 0.89 0.87 0.86
O
Me
O
nHex
Cl
3i
^{1}H NMR (400 MHz, CDCl$_3$)
1.97
1.98
1.00
1.24
1.77
11.32
3.16
f1 (ppm)

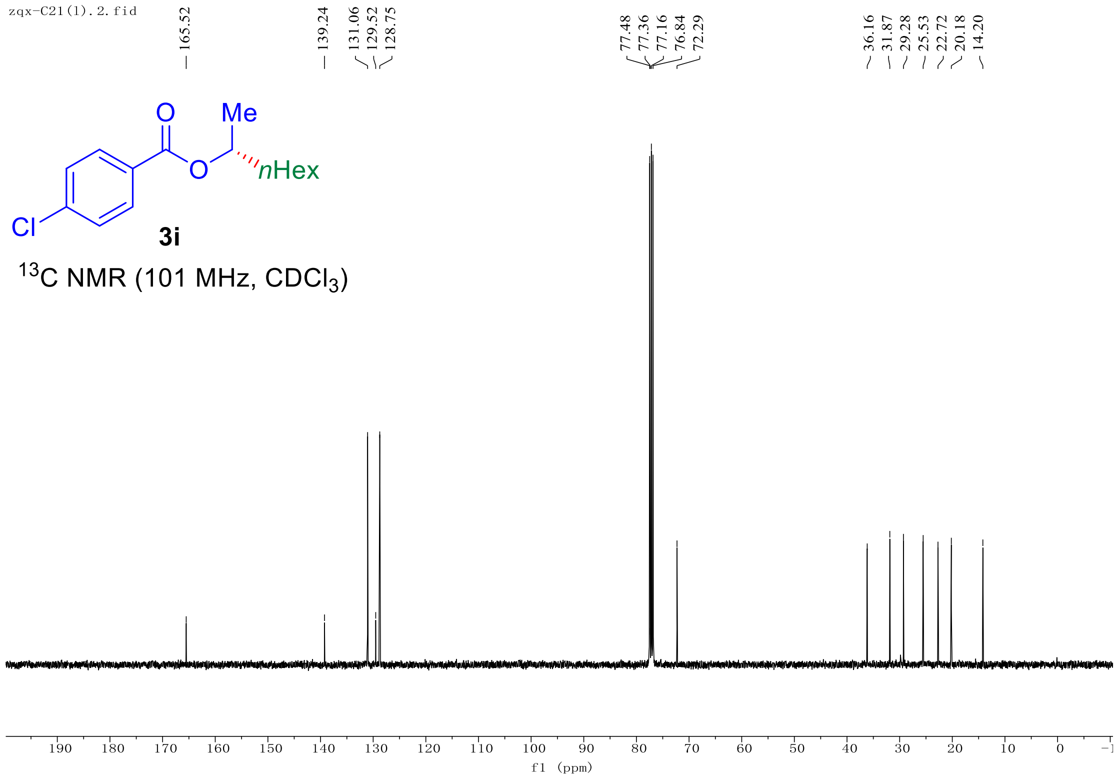
zqx-C21(1).2.fid
165.52
139.24
131.06
129.52
128.75
77.48
77.36
77.16
76.84
72.29
36.16
31.87
29.28
25.53
22.72
20.18
14.20
O
Me
O
nHex
Cl
3i
^{13}C NMR (101 MHz, $CDCl_3$)
f1 (ppm)

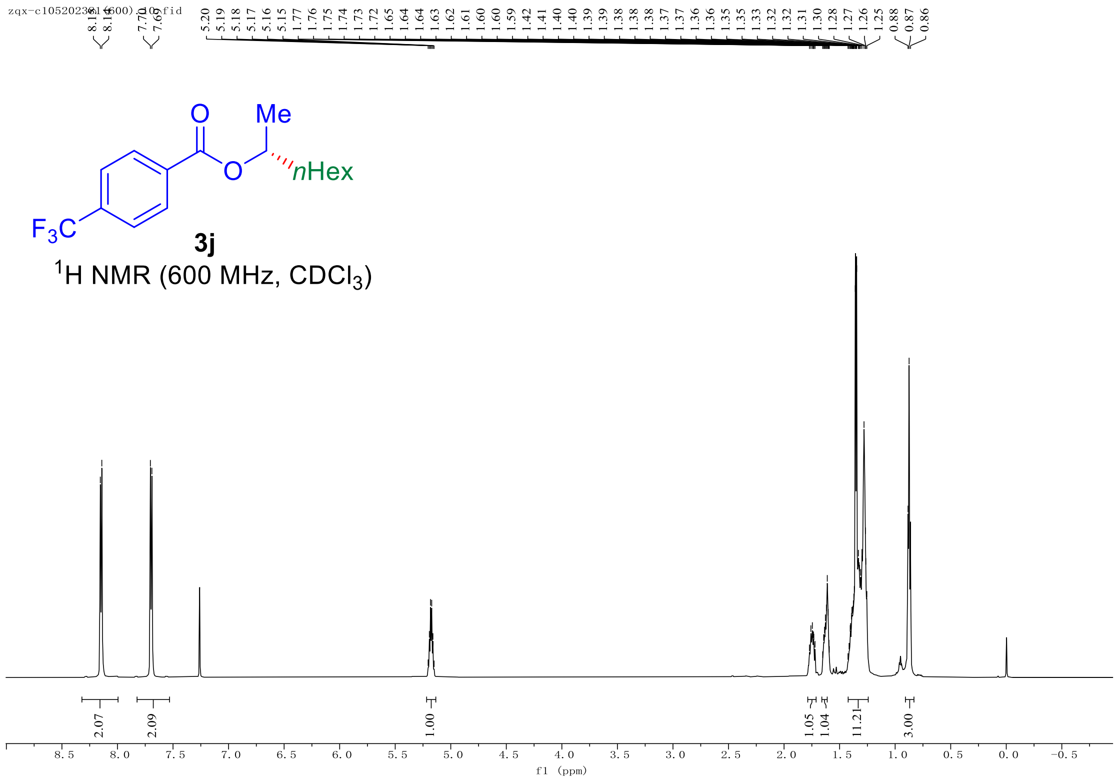
O
Me
O
nHex
F_3C
3j
^{1}H NMR (600 MHz, $CDCl_3$)
2.07
2.09
1.00
1.05
1.04
11.21
3.00
f1 (ppm)

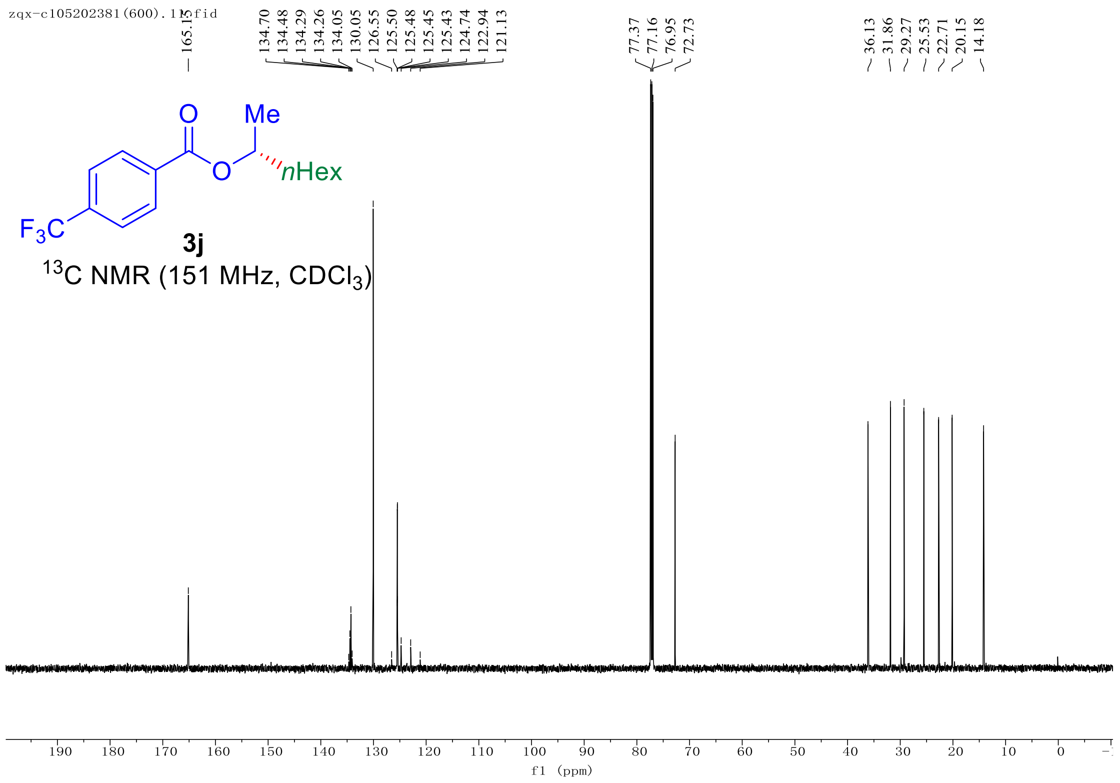

165.13
134.70
134.48
134.29
134.26
134.05
130.05
126.55
125.50
125.48
125.45
125.43
124.74
122.94
121.13
77.37
77.16
76.95
72.73
36.13
31.86
29.27
25.53
22.71
20.15
14.18
O
Me
O
nHex
F3C
3j
13C NMR (151 MHz, CDCl3)
190 180 170 160 150 140 130 120 110 100 90 80 70 60 50 40 30 20 10 0
f1 (ppm)

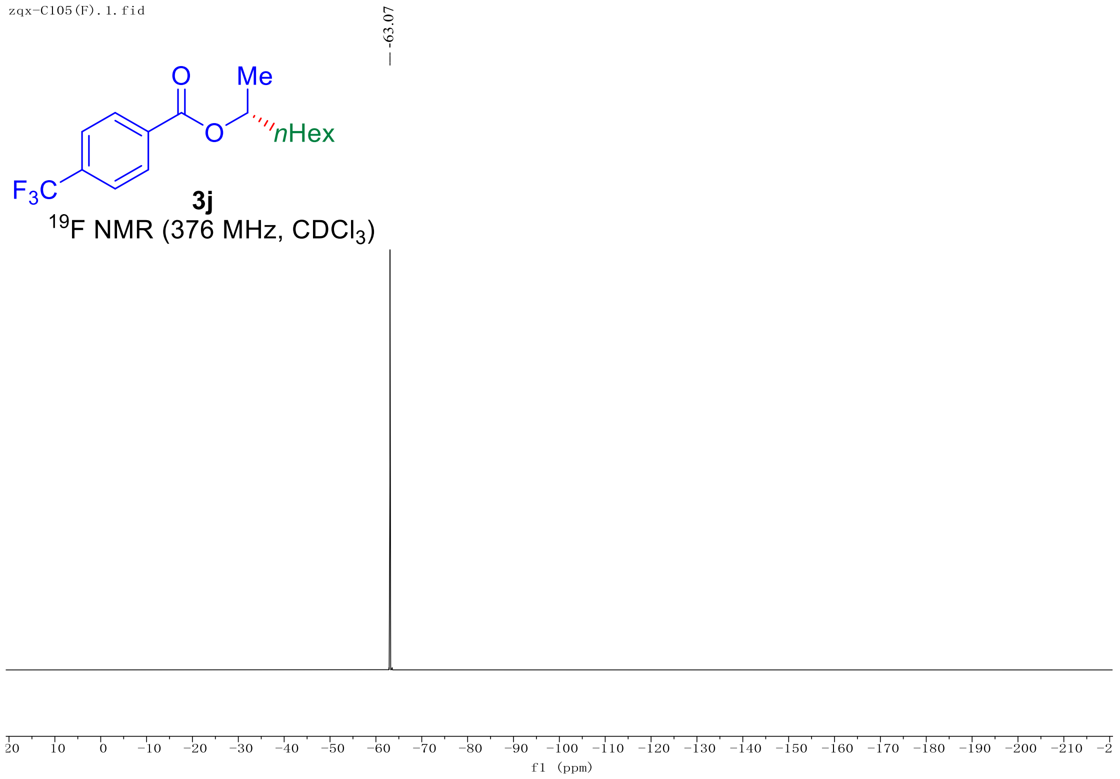

zqx-C105(F).1.fid
-63.07
O
Me
O
nHex
F3C
3j
19F NMR (376 MHz, CDCl3)
20 10 0 −10 −20 −30 −40 −50 −60 −70 −80 −90 −100 −110 −120 −130 −140 −150 −160 −170 −180 −190 −200 −210
f1 (ppm)

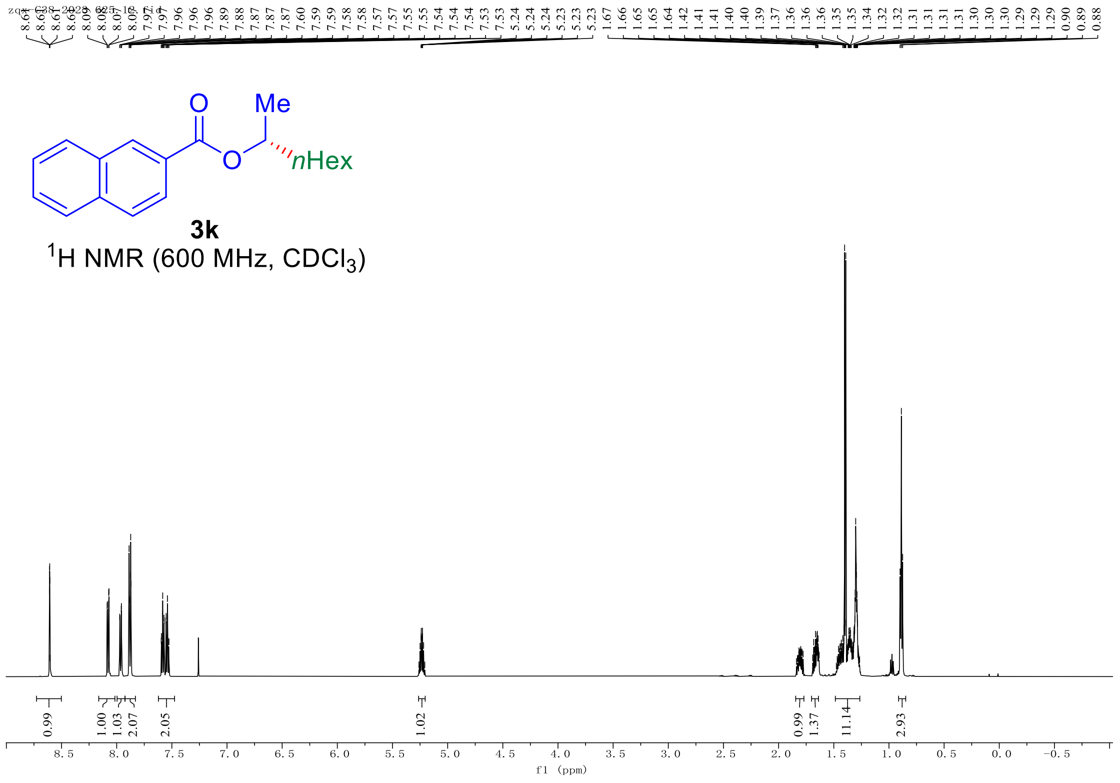
zqx-C38 2023 625.11.fid
O
Me
O
nHex
3k
1H NMR (600 MHz, CDCl3)
f1 (ppm)

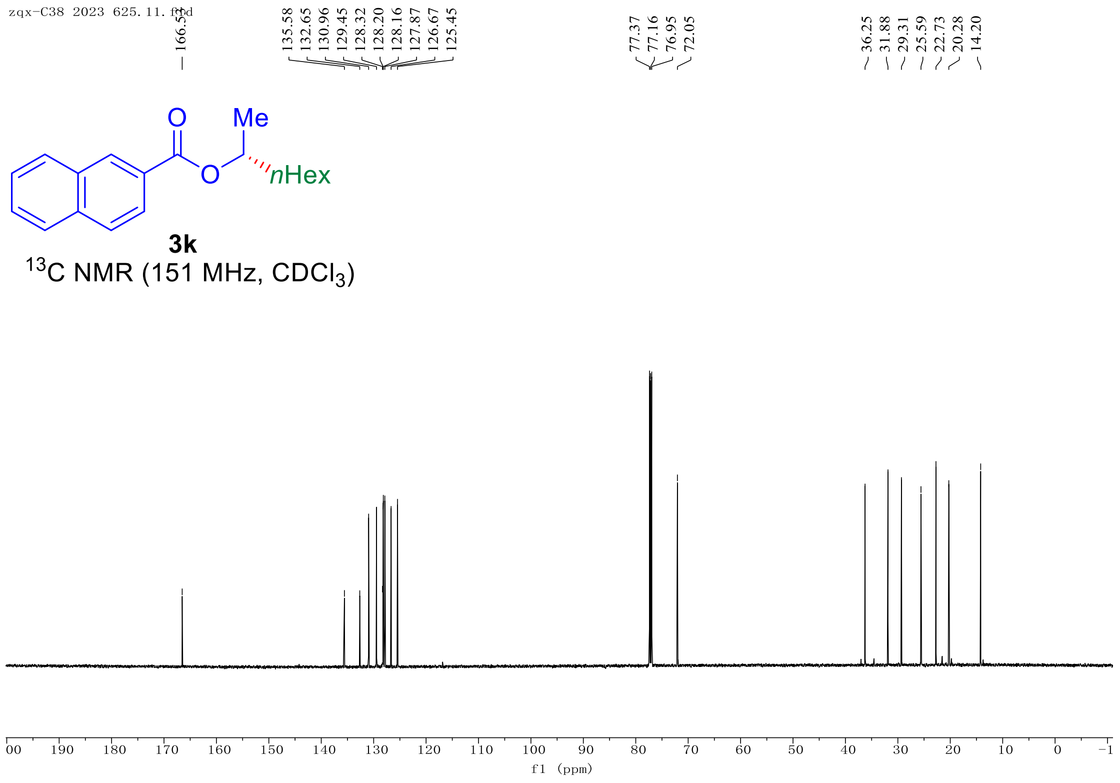
zqx-C38 2023 625.11.fid
166.53
135.58
132.65
130.96
129.45
128.32
128.20
128.16
127.87
126.67
125.45
77.37
77.16
76.95
72.05
36.25
31.88
29.31
25.59
22.73
20.28
14.20
O
Me
O
nHex
3k
13C NMR (151 MHz, CDCl3)
f1 (ppm)

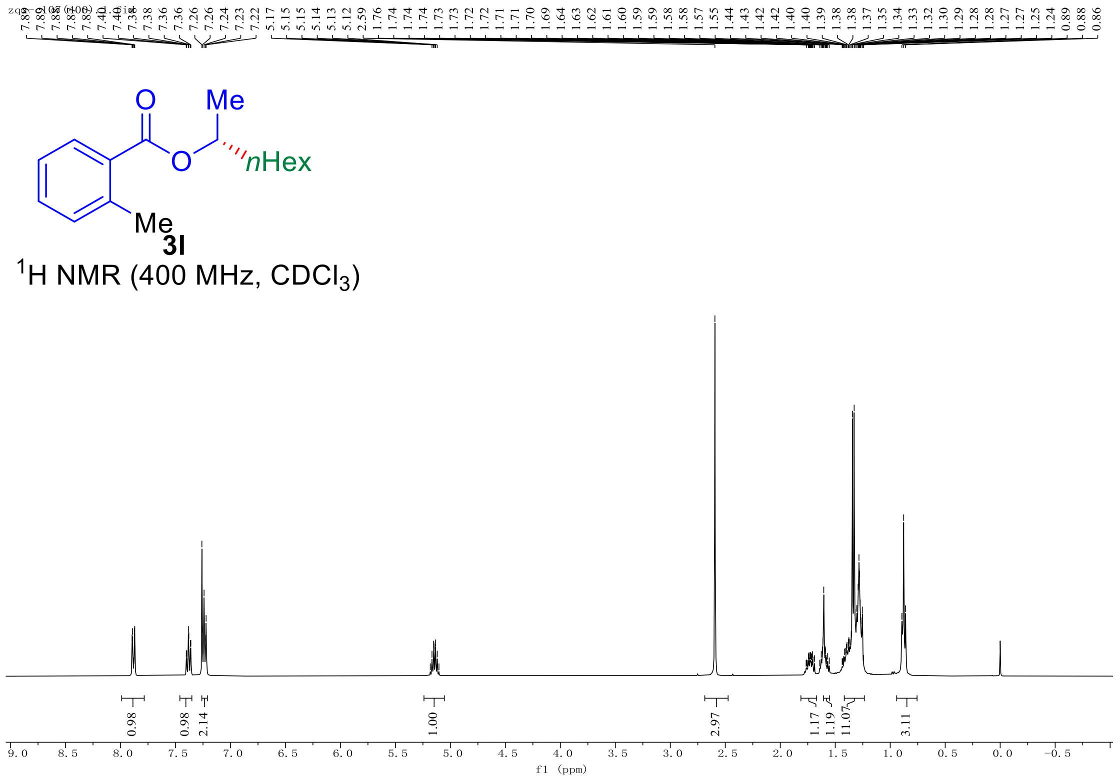
Me
O
O
nHex
Me
3l
1H NMR (400 MHz, CDCl3)
7.38 7.36 7.36 7.26 7.26 7.24 7.23 7.22 5.17 5.15 5.15 5.14 5.13 5.12 2.59 1.76 1.74 1.74 1.74 1.73 1.73 1.72 1.72 1.71 1.70 1.69 1.64 1.63 1.62 1.61 1.60 1.59 1.59 1.58 1.58 1.57 1.55 1.44 1.43 1.42 1.42 1.40 1.40 1.39 1.38 1.38 1.37 1.35 1.34 1.33 1.32 1.30 1.29 1.28 1.28 1.27 1.25 1.24 0.89 0.88 0.86
0.98
0.98
2.14
1.00
2.97
1.17
1.19
11.07
3.11
f1 (ppm)

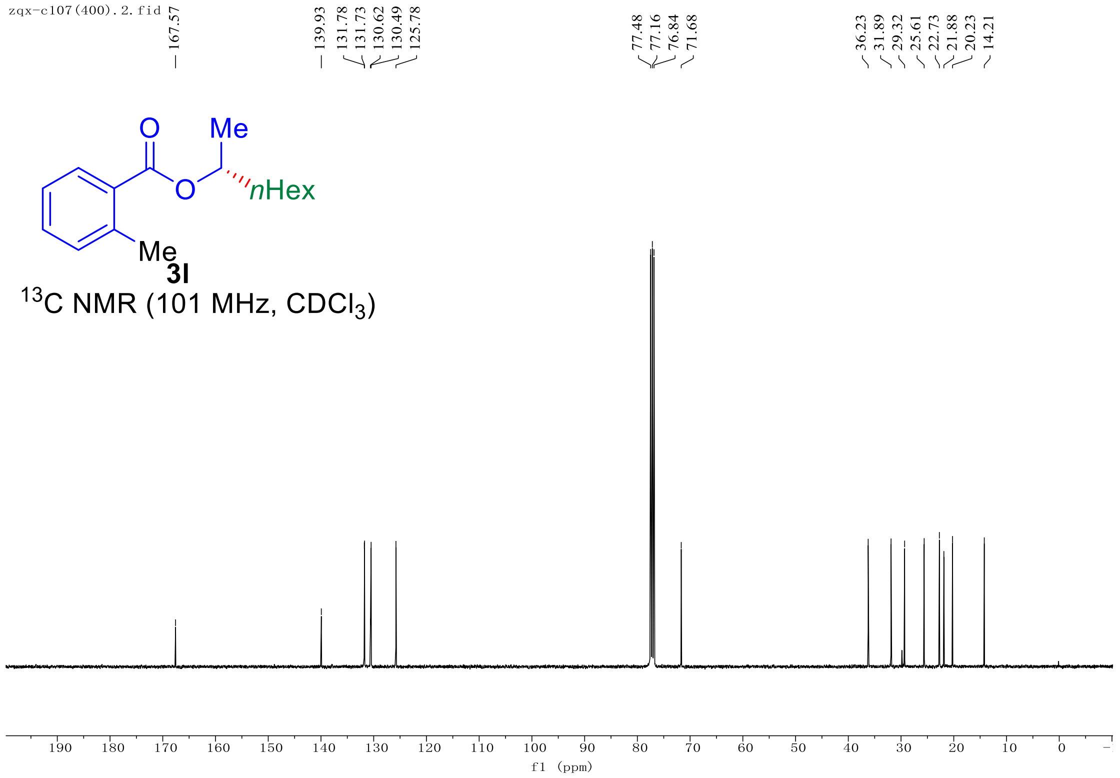
zqx-c107(400).2.fid
167.57
139.93 131.78 131.73 130.62 130.49 125.78
77.48 77.16 76.84 71.68
36.23 31.89 29.32 25.61 22.73 21.88 20.23 14.21
Me
O
O
nHex
Me
3l
13C NMR (101 MHz, CDCl3)
f1 (ppm)

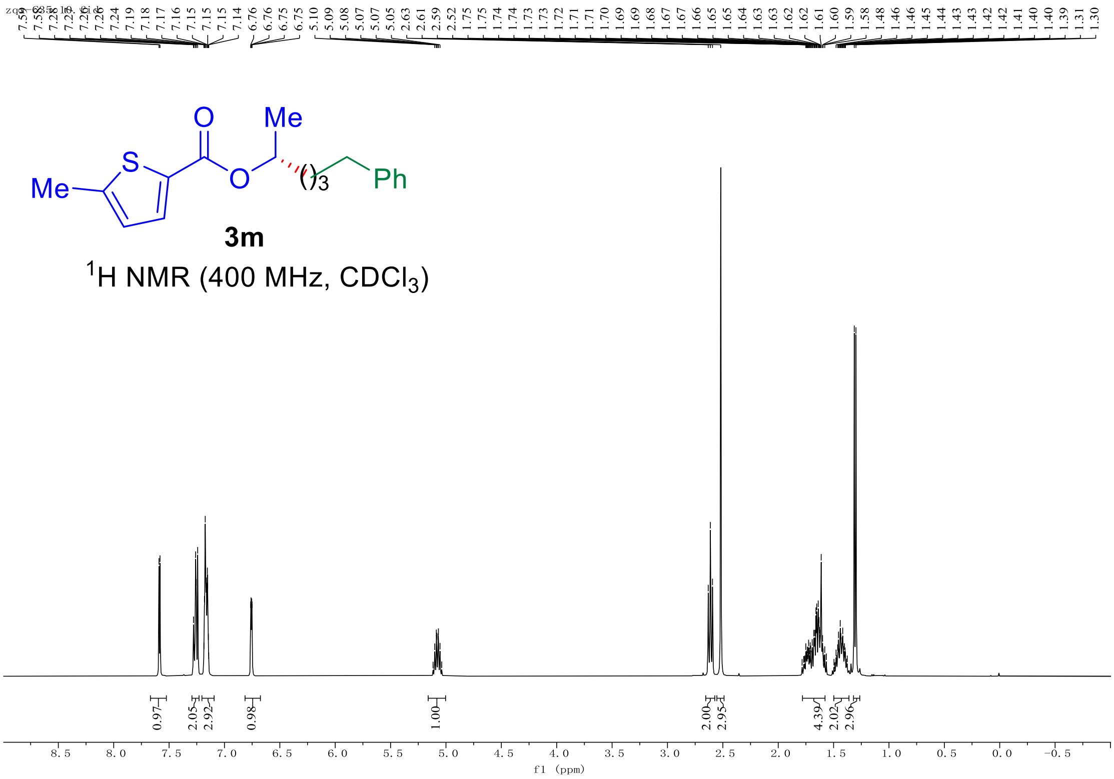

Me
O
S
O
Ph
3m
1H NMR (400 MHz, CDCl3)
0.97
2.05
2.92
0.98
1.00
2.00
2.95
4.39
2.02
2.96
f1 (ppm)

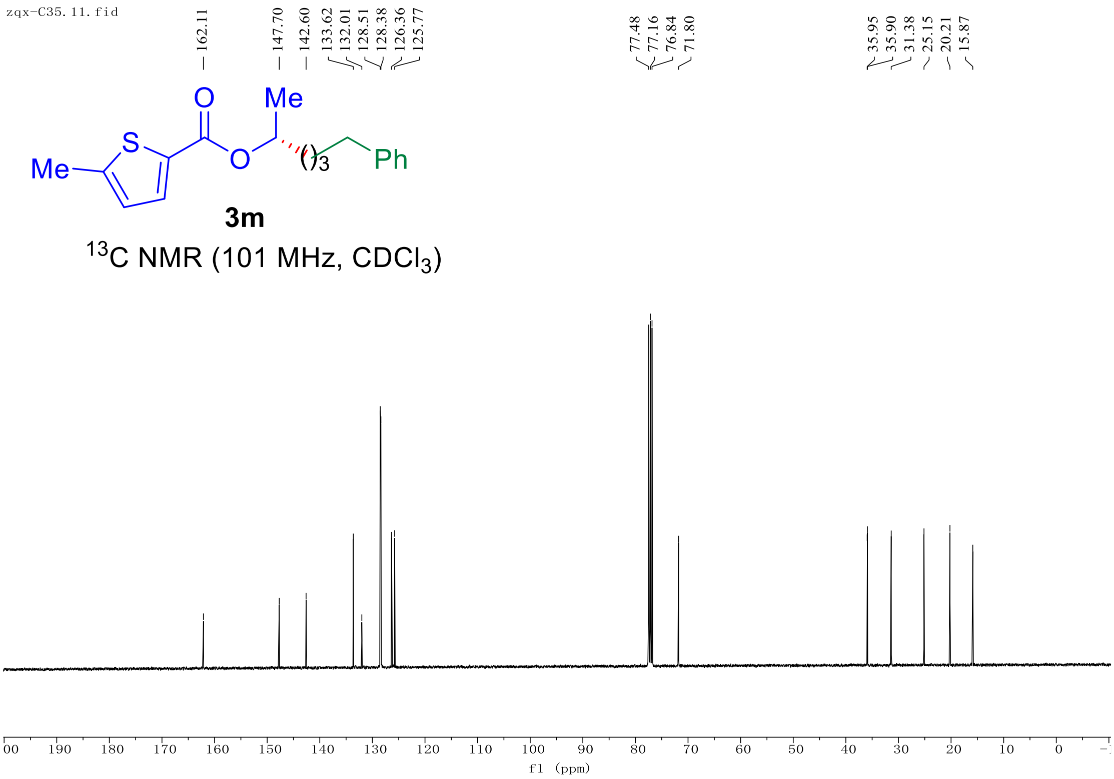

zqx-C35.11.fid
162.11
147.70
142.60
133.62
132.01
128.51
128.38
126.36
125.77
77.48
77.16
76.84
71.80
35.95
35.90
31.38
25.15
20.21
15.87
Me
O
S
O
Ph
3m
13C NMR (101 MHz, CDCl3)
f1 (ppm)

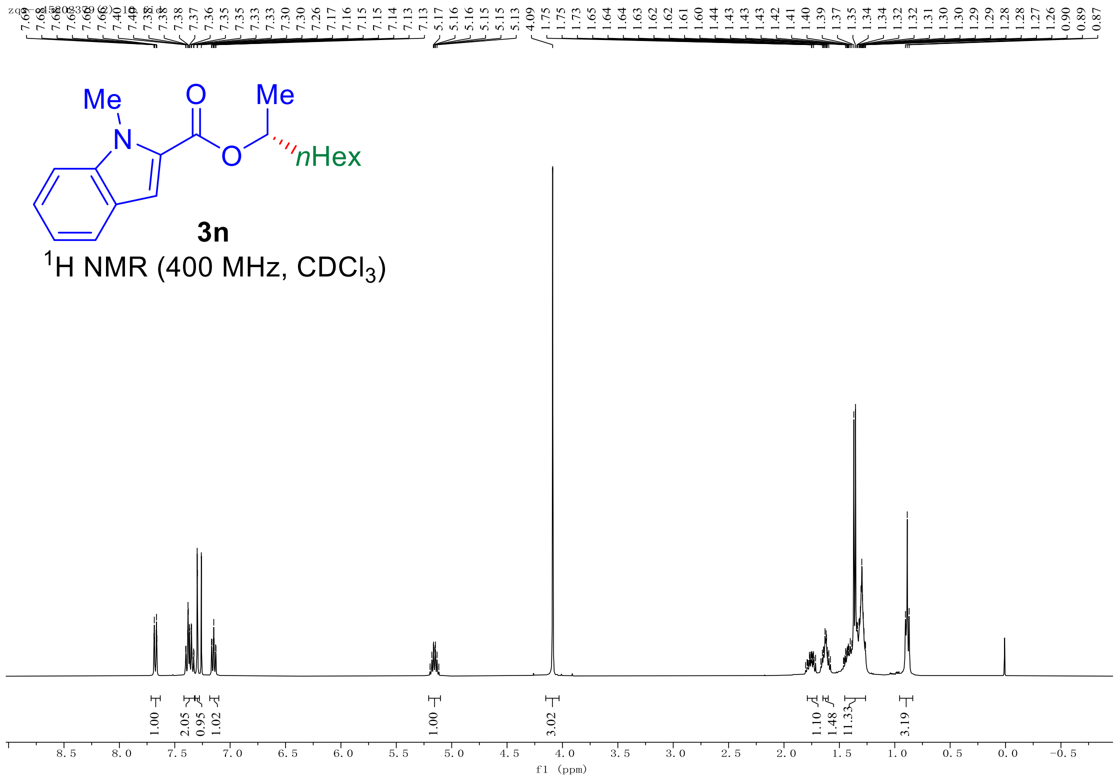
Me
O
Me
N
O
nHex
3n
1H NMR (400 MHz, CDCl3)
f1 (ppm)

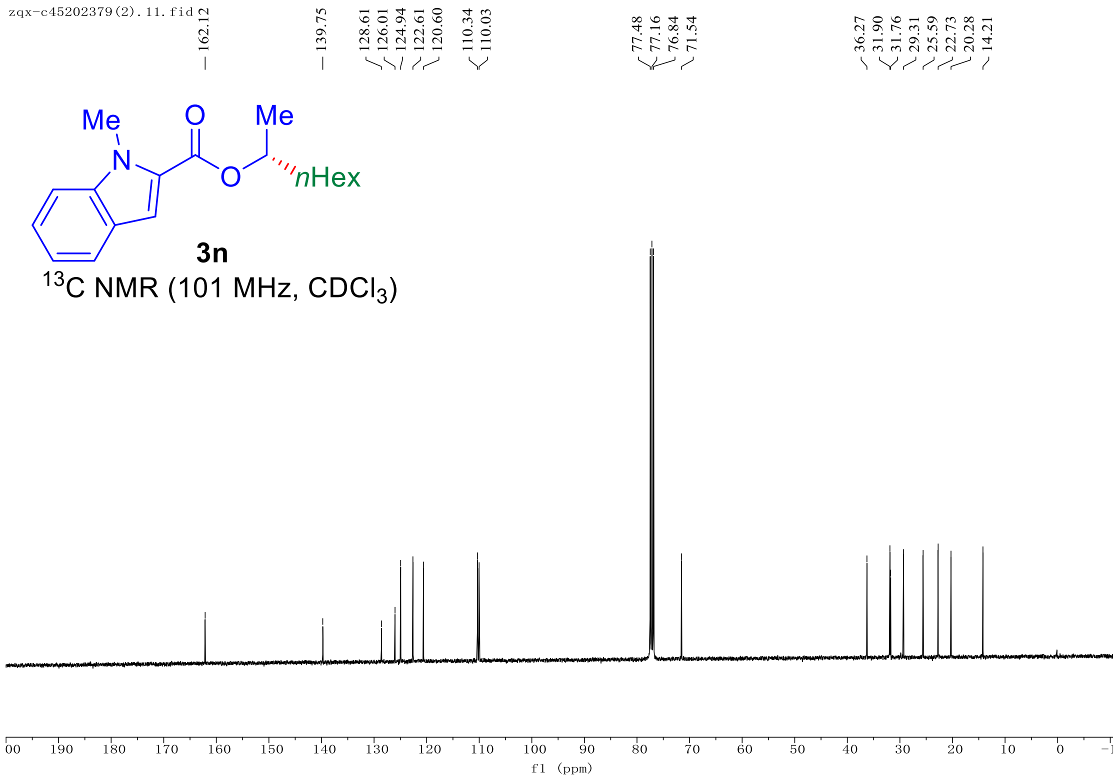
zqx-c45202379(2).11.fid
162.12
139.75
128.61
126.01
124.94
122.61
120.60
110.34
110.03
77.48
77.16
76.84
71.54
36.27
31.90
31.76
29.31
25.59
22.73
20.28
14.21
Me
O
Me
N
O
nHex
3n
13C NMR (101 MHz, CDCl3)
f1 (ppm)

zqx-D46.54

8.08 8.07 8.07 8.06 8.05 8.05 7.56 7.54 7.47 7.46 7.45 7.45 7.44 7.43 7.43 7.29 7.29 7.27 7.26 7.25 6.95 6.93 6.93 6.93 6.91 6.91 6.90 6.90 6.90 6.89 6.88 6.88 6.88 6.88 5.10 3.95 3.94 3.92 1.79 1.77 1.75 1.74 1.74 1.72 1.72 1.70 1.69 1.68 1.47 1.46 1.46 1.45 1.45 1.44 1.44 1.43 1.42 1.42 1.41 1.41 1.40 1.39 0.98 0.96 0.95

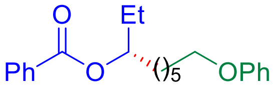

3o

^{1}H NMR (400 MHz, $CDCl_3$)

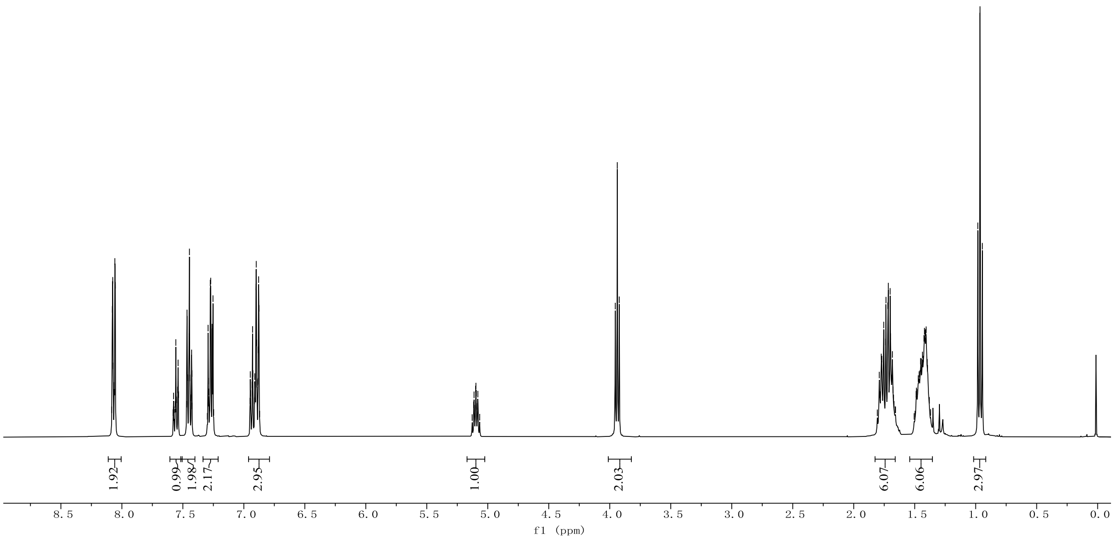

zqx-D46.54.fid

166.54 159.21 132.83 130.96 129.66 129.52 128.44 120.58 114.61 77.48 77.16 76.84 76.23 67.87 33.76 29.44 29.34 27.23 26.10 25.43 9.79

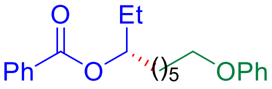

3o

^{13}C NMR (101 MHz, $CDCl_3$)

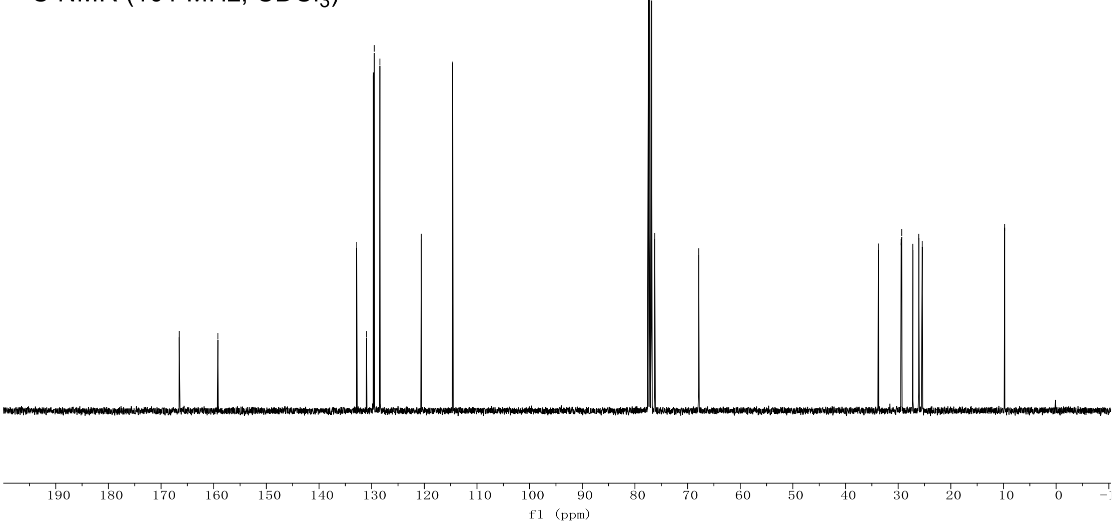

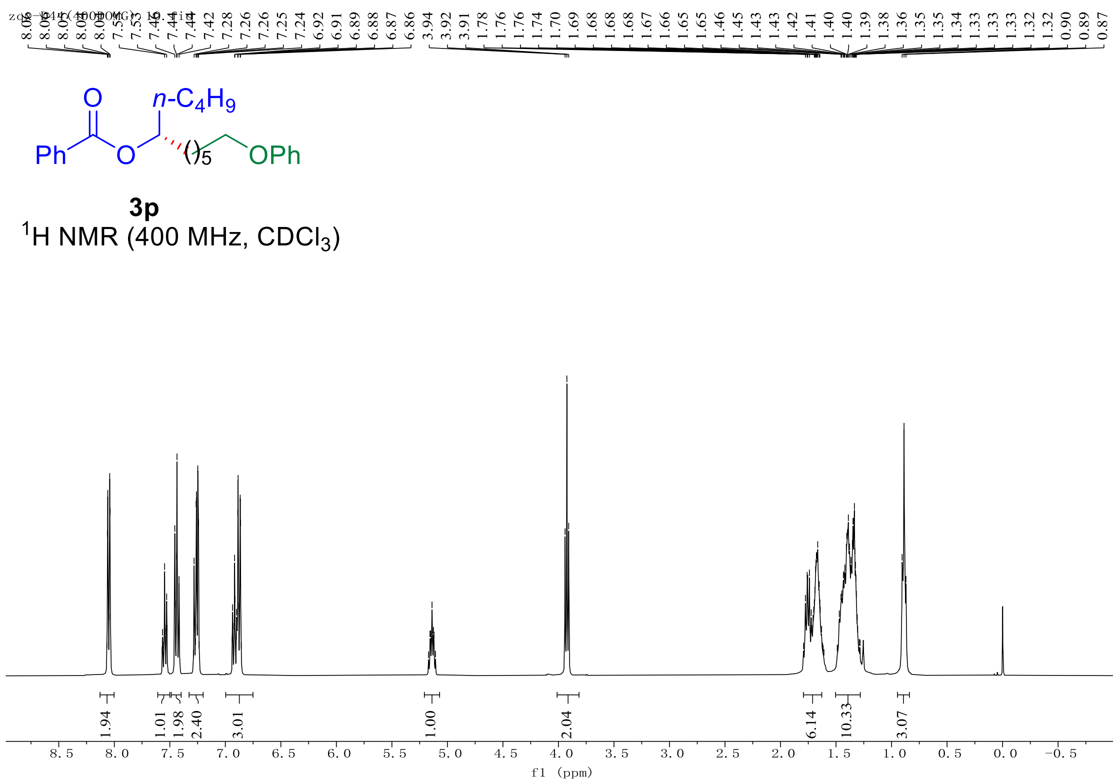

3p

^{1}H NMR (400 MHz, $CDCl_3$)

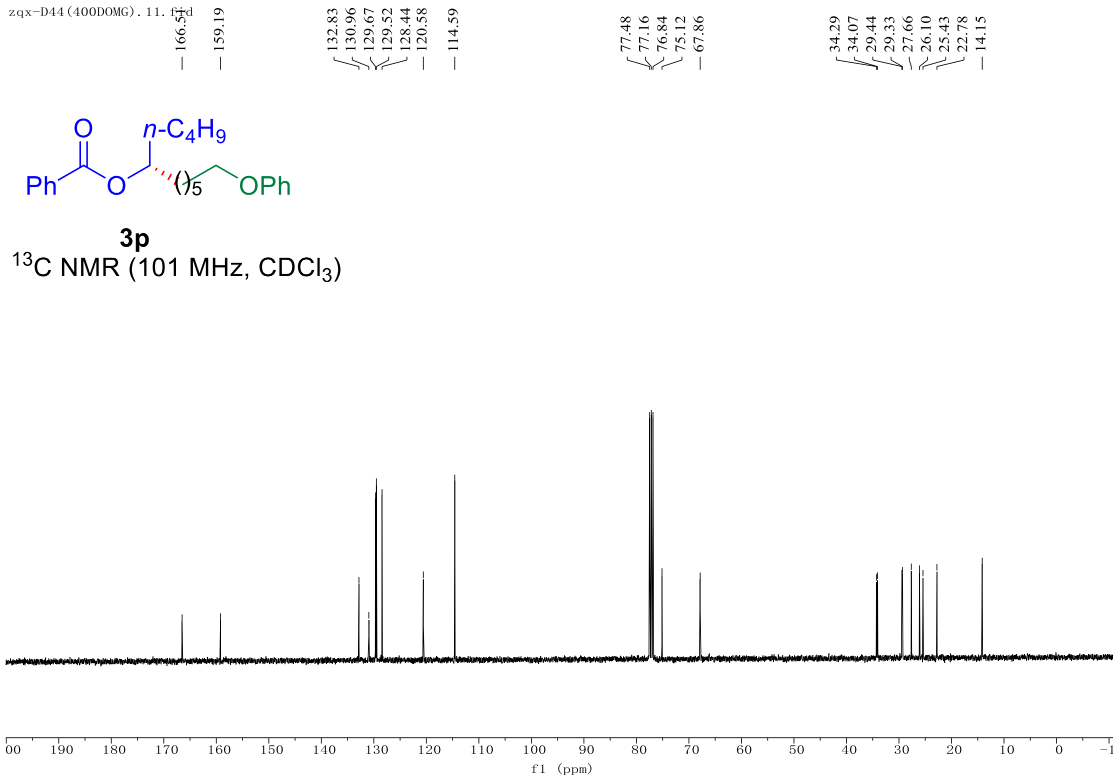

3p

^{13}C NMR (101 MHz, $CDCl_3$)

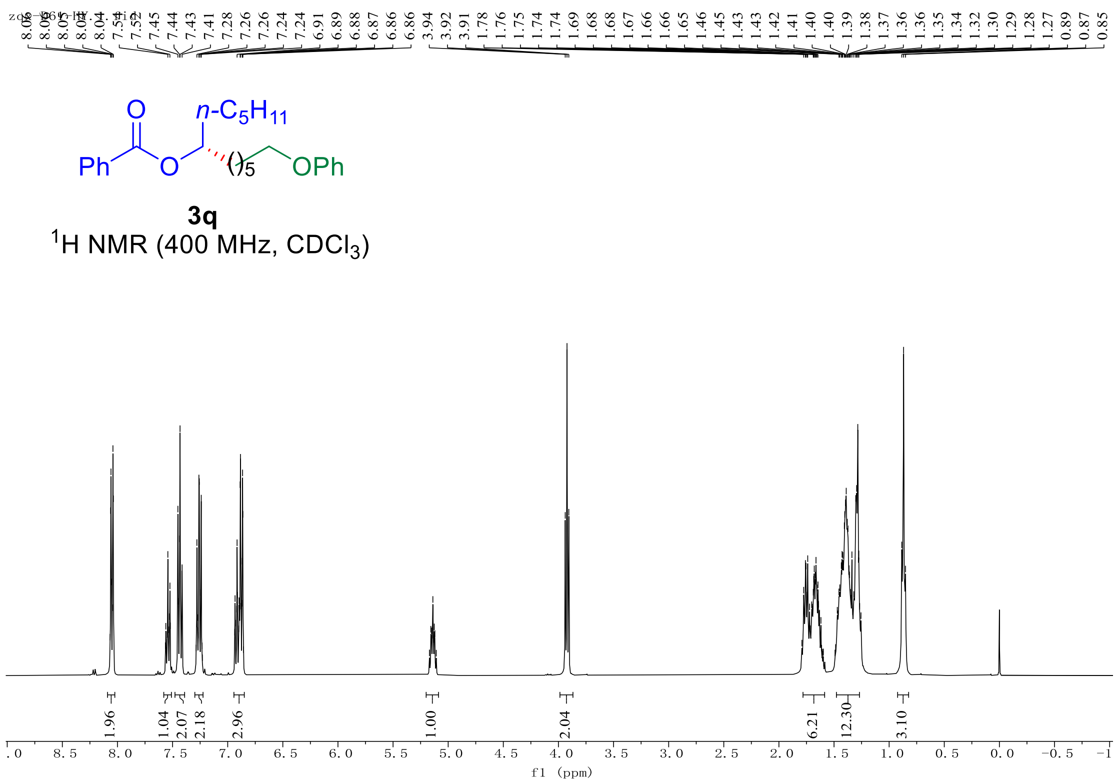
zqx-D61-H.1.fid
8.06
8.05
8.05
8.04
8.04
7.54
7.52
7.45
7.44
7.43
7.41
7.28
7.26
7.26
7.24
7.24
6.91
6.89
6.88
6.87
6.86
6.86
3.94
3.92
3.91
1.78
1.76
1.75
1.74
1.74
1.69
1.68
1.68
1.67
1.66
1.66
1.65
1.46
1.45
1.43
1.43
1.42
1.41
1.40
1.40
1.39
1.38
1.37
1.36
1.36
1.35
1.34
1.32
1.30
1.29
1.28
1.27
0.89
0.87
0.85
O
n-C5H11
Ph
O
()5
OPh
3q
1H NMR (400 MHz, CDCl3)
1.96
1.04
2.07
2.18
2.96
1.00
2.04
6.21
12.30
3.10
f1 (ppm)

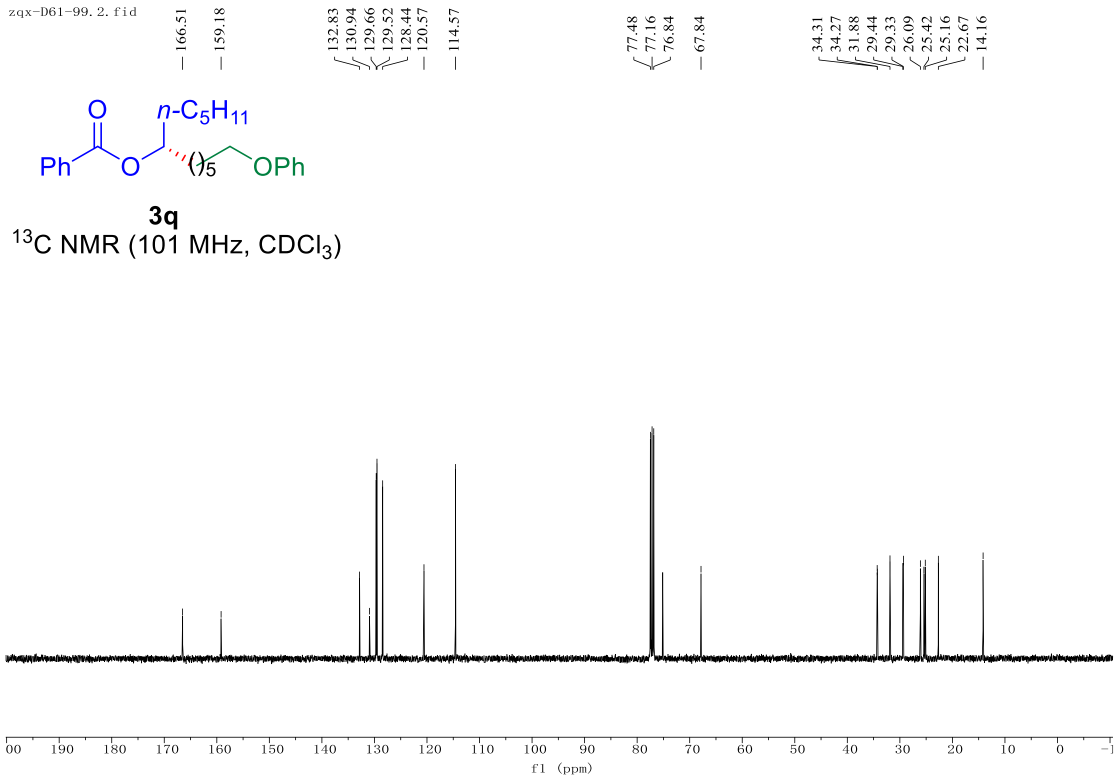
zqx-D61-99.2.fid
166.51
159.18
132.83
130.94
129.66
129.52
128.44
120.57
114.57
77.48
77.16
76.84
67.84
34.31
34.27
31.88
29.44
29.33
26.09
25.42
25.16
22.67
14.16
O
n-C5H11
Ph
O
()5
OPh
3q
13C NMR (101 MHz, CDCl3)
f1 (ppm)

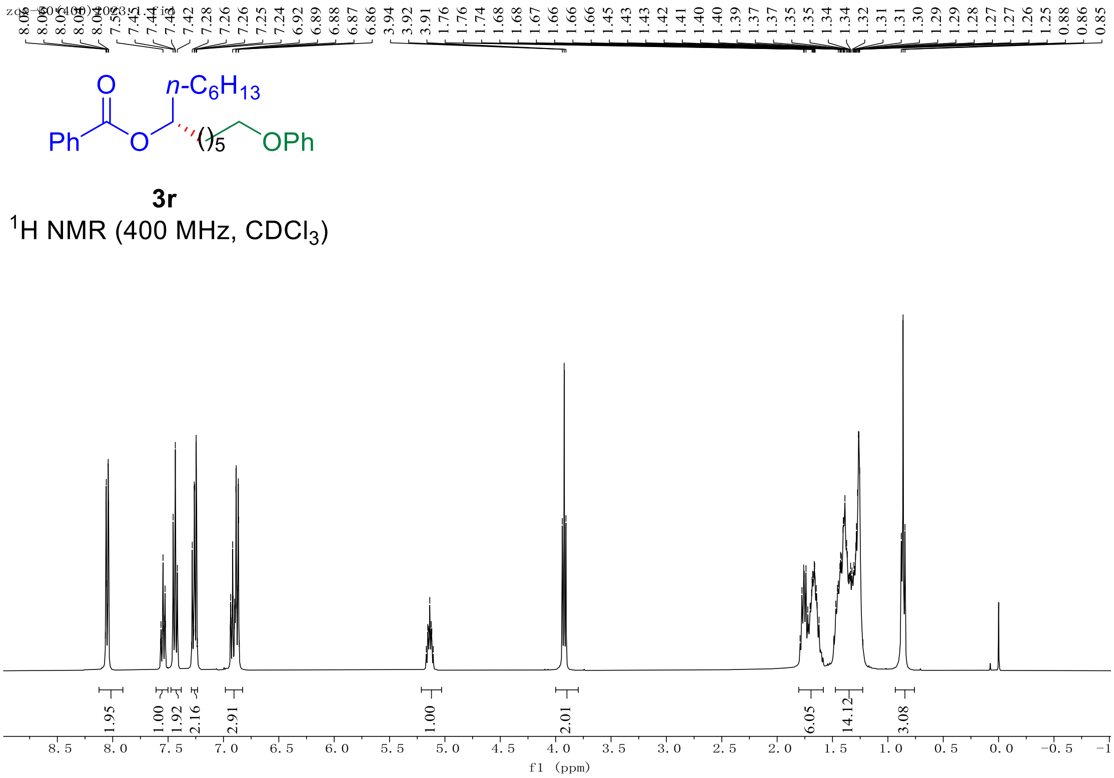

3r

^{1}H NMR (400 MHz, $CDCl_3$)

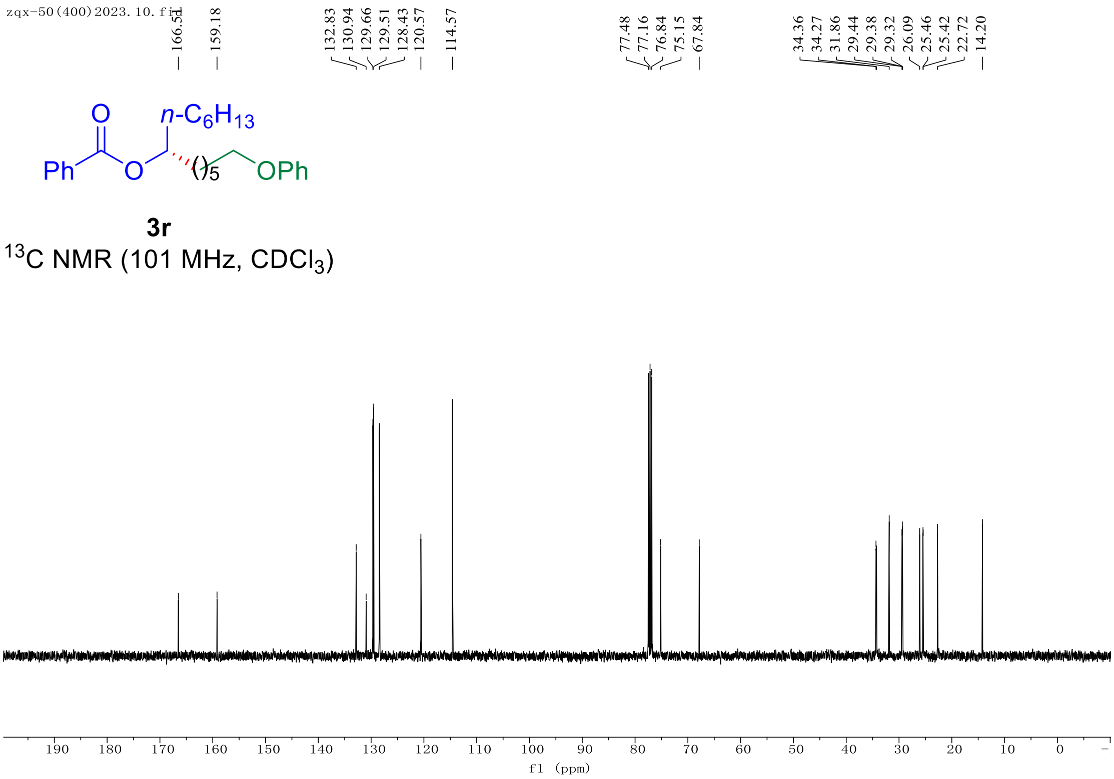

3r

^{13}C NMR (101 MHz, $CDCl_3$)

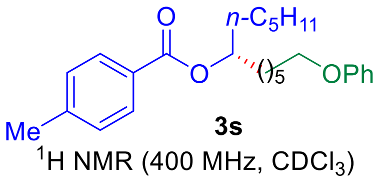

3s

^{1}H NMR (400 MHz, $CDCl_3$)

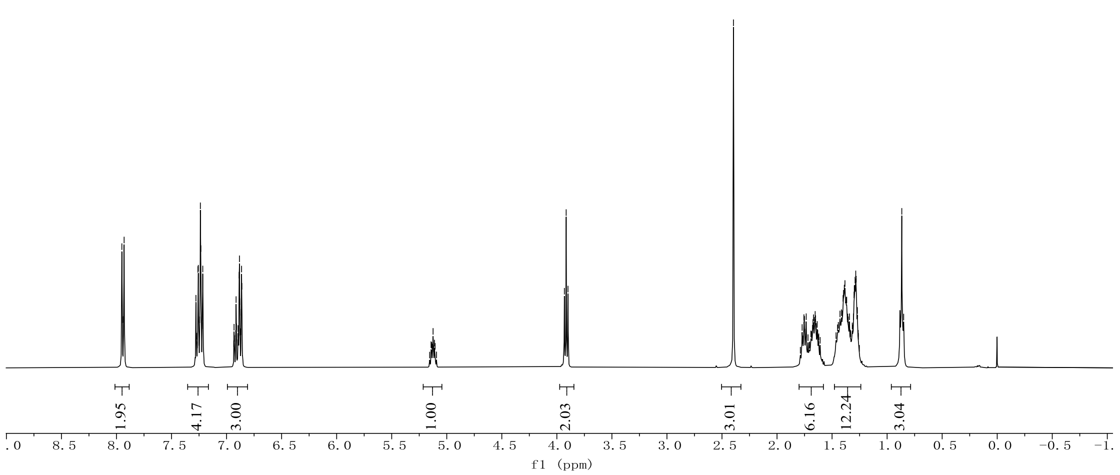

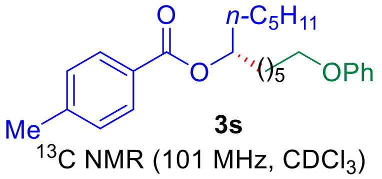

3s

^{13}C NMR (101 MHz, $CDCl_3$)

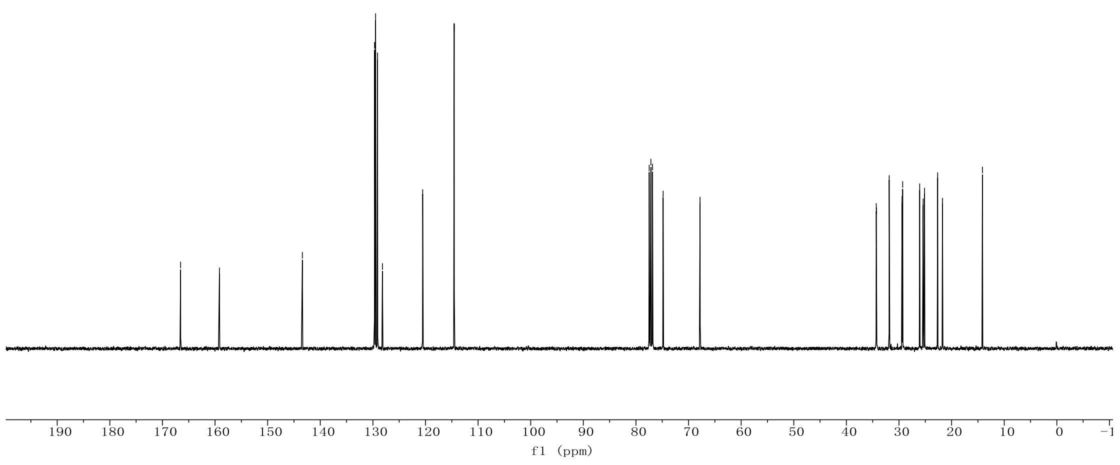

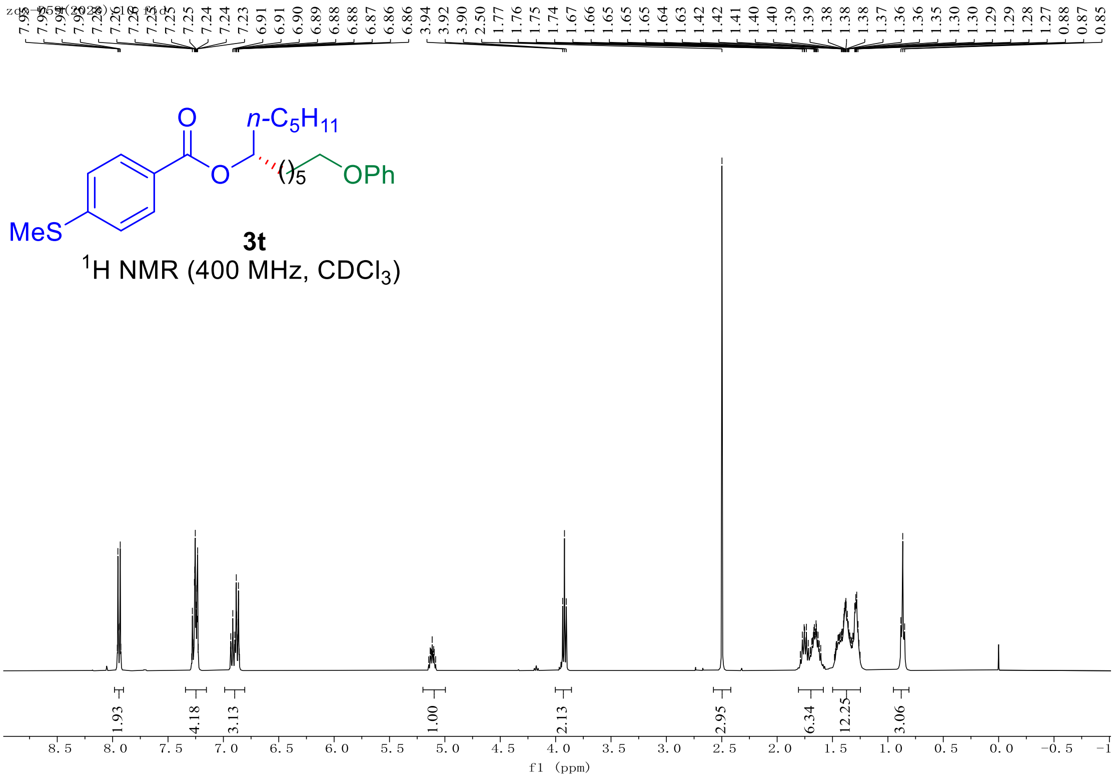
7.95 7.95 7.94 7.93 7.26 7.26 7.25 7.25 7.25 7.24 7.24 7.23 6.91 6.91 6.90 6.89 6.88 6.88 6.87 6.86 6.86 3.94 3.92 3.90 2.50 1.77 1.76 1.75 1.74 1.67 1.66 1.65 1.65 1.65 1.64 1.63 1.42 1.42 1.41 1.40 1.40 1.39 1.39 1.38 1.38 1.38 1.37 1.36 1.36 1.35 1.30 1.30 1.29 1.29 1.28 1.27 0.88 0.87 0.85
O
n-C5H11
O
()5
OPh
MeS
3t
1H NMR (400 MHz, CDCl3)
1.93 4.18 3.13 1.00 2.13 2.95 6.34 12.25 3.06
8.5 8.0 7.5 7.0 6.5 6.0 5.5 5.0 4.5 4.0 3.5 3.0 2.5 2.0 1.5 1.0 0.5 0.0 -0.5 -1
f1 (ppm)

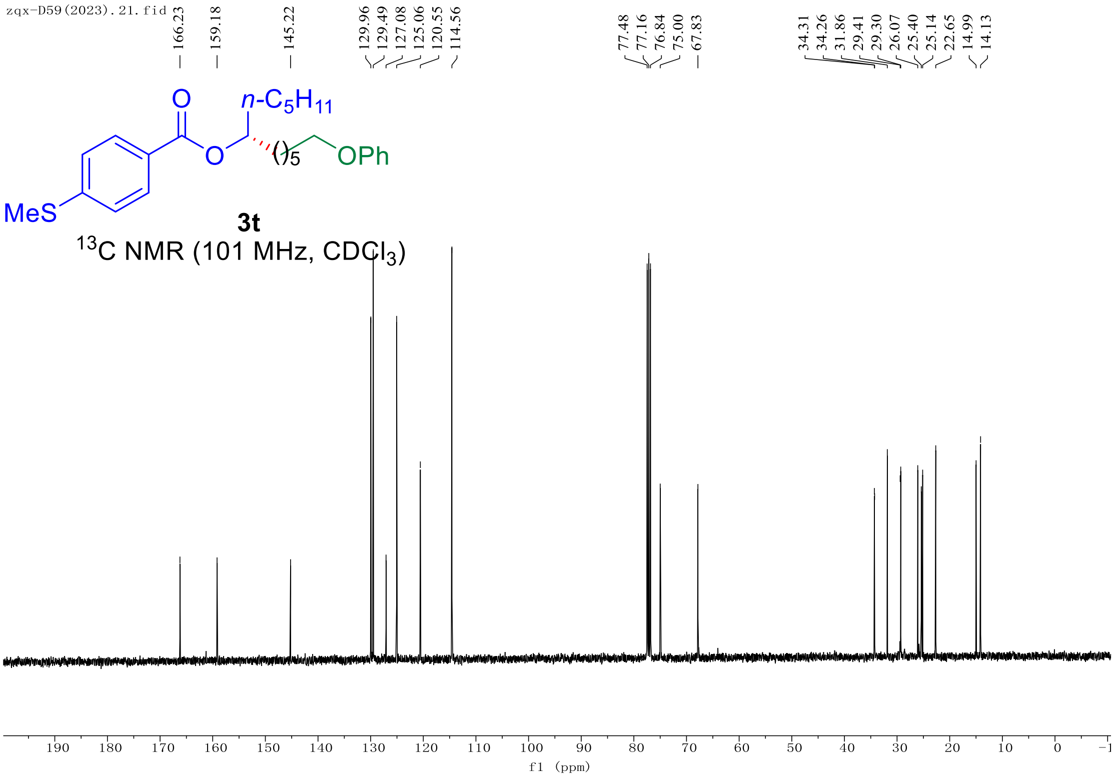
zqx-D59(2023).21.fid
166.23 159.18 145.22 129.96 129.49 127.08 125.06 120.55 114.56 77.48 77.16 76.84 75.00 67.83 34.31 34.26 31.86 29.41 29.30 26.07 25.40 25.14 22.65 14.99 14.13
O
n-C5H11
O
()5
OPh
MeS
3t
13C NMR (101 MHz, CDCl3)
190 180 170 160 150 140 130 120 110 100 90 80 70 60 50 40 30 20 10 0 -1
f1 (ppm)

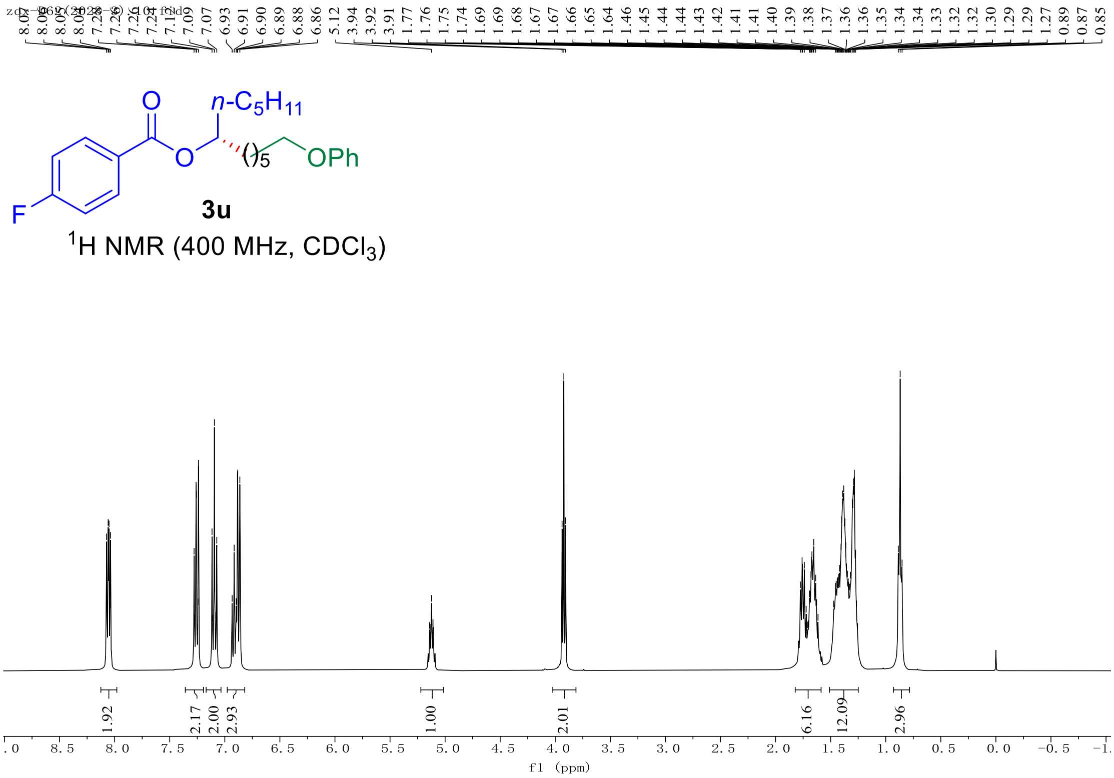

3u

^{1}H NMR (400 MHz, $CDCl_3$)

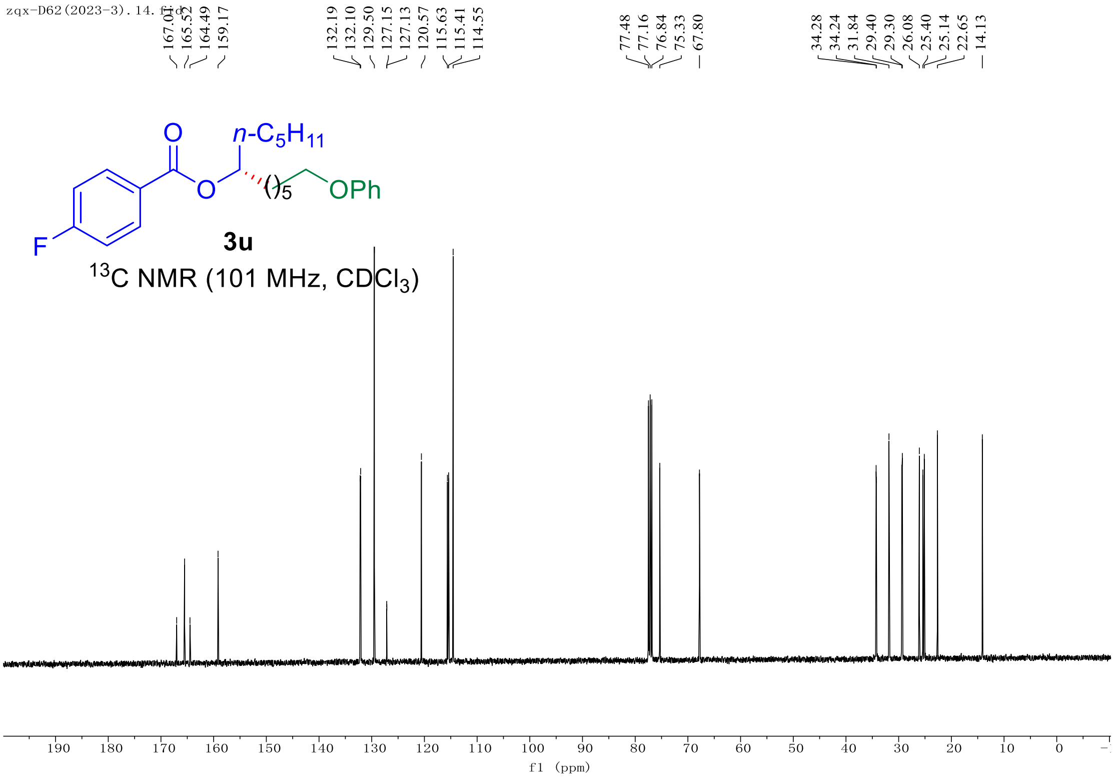

3u

^{13}C NMR (101 MHz, $CDCl_3$)

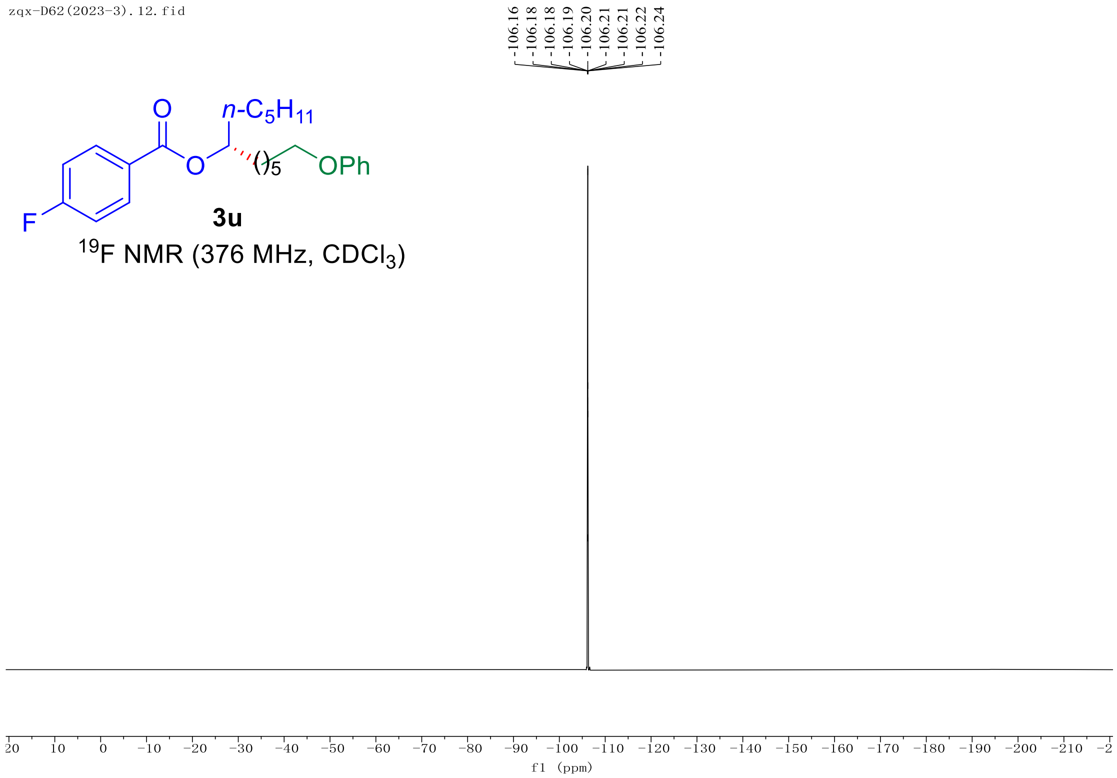
zqx-D62(2023-3).12.fid
-106.16
-106.18
-106.18
-106.19
-106.20
-106.21
-106.21
-106.22
-106.24
n-C5H11
O
O
F
OPh
5
3u
19F NMR (376 MHz, CDCl3)
f1 (ppm)

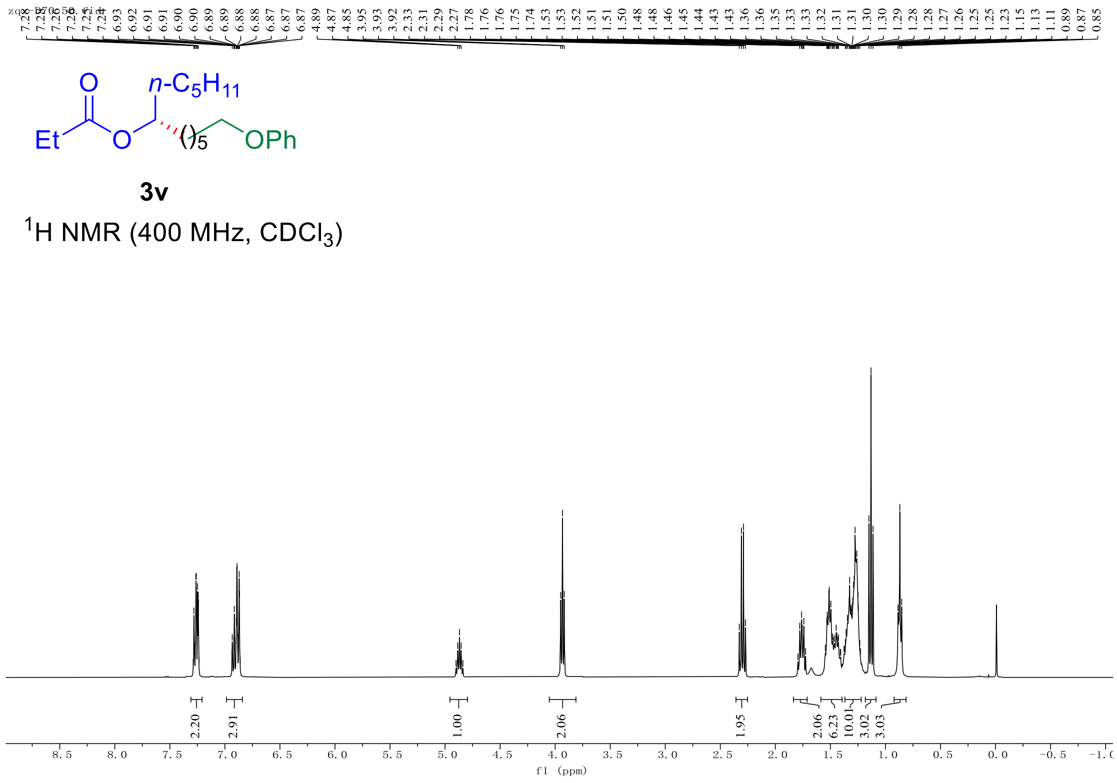
7.28
7.26
7.26
7.26
7.24
7.24
6.93
6.92
6.91
6.91
6.90
6.90
6.89
6.89
6.88
6.88
6.87
6.87
4.89
4.87
4.85
3.95
3.93
3.92
2.33
2.31
2.29
2.27
1.78
1.76
1.75
1.74
1.53
1.53
1.52
1.51
1.51
1.50
1.48
1.48
1.46
1.45
1.44
1.43
1.43
1.36
1.36
1.35
1.33
1.33
1.32
1.31
1.31
1.30
1.30
1.29
1.28
1.28
1.27
1.26
1.25
1.25
1.23
1.15
1.13
1.11
0.89
0.87
0.85
n-C5H11
O
Et
O
OPh
5
3v
1H NMR (400 MHz, CDCl3)
2.20
2.91
1.00
2.06
1.95
2.06
6.23
10.01
3.02
3.03
f1 (ppm)

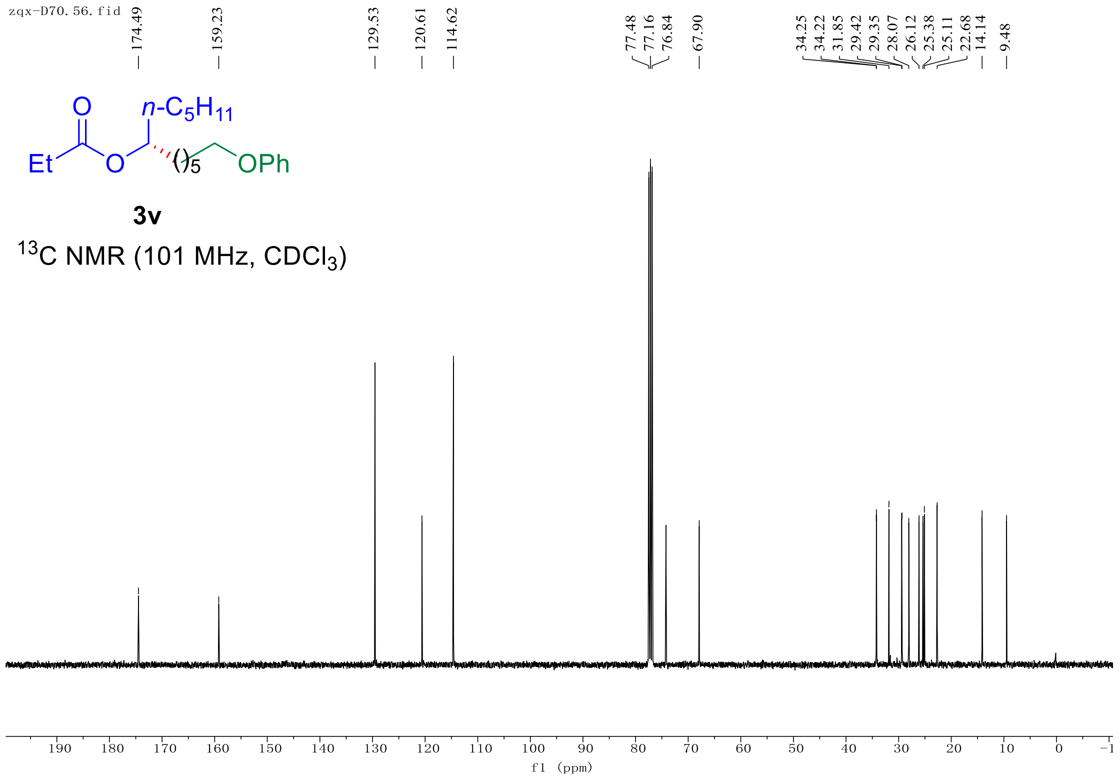
zqx-D70.56.fid
174.49
159.23
129.53
120.61
114.62
77.48
77.16
76.84
67.90
34.25
34.22
31.85
29.42
29.35
28.07
26.12
25.38
25.11
22.68
14.14
9.48
O
n-C5H11
Et
O
()5
OPh
3v
13C NMR (101 MHz, CDCl3)
f1 (ppm)

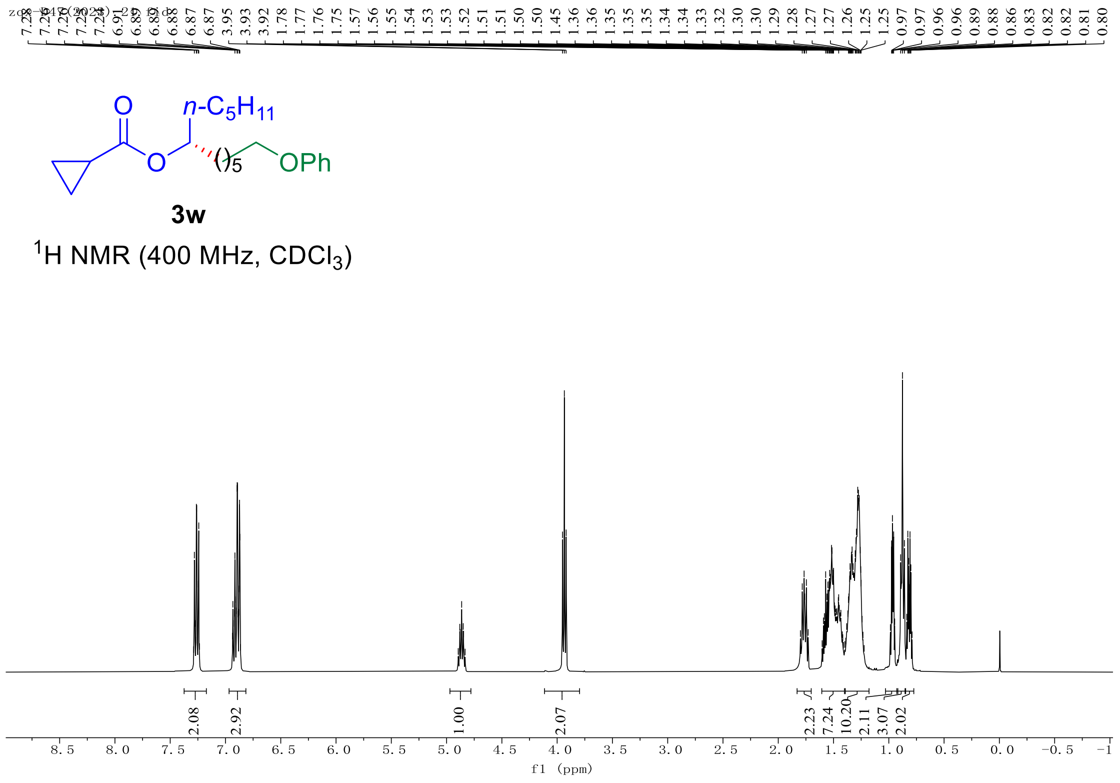
6.91
6.89
6.87
6.87
3.95
3.93
3.92
1.78
1.77
1.76
1.75
1.57
1.56
1.55
1.54
1.53
1.53
1.52
1.51
1.51
1.50
1.50
1.45
1.36
1.36
1.35
1.35
1.35
1.34
1.34
1.33
1.32
1.30
1.30
1.29
1.28
1.27
1.27
1.26
1.25
1.25
0.97
0.97
0.96
0.96
0.89
0.88
0.86
0.83
0.82
0.82
0.81
0.80
O
n-C5H11
O
()5
OPh
3w
1H NMR (400 MHz, CDCl3)
2.08
2.92
1.00
2.07
2.23
7.24
10.20
2.11
3.07
2.02
f1 (ppm)

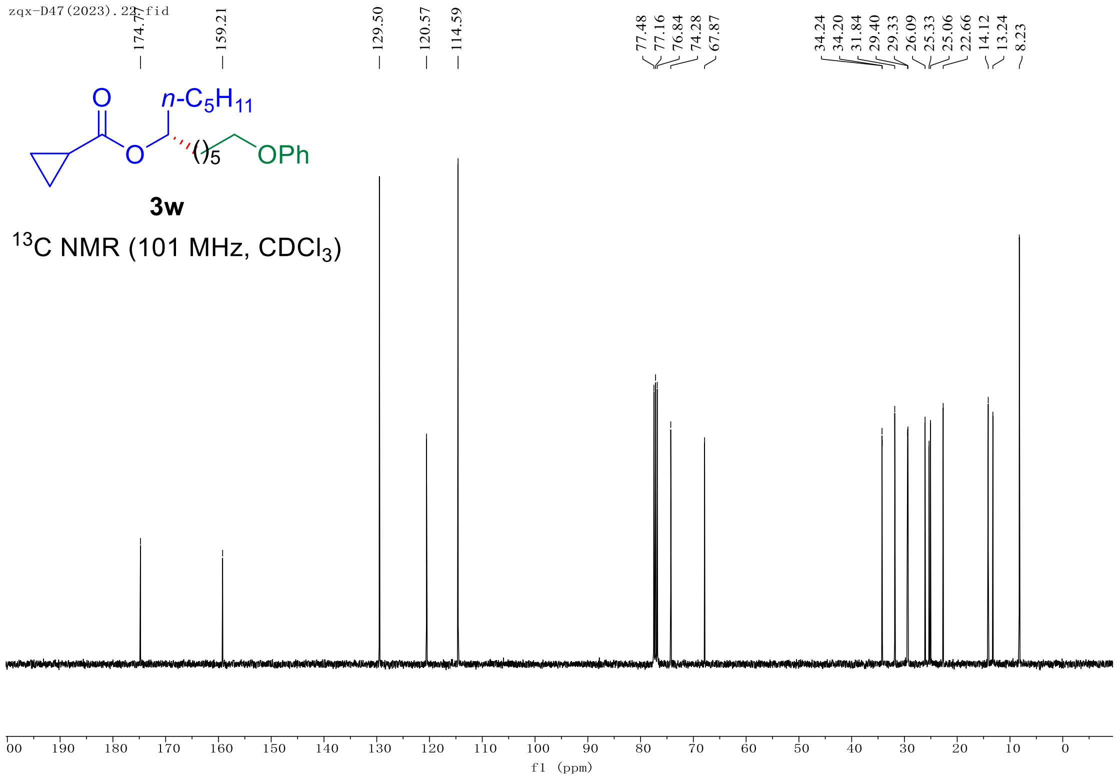
zqx-D47(2023).22.fid
3w
^{13}C NMR (101 MHz, CDCl$_3$)
f1 (ppm)

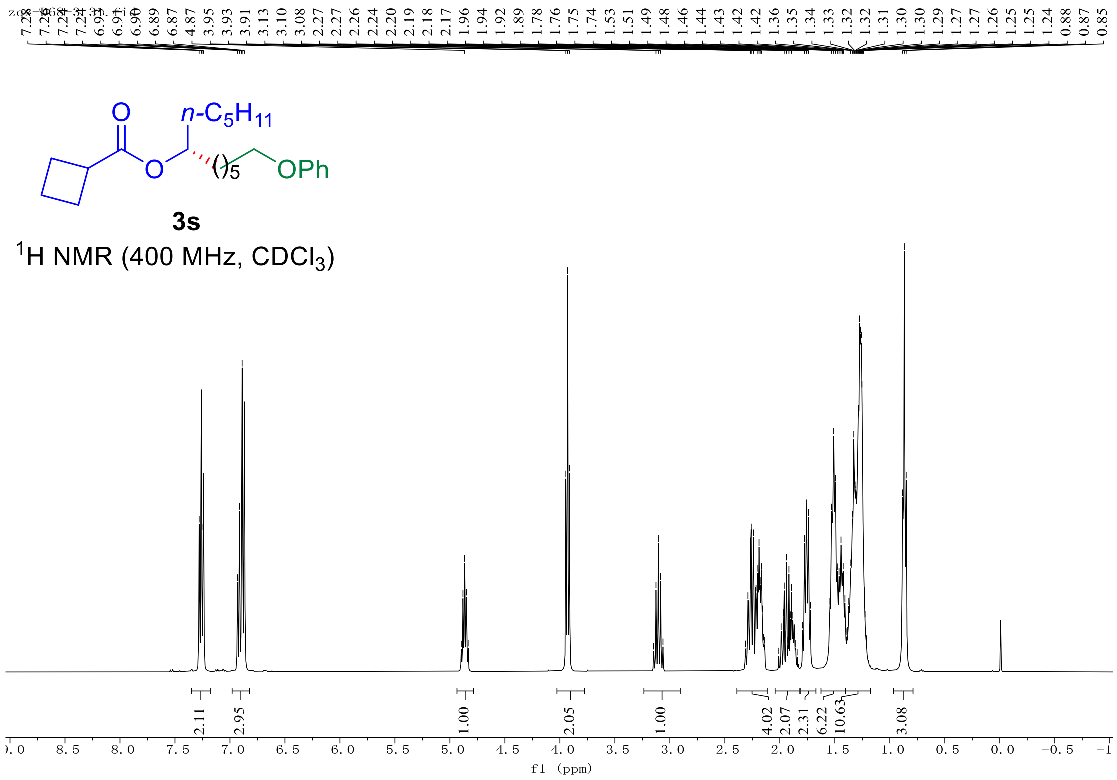
3s
^{1}H NMR (400 MHz, CDCl$_3$)
f1 (ppm)

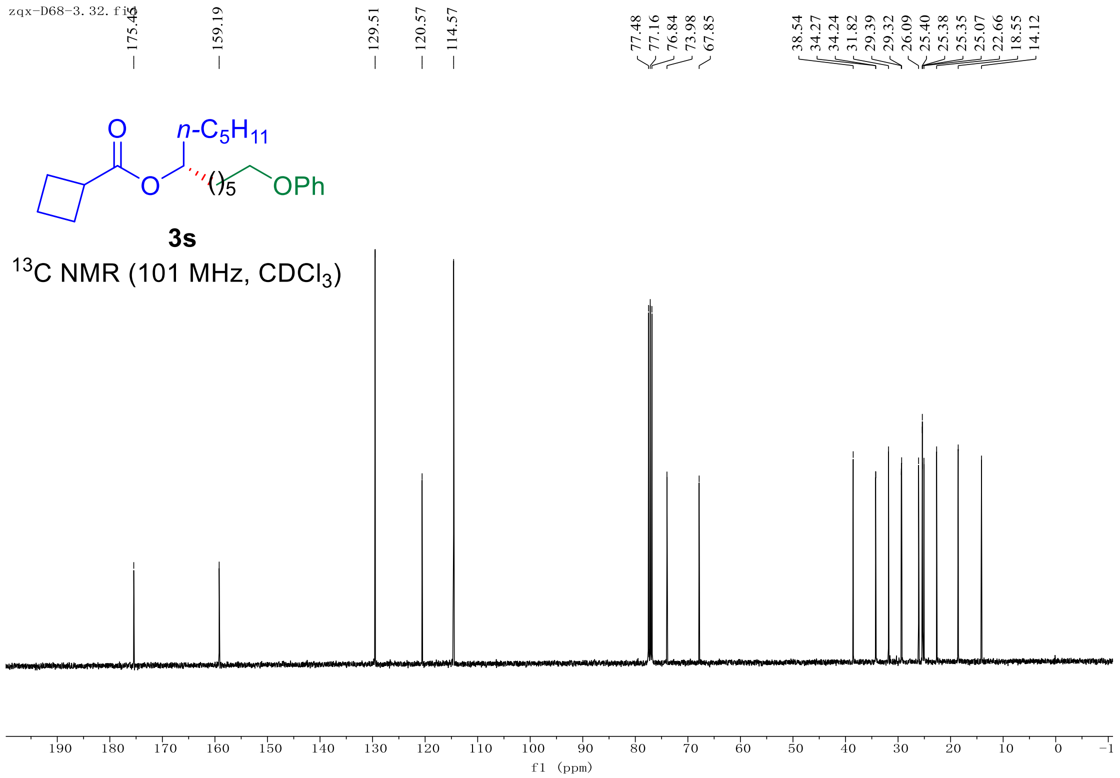
zqx-D68-3.32.fid
175.4
159.19
129.51
120.57
114.57
77.48
77.16
76.84
73.98
67.85
38.54
34.27
34.24
31.82
29.39
29.32
26.09
25.40
25.38
25.35
25.07
22.66
18.55
14.12
O
n-C5H11
O
()5
OPh
3s
13C NMR (101 MHz, CDCl3)
f1 (ppm)

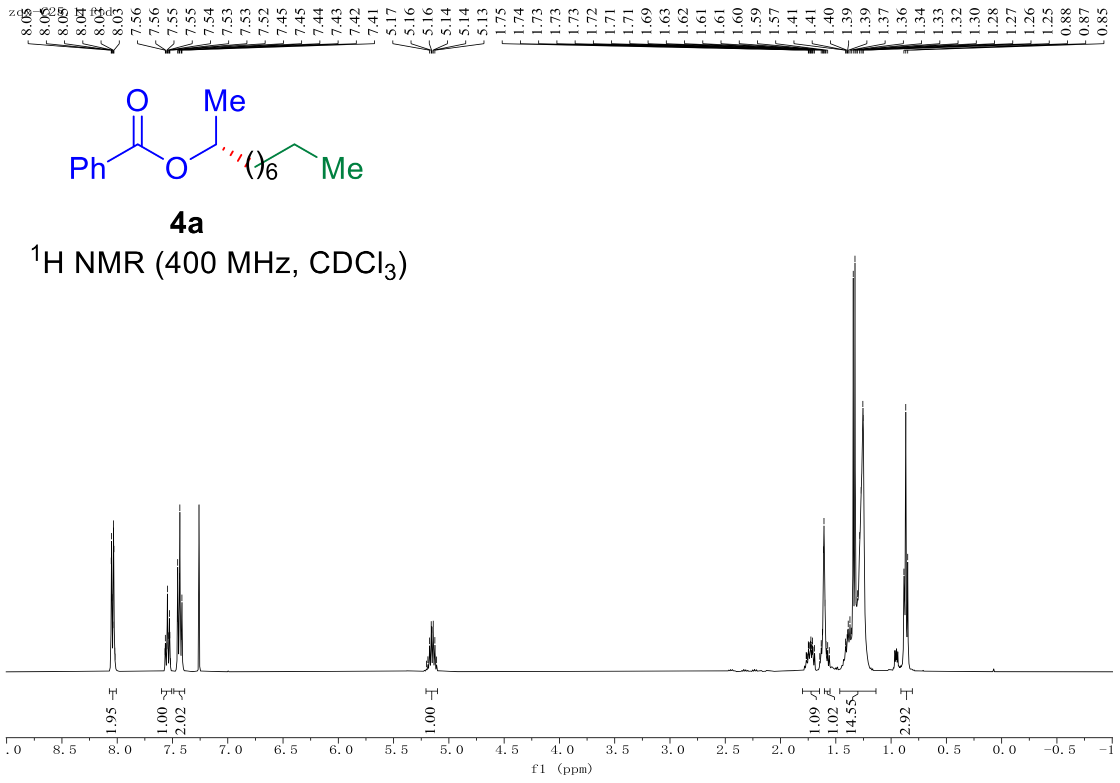
7.56
7.56
7.55
7.55
7.54
7.53
7.53
7.52
7.45
7.45
7.44
7.43
7.42
7.41
5.17
5.16
5.16
5.14
5.14
5.13
1.75
1.74
1.73
1.73
1.72
1.71
1.71
1.69
1.63
1.62
1.61
1.61
1.60
1.59
1.57
1.41
1.41
1.40
1.39
1.39
1.37
1.36
1.34
1.33
1.32
1.30
1.28
1.27
1.26
1.25
0.88
0.87
0.85
O
Me
Ph
O
()6
Me
4a
1H NMR (400 MHz, CDCl3)
1.95
1.00
2.02
1.00
1.09
1.02
14.55
2.92
f1 (ppm)

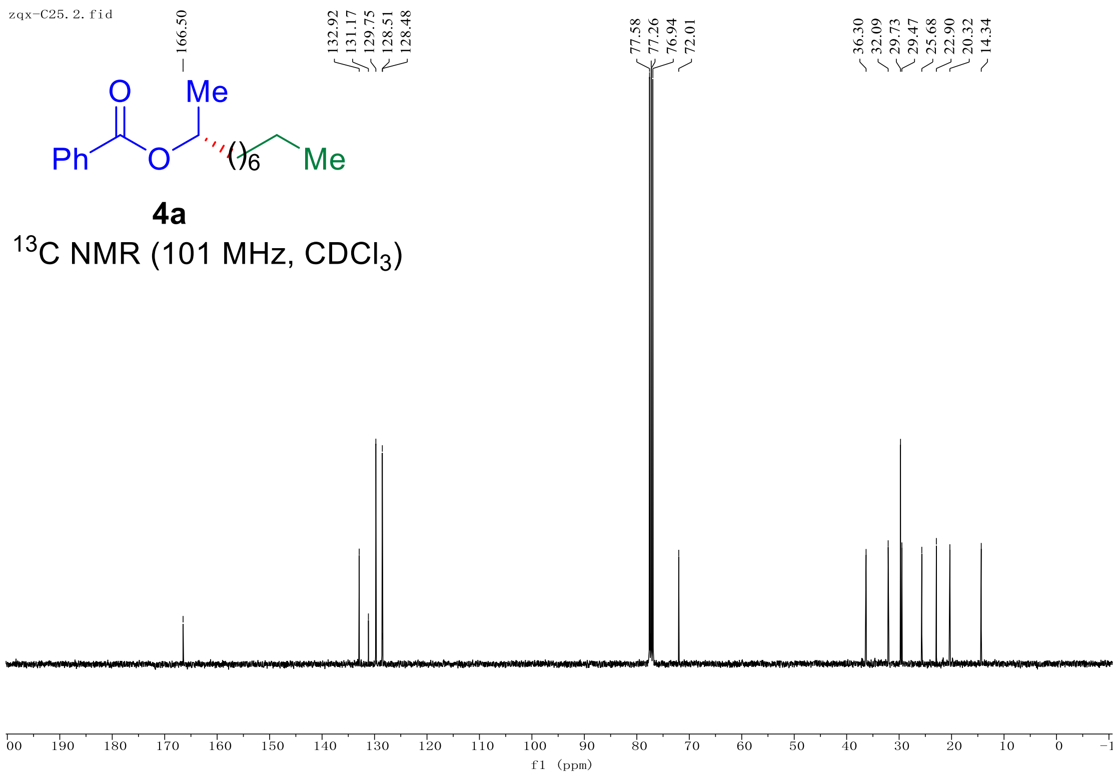

zqx-C25.2.fid
166.50
132.92
131.17
129.75
128.51
128.48
77.58
77.26
76.94
72.01
36.30
32.09
29.73
29.47
25.68
22.90
20.32
14.34
O
Me
Ph
O
6
Me
4a
13C NMR (101 MHz, CDCl3)
f1 (ppm)

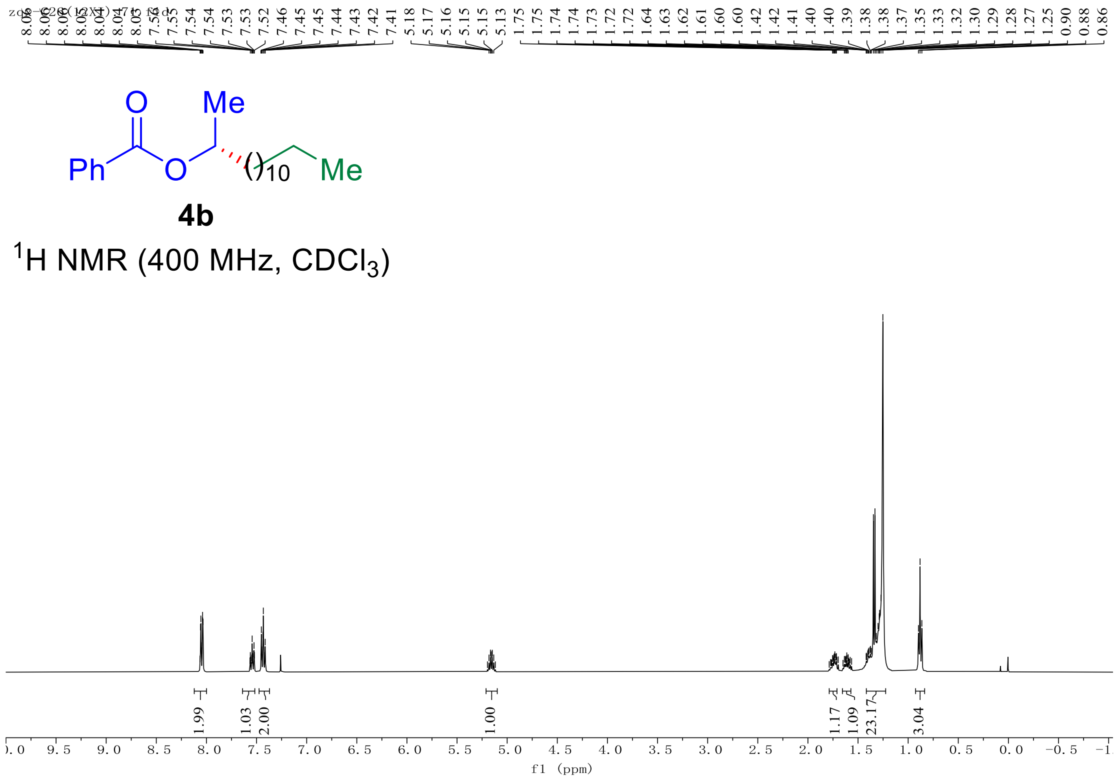

7.55
7.54
7.54
7.53
7.53
7.52
7.46
7.45
7.45
7.44
7.43
7.42
7.41
5.18
5.17
5.16
5.15
5.15
5.13
1.75
1.75
1.74
1.74
1.73
1.72
1.72
1.64
1.63
1.62
1.61
1.60
1.60
1.42
1.42
1.41
1.40
1.40
1.39
1.38
1.38
1.37
1.35
1.33
1.32
1.30
1.29
1.28
1.27
1.25
0.90
0.88
0.86
O
Me
Ph
O
10
Me
4b
1H NMR (400 MHz, CDCl3)
1.99
1.03
2.00
1.00
1.17
1.09
23.17
3.04
f1 (ppm)

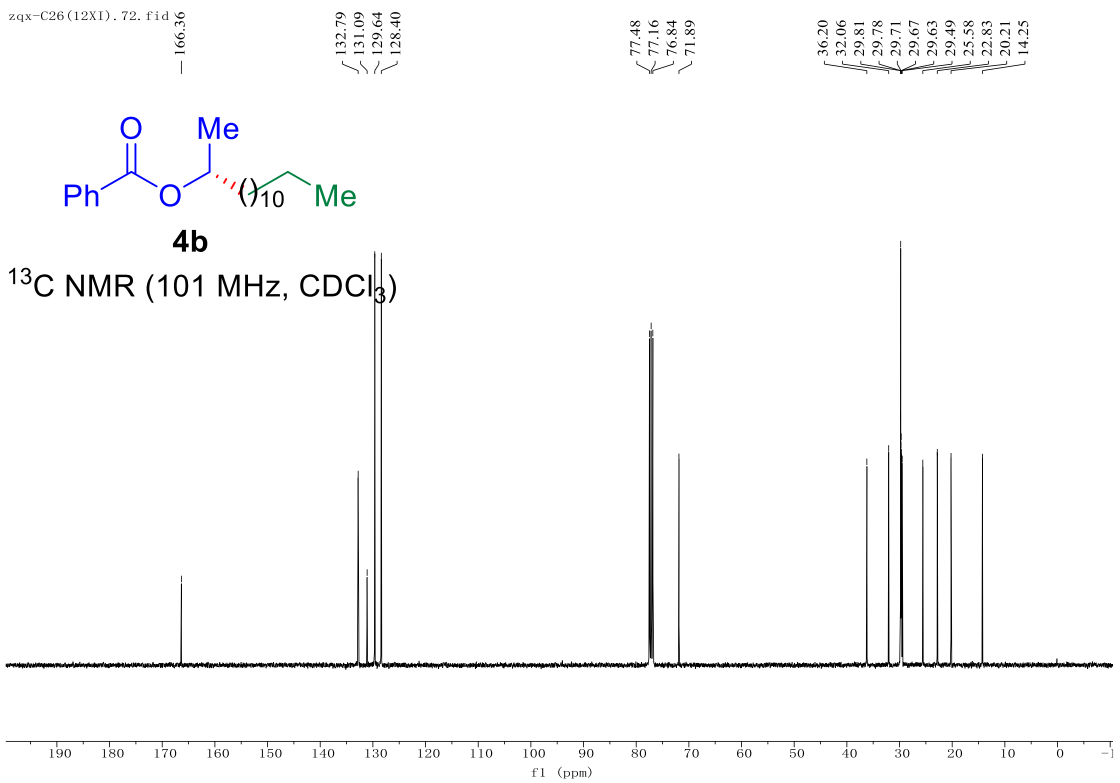
zqx-C26(12XI).72.fid
166.36
132.79
131.09
129.64
128.40
77.48
77.16
76.84
71.89
36.20
32.06
29.81
29.78
29.71
29.67
29.63
29.49
25.58
22.83
20.21
14.25
O
Me
Ph
O
10
Me
4b
13C NMR (101 MHz, CDCl3)
190 180 170 160 150 140 130 120 110 100 90 80 70 60 50 40 30 20 10 0
f1 (ppm)

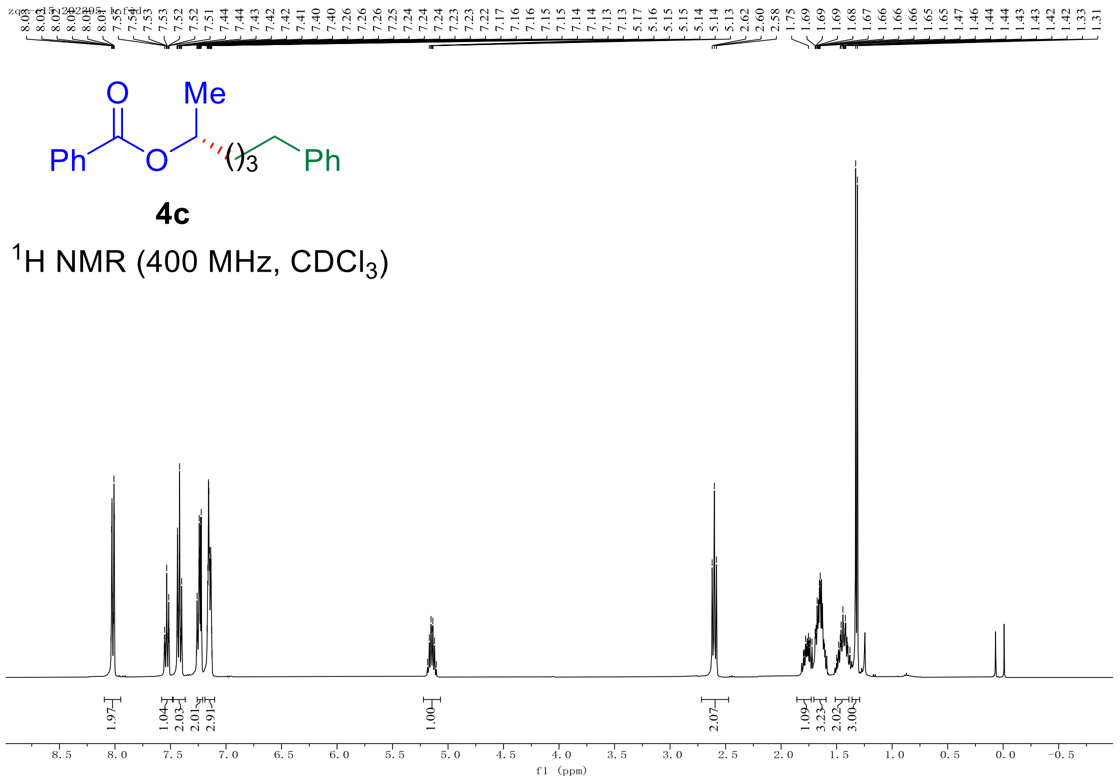
8.03 8.03 8.02 8.02 8.01 8.01 7.55 7.54 7.53 7.53 7.52 7.52 7.51 7.44 7.43 7.42 7.42 7.41 7.40 7.40 7.26 7.26 7.25 7.24 7.24 7.24 7.23 7.23 7.22 7.17 7.16 7.16 7.15 7.15 7.14 7.14 7.13 7.13 5.17 5.16 5.15 5.15 5.14 5.14 5.13 2.62 2.60 2.58 1.75 1.69 1.69 1.69 1.68 1.67 1.66 1.66 1.65 1.65 1.47 1.46 1.44 1.44 1.43 1.42 1.42 1.33 1.31
O
Me
Ph
O
3
Ph
4c
1H NMR (400 MHz, CDCl3)
1.97
1.04
2.03
2.01
2.91
1.00
2.07
1.09
3.23
2.02
3.00
8.5 8.0 7.5 7.0 6.5 6.0 5.5 5.0 4.5 4.0 3.5 3.0 2.5 2.0 1.5 1.0 0.5 0.0 -0.5
f1 (ppm)

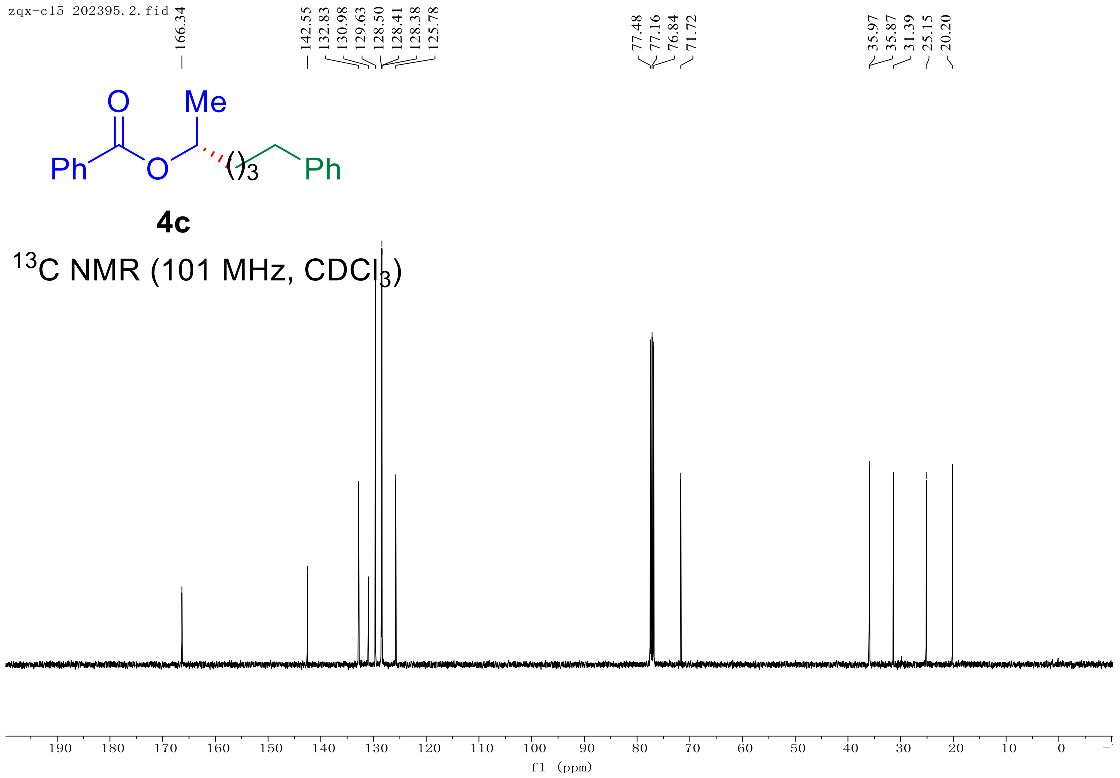
zqx-c15 202395.2.fid
166.34
142.55
132.83
130.98
129.63
128.50
128.41
128.38
125.78
77.48
77.16
76.84
71.72
35.97
35.87
31.39
25.15
20.20
O
Me
Ph
O
()3
Ph
4c
13C NMR (101 MHz, CDCl3)
f1 (ppm)

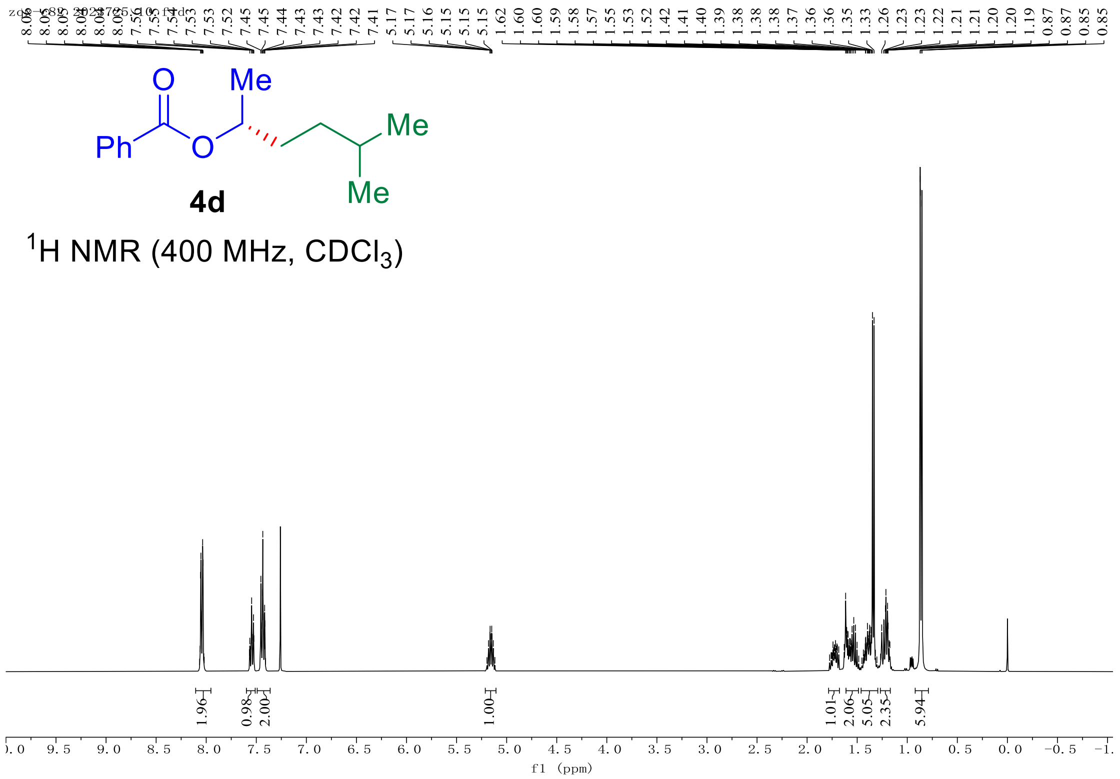
7.53
7.53
7.52
7.45
7.45
7.44
7.43
7.43
7.42
7.42
7.41
5.17
5.17
5.16
5.15
5.15
5.15
1.62
1.60
1.60
1.59
1.58
1.57
1.55
1.53
1.52
1.42
1.41
1.40
1.39
1.38
1.38
1.38
1.37
1.36
1.36
1.35
1.33
1.26
1.23
1.23
1.22
1.21
1.21
1.20
1.19
0.87
0.85
0.85
O
Me
Ph
O
Me
Me
4d
1H NMR (400 MHz, CDCl3)
1.96
0.98
2.00
1.00
1.01
2.06
5.05
2.35
5.94
f1 (ppm)

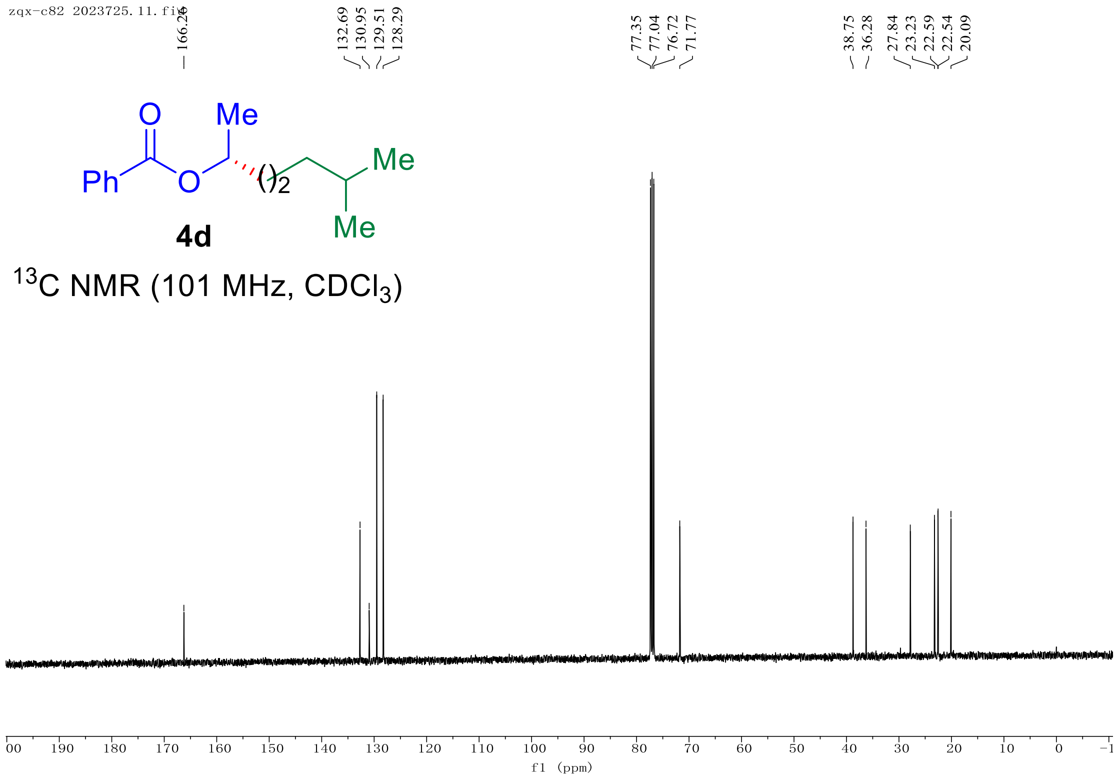
zqx-c82 2023725.11.fid
166.26
132.69
130.95
129.51
128.29
77.35
77.04
76.72
71.77
38.75
36.28
27.84
23.23
22.59
22.54
20.09
O
Me
Ph
O
()2
Me
Me
4d
^{13}C NMR (101 MHz, CDCl$_3$)
f1 (ppm)

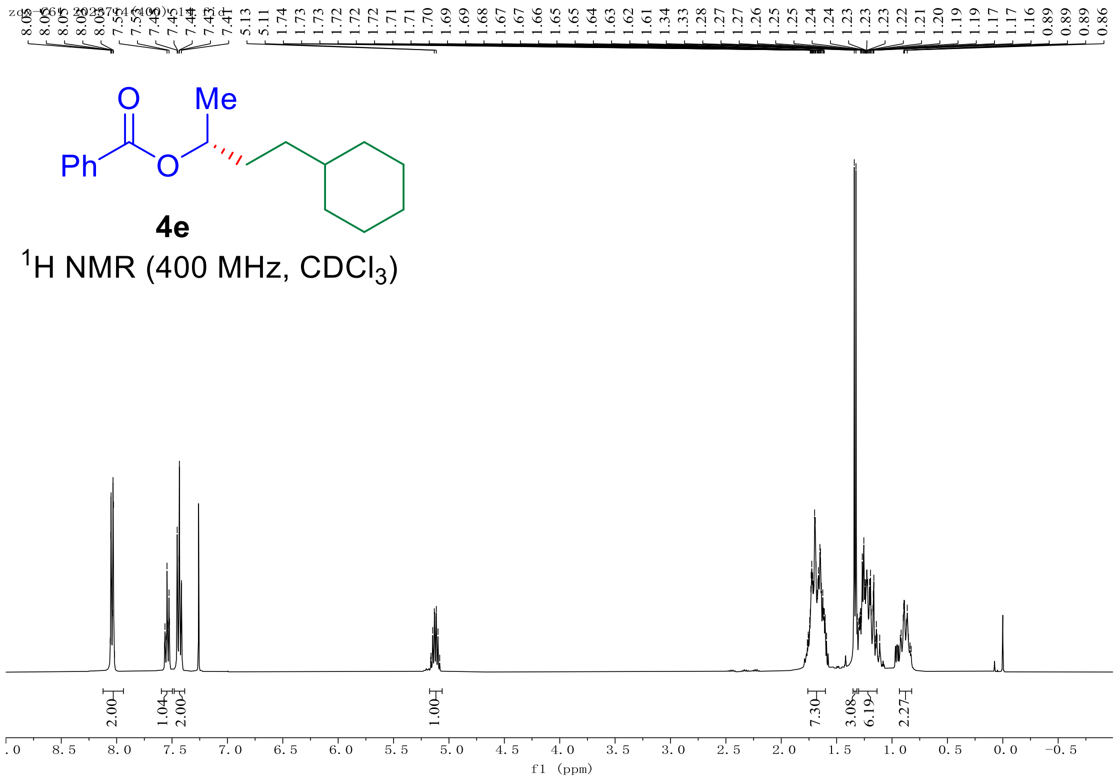
5.13
5.11
1.74
1.73
1.73
1.72
1.72
1.72
1.71
1.71
1.70
1.69
1.69
1.68
1.67
1.67
1.66
1.65
1.65
1.64
1.63
1.62
1.61
1.34
1.33
1.28
1.27
1.27
1.26
1.25
1.25
1.24
1.24
1.23
1.23
1.23
1.22
1.21
1.20
1.19
1.19
1.17
1.16
0.89
0.89
0.89
0.86
O
Me
Ph
O
4e
^{1}H NMR (400 MHz, CDCl$_3$)
2.00
1.04
2.00
1.00
7.30
3.08
6.19
2.27
f1 (ppm)

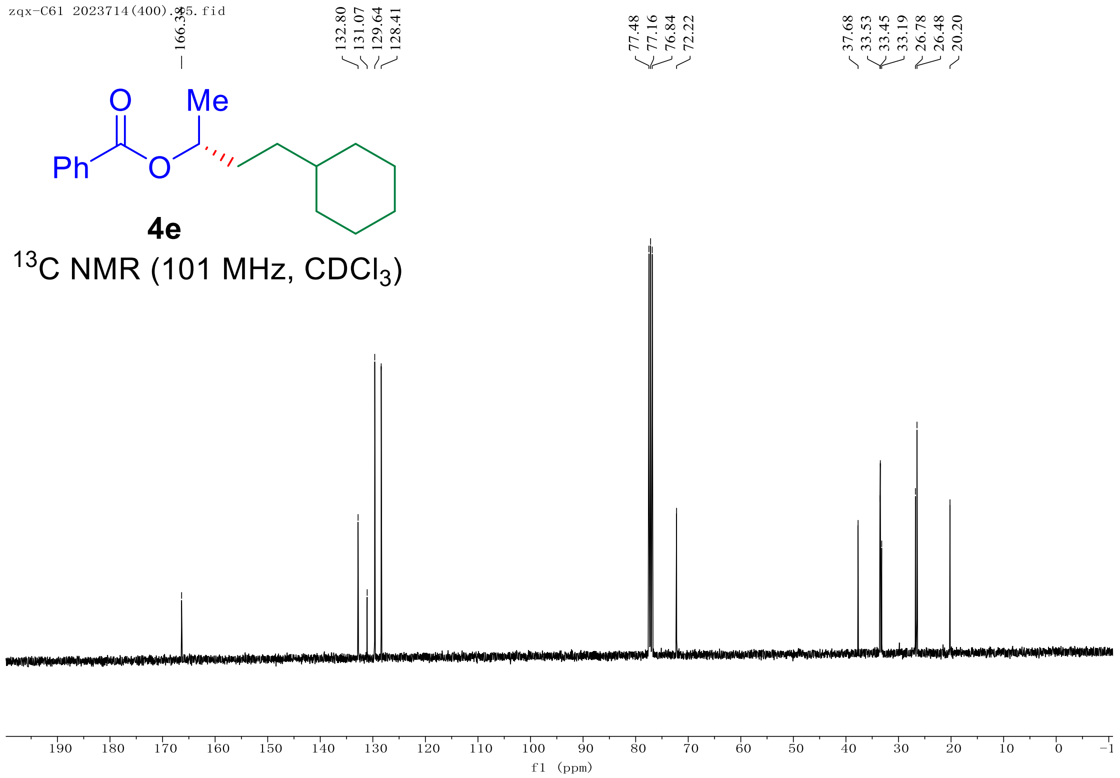

132.80
131.07
129.64
128.41
77.48
77.16
76.84
72.22
37.68
33.53
33.45
33.19
26.78
26.48
20.20
O
Me
Ph
O
4e
^{13}C NMR (101 MHz, CDCl$_3$)
f1 (ppm)

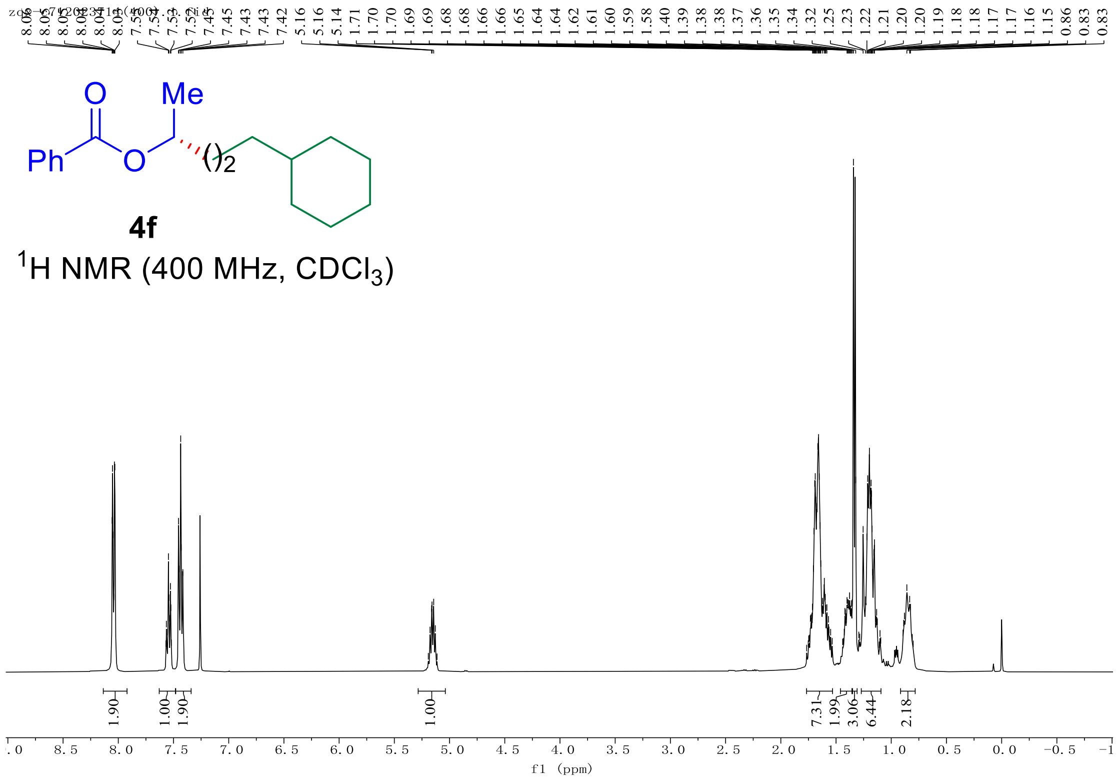

O
Me
Ph
O
()$_2$
4f
^{1}H NMR (400 MHz, CDCl$_3$)
1.90
1.00
1.90
1.00
7.31
1.99
3.06
6.44
2.18
f1 (ppm)

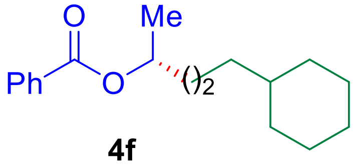

4f

^{13}C NMR (101 MHz, $CDCl_3$)

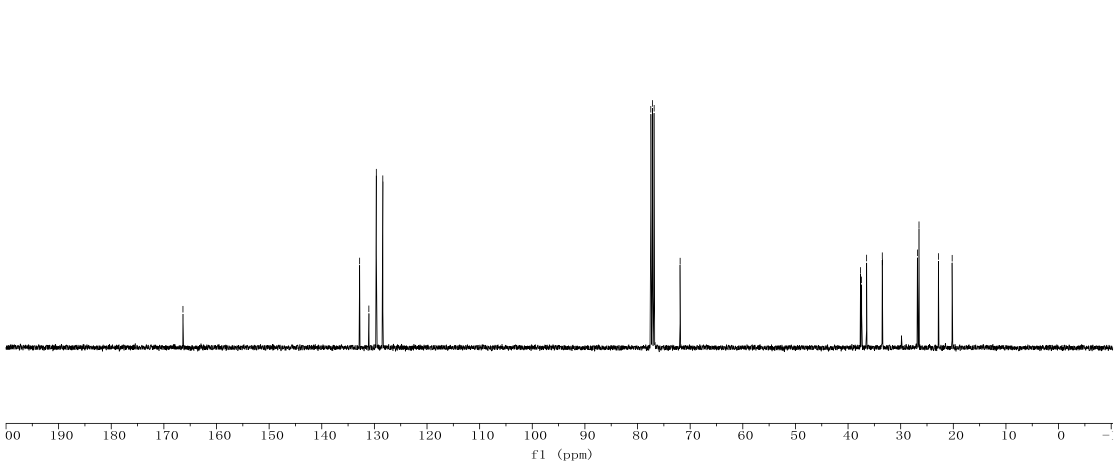

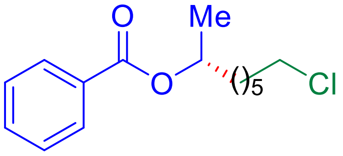

4g

^{1}H NMR (400 MHz, $CDCl_3$)

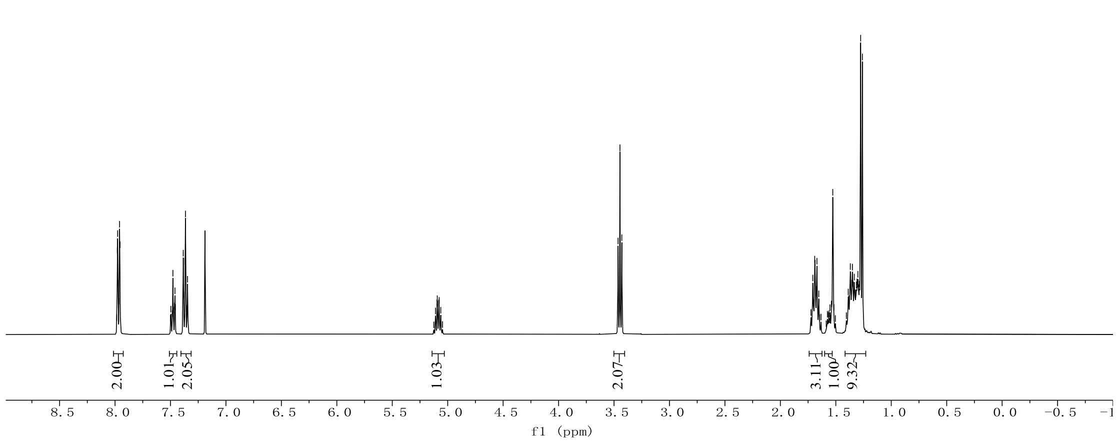

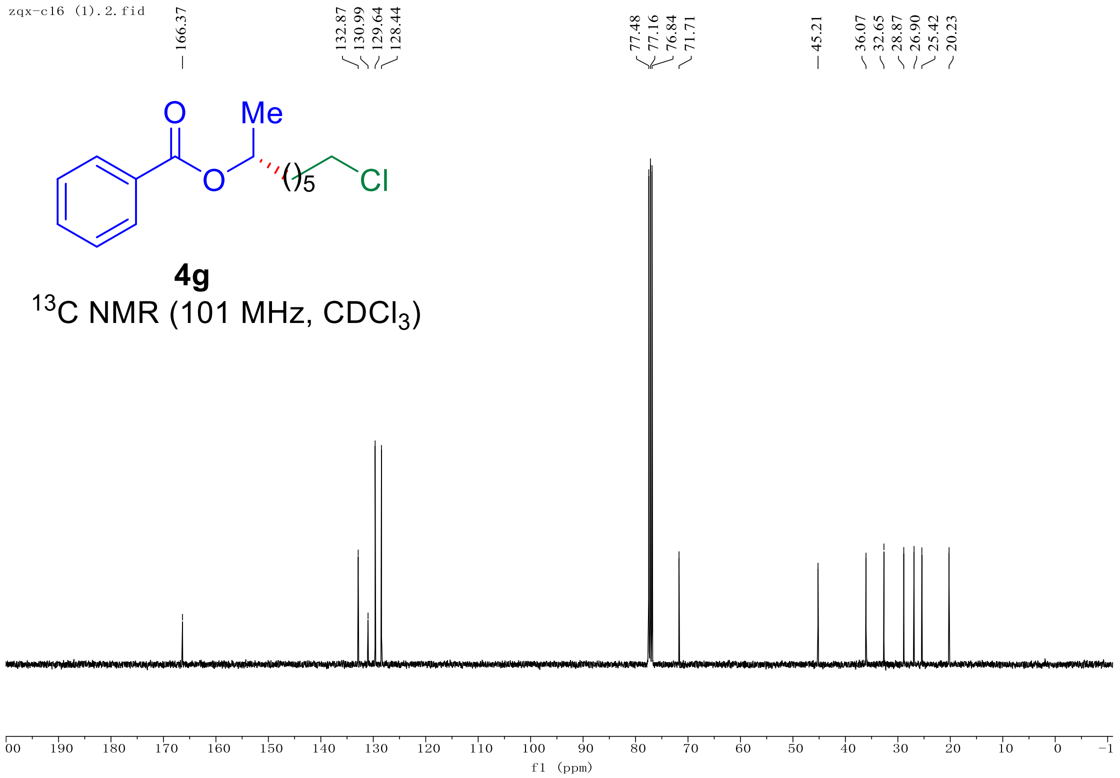
zqx-c16 (1).2.fid
166.37
132.87
130.99
129.64
128.44
77.48
77.16
76.84
71.71
45.21
36.07
32.65
28.87
26.90
25.42
20.23
O
Me
O
()5
Cl
4g
13C NMR (101 MHz, CDCl3)
f1 (ppm)

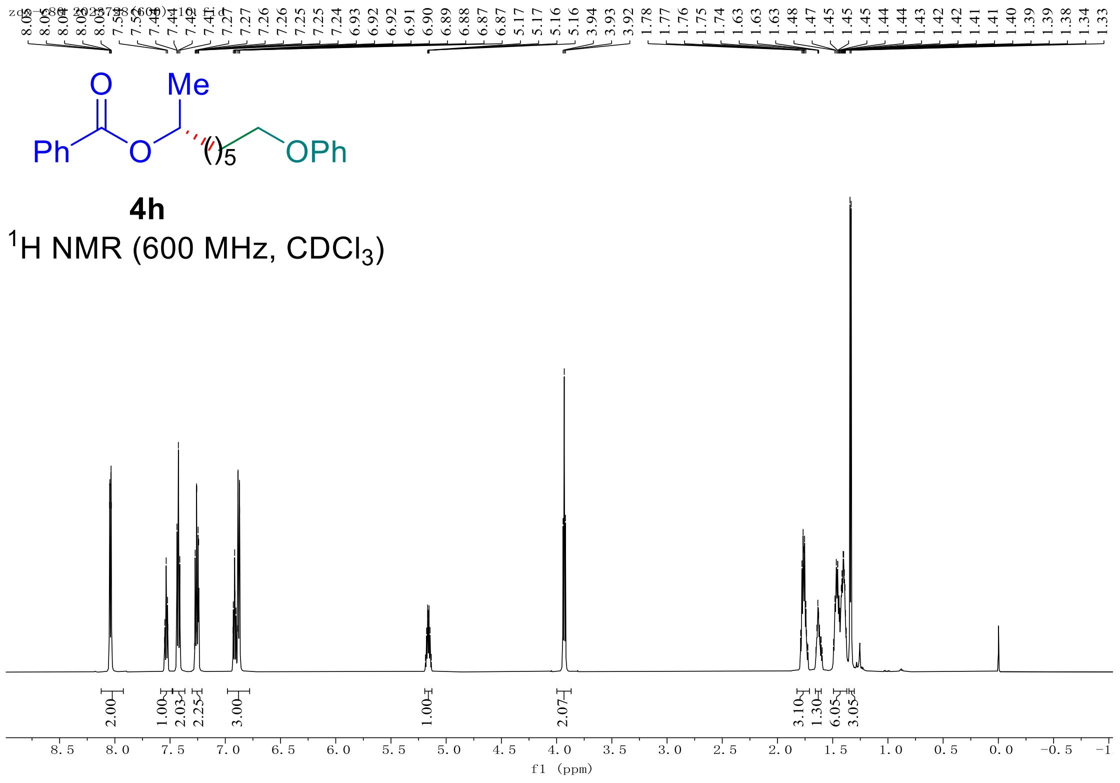
O
Me
Ph
O
()5
OPh
4h
1H NMR (600 MHz, CDCl3)
2.00
1.00
2.03
2.25
3.00
1.00
2.07
3.10
1.30
6.05
3.05
f1 (ppm)

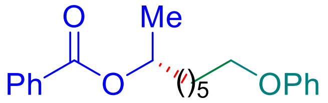

4h

^{13}C NMR (151 MHz, $CDCl_3$)

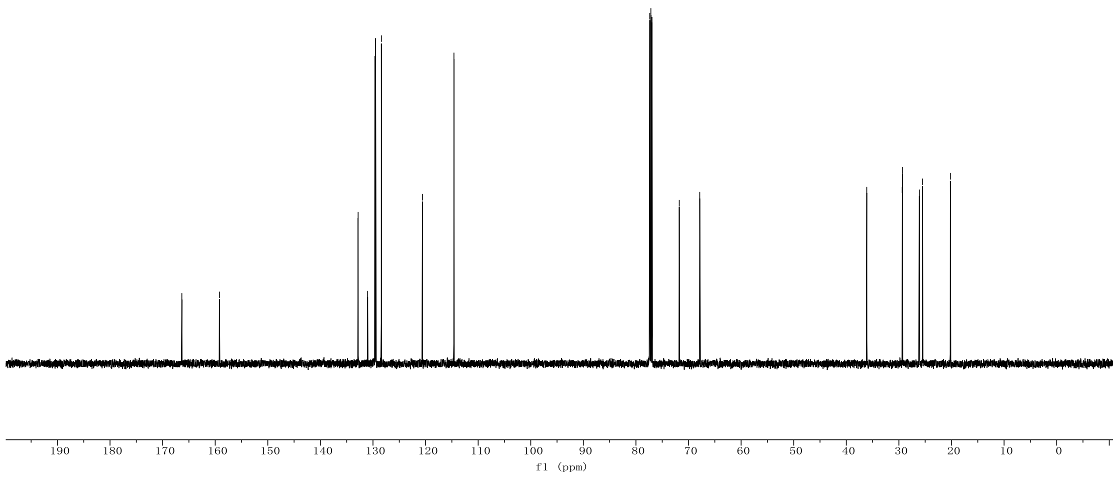

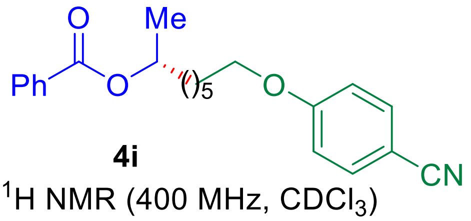

4i

^{1}H NMR (400 MHz, $CDCl_3$)

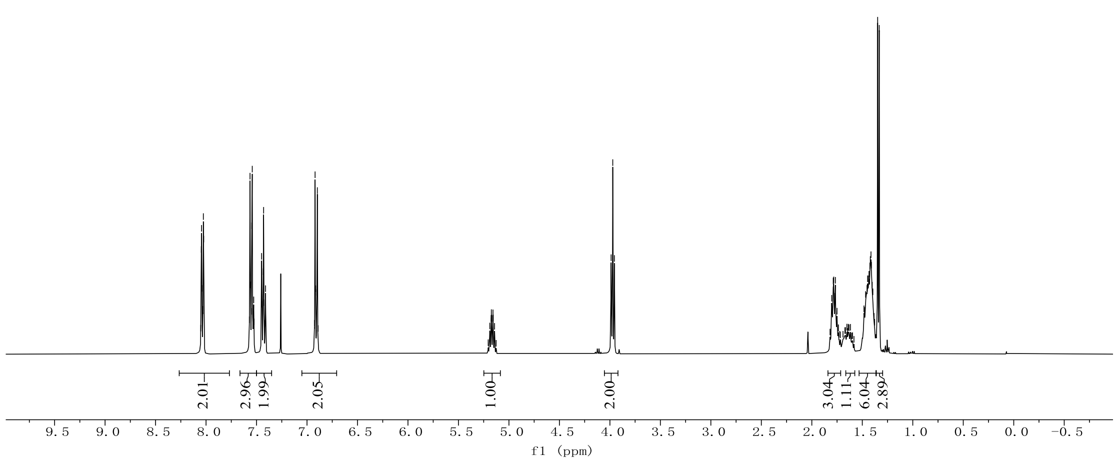

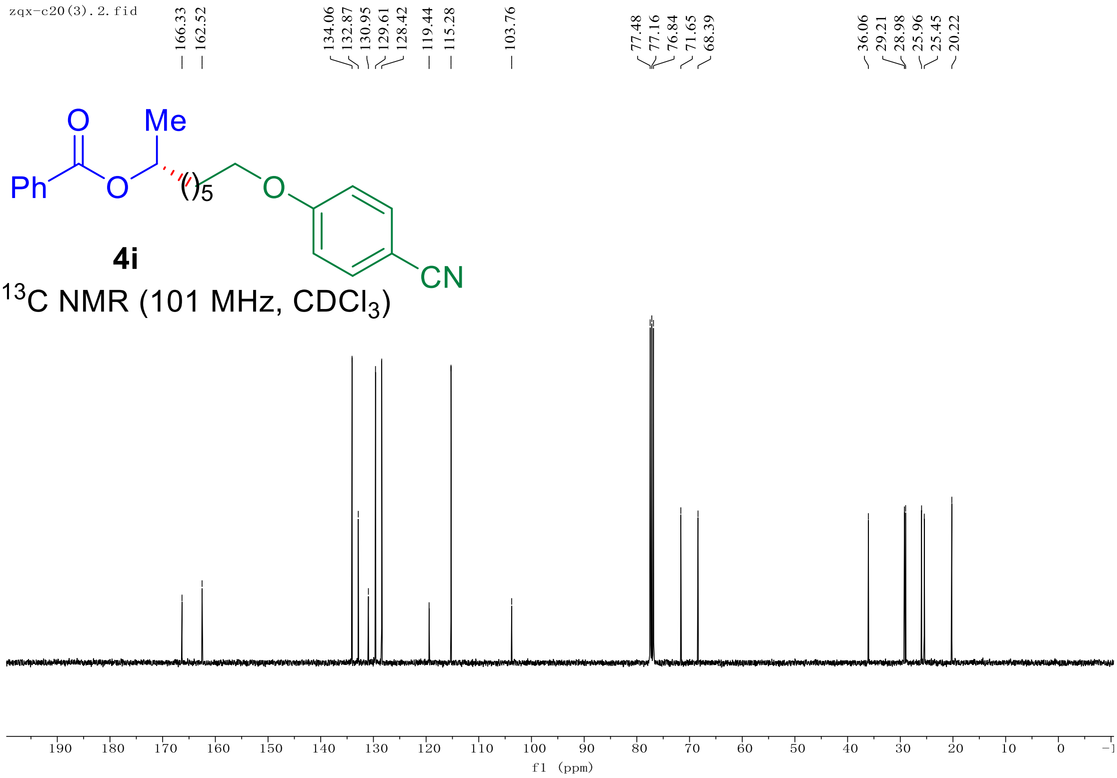
zqx-c20(3).2.fid
166.33
162.52
134.06
132.87
130.95
129.61
128.42
119.44
115.28
103.76
77.48
77.16
76.84
71.65
68.39
36.06
29.21
28.98
25.96
25.45
20.22
O
Me
Ph
O
( )5
O
CN
4i
^{13}C NMR (101 MHz, CDCl$_3$)
190 180 170 160 150 140 130 120 110 100 90 80 70 60 50 40 30 20 10 0 -1
f1 (ppm)

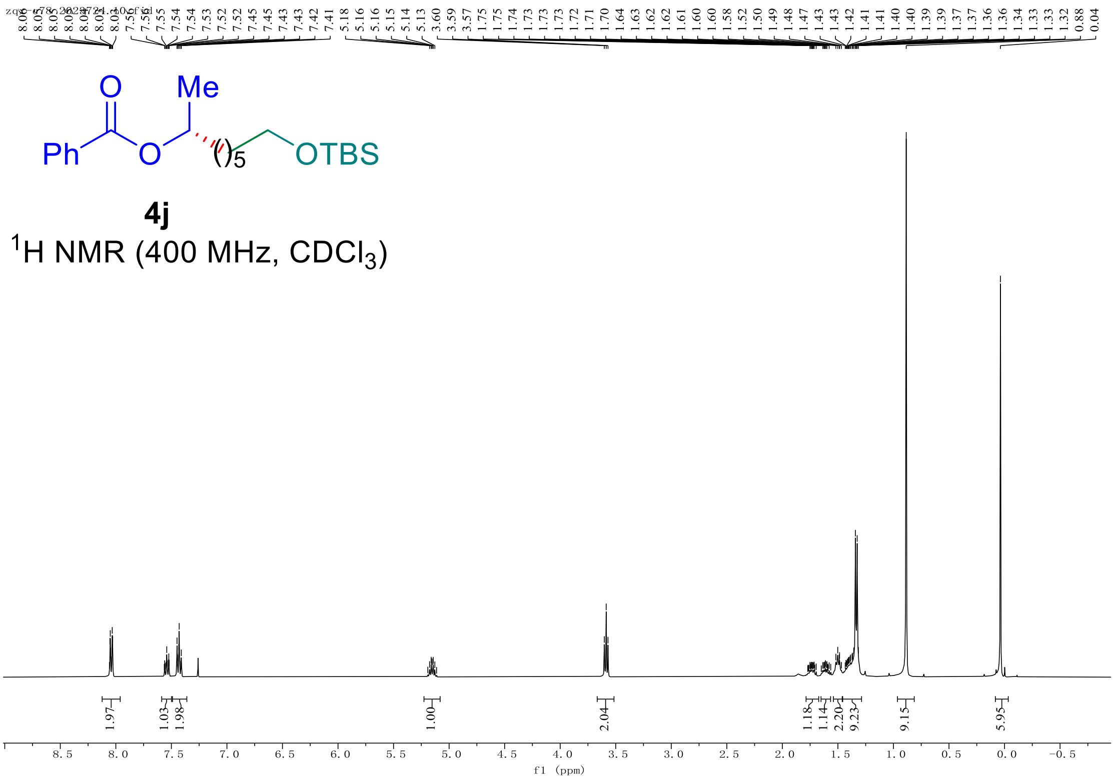
O
Me
Ph
O
( )5
OTBS
4j
^{1}H NMR (400 MHz, CDCl$_3$)
1.97
1.03
1.98
1.00
2.04
1.18
1.14
2.20
9.23
9.15
5.95
8.5 8.0 7.5 7.0 6.5 6.0 5.5 5.0 4.5 4.0 3.5 3.0 2.5 2.0 1.5 1.0 0.5 0.0 -0.5
f1 (ppm)

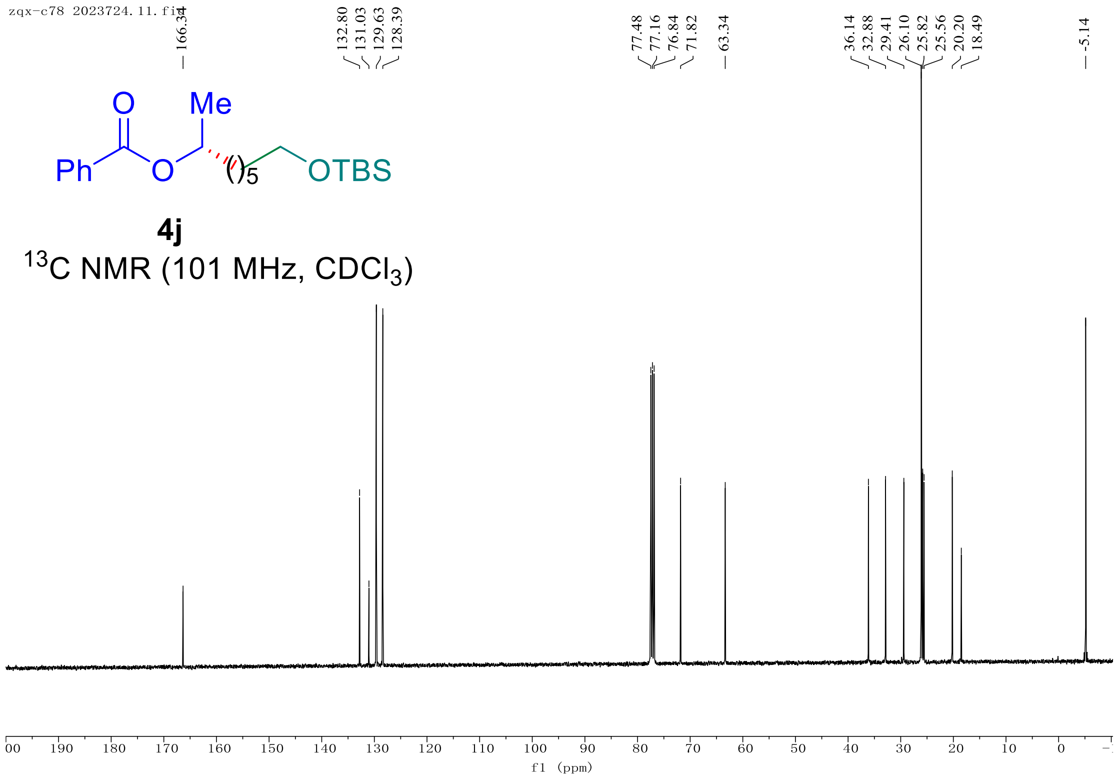
zqx-c78 2023724.11.fid
166.34
132.80
131.03
129.63
128.39
77.48
77.16
76.84
71.82
63.34
36.14
32.88
29.41
26.10
25.82
25.56
20.20
18.49
-5.14
O
Me
Ph
O
( )5
OTBS
4j
^{13}C NMR (101 MHz, $CDCl_3$)
f1 (ppm)

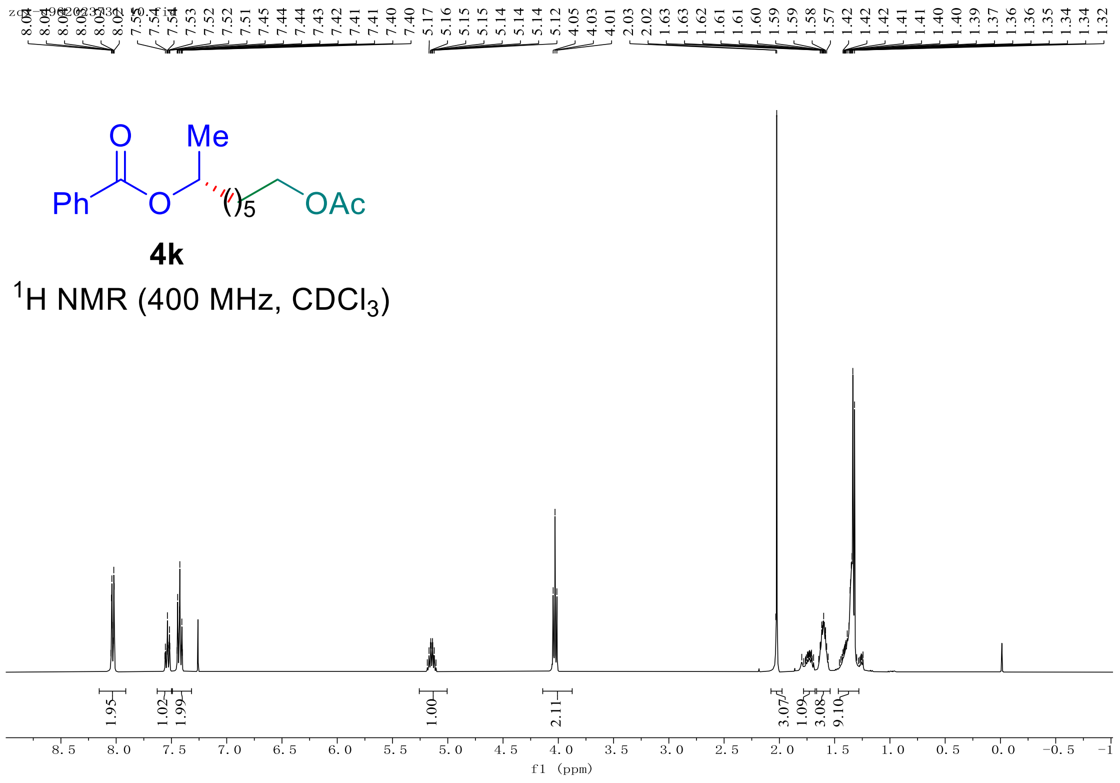
8.04
8.03
8.03
8.02
8.02
8.02
7.55
7.54
7.54
7.53
7.52
7.52
7.51
7.45
7.44
7.44
7.43
7.42
7.41
7.41
7.40
7.40
5.17
5.16
5.15
5.15
5.14
5.14
5.14
5.12
4.05
4.03
4.01
2.03
2.02
1.63
1.63
1.62
1.61
1.61
1.60
1.59
1.59
1.58
1.57
1.42
1.42
1.42
1.41
1.41
1.40
1.40
1.39
1.37
1.36
1.36
1.35
1.34
1.34
1.32
O
Me
Ph
O
( )5
OAc
4k
^{1}H NMR (400 MHz, $CDCl_3$)
1.95
1.02
1.99
1.00
2.11
3.07
1.09
3.08
9.10
f1 (ppm)

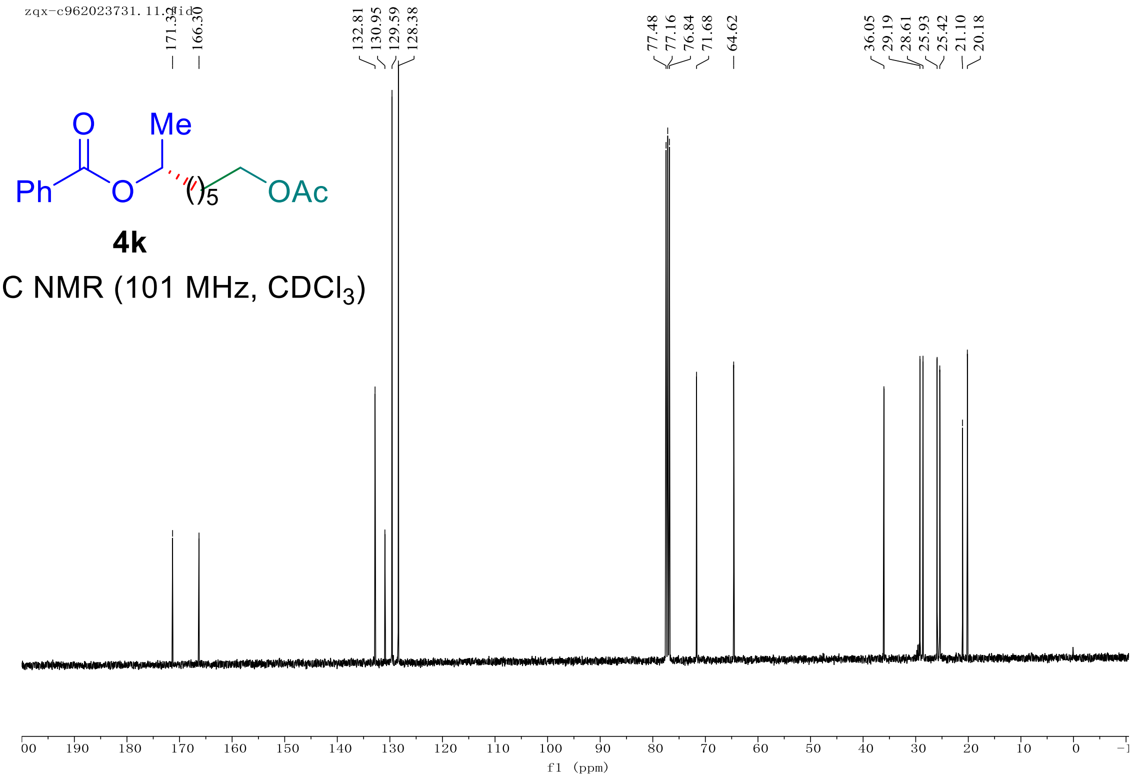
zqx-c962023731.11.2.fid
171.32
166.30
132.81
130.95
129.59
128.38
77.48
77.16
76.84
71.68
64.62
36.05
29.19
28.61
25.93
25.42
21.10
20.18
O
Me
Ph
O
()5
OAc
4k
13C NMR (101 MHz, CDCl3)
f1 (ppm)

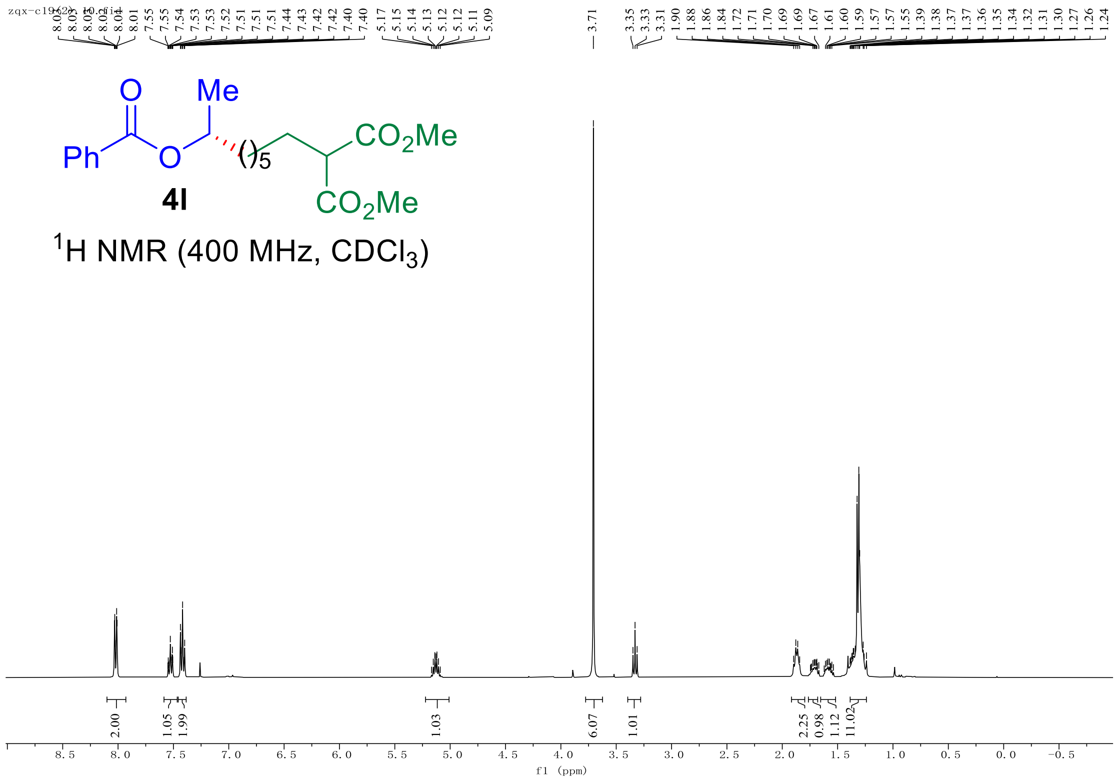
8.03
8.03
8.02
8.02
8.01
8.01
7.55
7.55
7.54
7.53
7.53
7.52
7.51
7.51
7.51
7.44
7.43
7.42
7.42
7.40
5.17
5.15
5.14
5.13
5.12
5.12
5.11
5.09
3.71
3.35
3.33
3.31
1.90
1.88
1.86
1.84
1.72
1.71
1.70
1.69
1.69
1.67
1.61
1.60
1.59
1.57
1.57
1.55
1.39
1.38
1.37
1.37
1.36
1.35
1.34
1.32
1.31
1.30
1.27
1.26
1.24
O
Me
Ph
O
()5
CO2Me
CO2Me
4l
1H NMR (400 MHz, CDCl3)
2.00
1.05
1.99
1.03
6.07
1.01
2.25
0.98
1.12
11.02
f1 (ppm)

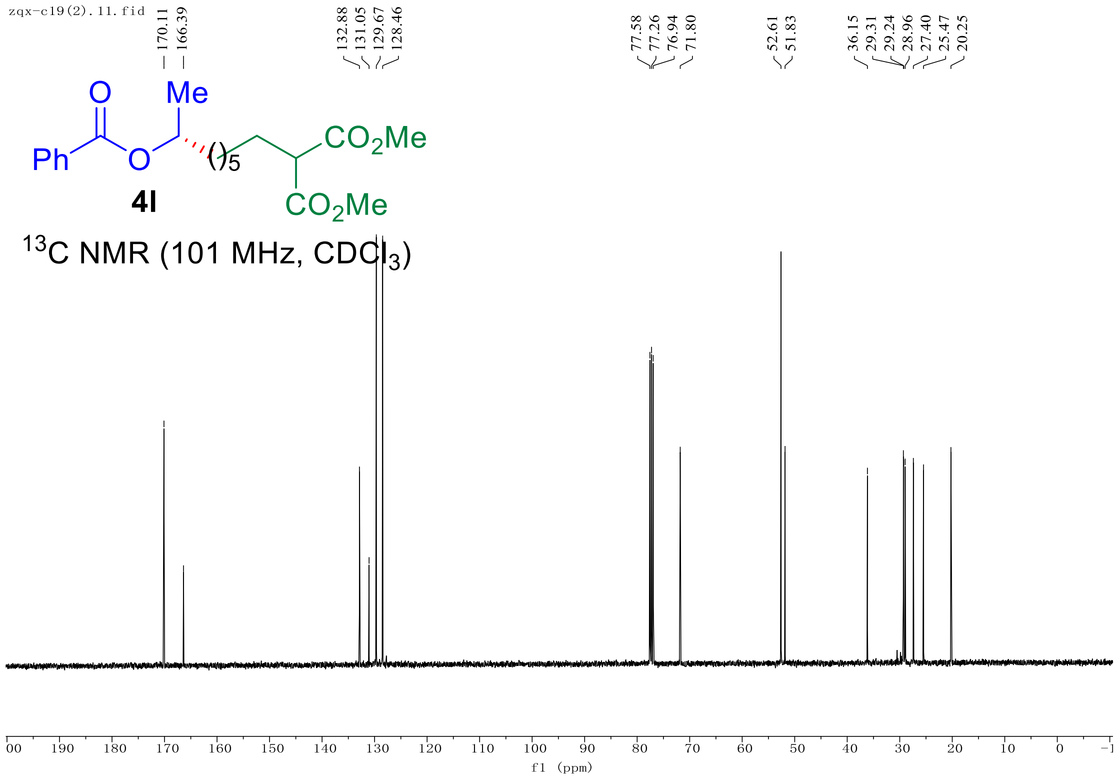

zqx-c19(2).11.fid
4l
13C NMR (101 MHz, CDCl3)
f1 (ppm)

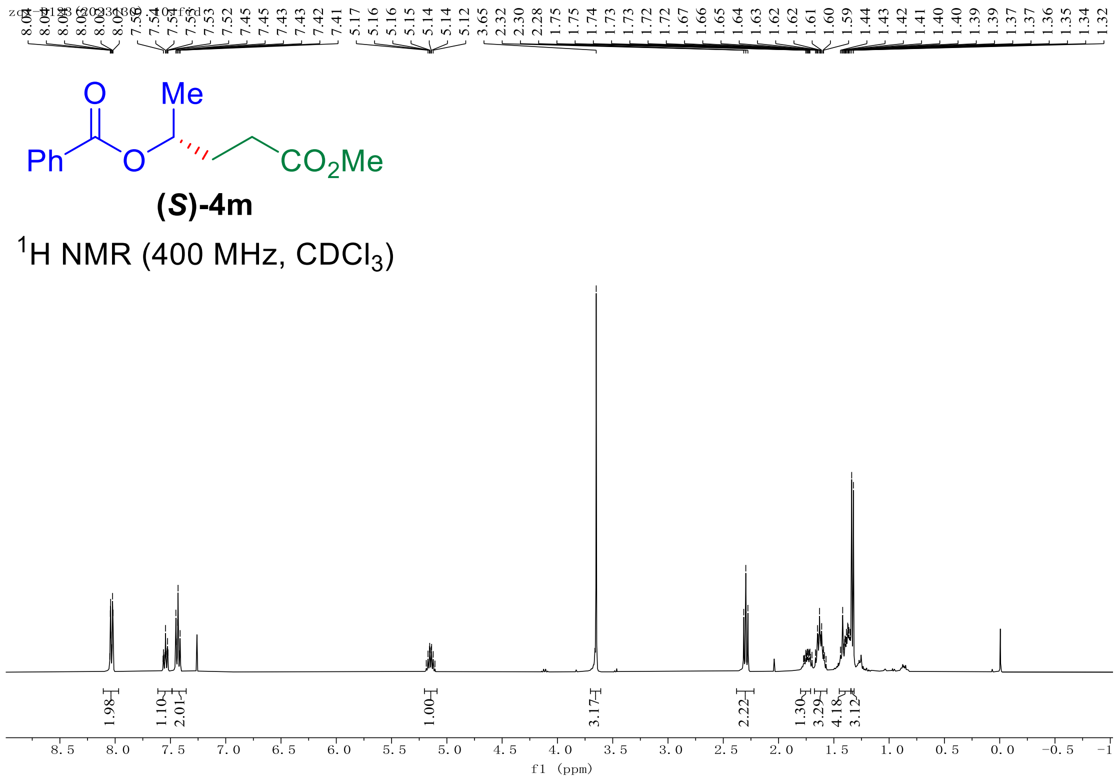

(S)-4m
1H NMR (400 MHz, CDCl3)
f1 (ppm)

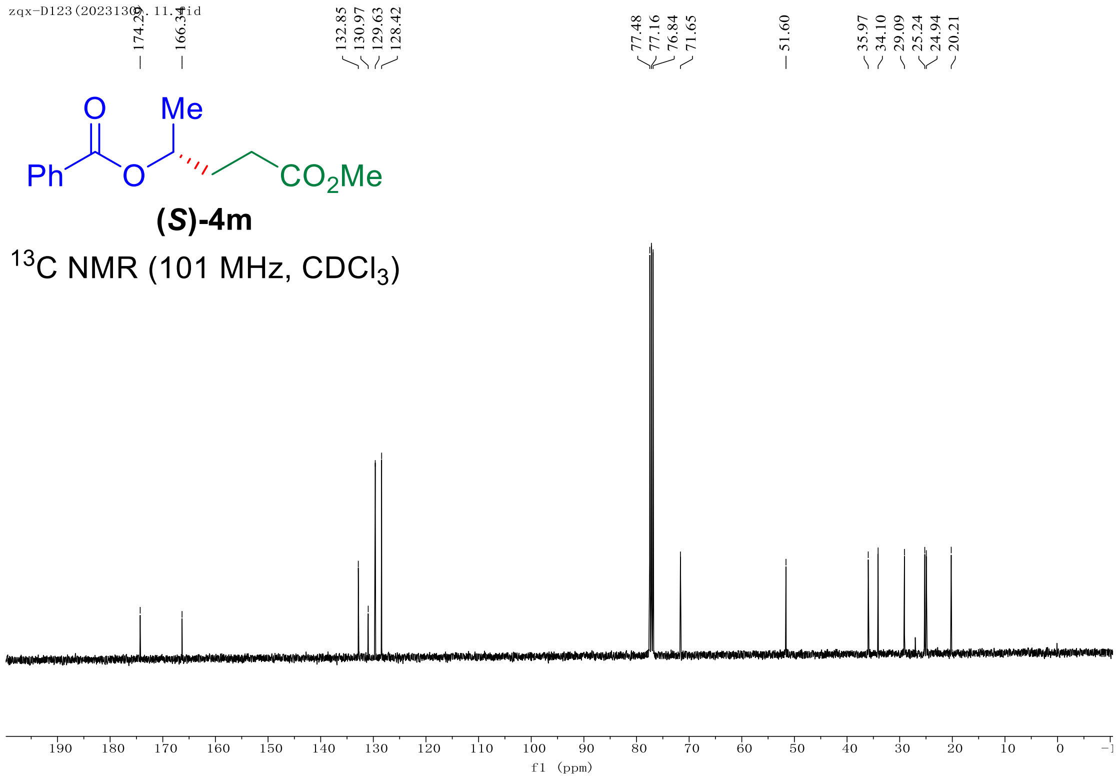

zqx-D123(20231109.11.fid
174.29
166.34
132.85
130.97
129.63
128.42
77.48
77.16
76.84
71.65
51.60
35.97
34.10
29.09
25.24
24.94
20.21
Ph
O
O
Me
CO2Me
(S)-4m
13C NMR (101 MHz, CDCl3)
f1 (ppm)

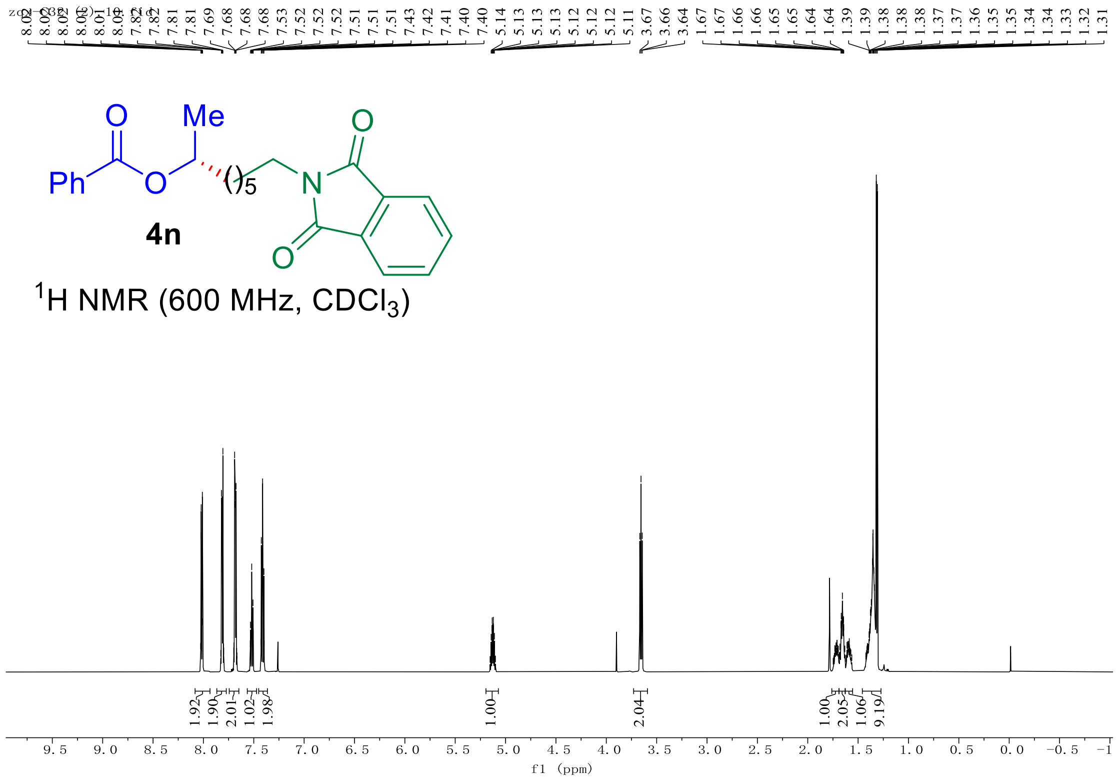

8.02
8.02
8.02
8.01
8.01
8.01
7.82
7.82
7.81
7.81
7.69
7.68
7.68
7.68
7.53
7.52
7.52
7.52
7.51
7.51
7.51
7.43
7.42
7.41
7.40
7.40
5.14
5.13
5.13
5.13
5.12
5.12
5.12
5.11
3.67
3.66
3.64
1.67
1.67
1.66
1.66
1.65
1.65
1.64
1.64
1.39
1.39
1.38
1.38
1.38
1.37
1.37
1.36
1.35
1.35
1.34
1.34
1.33
1.32
1.31
Ph
O
O
Me
N
O
O
4n
1H NMR (600 MHz, CDCl3)
1.92
1.90
2.01
1.02
1.98
1.00
2.04
1.00
2.05
1.06
9.19
f1 (ppm)

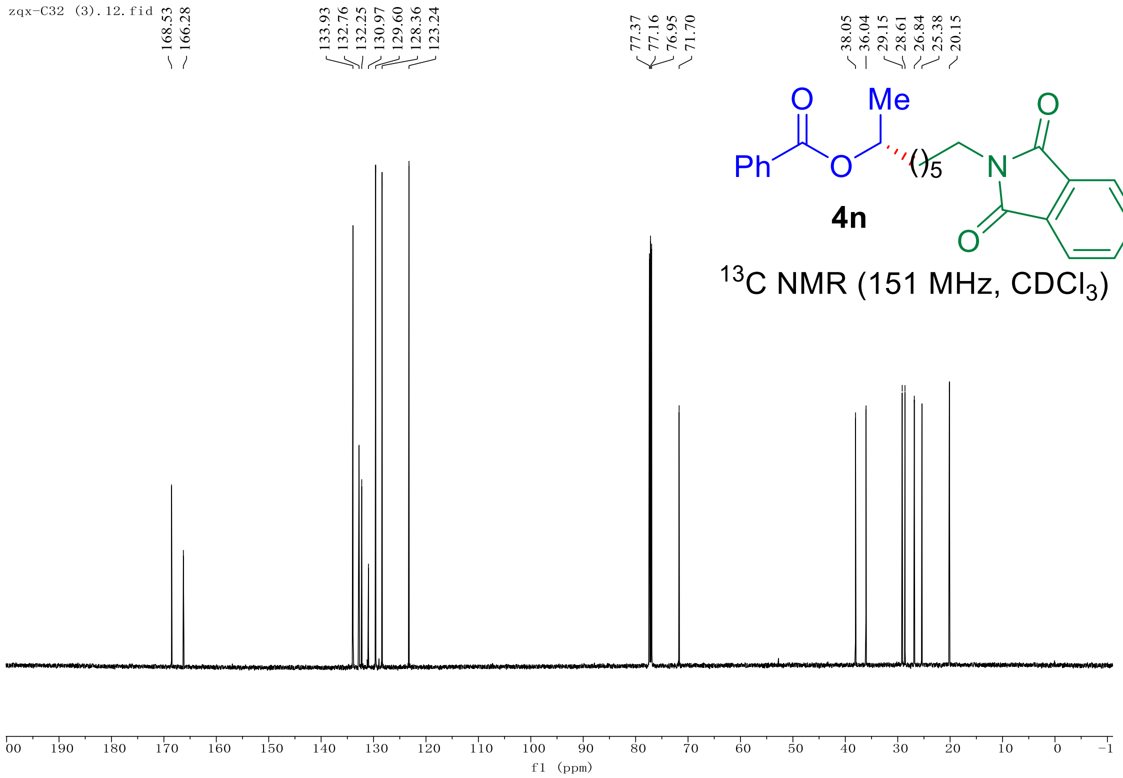
zqx-C32 (3).12.fid
168.53
166.28
133.93
132.76
132.25
130.97
129.60
128.36
123.24
77.37
77.16
76.95
71.70
38.05
36.04
29.15
28.61
26.84
25.38
20.15
O
Me
O
Ph
O
( )5
N
O
4n
13C NMR (151 MHz, CDCl3)
f1 (ppm)

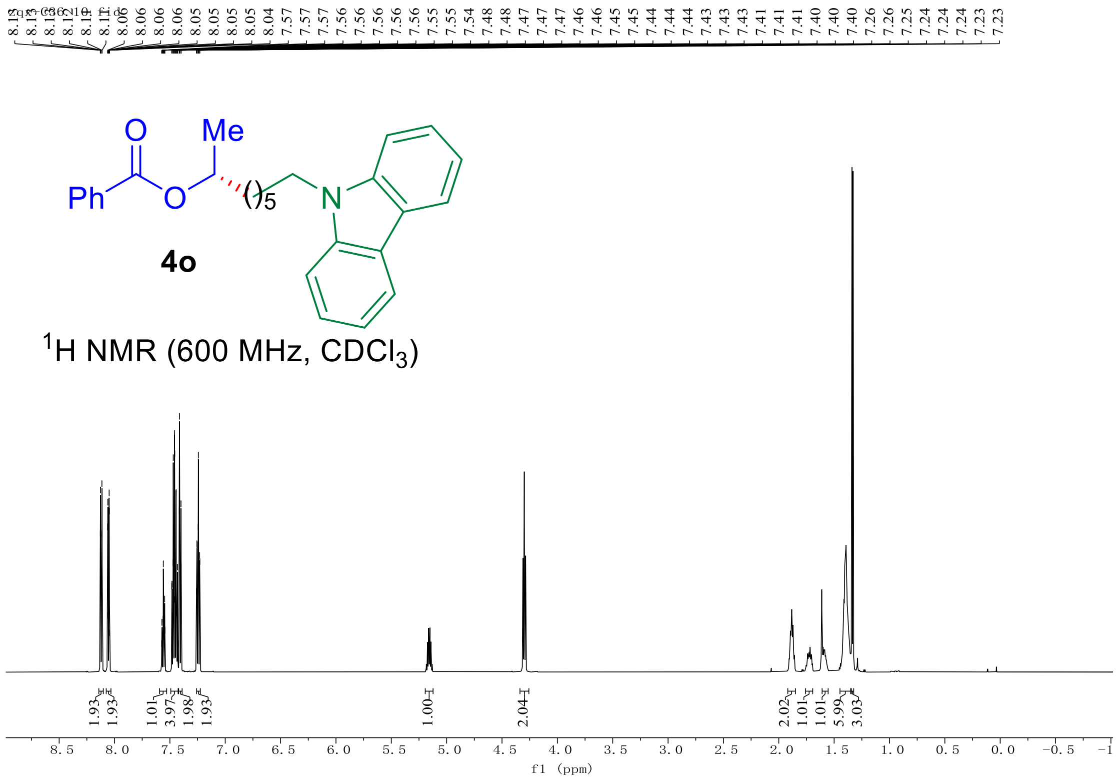
O
Me
Ph
O
( )5
N
4o
1H NMR (600 MHz, CDCl3)
1.93
1.93
1.01
3.97
1.98
1.93
1.00
2.04
2.02
1.01
1.01
5.99
3.03
f1 (ppm)

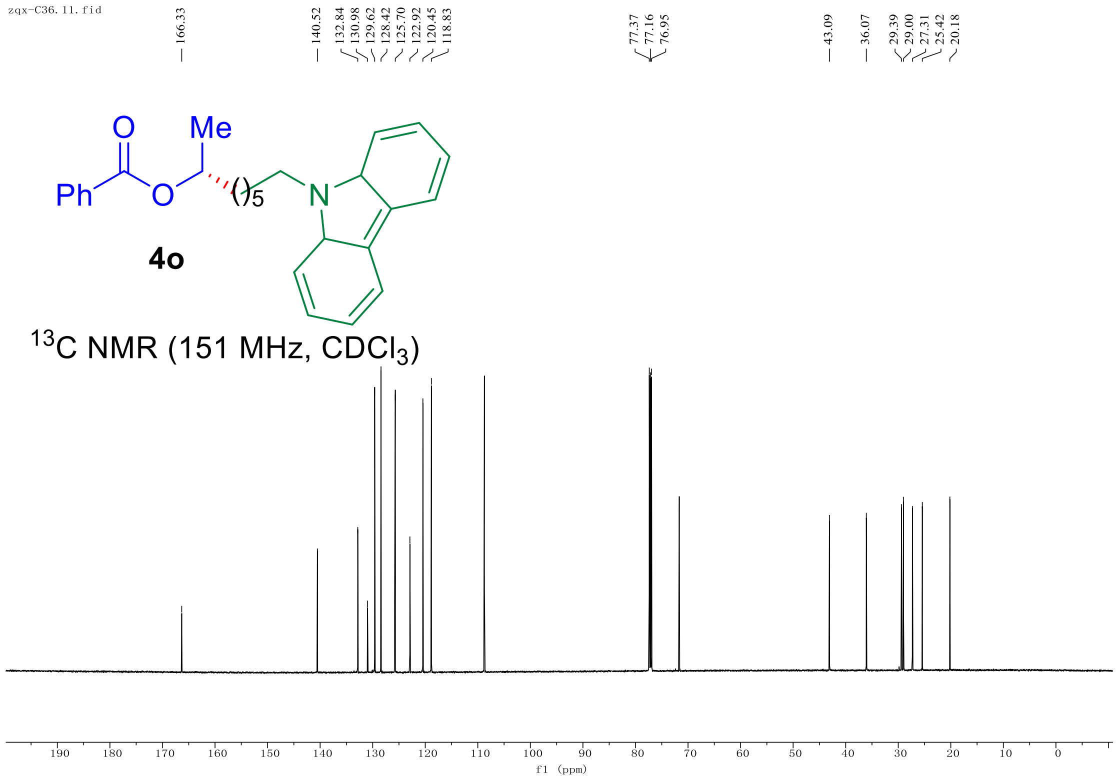
zqx-C36.11.fid
166.33
140.52
132.84
130.98
129.62
128.42
125.70
122.92
120.45
118.83
77.37
77.16
76.95
43.09
36.07
29.39
29.00
27.31
25.42
20.18
O
Me
Ph
O
()5
N
4o
^{13}C NMR (151 MHz, $CDCl_3$)
190 180 170 160 150 140 130 120 110 100 90 80 70 60 50 40 30 20 10 0
f1 (ppm)

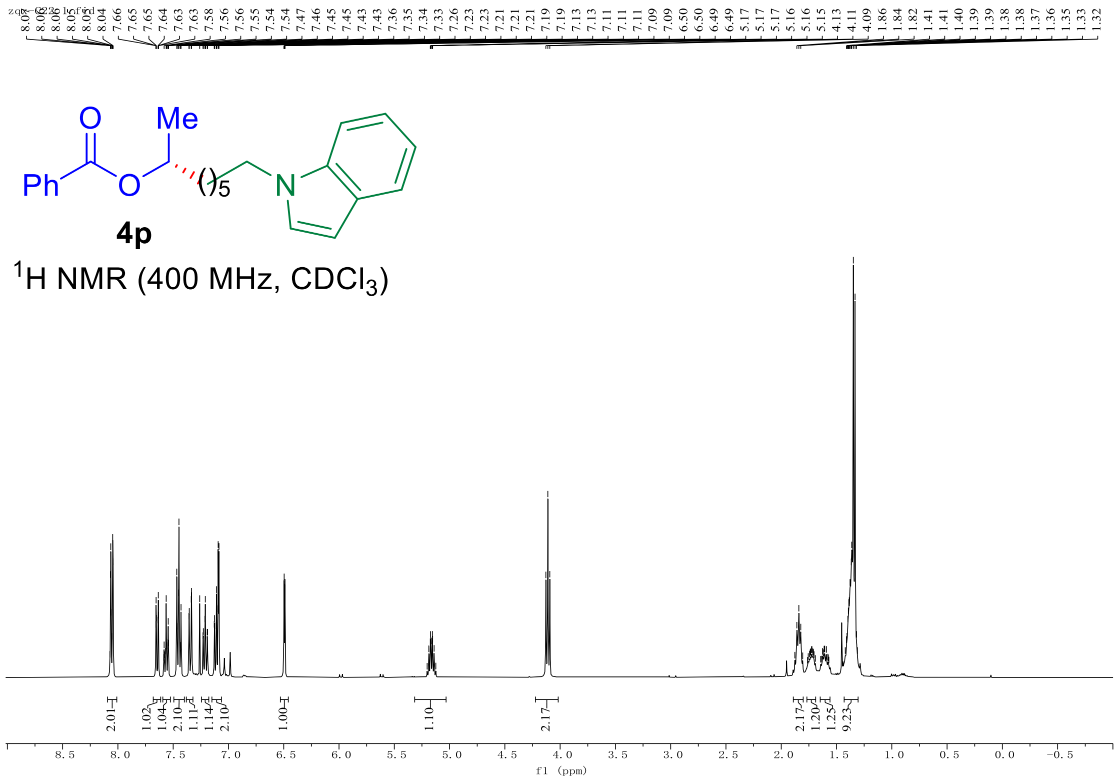
8.06
8.05
8.05
8.04
7.66
7.65
7.65
7.64
7.63
7.63
7.58
7.56
7.55
7.54
7.54
7.47
7.46
7.45
7.45
7.43
7.43
7.36
7.35
7.34
7.33
7.26
7.23
7.23
7.21
7.21
7.21
7.19
7.19
7.13
7.13
7.11
7.11
7.11
7.09
7.09
6.50
6.49
6.49
5.17
5.17
5.16
5.16
5.15
4.13
4.11
4.09
1.86
1.84
1.82
1.41
1.41
1.40
1.39
1.39
1.38
1.38
1.37
1.36
1.35
1.33
1.32
O
Me
Ph
O
()5
N
4p
^{1}H NMR (400 MHz, $CDCl_3$)
2.01
1.02
1.04
2.10
1.11
1.14
2.10
1.00
1.10
2.17
2.17
1.20
1.25
9.23
8.5 8.0 7.5 7.0 6.5 6.0 5.5 5.0 4.5 4.0 3.5 3.0 2.5 2.0 1.5 1.0 0.5 0.0 -0.5
f1 (ppm)

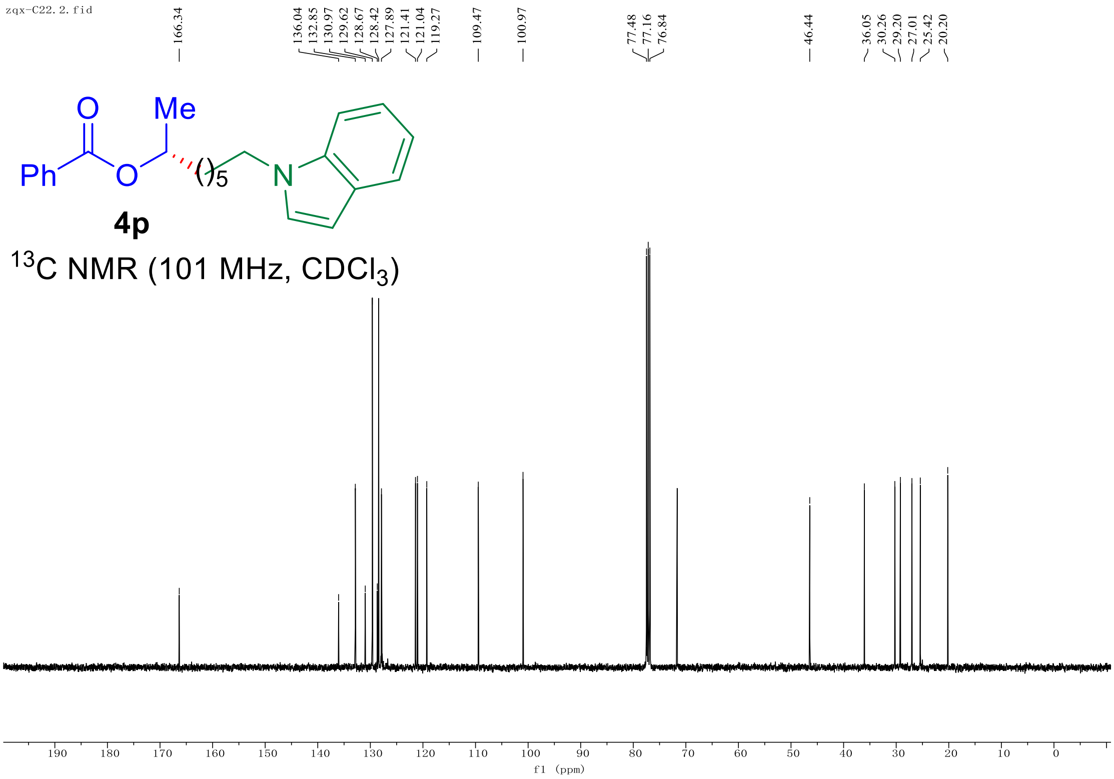

4p

^{13}C NMR (101 MHz, $CDCl_3$)

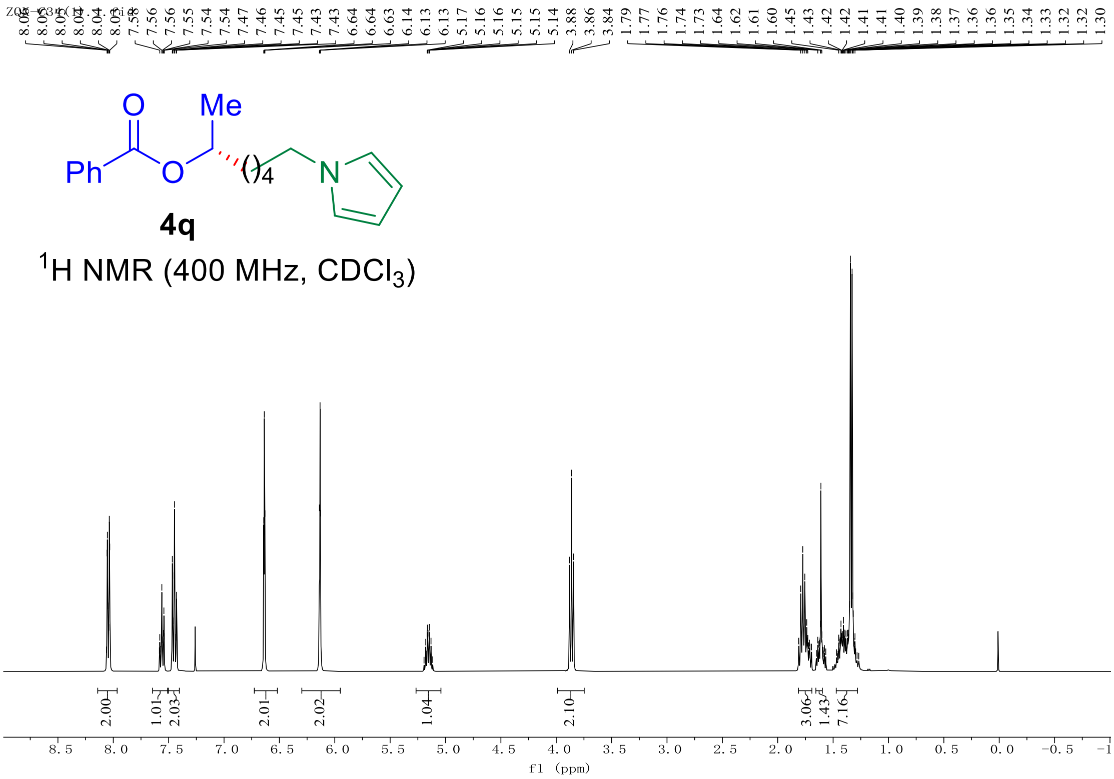

4q

^{1}H NMR (400 MHz, $CDCl_3$)

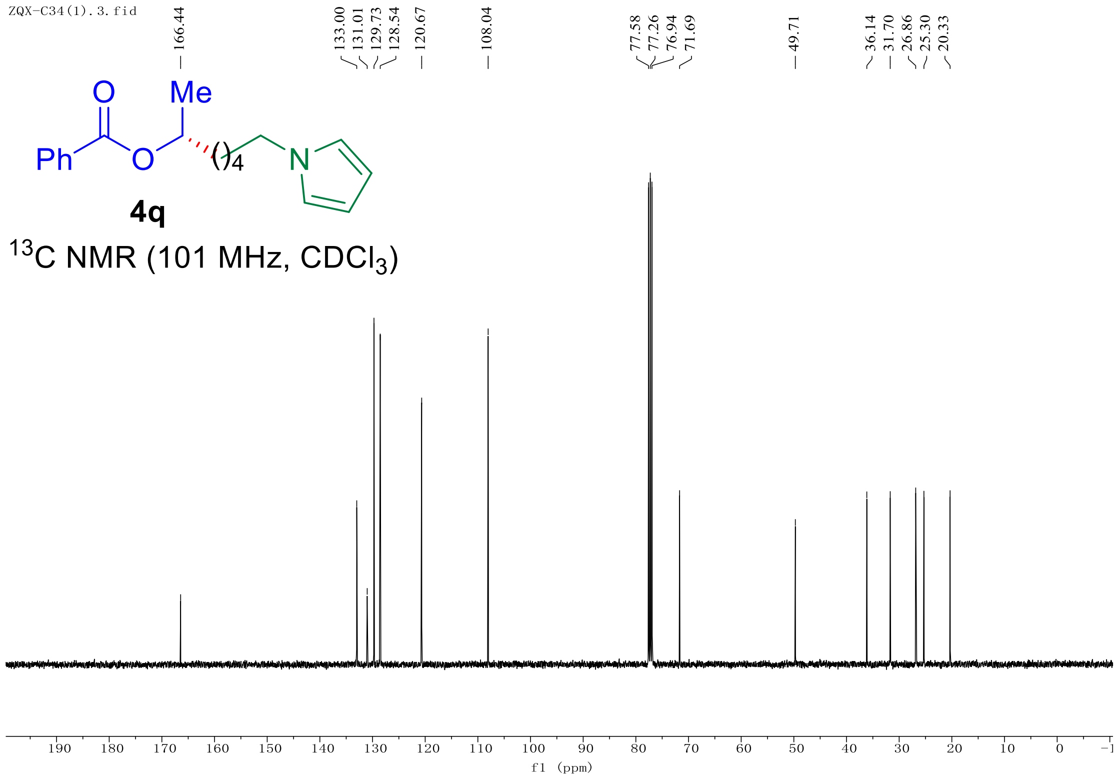
ZQX-C34(1).3.fid
166.44
133.00
131.01
129.73
128.54
120.67
108.04
77.58
77.26
76.94
71.69
49.71
36.14
31.70
26.86
25.30
20.33
O
Me
Ph
O
( )4
N
4q
^{13}C NMR (101 MHz, CDCl$_3$)
190 180 170 160 150 140 130 120 110 100 90 80 70 60 50 40 30 20 10 0
f1 (ppm)

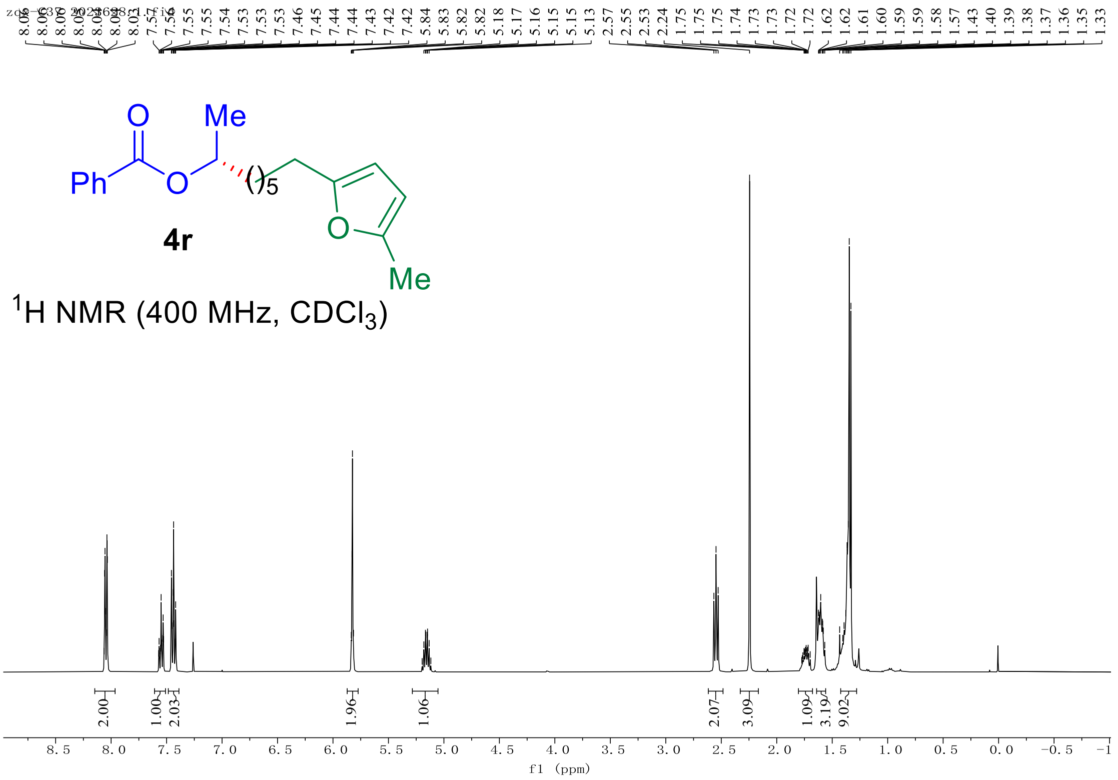
8.02
7.57
7.56
7.55
7.55
7.54
7.53
7.53
7.53
7.46
7.45
7.44
7.44
7.43
7.42
7.42
5.84
5.83
5.82
5.82
5.18
5.17
5.16
5.15
5.15
5.13
2.57
2.55
2.53
2.24
1.75
1.75
1.74
1.73
1.73
1.72
1.72
1.62
1.62
1.61
1.60
1.59
1.59
1.58
1.57
1.43
1.40
1.39
1.38
1.37
1.36
1.35
1.33
O
Me
Ph
O
( )5
O
Me
4r
^{1}H NMR (400 MHz, CDCl$_3$)
2.00
1.00
2.03
1.96
1.06
2.07
3.09
1.09
3.19
9.02
8.5 8.0 7.5 7.0 6.5 6.0 5.5 5.0 4.5 4.0 3.5 3.0 2.5 2.0 1.5 1.0 0.5 0.0 -0.5
f1 (ppm)

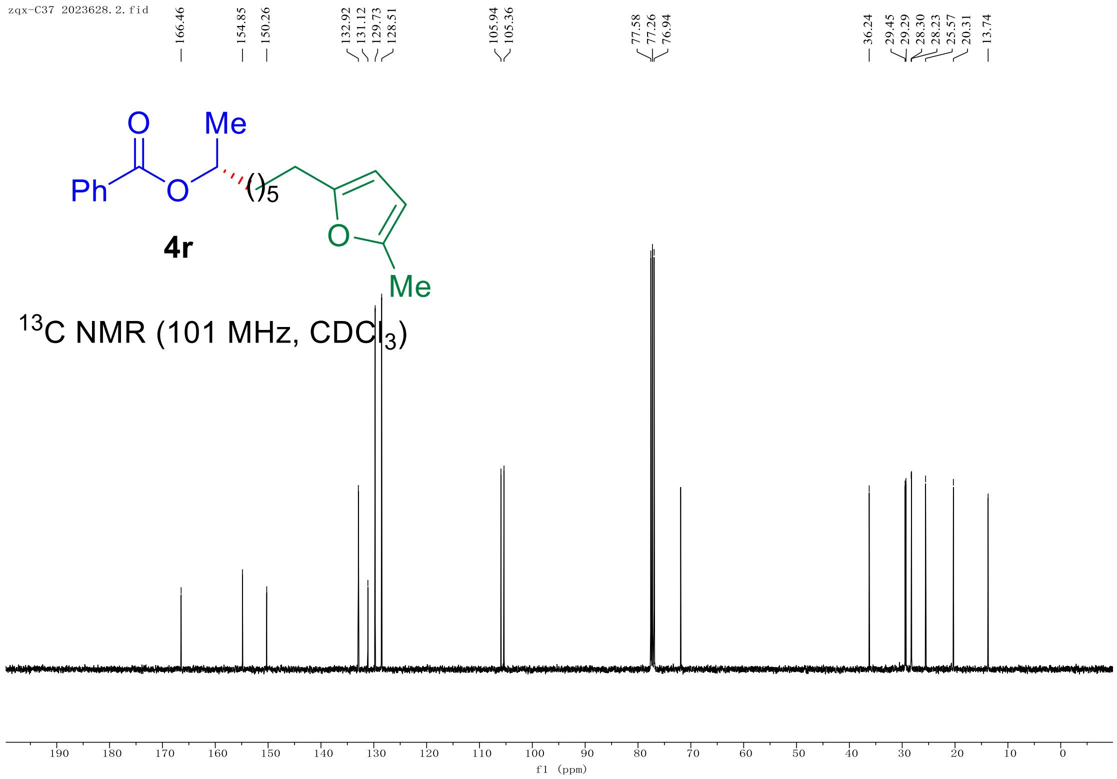
zqx-C37 2023628.2.fid
166.46
154.85
150.26
132.92
131.12
129.73
128.51
105.94
105.36
77.58
77.26
76.94
36.24
29.45
29.29
28.30
28.23
25.57
20.31
13.74
Ph
O
O
Me
)5
O
Me
4r
13C NMR (101 MHz, CDCl3)
f1 (ppm)

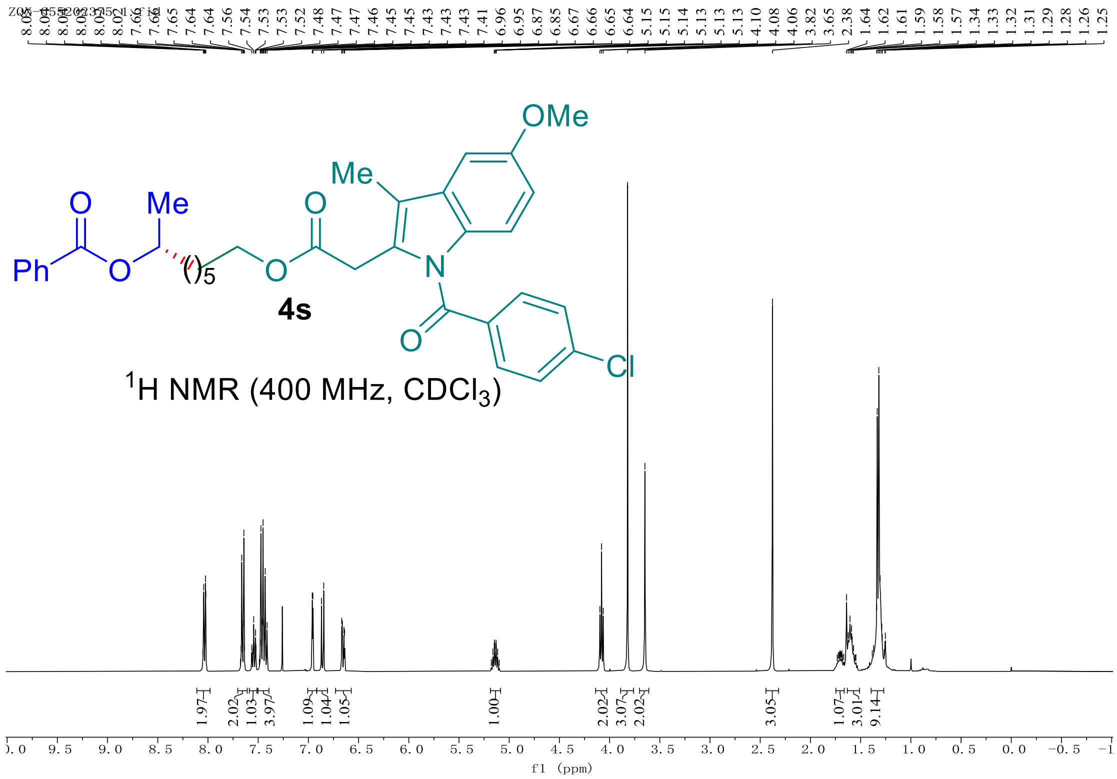
8.05
8.05
8.04
8.03
8.03
8.02
7.66
7.66
7.65
7.64
7.64
7.56
7.54
7.53
7.53
7.52
7.48
7.47
7.47
7.46
7.45
7.45
7.43
7.43
7.43
7.41
6.96
6.95
6.87
6.85
6.67
6.66
6.65
6.64
5.15
5.15
5.14
5.13
5.13
5.13
4.10
4.08
4.06
3.82
3.65
2.38
1.64
1.62
1.61
1.59
1.58
1.57
1.34
1.33
1.32
1.31
1.29
1.28
1.26
1.25
OMe
Me
O
Me
Ph
O
)5
O
O
N
4s
O
Cl
1H NMR (400 MHz, CDCl3)
1.97
2.02
1.03
3.97
1.09
1.04
1.05
1.00
2.02
3.07
2.02
3.05
1.07
3.01
9.14
f1 (ppm)

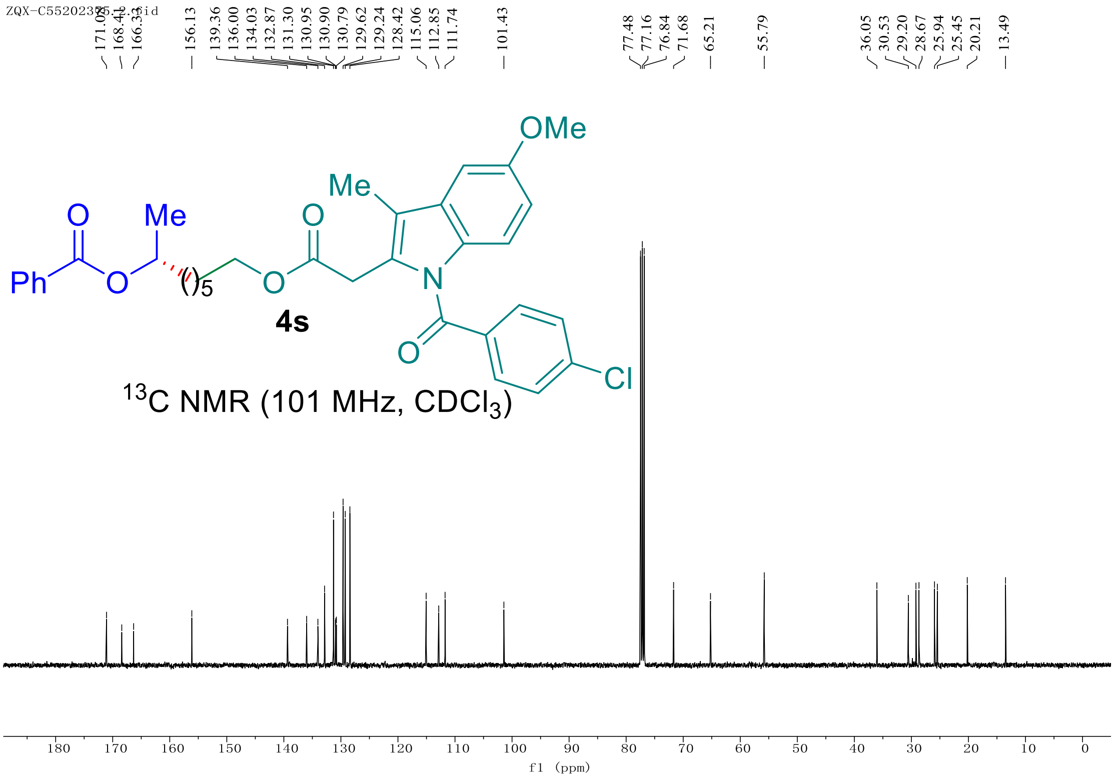

171.05
168.41
166.33
156.13
139.36
136.00
134.03
132.87
131.30
130.95
130.90
130.79
129.62
129.24
128.42
115.06
112.85
111.74
101.43
77.48
77.16
76.84
71.68
65.21
55.79
36.05
30.53
29.20
28.67
25.94
25.45
20.21
13.49
OMe
Me
O
Me
Ph
O
()5
O
O
N
4s
O
Cl
13C NMR (101 MHz, CDCl3)
f1 (ppm)

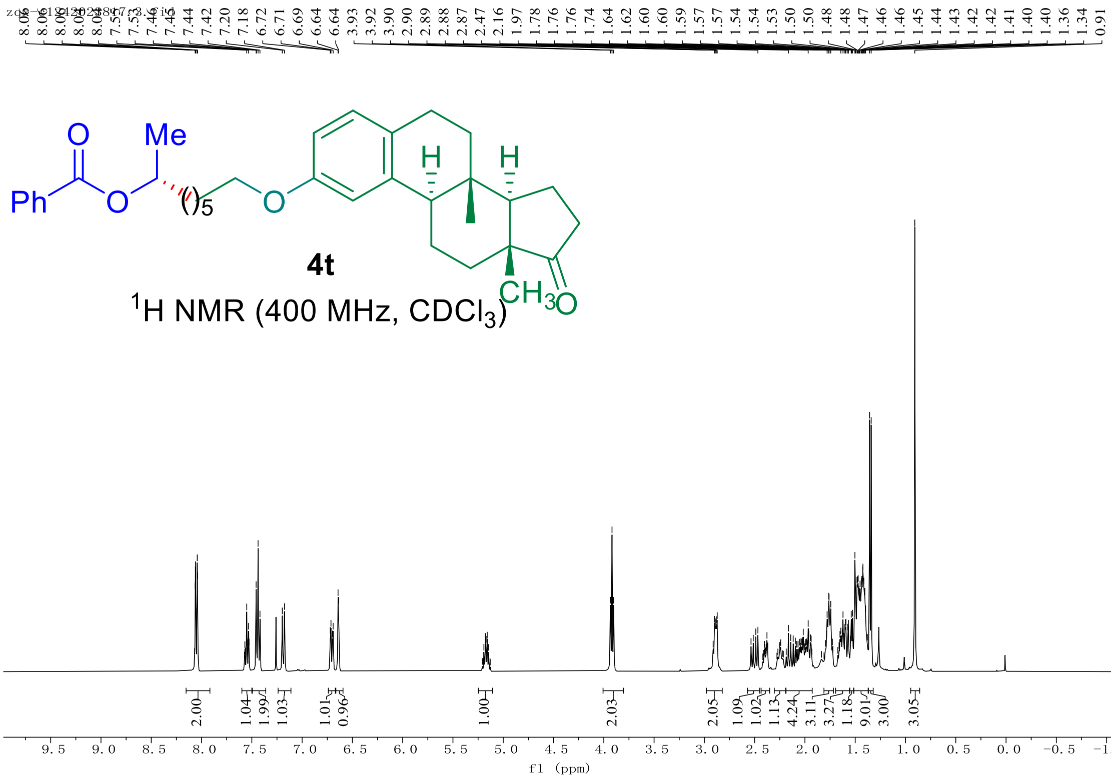

O
Me
Ph
O
()5
O
H
H
4t
1H NMR (400 MHz, CDCl3)
CH3
O
f1 (ppm)

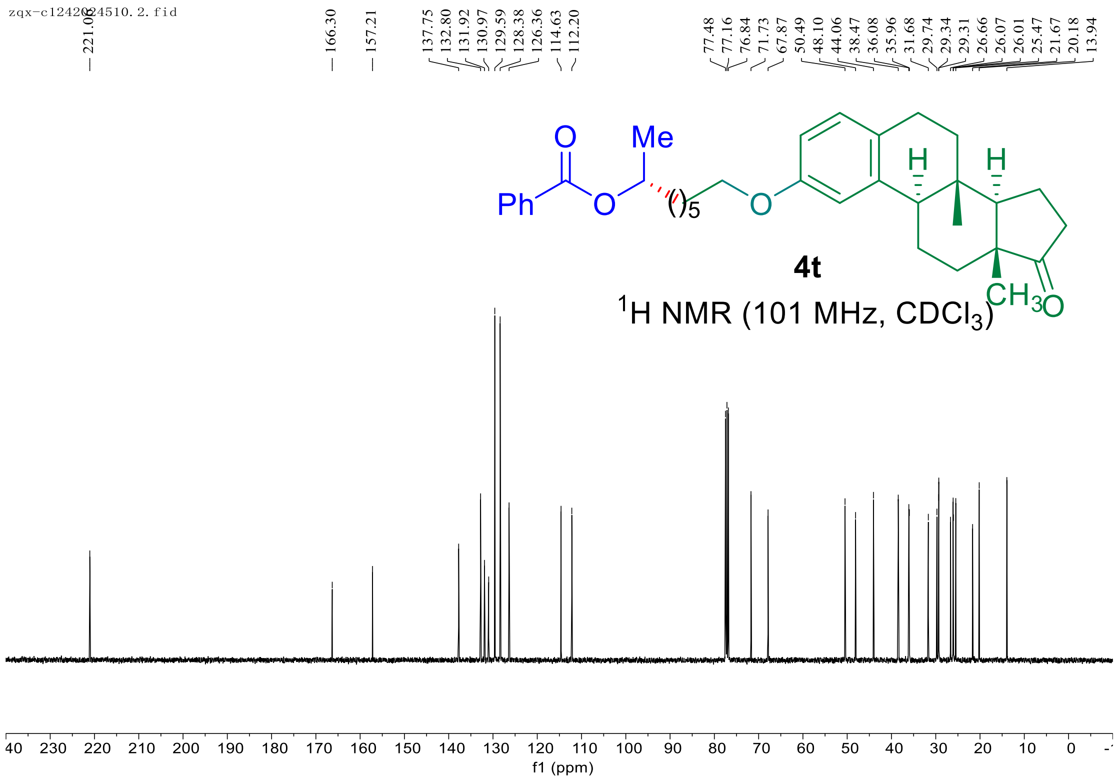
zqx-c1242024510.2.fid
221.06
166.30
157.21
137.75
132.80
131.92
130.97
129.59
128.38
126.36
114.63
112.20
77.48
77.16
76.84
71.73
67.87
50.49
48.10
44.06
38.47
36.08
35.96
31.68
29.74
29.34
29.31
26.66
26.07
26.01
25.47
21.67
20.18
13.94
O
Me
Ph
O
( )5
O
H
H
CH3
O
4t
1H NMR (101 MHz, CDCl3)
f1 (ppm)

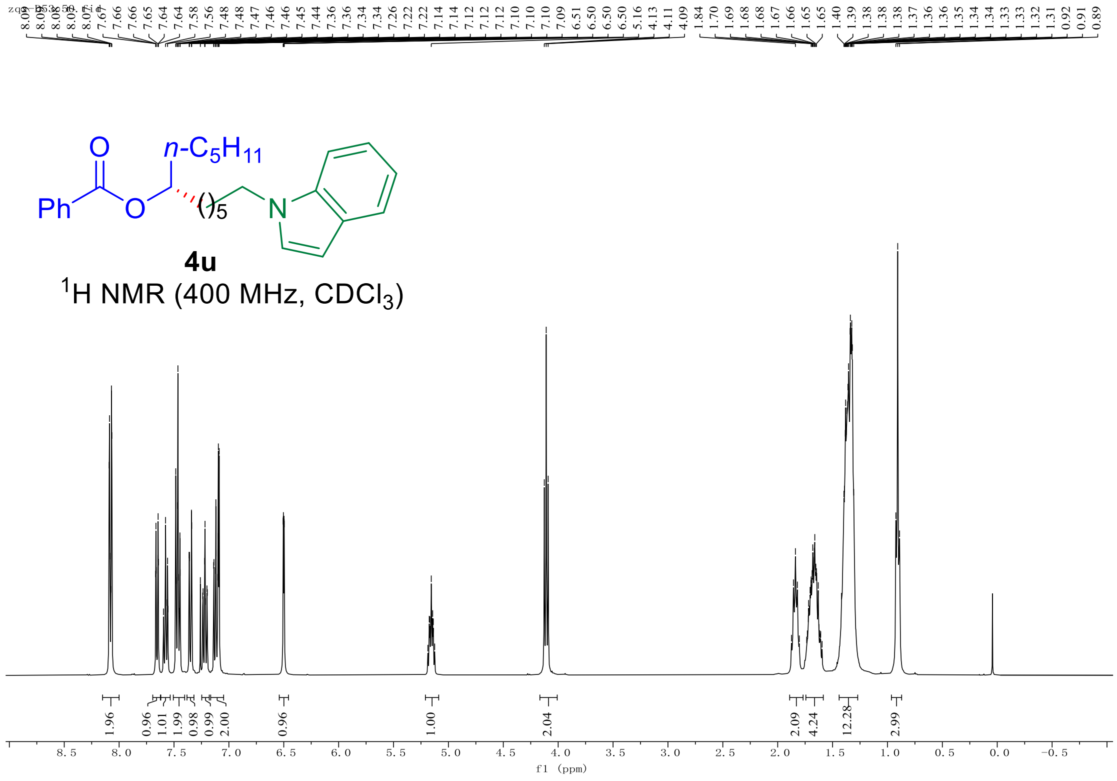
8.09 8.09 8.08 8.07 8.07 7.67 7.66 7.65 7.64 7.64 7.58 7.56 7.48 7.48 7.47 7.46 7.46 7.45 7.44 7.36 7.36 7.34 7.34 7.26 7.22 7.22 7.14 7.14 7.12 7.12 7.10 7.10 7.09 6.51 6.50 6.50 5.16 4.13 4.11 4.09 1.84 1.70 1.69 1.68 1.67 1.66 1.65 1.65 1.40 1.39 1.38 1.38 1.37 1.36 1.36 1.35 1.34 1.34 1.33 1.33 1.32 1.31 0.92 0.91 0.89
O
n-C5H11
Ph
O
( )5
N
4u
1H NMR (400 MHz, CDCl3)
1.96
0.96
1.01
1.99
0.98
0.99
2.00
0.96
1.00
2.04
2.09
4.24
12.28
2.99
f1 (ppm)

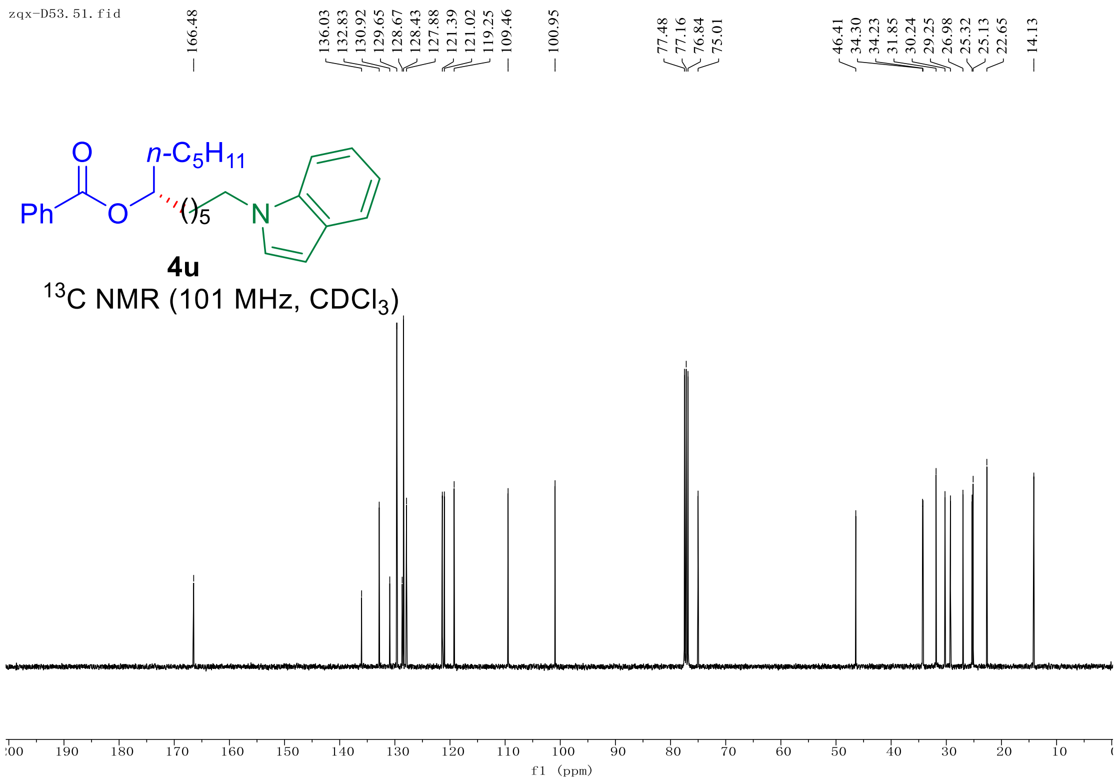
zqx-D53.51.fid
166.48
136.03
132.83
130.92
129.65
128.67
128.43
127.88
121.39
121.02
119.25
109.46
100.95
77.48
77.16
76.84
75.01
46.41
34.30
34.23
31.85
30.24
29.25
26.98
25.32
25.13
22.65
14.13
O
Ph
O
n-C5H11
)5
N
4u
13C NMR (101 MHz, CDCl3)
f1 (ppm)

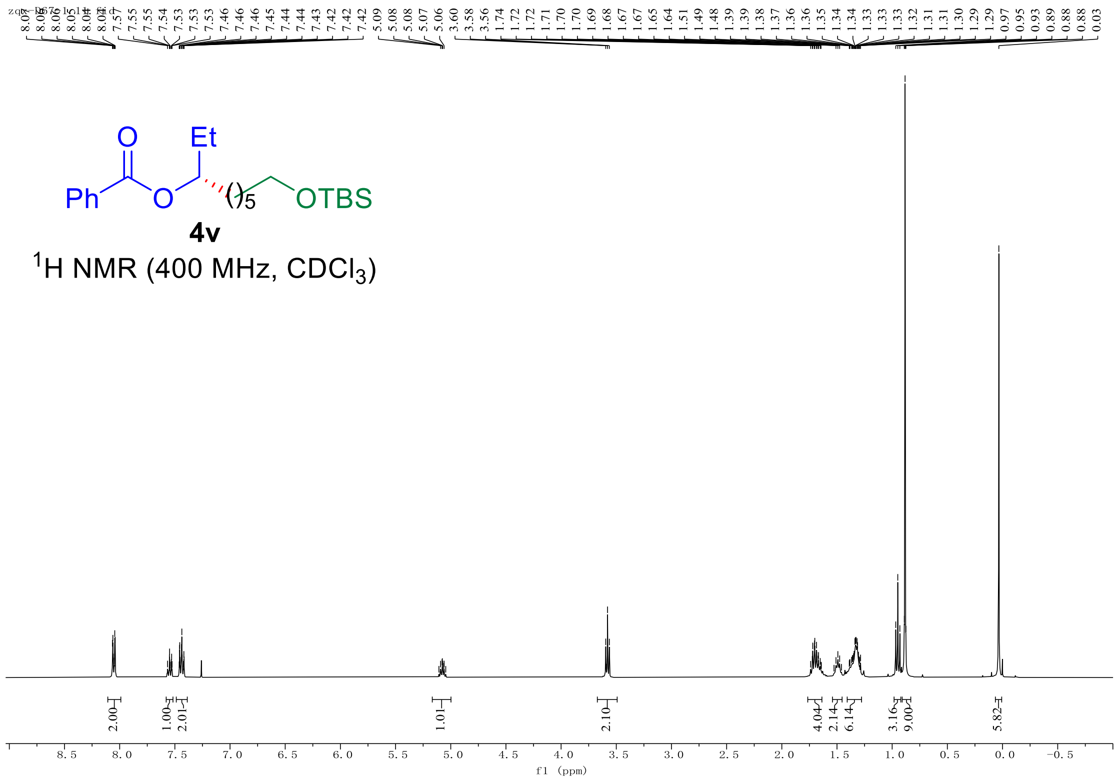
8.07 8.06 8.05 8.04 7.57 7.55 7.55 7.54 7.53 7.53 7.46 7.46 7.45 7.44 7.44 7.43 7.42 7.42 5.09 5.08 5.07 5.06 3.60 3.58 3.56 1.74 1.72 1.72 1.71 1.70 1.70 1.69 1.68 1.67 1.65 1.64 1.51 1.49 1.48 1.39 1.39 1.38 1.37 1.36 1.36 1.35 1.34 1.34 1.33 1.33 1.33 1.32 1.31 1.31 1.30 1.29 1.29 0.97 0.95 0.93 0.89 0.88 0.88 0.03
O
Et
Ph
O
)5
OTBS
4v
1H NMR (400 MHz, CDCl3)
2.00
1.00
2.01
1.01
2.10
4.04
2.14
6.14
3.16
9.00
5.82
f1 (ppm)

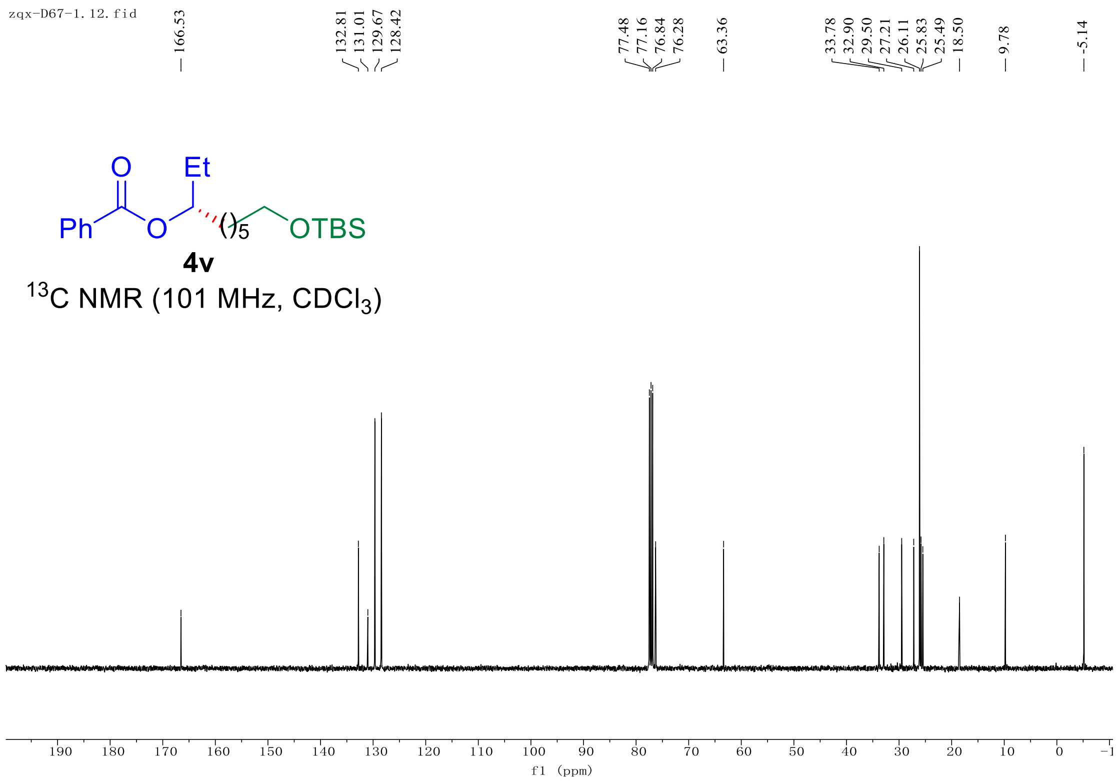

zqx-D67-1.12.fid
166.53
132.81
131.01
129.67
128.42
77.48
77.16
76.84
76.28
63.36
33.78
32.90
29.50
27.21
26.11
25.83
25.49
18.50
9.78
-5.14
O
Et
Ph
O
( )5
OTBS
4v
^{13}C NMR (101 MHz, CDCl$_3$)
f1 (ppm)

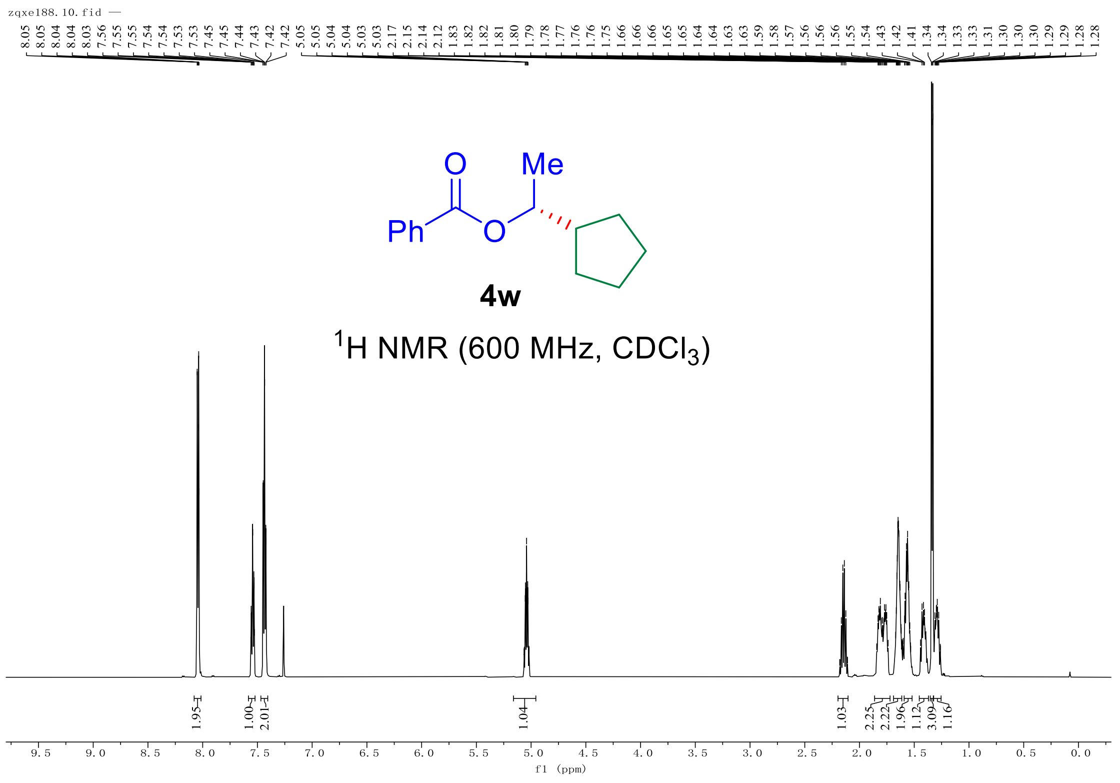

zqxe188.10.fid
O
Me
Ph
O
4w
^{1}H NMR (600 MHz, CDCl$_3$)
1.95
1.00
2.01
1.04
1.03
2.25
2.22
1.96
1.12
3.09
1.16
f1 (ppm)

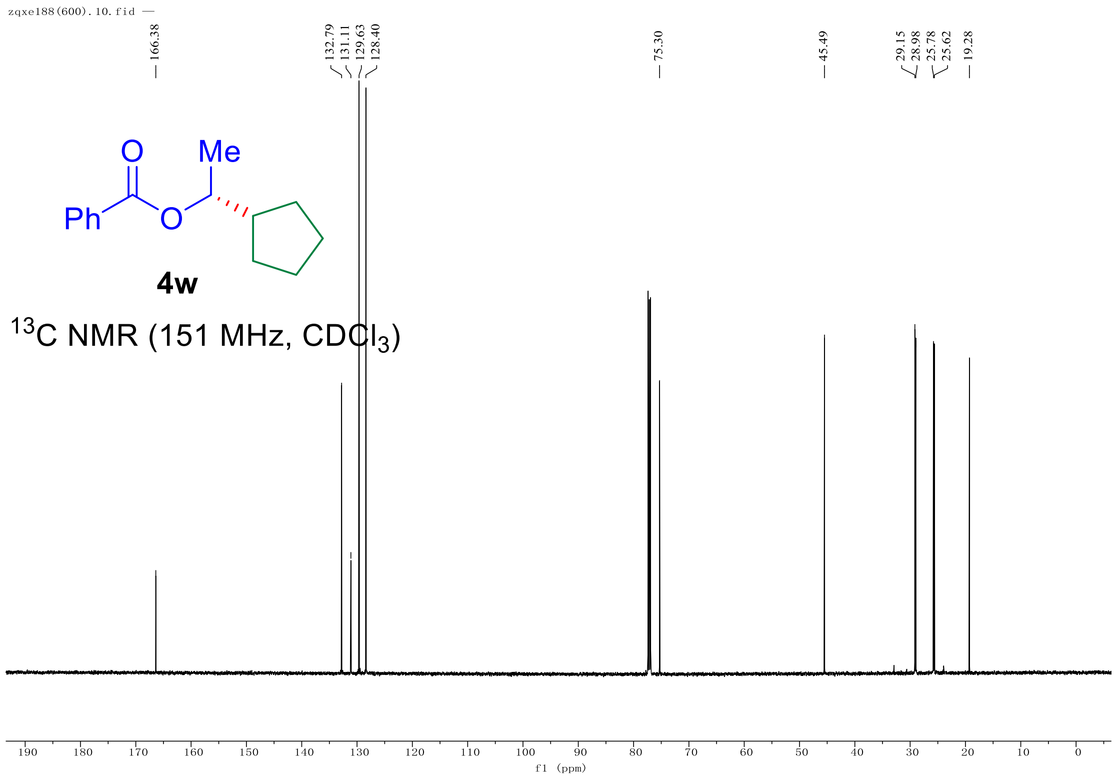
zqxe188(600).10.fid
166.38
132.79
131.11
129.63
128.40
75.30
45.49
29.15
28.98
25.78
25.62
19.28
O
Me
Ph
O
4w
13C NMR (151 MHz, CDCl3)
f1 (ppm)

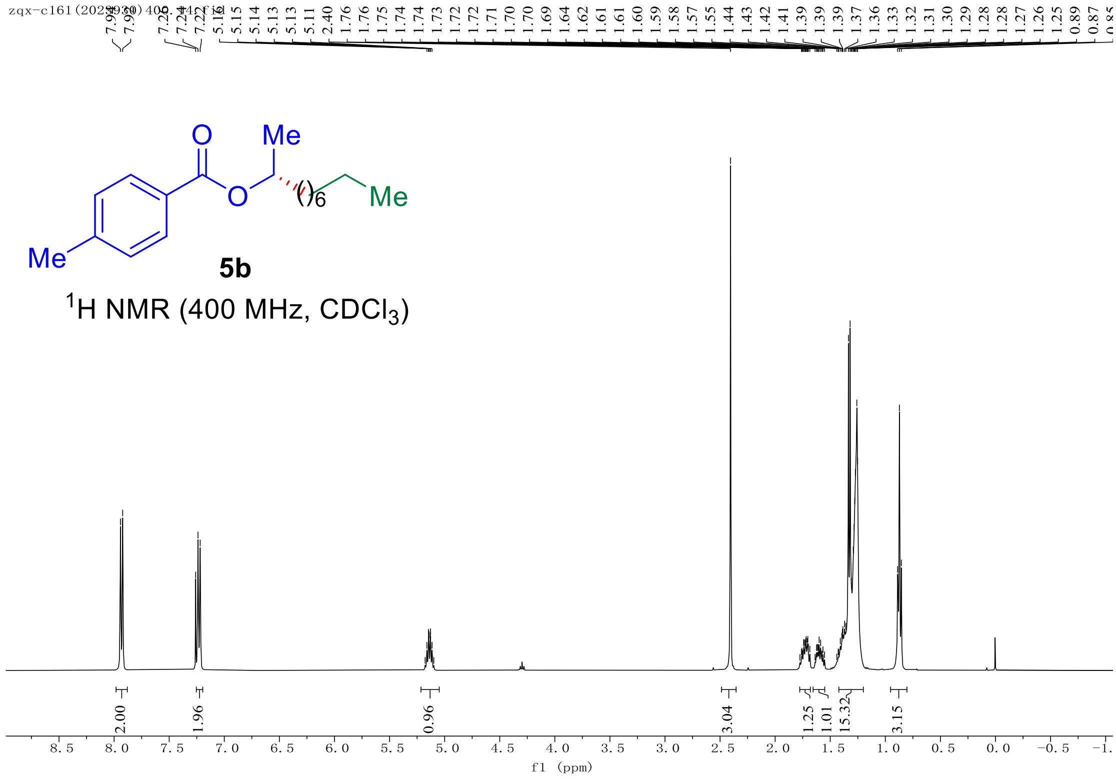
O
Me
O
Me
Me
5b
1H NMR (400 MHz, CDCl3)
2.00
1.96
0.96
3.04
1.25
1.01
15.32
3.15
f1 (ppm)

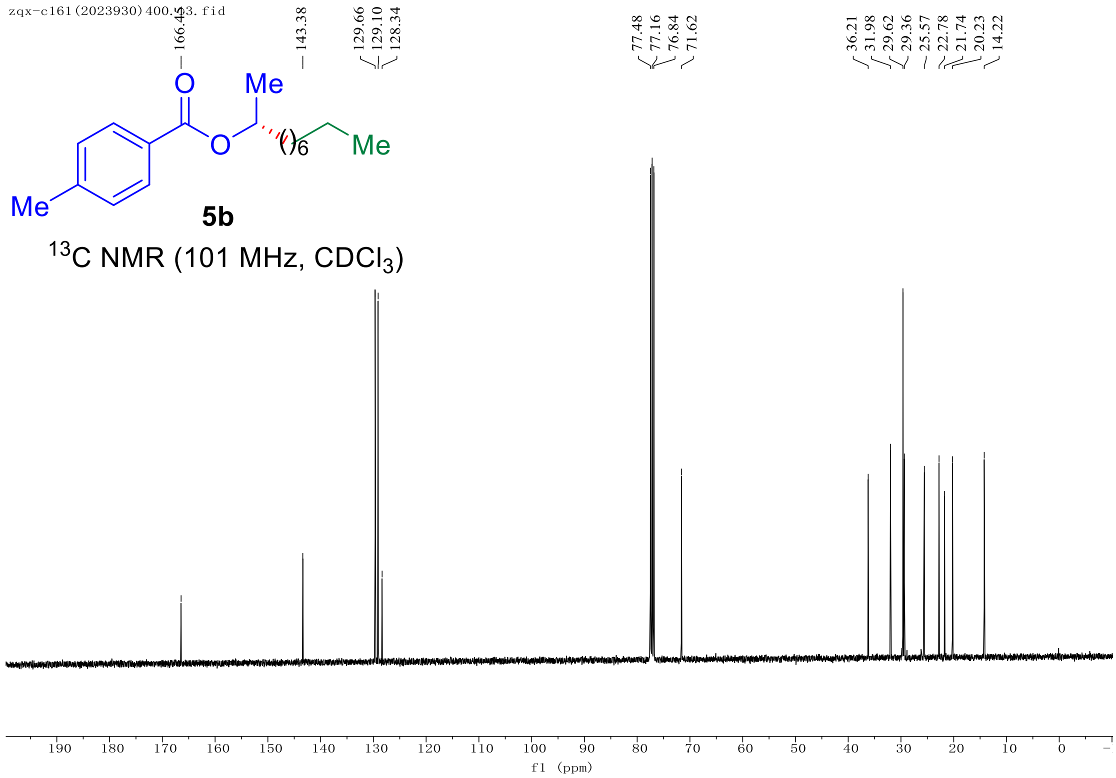

5b

^{13}C NMR (101 MHz, $CDCl_3$)

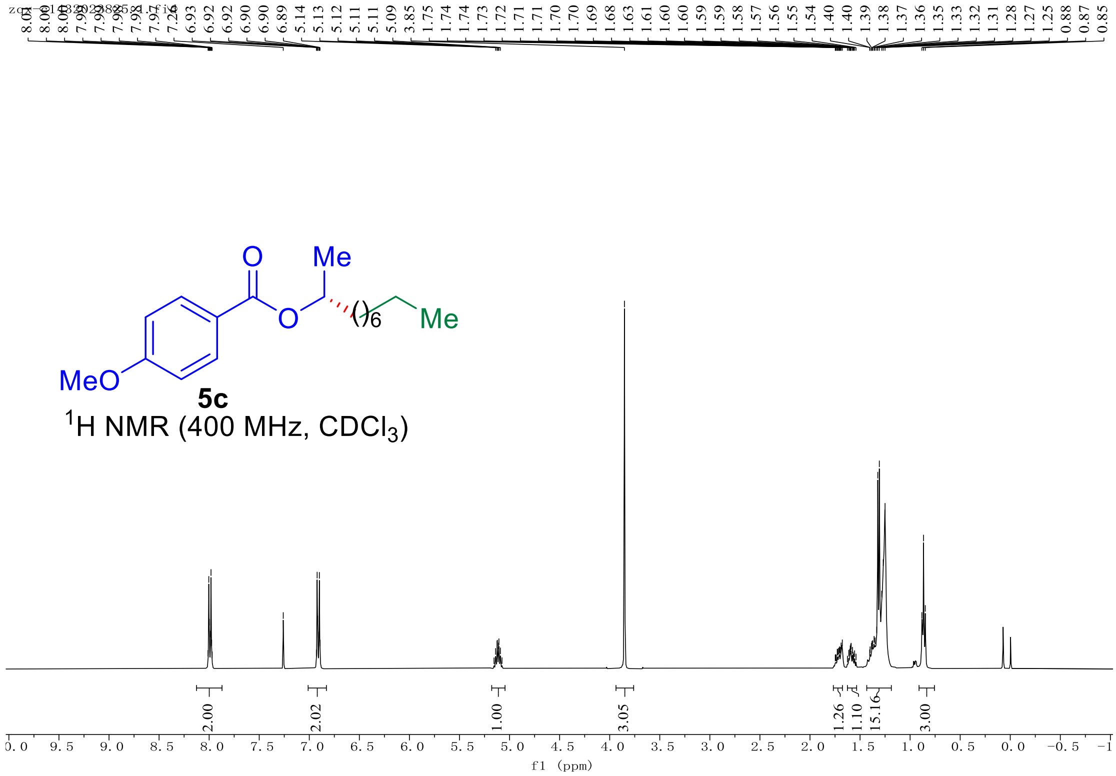

5c

^{1}H NMR (400 MHz, $CDCl_3$)

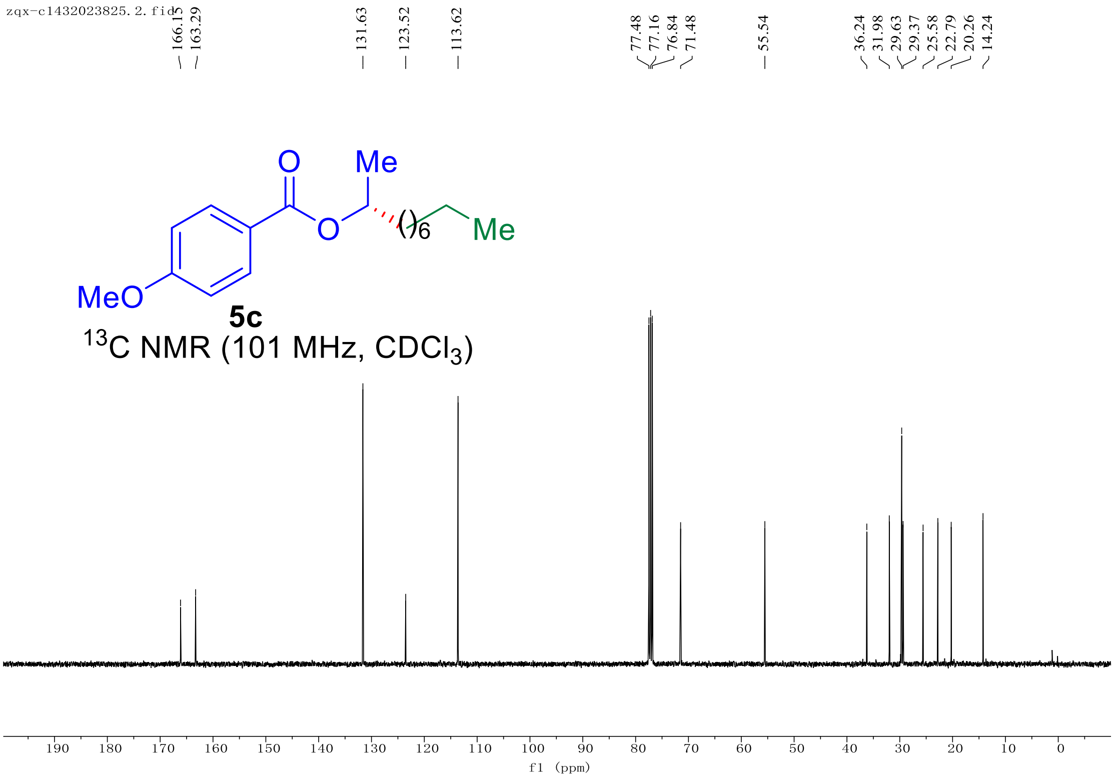
zqx-c1432023825.2.fid
166.15
163.29
131.63
123.52
113.62
77.48
77.16
76.84
71.48
55.54
36.24
31.98
29.63
29.37
25.58
22.79
20.26
14.24
O
Me
O
()6
Me
MeO
5c
13C NMR (101 MHz, CDCl3)
f1 (ppm)

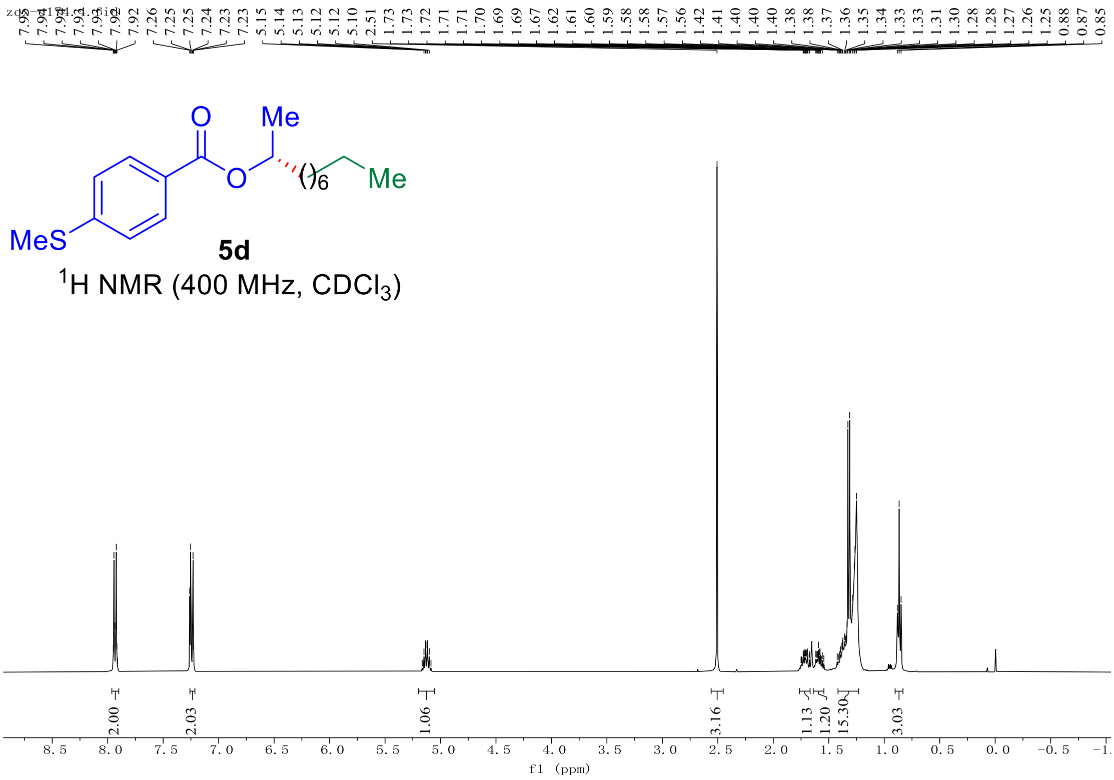
7.26
7.25
7.25
7.24
7.23
7.23
5.15
5.14
5.13
5.12
5.12
5.10
2.51
1.73
1.73
1.72
1.71
1.71
1.70
1.69
1.69
1.67
1.62
1.61
1.60
1.59
1.58
1.58
1.57
1.56
1.42
1.41
1.40
1.40
1.40
1.38
1.38
1.37
1.36
1.35
1.34
1.33
1.33
1.31
1.30
1.28
1.28
1.27
1.26
1.25
0.88
0.87
0.85
O
Me
O
()6
Me
MeS
5d
1H NMR (400 MHz, CDCl3)
2.00
2.03
1.06
3.16
1.13
1.20
15.30
3.03
f1 (ppm)

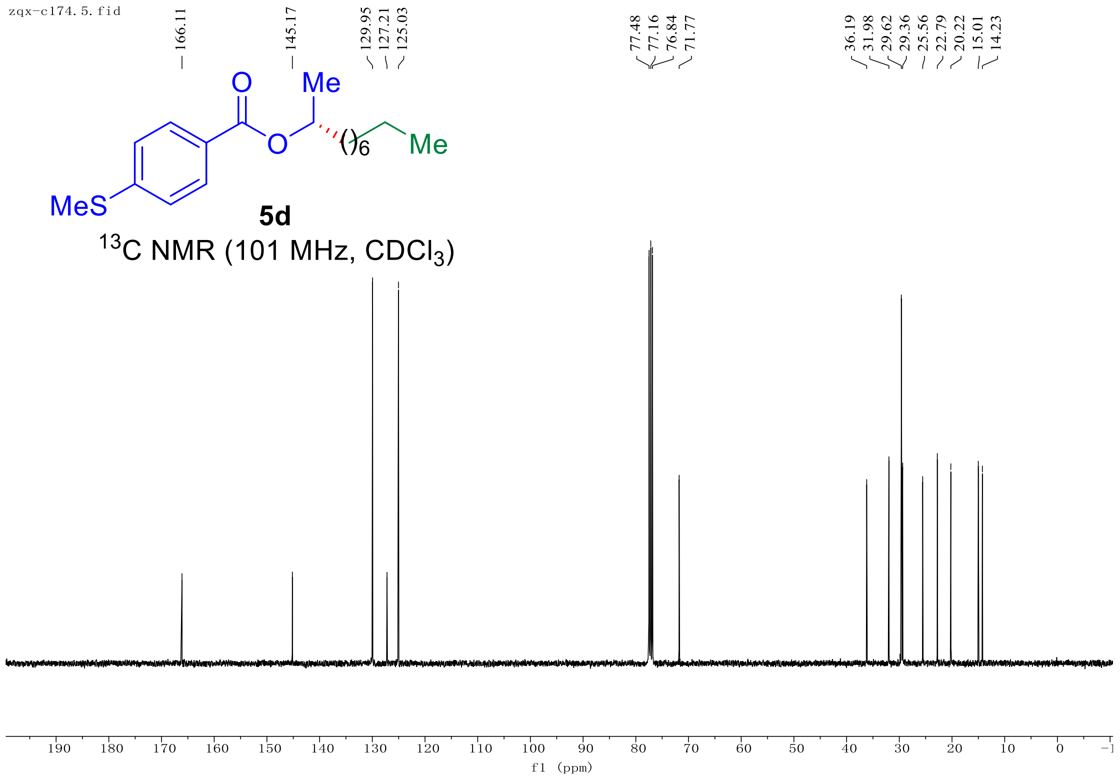
zqx-c174.5.fid
166.11
145.17
129.95
127.21
125.03
77.48
77.16
76.84
71.77
36.19
31.98
29.62
29.36
25.56
22.79
20.22
15.01
14.23
O
Me
O
()6
Me
MeS
5d
13C NMR (101 MHz, CDCl3)
190 180 170 160 150 140 130 120 110 100 90 80 70 60 50 40 30 20 10 0
f1 (ppm)

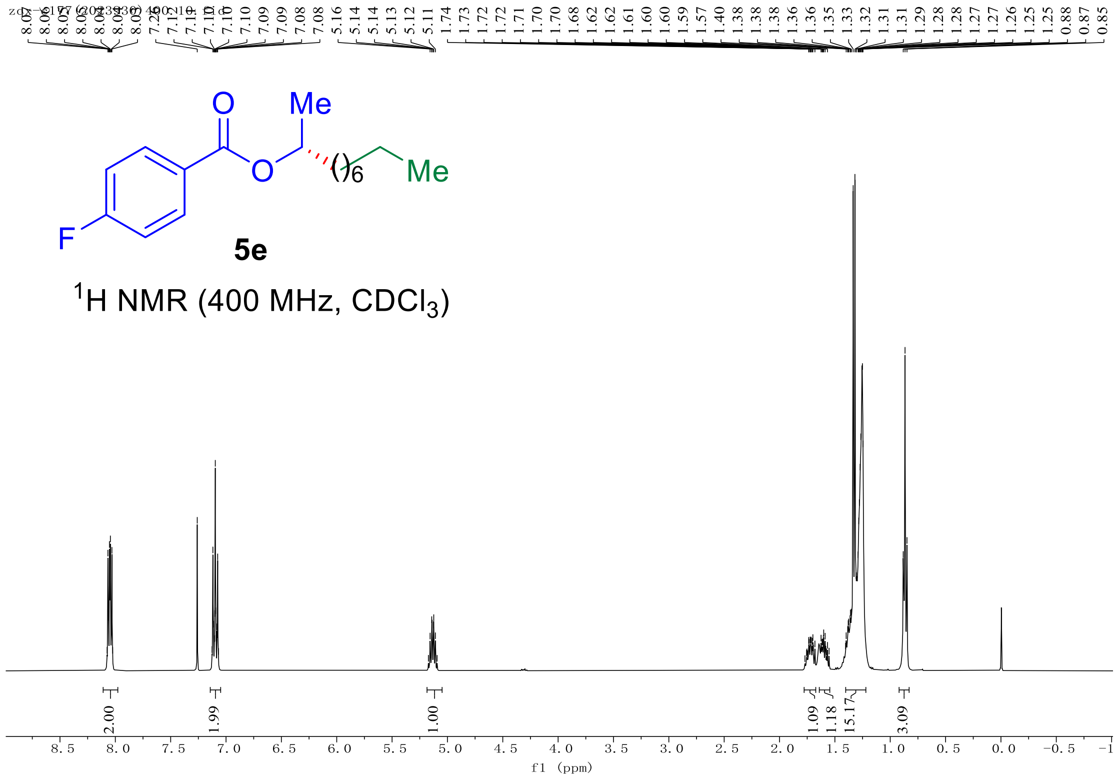
O
Me
O
()6
Me
F
5e
1H NMR (400 MHz, CDCl3)
2.00
1.99
1.00
1.09
1.18
15.17
3.09
8.5 8.0 7.5 7.0 6.5 6.0 5.5 5.0 4.5 4.0 3.5 3.0 2.5 2.0 1.5 1.0 0.5 0.0 −0.5
f1 (ppm)

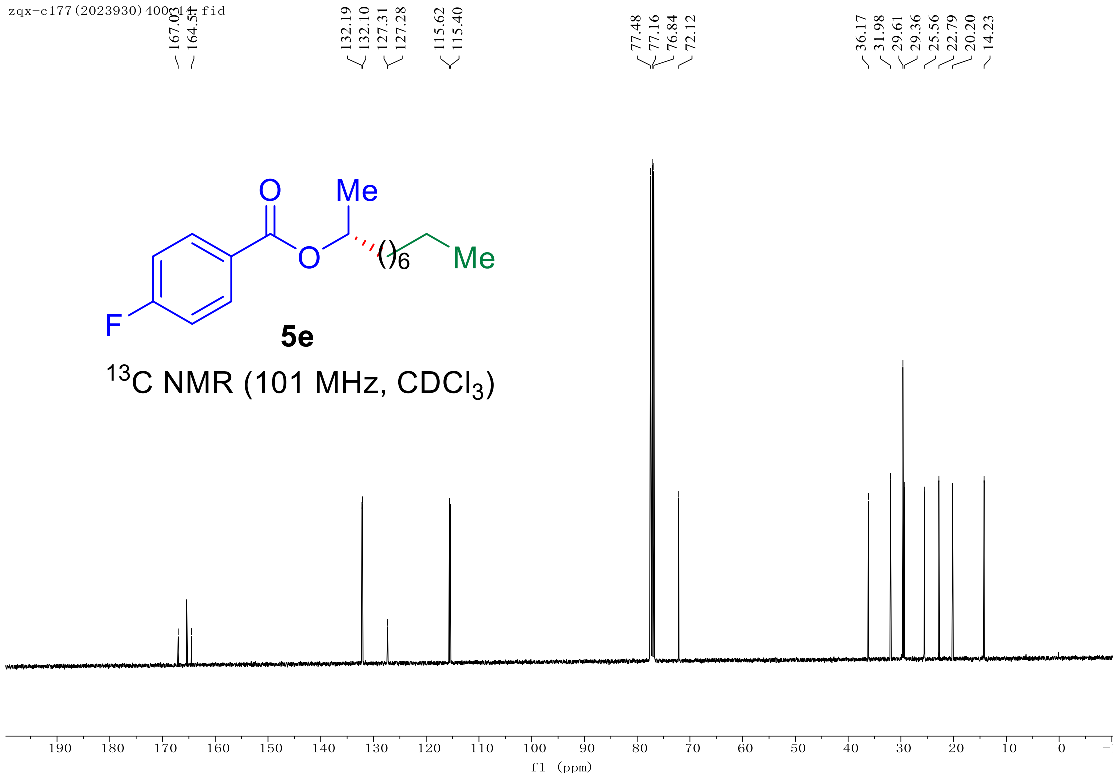

zqx-c177(2023930)400.11.fid
167.03
164.51
132.19
132.10
127.31
127.28
115.62
115.40
77.48
77.16
76.84
72.12
36.17
31.98
29.61
29.36
25.56
22.79
20.20
14.23
O
Me
O
Me
6
F
5e
13C NMR (101 MHz, CDCl3)
f1 (ppm)

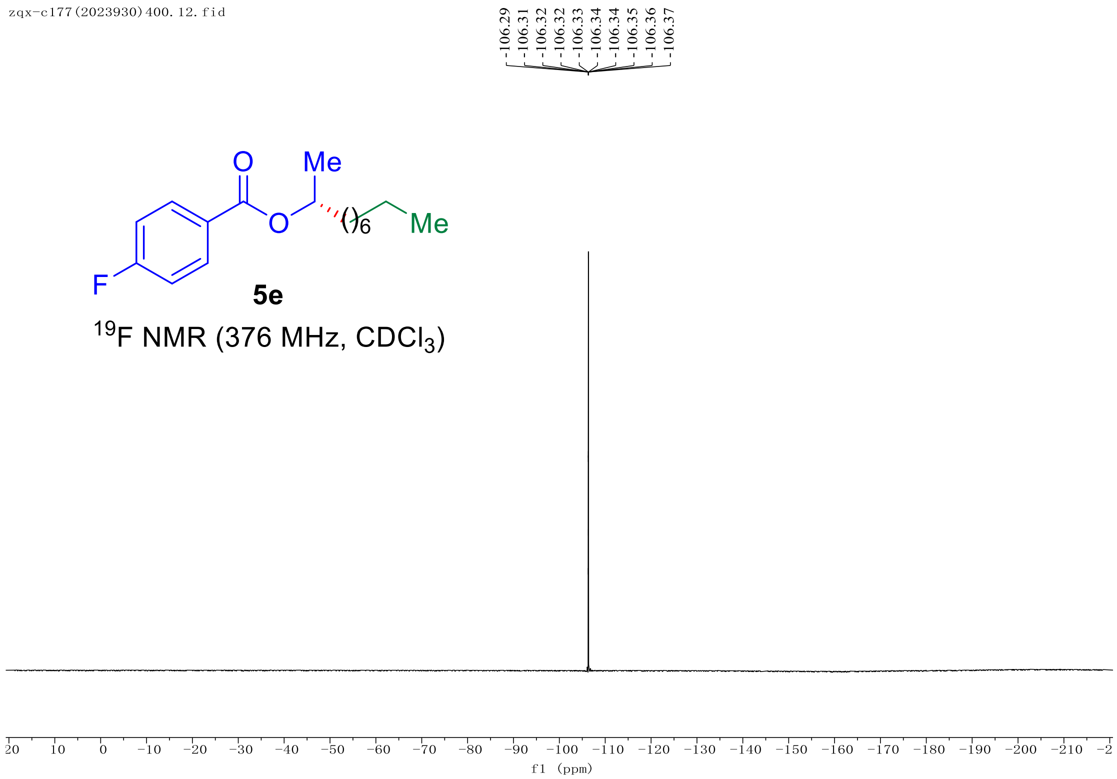

zqx-c177(2023930)400.12.fid
-106.29
-106.31
-106.32
-106.32
-106.33
-106.34
-106.34
-106.35
-106.36
-106.37
O
Me
O
Me
6
F
5e
19F NMR (376 MHz, CDCl3)
f1 (ppm)

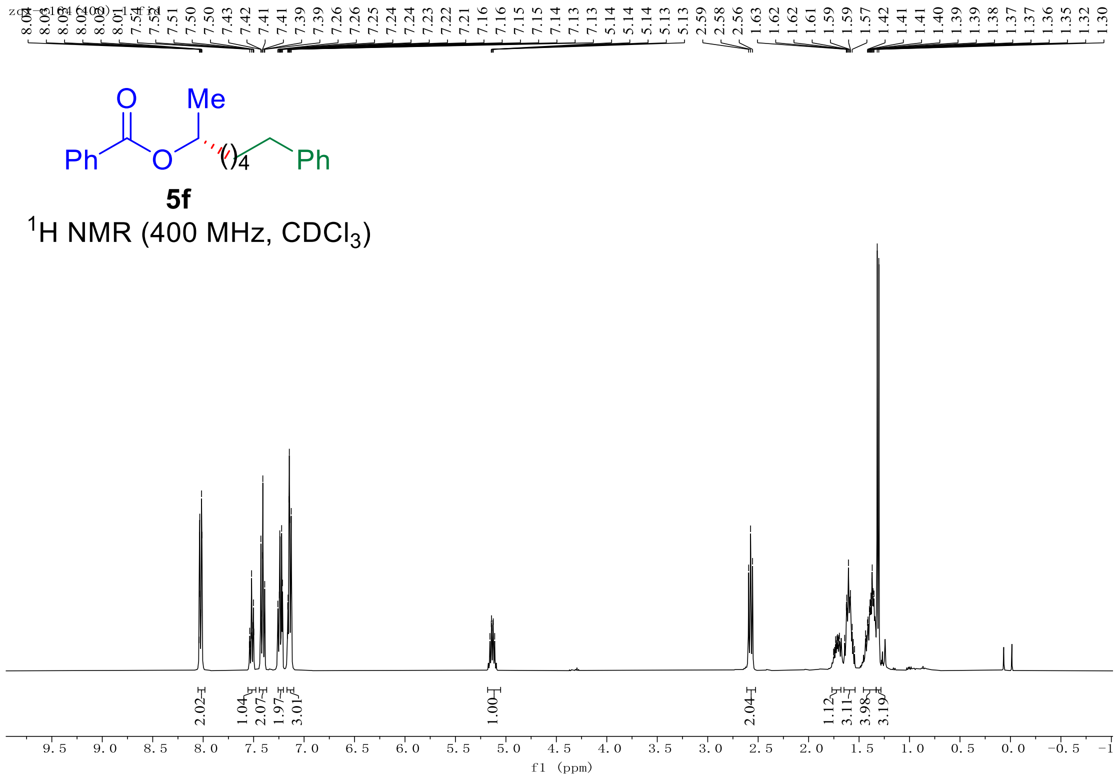
8.04 8.03 8.03 8.02 8.02 8.01 7.54 7.52 7.51 7.50 7.50 7.43 7.42 7.41 7.41 7.39 7.39 7.26 7.26 7.25 7.24 7.24 7.23 7.22 7.21 7.16 7.16 7.15 7.15 7.14 7.13 7.13 5.14 5.14 5.14 5.13 5.13 2.59 2.58 2.56 1.63 1.62 1.62 1.61 1.59 1.59 1.57 1.42 1.41 1.41 1.40 1.39 1.39 1.38 1.37 1.37 1.36 1.35 1.32 1.30
O
Me
Ph
O
4
Ph
5f
1H NMR (400 MHz, CDCl3)
2.02 1.04 2.07 1.97 3.01 1.00 2.04 1.12 3.11 3.98 3.19
f1 (ppm)

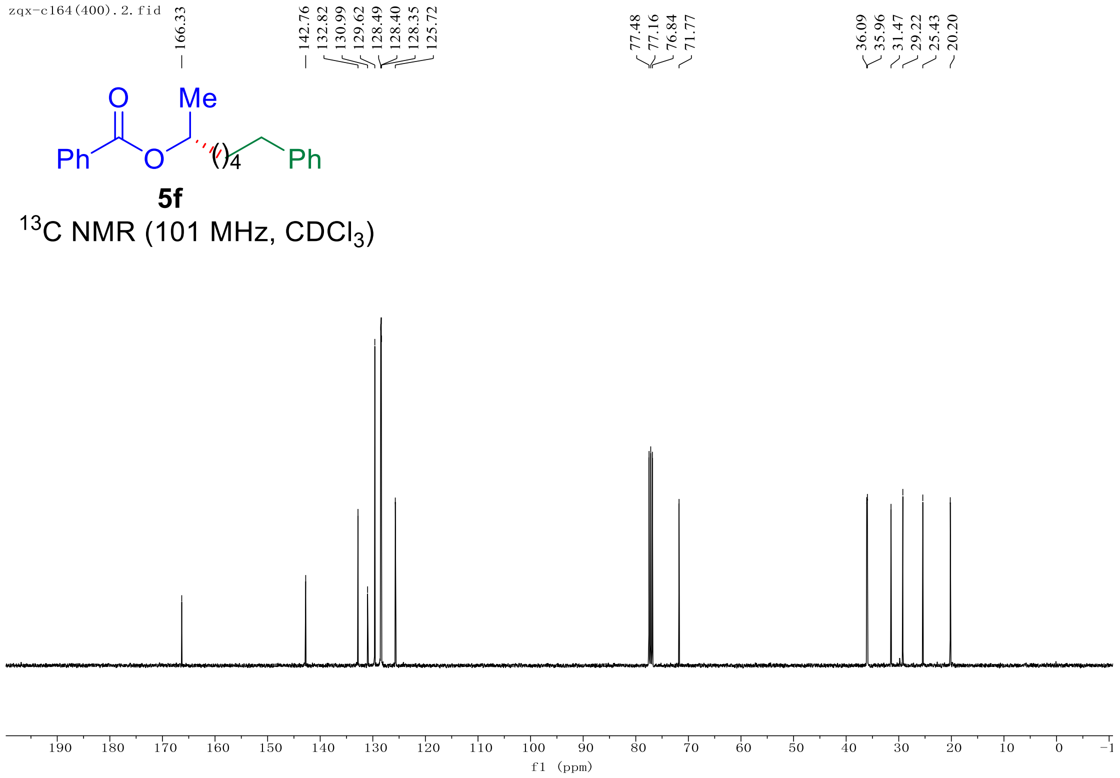
zqx-c164(400).2.fid
166.33
142.76 132.82 130.99 129.62 128.49 128.40 128.35 125.72
77.48 77.16 76.84 71.77
36.09 35.96 31.47 29.22 25.43 20.20
O
Me
Ph
O
4
Ph
5f
13C NMR (101 MHz, CDCl3)
f1 (ppm)

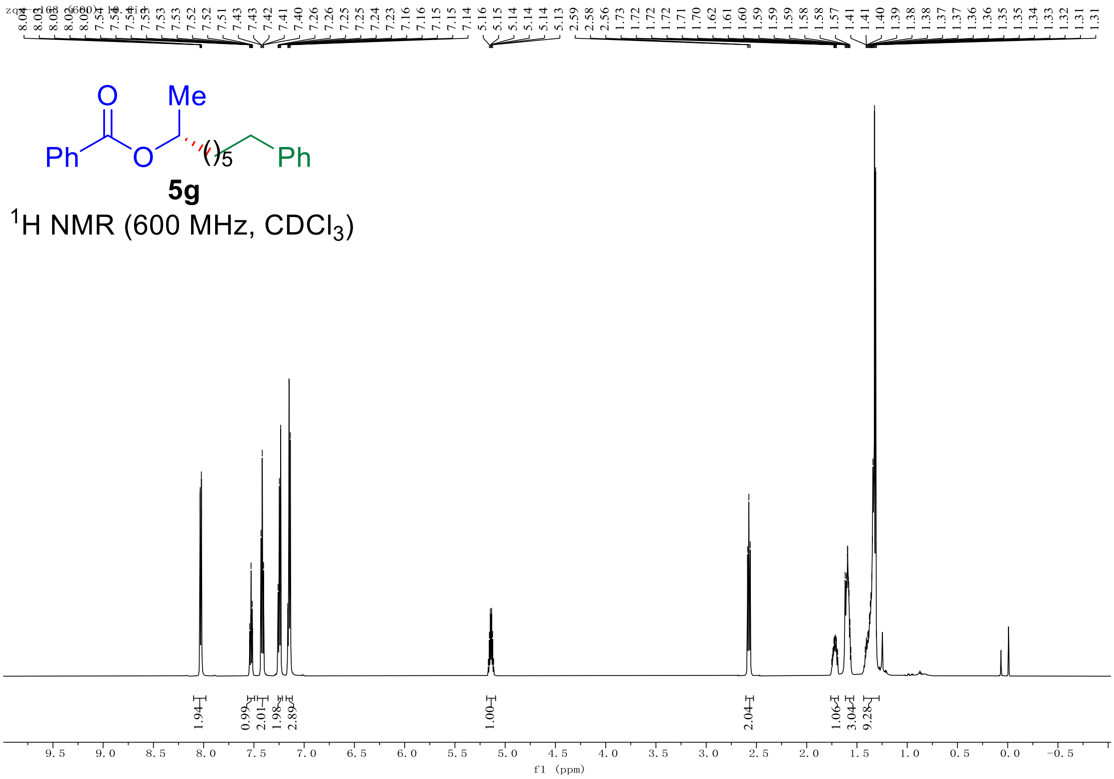
8.04 8.03 8.03 8.02 7.54 7.54 7.53 7.53 7.52 7.52 7.51 7.43 7.42 7.41 7.40 7.26 7.26 7.25 7.25 7.24 7.23 7.16 7.16 7.15 7.15 7.14 5.16 5.15 5.14 5.14 5.14 5.13 2.59 2.58 2.56 1.73 1.72 1.72 1.71 1.70 1.62 1.61 1.60 1.59 1.59 1.59 1.58 1.57 1.41 1.41 1.40 1.39 1.38 1.38 1.37 1.37 1.36 1.36 1.35 1.35 1.34 1.33 1.32 1.31 1.31
O
Me
Ph
O
5
Ph
5g
1H NMR (600 MHz, CDCl3)
1.94 0.99 2.01 1.98 2.89 1.00 2.04 1.06 3.04 9.28
9.5 9.0 8.5 8.0 7.5 7.0 6.5 6.0 5.5 5.0 4.5 4.0 3.5 3.0 2.5 2.0 1.5 1.0 0.5 0.0 -0.5
f1 (ppm)

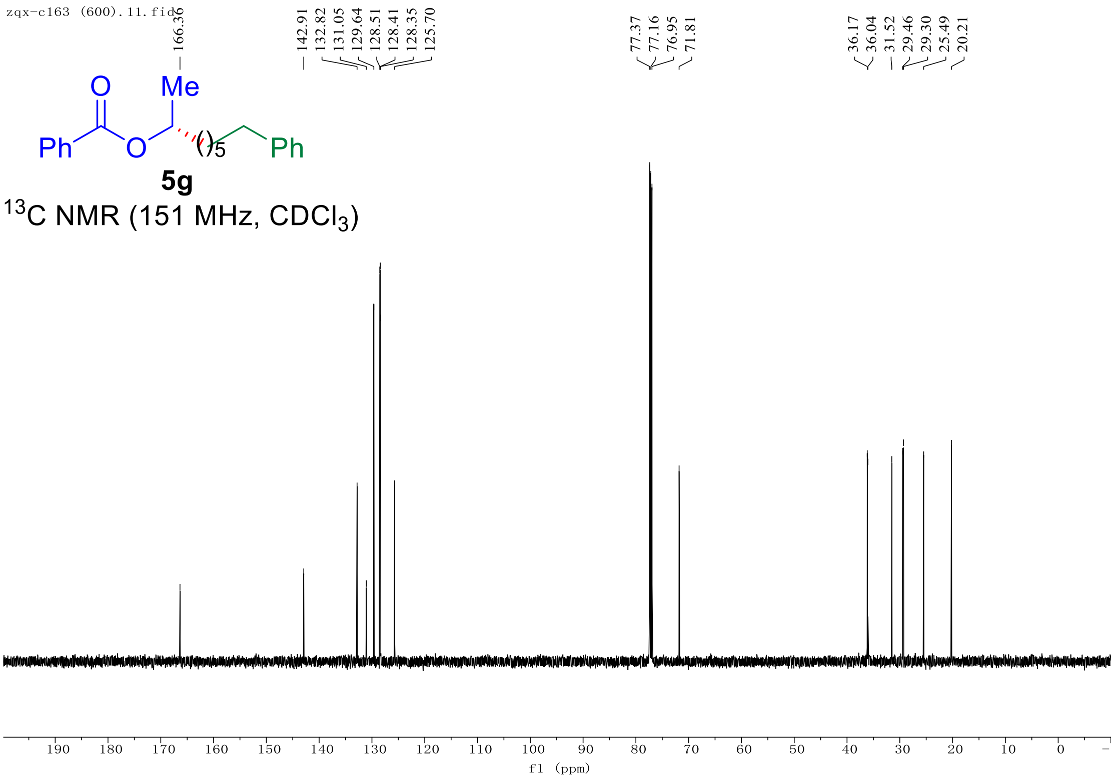
zqx-c163 (600).11.fid
166.36
142.91 132.82 131.05 129.64 128.51 128.41 128.35 125.70
77.37 77.16 76.95 71.81
36.17 36.04 31.52 29.46 29.30 25.49 20.21
O
Me
Ph
O
5
Ph
5g
13C NMR (151 MHz, CDCl3)
190 180 170 160 150 140 130 120 110 100 90 80 70 60 50 40 30 20 10 0
f1 (ppm)

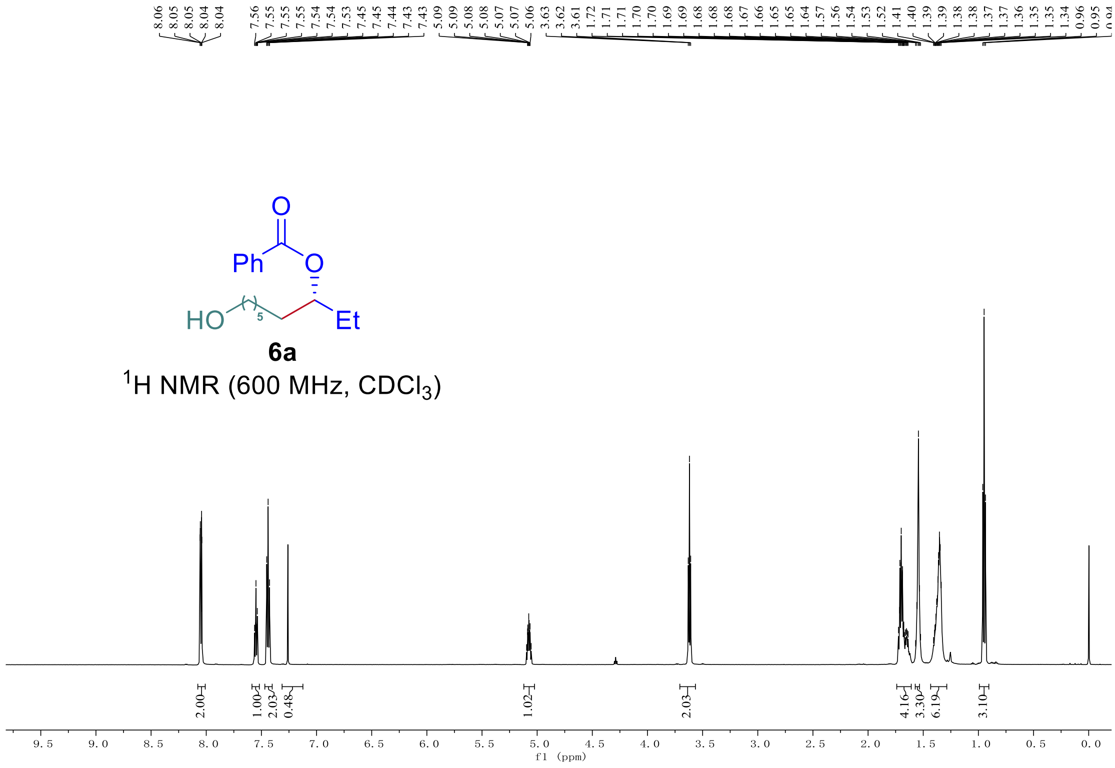

6a
^{1}H NMR (600 MHz, CDCl$_3$)

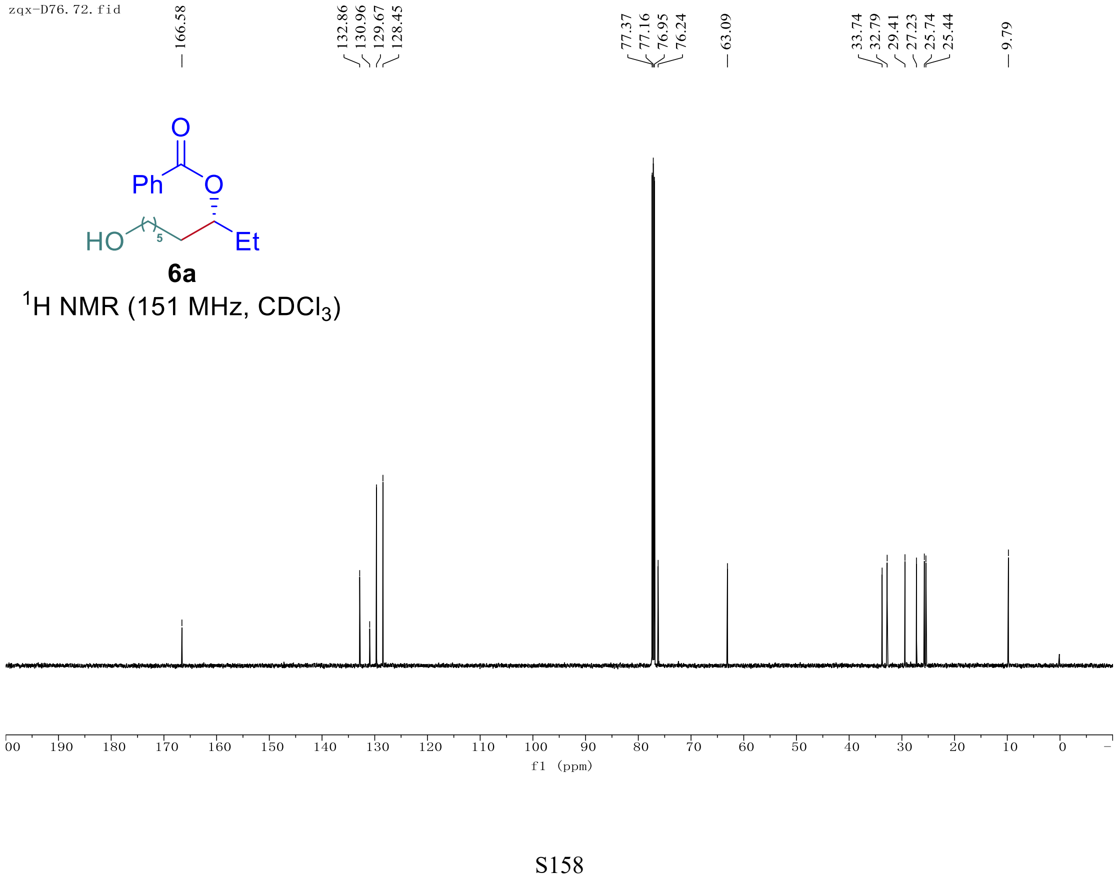

zqx-D76.72.fid
6a
^{1}H NMR (151 MHz, CDCl$_3$)

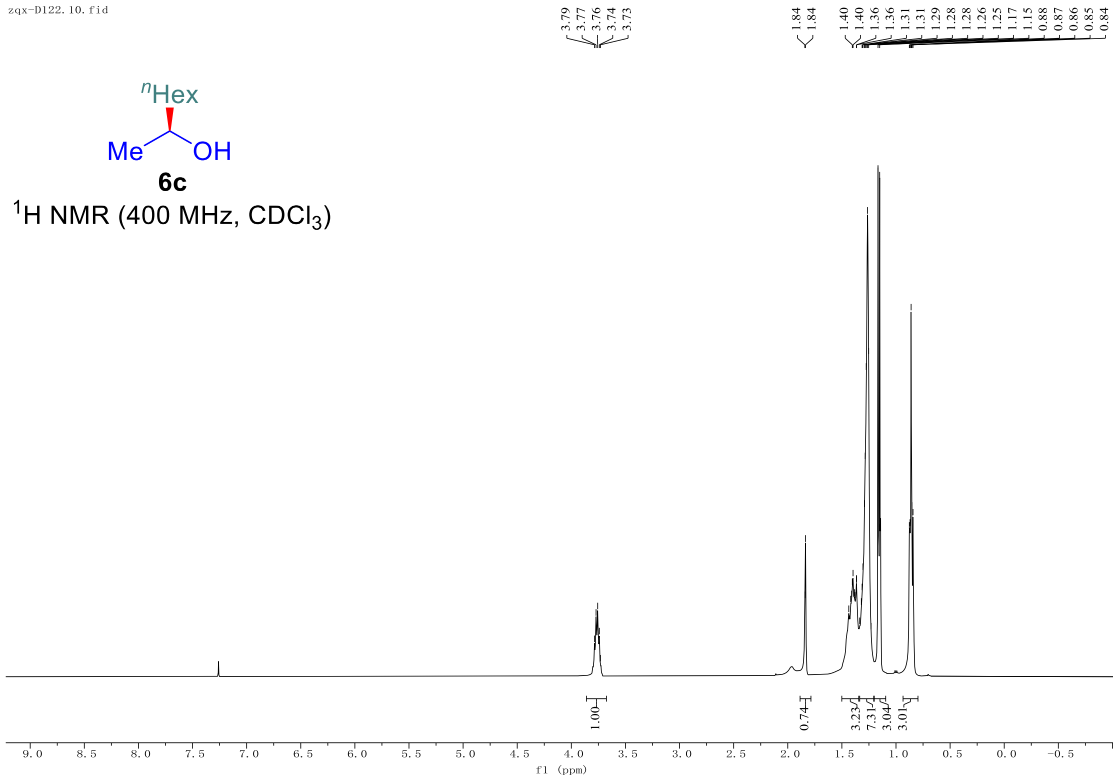
zqx-D122.10.fid
3.79
3.77
3.76
3.74
3.73
1.84
1.84
1.40
1.40
1.36
1.36
1.31
1.31
1.29
1.28
1.28
1.26
1.25
1.17
1.15
0.88
0.87
0.86
0.85
0.84
nHex
Me
OH
6c
1H NMR (400 MHz, CDCl3)
1.00
0.74
3.23
7.31
3.04
3.01
f1 (ppm)

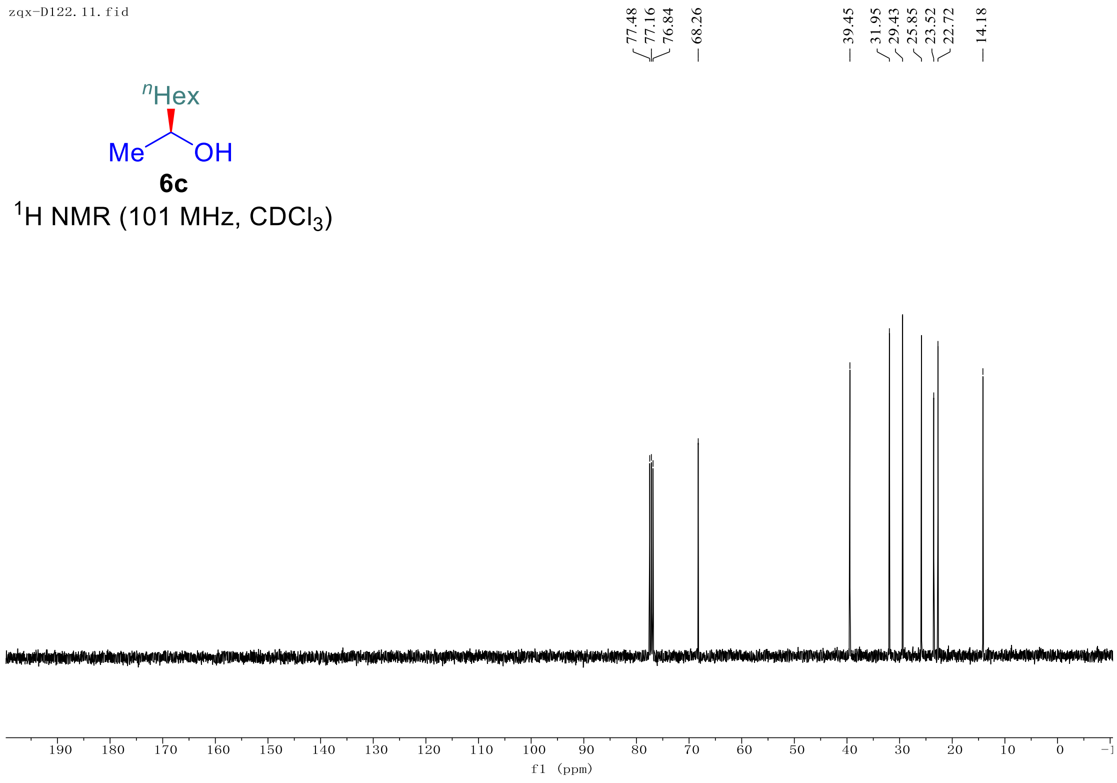
zqx-D122.11.fid
77.48
77.16
76.84
68.26
39.45
31.95
29.43
25.85
23.52
22.72
14.18
nHex
Me
OH
6c
1H NMR (101 MHz, CDCl3)
f1 (ppm)

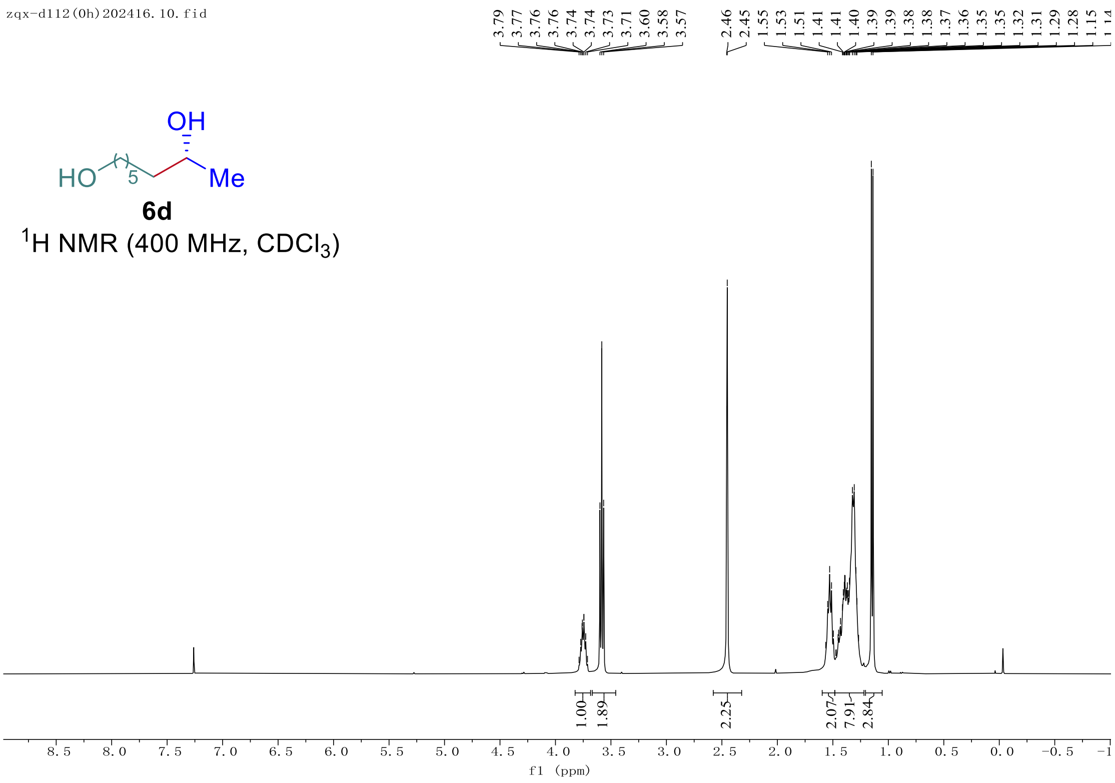
zqx-d112(0h)202416.10.fid
3.79
3.77
3.76
3.76
3.74
3.74
3.73
3.71
3.60
3.58
3.57
2.46
2.45
1.55
1.53
1.51
1.41
1.41
1.40
1.39
1.39
1.38
1.38
1.37
1.36
1.35
1.35
1.32
1.31
1.29
1.28
1.15
OH
HO
5
Me
6d
1H NMR (400 MHz, CDCl3)
1.00
1.89
2.25
2.07
7.91
2.84
8.5
8.0
7.5
7.0
6.5
6.0
5.5
5.0
4.5
4.0
3.5
3.0
2.5
2.0
1.5
1.0
0.5
0.0
-0.5
-1
f1 (ppm)

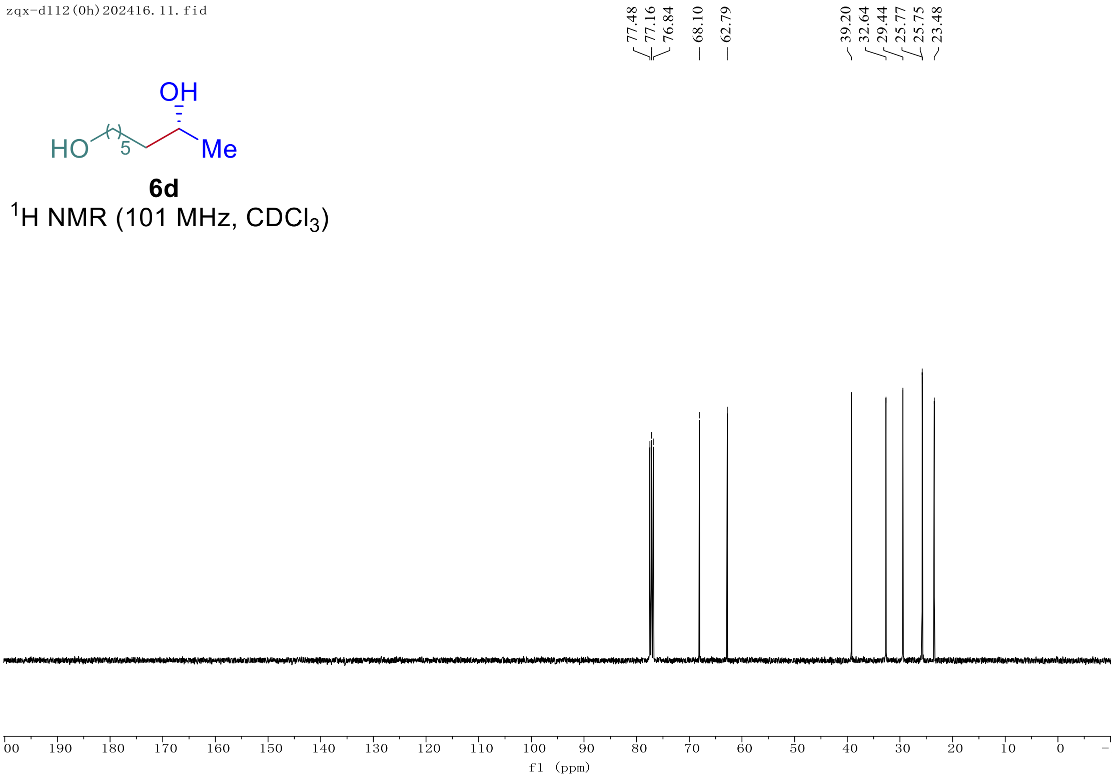
zqx-d112(0h)202416.11.fid
77.48
77.16
76.84
68.10
62.79
39.20
32.64
29.44
25.77
25.75
23.48
OH
HO
5
Me
6d
1H NMR (101 MHz, CDCl3)
00
190
180
170
160
150
140
130
120
110
100
90
80
70
60
50
40
30
20
10
0
f1 (ppm)

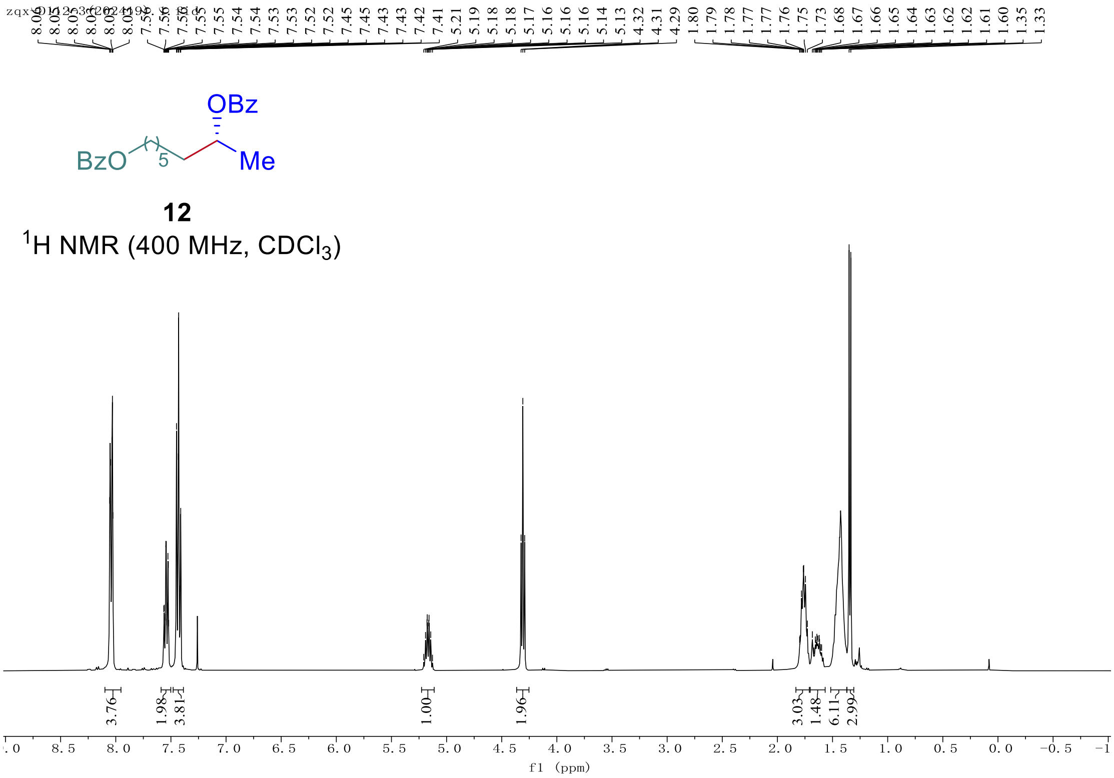

12

^{1}H NMR (400 MHz, $CDCl_3$)

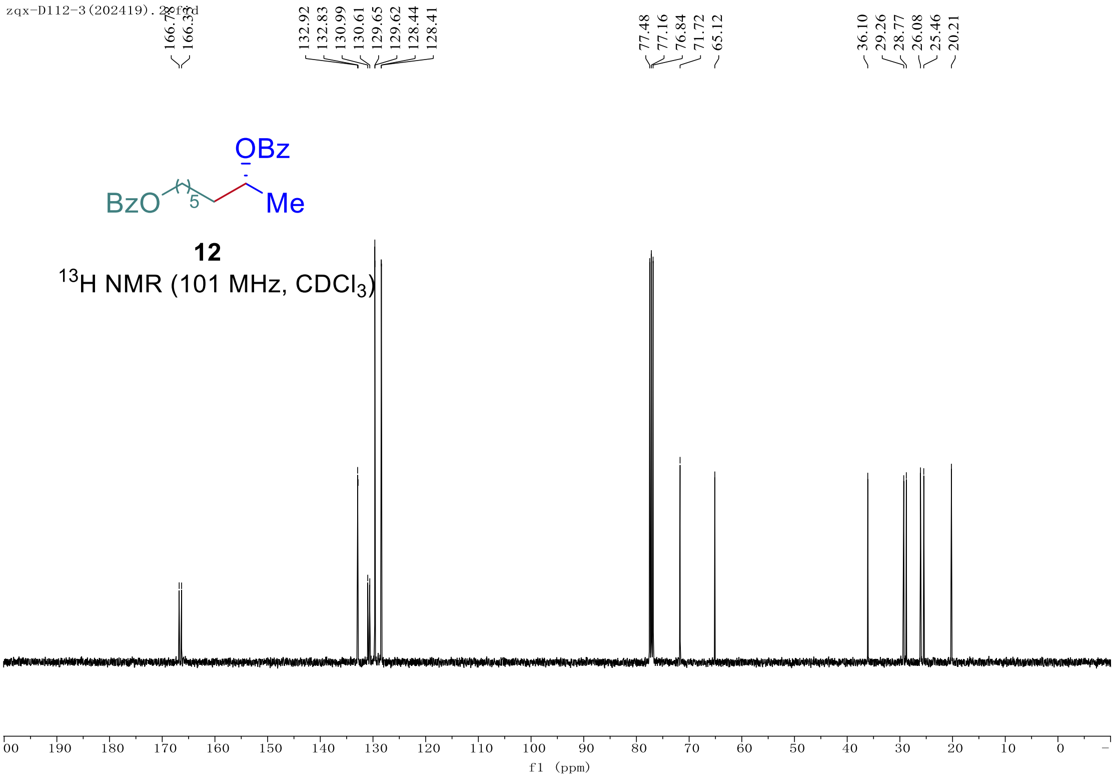

12

^{13}H NMR (101 MHz, $CDCl_3$)

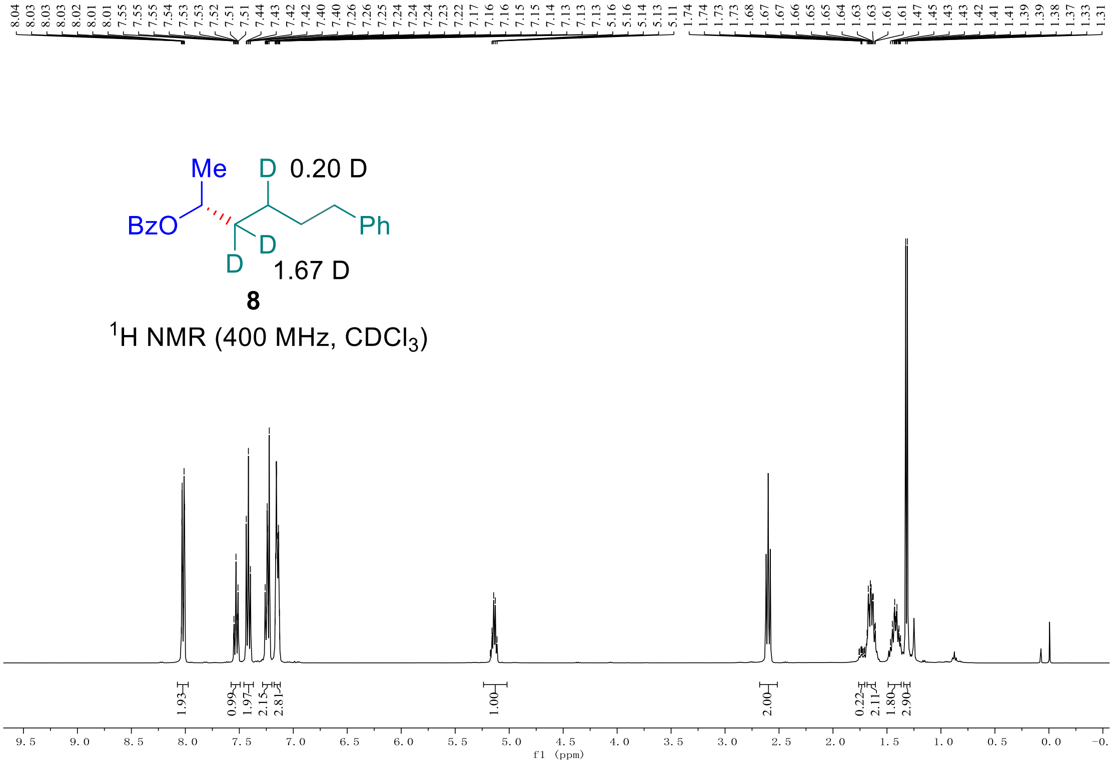
8.04 8.03 8.03 8.02 8.01 8.01 7.55 7.55 7.55 7.54 7.53 7.53 7.52 7.51 7.51 7.44 7.43 7.42 7.42 7.40 7.26 7.26 7.25 7.24 7.24 7.24 7.23 7.22 7.17 7.16 7.16 7.15 7.15 7.14 7.13 7.13 7.13 5.16 5.16 5.14 5.13 5.11 1.74 1.74 1.73 1.73 1.68 1.67 1.67 1.66 1.65 1.65 1.64 1.63 1.63 1.61 1.61 1.61 1.47 1.45 1.43 1.42 1.41 1.41 1.39 1.39 1.38 1.37 1.33 1.31
Me
D 0.20 D
BzO
Ph
D
D 1.67 D
8
¹H NMR (400 MHz, CDCl₃)
1.93
0.99 1.97 2.15 2.81
1.00
2.00
0.22 2.11 1.80 2.90
f1 (ppm)

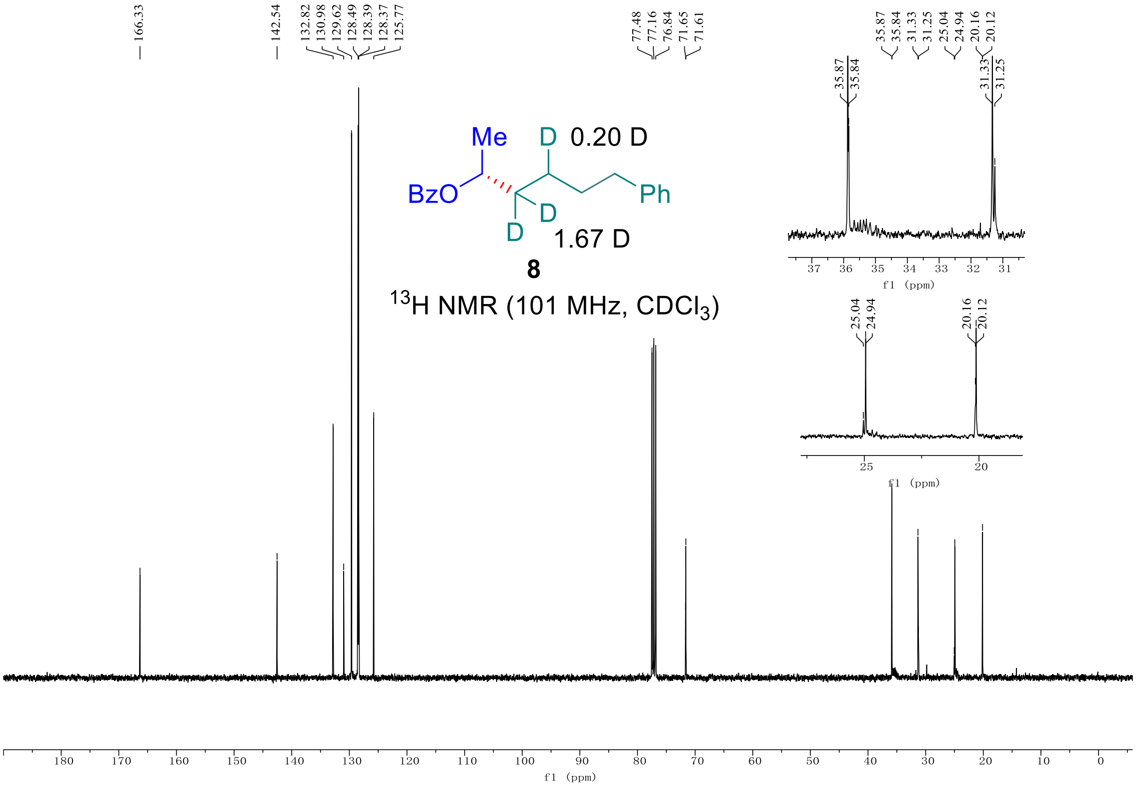
166.33
142.54
132.82 130.98 129.62 128.49 128.39 128.37 125.77
77.48 77.16 76.84 71.65 71.61
35.87 35.84 31.33 31.25 25.04 24.94 20.16 20.12
Me
D 0.20 D
BzO
Ph
D
D 1.67 D
8
¹³H NMR (101 MHz, CDCl₃)
f1 (ppm)

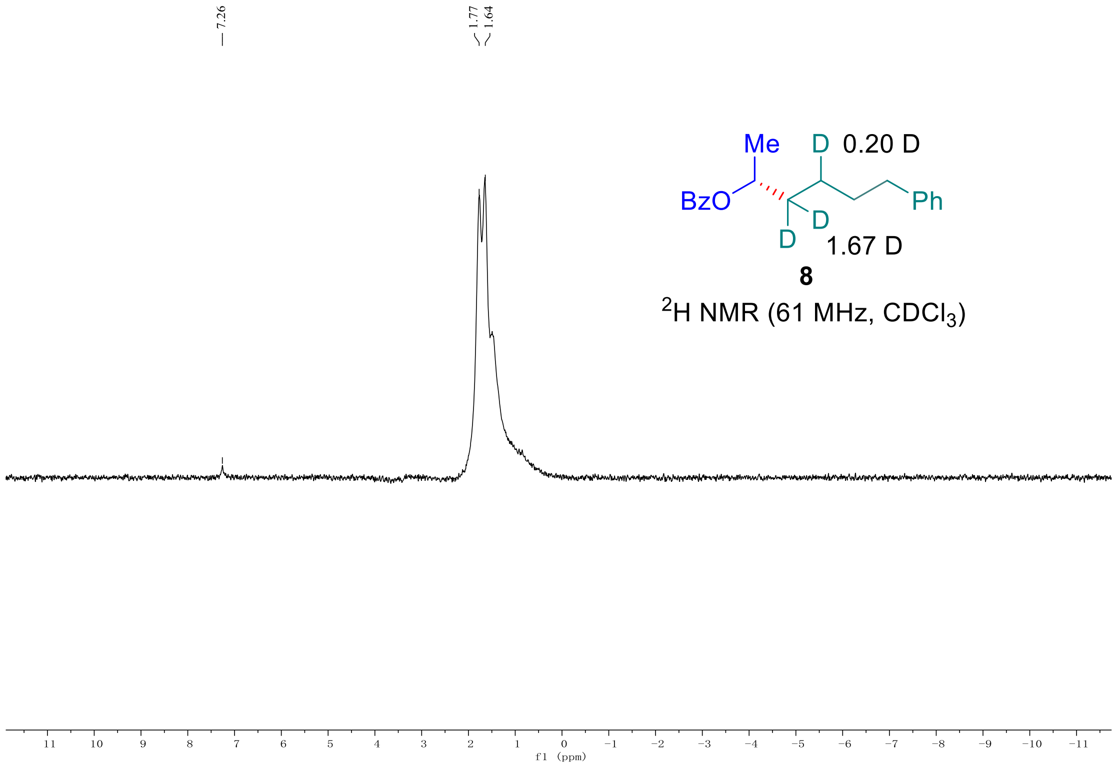

7.26
1.77
1.64
Me
D 0.20 D
BzO
Ph
D
D 1.67 D
8
^{2}H NMR (61 MHz, CDCl$_3$)
f1 (ppm)

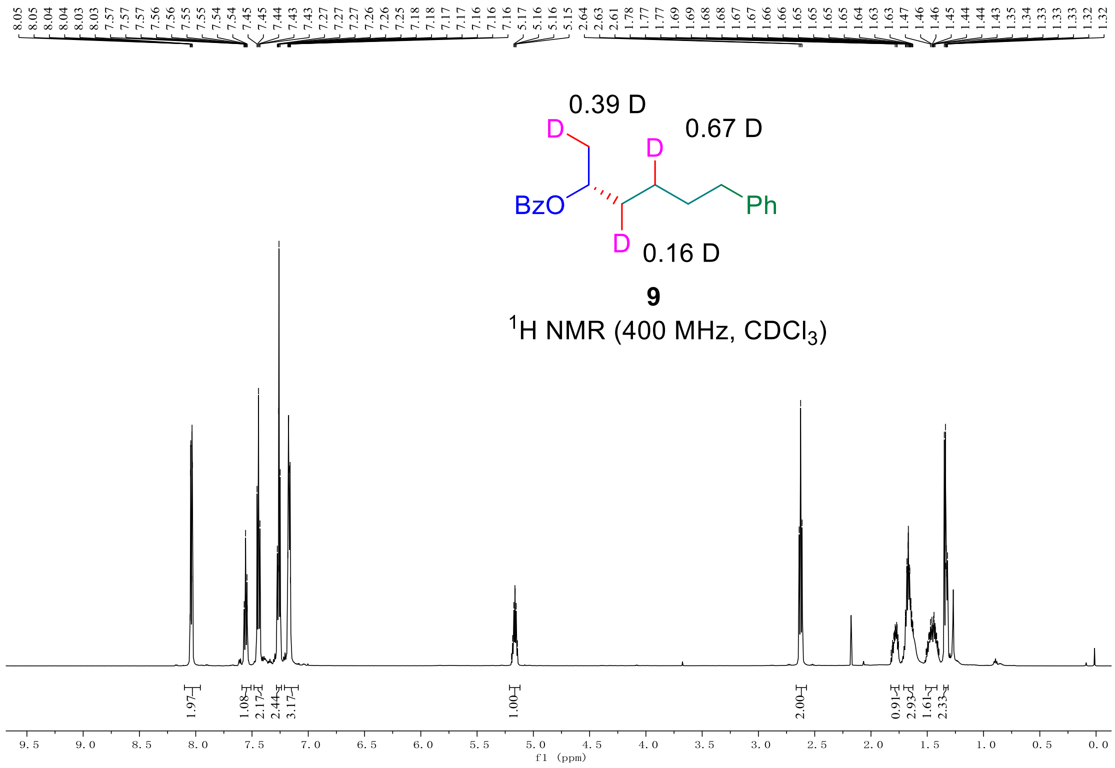

0.39 D
0.67 D
D
D
BzO
Ph
D 0.16 D
9
^{1}H NMR (400 MHz, CDCl$_3$)
1.97
1.08
2.17
2.44
3.17
1.00
2.00
0.91
2.93
1.61
2.33
f1 (ppm)

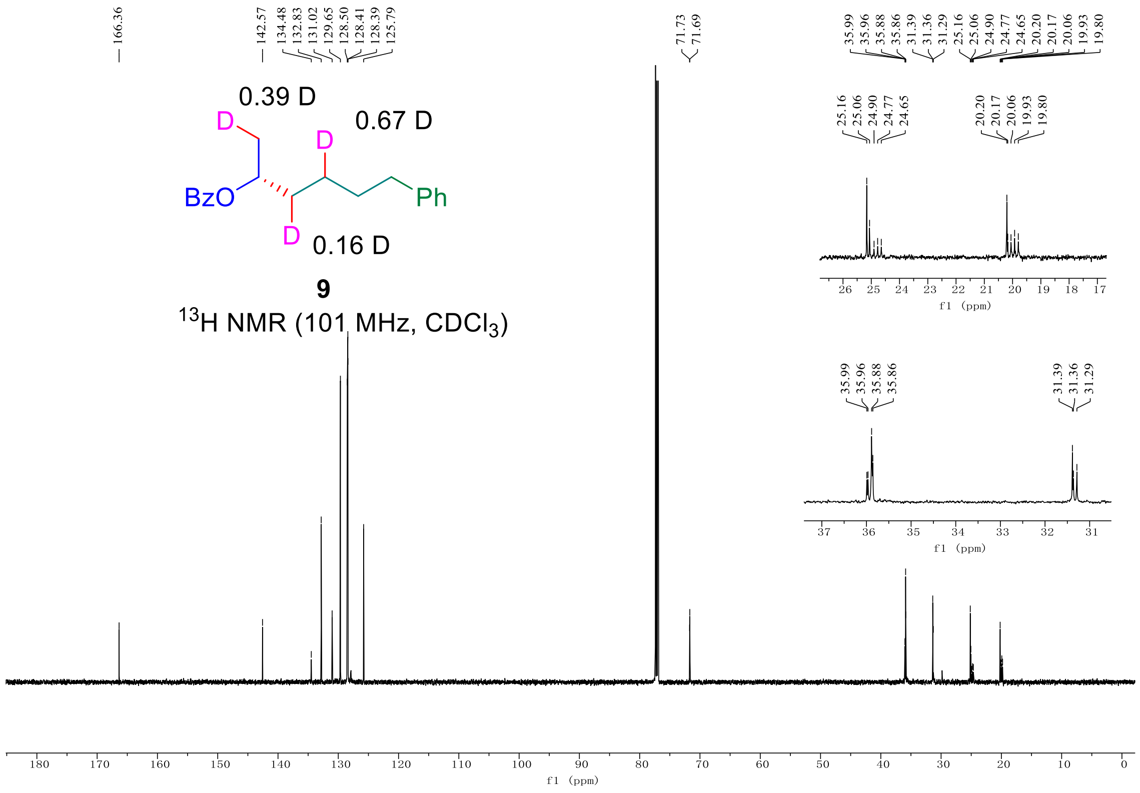
166.36
142.57
134.48
132.83
131.02
129.65
128.50
128.41
128.39
125.79
71.73
71.69
35.99
35.96
35.88
35.86
31.39
31.36
31.29
25.16
25.06
24.90
24.77
24.65
20.20
20.17
20.06
19.93
19.80
0.39 D
0.67 D
D
BzO
Ph
0.16 D
9
^{13}H NMR (101 MHz, CDCl$_3$)
f1 (ppm)

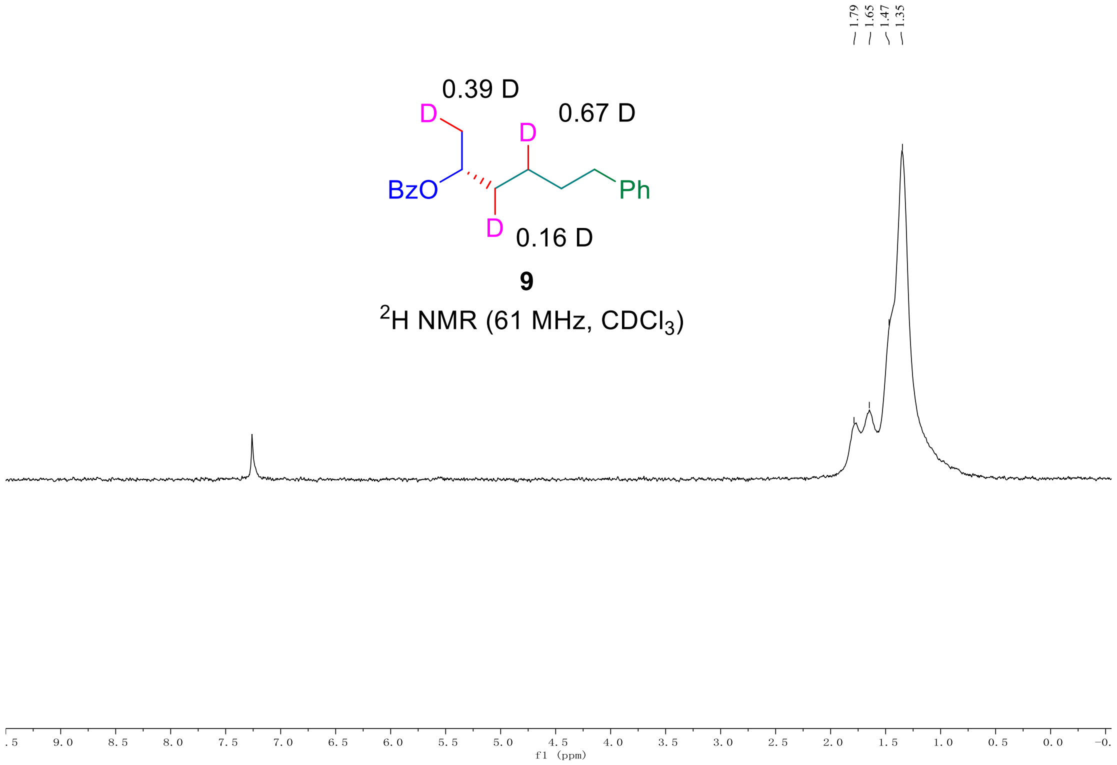
1.79
1.65
1.47
1.35
0.39 D
0.67 D
D
BzO
Ph
0.16 D
9
^{2}H NMR (61 MHz, CDCl$_3$)
f1 (ppm)

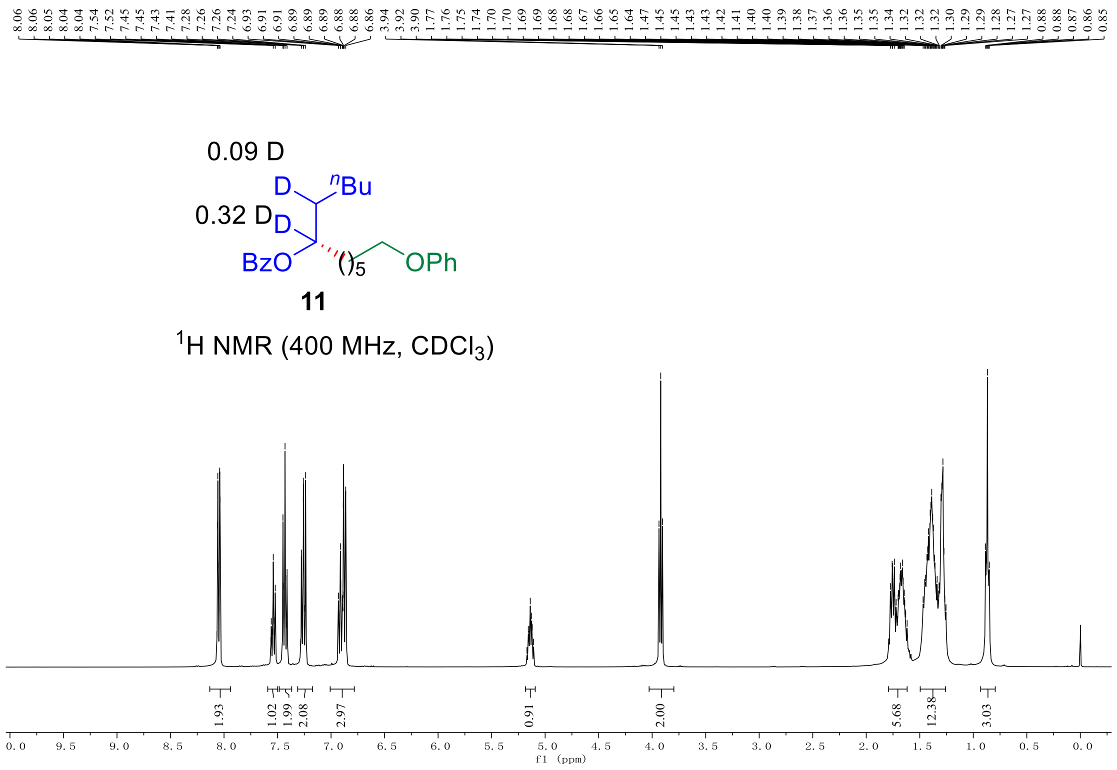
0.09 D
0.32 D
D
D
^{n}Bu
BzO
OPh
11
^{1}H NMR (400 MHz, $CDCl_3$)

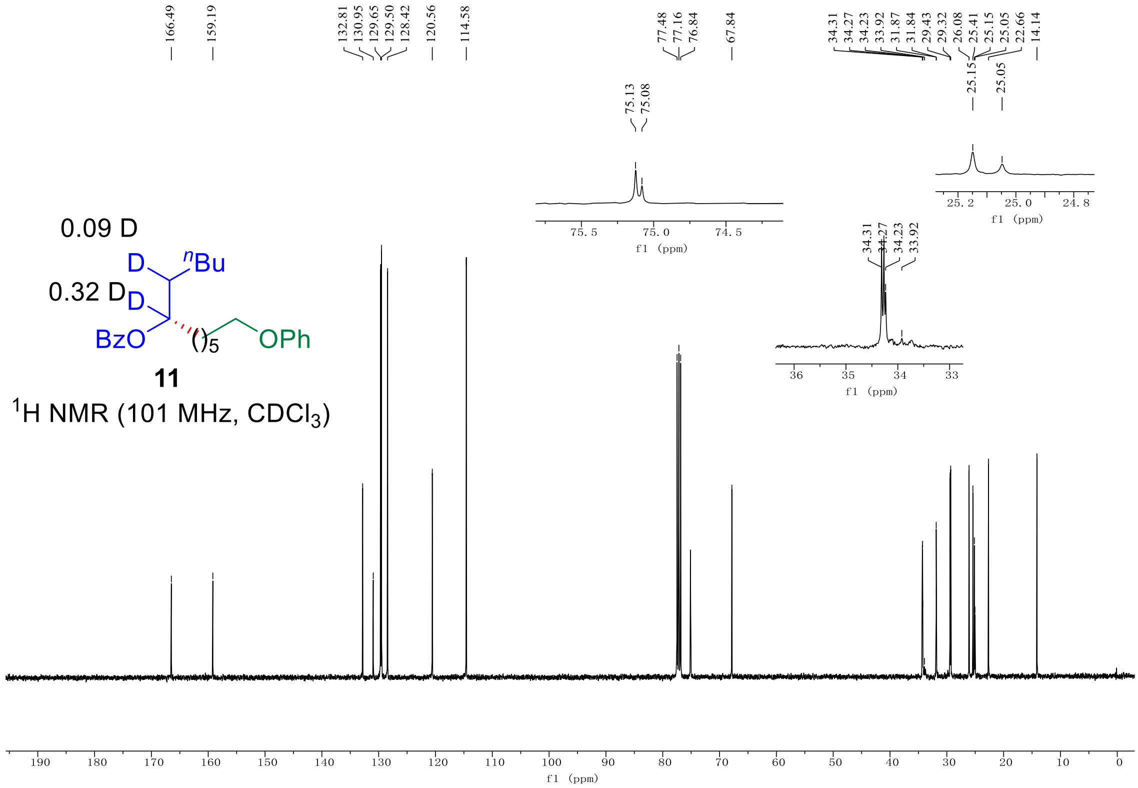
0.09 D
0.32 D
D
D
^{n}Bu
BzO
OPh
11
^{1}H NMR (101 MHz, $CDCl_3$)

XI. HPLC Traces

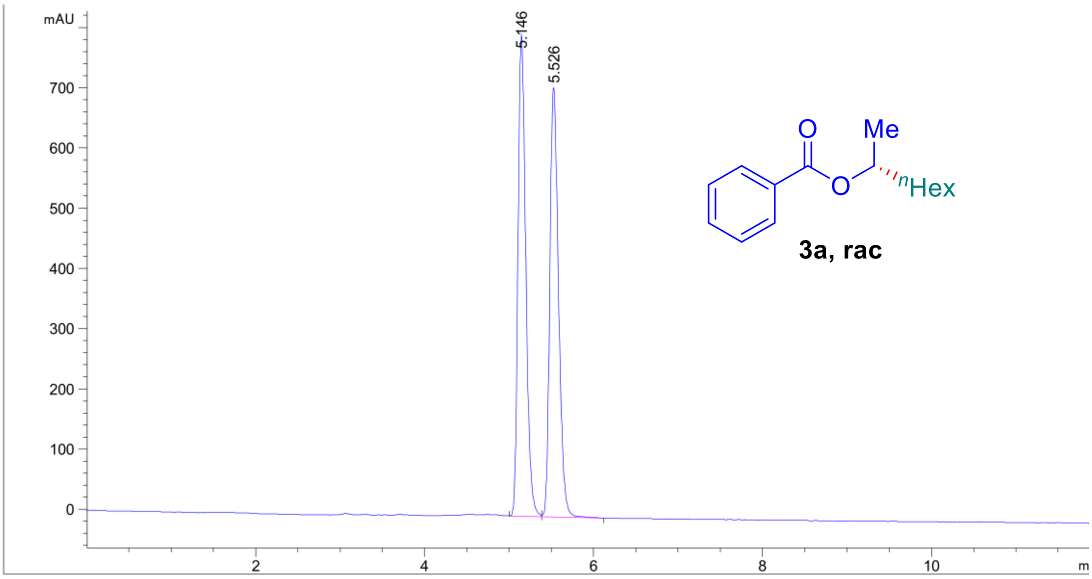

Peak #	RetTime [min]	Type	Width [min]	Area [mAU*s]	Height [mAU]	Area %
1	5.146	BV	0.0987	5116.66504	799.57074	50.1186
2	5.526	VB	0.1113	5092.44385	713.08191	49.8814

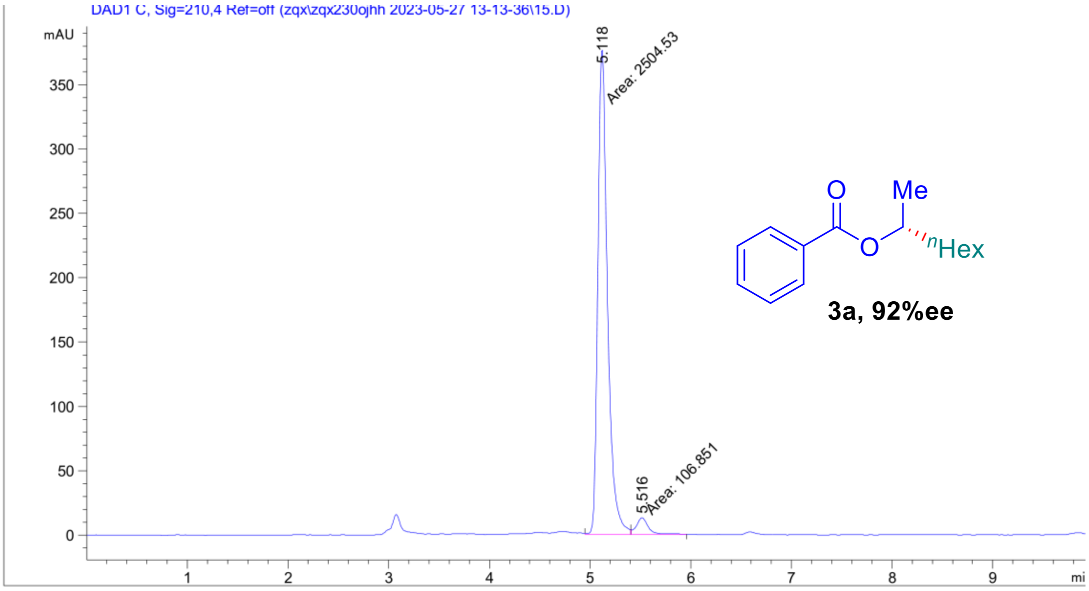

Peak #	RetTime [min]	Type	Width [min]	Area [mAU*s]	Height [mAU]	Area %
1	5.118	FM	0.1106	2504.52759	377.25928	95.9083
2	5.516	FM	0.1388	106.85081	12.83204	4.0917

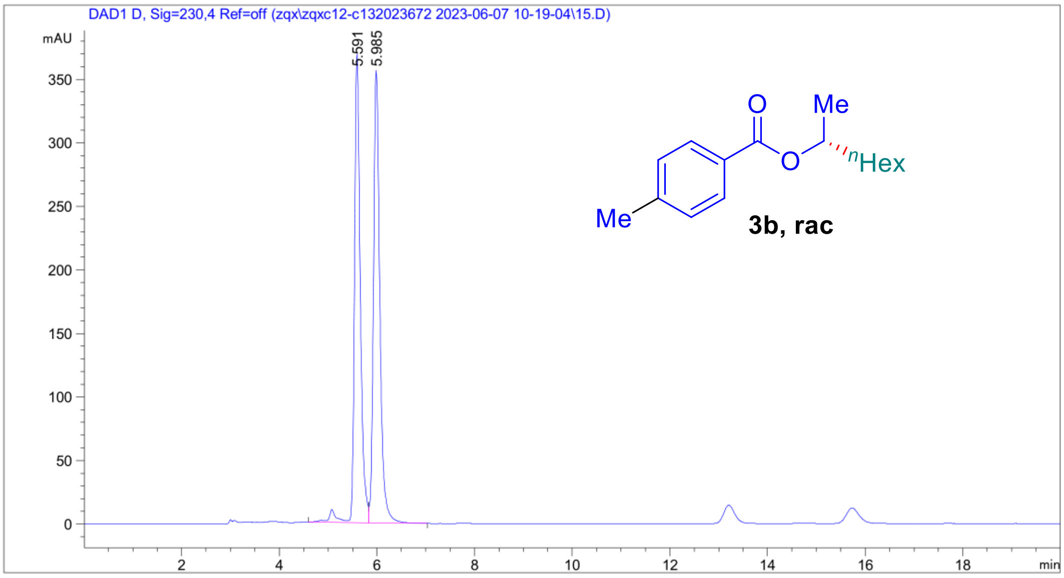

Peak #	RetTime [min]	Type	Width [min]	Area [mAU*s]	Height [mAU]	Area %
1	5.591	VV R	0.1258	3138.68408	368.80157	49.5308
2	5.985	VB	0.1348	3198.14307	356.18915	50.4692

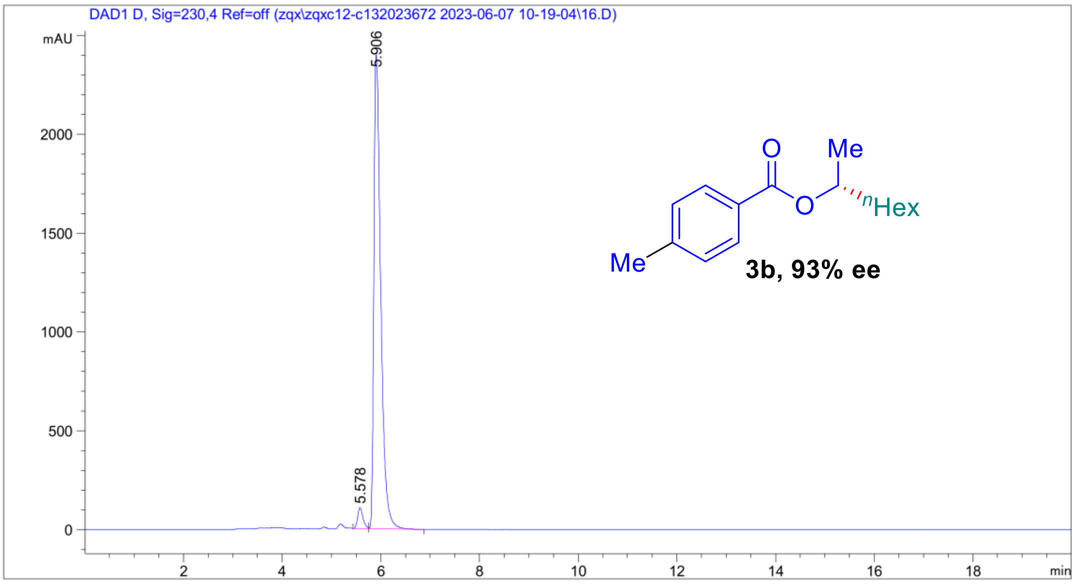

Peak #	RetTime [min]	Type	Width [min]	Area [mAU*s]	Height [mAU]	Area %
1	5.578	BV	0.1160	799.41541	106.02410	3.3304
2	5.906	VB	0.1468	2.32040e4	2400.66577	96.6696

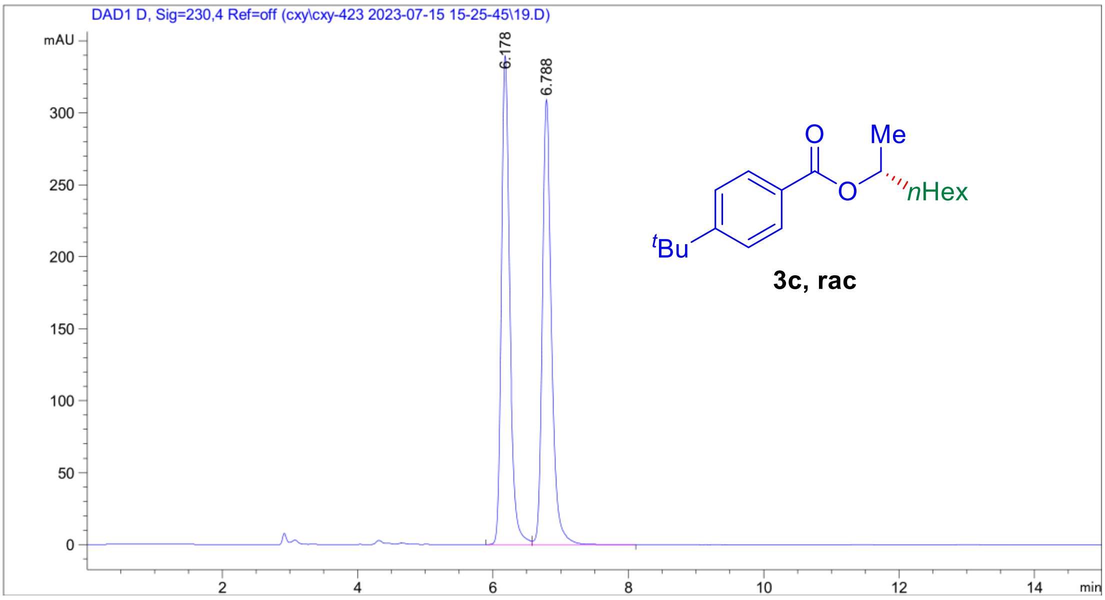

Peak #	RetTime [min]	Type	Width [min]	Area [mAU*s]	Height [mAU]	Area %
1	6.178	BV	0.1290	2884.19238	339.95779	49.5437
2	6.788	VB	0.1449	2937.31592	309.12170	50.4563

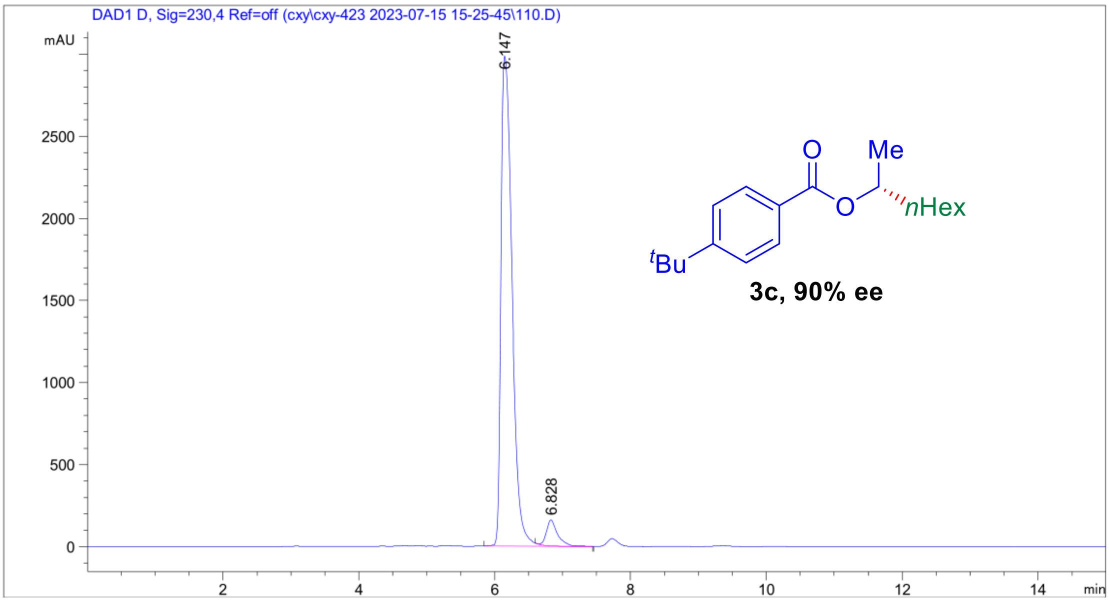

Peak #	RetTime [min]	Type	Width [min]	Area [mAU*s]	Height [mAU]	Area %
1	6.147	BV R	0.1770	3.35178e4	2985.09326	94.7634
2	6.828	VB E	0.1714	1852.19946	159.55119	5.2366

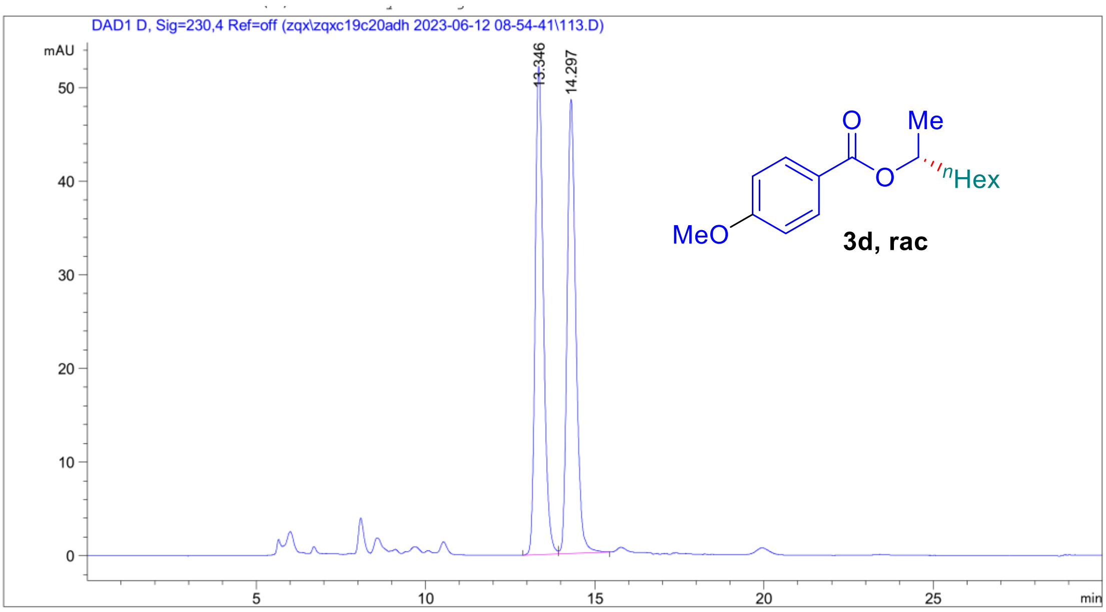

Peak #	RetTime [min]	Type	Width [min]	Area [mAU*s]	Height [mAU]	Area %
1	13.346	BV	0.2559	875.70258	52.15761	50.2846
2	14.297	VB	0.2743	865.79120	48.50151	49.7154

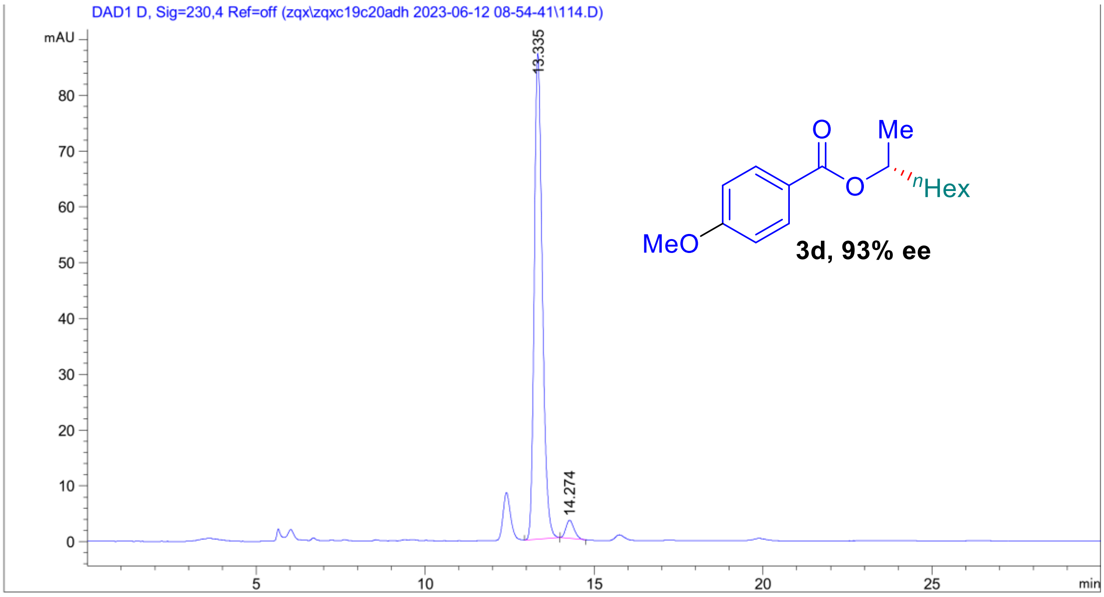

Peak #	RetTime [min]	Type	Width [min]	Area [mAU*s]	Height [mAU]	Area %
1	13.335	BB	0.2558	1445.82727	87.05665	96.3212
2	14.274	BB	0.2588	55.22060	3.27334	3.6788

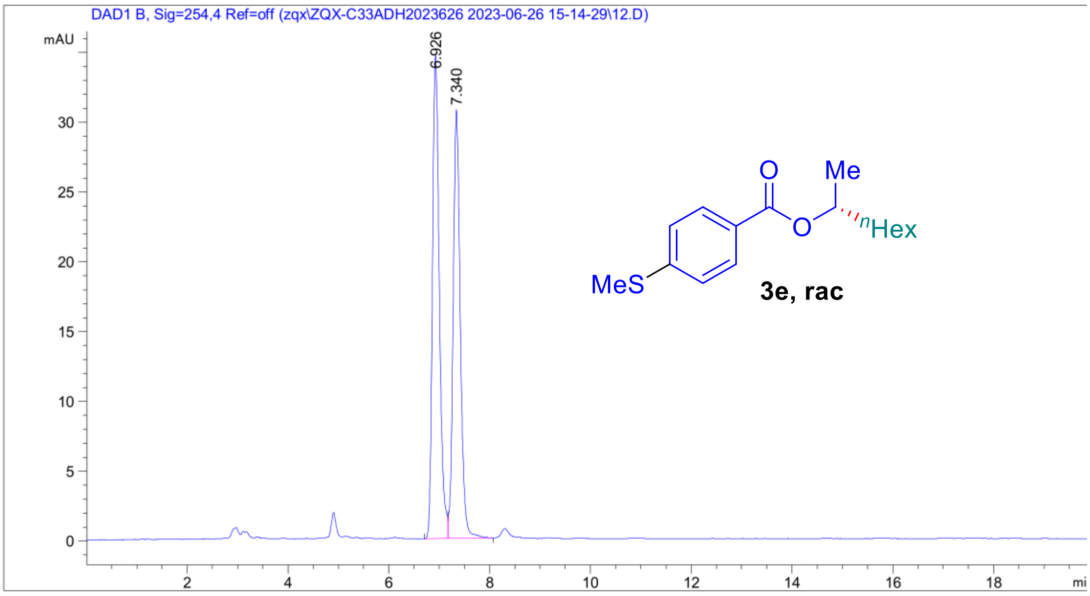

Peak #	RetTime [min]	Type	Width [min]	Area [mAU*s]	Height [mAU]	Area %
1	6.926	BV	0.1438	325.79269	34.62651	51.9716
2	7.340	VB	0.1483	301.07452	30.72608	48.0284

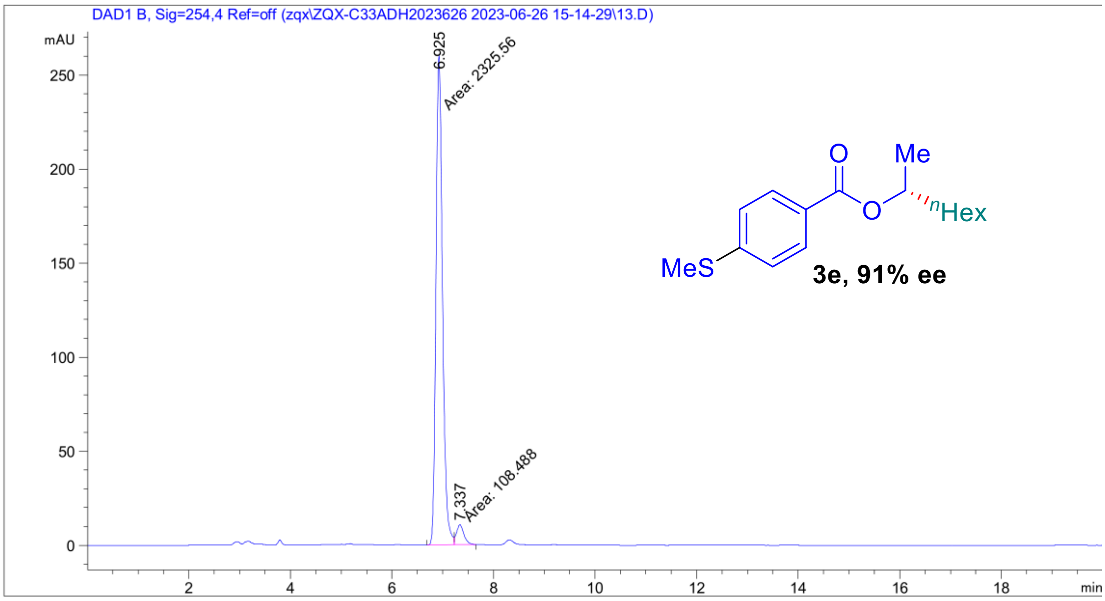

Peak #	RetTime [min]	Type	Width [min]	Area [mAU*s]	Height [mAU]	Area %
1	6.925	MF	0.1490	2325.56323	260.14688	95.5429
2	7.337	FM	0.1697	108.48837	10.65329	4.4571

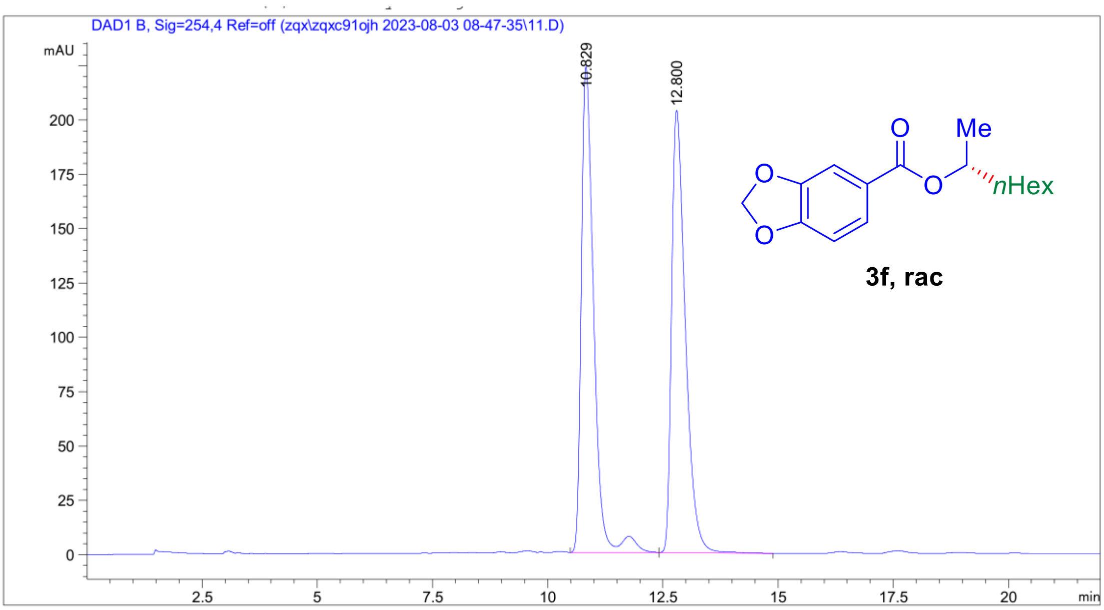

Peak #	RetTime [min]	Type	Width [min]	Area [mAU*s]	Height [mAU]	Area %
1	10.829	BV R	0.2748	4222.78711	223.53729	50.1265
2	12.800	BB	0.3148	4201.47900	203.53604	49.8735

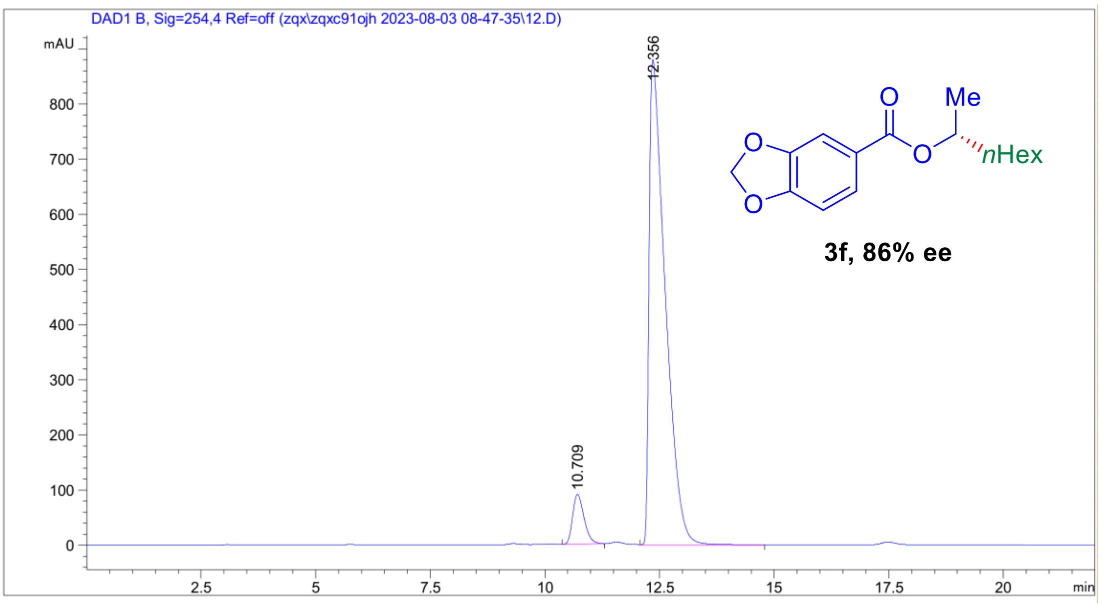

Peak #	RetTime [min]	Type	Width [min]	Area [mAU*s]	Height [mAU]	Area %
1	10.709	BB	0.2674	1564.06519	90.59634	6.7667
2	12.356	BB	0.3617	2.15500e4	879.99512	93.2333

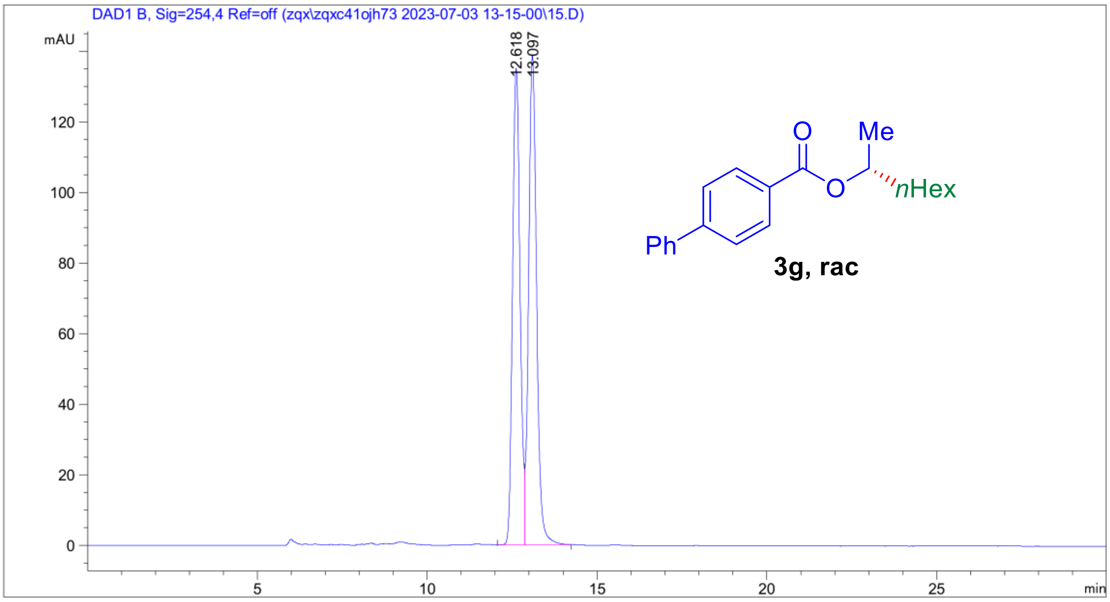

Peak #	RetTime [min]	Type	Width [min]	Area [mAU*s]	Height [mAU]	Area %
1	12.618	BV	0.2372	2052.85156	135.12733	47.4780
2	13.097	VB	0.2510	2270.94019	138.74142	52.5220

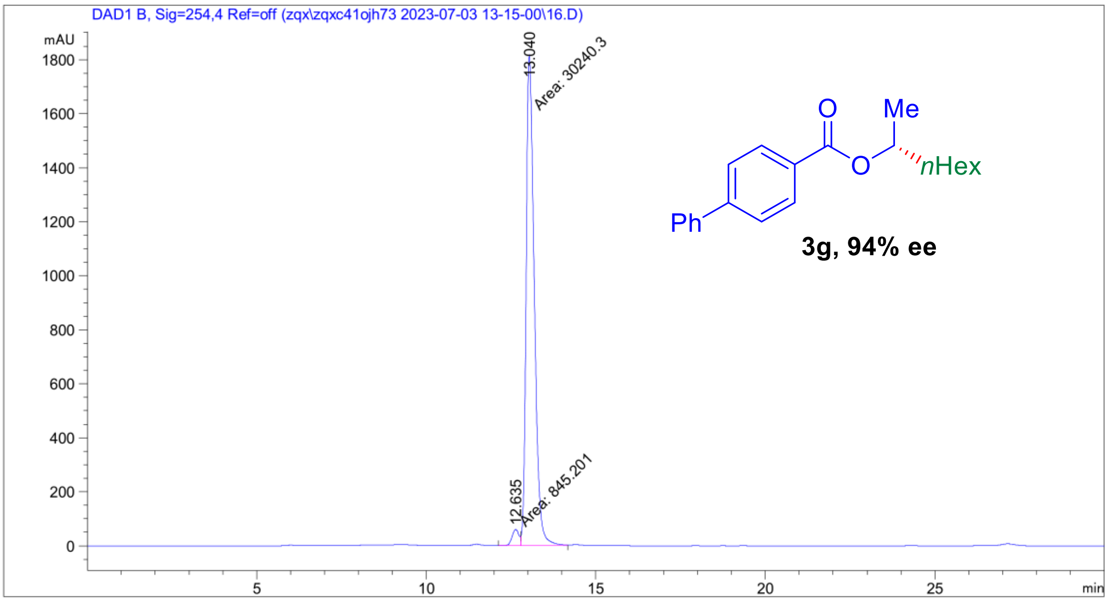

Peak #	RetTime [min]	Type	Width [min]	Area [mAU*s]	Height [mAU]	Area %
1	12.635	MF	0.2369	845.20093	59.45129	2.7190
2	13.040	FM	0.2779	3.02403e4	1813.40417	97.2810

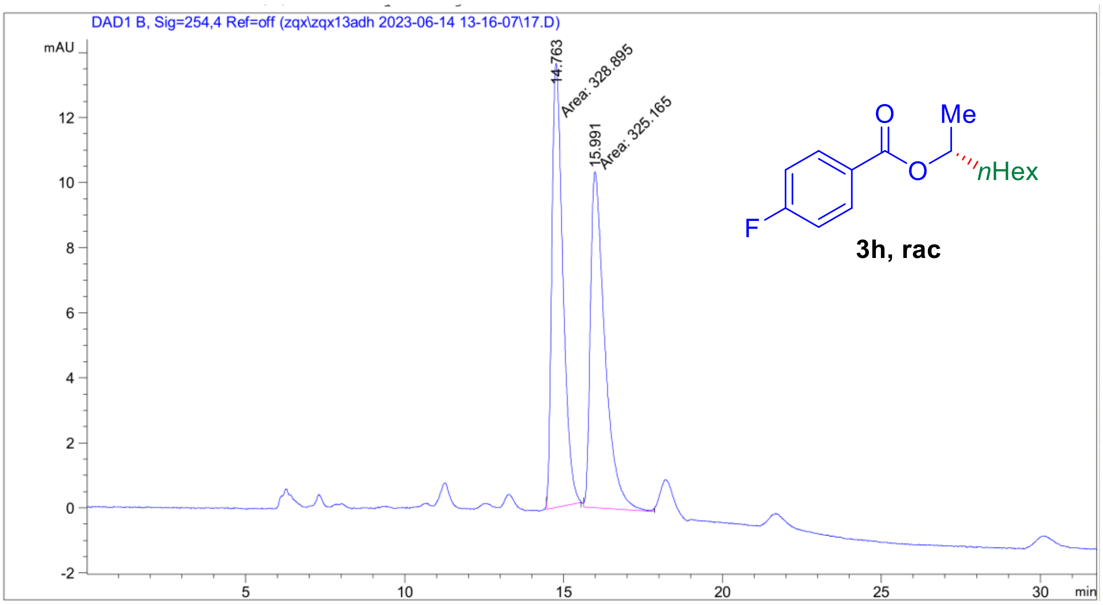

Peak #	RetTime [min]	Type	Width [min]	Area [mAU*s]	Height [mAU]	Area %
1	14.763	MM	0.4017	328.89532	13.64437	50.2852
2	15.991	MM	0.5256	325.16470	10.31047	49.7148

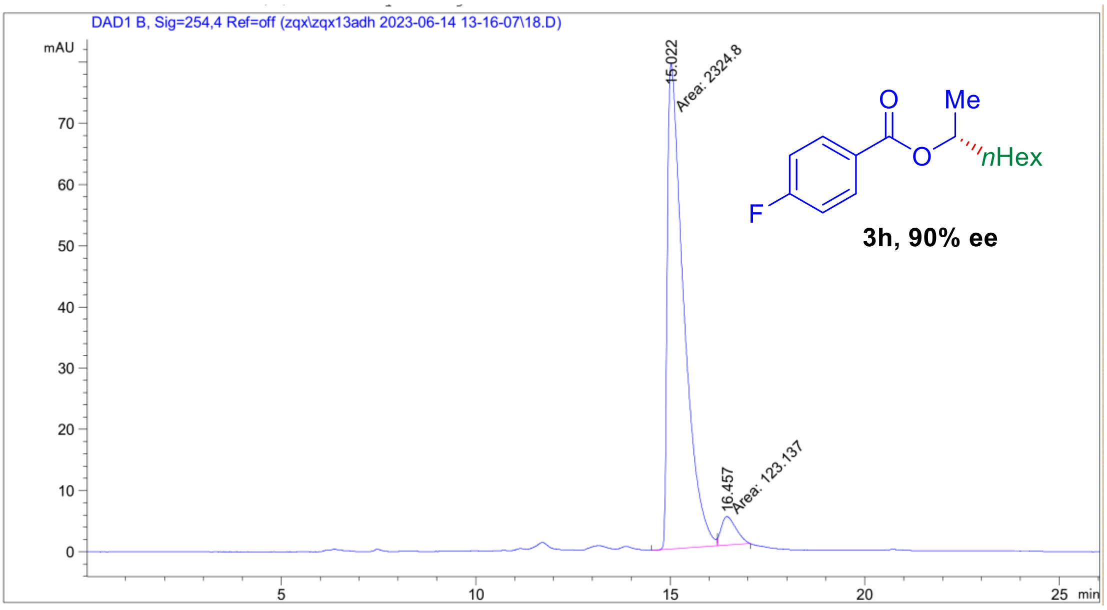

Peak #	RetTime [min]	Type	Width [min]	Area [mAU*s]	Height [mAU]	Area %
1	15.022	MF	0.4887	2324.80273	79.28197	94.9698
2	16.457	FM	0.4420	123.13657	4.64265	5.0302

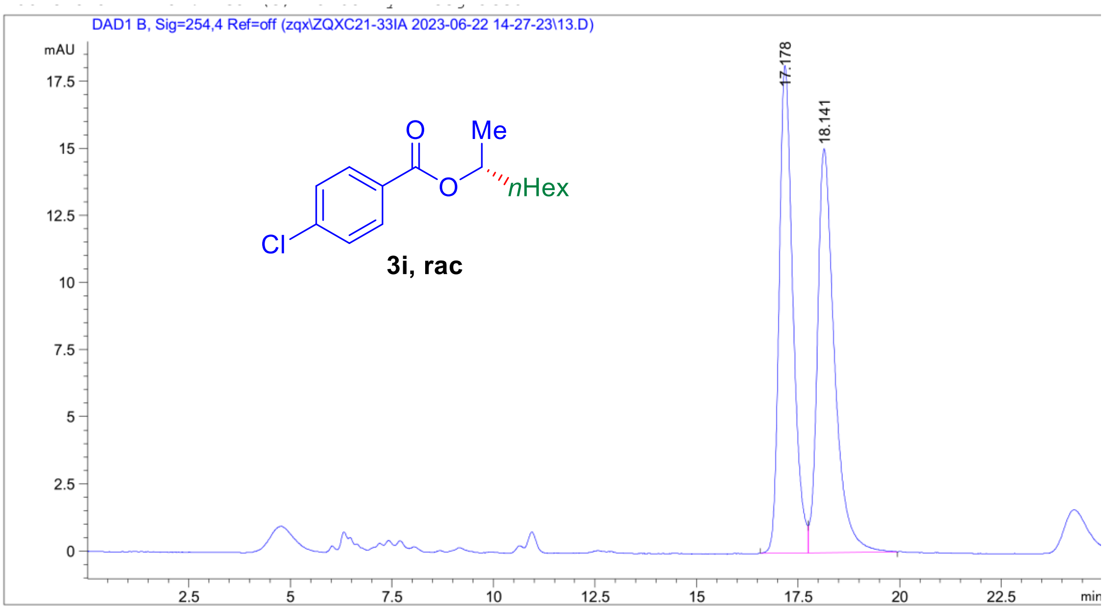

Peak #	RetTime [min]	Type	Width [min]	Area [mAU*s]	Height [mAU]	Area %
1	17.178	BV	0.3598	426.35196	18.16512	49.3427
2	18.141	VB	0.4312	437.71048	15.05621	50.6573

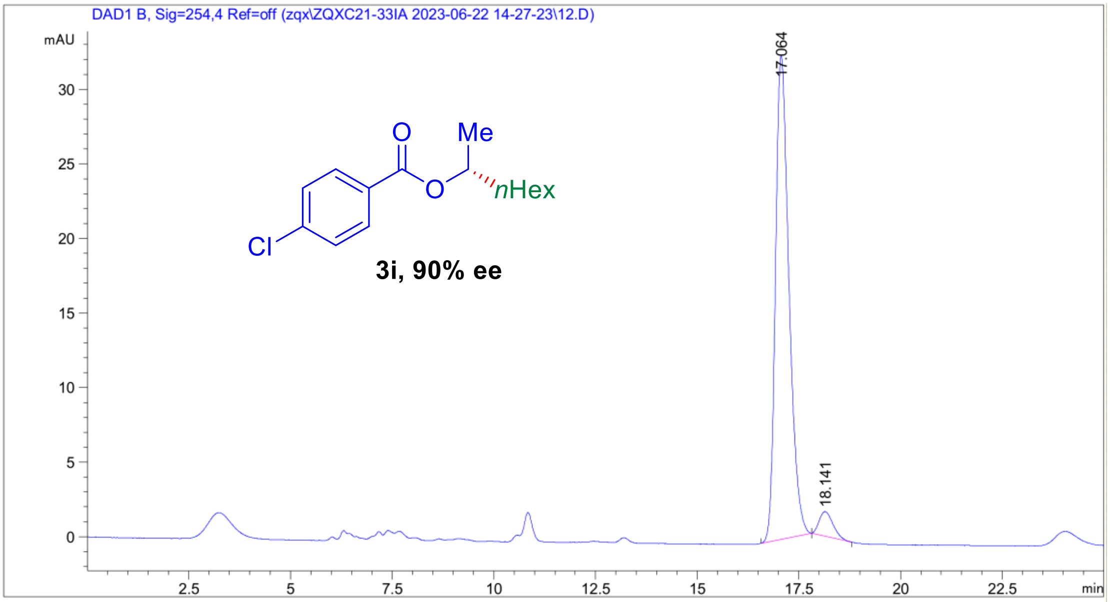

Peak #	RetTime [min]	Type	Width [min]	Area [mAU*s]	Height [mAU]	Area %
1	17.064	BB	0.3526	746.72229	32.43024	95.0067
2	18.141	BB	0.3647	39.24599	1.65447	4.9933

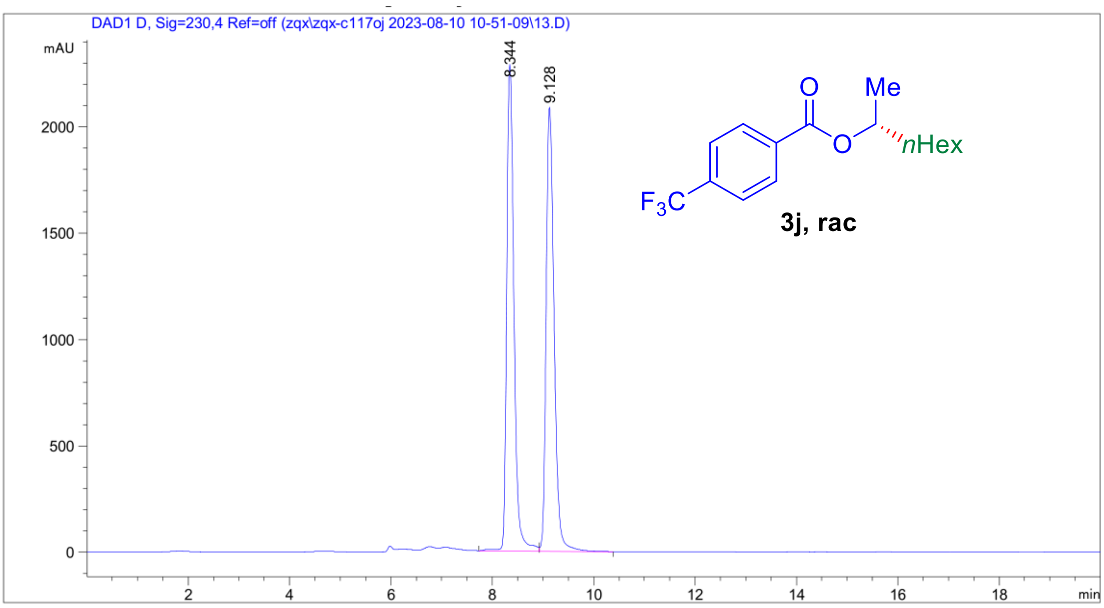

Peak #	RetTime [min]	Type	Width [min]	Area [mAU*s]	Height [mAU]	Area %
1	8.344	VV R	0.1535	2.27670e4	2291.52026	49.9229
2	9.128	VB	0.1696	2.28373e4	2087.73950	50.0771

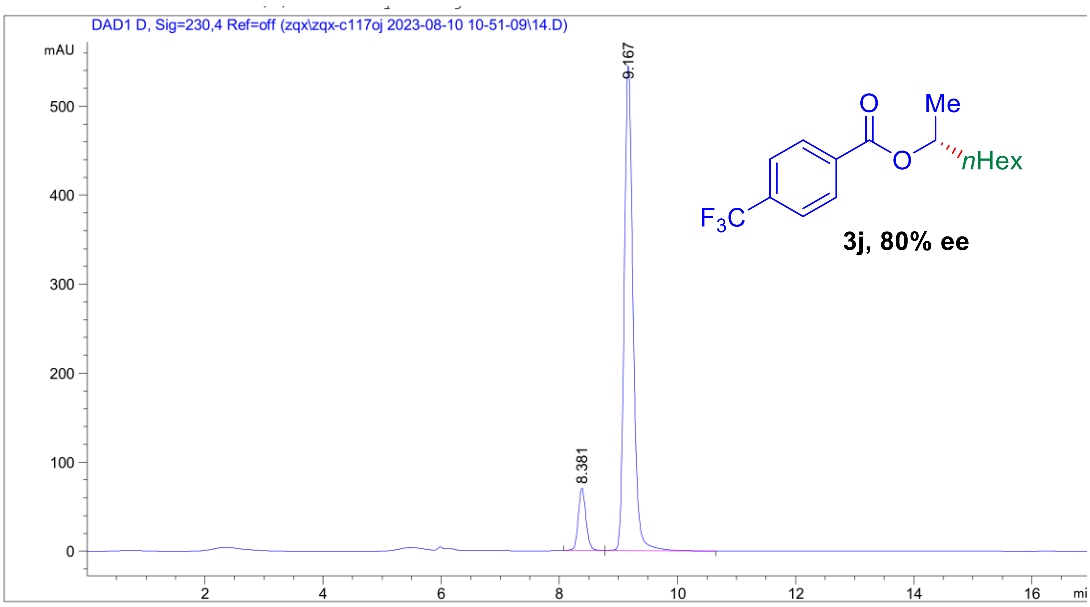

Peak #	RetTime [min]	Type	Width [min]	Area [mAU*s]	Height [mAU]	Area %
1	8.381	BB	0.1377	634.09326	69.99474	10.0901
2	9.167	BB	0.1610	5650.20996	544.85297	89.9099

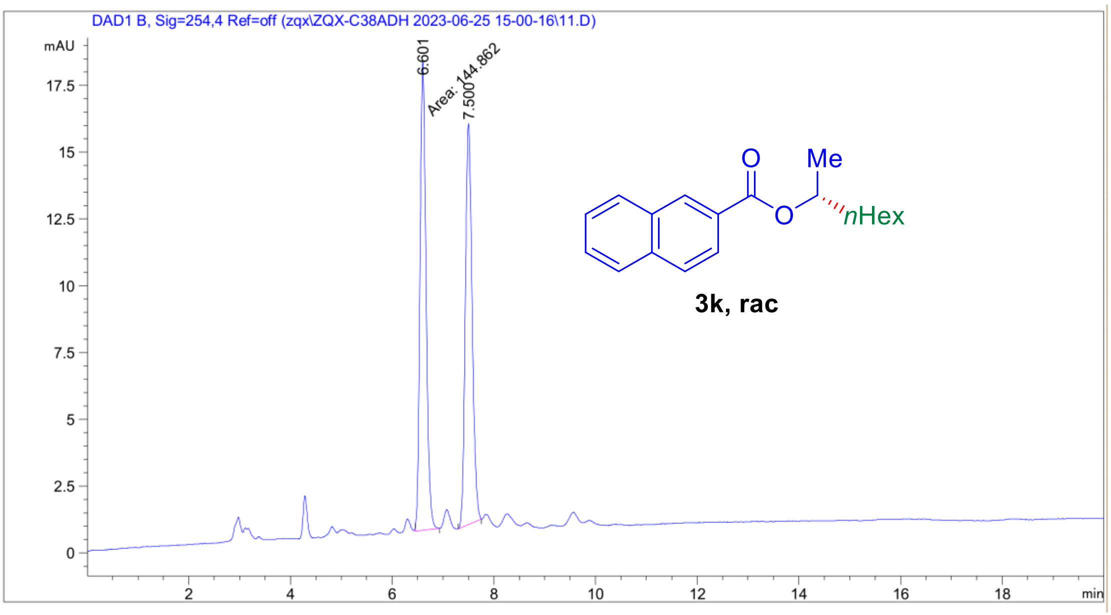

Peak #	RetTime [min]	Type	Width [min]	Area [mAU*s]	Height [mAU]	Area %
1	6.601	FM	0.1374	144.86221	17.56613	50.7737
2	7.500	BB	0.1432	140.44739	14.99879	49.2263

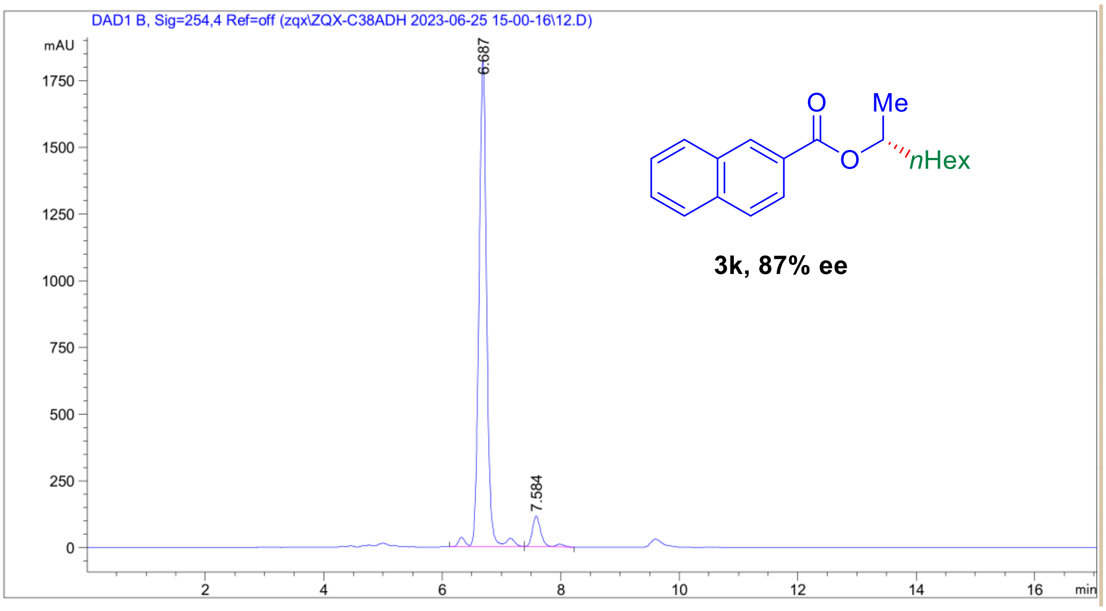

Peak #	RetTime [min]	Type	Width [min]	Area [mAU*s]	Height [mAU]	Area %
1	6.687	VV R	0.1342	1.66045e4	1820.26587	93.2454
2	7.584	BV R	0.1483	1202.82349	114.57615	6.7546

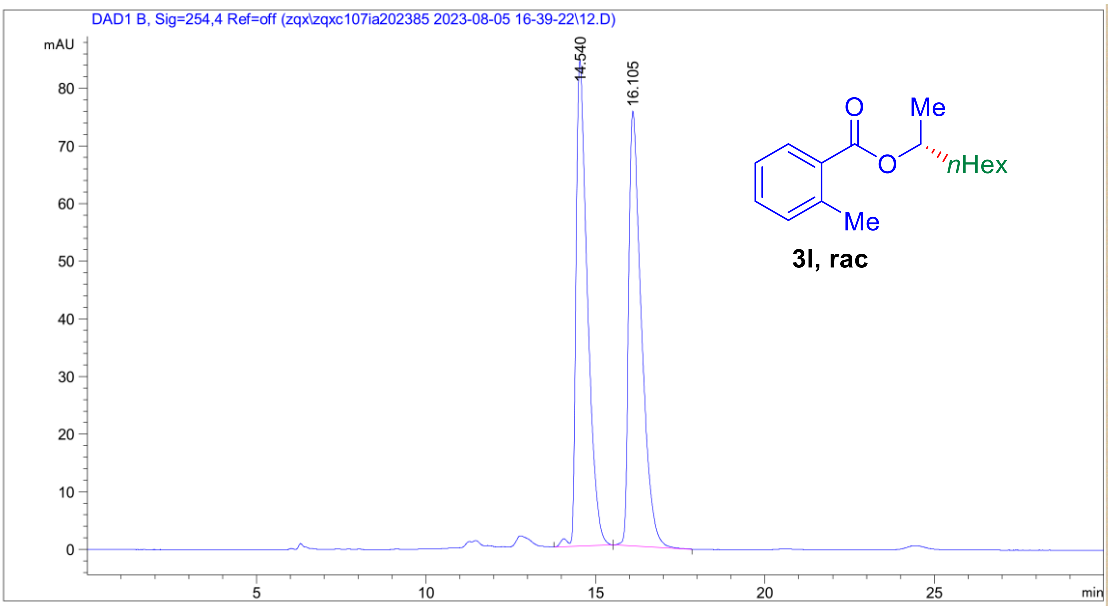

Peak #	RetTime [min]	Type	Width [min]	Area [mAU*s]	Height [mAU]	Area %
1	14.540	VB R	0.3486	1974.96240	84.27333	49.9204
2	16.105	BB	0.3923	1981.25720	75.47320	50.0796

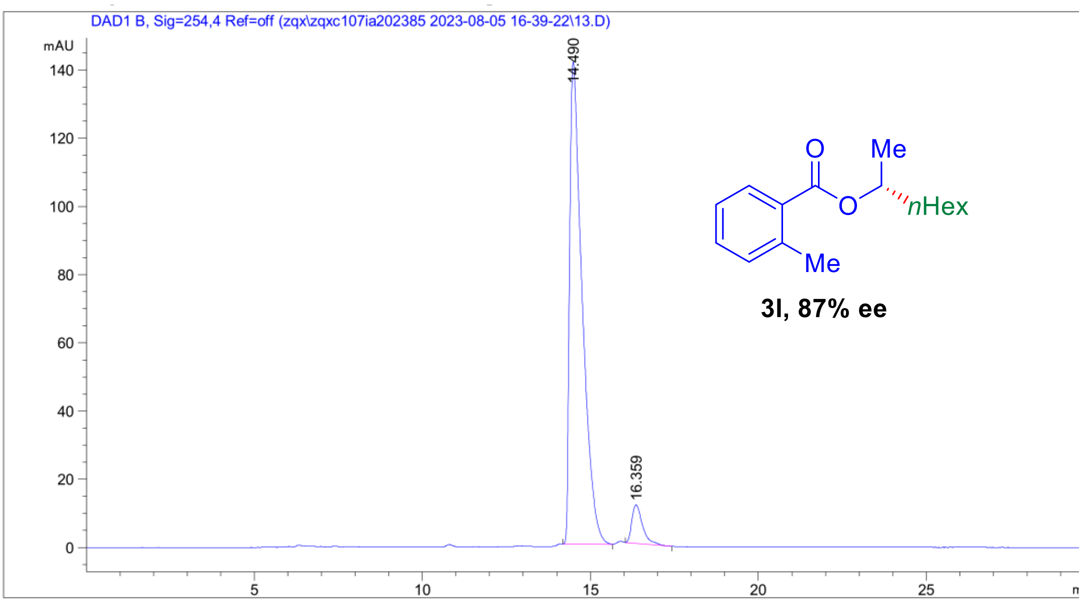

Peak #	RetTime [min]	Type	Width [min]	Area [mAU*s]	Height [mAU]	Area %
1	14.490	BB	0.3819	3658.69897	141.39716	93.6295
2	16.359	BB	0.3361	248.93427	11.25164	6.3705

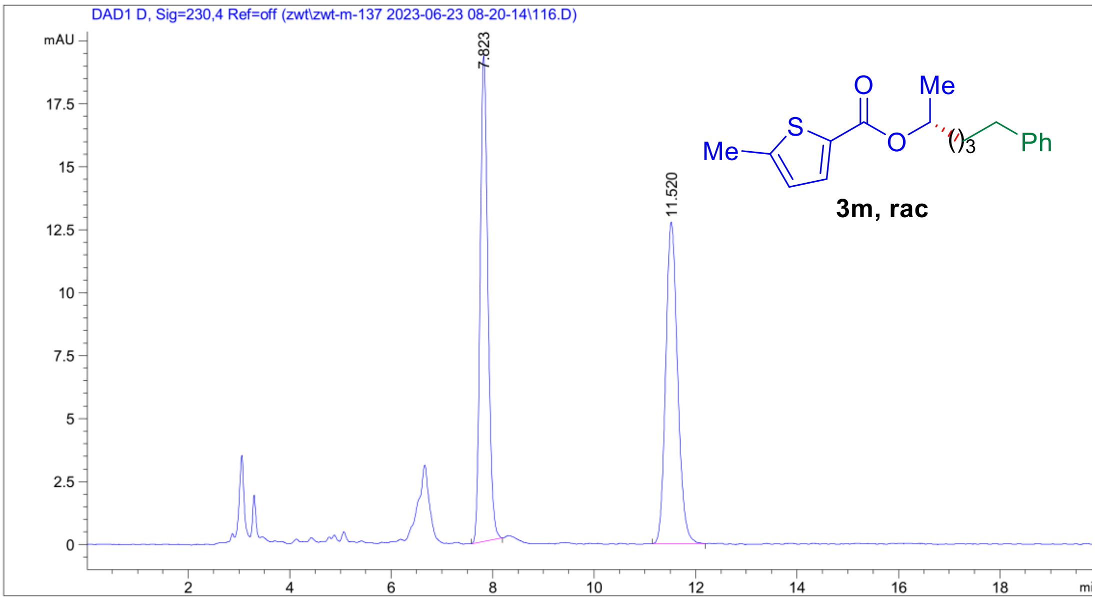

Peak #	RetTime [min]	Type	Width [min]	Area [mAU*s]	Height [mAU]	Area %
1	7.823	BB	0.1642	205.48811	19.29844	49.6584
2	11.520	BB	0.2522	208.31561	12.77861	50.3416

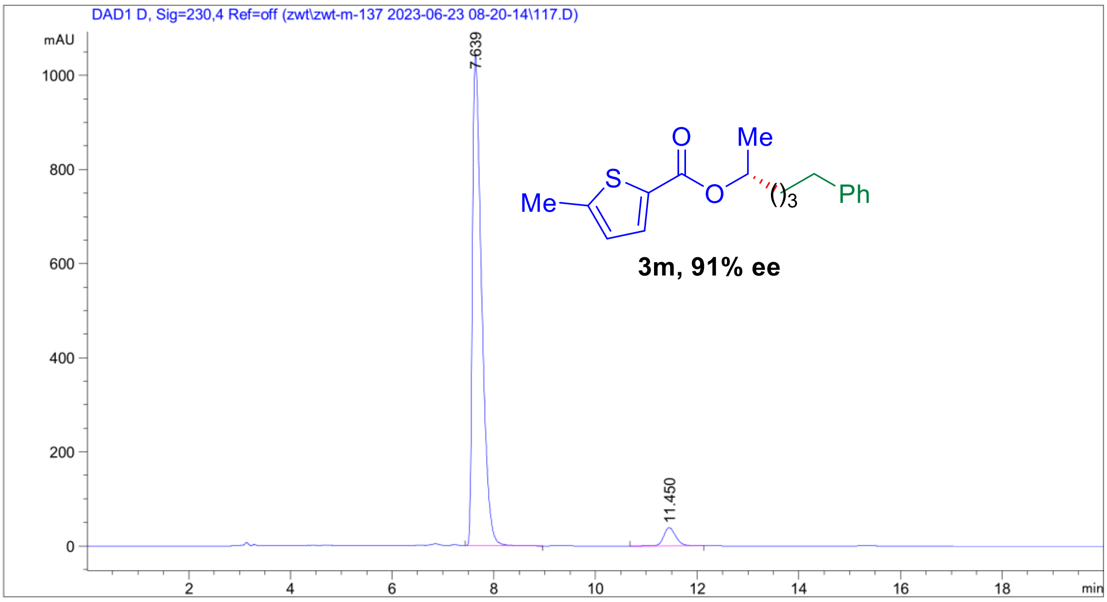

Peak #	RetTime [min]	Type	Width [min]	Area [mAU*s]	Height [mAU]	Area %
1	7.639	BB	0.1899	1.30218e4	1041.40710	95.2883
2	11.450	BB	0.2556	643.88672	38.80512	4.7117

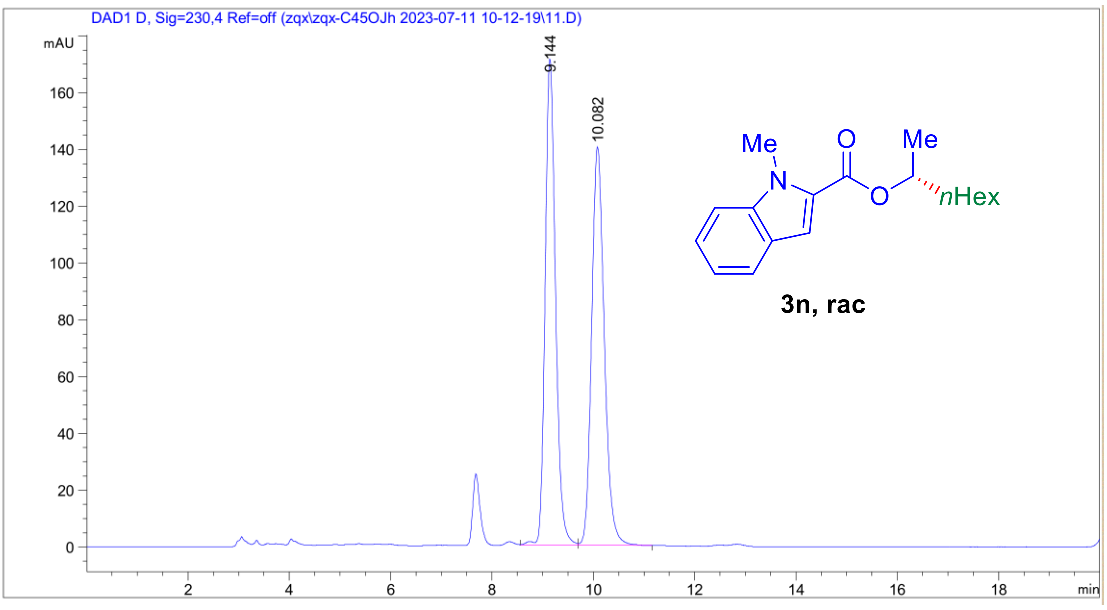

Peak #	RetTime [min]	Type	Width [min]	Area [mAU*s]	Height [mAU]	Area %
1	9.144	VV R	0.2171	2432.66821	171.12933	49.9148
2	10.082	VB	0.2670	2440.97656	140.26237	50.0852

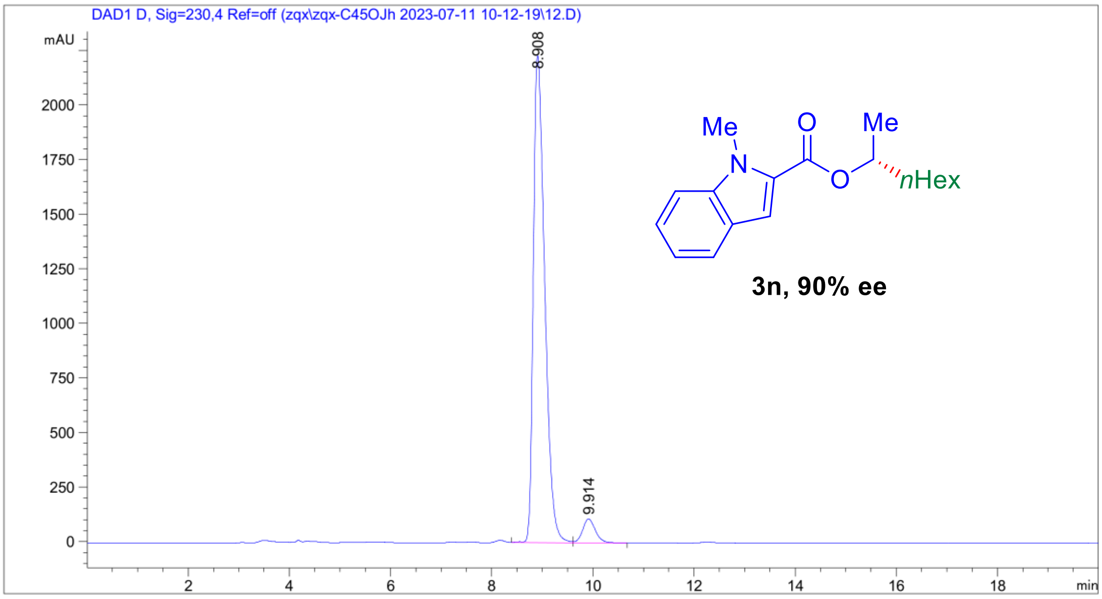

Peak #	RetTime [min]	Type	Width [min]	Area [mAU*s]	Height [mAU]	Area %
1	8.908	VV R	0.2425	3.49122e4	2230.19775	94.8040
2	9.914	VB	0.2700	1913.47559	109.44203	5.1960

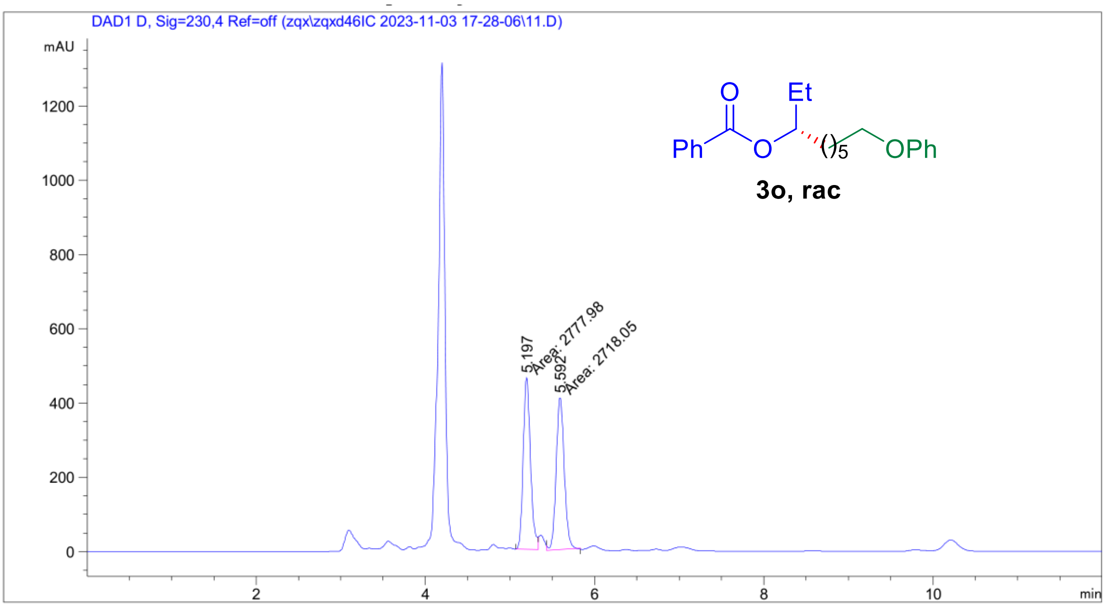

Peak #	RetTime [min]	Type	Width [min]	Area [mAU*s]	Height [mAU]	Area %
1	5.197	MF	0.0999	2777.98169	463.62170	50.5452
2	5.592	MM	0.1107	2718.04810	409.33444	49.4548

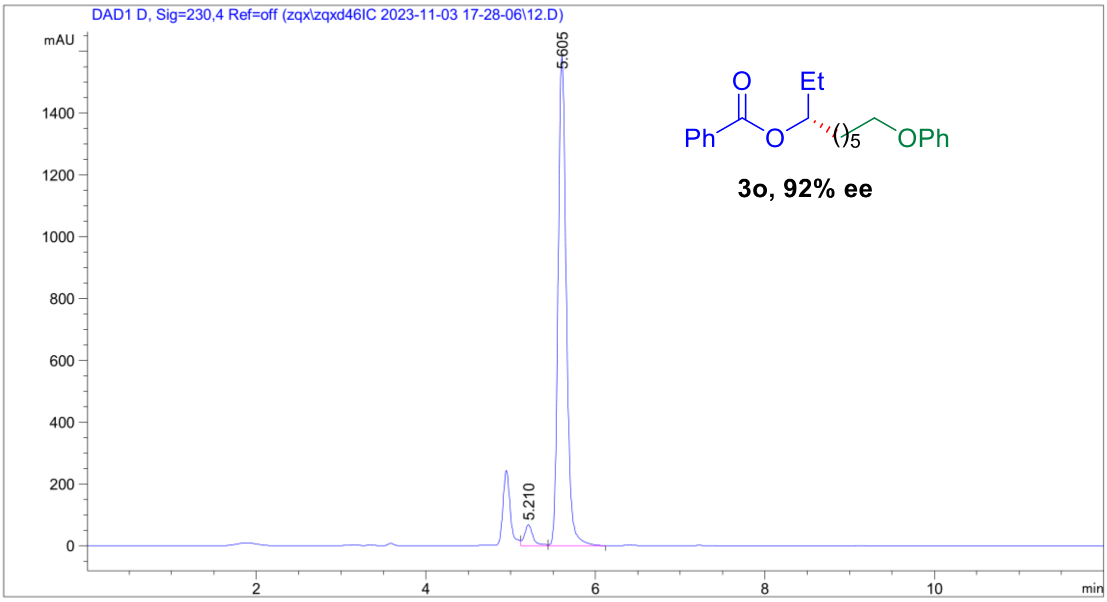

Peak #	RetTime [min]	Type	Width [min]	Area [mAU*s]	Height [mAU]	Area %
1	5.210	VV	0.1021	460.82599	67.15859	4.1249
2	5.605	VB	0.1049	1.07111e4	1583.87903	95.8751

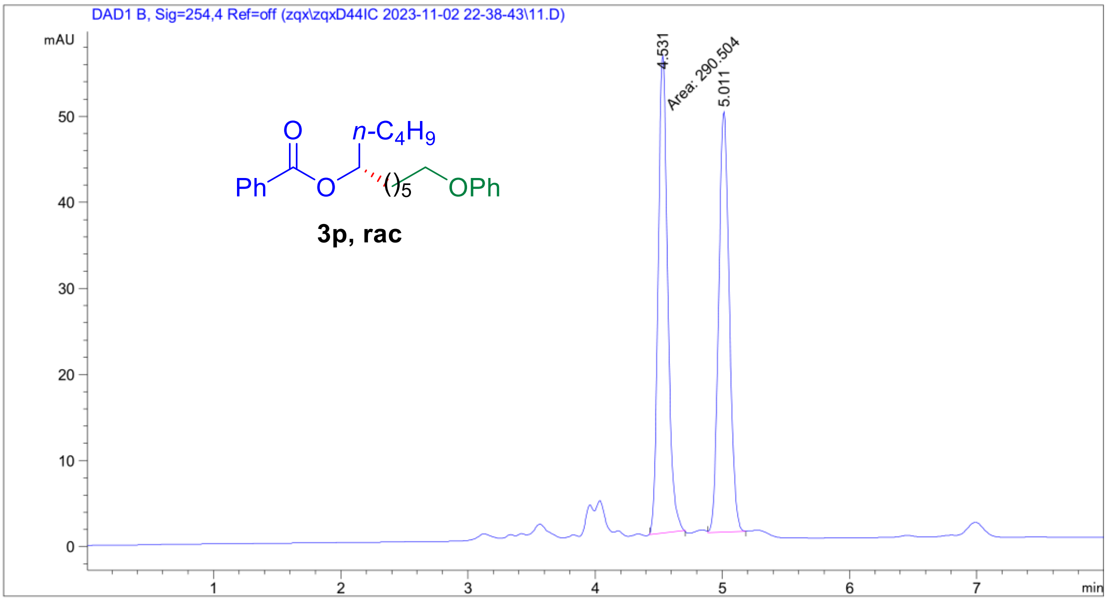

Peak #	RetTime [min]	Type	Width [min]	Area [mAU*s]	Height [mAU]	Area %
1	4.531	MM	0.0871	290.50424	55.61526	50.5737
2	5.011	VB	0.0898	283.91394	48.88302	49.4263

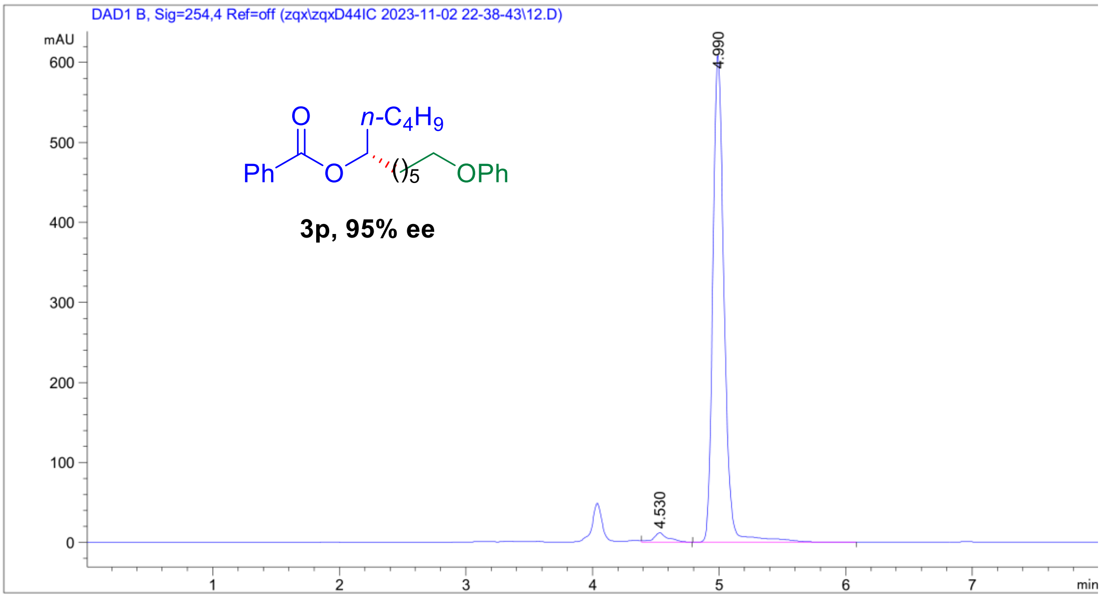

Peak #	RetTime [min]	Type	Width [min]	Area [mAU*s]	Height [mAU]	Area %
1	4.530	VB	0.1101	92.03794	11.66411	2.3847
2	4.990	BB	0.0961	3767.47754	609.60718	97.6153

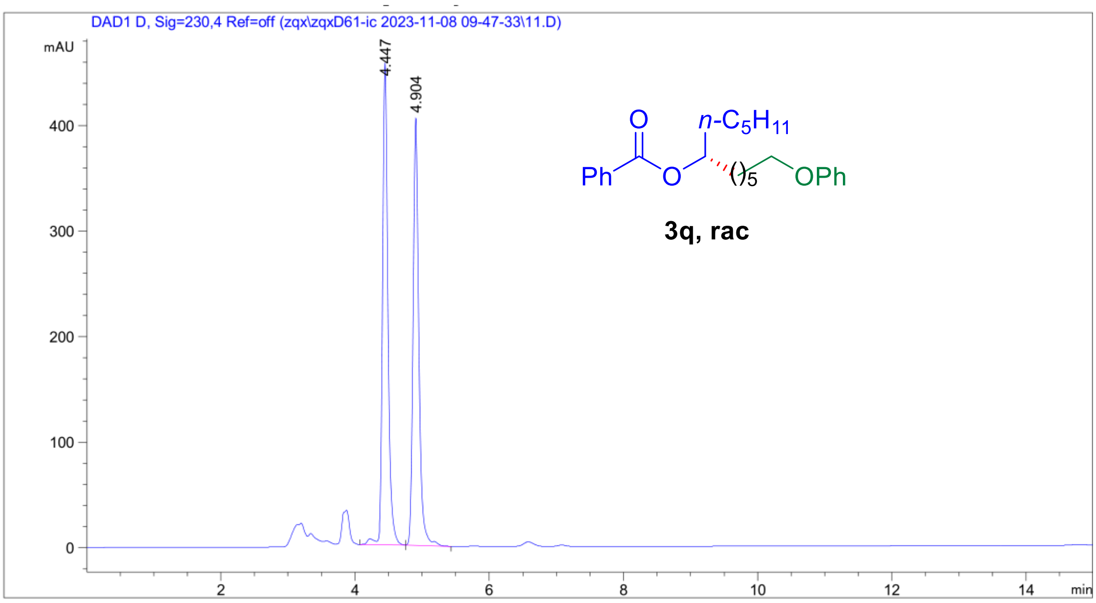

Peak #	RetTime [min]	Type	Width [min]	Area [mAU*s]	Height [mAU]	Area %
1	4.447	VB R	0.0815	2529.00342	456.43686	50.2348
2	4.904	BV R	0.0936	2505.36084	405.64355	49.7652

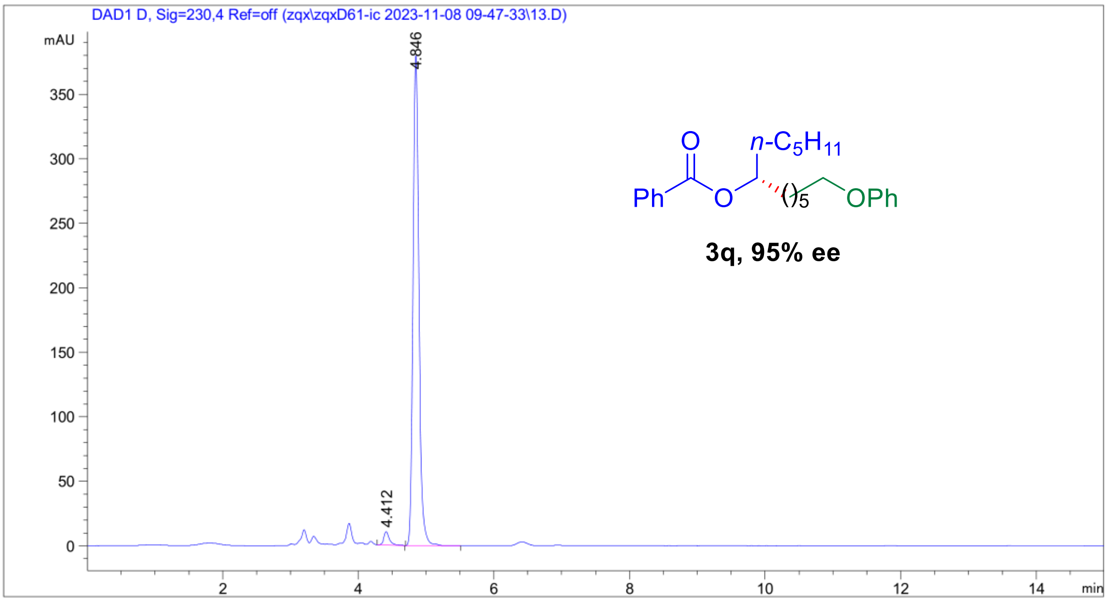

Peak #	RetTime [min]	Type	Width [min]	Area [mAU*s]	Height [mAU]	Area %
1	4.412	VB	0.0883	60.43725	10.32506	2.5686
2	4.846	BB	0.0923	2292.49487	380.70999	97.4314

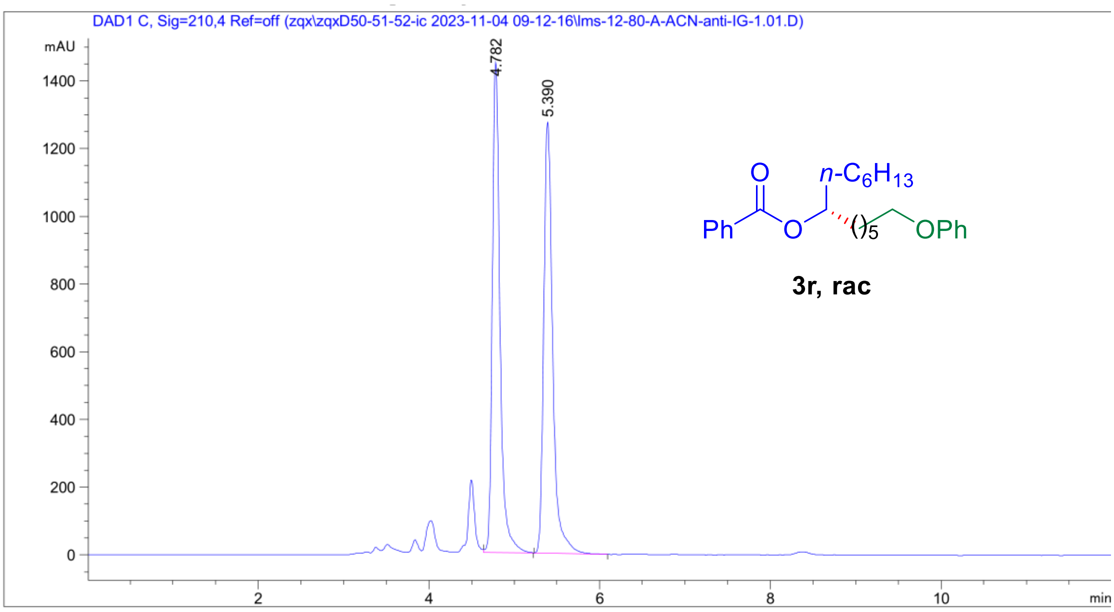

Peak #	RetTime [min]	Type	Width [min]	Area [mAU*s]	Height [mAU]	Area %
1	4.782	VB	0.0948	9037.05469	1448.47668	49.3834
2	5.390	BB	0.1107	9262.72559	1275.11060	50.6166

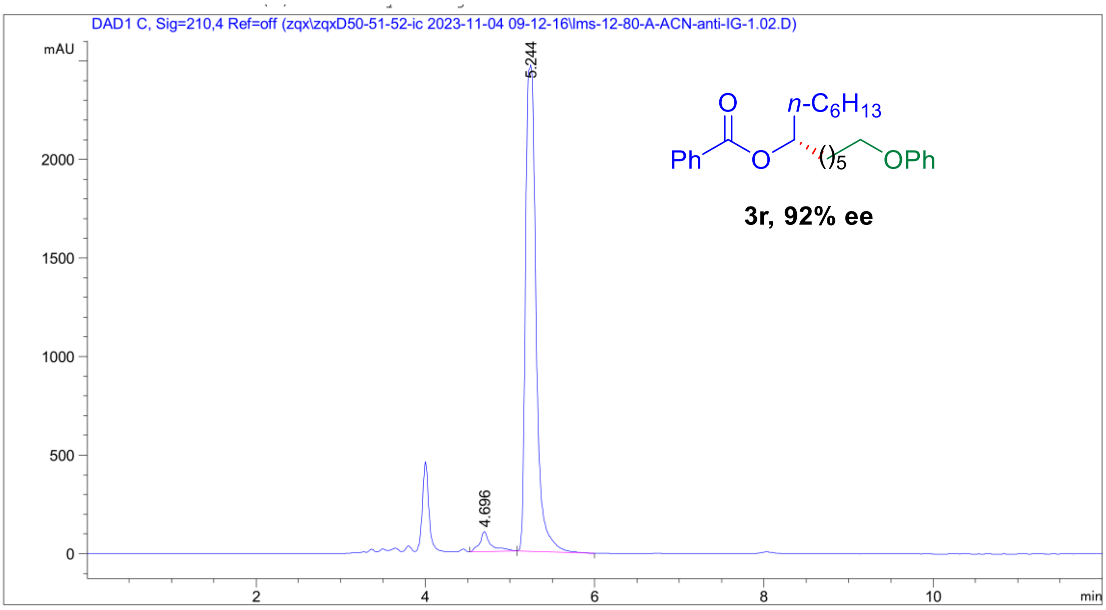

Peak #	RetTime [min]	Type	Width [min]	Area [mAU*s]	Height [mAU]	Area %
1	4.696	BV R	0.1211	928.86542	101.65501	4.1638
2	5.244	BB	0.1371	2.13792e4	2466.99414	95.8362

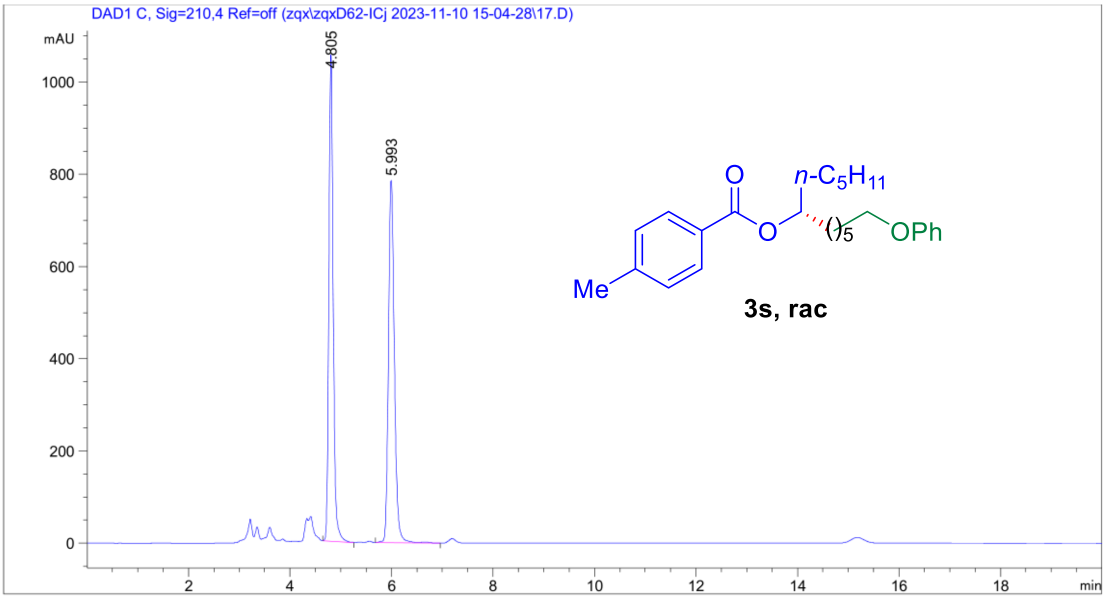

Peak #	RetTime [min]	Type	Width [min]	Area [mAU*s]	Height [mAU]	Area %
1	4.805	BB	0.0912	6271.57275	1057.89258	49.6010
2	5.993	BV R	0.1242	6372.46582	786.22681	50.3990

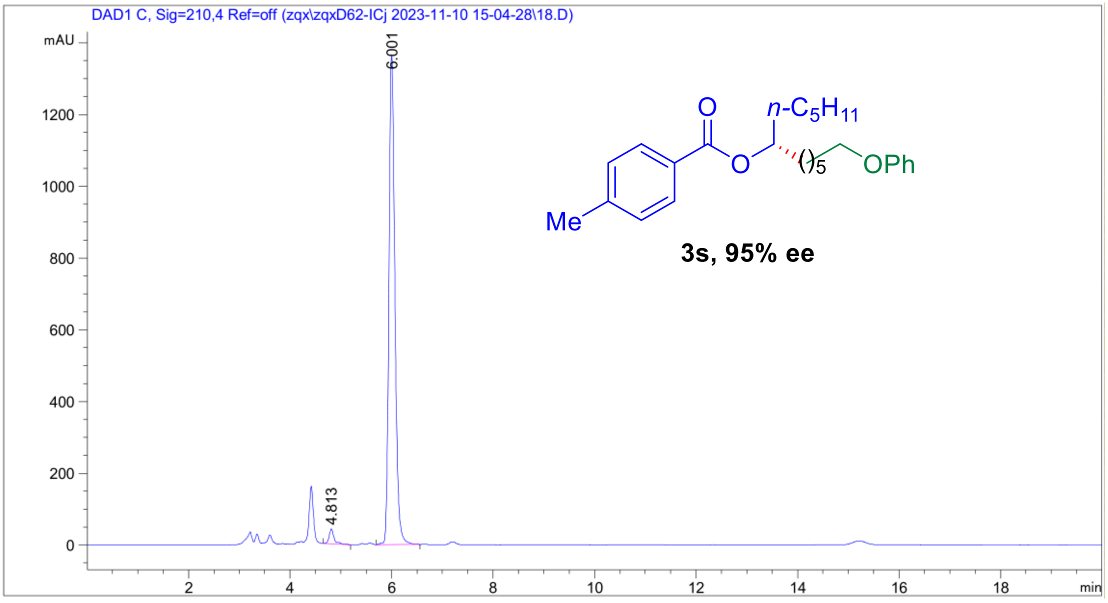

Peak #	RetTime [min]	Type	Width [min]	Area [mAU*s]	Height [mAU]	Area %
1	4.813	BV R	0.1007	281.31387	41.67985	2.4743
2	6.001	VB	0.1250	1.10881e4	1362.15576	97.5257

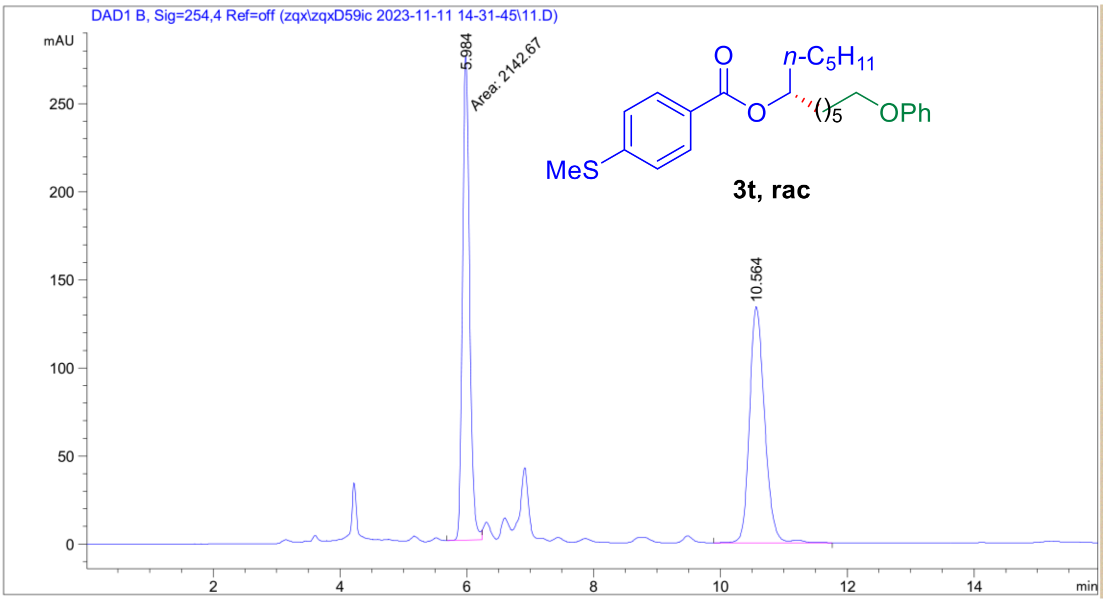

Peak #	RetTime [min]	Type	Width [min]	Area [mAU*s]	Height [mAU]	Area %
1	5.984	MF	0.1299	2142.66528	274.94540	49.0745
2	10.564	BV R	0.2536	2223.48511	134.02350	50.9255

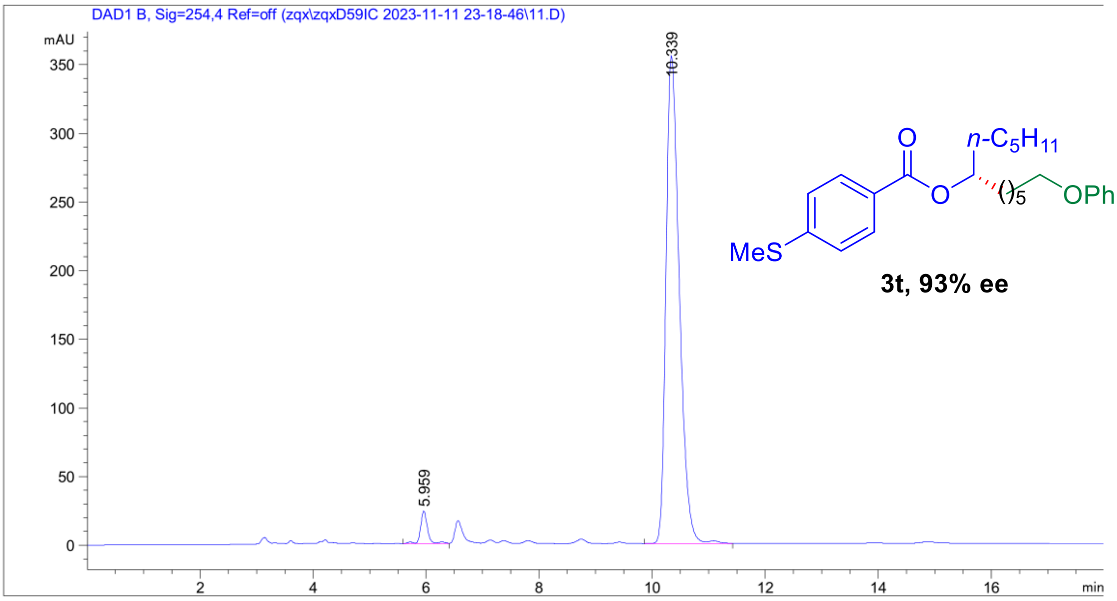

Peak #	RetTime [min]	Type	Width [min]	Area [mAU*s]	Height [mAU]	Area %
1	5.959	VV R	0.1255	201.01820	23.59881	3.3448
2	10.339	BV R	0.2520	5808.79541	355.16196	96.6552

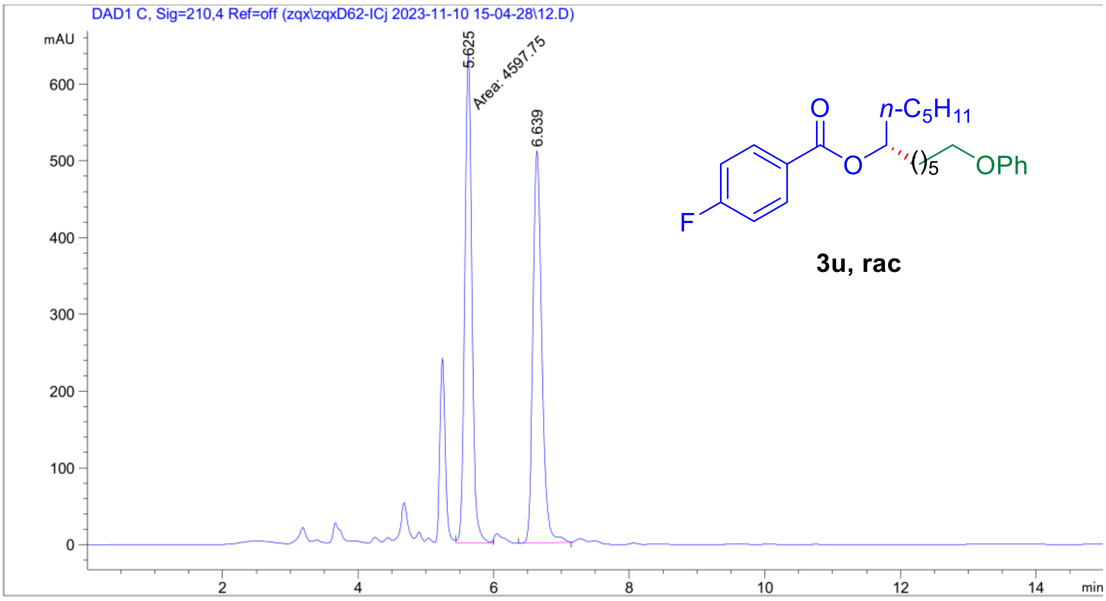

Peak #	RetTime [min]	Type	Width [min]	Area [mAU*s]	Height [mAU]	Area %
1	5.625	MF	0.1205	4597.74658	635.88959	49.9464
2	6.639	BV R	0.1389	4607.62012	510.54120	50.0536

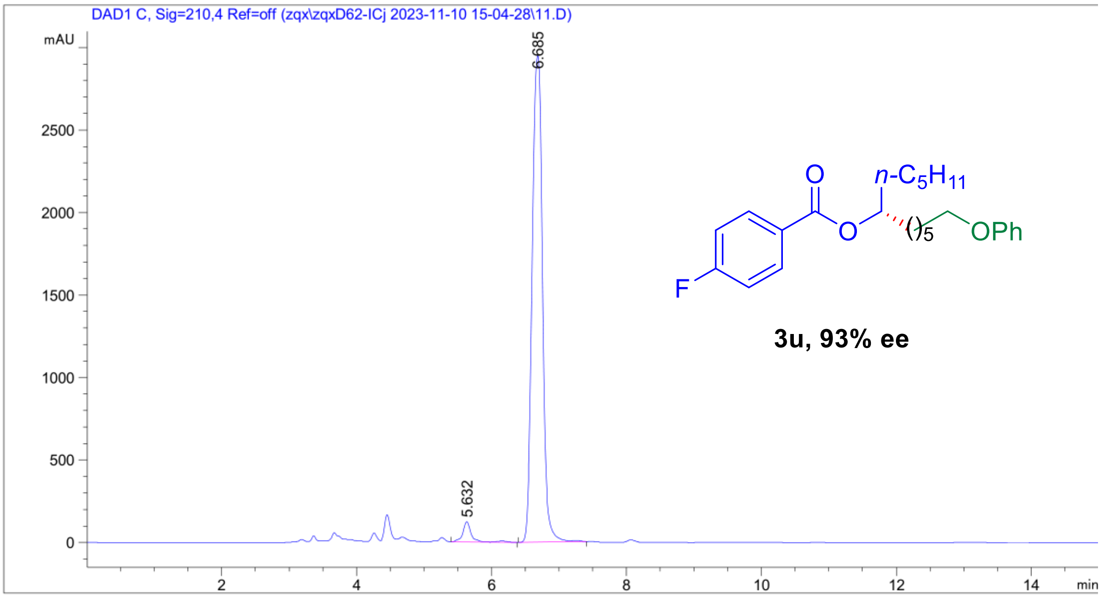

Peak #	RetTime [min]	Type	Width [min]	Area [mAU*s]	Height [mAU]	Area %
1	5.632	VV R	0.1226	1101.62134	123.78143	3.4386
2	6.685	BV R	0.1662	3.09350e4	2948.88135	96.5614

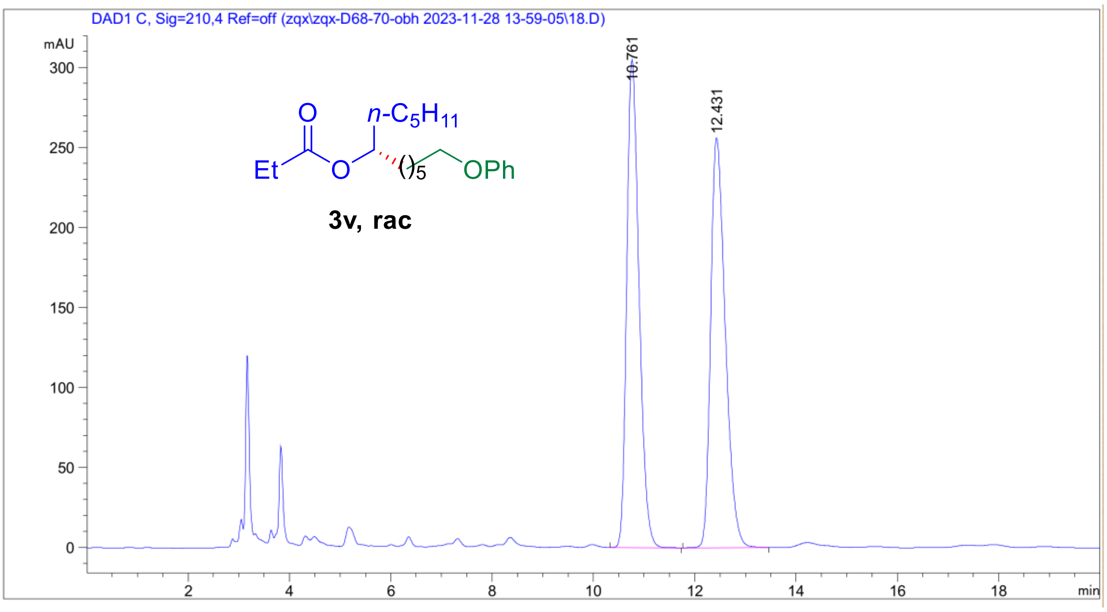

Peak #	RetTime [min]	Type	Width [min]	Area [mAU*s]	Height [mAU]	Area %
1	10.761	BB	0.2605	5135.20215	304.96240	49.8998
2	12.431	BB	0.3087	5155.82813	256.31247	50.1002

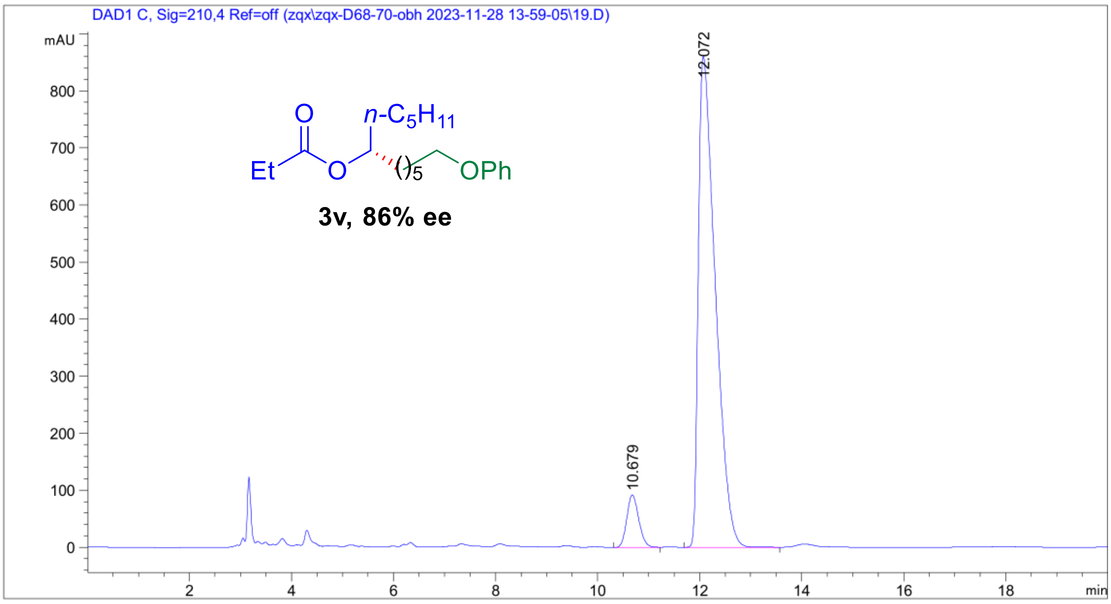

Peak #	RetTime [min]	Type	Width [min]	Area [mAU*s]	Height [mAU]	Area %
1	10.679	BB	0.2510	1489.27563	91.90667	7.0098
2	12.072	BB	0.3439	1.97563e4	860.49011	92.9902

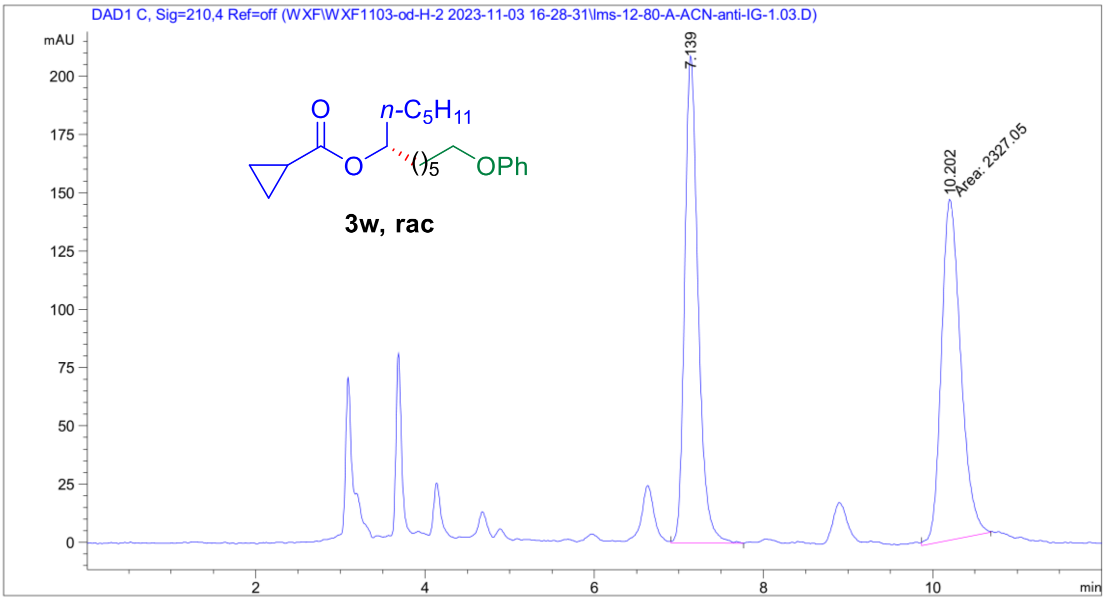

Peak #	RetTime [min]	Type	Width [min]	Area [mAU*s]	Height [mAU]	Area %
1	7.139	VV R	0.1667	2303.81104	208.88762	49.7491
2	10.202	MM	0.2652	2327.04980	146.25710	50.2509

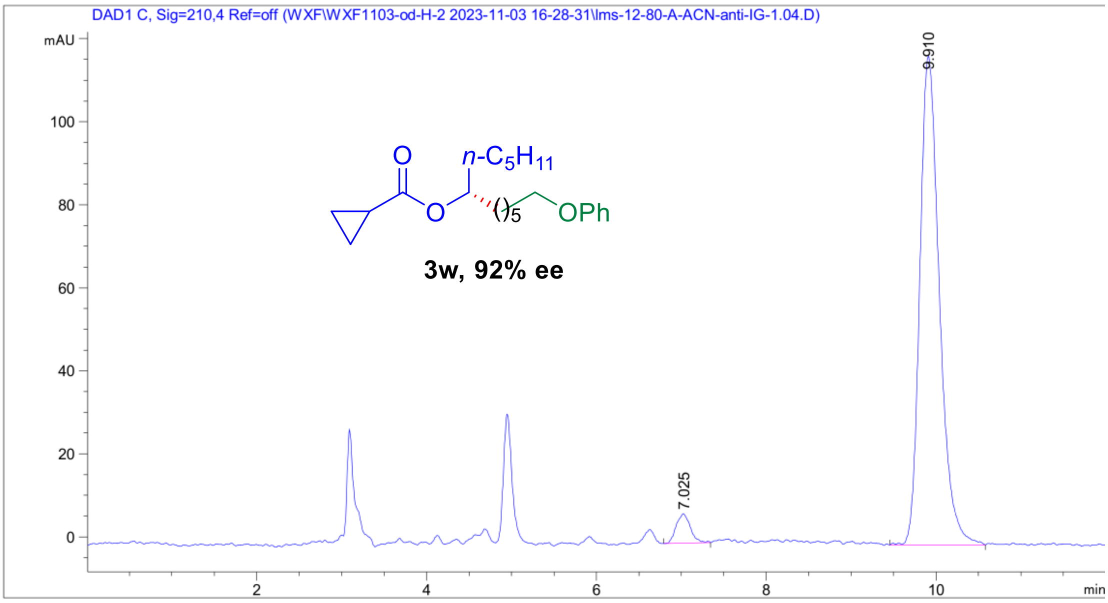

Peak #	RetTime [min]	Type	Width [min]	Area [mAU*s]	Height [mAU]	Area %
1	7.025	BV R	0.1515	82.66106	7.11471	4.2000
2	9.910	VB R	0.2448	1885.44885	117.76001	95.8000

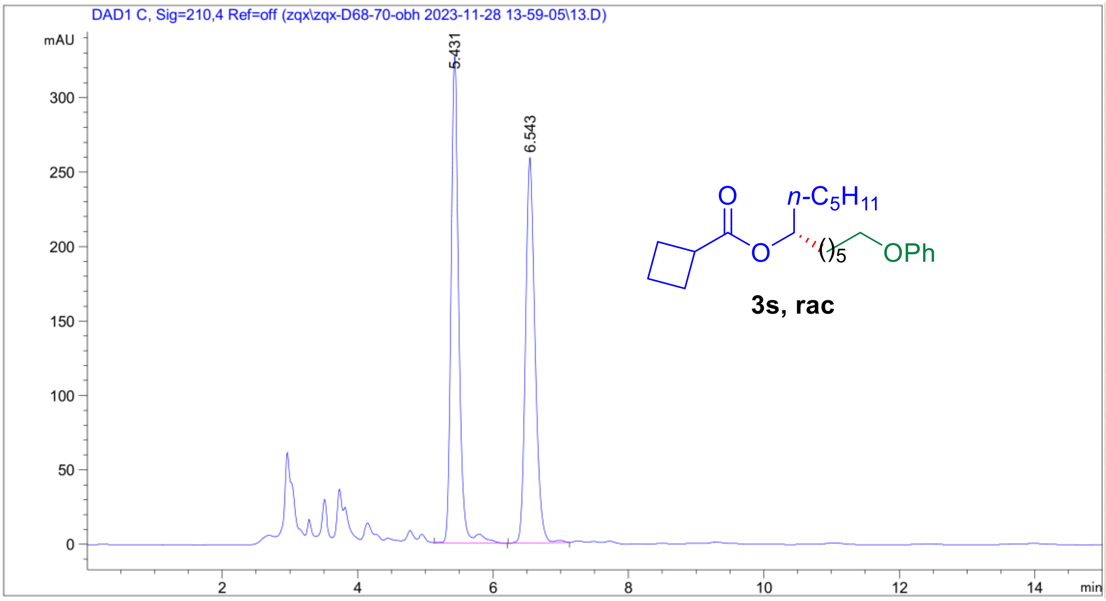

Peak #	RetTime [min]	Type	Width [min]	Area [mAU*s]	Height [mAU]	Area %
1	5.431	BV R	0.1196	2653.17749	327.14386	50.7550
2	6.543	BV R	0.1513	2574.23901	259.23291	49.2450

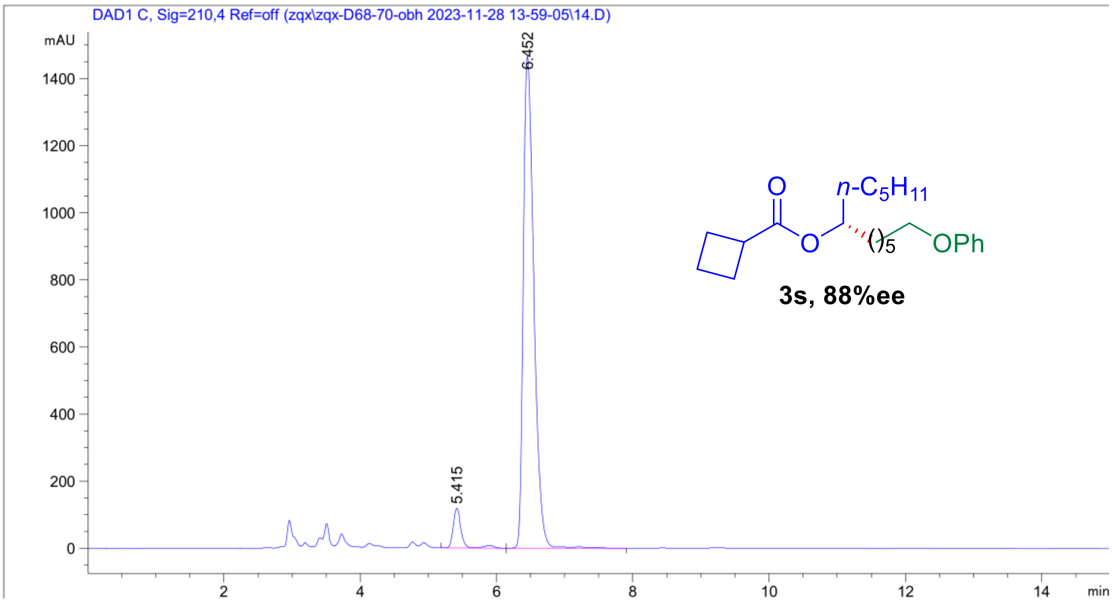

Peak #	RetTime [min]	Type	Width [min]	Area [mAU*s]	Height [mAU]	Area %
1	5.415	BV R	0.1181	1009.53204	118.87167	6.0376
2	6.452	BV R	0.1659	1.57113e4	1465.54431	93.9624

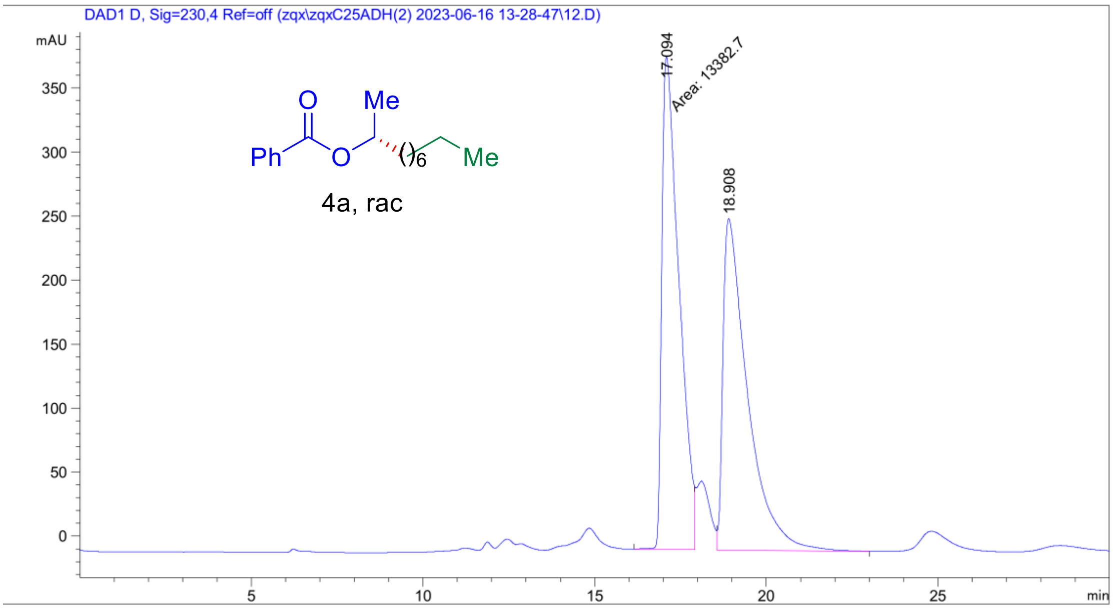

Peak #	RetTime [min]	Type	Width [min]	Area [mAU*s]	Height [mAU]	Area %
1	17.094	MF	0.5794	1.33827e4	384.93442	50.9246
2	18.908	VB	0.7132	1.28968e4	258.88742	49.0754

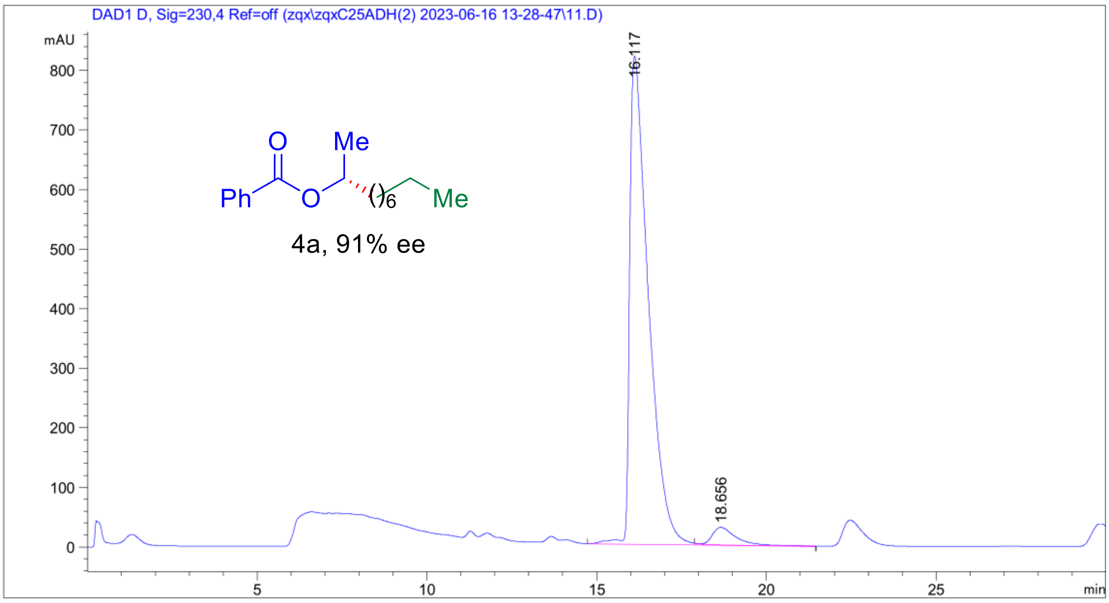

Peak #	RetTime [min]	Type	Width [min]	Area [mAU*s]	Height [mAU]	Area %
1	16.117	VV R	0.5598	3.12909e4	819.50201	95.6480
2	18.656	VB E	0.7070	1423.74683	29.71255	4.3520

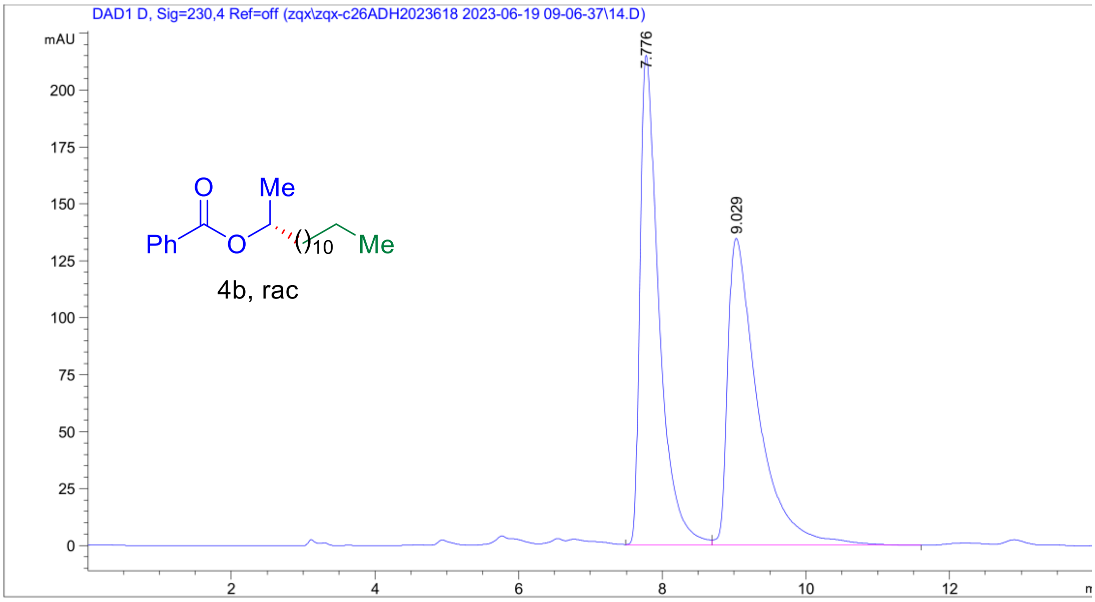

Peak #	RetTime [min]	Type	Width [min]	Area [mAU*s]	Height [mAU]	Area %
1	7.776	BV	0.2729	3955.47998	214.78363	50.1889
2	9.029	VB	0.4243	3925.70605	134.57524	49.8111

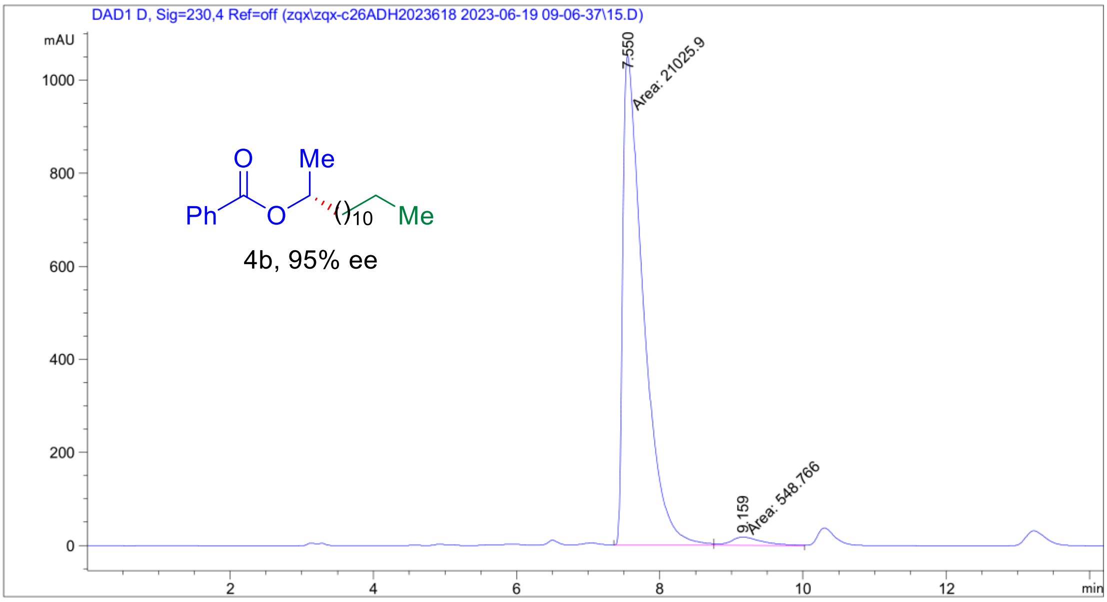

Peak #	RetTime [min]	Type	Width [min]	Area [mAU*s]	Height [mAU]	Area %
1	7.550	MF	0.3334	2.10259e4	1051.00623	97.4564
2	9.159	FM	0.5147	548.76556	17.76874	2.5436

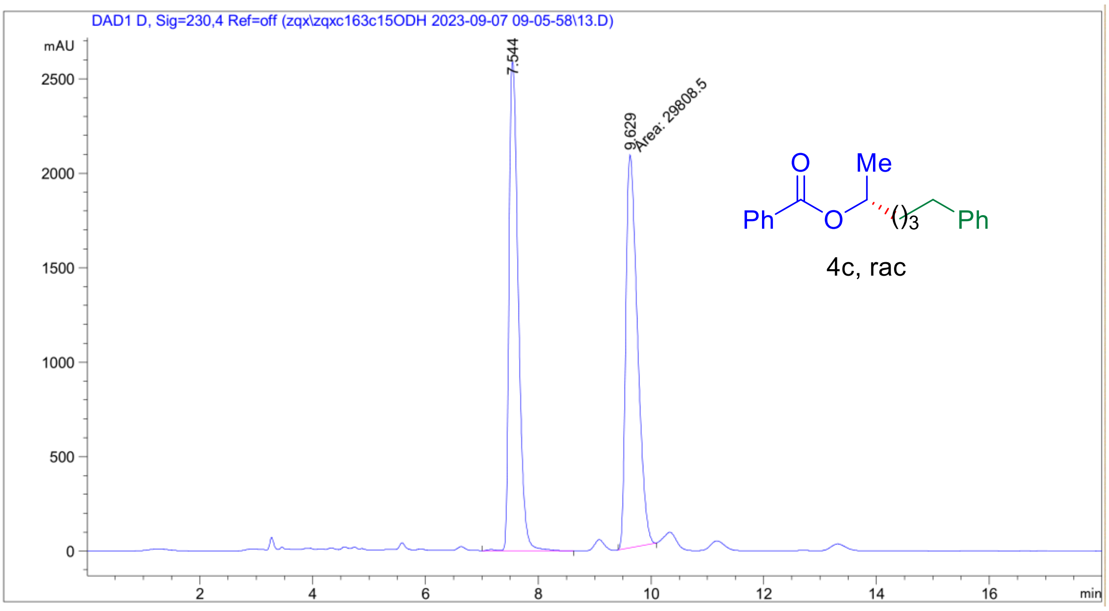

Peak #	RetTime [min]	Type	Width [min]	Area [mAU*s]	Height [mAU]	Area %
1	7.544	VB R	0.1765	2.94698e4	2588.36987	49.7143
2	9.629	MM	0.2387	2.98085e4	2081.02173	50.2857

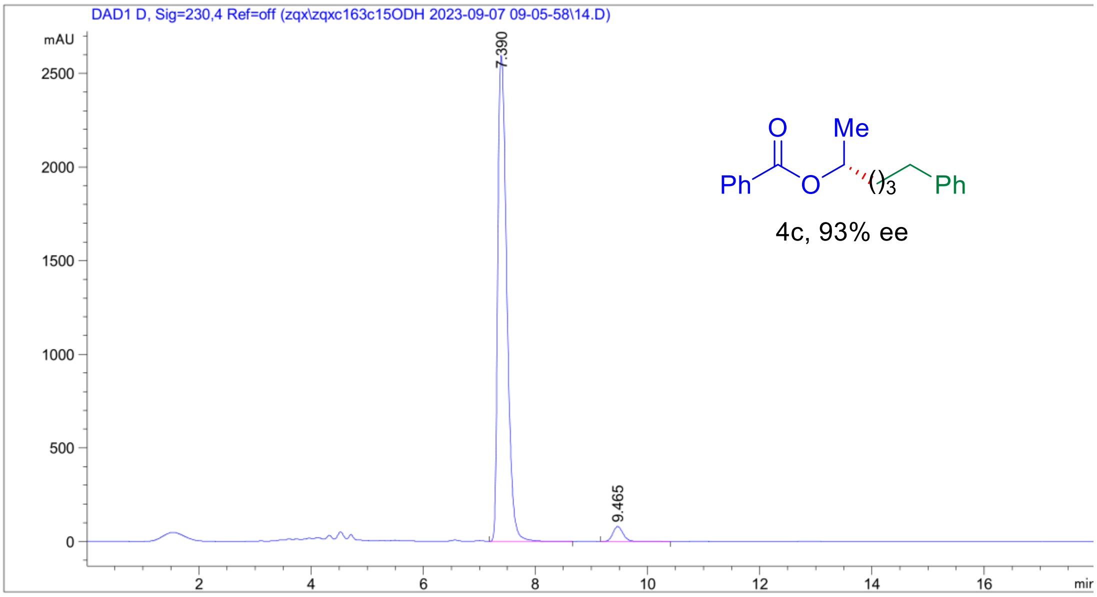

Peak #	RetTime [min]	Type	Width [min]	Area [mAU*s]	Height [mAU]	Area %
1	7.390	VB	0.1744	2.90036e4	2594.97607	96.4928
2	9.465	BV R	0.1939	1054.17932	80.57936	3.5072

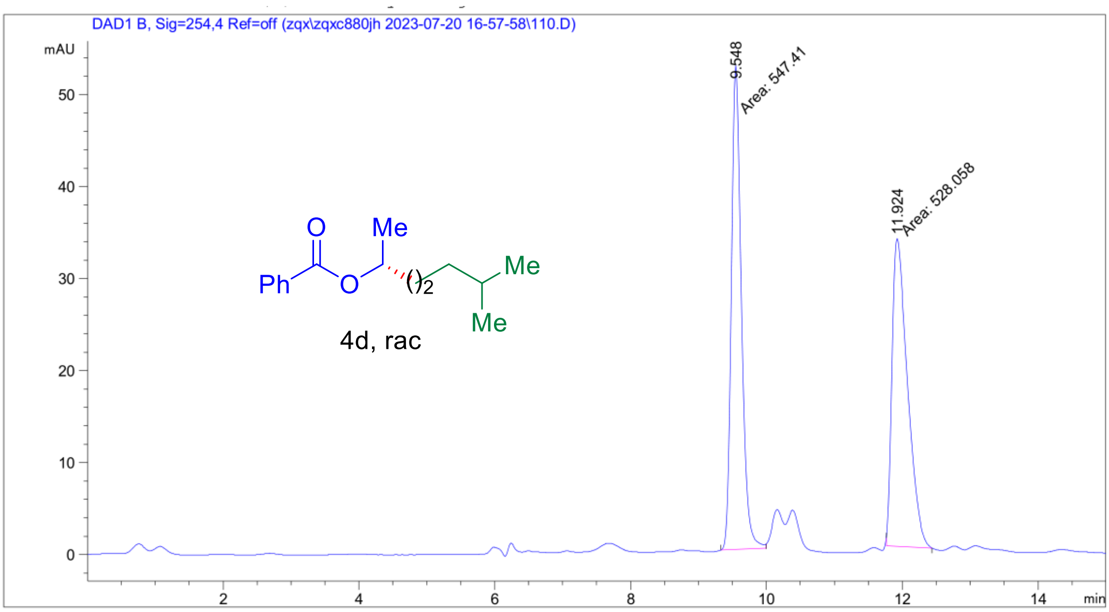

Peak #	RetTime [min]	Type	Width [min]	Area [mAU*s]	Height [mAU]	Area %
1	9.548	PM	0.1733	547.40973	52.65744	50.8997
2	11.924	MM	0.2633	528.05780	33.42463	49.1003

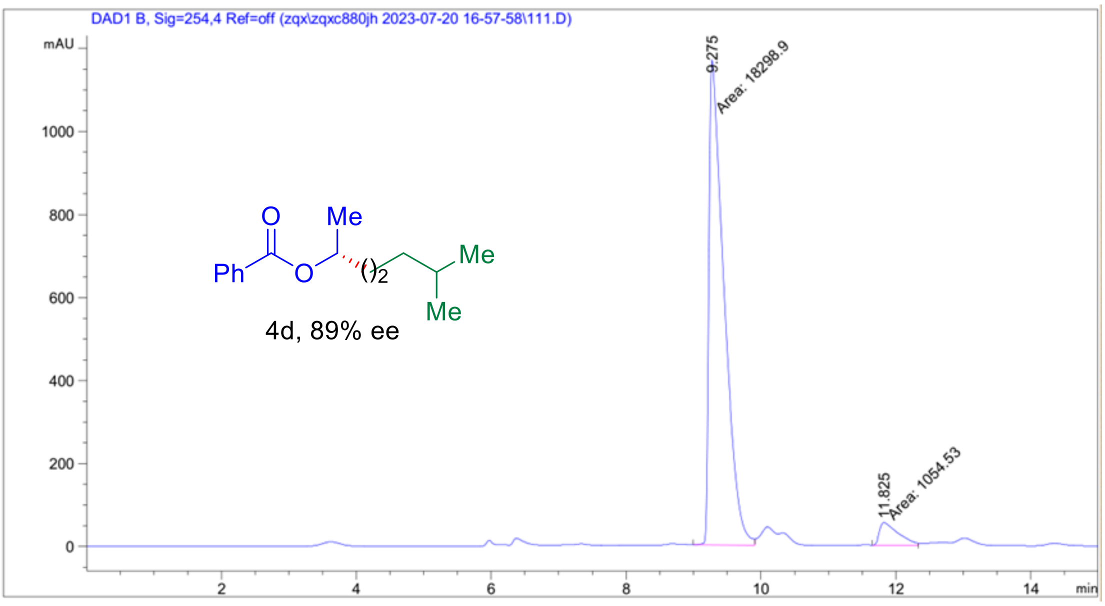

Peak #	RetTime [min]	Type	Width [min]	Area [mAU*s]	Height [mAU]	Area %
1	9.275	MF	0.2606	1.82989e4	1170.14319	94.5512
2	11.825	MF	0.3242	1054.52771	54.20939	5.4488

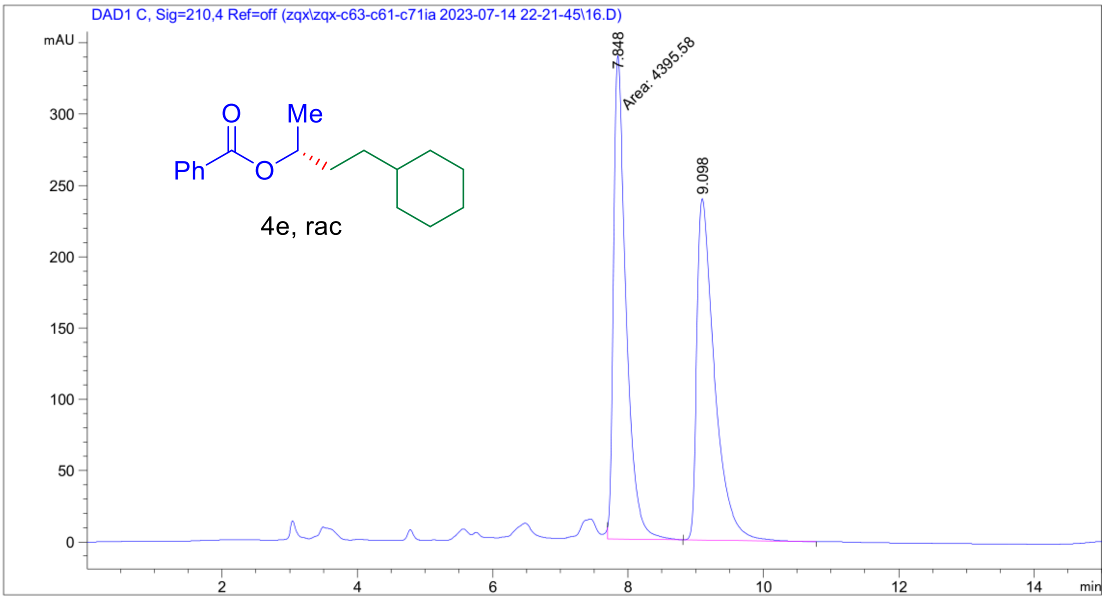

Peak #	RetTime [min]	Type	Width [min]	Area [mAU*s]	Height [mAU]	Area %
1	7.848	FM	0.2160	4395.58350	339.18066	50.2198
2	9.098	BB	0.2681	4357.11279	239.62402	49.7802

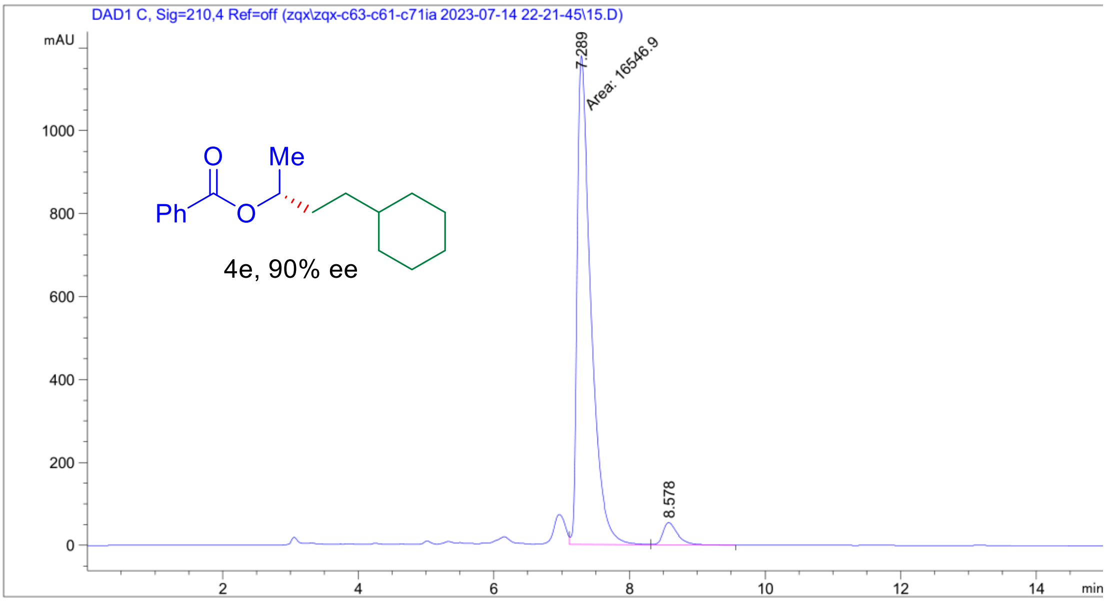

Peak #	RetTime [min]	Type	Width [min]	Area [mAU*s]	Height [mAU]	Area %
1	7.289	FM	0.2341	1.65469e4	1178.10486	95.3467
2	8.578	VB	0.2226	807.56055	53.96977	4.6533

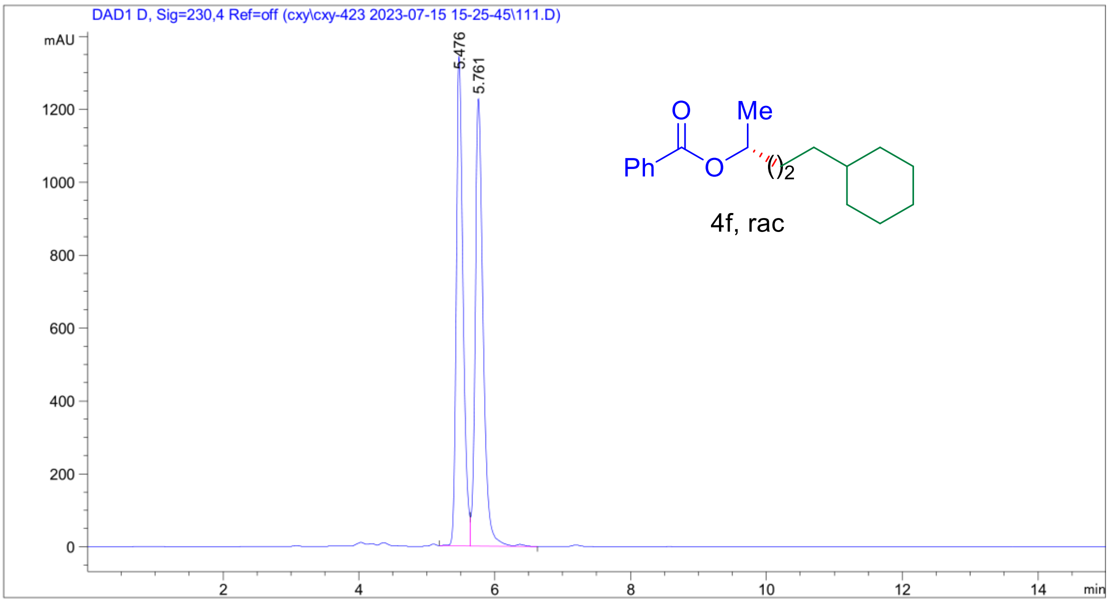

Peak #	RetTime [min]	Type	Width [min]	Area [mAU*s]	Height [mAU]	Area %
1	5.476	VV R	0.1088	9541.70410	1343.74573	48.7244
2	5.761	VV R	0.1230	1.00413e4	1228.41943	51.2756

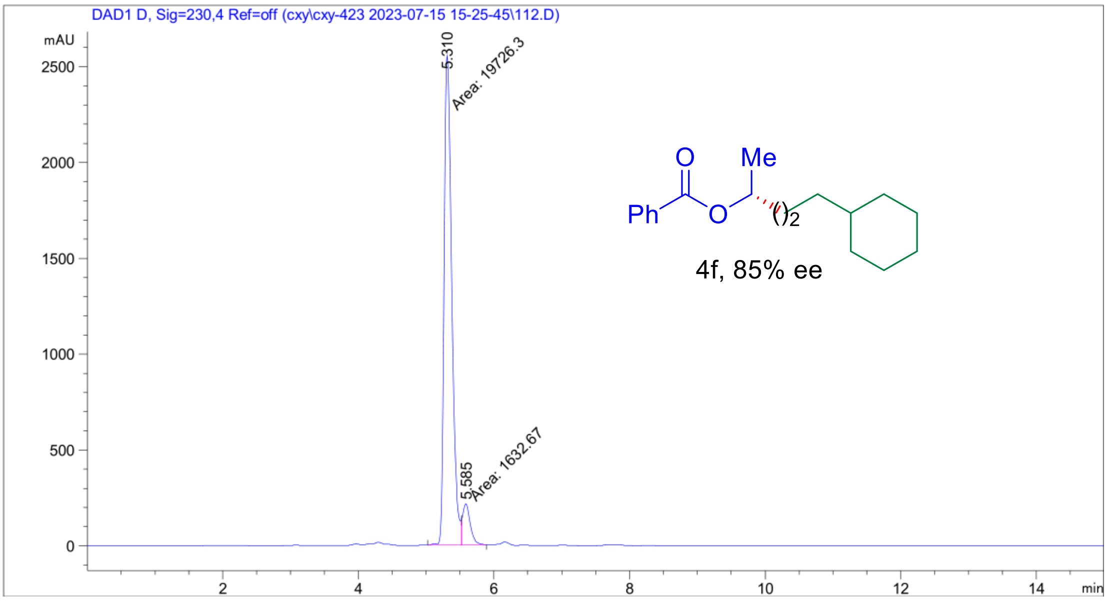

Peak #	RetTime [min]	Type	Width [min]	Area [mAU*s]	Height [mAU]	Area %
1	5.310	MF	0.1287	1.97263e4	2553.83936	92.3561
2	5.585	FM	0.1270	1632.66956	214.20558	7.6439

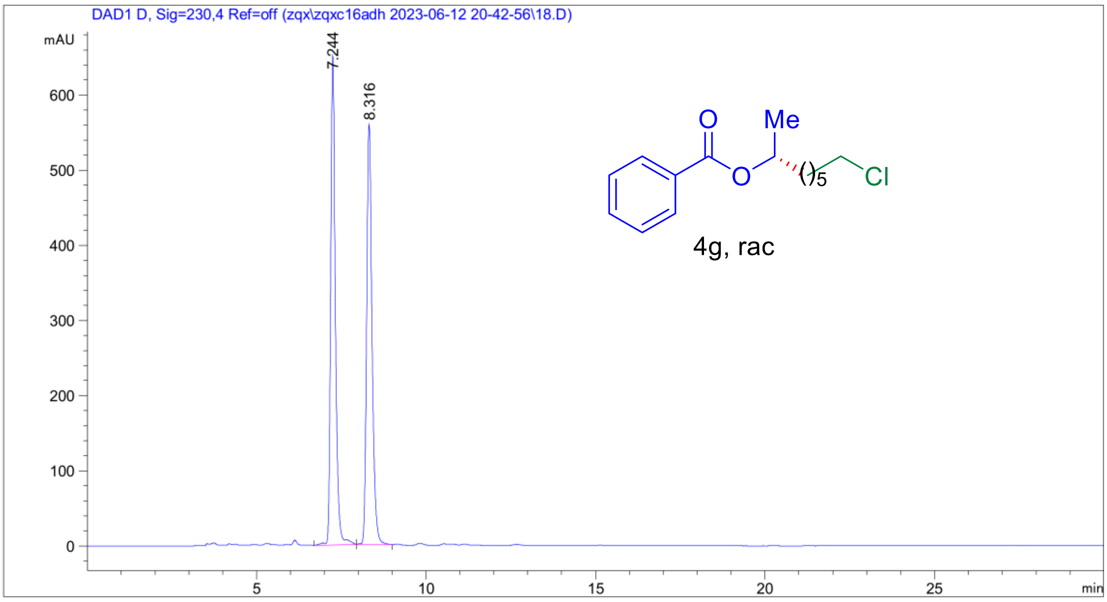

Peak #	RetTime [min]	Type	Width [min]	Area [mAU*s]	Height [mAU]	Area %
1	7.244	VV R	0.1459	6346.68066	651.03583	50.6066
2	8.316	BB	0.1673	6194.52051	559.01691	49.3934

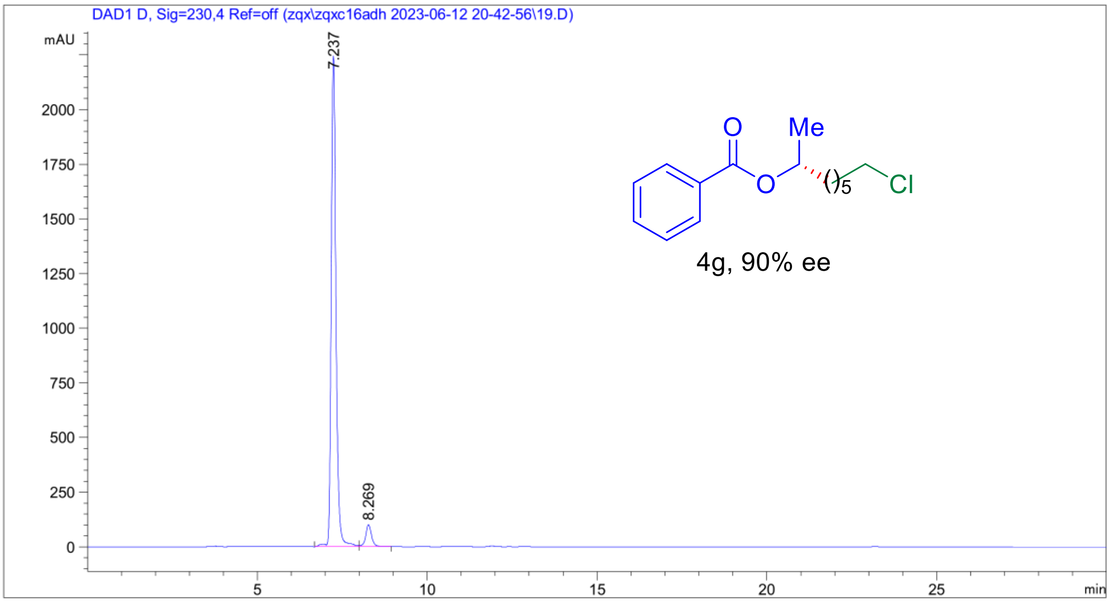

Peak #	RetTime [min]	Type	Width [min]	Area [mAU*s]	Height [mAU]	Area %
1	7.237	VV R	0.1499	2.20982e4	2248.11426	94.8198
2	8.269	VB	0.1772	1207.27966	101.16129	5.1802

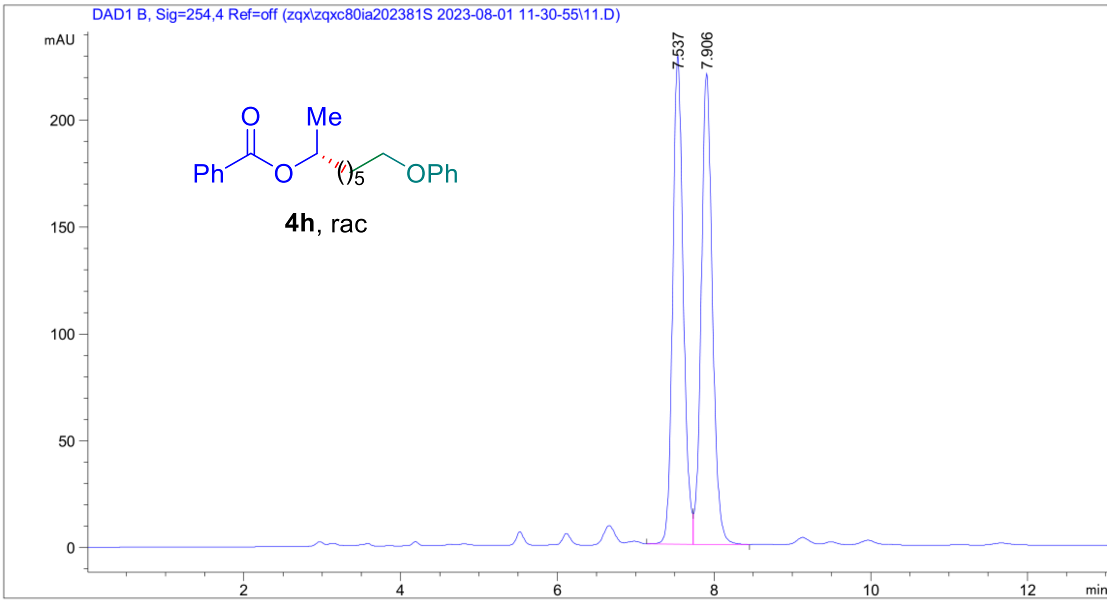

Peak #	RetTime [min]	Type	Width [min]	Area [mAU*s]	Height [mAU]	Area %
1	7.537	BV	0.1432	2139.63354	228.54527	49.4681
2	7.906	VB	0.1517	2185.64624	220.34450	50.5319

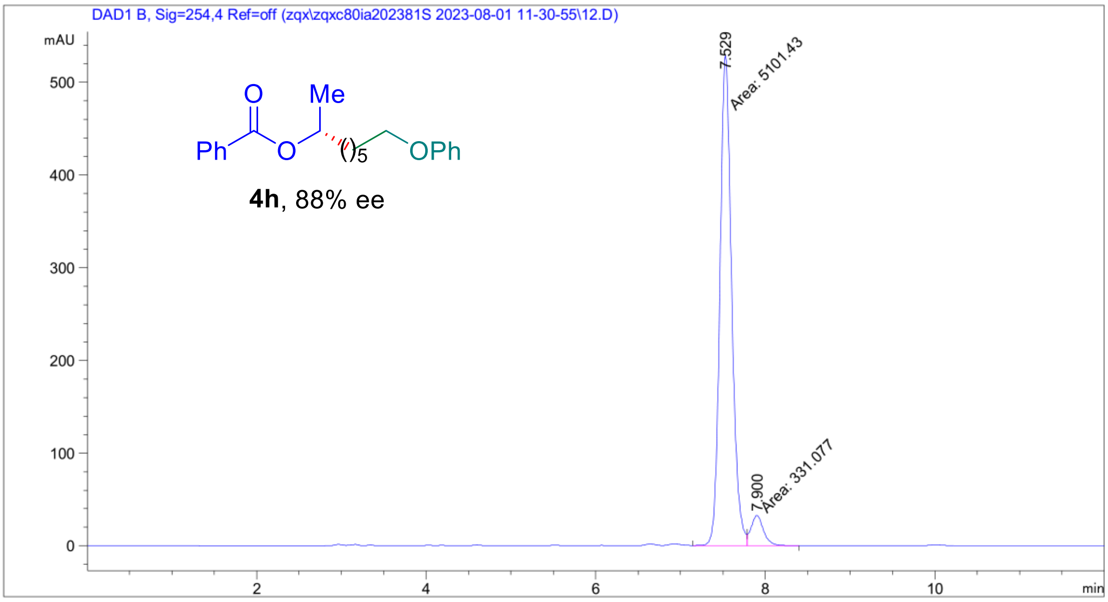

Peak #	RetTime [min]	Type	Width [min]	Area [mAU*s]	Height [mAU]	Area %
1	7.529	MF	0.1609	5101.43164	528.58417	93.9056
2	7.900	FM	0.1695	331.07700	32.54933	6.0944

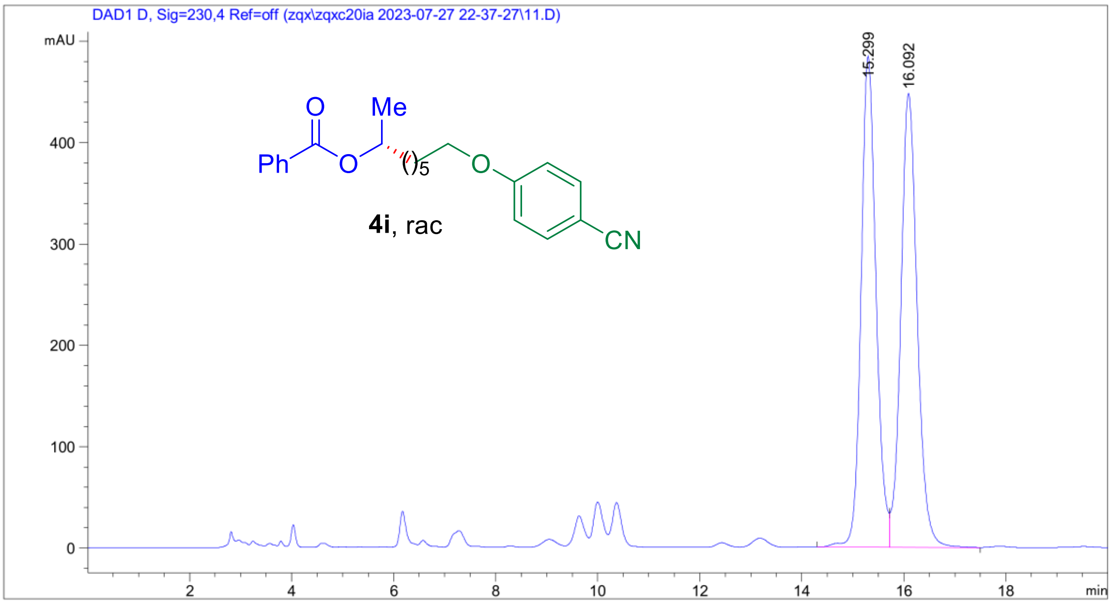

Peak #	RetTime [min]	Type	Width [min]	Area [mAU*s]	Height [mAU]	Area %
1	15.299	BV	0.3138	9873.64648	484.38776	49.7071
2	16.092	VB	0.3402	9989.99512	447.90103	50.2929

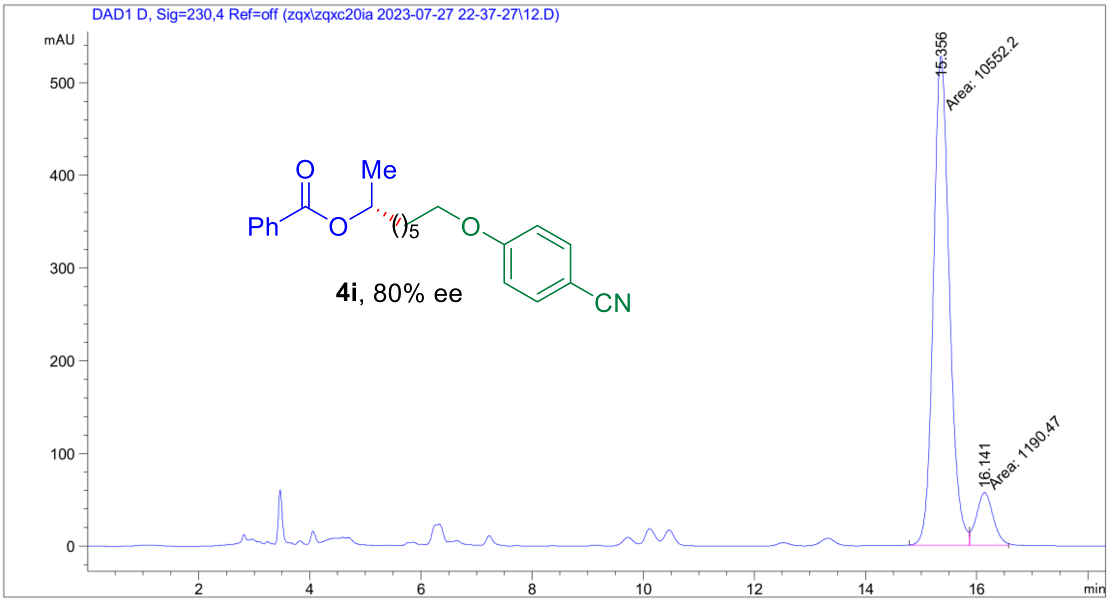

Peak #	RetTime [min]	Type	Width [min]	Area [mAU*s]	Height [mAU]	Area %
1	15.356	MF	0.3333	1.05522e4	527.68994	89.8620
2	16.141	FM	0.3486	1190.47278	56.92372	10.1380

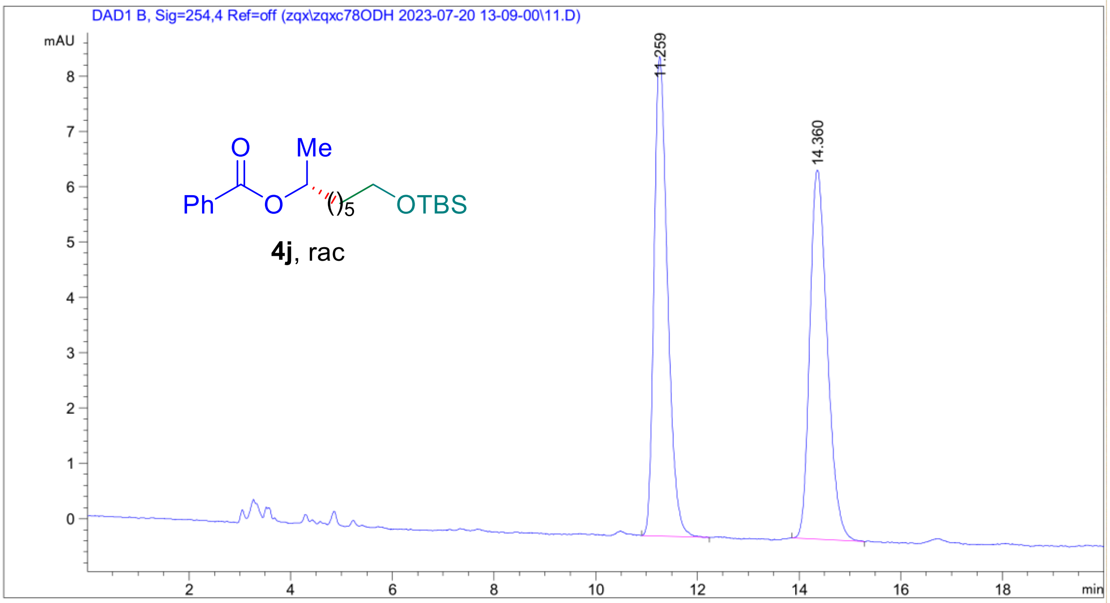

Peak #	RetTime [min]	Type	Width [min]	Area [mAU*s]	Height [mAU]	Area %
1	11.259	BB	0.2835	160.01901	8.66472	50.1644
2	14.360	BB	0.3677	158.97017	6.67852	49.8356

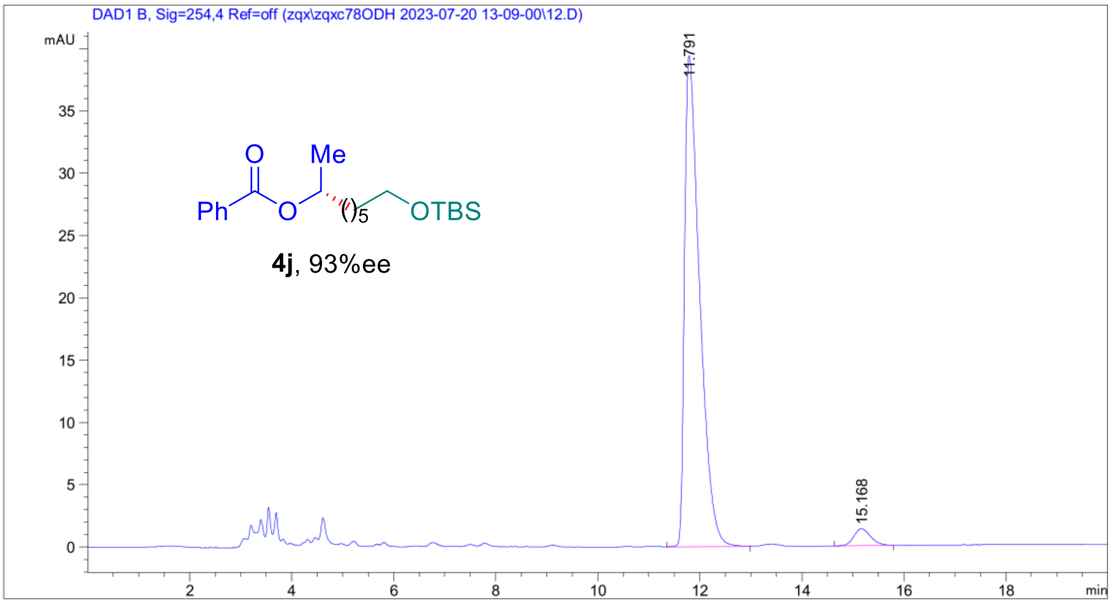

Peak #	RetTime [min]	Type	Width [min]	Area [mAU*s]	Height [mAU]	Area %
1	11.791	BB	0.3057	809.31567	39.39007	96.2571
2	15.168	BB	0.3423	31.46935	1.36822	3.7429

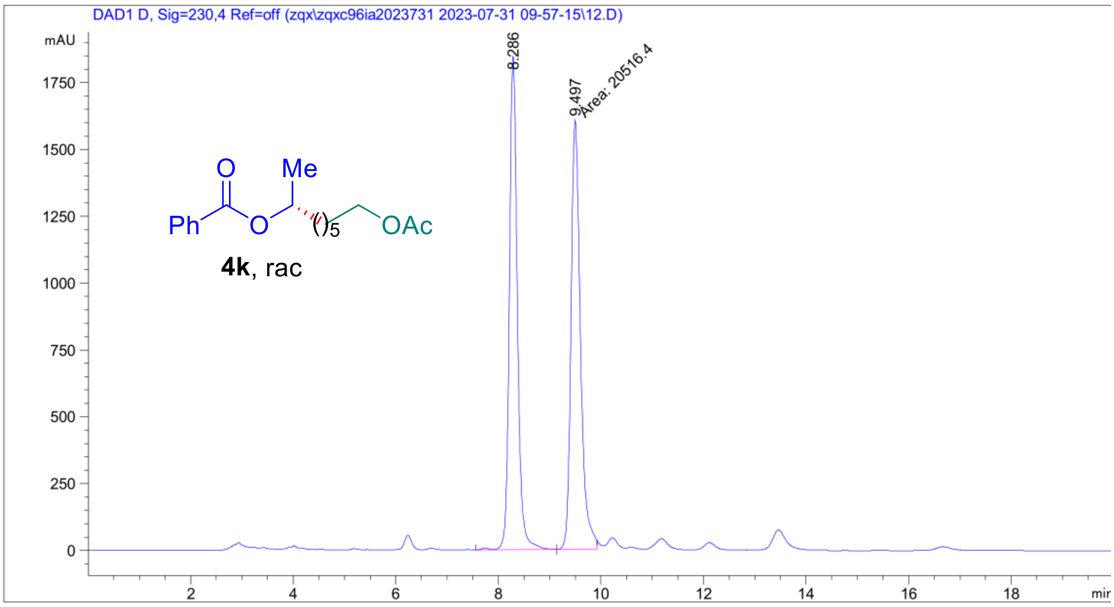

Peak #	RetTime [min]	Type	Width [min]	Area [mAU*s]	Height [mAU]	Area %
1	8.286	VB R	0.1645	2.00760e4	1846.22754	49.4575
2	9.497	MF	0.2130	2.05164e4	1605.16797	50.5425

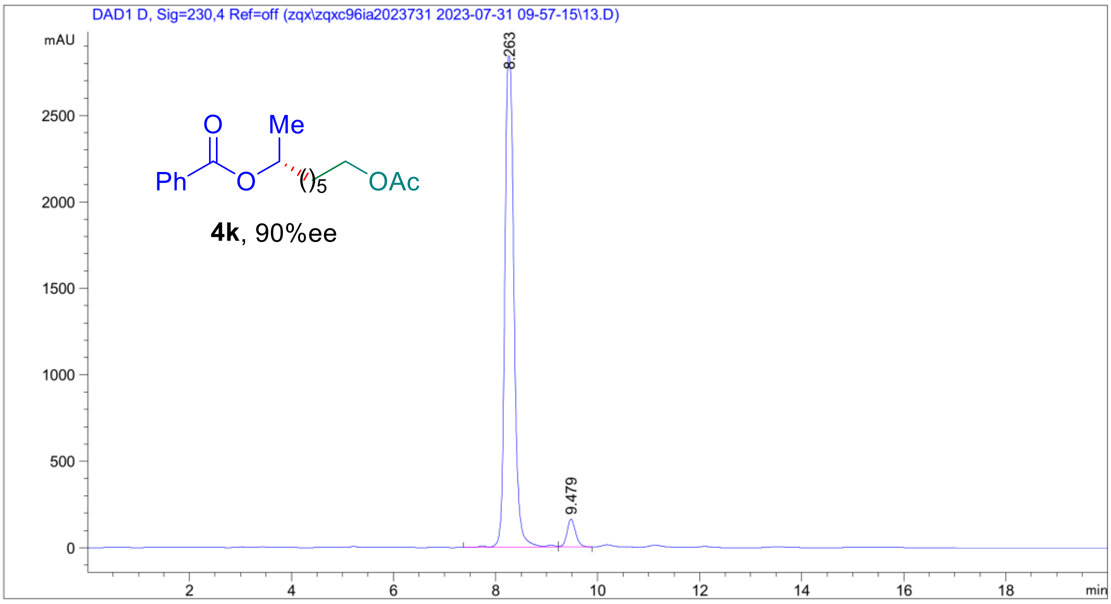

Peak #	RetTime [min]	Type	Width [min]	Area [mAU*s]	Height [mAU]	Area %
1	8.263	VV R	0.1947	3.54451e4	2841.89404	94.9261
2	9.479	VB	0.1787	1894.58142	161.70644	5.0739

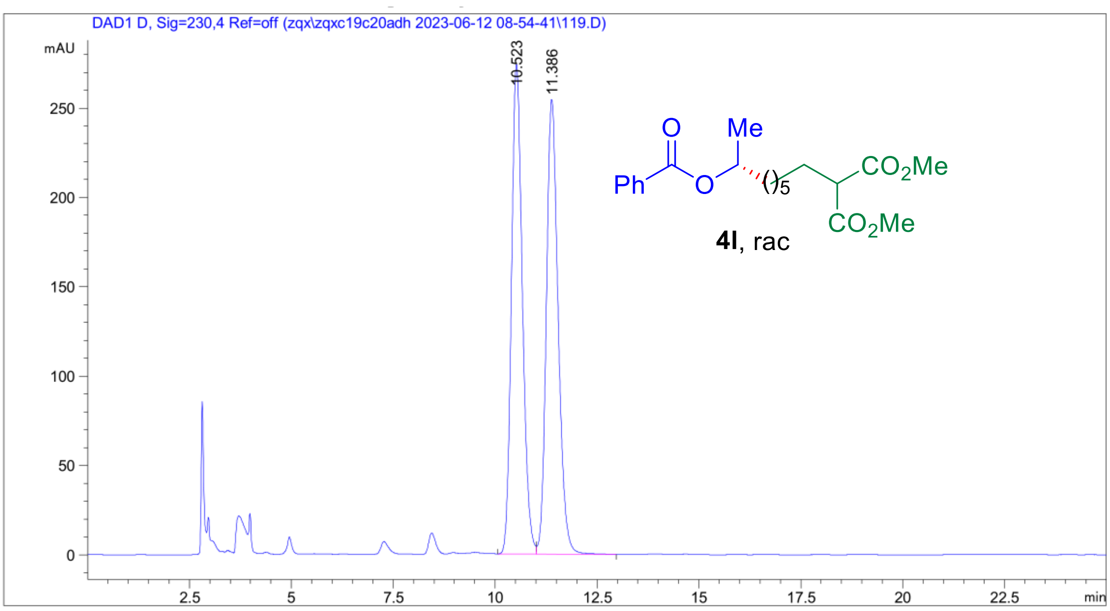

Peak #	RetTime [min]	Type	Width [min]	Area [mAU*s]	Height [mAU]	Area %
1	10.523	BV	0.2847	5091.29834	274.05908	49.8787
2	11.386	VB	0.3084	5116.06787	254.61333	50.1213

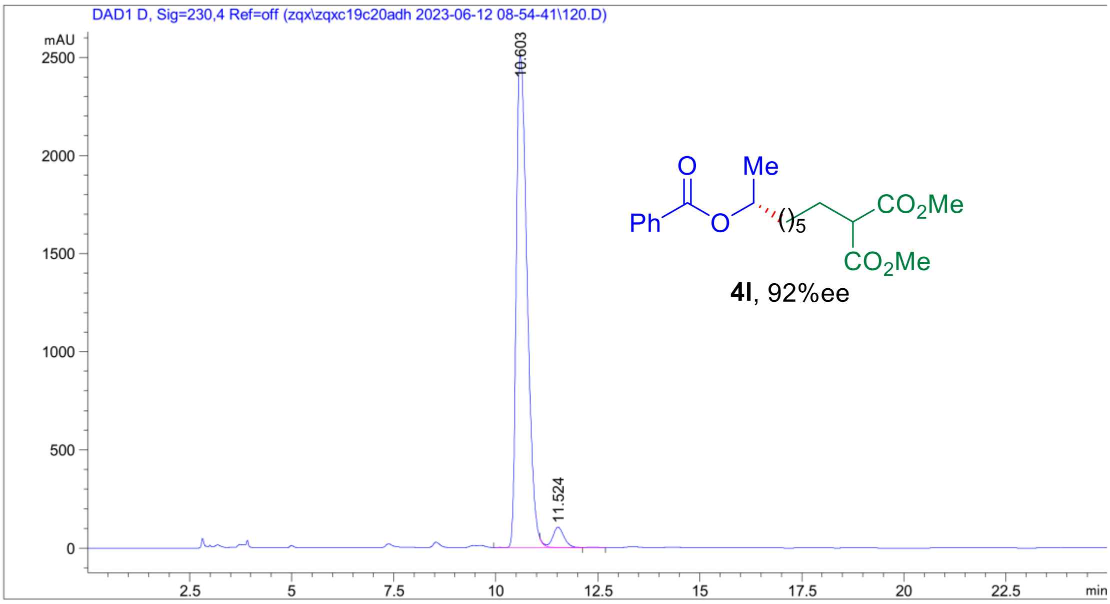

Peak #	RetTime [min]	Type	Width [min]	Area [mAU*s]	Height [mAU]	Area %
1	10.603	VV R	0.2907	4.65459e4	2503.61572	95.8831
2	11.524	VV E	0.2917	1998.51477	104.21938	4.1169

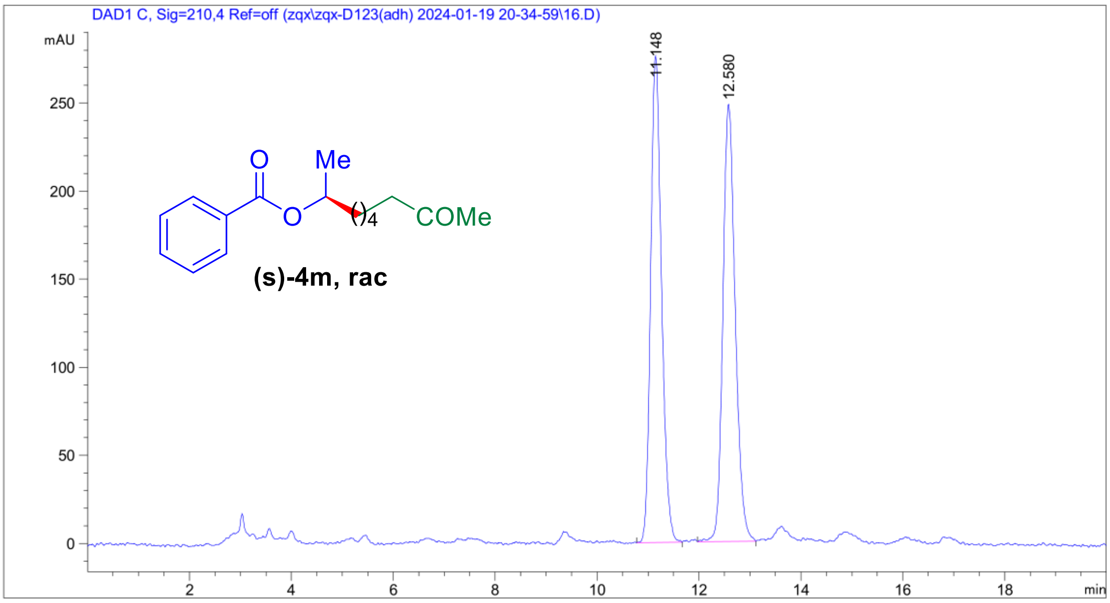

Peak #	RetTime [min]	Type	Width [min]	Area [mAU*s]	Height [mAU]	Area %
1	11.148	VV R	0.2268	4089.81836	276.12997	49.5225
2	12.580	VB R	0.2561	4168.68896	248.03644	50.4775

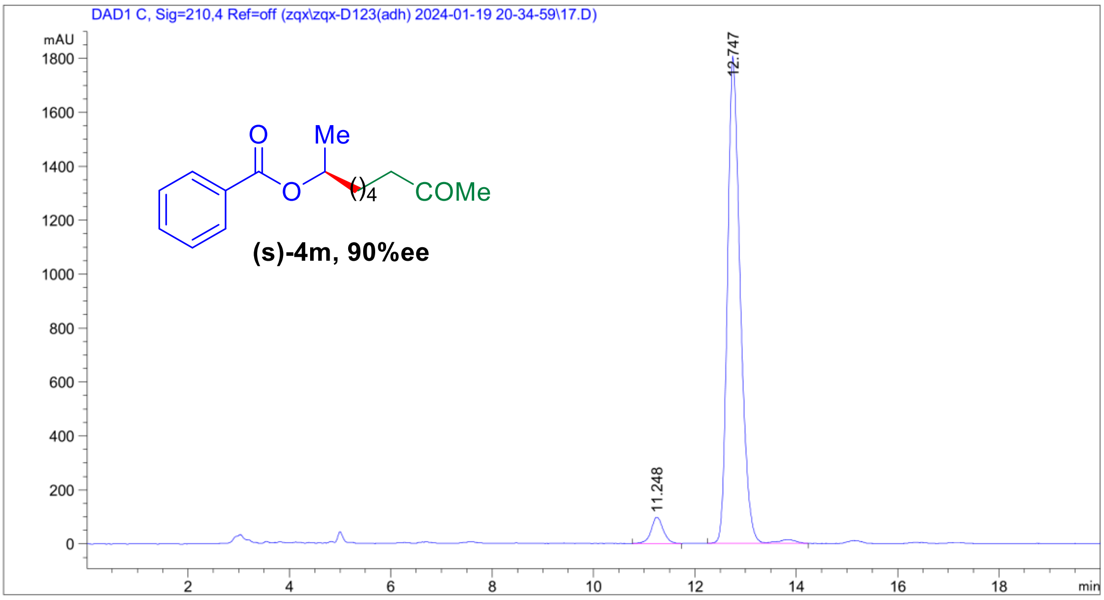

Peak #	RetTime [min]	Type	Width [min]	Area [mAU*s]	Height [mAU]	Area %
1	11.248	VV R	0.2481	1647.02368	98.00902	4.9200
2	12.747	VV R	0.2631	3.18291e4	1808.54529	95.0800

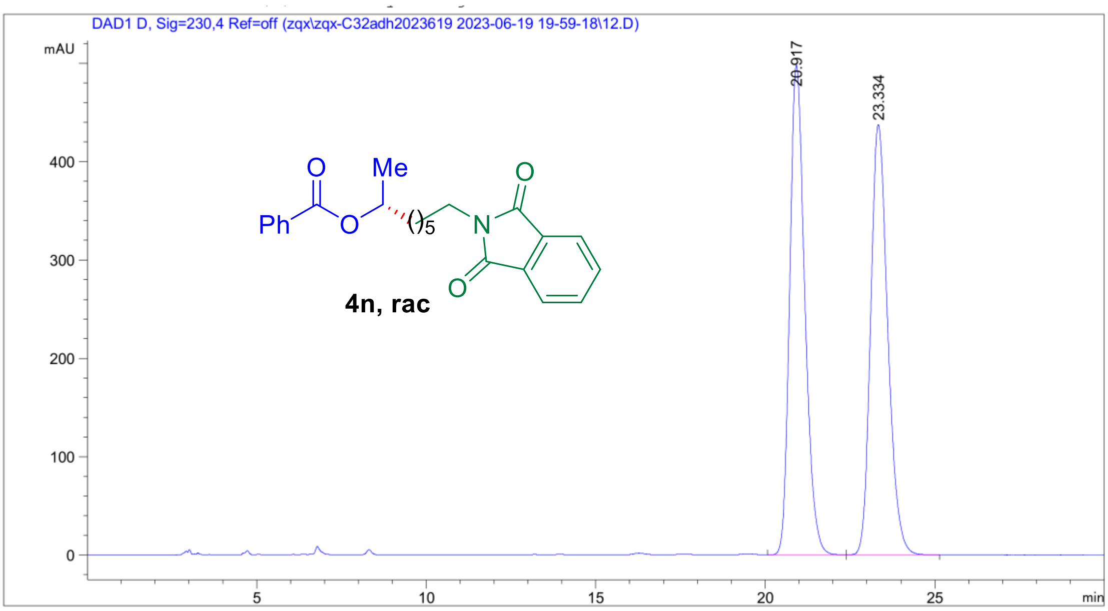

Peak #	RetTime [min]	Type	Width [min]	Area [mAU*s]	Height [mAU]	Area %
1	20.917	BB	0.4756	1.54425e4	498.17694	50.1370
2	23.334	BB	0.5395	1.53581e4	437.58942	49.8630

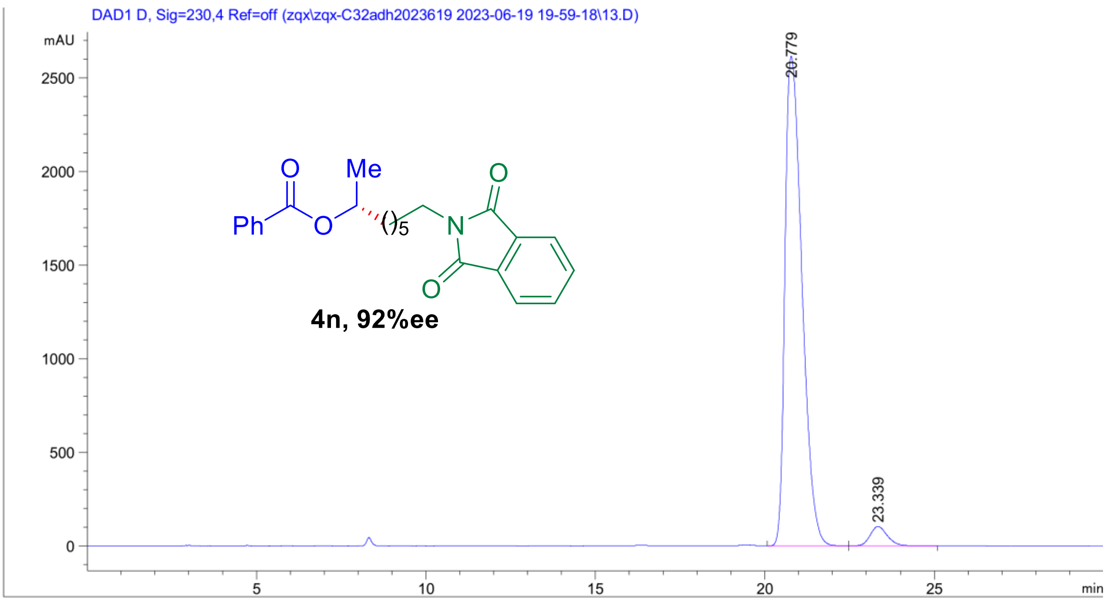

Peak #	RetTime [min]	Type	Width [min]	Area [mAU*s]	Height [mAU]	Area %
1	20.779	BB	0.5426	9.11305e4	2614.55908	96.1108
2	23.339	BB	0.5478	3687.70874	103.47418	3.8892

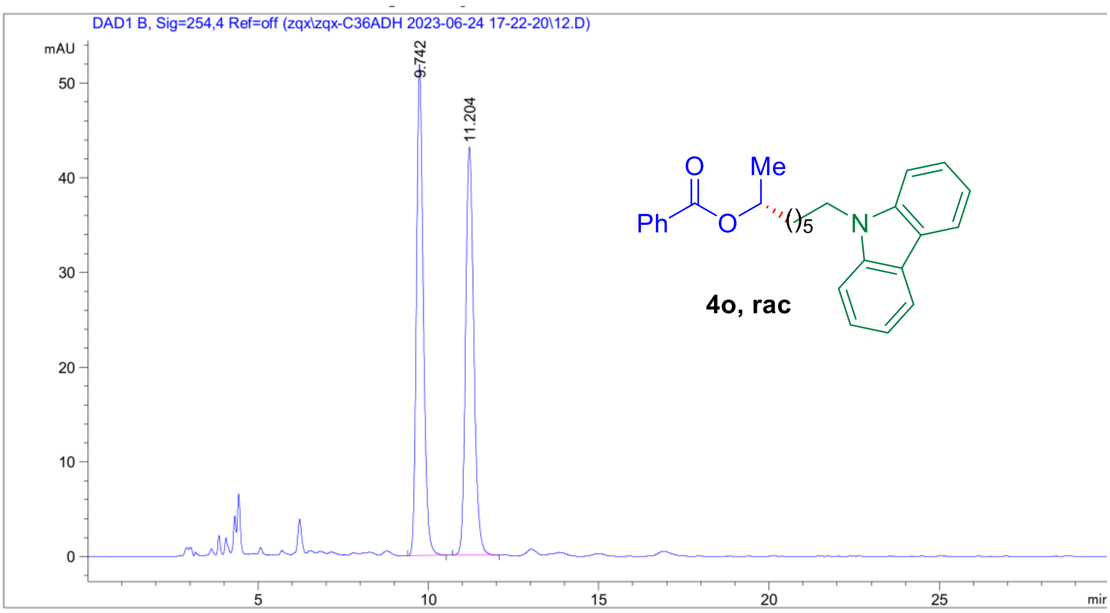

Peak #	RetTime [min]	Type	Width [min]	Area [mAU*s]	Height [mAU]	Area %
1	9.742	BB	0.2164	730.79437	51.86336	50.4517
2	11.204	BB	0.2563	717.70978	43.10427	49.5483

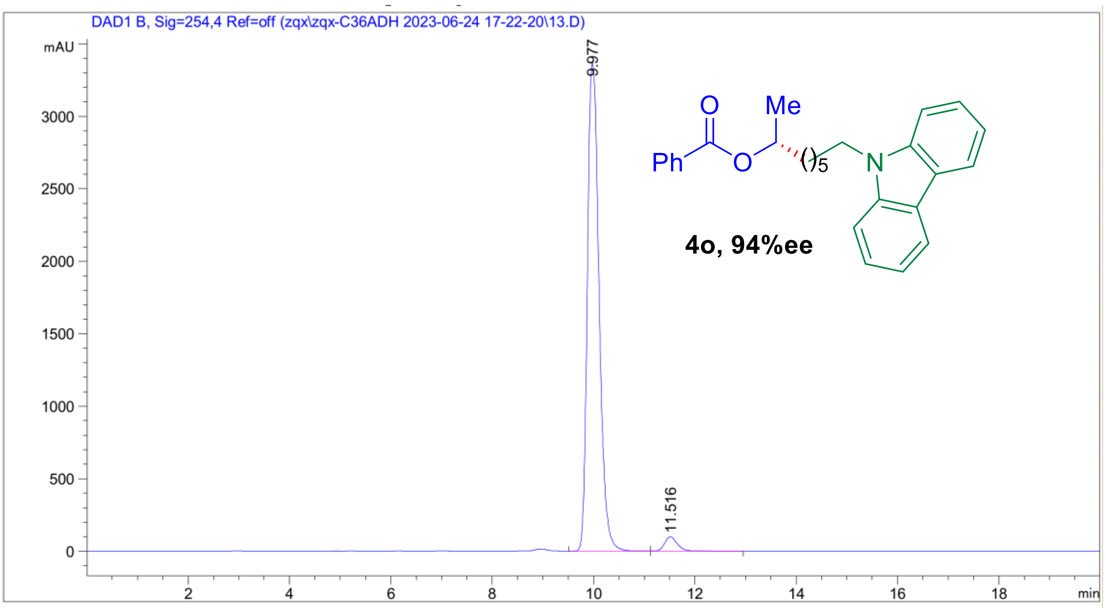

Peak #	RetTime [min]	Type	Width [min]	Area [mAU*s]	Height [mAU]	Area %
1	9.977	BV	0.2485	5.38429e4	3367.94922	96.8538
2	11.516	VB	0.2703	1749.04639	98.90898	3.1462

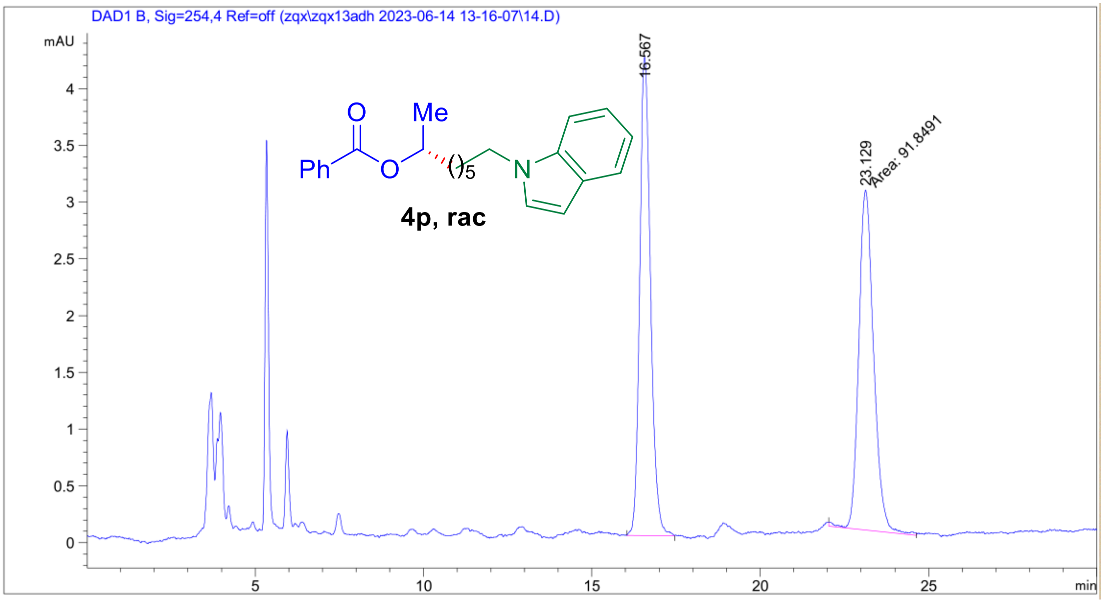

Peak #	RetTime [min]	Type	Width [min]	Area [mAU*s]	Height [mAU]	Area %
1	16.567	BB	0.3358	93.82381	4.21263	50.5318
2	23.129	MM	0.5116	91.84911	2.99251	49.4682

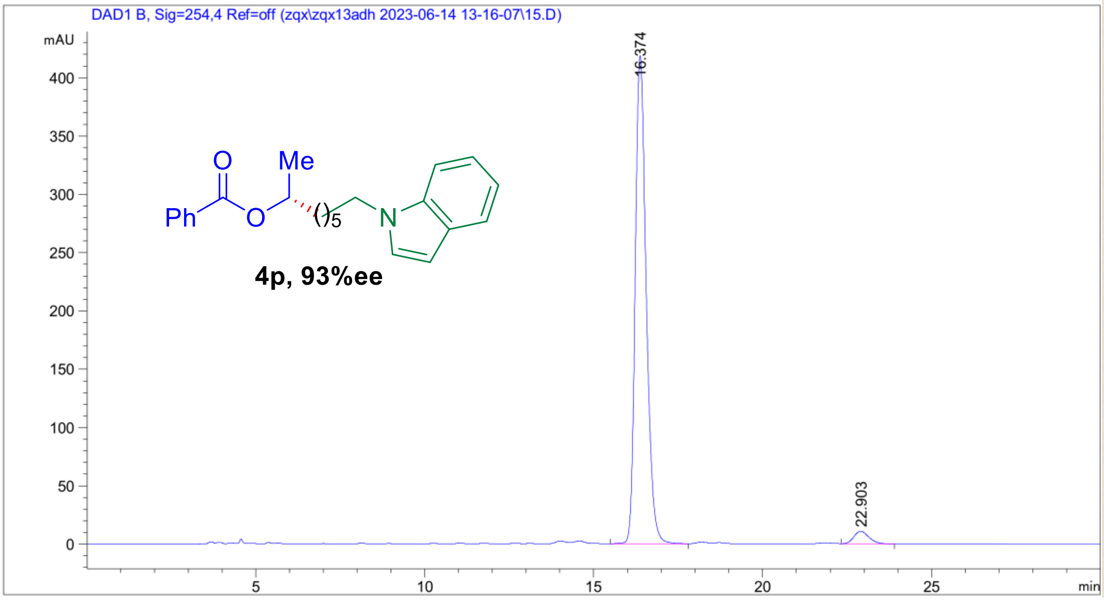

Peak #	RetTime [min]	Type	Width [min]	Area [mAU*s]	Height [mAU]	Area %
1	16.374	BB	0.3445	9416.80176	418.50095	96.6253
2	22.903	BB	0.4650	328.88678	10.68876	3.3747

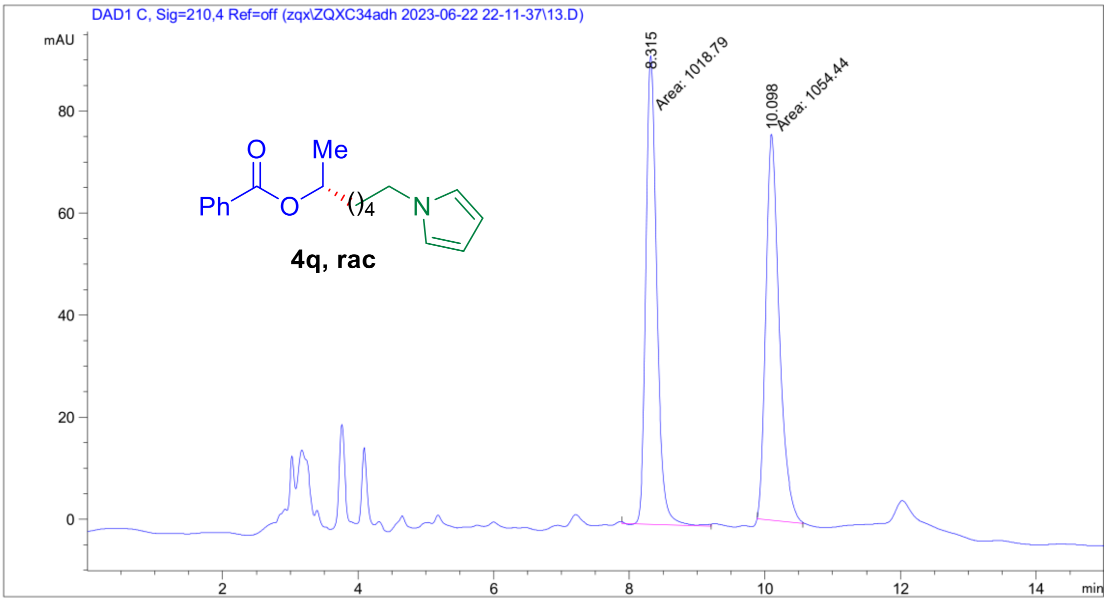

Peak #	RetTime [min]	Type	Width [min]	Area [mAU*s]	Height [mAU]	Area %
1	8.315	MM	0.1848	1018.79401	91.87798	49.1404
2	10.098	MM	0.2321	1054.43726	75.72505	50.8596

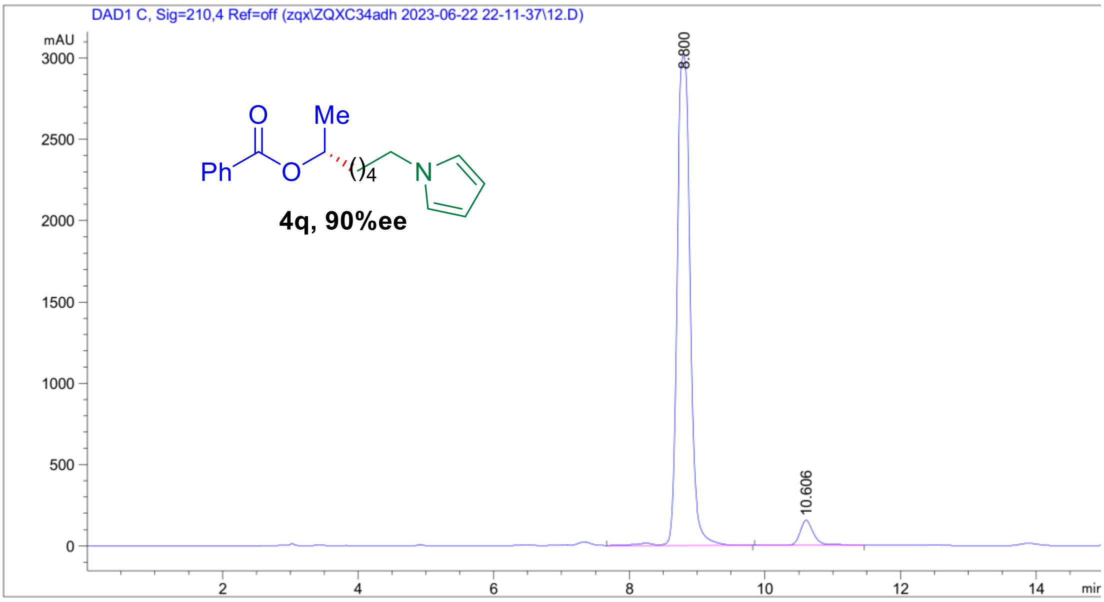

Peak #	RetTime [min]	Type	Width [min]	Area [mAU*s]	Height [mAU]	Area %
1	8.800	VB R	0.2085	4.01514e4	3009.95703	94.8920
2	10.606	VV R	0.2039	2161.32715	154.47028	5.1080

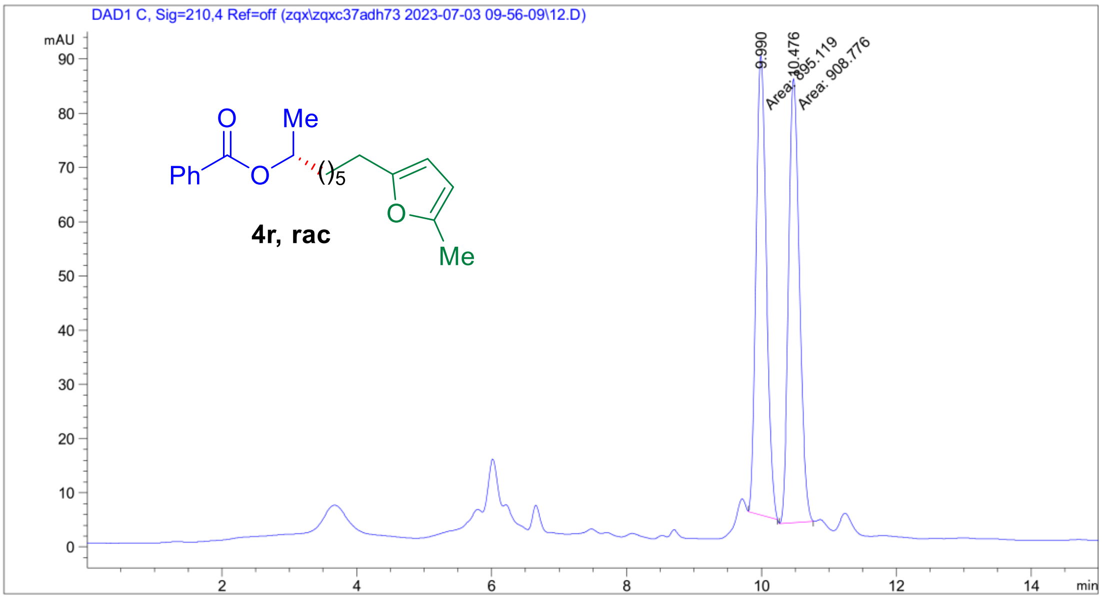

Peak #	RetTime [min]	Type	Width [min]	Area [mAU*s]	Height [mAU]	Area %
1	9.990	MP	0.1760	895.11871	84.78143	49.6215
2	10.476	MP	0.1850	908.77588	81.87817	50.3785

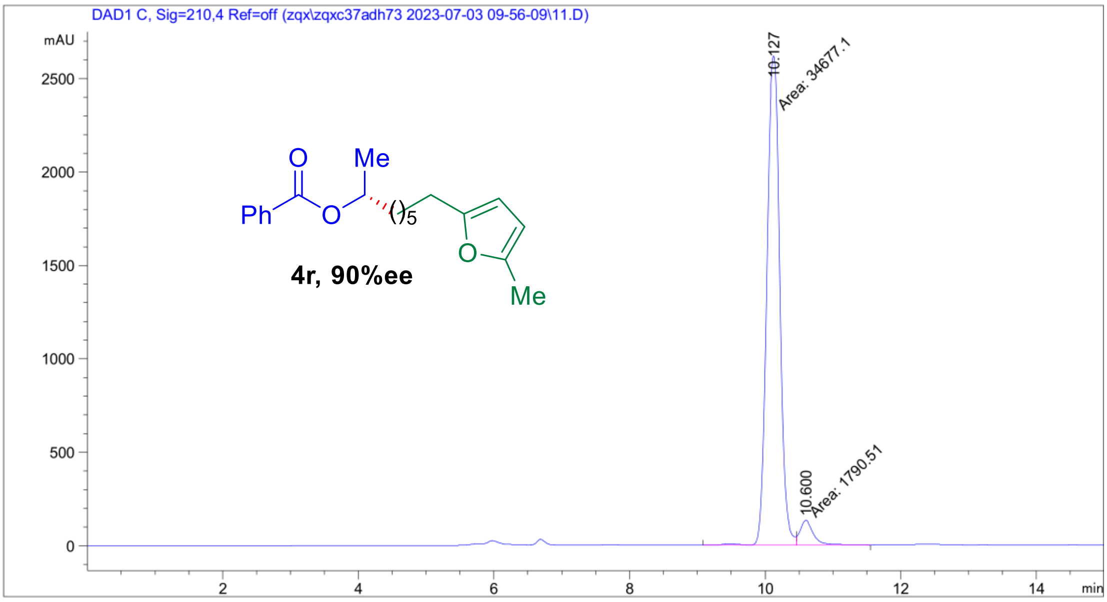

Peak #	RetTime [min]	Type	Width [min]	Area [mAU*s]	Height [mAU]	Area %
1	10.127	MF	0.2209	3.46771e4	2616.19727	95.0901
2	10.600	FM	0.2252	1790.50684	132.52052	4.9099

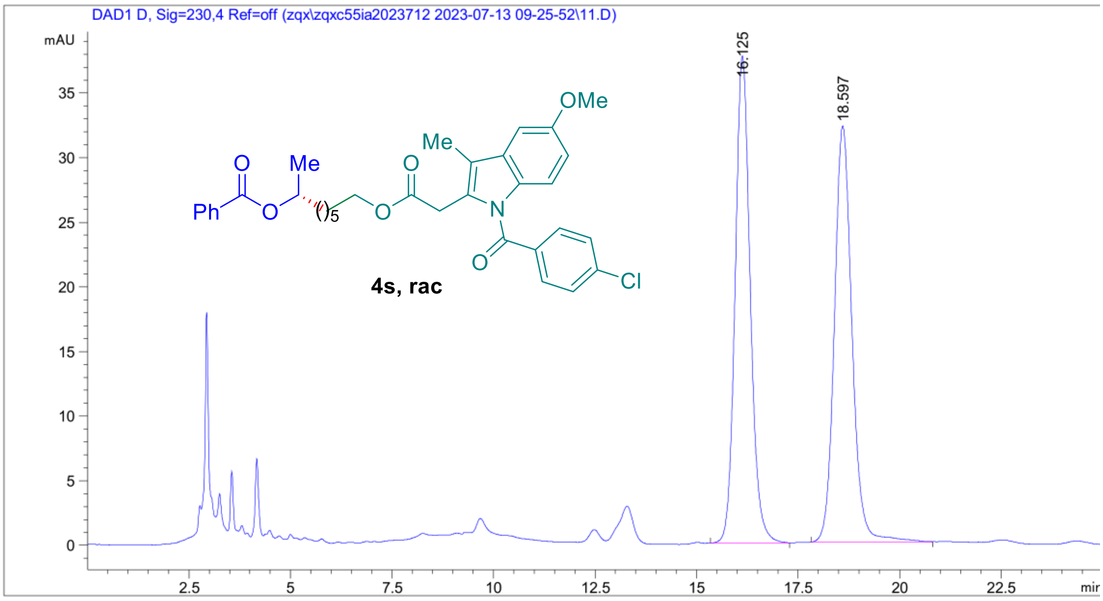

Peak #	RetTime [min]	Type	Width [min]	Area [mAU*s]	Height [mAU]	Area %
1	16.125	BB	0.3950	972.35547	37.68213	50.1447
2	18.597	BB	0.4581	966.74402	32.21761	49.8553

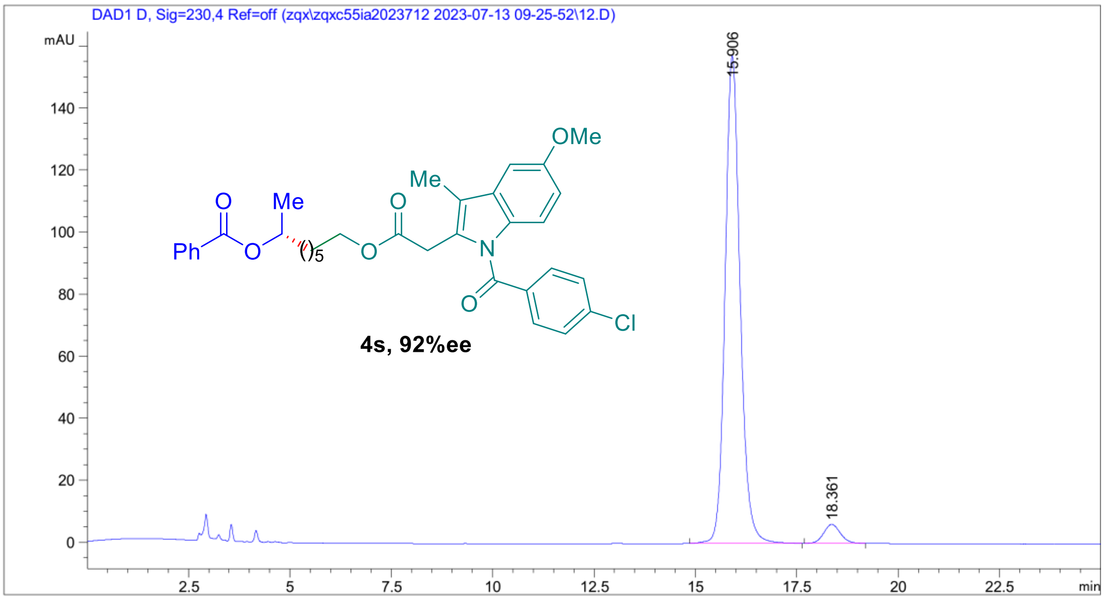

Peak #	RetTime [min]	Type	Width [min]	Area [mAU*s]	Height [mAU]	Area %
1	15.906	BB	0.3915	4033.36768	157.13470	95.8623
2	18.361	BB	0.4363	174.09019	6.11269	4.1377

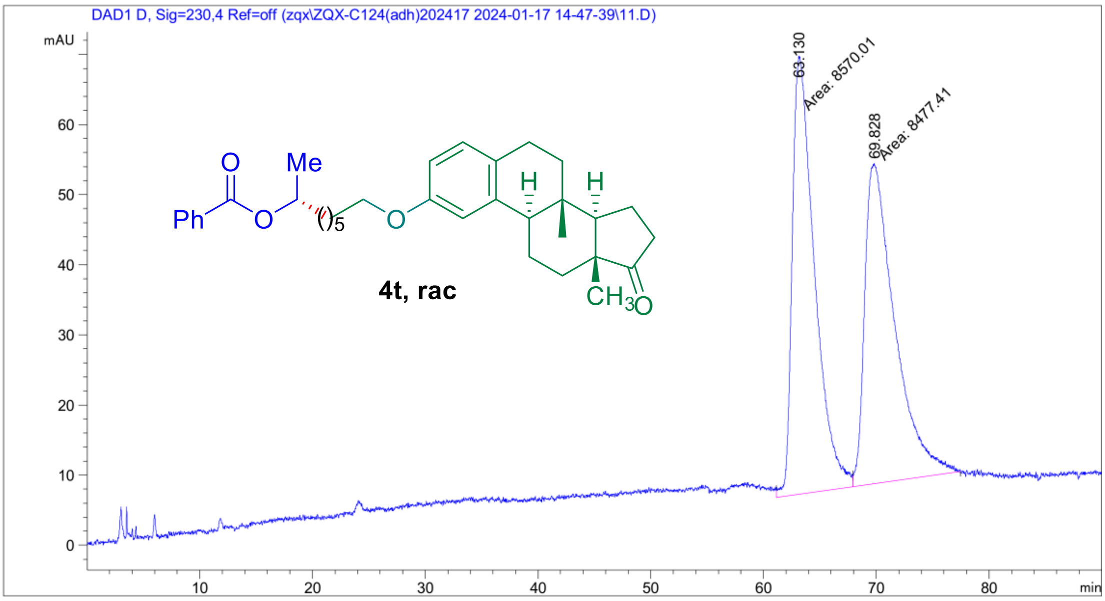

Peak #	RetTime [min]	Type	Width [min]	Area [mAU*s]	Height [mAU]	Area %
1	63.130	MF	2.2873	8570.01270	62.44722	50.2716
2	69.828	FM	3.0929	8477.40820	45.68228	49.7284

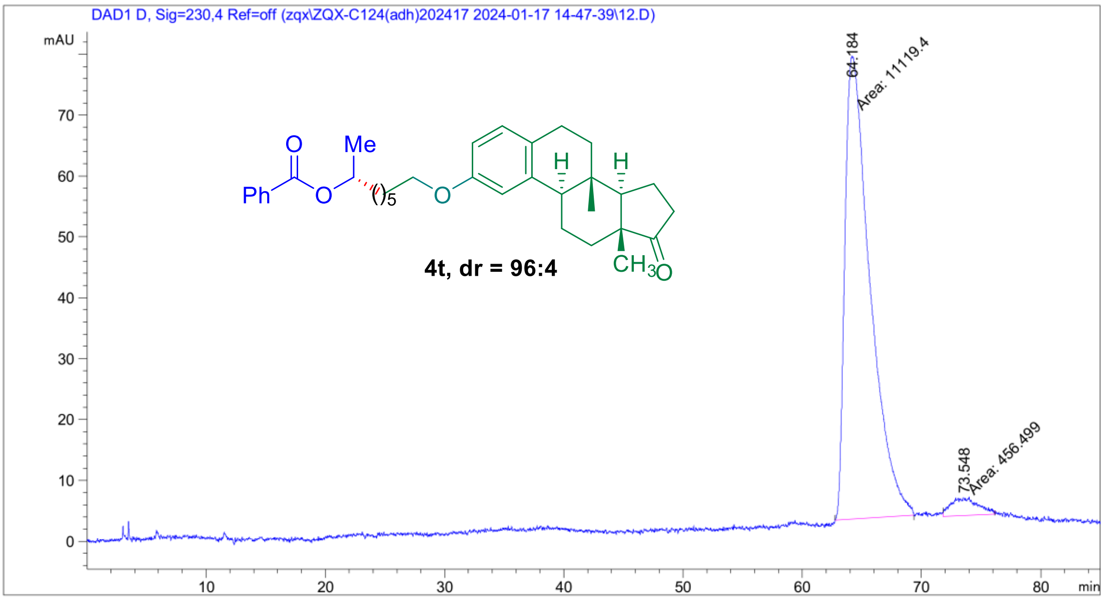

Peak #	RetTime [min]	Type	Width [min]	Area [mAU*s]	Height [mAU]	Area %
1	64.184	PP	2.4363	1.11194e4	76.06777	96.0565
2	73.548	MM	2.5489	456.49933	2.98493	3.9435

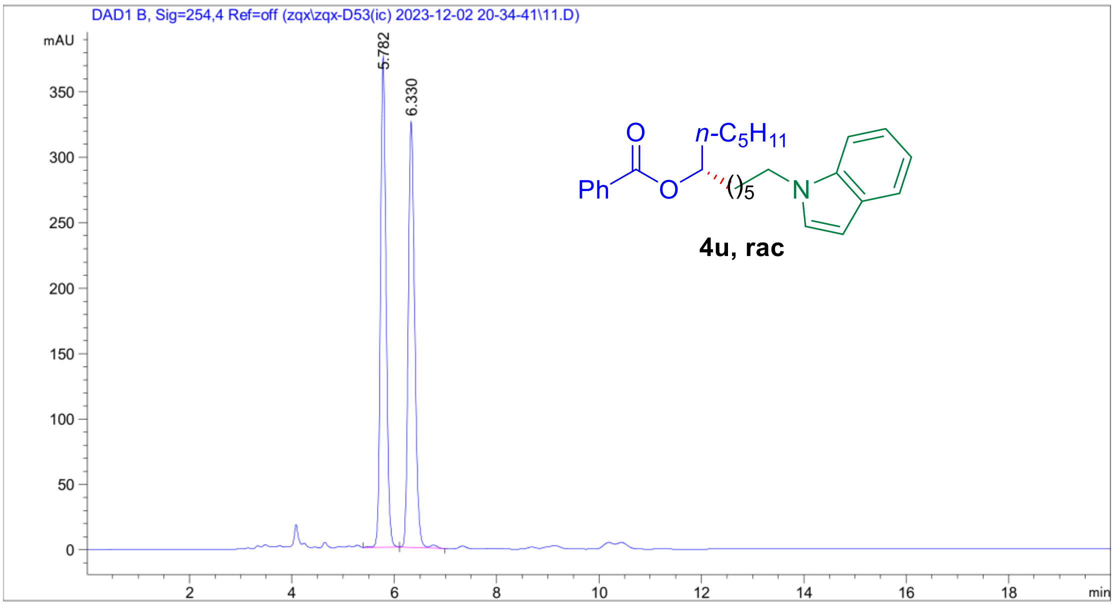

Peak #	RetTime [min]	Type	Width [min]	Area [mAU*s]	Height [mAU]	Area %
1	5.782	VB R	0.1183	2840.50903	375.11716	49.9128
2	6.330	BV R	0.1331	2850.43066	326.42410	50.0872

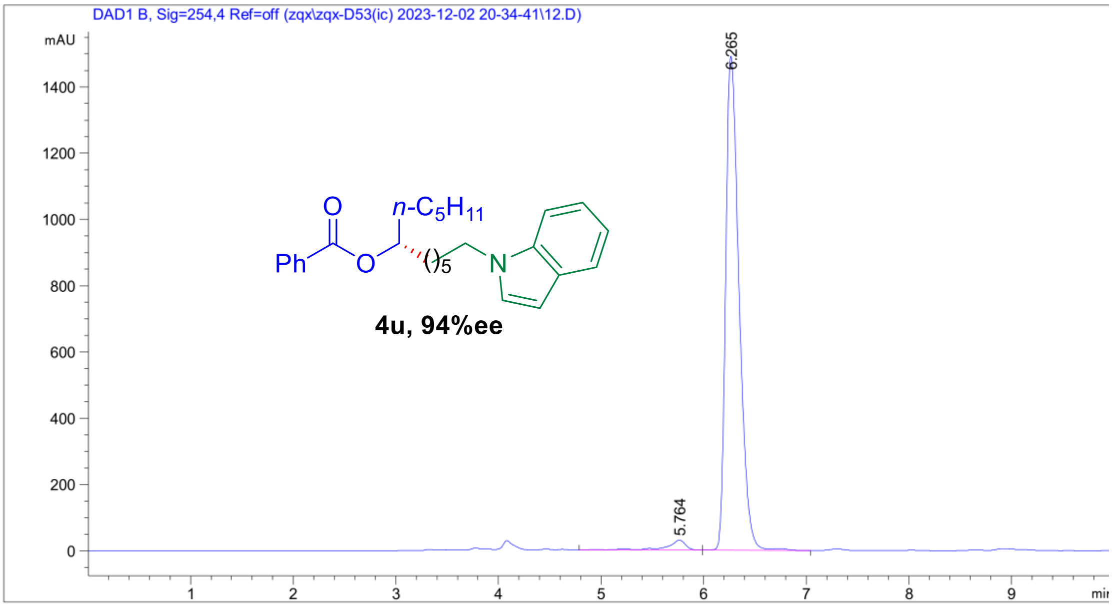

Peak #	RetTime [min]	Type	Width [min]	Area [mAU*s]	Height [mAU]	Area %
1	5.764	VB R	0.1579	422.25510	30.80956	3.0167
2	6.265	BV R	0.1395	1.35749e4	1493.13171	96.9833

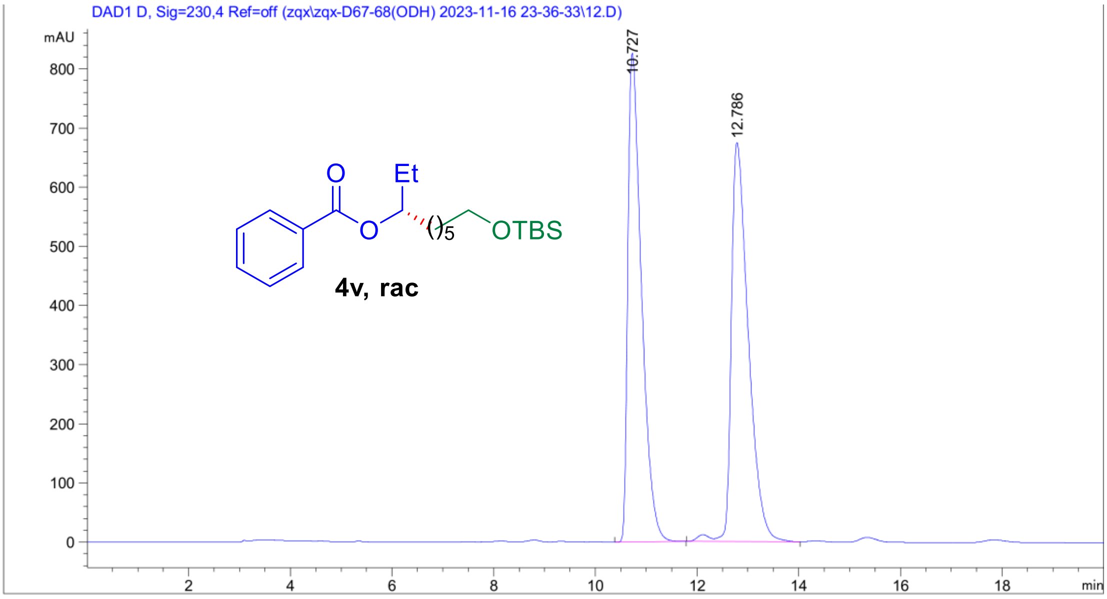

Peak #	RetTime [min]	Type	Width [min]	Area [mAU*s]	Height [mAU]	Area %
1	10.727	BB	0.2817	1.54152e4	826.27338	49.5146
2	12.786	VB R	0.3474	1.57174e4	674.72528	50.4854

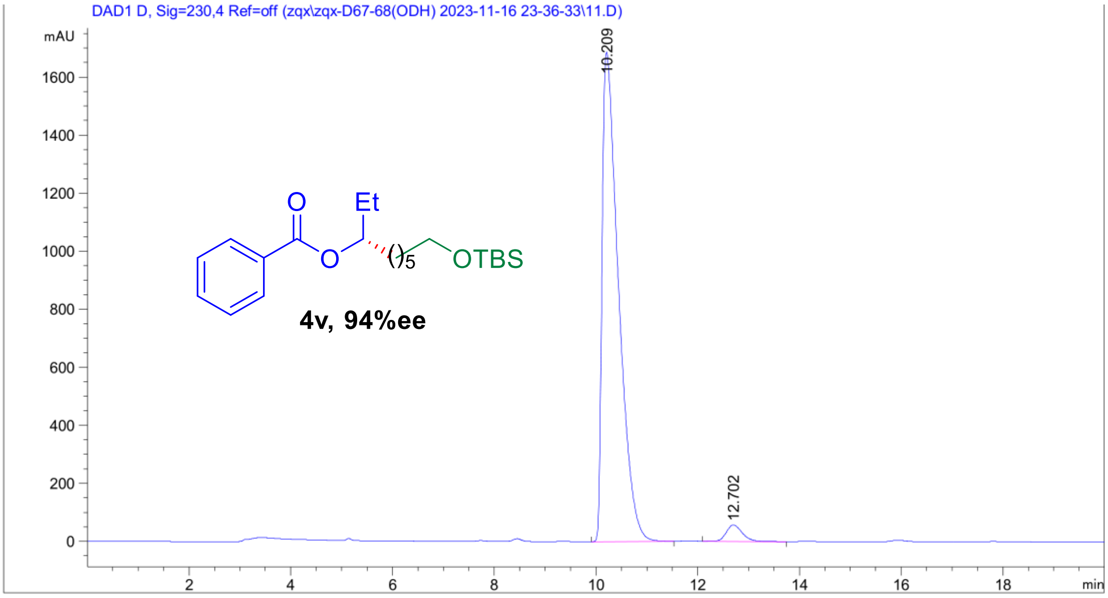

Peak #	RetTime [min]	Type	Width [min]	Area [mAU*s]	Height [mAU]	Area %
1	10.209	BB	0.3349	3.76964e4	1685.81262	96.7406
2	12.702	BB	0.3401	1270.05457	56.97045	3.2594

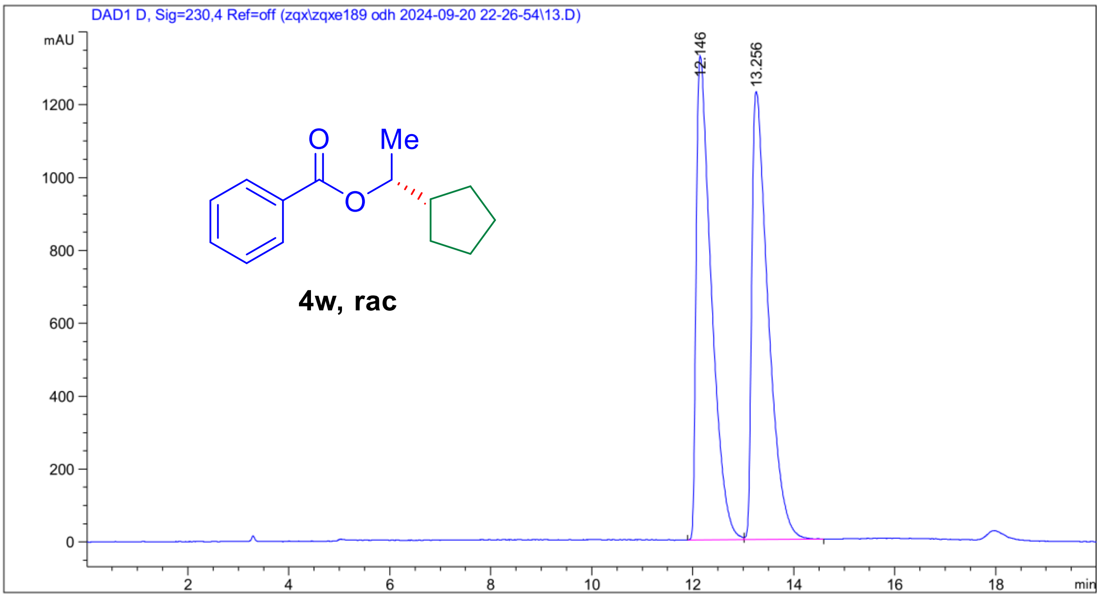

Peak #	RetTime [min]	Type	Width [min]	Area [mAU*s]	Height [mAU]	Area %
1	12.146	BV	0.2890	2.76726e4	1328.63806	49.8590
2	13.256	VV R	0.3140	2.78292e4	1229.78076	50.1410

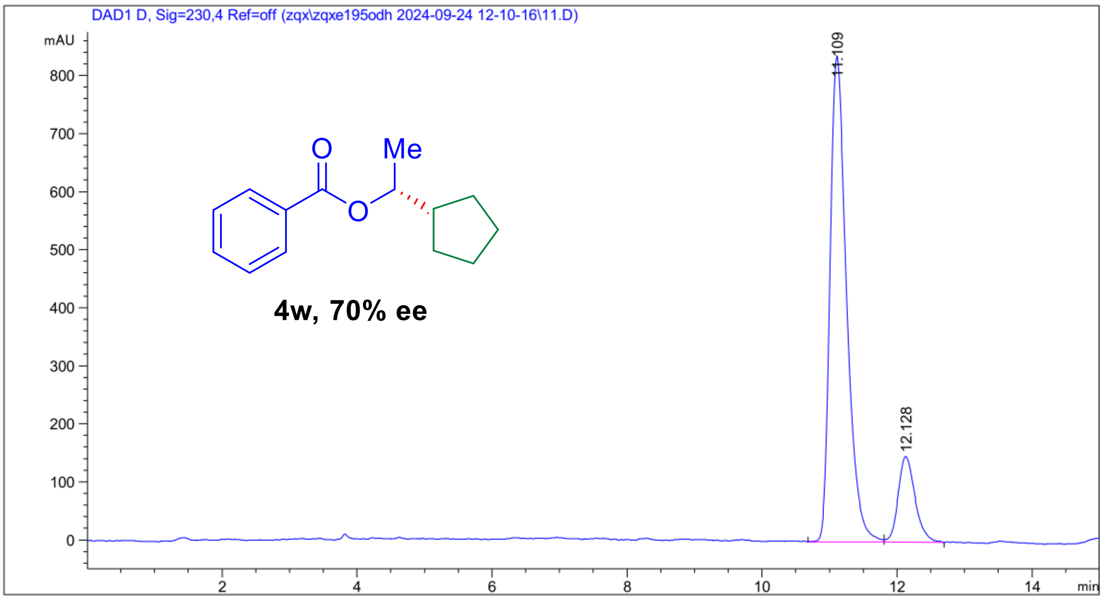

Peak #	RetTime [min]	Type	Width [min]	Area [mAU*s]	Height [mAU]	Area %
1	11.109	BV	0.2610	1.42813e4	837.05273	84.7130
2	12.128	VB	0.2637	2577.15015	147.61203	15.2870

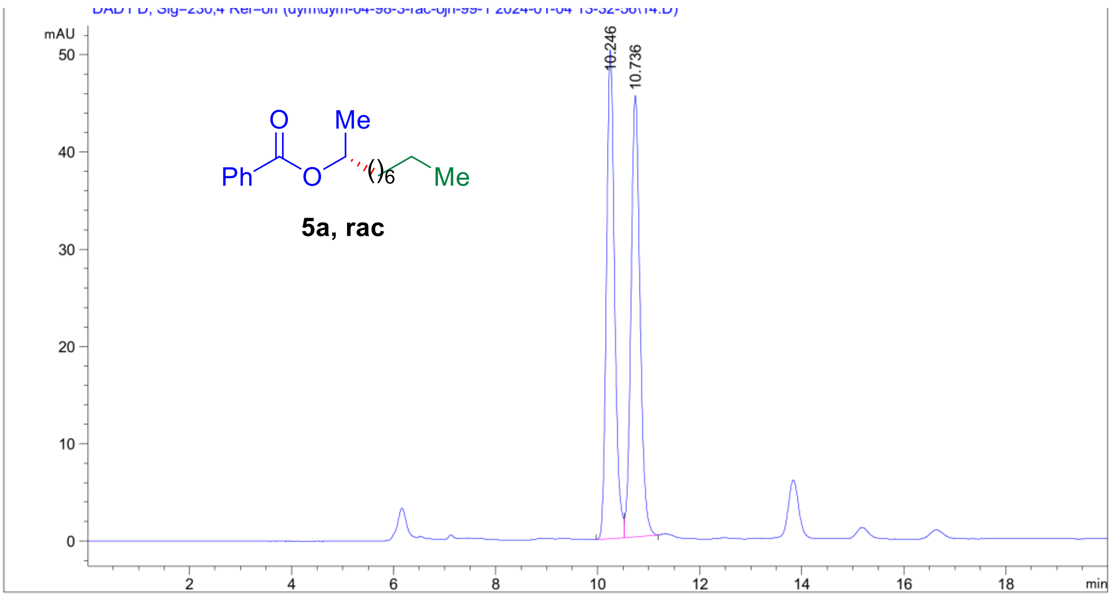

Peak #	RetTime [min]	Type	Width [min]	Area [mAU*s]	Height [mAU]	Area %
1	10.246	BV	0.1707	554.19977	50.24031	49.8230
2	10.736	VB	0.1893	558.13690	45.44393	50.1770

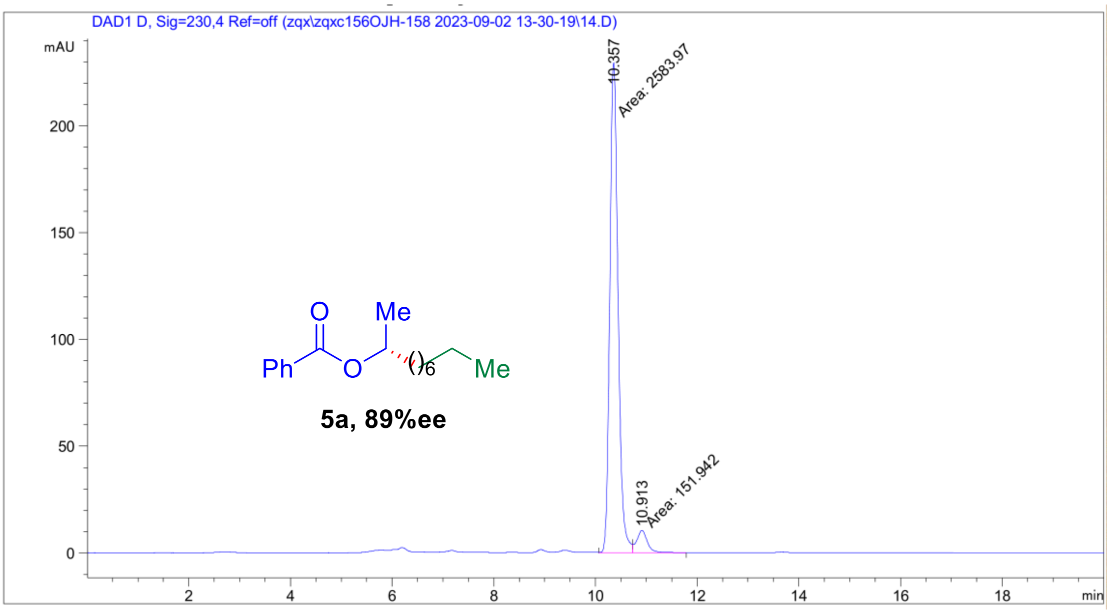

Peak #	RetTime [min]	Type	Width [min]	Area [mAU*s]	Height [mAU]	Area %
1	10.357	MF	0.1876	2583.96606	229.50668	94.4464
2	10.913	FM	0.2445	151.94234	10.35830	5.5536

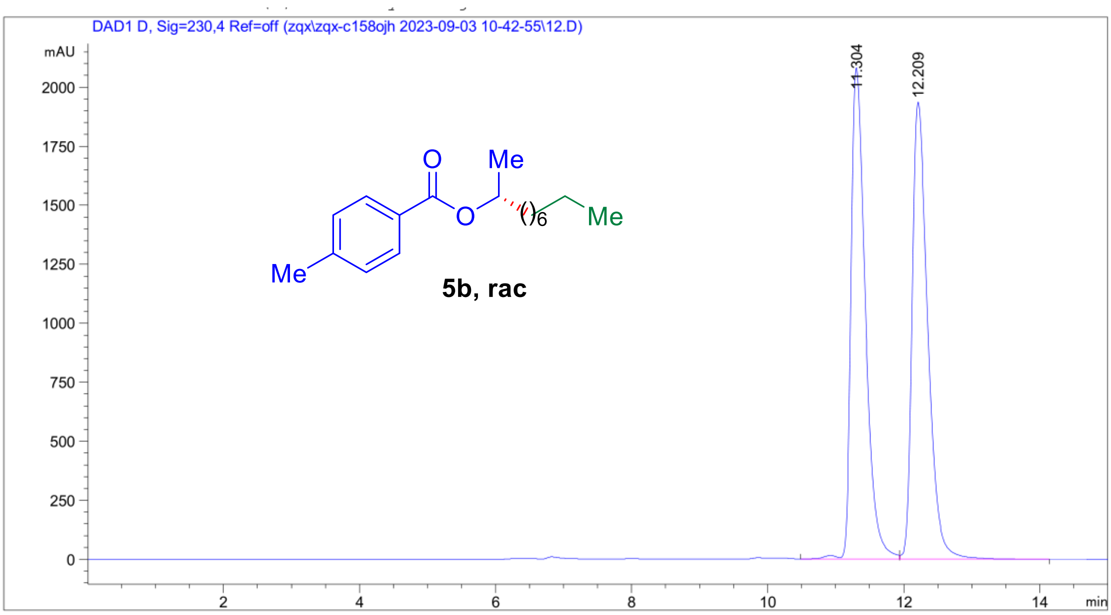

Peak #	RetTime [min]	Type	Width [min]	Area [mAU*s]	Height [mAU]	Area %
1	11.304	VV R	0.2219	3.04918e4	2080.65503	49.6887
2	12.209	VB	0.2441	3.08738e4	1935.90674	50.3113

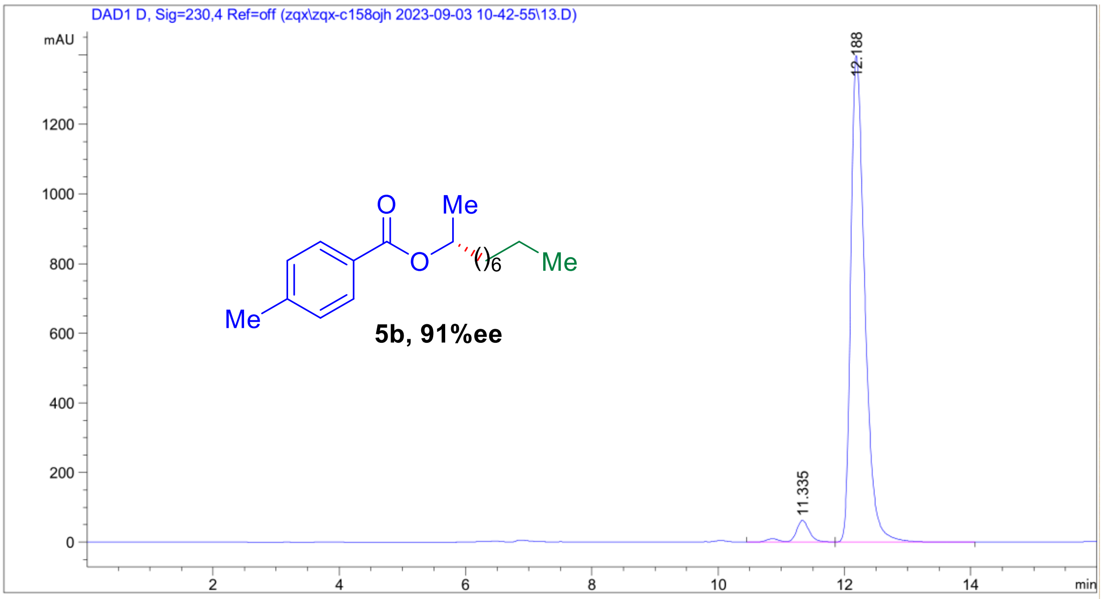

Peak #	RetTime [min]	Type	Width [min]	Area [mAU*s]	Height [mAU]	Area %
1	11.335	VV R	0.2161	1014.33142	62.50002	4.5065
2	12.188	VB	0.2354	2.14941e4	1397.94470	95.4935

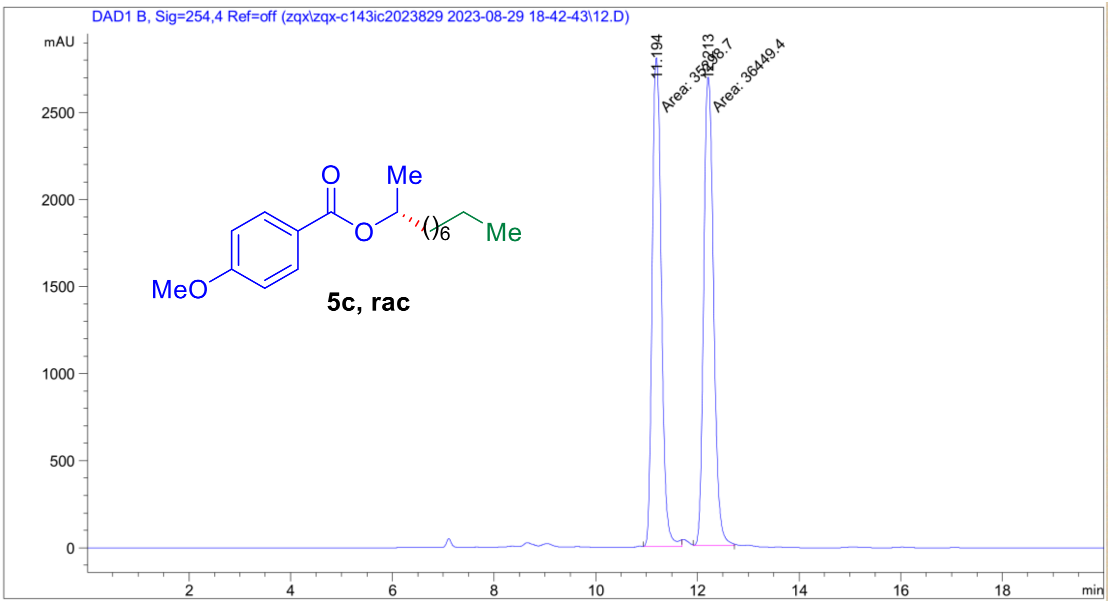

Peak #	RetTime [min]	Type	Width [min]	Area [mAU*s]	Height [mAU]	Area %
1	11.194	MF	0.2096	3.52987e4	2806.46411	49.1981
2	12.213	MM	0.2257	3.64494e4	2692.14185	50.8019

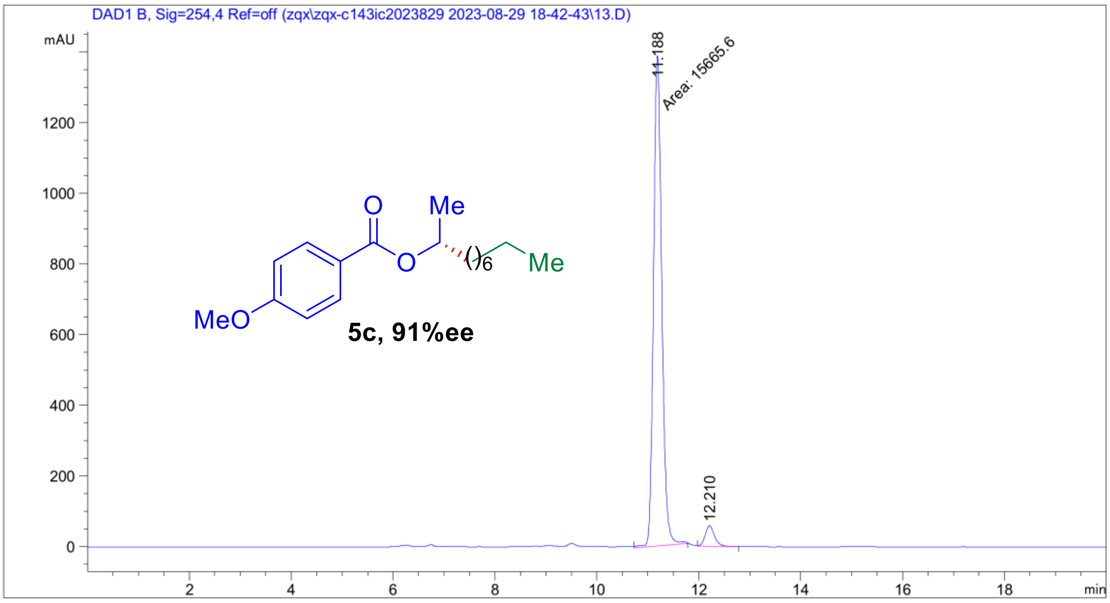

Peak #	RetTime [min]	Type	Width [min]	Area [mAU*s]	Height [mAU]	Area %
1	11.188	MM	0.1880	1.56656e4	1389.09424	95.4072
2	12.210	VB	0.1966	754.12225	58.40374	4.5928

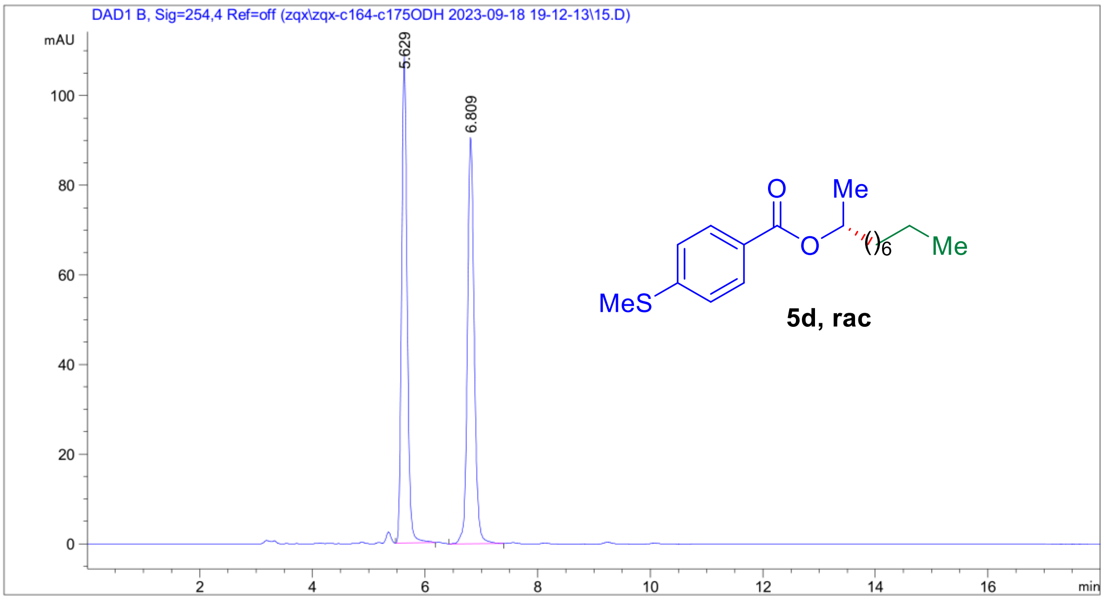

Peak #	RetTime [min]	Type	Width [min]	Area [mAU*s]	Height [mAU]	Area %
1	5.629	BB	0.1056	741.93964	108.73130	49.5484
2	6.809	BB	0.1271	755.46344	90.72363	50.4516

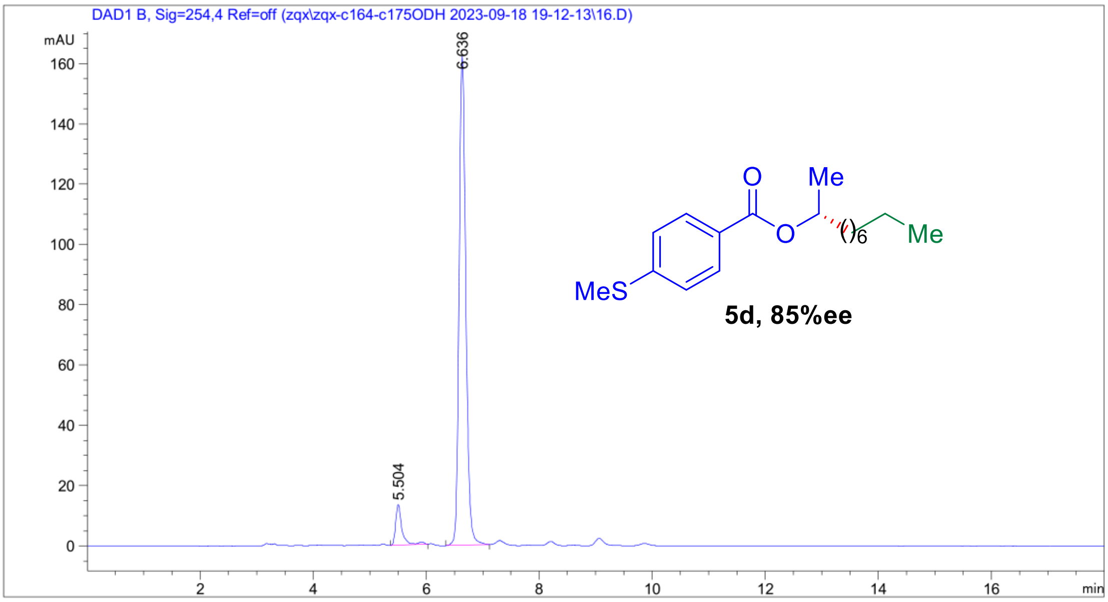

Peak #	RetTime [min]	Type	Width [min]	Area [mAU*s]	Height [mAU]	Area %
1	5.504	BV R	0.1154	106.34048	13.57595	7.3225
2	6.636	BB	0.1267	1345.90393	162.33234	92.6775

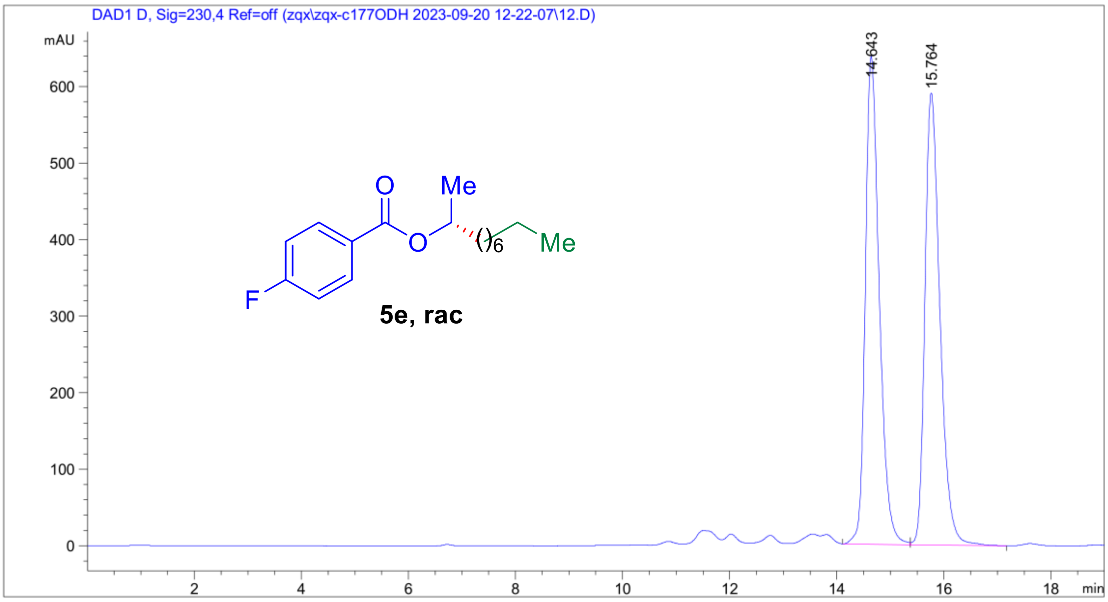

Peak #	RetTime [min]	Type	Width [min]	Area [mAU*s]	Height [mAU]	Area %
1	14.643	BV	0.2747	1.15244e4	638.12427	50.0639
2	15.764	VB	0.2990	1.14950e4	590.66107	49.9361

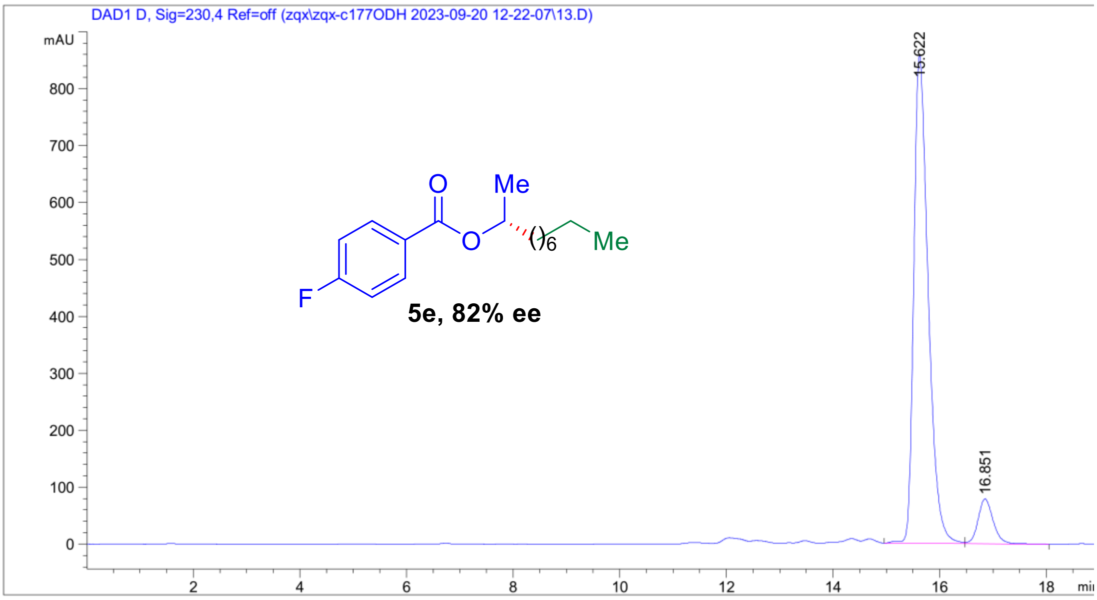

Peak #	RetTime [min]	Type	Width [min]	Area [mAU*s]	Height [mAU]	Area %
1	15.622	VV R	0.2869	1.61102e4	856.22772	91.1592
2	16.851	VB	0.3034	1562.39856	78.78352	8.8408

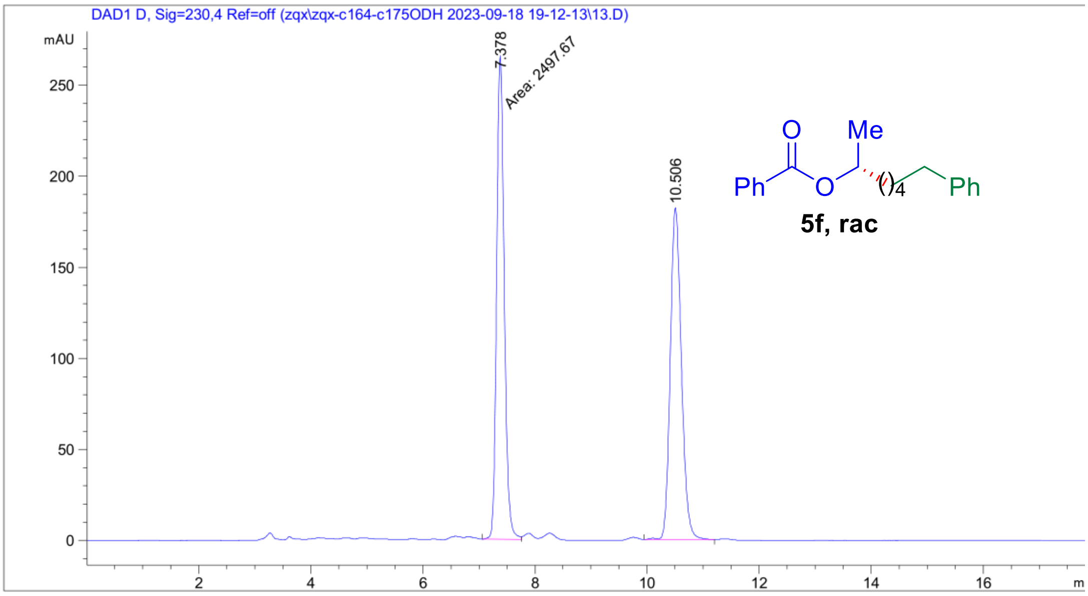

Peak #	RetTime [min]	Type	Width [min]	Area [mAU*s]	Height [mAU]	Area %
1	7.378	MF	0.1566	2497.66504	265.80887	49.8906
2	10.506	VB R	0.2126	2508.61865	182.19936	50.1094

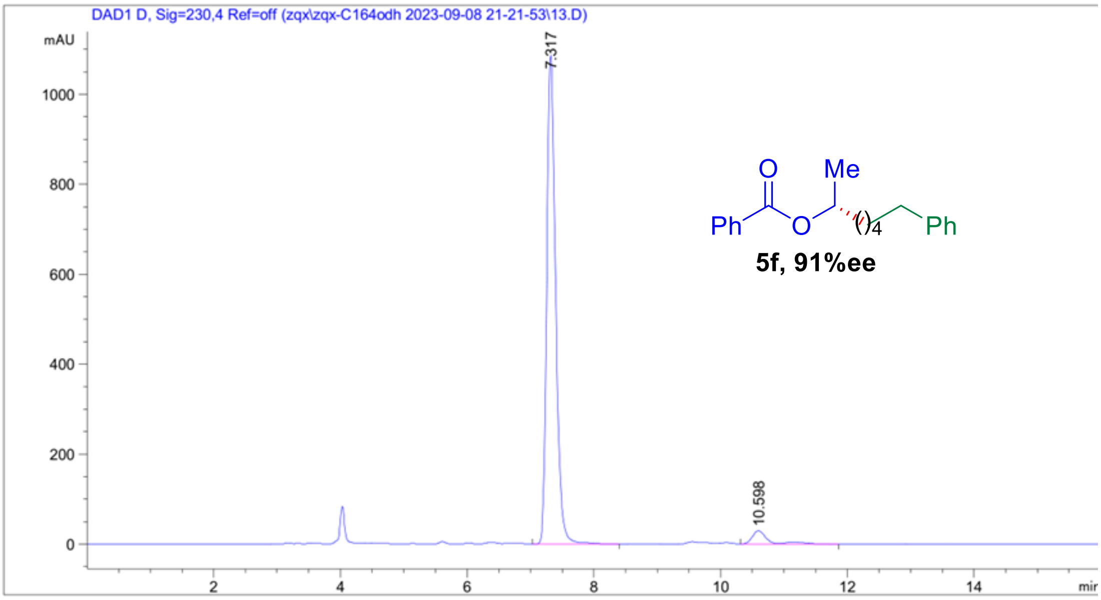

Peak #	RetTime [min]	Type	Width [min]	Area [mAU*s]	Height [mAU]	Area %
1	7.317	BB	0.1520	1.06062e4	1085.63440	95.3283
2	10.598	BV R	0.2196	519.76843	29.51682	4.6717

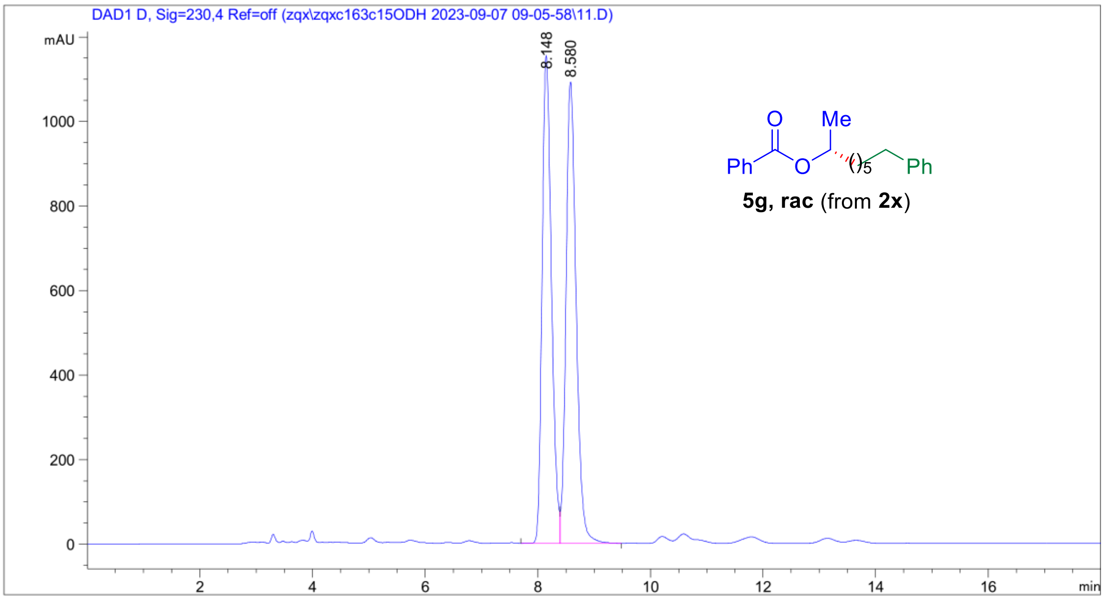

Peak #	RetTime [min]	Type	Width [min]	Area [mAU*s]	Height [mAU]	Area %
1	8.148	VV R	0.1781	1.32502e4	1153.12463	49.1386
2	8.580	VB	0.1925	1.37147e4	1091.87024	50.8614

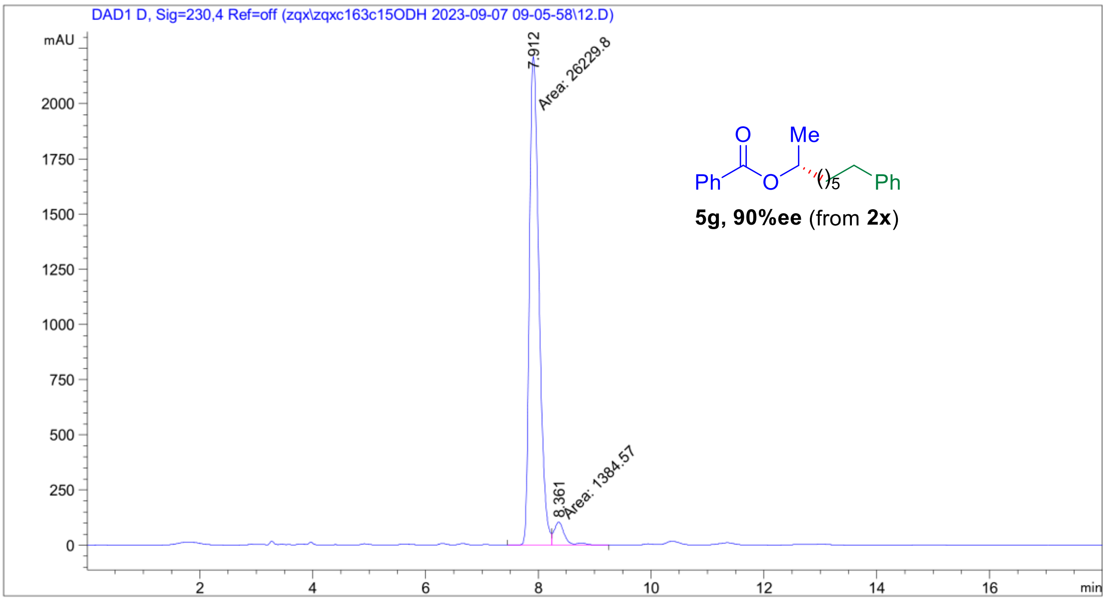

Peak #	RetTime [min]	Type	Width [min]	Area [mAU*s]	Height [mAU]	Area %
1	7.912	MF	0.1972	2.62298e4	2216.94775	94.9861
2	8.361	FM	0.2202	1384.56897	104.81065	5.0139

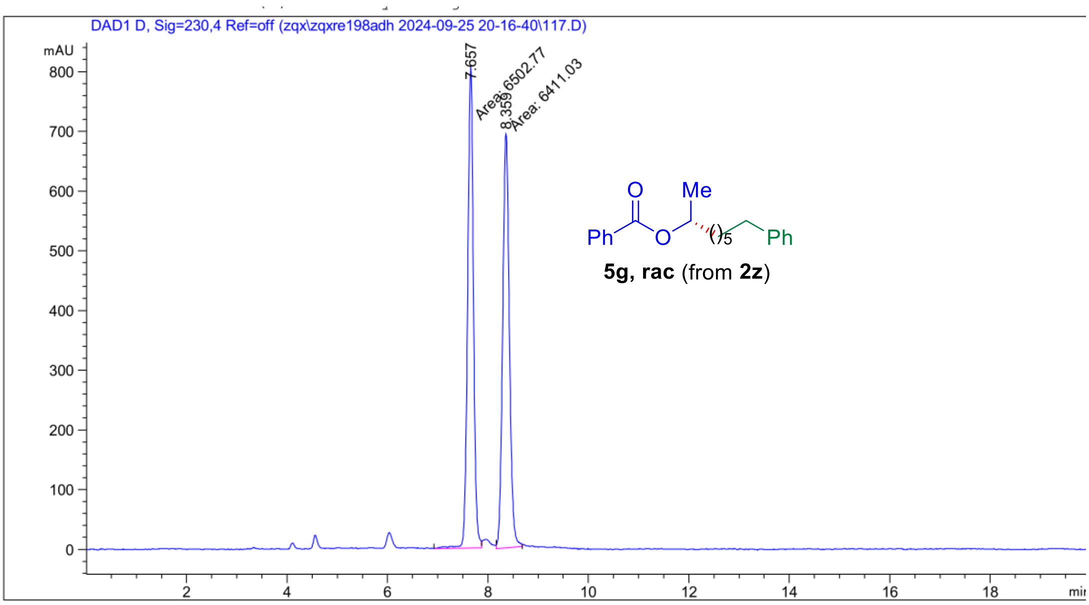

Peak #	RetTime [min]	Type	Width [min]	Area [mAU*s]	Height [mAU]	Area %
1	7.657	MF	0.1343	6502.76904	807.24689	50.3552
2	8.359	MM	0.1541	6411.03223	693.41473	49.6448

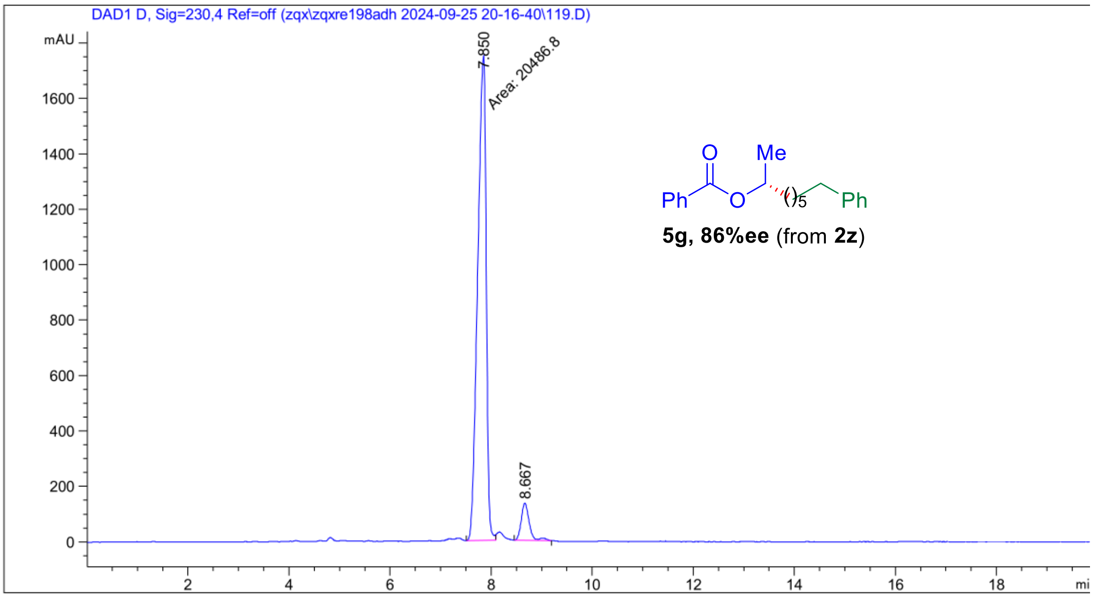

Peak #	RetTime [min]	Type	Width [min]	Area [mAU*s]	Height [mAU]	Area %
1	7.850	MF	0.1950	2.04868e4	1750.98169	93.1714
2	8.667	BV R	0.1625	1501.50171	134.39583	6.8286

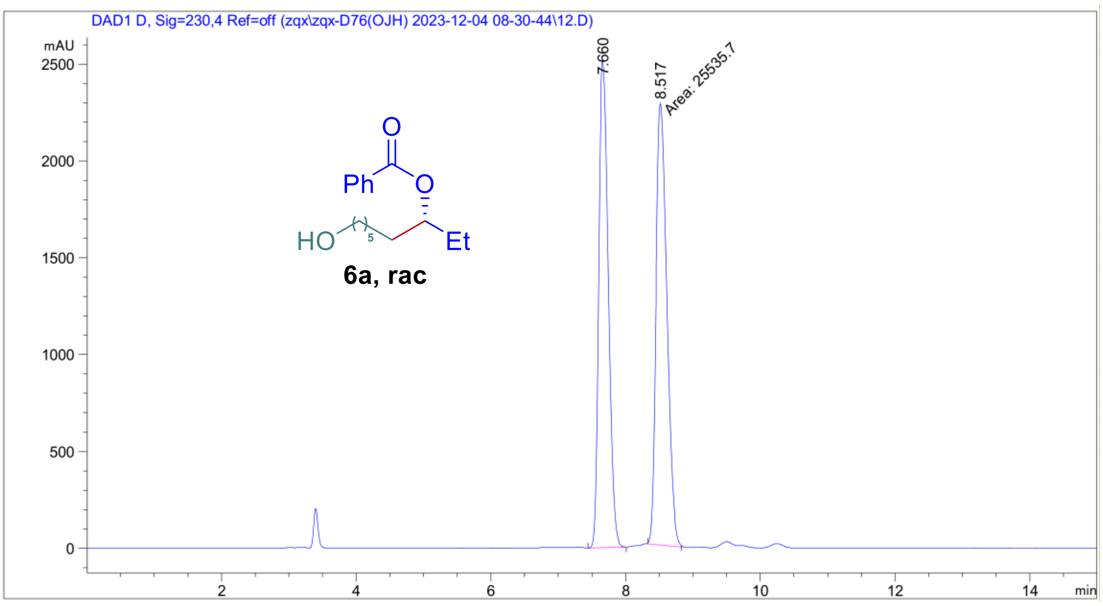

Peak #	RetTime [min]	Type	Width [min]	Area [mAU*s]	Height [mAU]	Area %
1	7.660	BB	0.1562	2.50214e4	2512.49561	49.4913
2	8.517	MM	0.1866	2.55357e4	2281.12036	50.5087

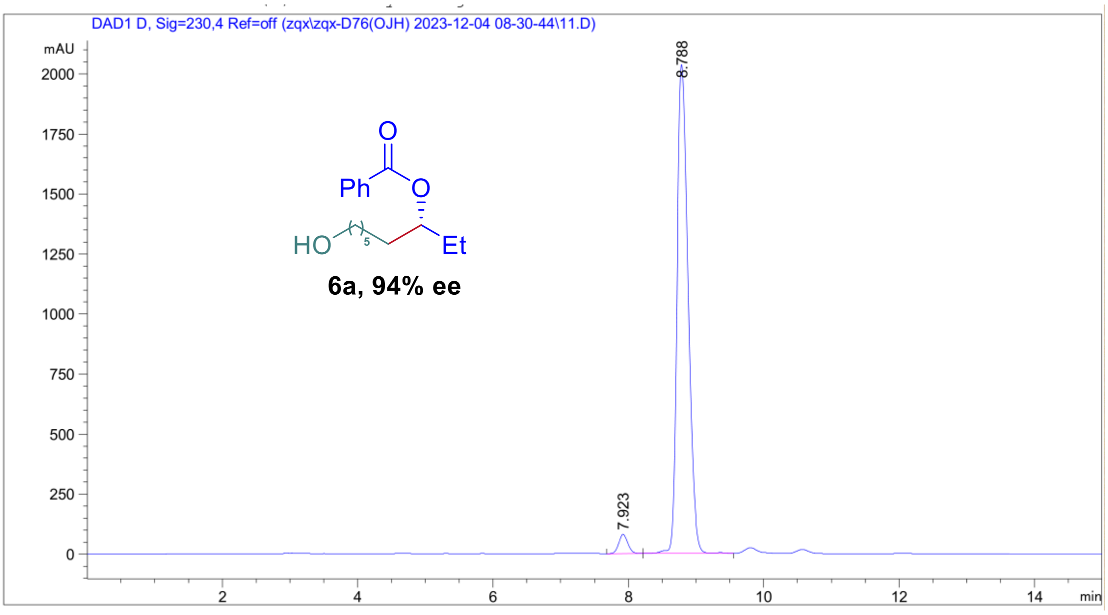

Peak #	RetTime [min]	Type	Width [min]	Area [mAU*s]	Height [mAU]	Area %
1	7.923	BB	0.1480	759.67529	80.60741	3.1837
2	8.788	BV R	0.1760	2.31020e4	2036.51660	96.8163

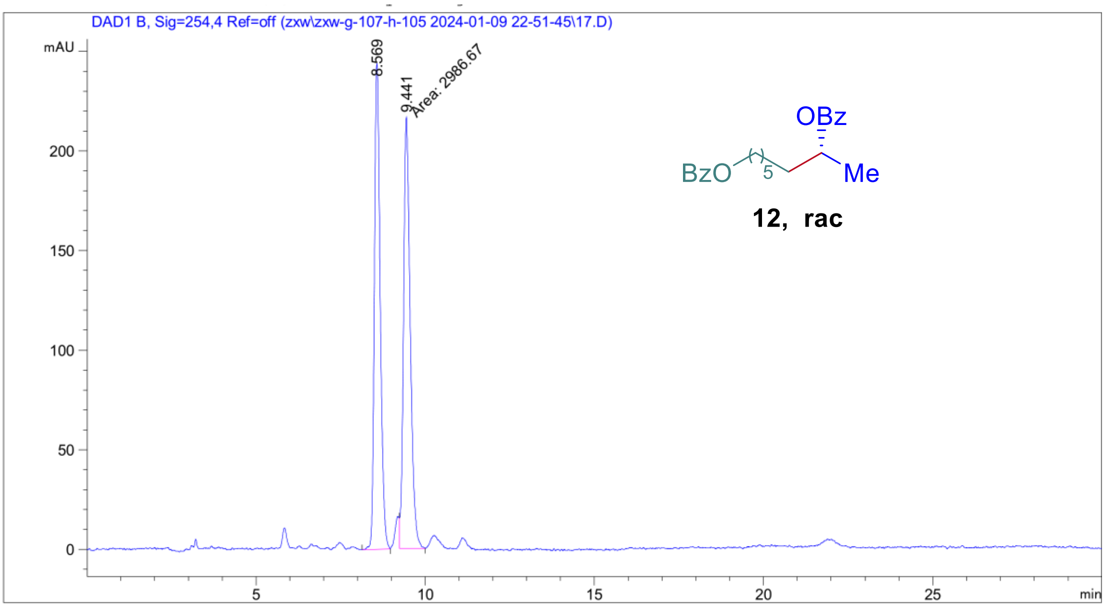

Peak #	RetTime [min]	Type	Width [min]	Area [mAU*s]	Height [mAU]	Area %
1	8.569	VB R	0.1812	2910.90552	244.02902	49.3577
2	9.441	FM	0.2297	2986.67065	216.69519	50.6423

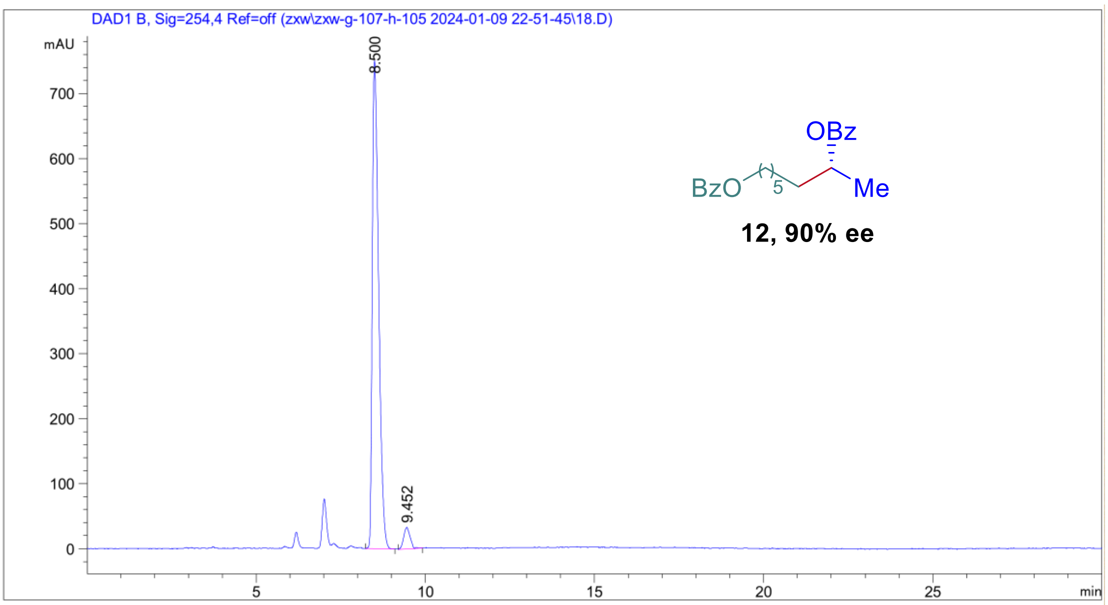

Peak #	RetTime [min]	Type	Width [min]	Area [mAU*s]	Height [mAU]	Area %
1	8.500	BB	0.1912	9608.43262	751.49756	95.6980
2	9.452	BV R	0.1995	431.93835	33.10950	4.3020

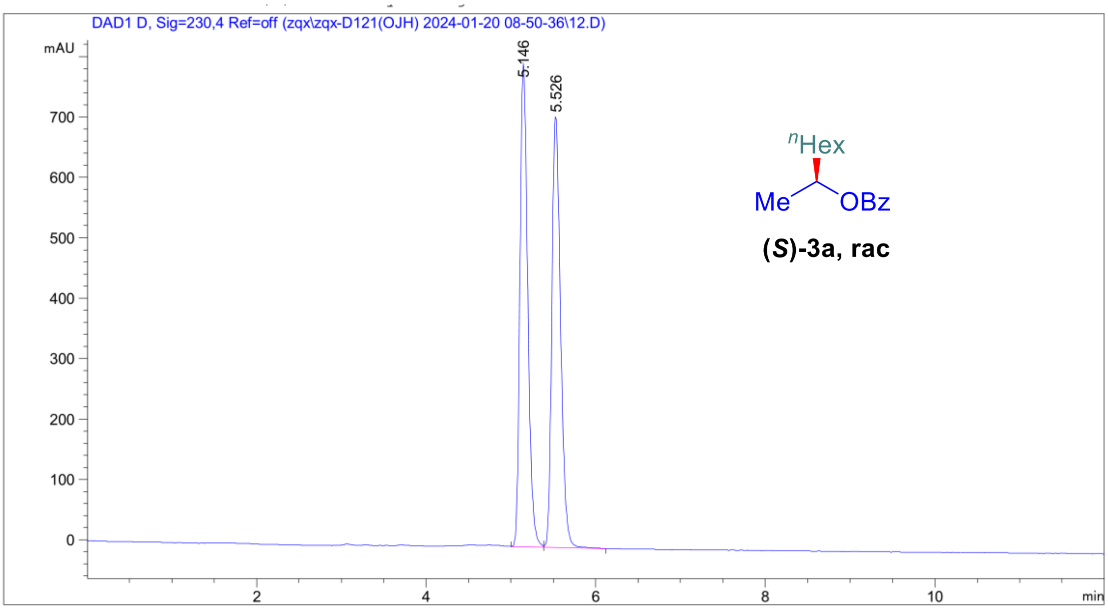

Peak #	RetTime [min]	Type	Width [min]	Area [mAU*s]	Height [mAU]	Area %
1	5.146	BV	0.0987	5116.66504	799.57074	50.1186
2	5.526	VB	0.1113	5092.44385	713.08191	49.8814

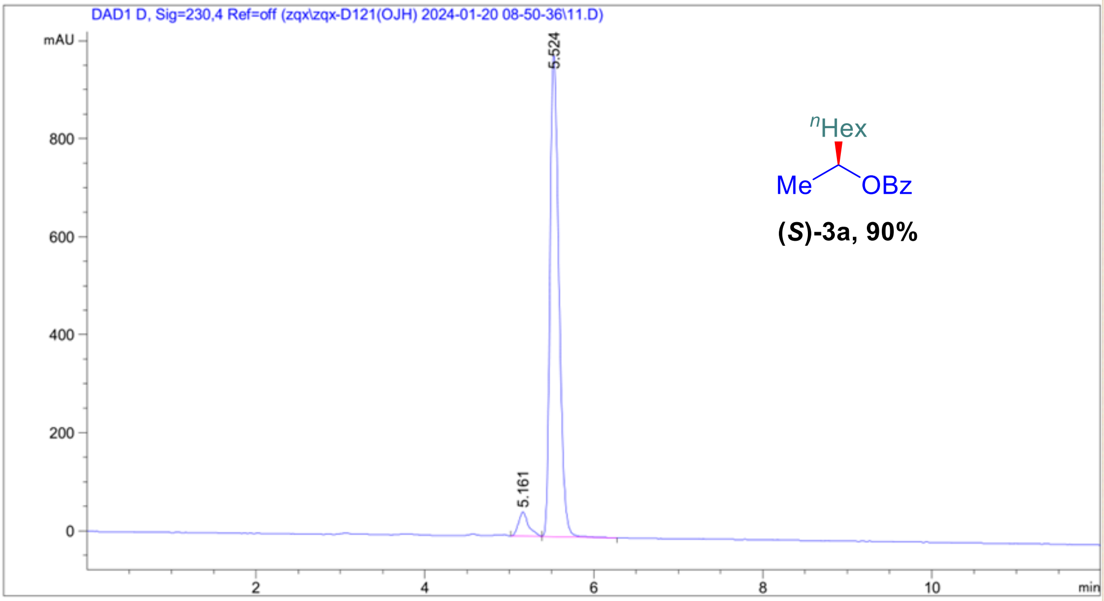

Peak #	RetTime [min]	Type	Width [min]	Area [mAU*s]	Height [mAU]	Area %
1	5.161	BV	0.1220	405.52286	49.30651	5.2872
2	5.524	VB	0.1162	7264.31885	983.73663	94.7128

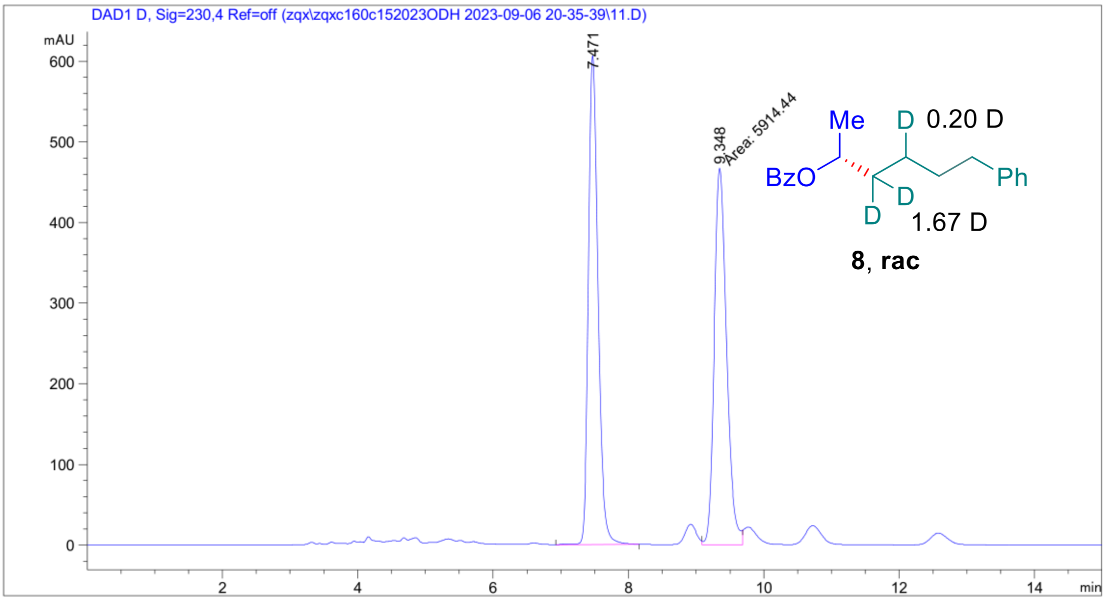

8, **rac**

Peak #	RetTime [min]	Type	Width [min]	Area [mAU*s]	Height [mAU]	Area %
1	7.471	BB	0.1503	5943.41553	606.42102	50.1222
2	9.348	FM	0.2111	5914.43799	466.94641	49.8778

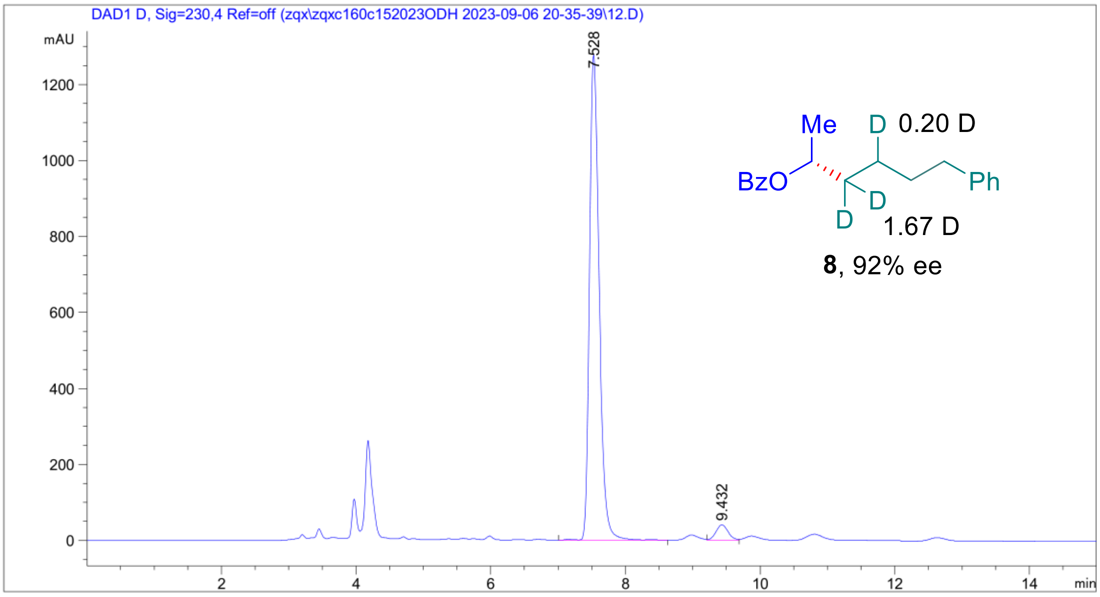

8, 92% ee

Peak #	RetTime [min]	Type	Width [min]	Area [mAU*s]	Height [mAU]	Area %
1	7.528	VV R	0.1534	1.26723e4	1276.64270	96.1356
2	9.432	VV	0.1948	509.39249	40.48809	3.8644

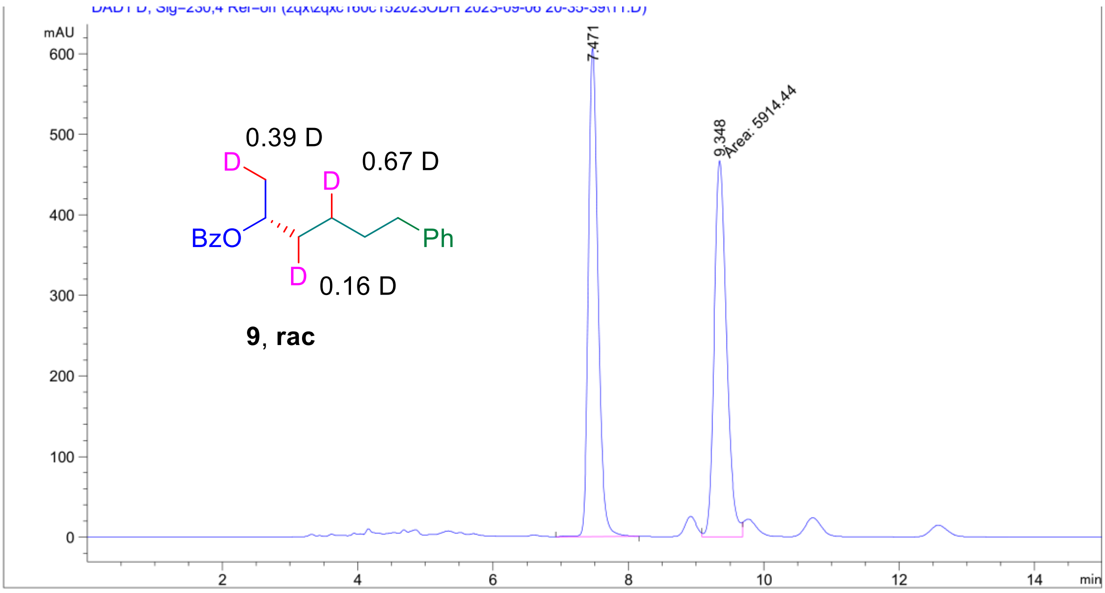

Peak #	RetTime [min]	Type	Width [min]	Area [mAU*s]	Height [mAU]	Area %
1	7.471	BB	0.1503	5943.41553	606.42102	50.1222
2	9.348	FM	0.2111	5914.43799	466.94641	49.8778

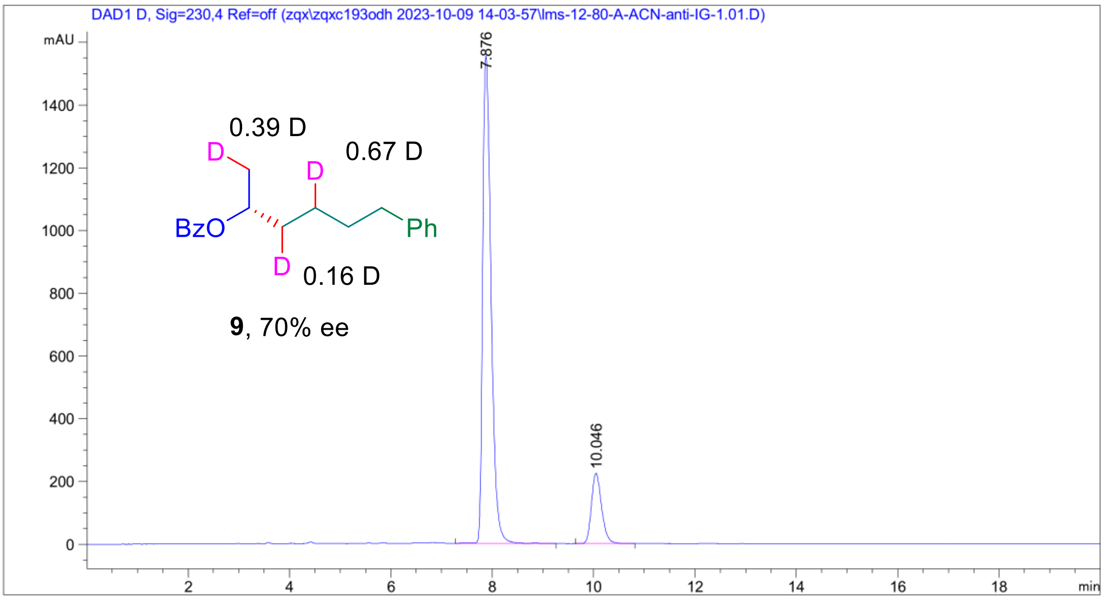

Peak #	RetTime [min]	Type	Width [min]	Area [mAU*s]	Height [mAU]	Area %
1	7.876	VV R	0.1801	1.79070e4	1554.86169	85.1473
2	10.046	BB	0.2143	3123.62109	224.54547	14.8527

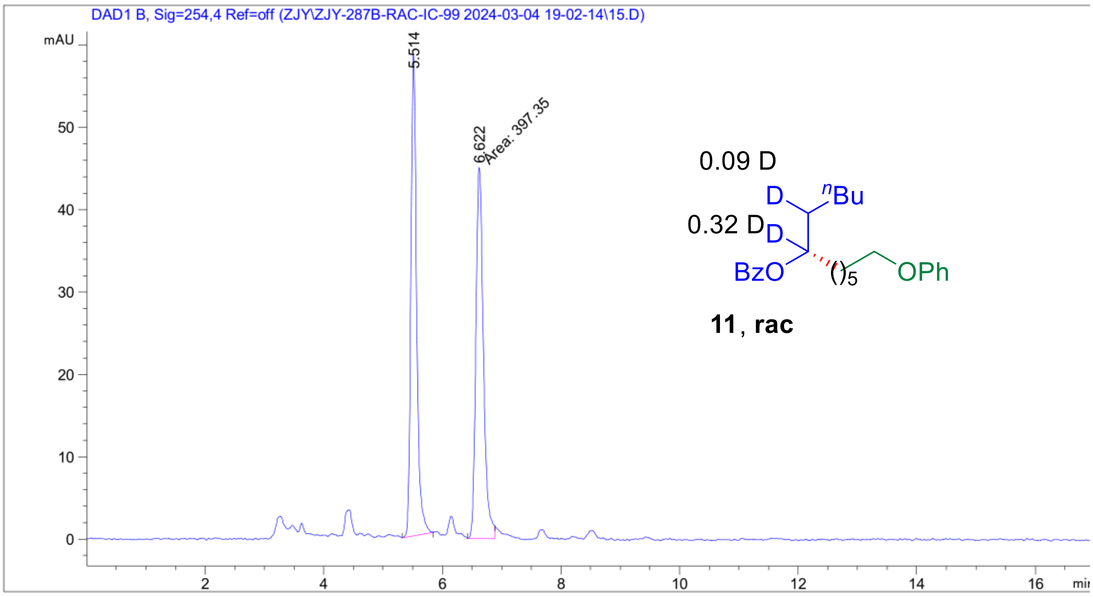

Peak #	RetTime [min]	Type	Width [min]	Area [mAU*s]	Height [mAU]	Area %
1	5.514	BB	0.1024	392.98993	58.48817	49.7242
2	6.622	MF	0.1469	397.34961	45.09466	50.2758

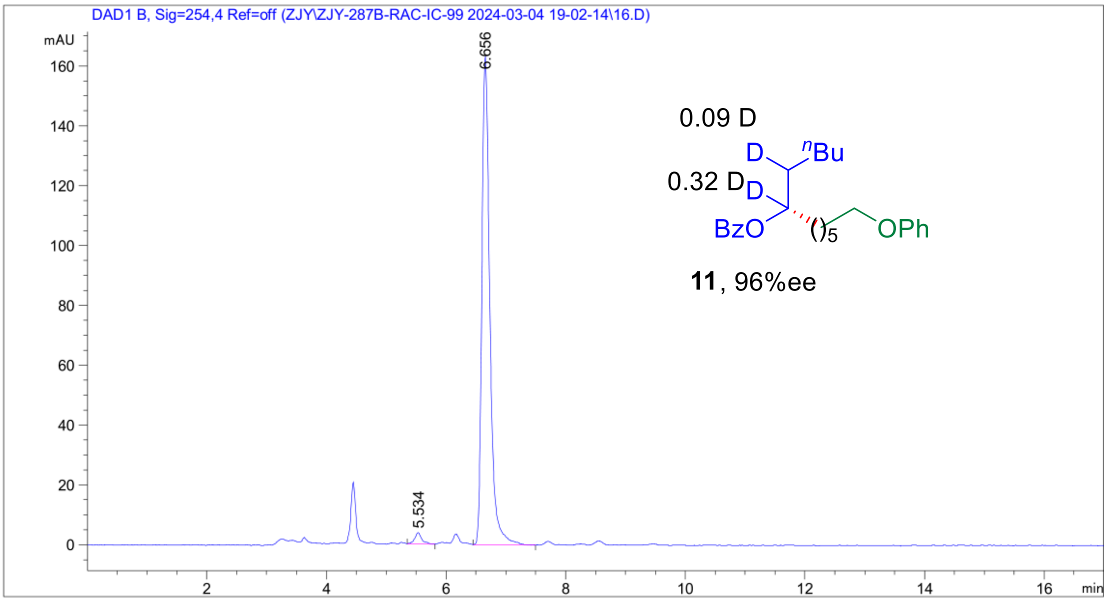

Peak #	RetTime [min]	Type	Width [min]	Area [mAU*s]	Height [mAU]	Area %
1	5.534	BB	0.1186	30.03330	3.70351	1.9539
2	6.656	BB	0.1417	1507.09363	163.22929	98.0461
